# Supplementary figures and images for: A pre-screening strategy to assess resected tumor margins by imaging cytoplasmic viscosity and hypoxia (part 1 of 3)
Source: eLife. 2021 Oct 11;10:e70471. doi: 10.7554/eLife.70471 (PMC8553343; doi:10.7554/eLife.70471)

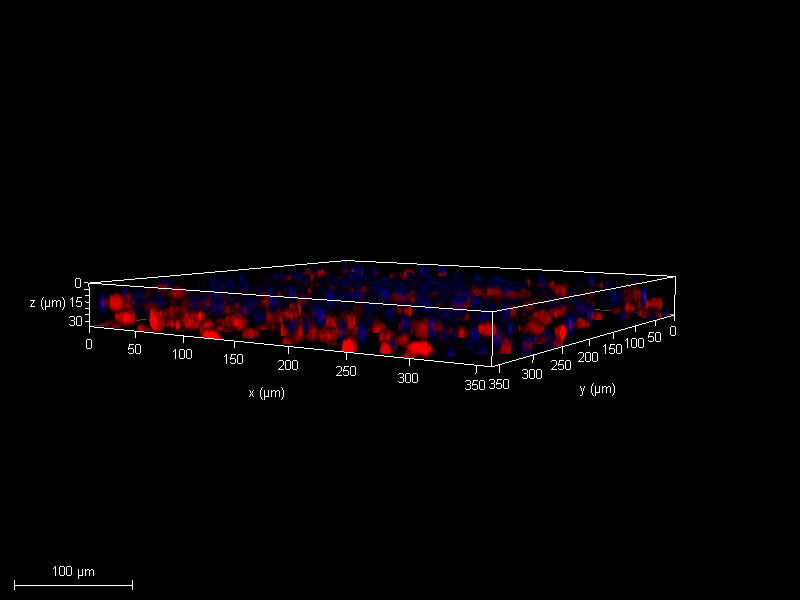

Supplement: Figure 2—source data 1. [file elife-70471-fig2-data1.zip › Figure 2-Source data/Raw data-tumor 3D reconstruction in Figure 2B.tif]

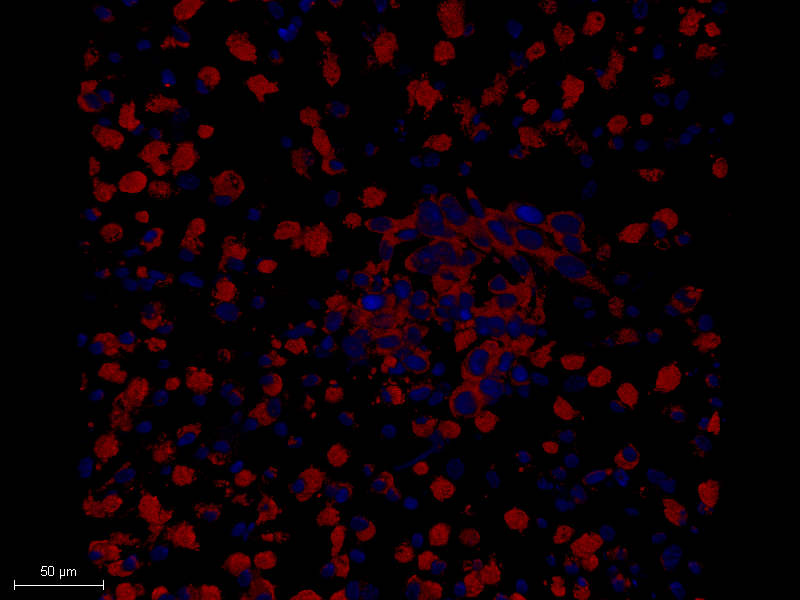

Supplement: Figure 2—source data 1. [file elife-70471-fig2-data1.zip › Figure 2-Source data/Raw data-tumor max merge image in Figure 2B.tif]

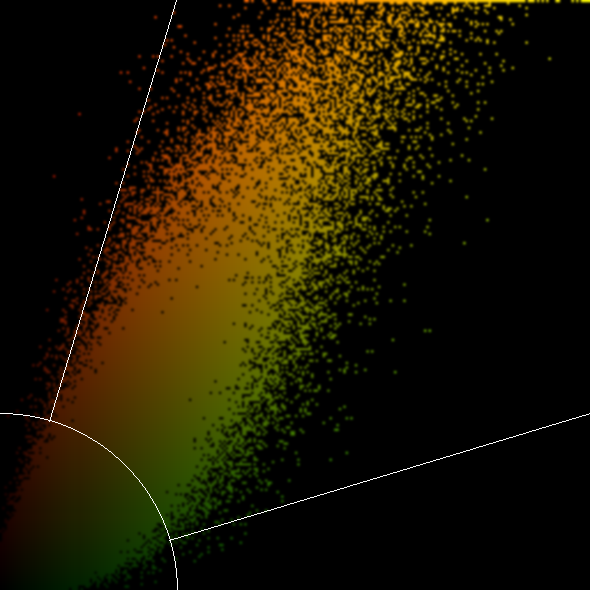

Supplement: Figure 2—source data 1. [file elife-70471-fig2-data1.zip › Figure 2-Source data/FaDu cells/Raw data-Colocalization in Figure 2A.tif]

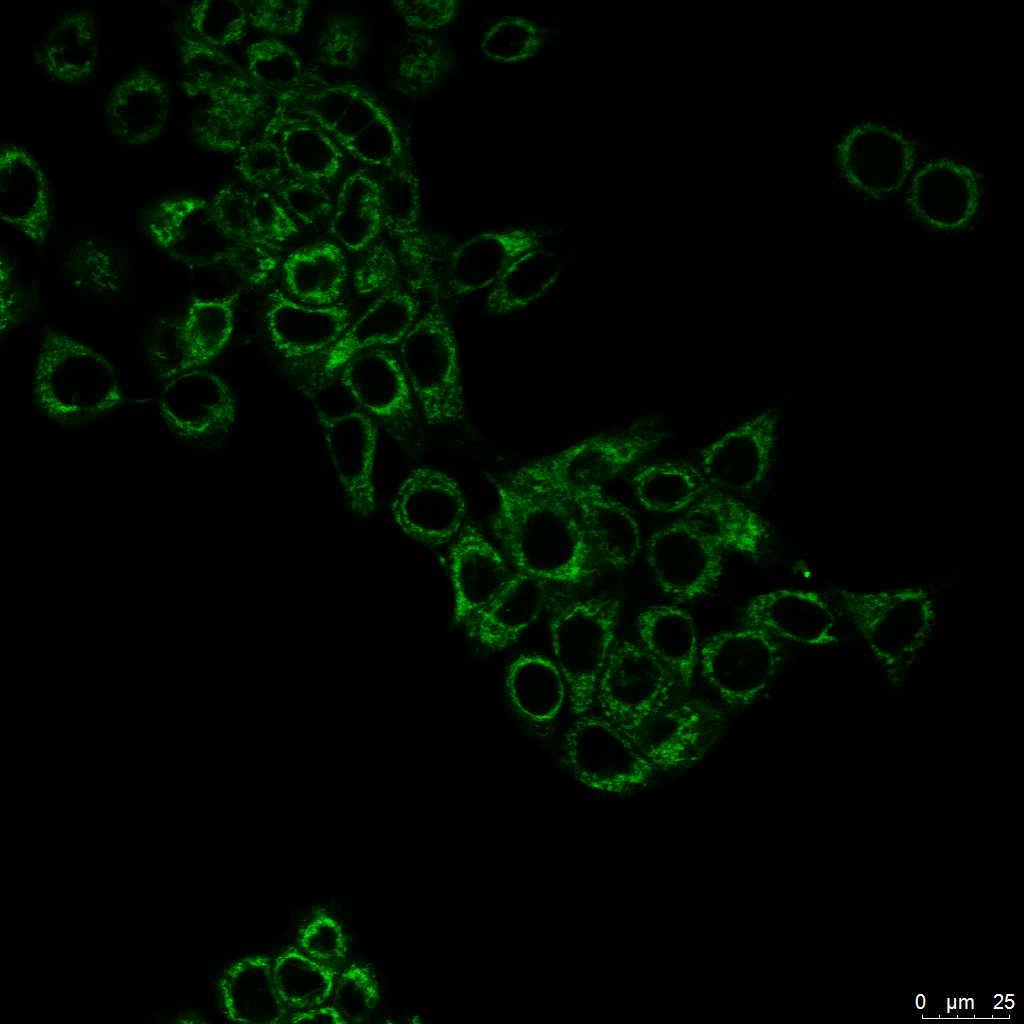

Supplement: Figure 2—source data 1. [file elife-70471-fig2-data1.zip › Figure 2-Source data/FaDu cells/Raw data-Mito Tracker imaging in Figure 2A.tif]

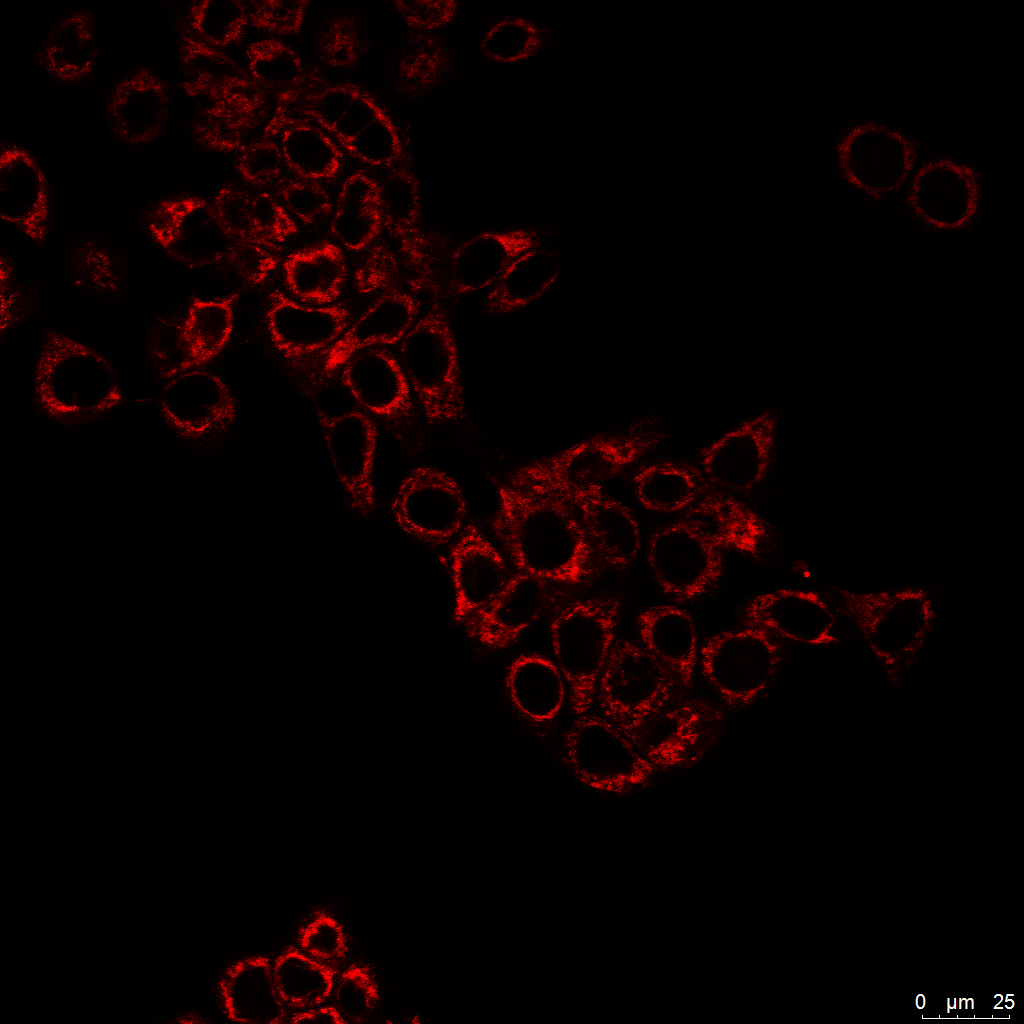

Supplement: Figure 2—source data 1. [file elife-70471-fig2-data1.zip › Figure 2-Source data/FaDu cells/Raw data-IBS440 imaging in Figure 2A.tif]

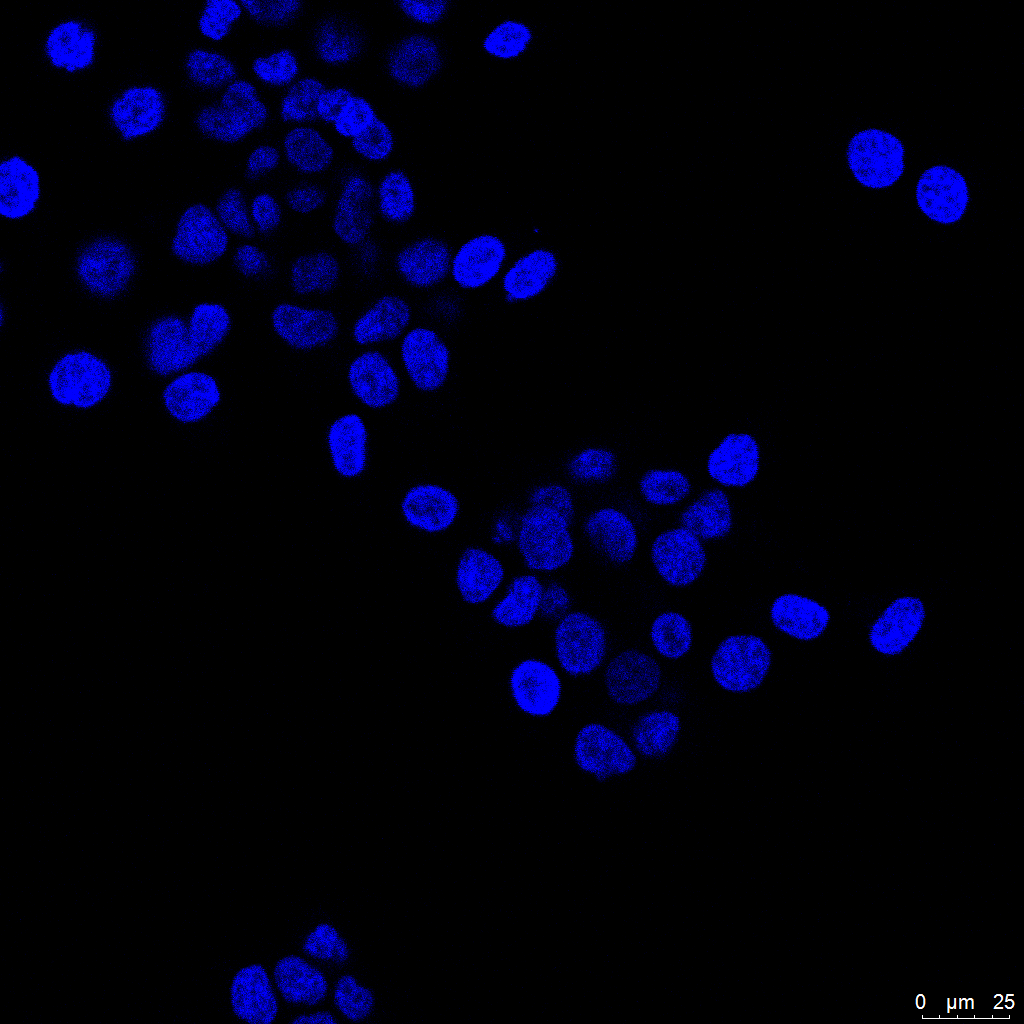

Supplement: Figure 2—source data 1. [file elife-70471-fig2-data1.zip › Figure 2-Source data/FaDu cells/Raw data-Hoechst imaging in Figure 2A.tif]

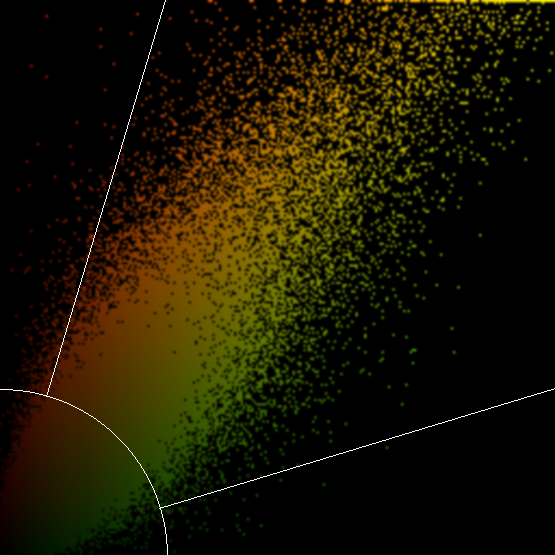

Supplement: Figure 2—source data 1. [file elife-70471-fig2-data1.zip › Figure 2-Source data/A549 cells/Raw data-Colocalization in Figure 2A.tif]

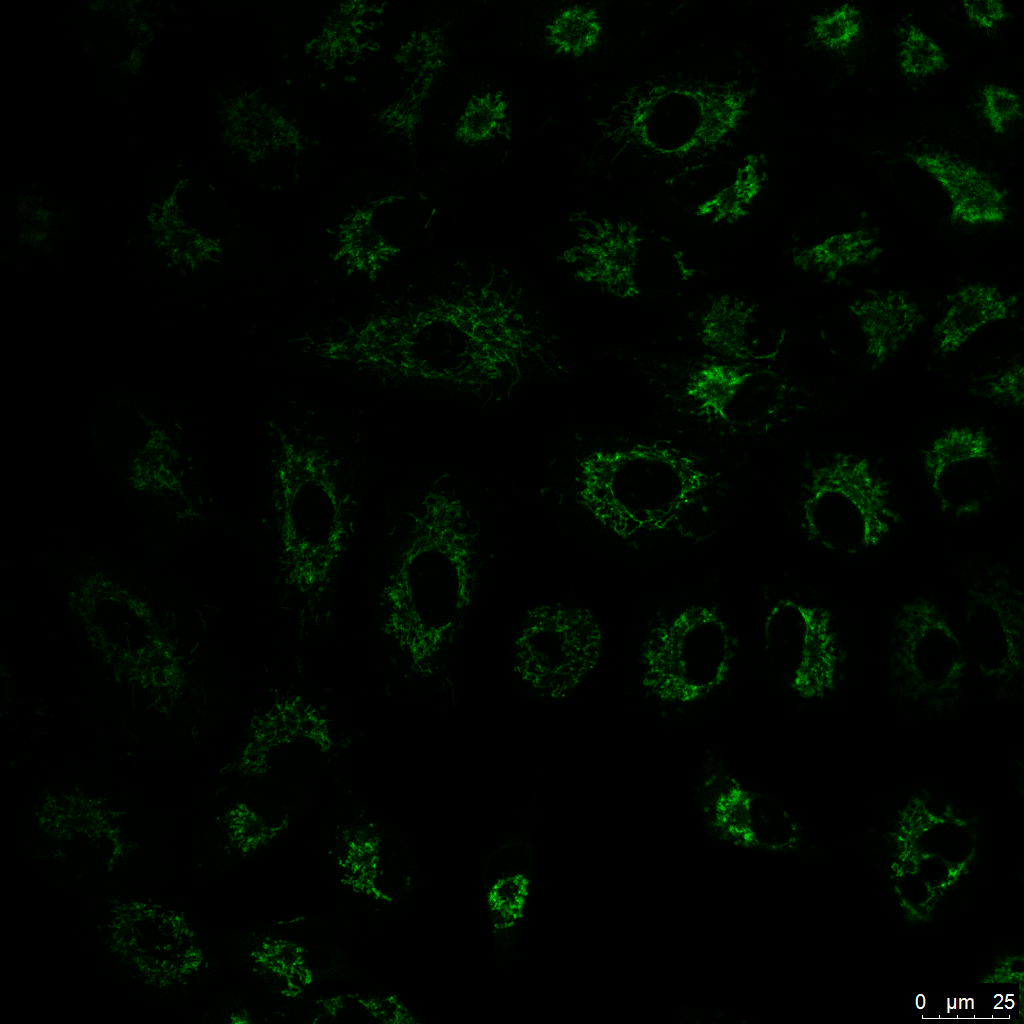

Supplement: Figure 2—source data 1. [file elife-70471-fig2-data1.zip › Figure 2-Source data/A549 cells/Raw data-Mito Tracker imaging in Figure 2A.tif]

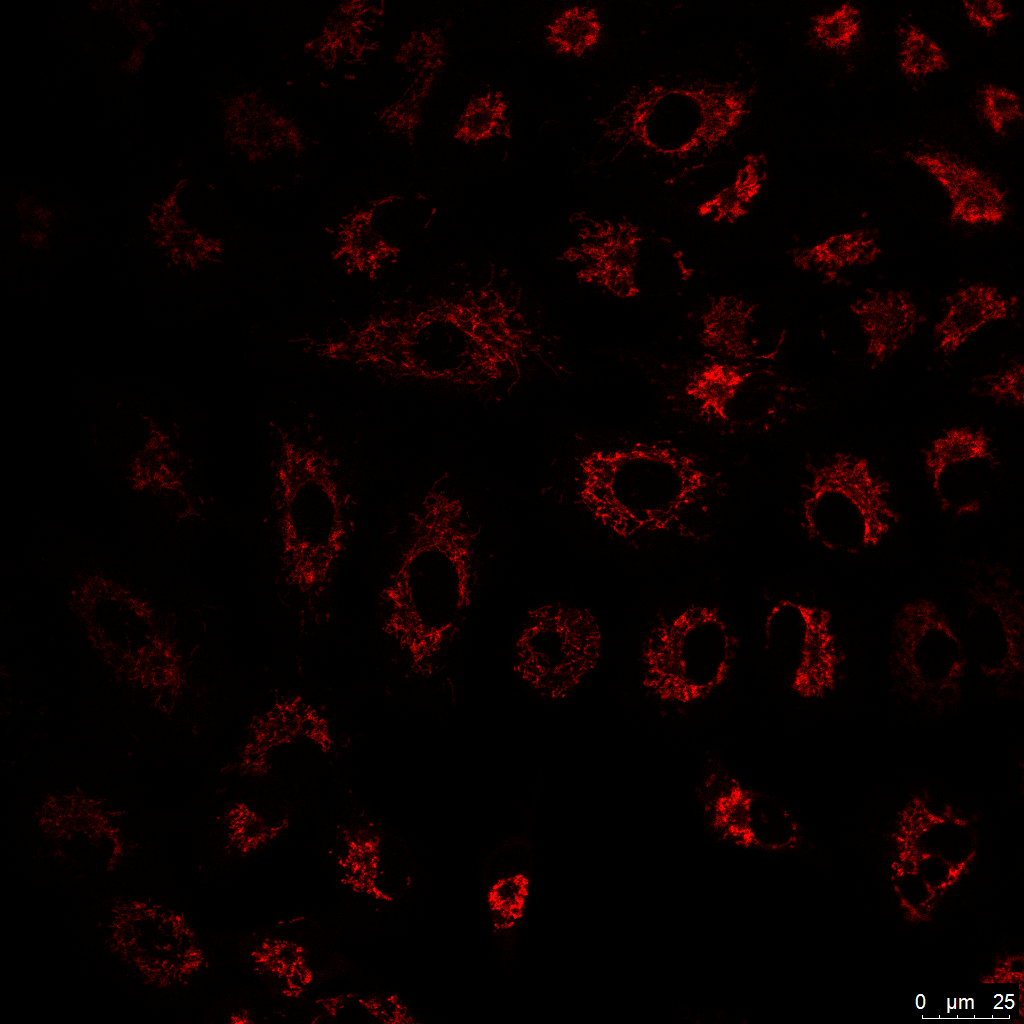

Supplement: Figure 2—source data 1. [file elife-70471-fig2-data1.zip › Figure 2-Source data/A549 cells/Raw data-IBS440 imaging in Figure 2A.tif]

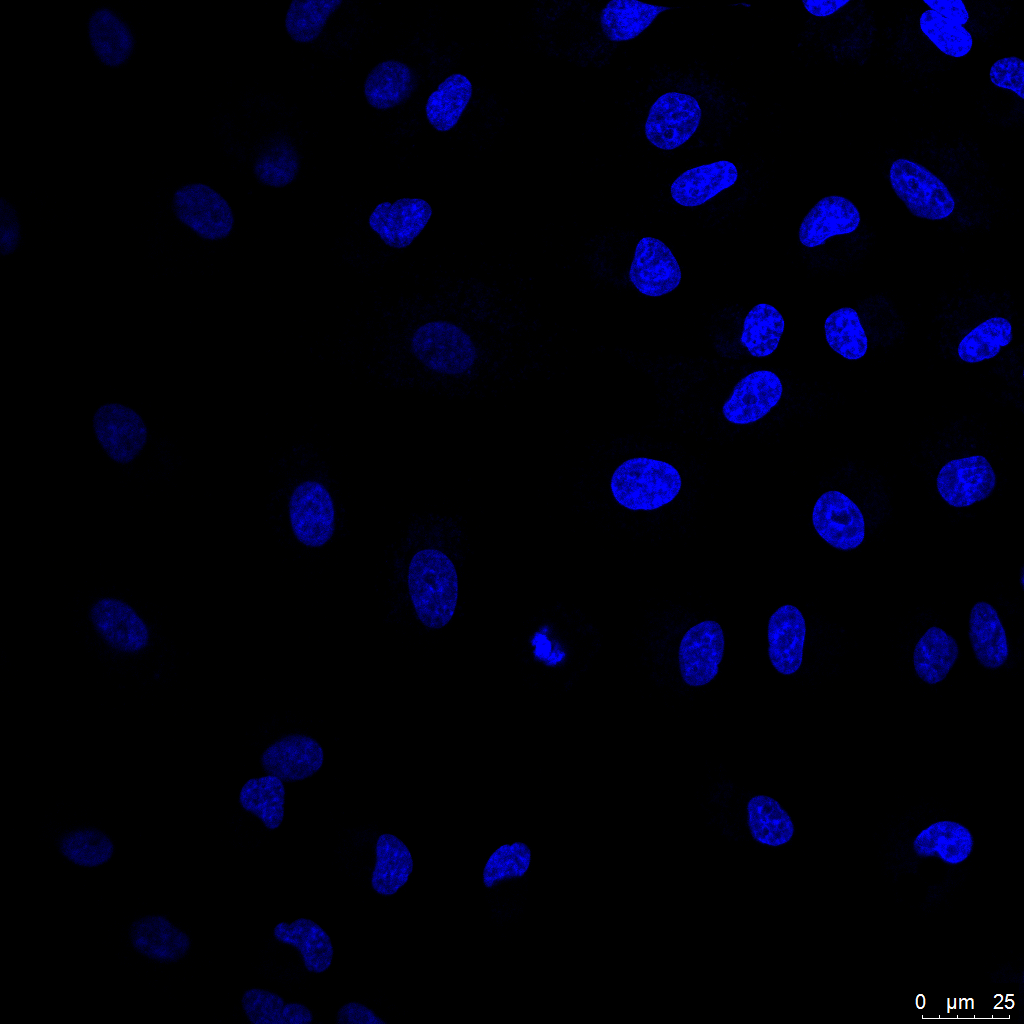

Supplement: Figure 2—source data 1. [file elife-70471-fig2-data1.zip › Figure 2-Source data/A549 cells/Raw data-Hoechst imaging in Figure 2A.tif]

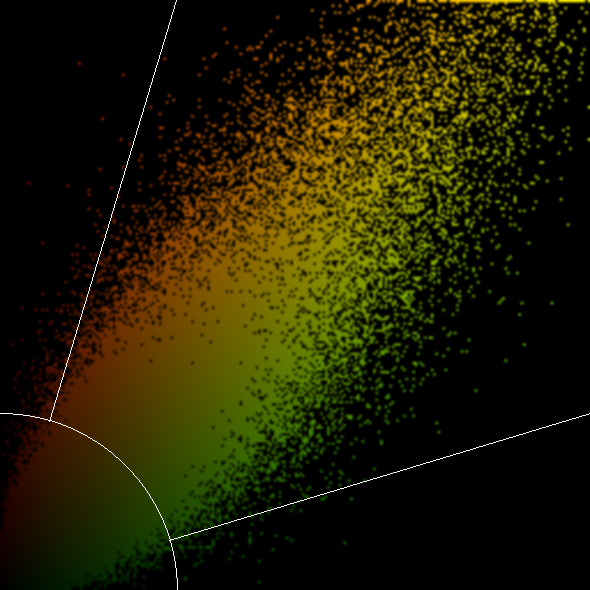

Supplement: Figure 2—source data 1. [file elife-70471-fig2-data1.zip › Figure 2-Source data/MHCC97H cells/Raw data-Colocalization in Figure 2A.tif]

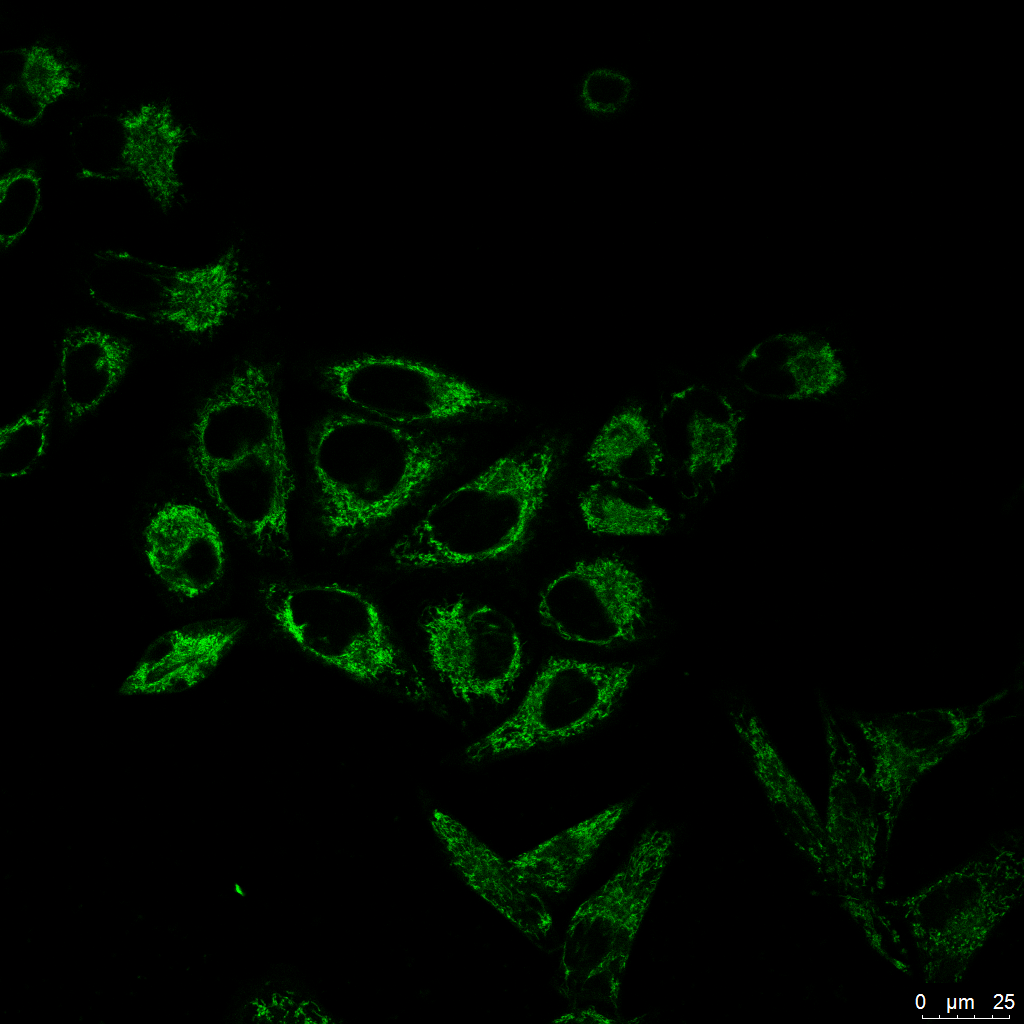

Supplement: Figure 2—source data 1. [file elife-70471-fig2-data1.zip › Figure 2-Source data/MHCC97H cells/Raw data-Mito Tracker imaging in Figure 2A.tif]

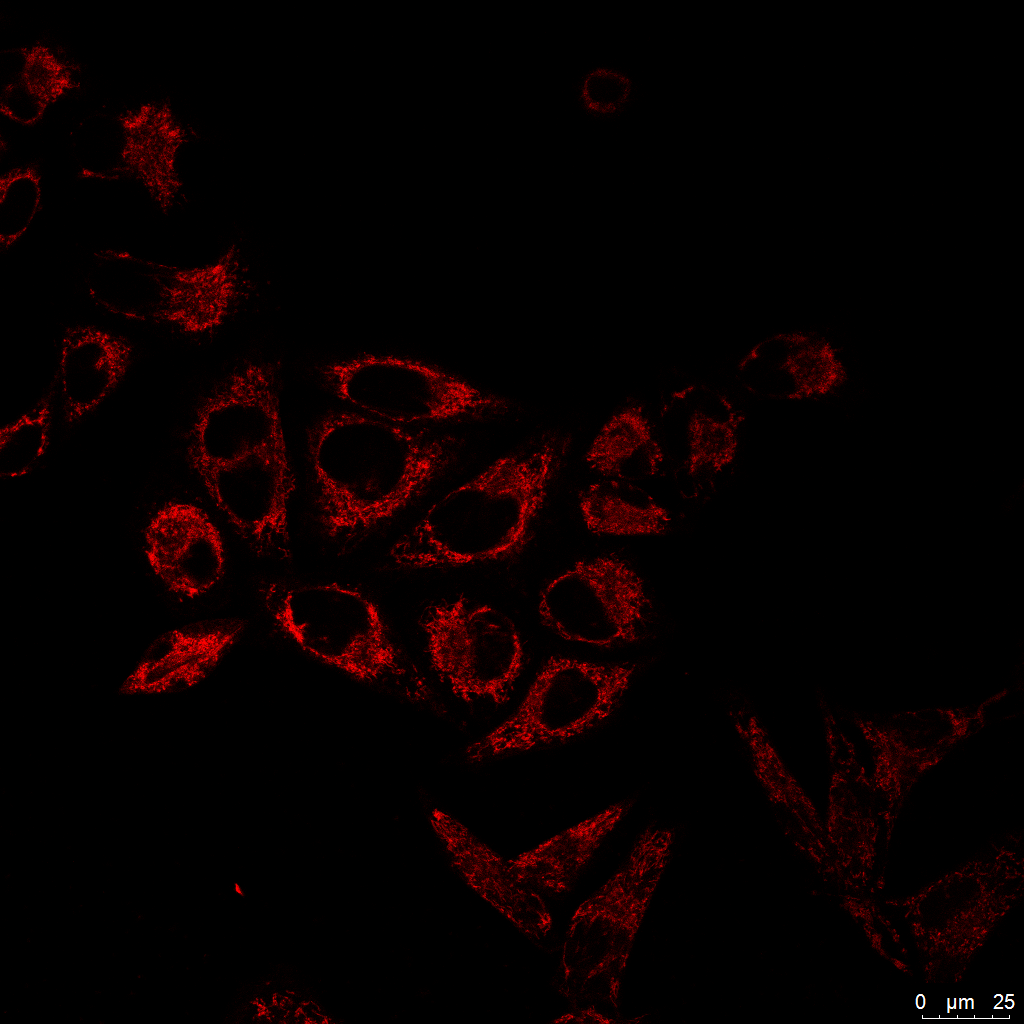

Supplement: Figure 2—source data 1. [file elife-70471-fig2-data1.zip › Figure 2-Source data/MHCC97H cells/Raw data-IBS440 imaging in Figure 2A.tif]

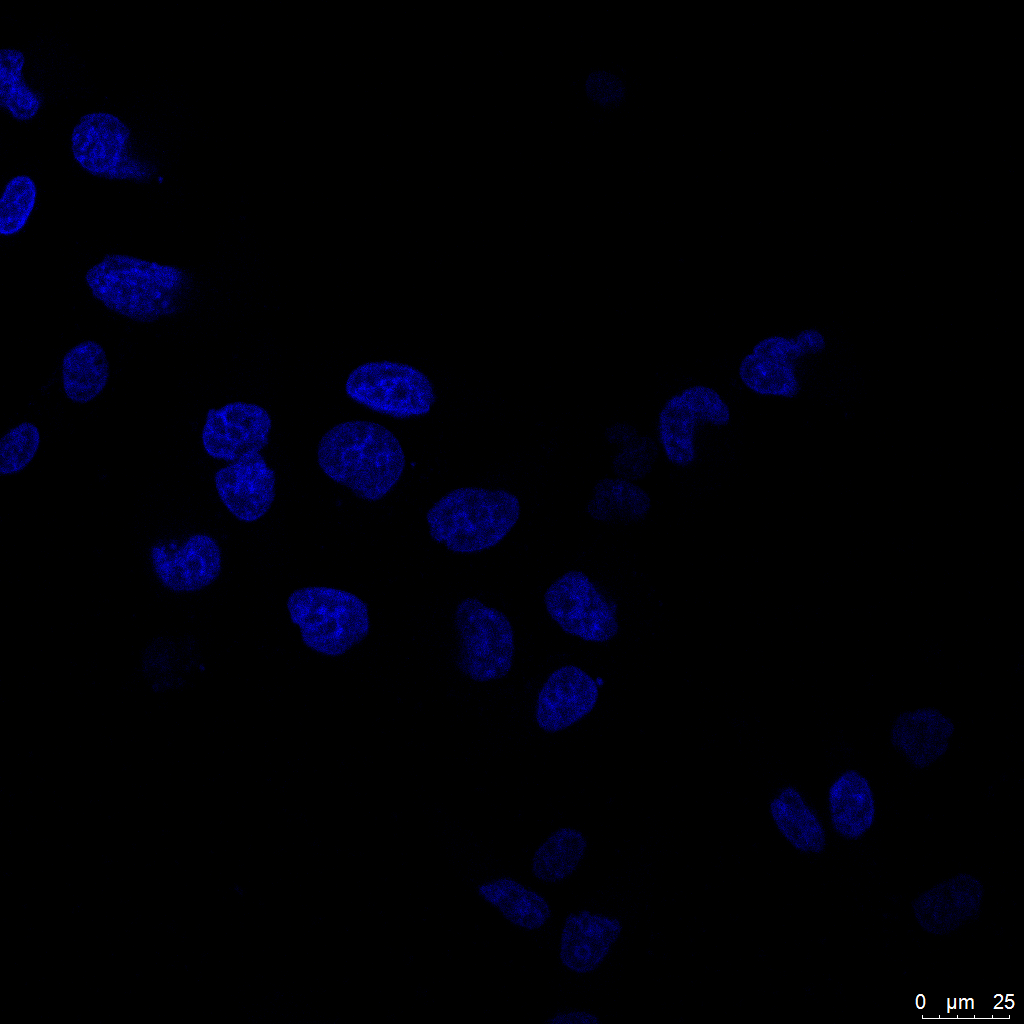

Supplement: Figure 2—source data 1. [file elife-70471-fig2-data1.zip › Figure 2-Source data/MHCC97H cells/Raw data-Hoechst imaging in Figure 2A.tif]

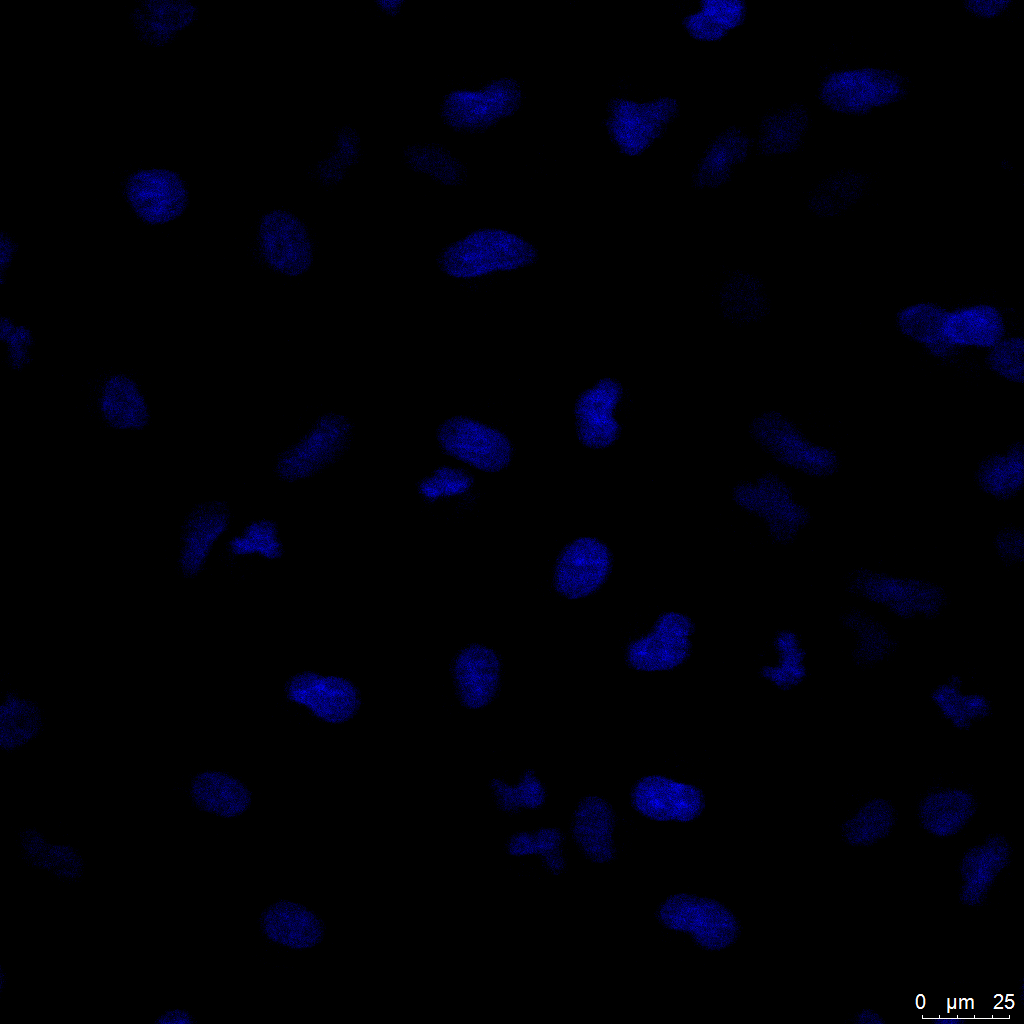

Supplement: Figure 2—figure supplement 2—source data 1. [file elife-70471-fig2-figsupp2-data1.zip › Figure 2-figure supplement 2-Source data 1/Mitotracker uptake in living cells/A549 mito_ch00.tif]

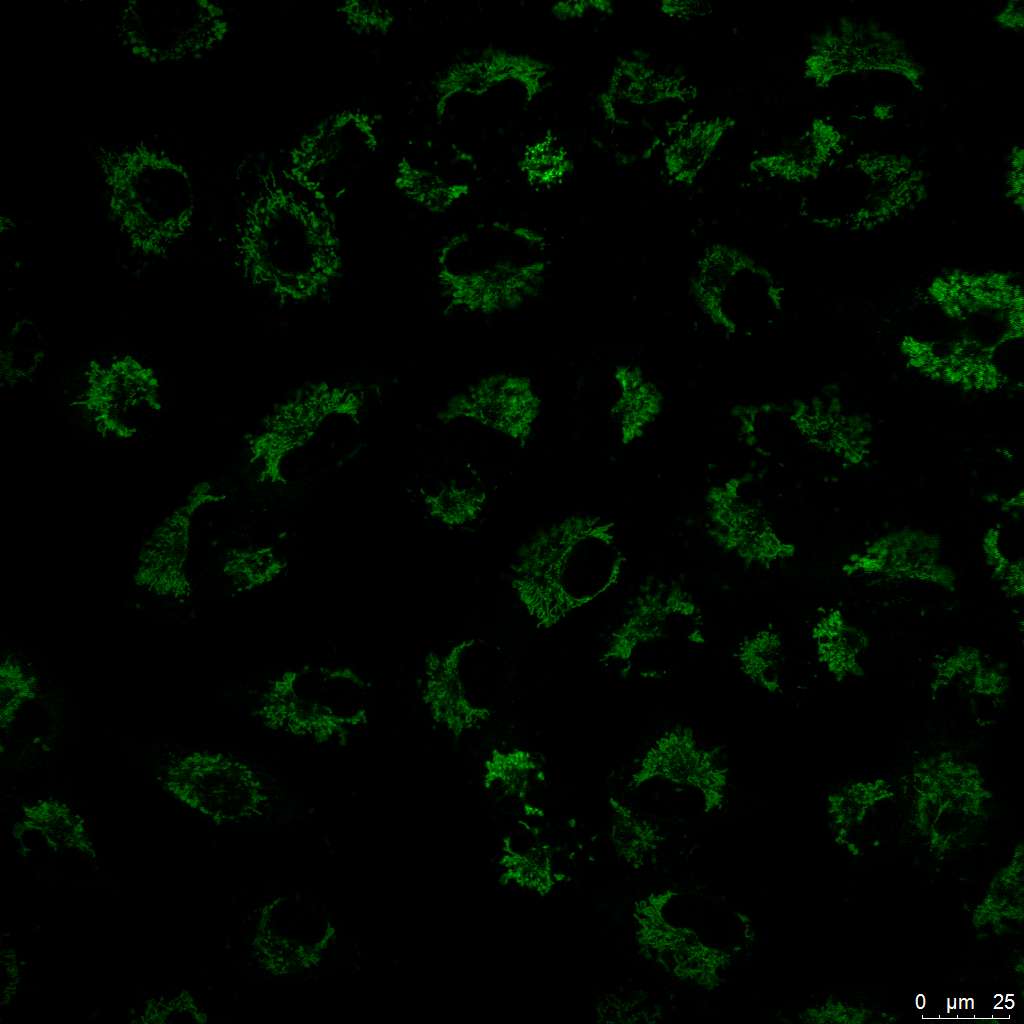

Supplement: Figure 2—figure supplement 2—source data 1. [file elife-70471-fig2-figsupp2-data1.zip › Figure 2-figure supplement 2-Source data 1/Mitotracker uptake in living cells/A549 mito_ch01.tif]

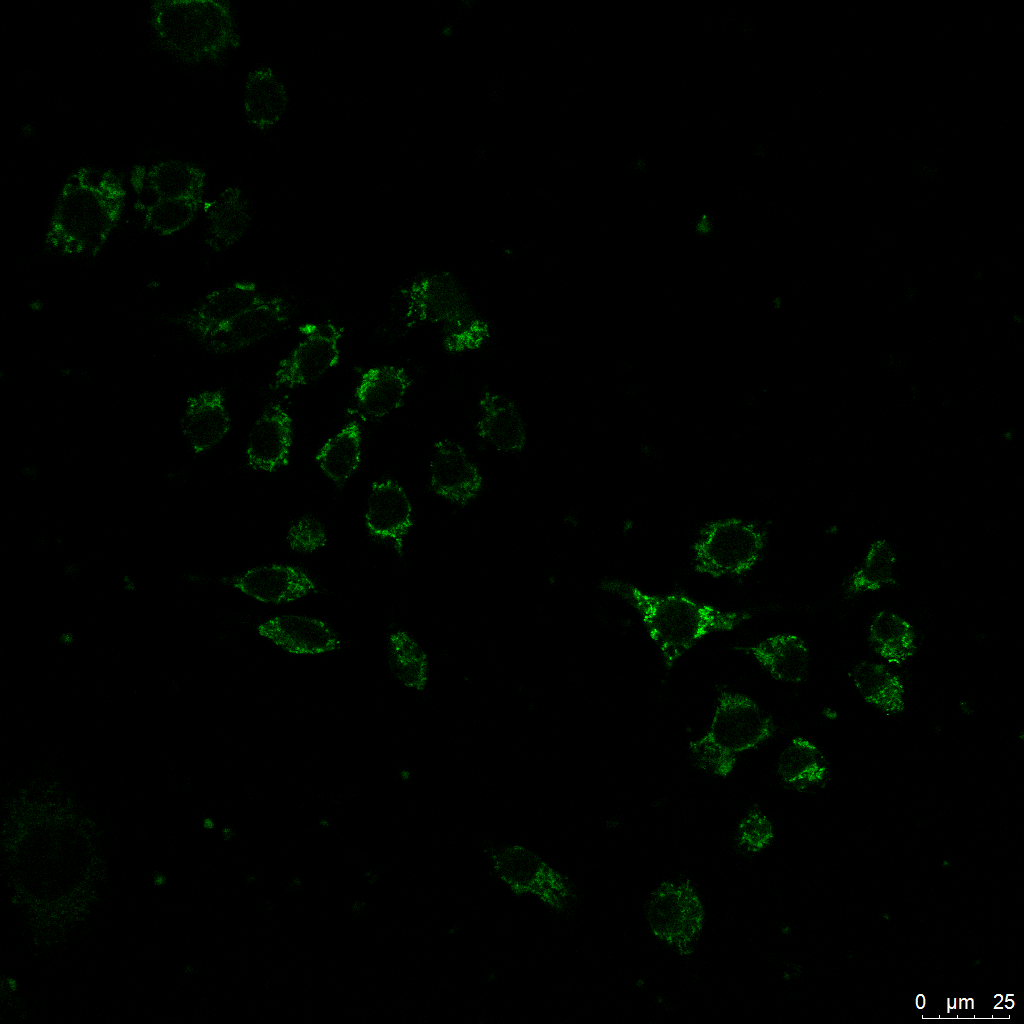

Supplement: Figure 2—figure supplement 2—source data 1. [file elife-70471-fig2-figsupp2-data1.zip › Figure 2-figure supplement 2-Source data 1/Mitotracker uptake in living cells/RAW264.7 mito_ch01.tif]

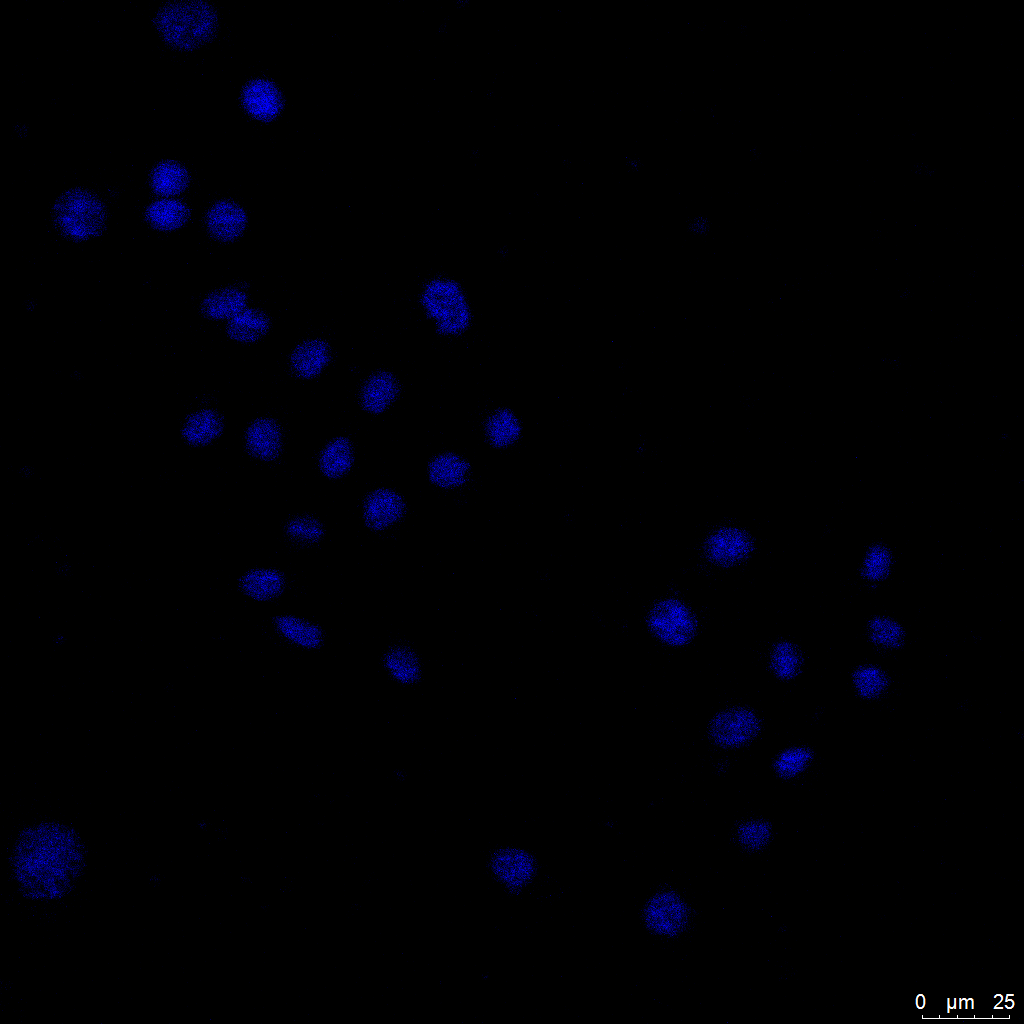

Supplement: Figure 2—figure supplement 2—source data 1. [file elife-70471-fig2-figsupp2-data1.zip › Figure 2-figure supplement 2-Source data 1/Mitotracker uptake in living cells/RAW264.7 mito_ch00.tif]

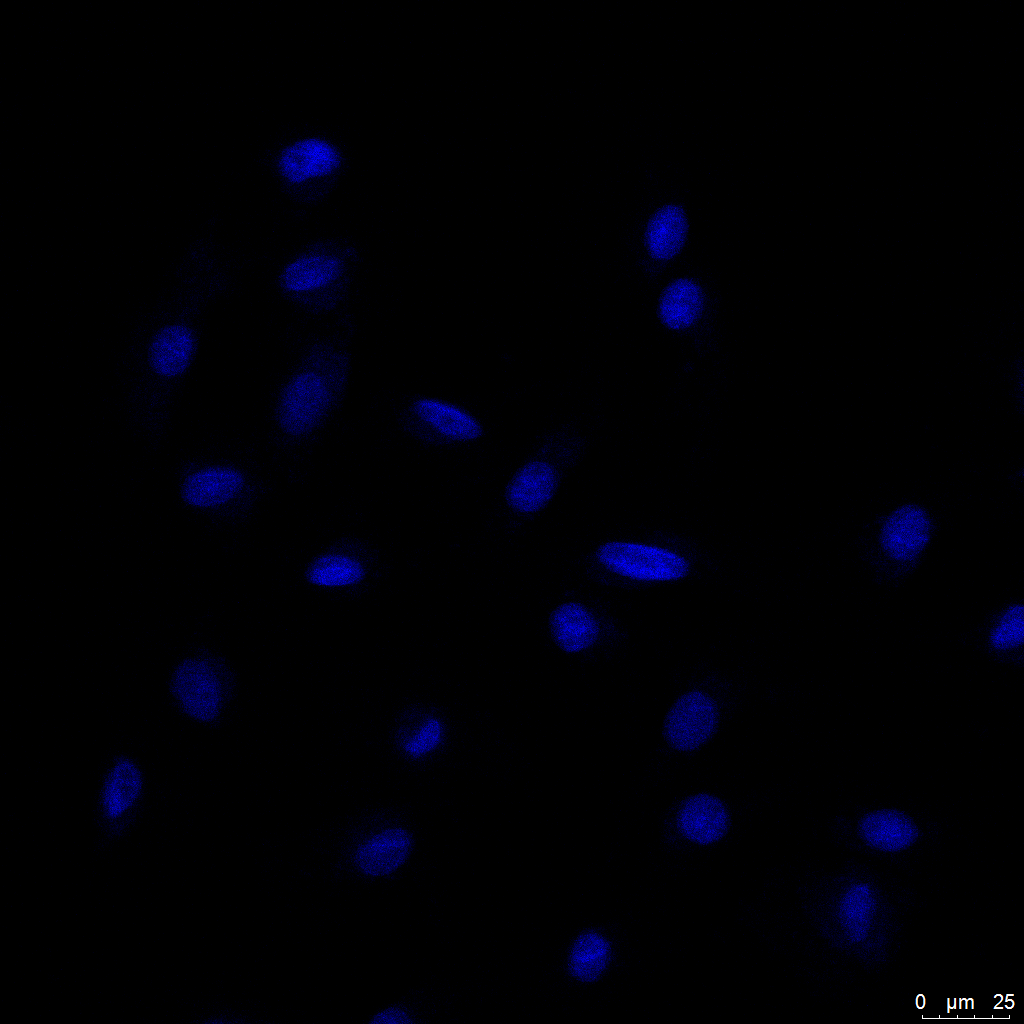

Supplement: Figure 2—figure supplement 2—source data 1. [file elife-70471-fig2-figsupp2-data1.zip › Figure 2-figure supplement 2-Source data 1/Mitotracker uptake in living cells/A7r5 mito_ch00.tif]

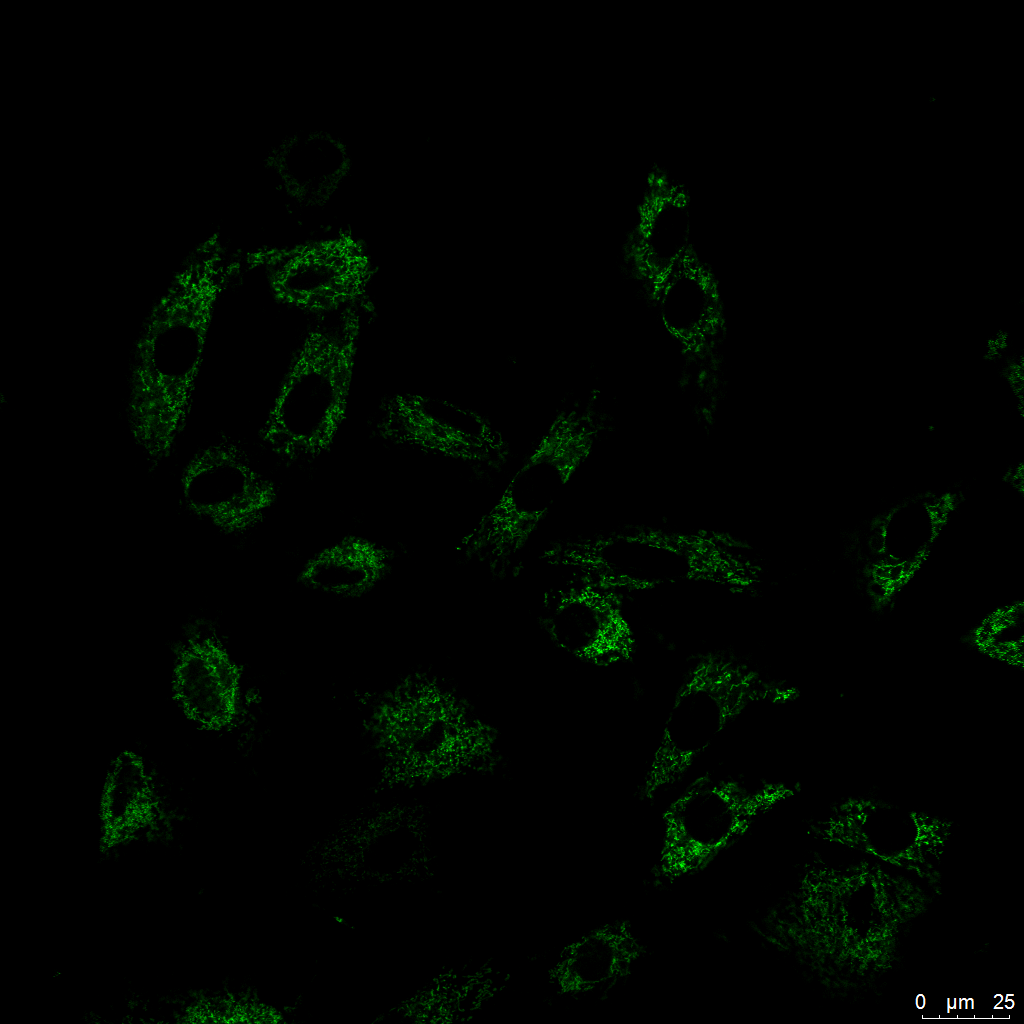

Supplement: Figure 2—figure supplement 2—source data 1. [file elife-70471-fig2-figsupp2-data1.zip › Figure 2-figure supplement 2-Source data 1/Mitotracker uptake in living cells/A7r5 mito_ch01.tif]

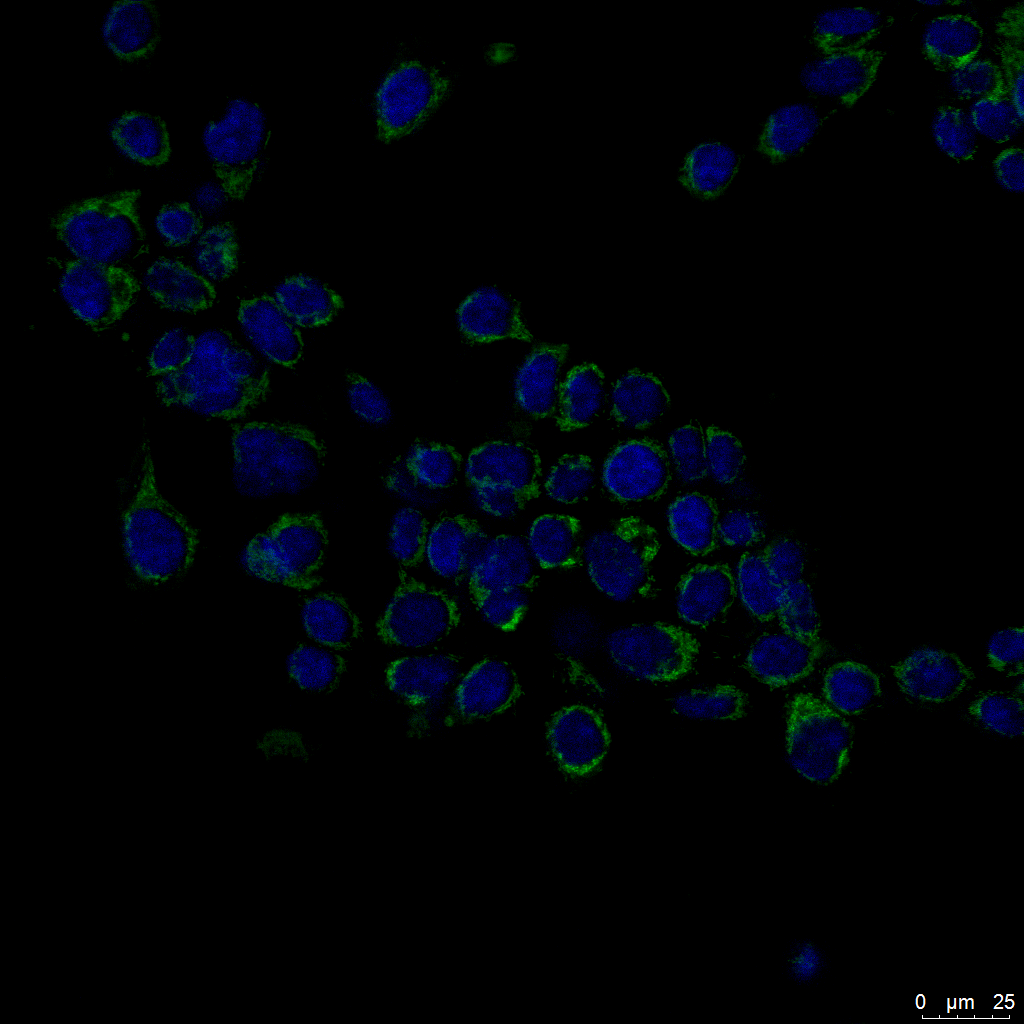

Supplement: Figure 2—figure supplement 2—source data 1. [file elife-70471-fig2-figsupp2-data1.zip › Figure 2-figure supplement 2-Source data 1/Mitotracker uptake in living cells/FaDu mito.tif]

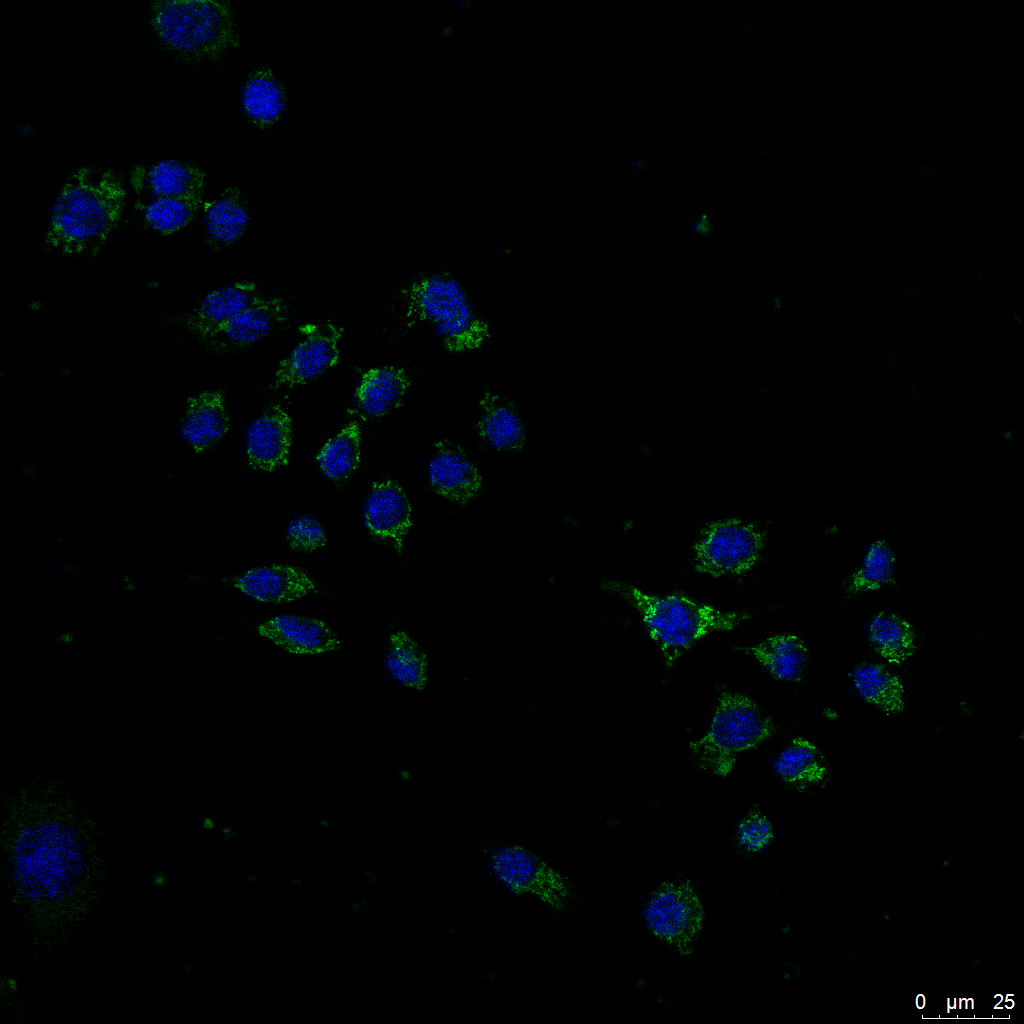

Supplement: Figure 2—figure supplement 2—source data 1. [file elife-70471-fig2-figsupp2-data1.zip › Figure 2-figure supplement 2-Source data 1/Mitotracker uptake in living cells/RAW264.7 mito.tif]

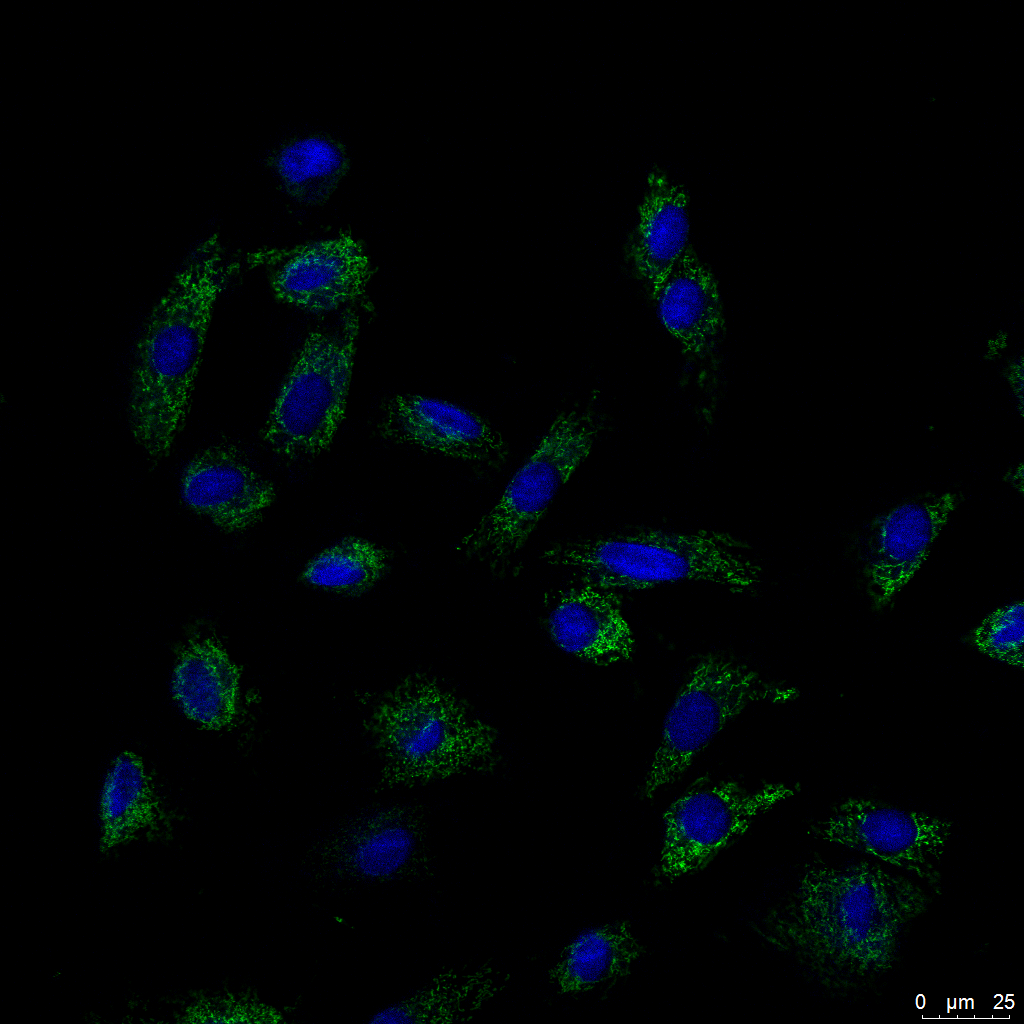

Supplement: Figure 2—figure supplement 2—source data 1. [file elife-70471-fig2-figsupp2-data1.zip › Figure 2-figure supplement 2-Source data 1/Mitotracker uptake in living cells/A7r5 mito.tif]

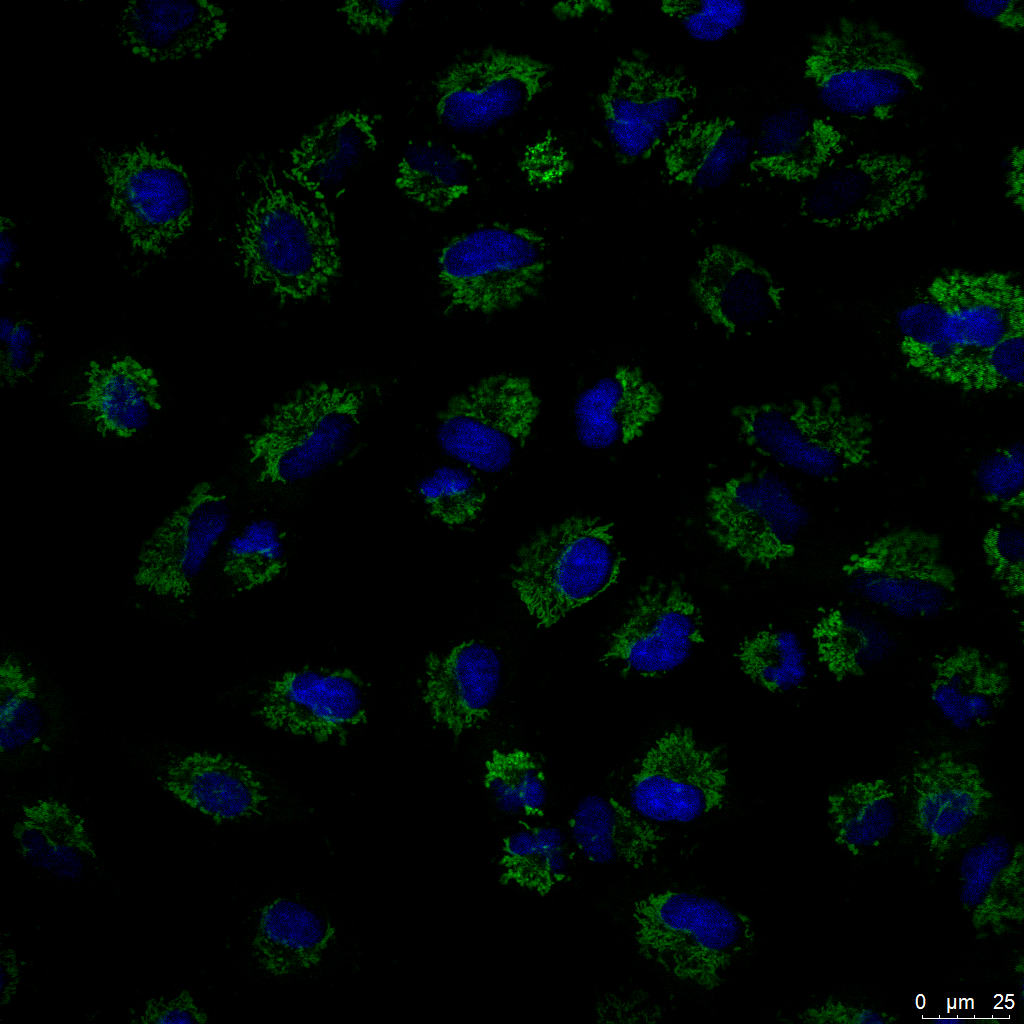

Supplement: Figure 2—figure supplement 2—source data 1. [file elife-70471-fig2-figsupp2-data1.zip › Figure 2-figure supplement 2-Source data 1/Mitotracker uptake in living cells/A549 mito.tif]

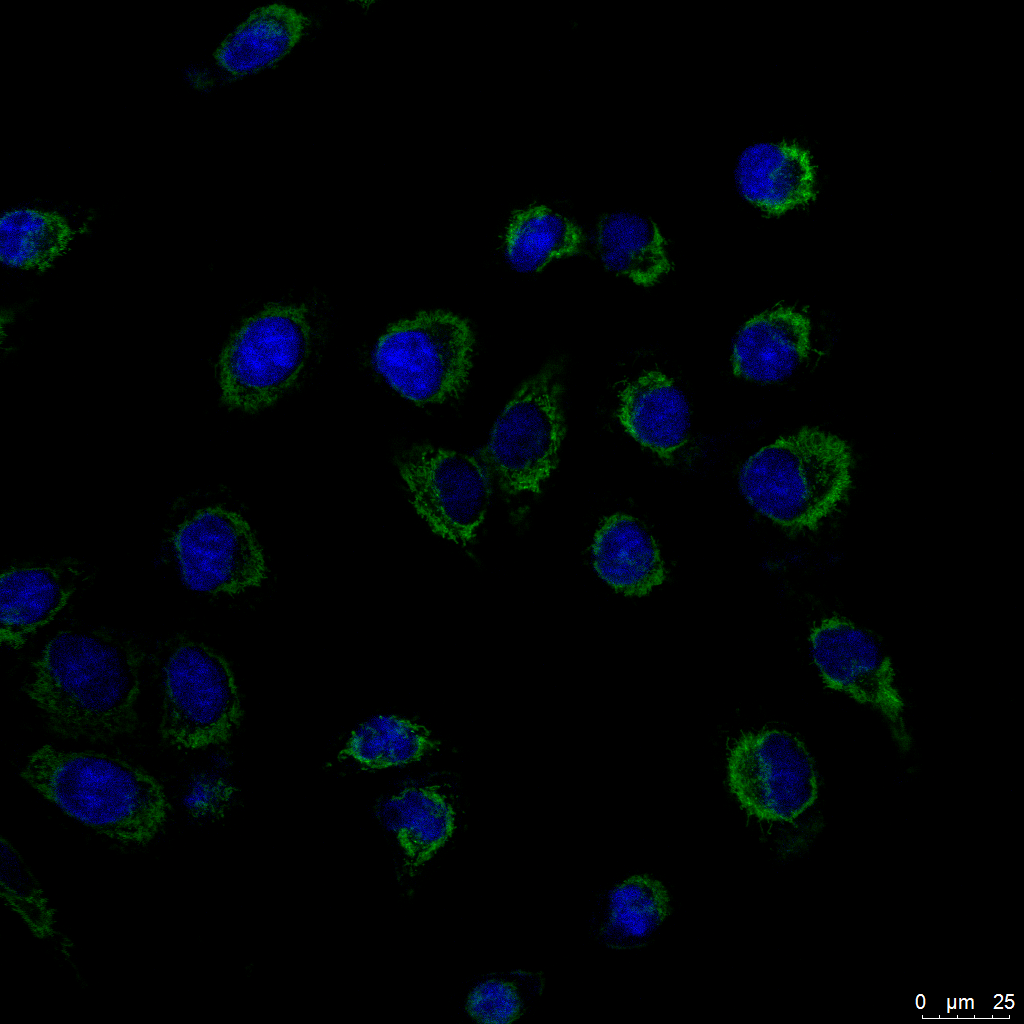

Supplement: Figure 2—figure supplement 2—source data 1. [file elife-70471-fig2-figsupp2-data1.zip › Figure 2-figure supplement 2-Source data 1/Mitotracker uptake in living cells/97H mito.tif]

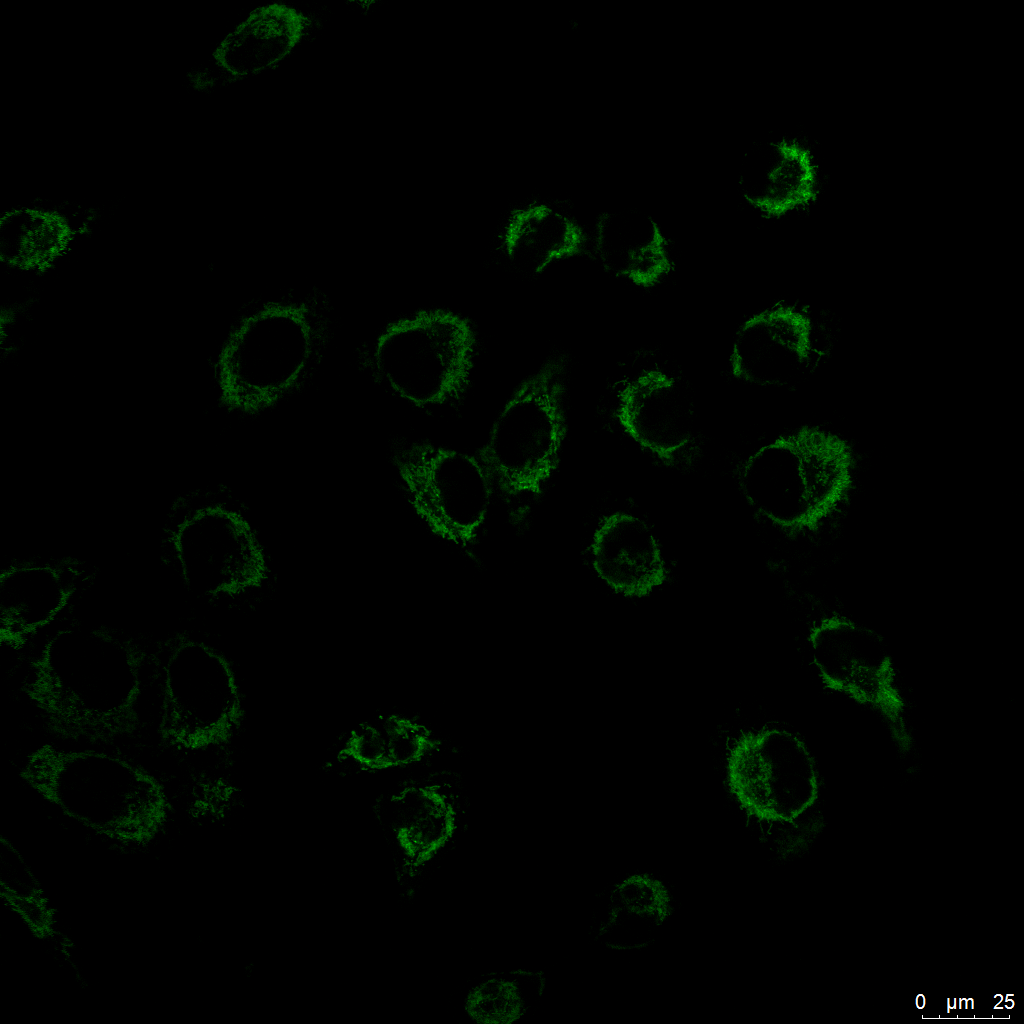

Supplement: Figure 2—figure supplement 2—source data 1. [file elife-70471-fig2-figsupp2-data1.zip › Figure 2-figure supplement 2-Source data 1/Mitotracker uptake in living cells/97H mito_ch01.tif]

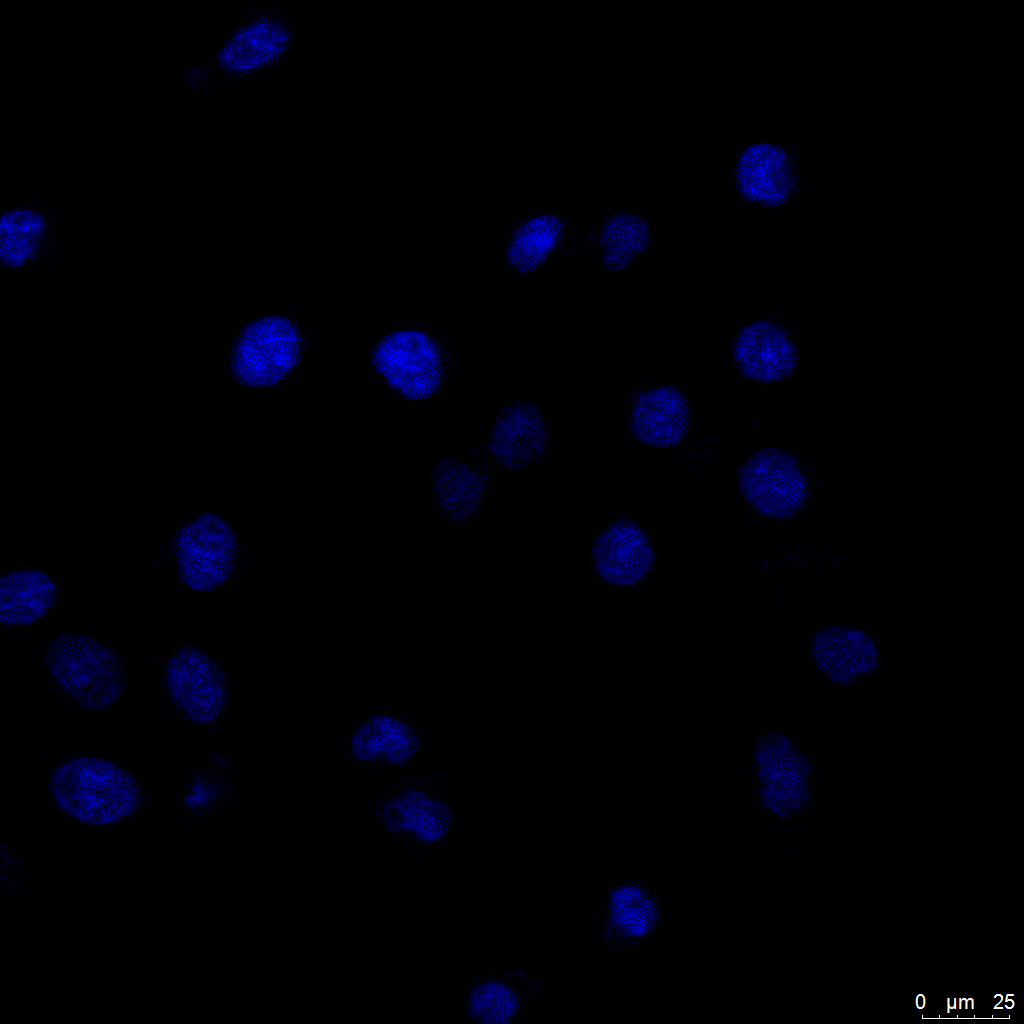

Supplement: Figure 2—figure supplement 2—source data 1. [file elife-70471-fig2-figsupp2-data1.zip › Figure 2-figure supplement 2-Source data 1/Mitotracker uptake in living cells/97H mito_ch00.tif]

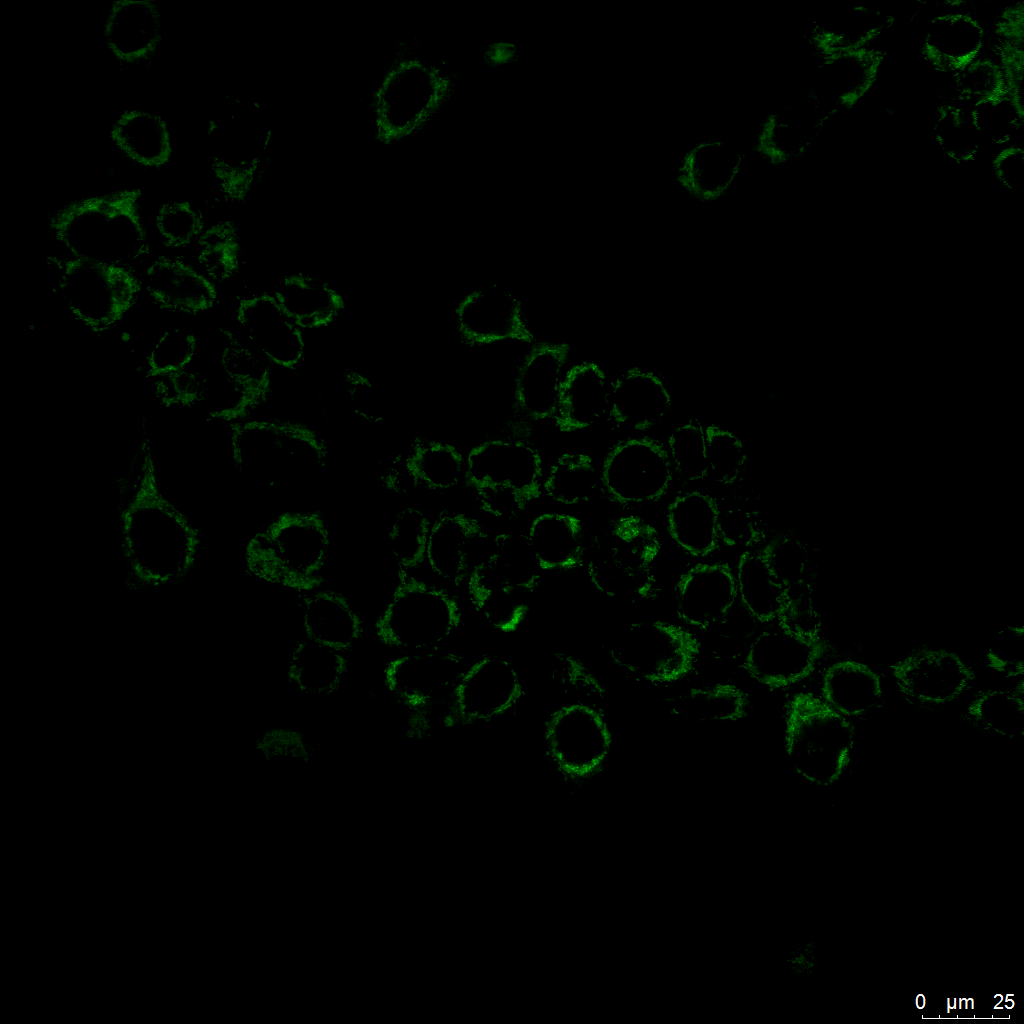

Supplement: Figure 2—figure supplement 2—source data 1. [file elife-70471-fig2-figsupp2-data1.zip › Figure 2-figure supplement 2-Source data 1/Mitotracker uptake in living cells/FaDu mito_ch01.tif]

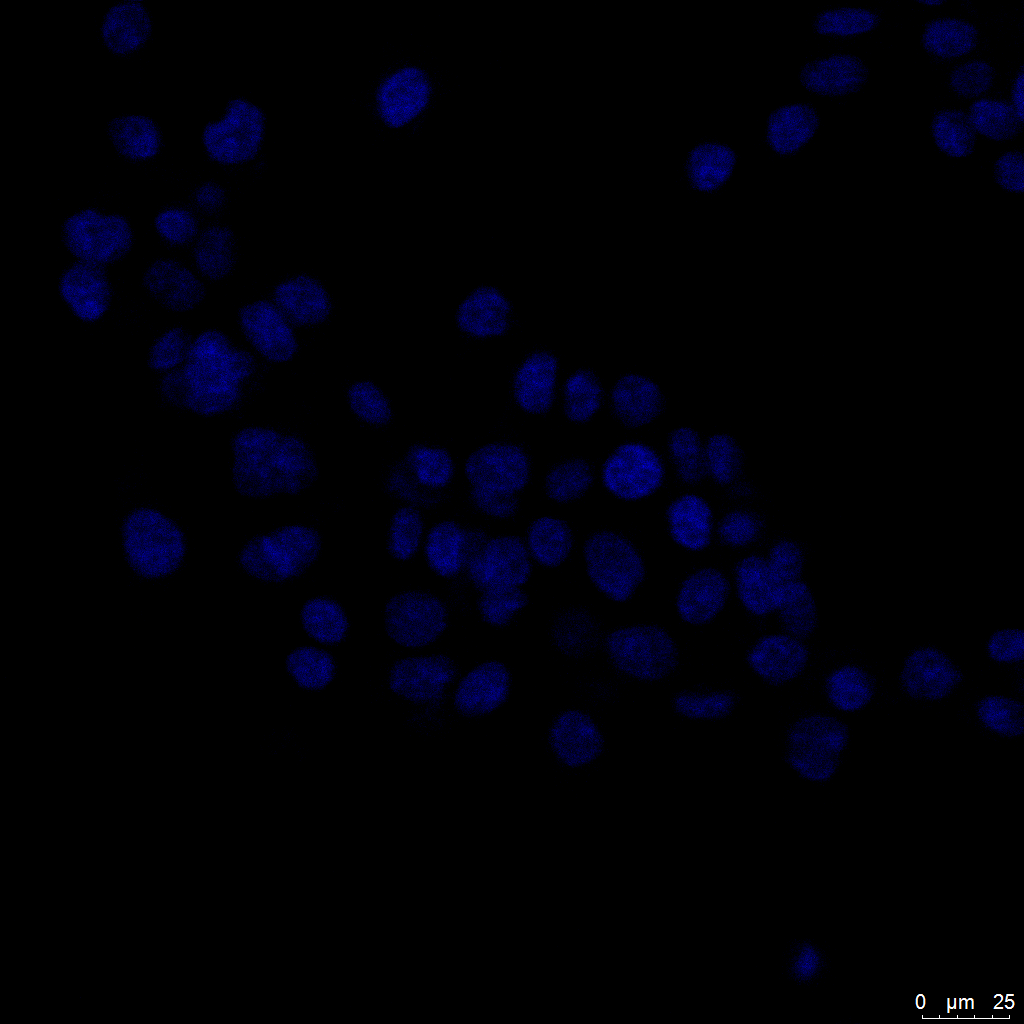

Supplement: Figure 2—figure supplement 2—source data 1. [file elife-70471-fig2-figsupp2-data1.zip › Figure 2-figure supplement 2-Source data 1/Mitotracker uptake in living cells/FaDu mito_ch00.tif]

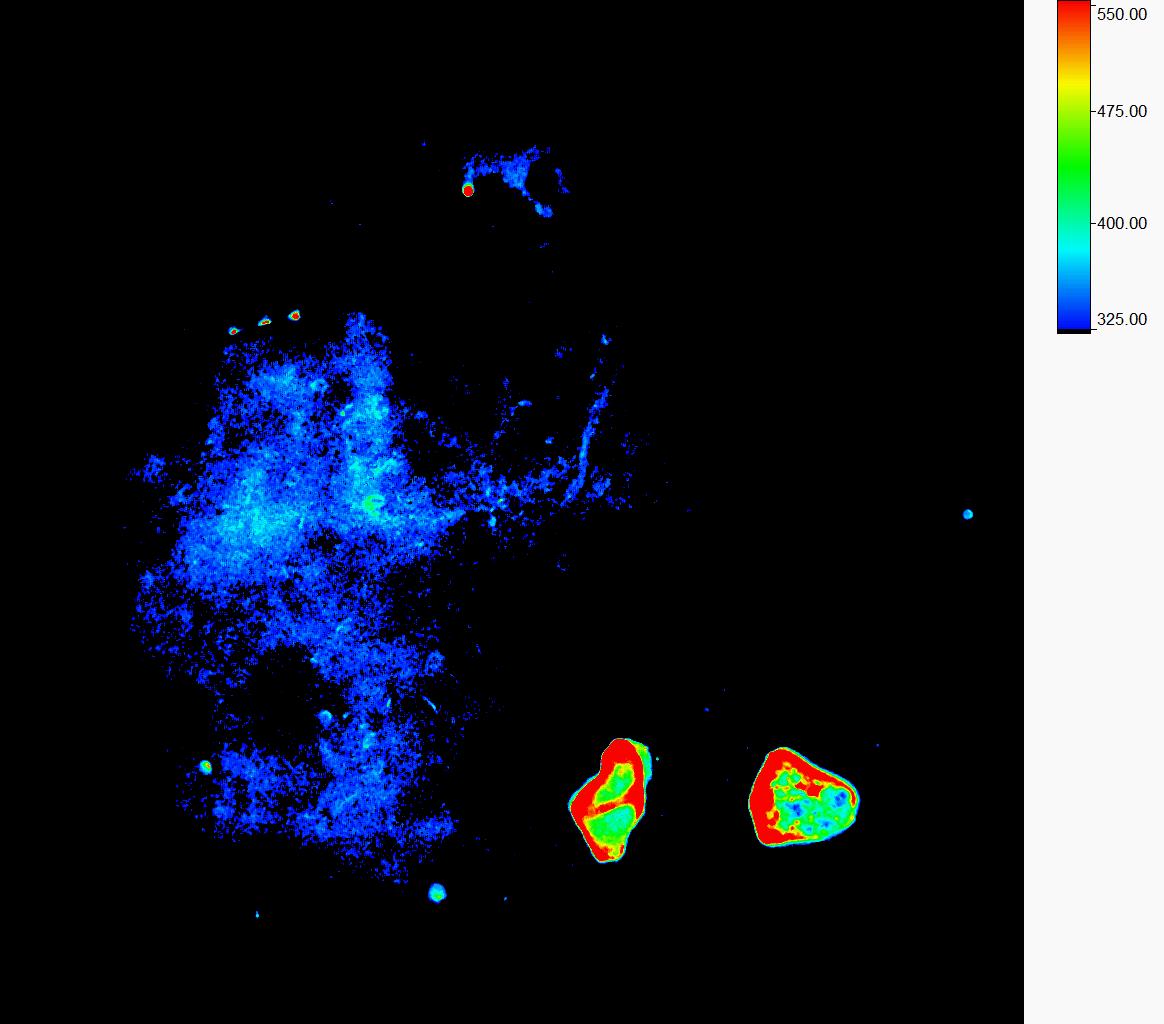

Supplement: Figure 2—figure supplement 2—source data 1. [file elife-70471-fig2-figsupp2-data1.zip › Figure 2-figure supplement 2-Source data 1/Mitotracker uptake in tissues/fluorescence detection.jpg]

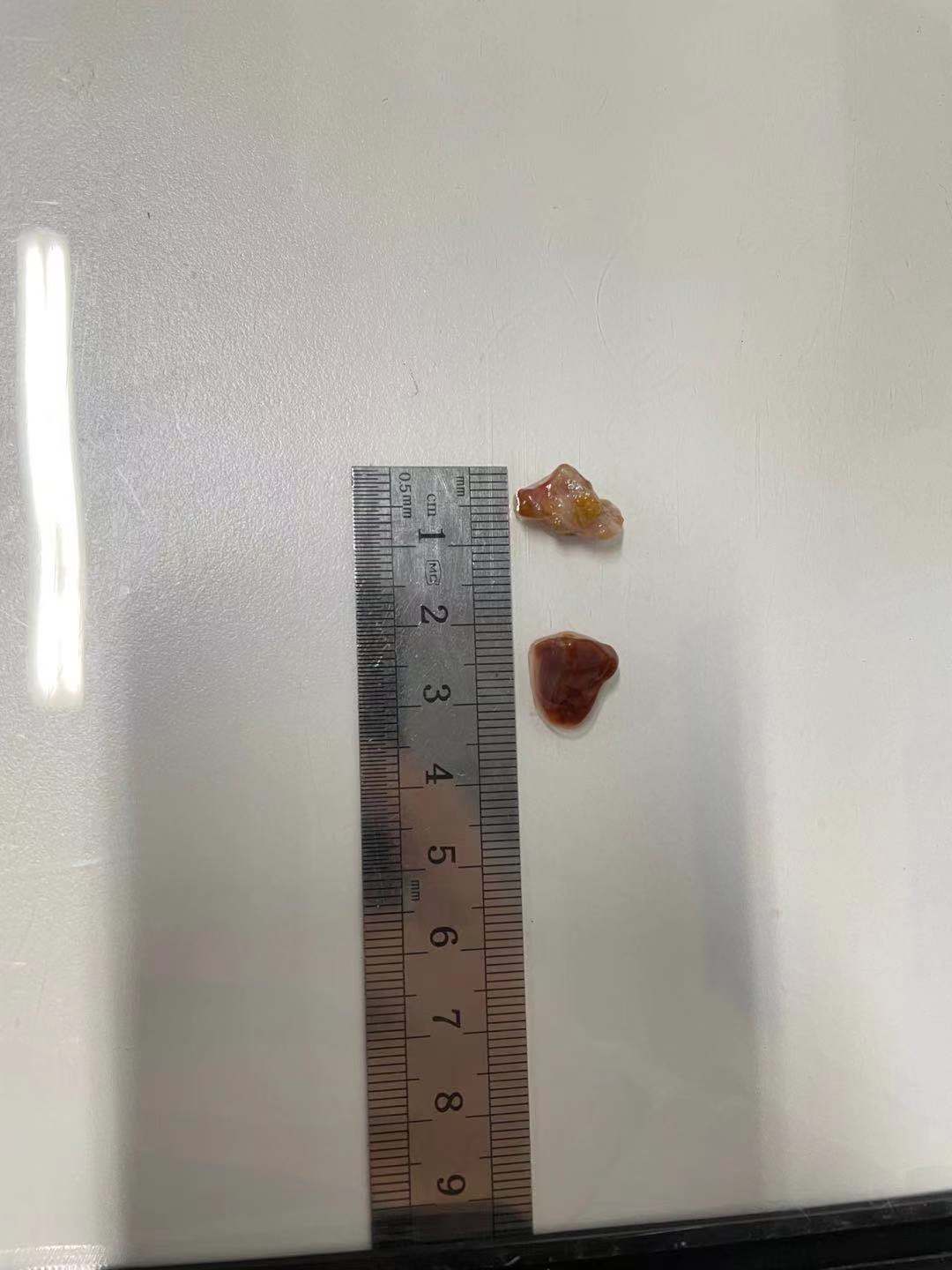

Supplement: Figure 2—figure supplement 2—source data 1. [file elife-70471-fig2-figsupp2-data1.zip › Figure 2-figure supplement 2-Source data 1/Mitotracker uptake in tissues/photograph.jpeg]

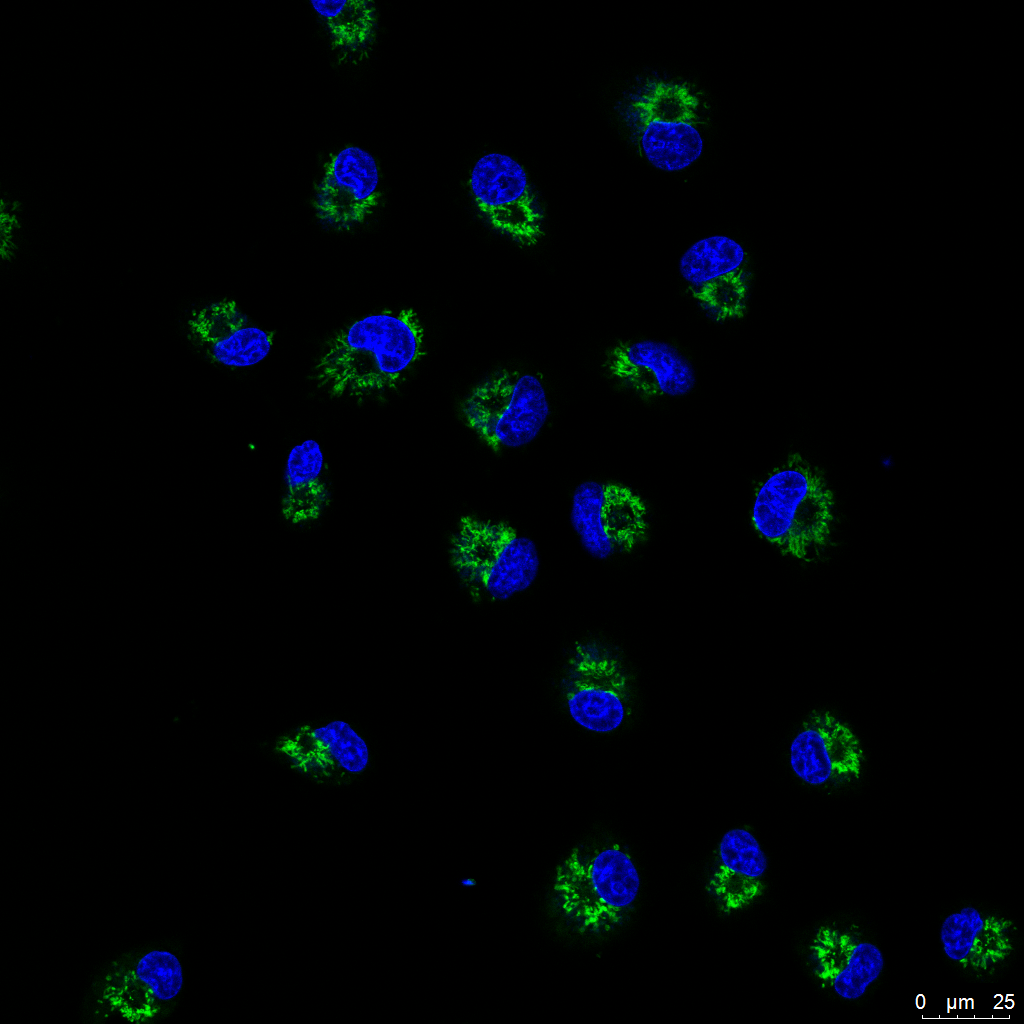

Supplement: Figure 2—figure supplement 5—source data 1. [file elife-70471-fig2-figsupp5-data1.zip › Figure 2-figure supplement 5-Source data 1/A549-hypoxia/201026_Series060 hypoxia.tif]

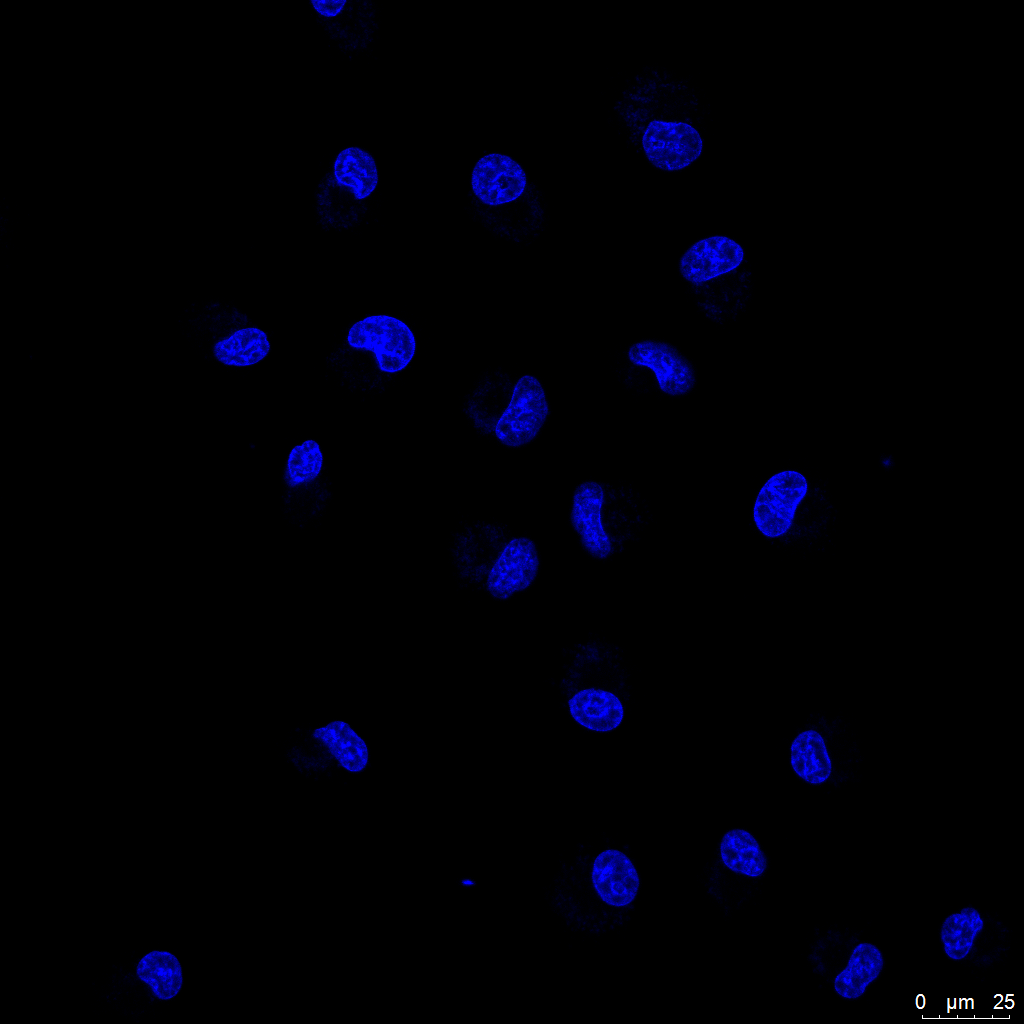

Supplement: Figure 2—figure supplement 5—source data 1. [file elife-70471-fig2-figsupp5-data1.zip › Figure 2-figure supplement 5-Source data 1/A549-hypoxia/201026_Series060 hypoxia_ch00.tif]

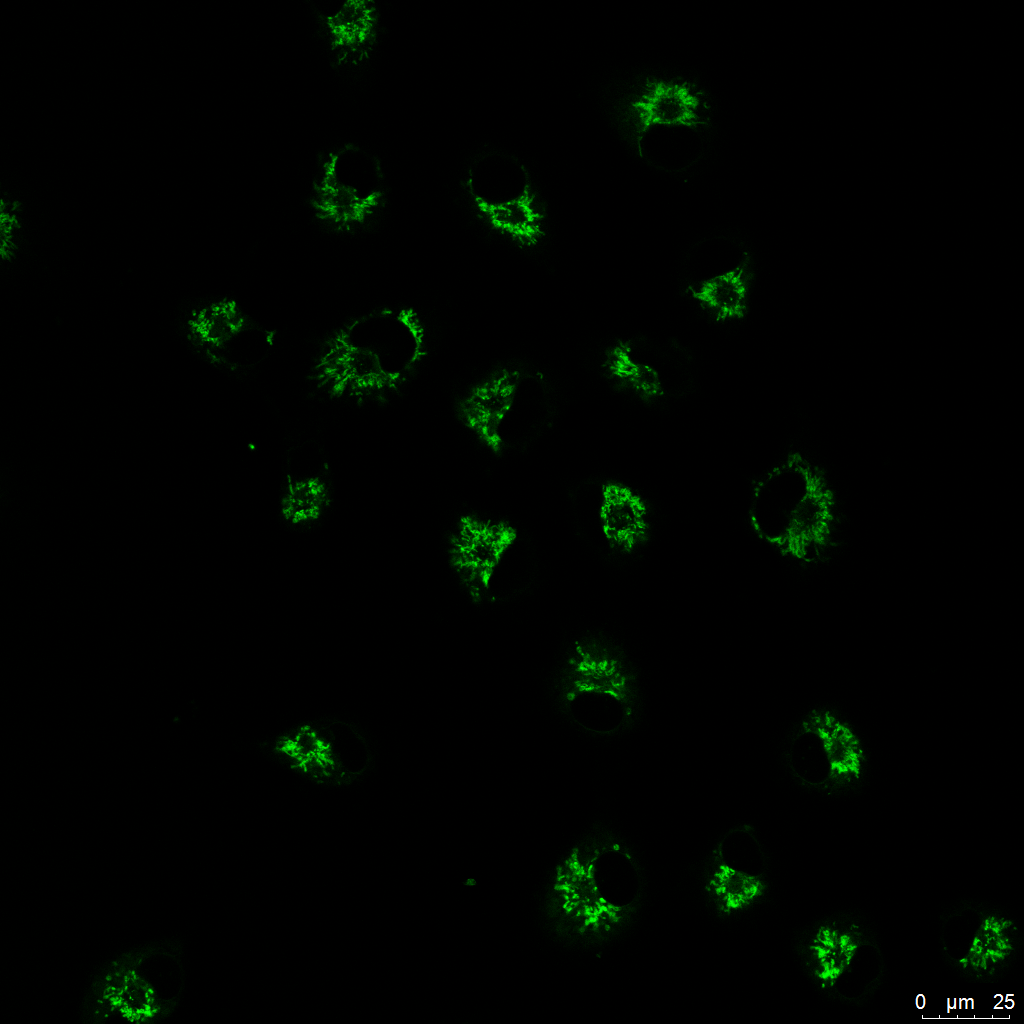

Supplement: Figure 2—figure supplement 5—source data 1. [file elife-70471-fig2-figsupp5-data1.zip › Figure 2-figure supplement 5-Source data 1/A549-hypoxia/201026_Series060 hypoxia_ch01.tif]

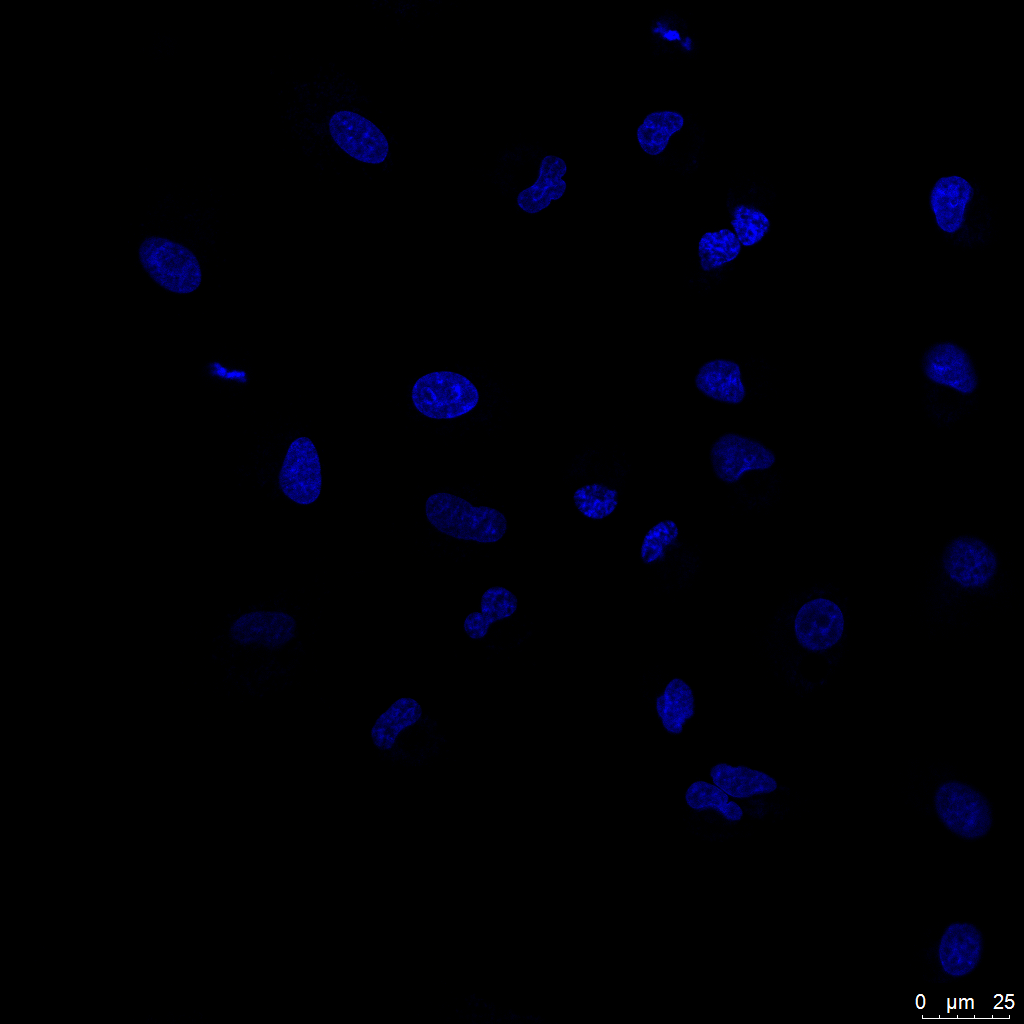

Supplement: Figure 2—figure supplement 5—source data 1. [file elife-70471-fig2-figsupp5-data1.zip › Figure 2-figure supplement 5-Source data 1/A549-normoxia/201026_Series040 normoxia_ch00.tif]

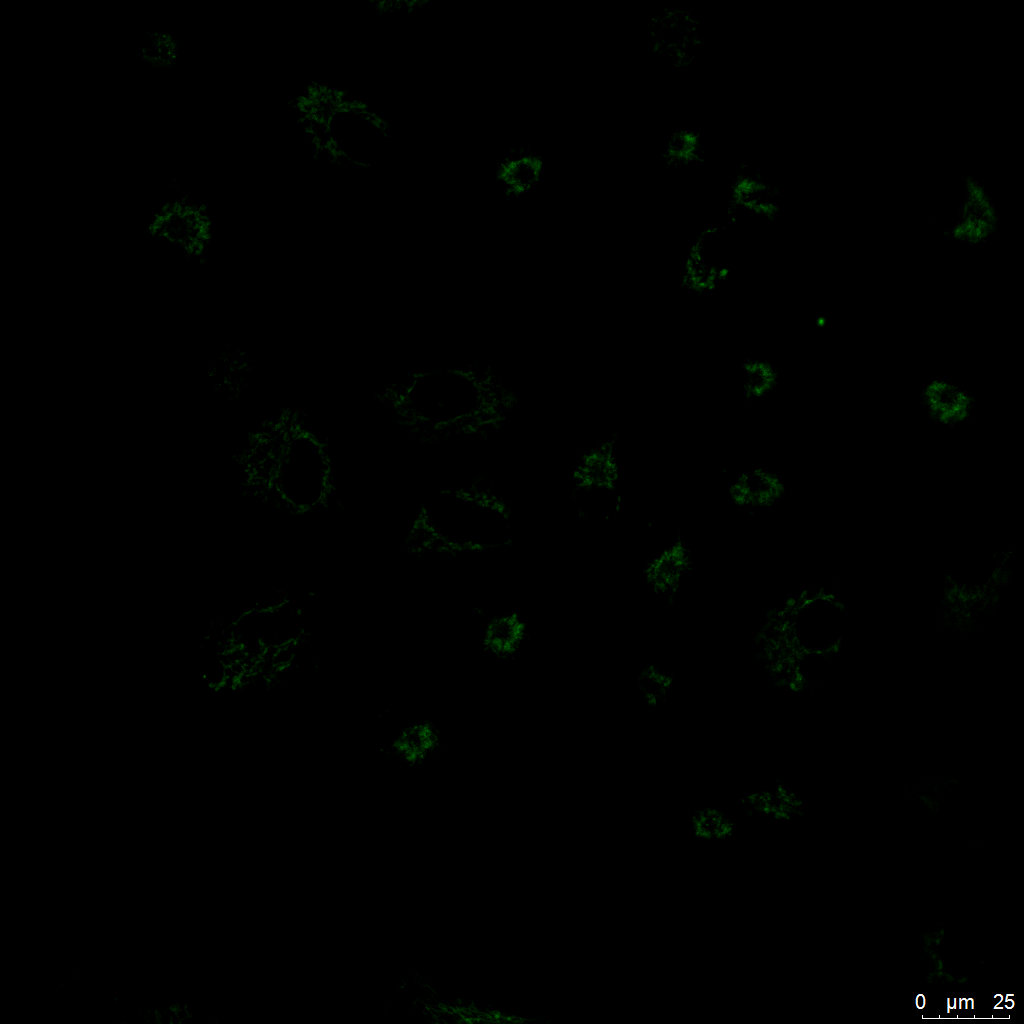

Supplement: Figure 2—figure supplement 5—source data 1. [file elife-70471-fig2-figsupp5-data1.zip › Figure 2-figure supplement 5-Source data 1/A549-normoxia/201026_Series040 normoxia_ch01.tif]

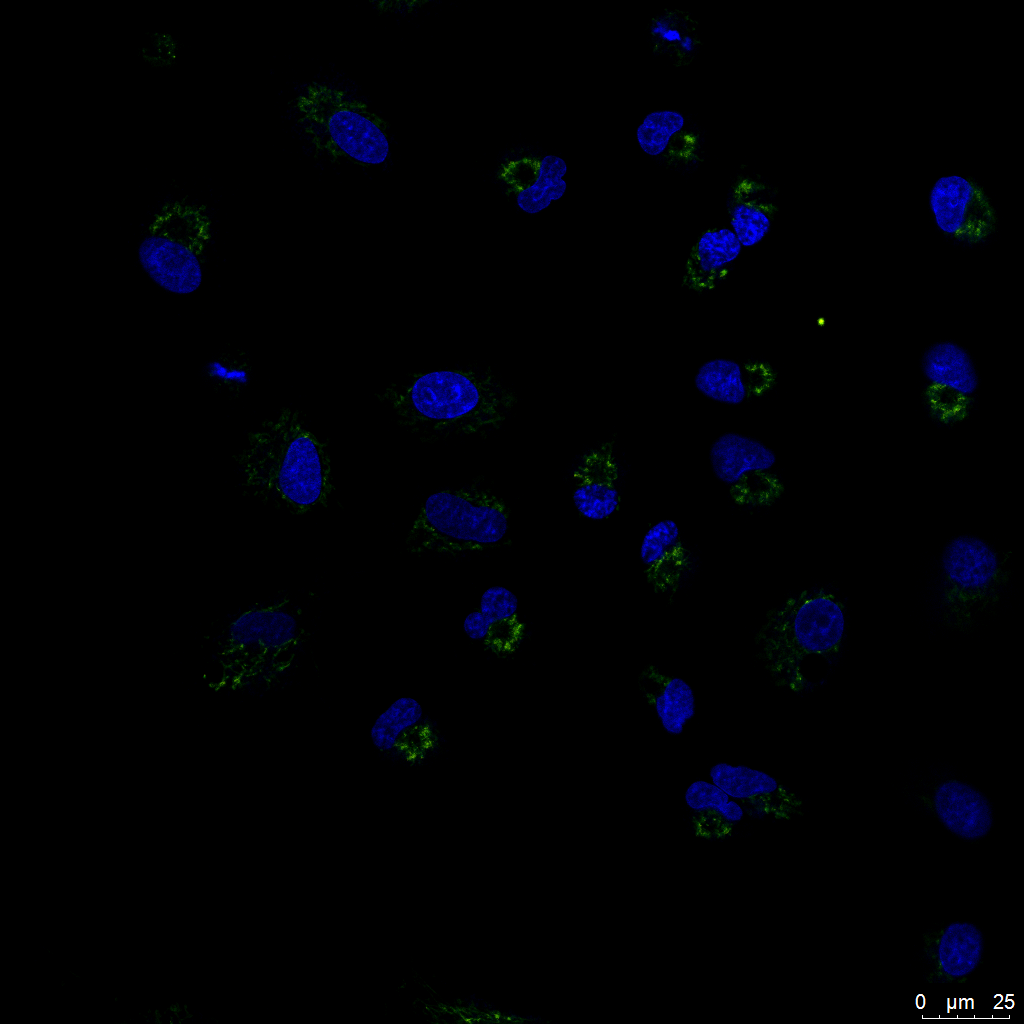

Supplement: Figure 2—figure supplement 5—source data 1. [file elife-70471-fig2-figsupp5-data1.zip › Figure 2-figure supplement 5-Source data 1/A549-normoxia/201026_Series040 normoxia.tif]

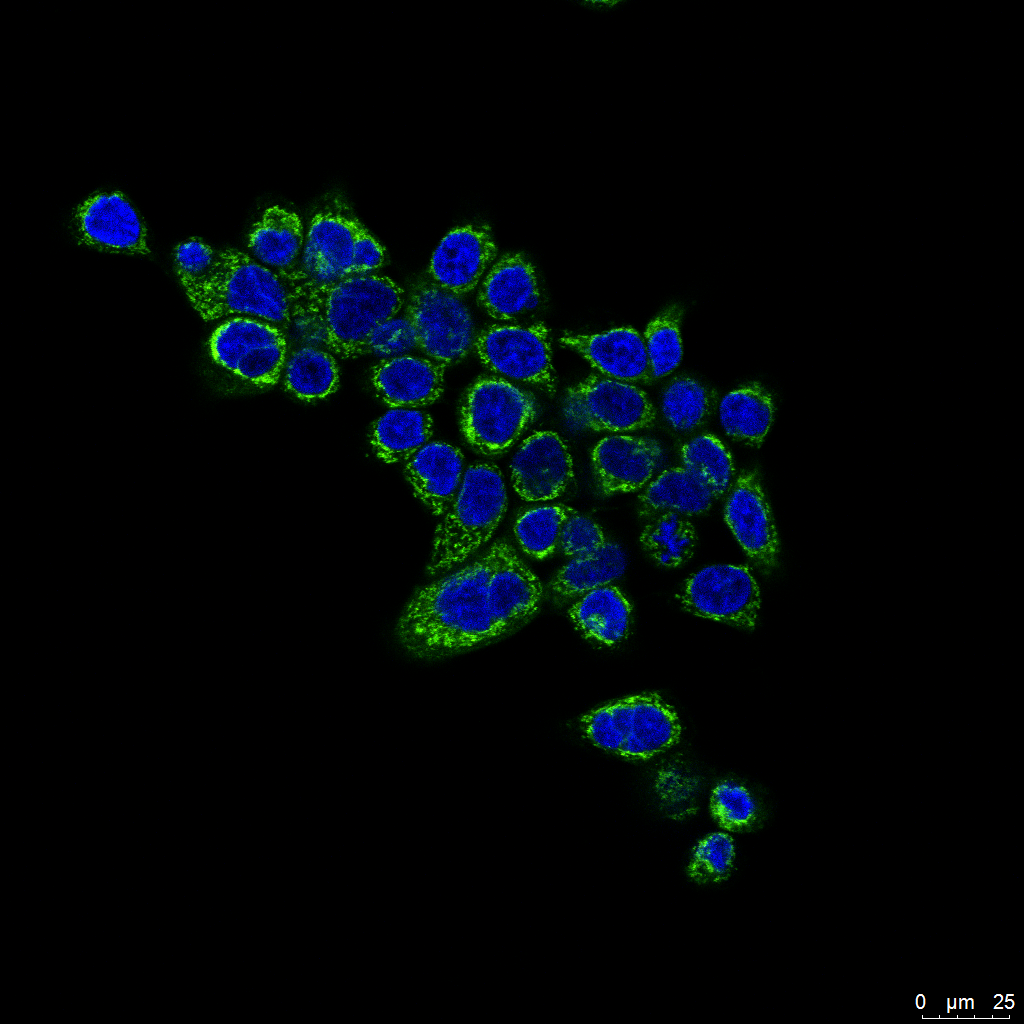

Supplement: Figure 2—figure supplement 5—source data 1. [file elife-70471-fig2-figsupp5-data1.zip › Figure 2-figure supplement 5-Source data 1/FaDu-hypoxia/201019_Series035 hypoxia.tif]

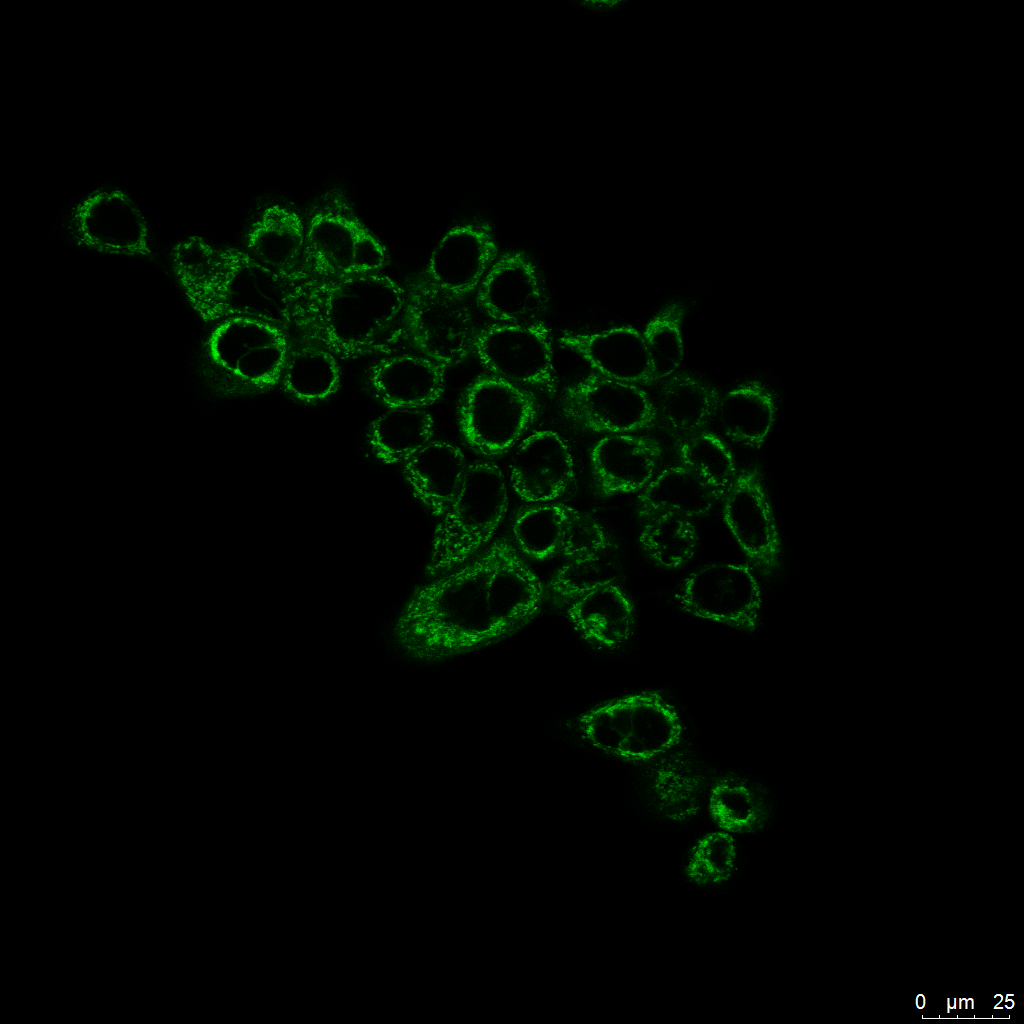

Supplement: Figure 2—figure supplement 5—source data 1. [file elife-70471-fig2-figsupp5-data1.zip › Figure 2-figure supplement 5-Source data 1/FaDu-hypoxia/201019_Series035 hypoxia_ch01.tif]

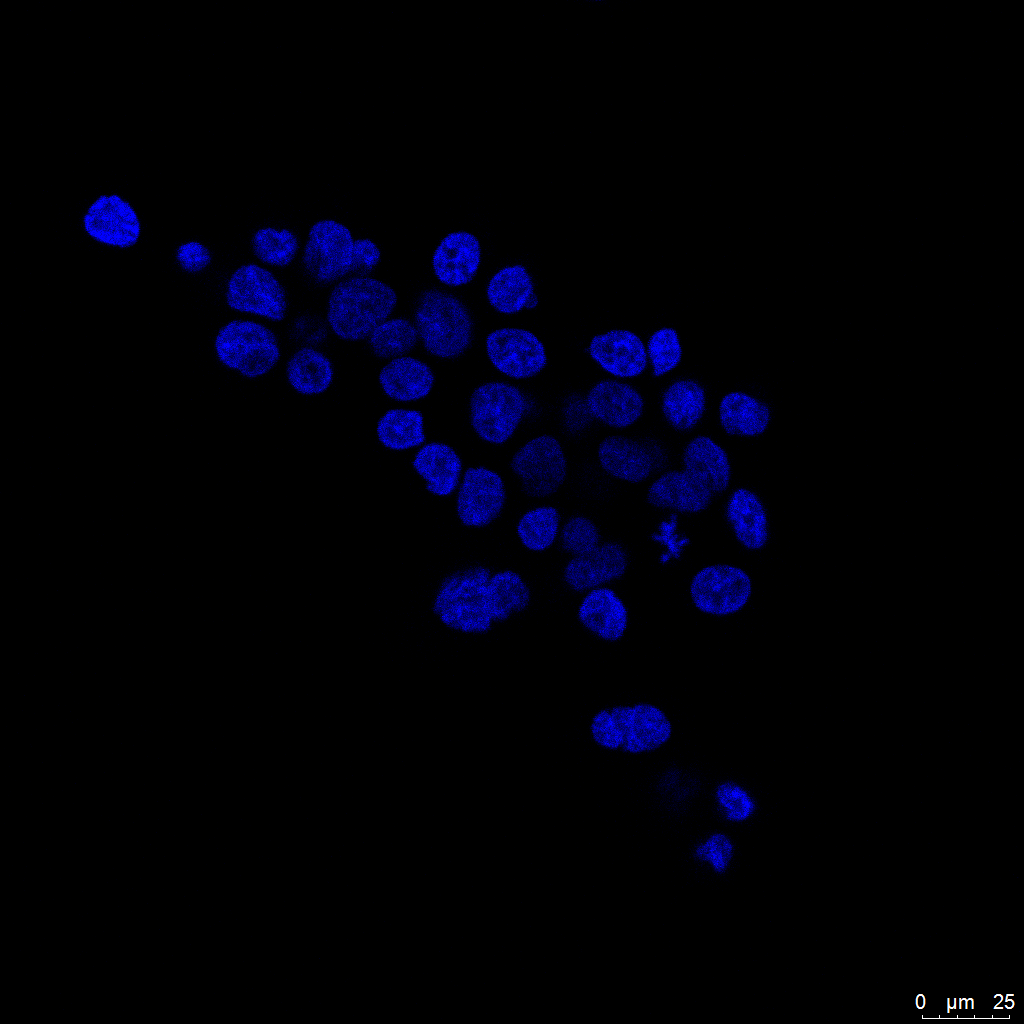

Supplement: Figure 2—figure supplement 5—source data 1. [file elife-70471-fig2-figsupp5-data1.zip › Figure 2-figure supplement 5-Source data 1/FaDu-hypoxia/201019_Series035 hypoxia_ch00.tif]

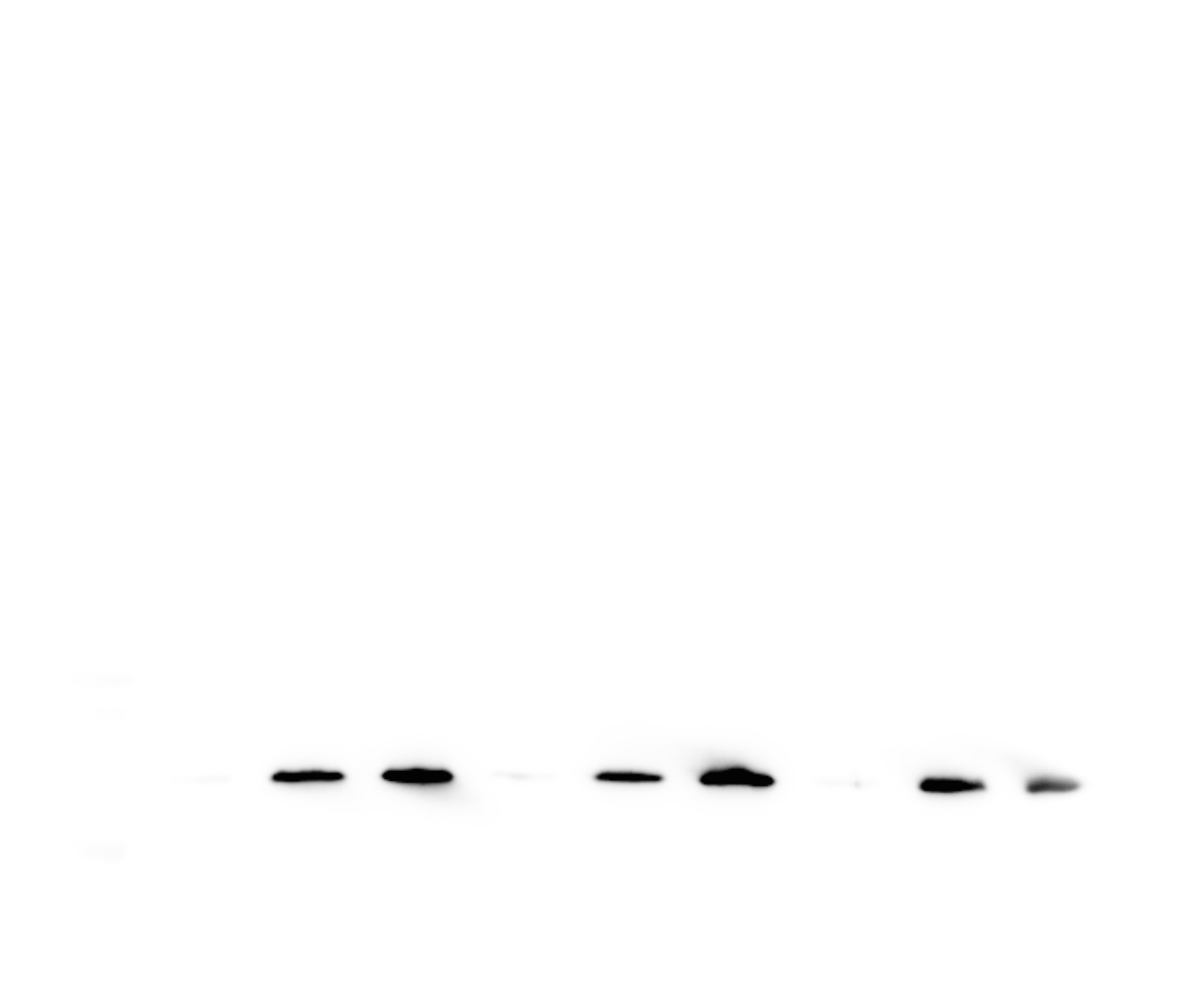

Supplement: Figure 2—figure supplement 5—source data 1. [file elife-70471-fig2-figsupp5-data1.zip › Figure 2-figure supplement 5-Source data 1/WB/HIF-1╬▒ expression level.jpg]

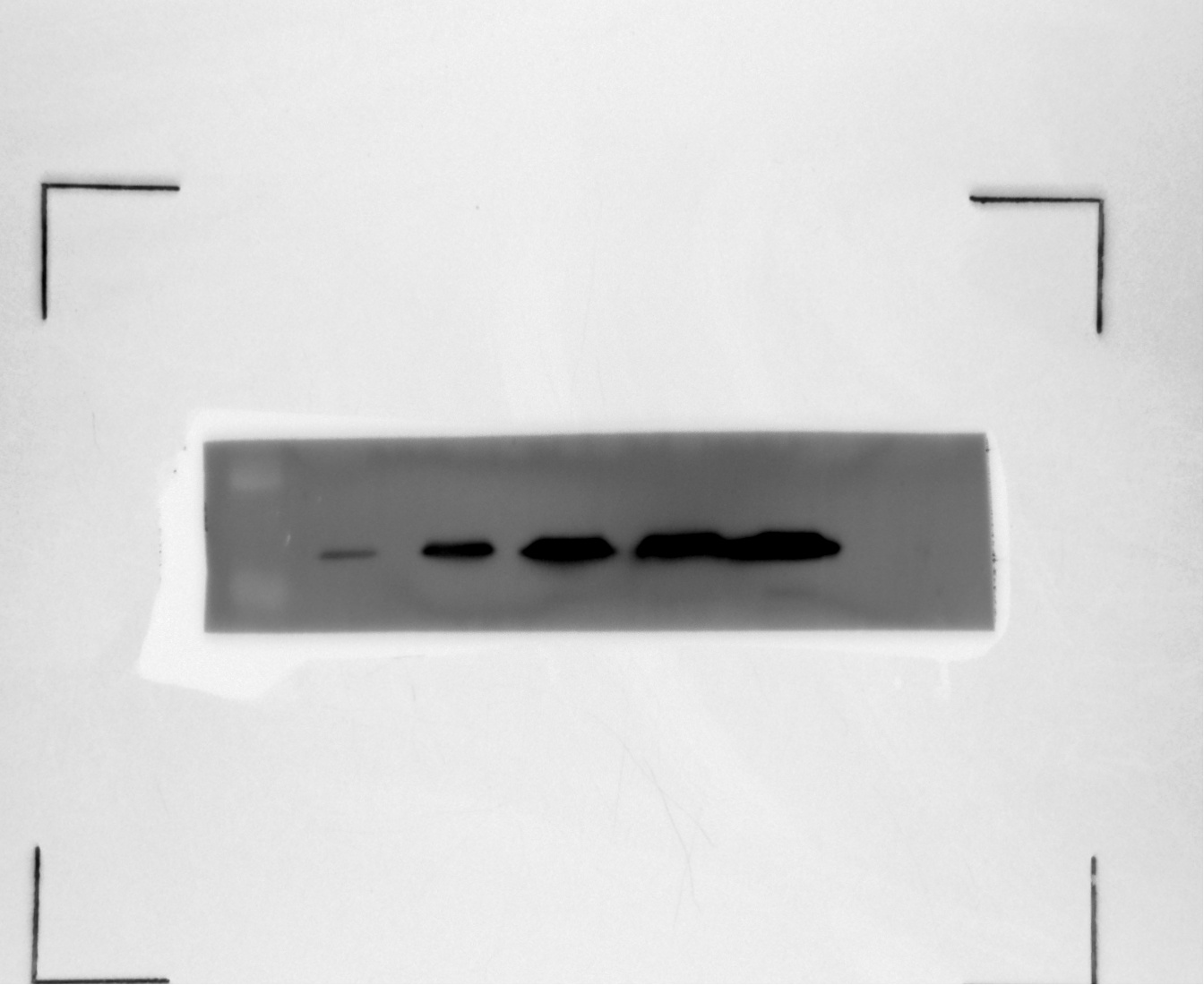

Supplement: Figure 2—figure supplement 5—source data 1. [file elife-70471-fig2-figsupp5-data1.zip › Figure 2-figure supplement 5-Source data 1/WB/Immunoblotting analysis of nitroreductase.jpg]

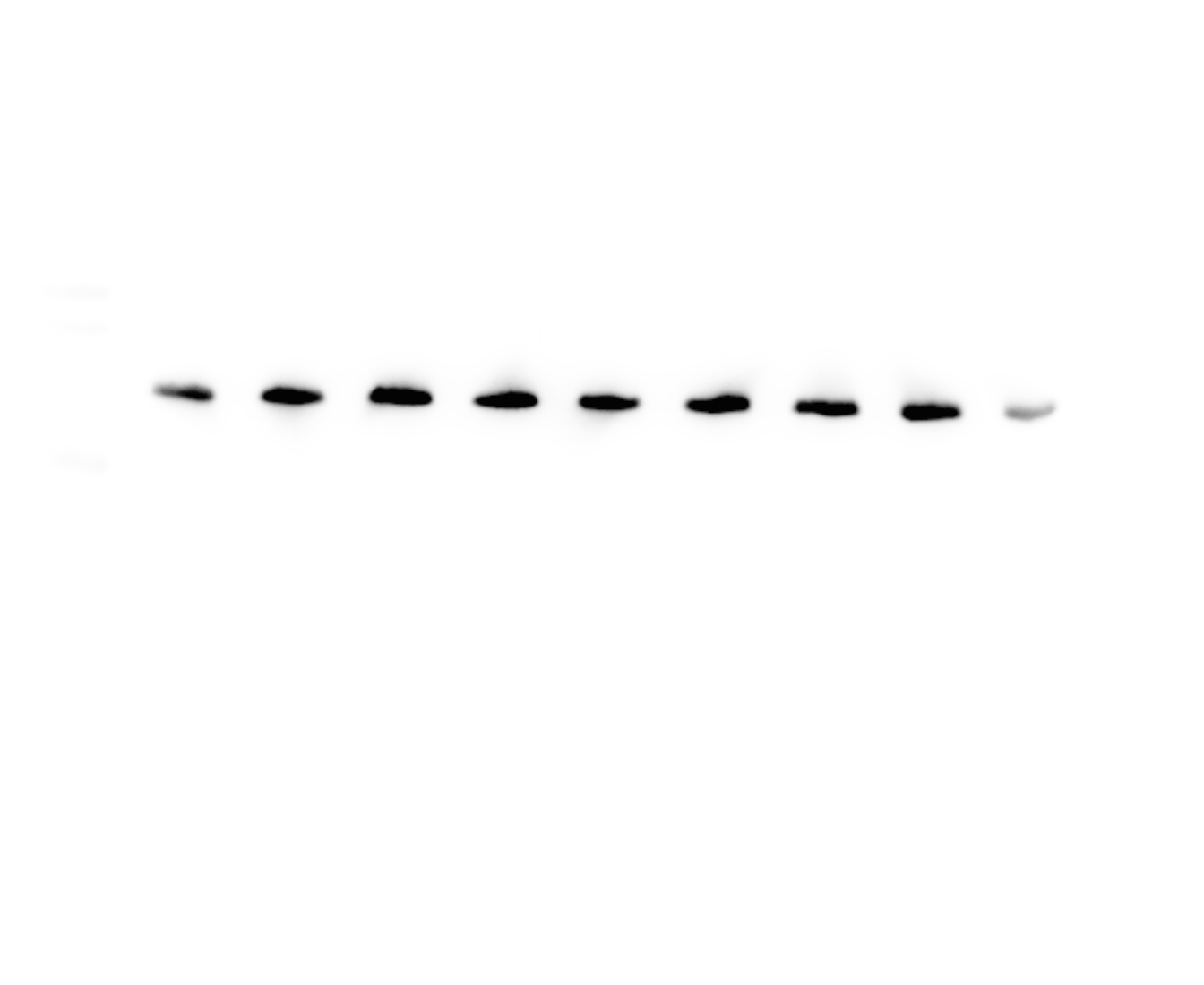

Supplement: Figure 2—figure supplement 5—source data 1. [file elife-70471-fig2-figsupp5-data1.zip › Figure 2-figure supplement 5-Source data 1/WB/╬▓-actin.jpg]

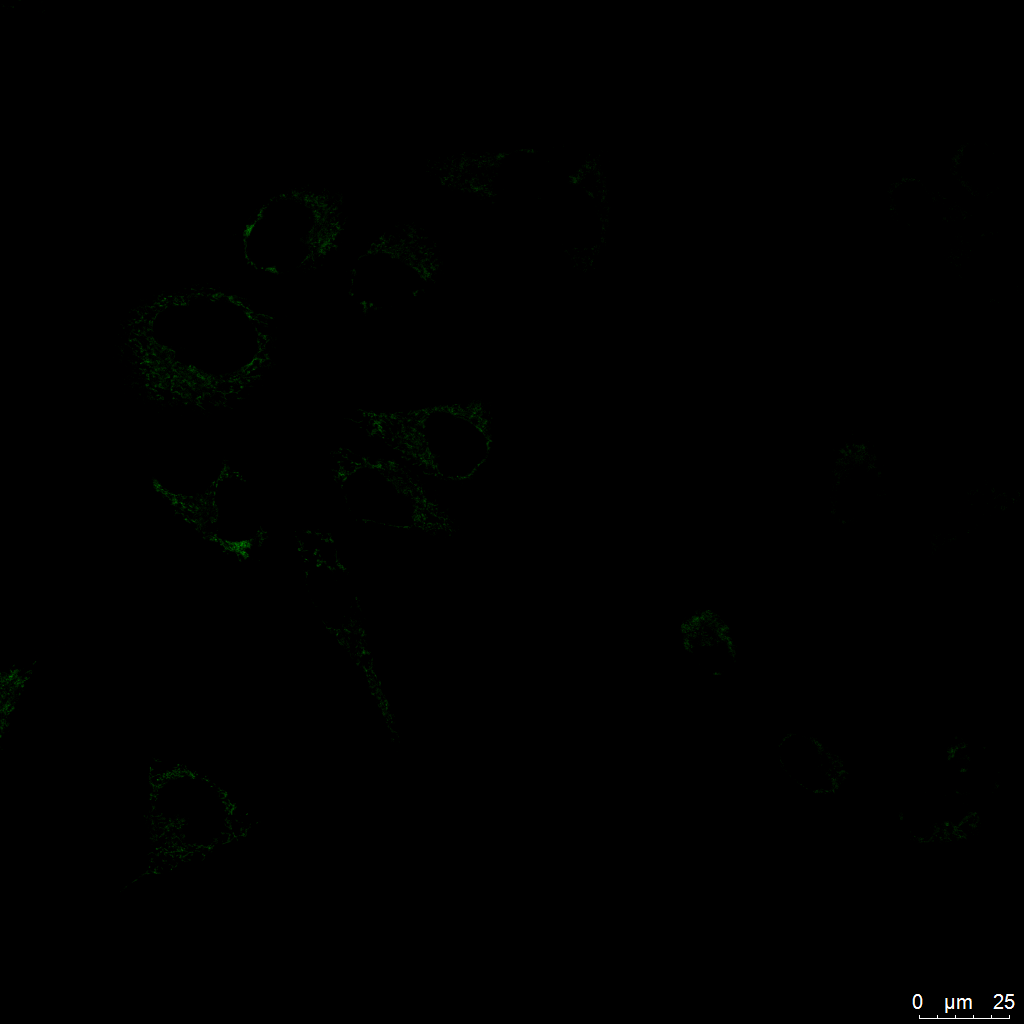

Supplement: Figure 2—figure supplement 5—source data 1. [file elife-70471-fig2-figsupp5-data1.zip › Figure 2-figure supplement 5-Source data 1/MHCC97H-normoxia/200921_17 normoxia_ch01.tif]

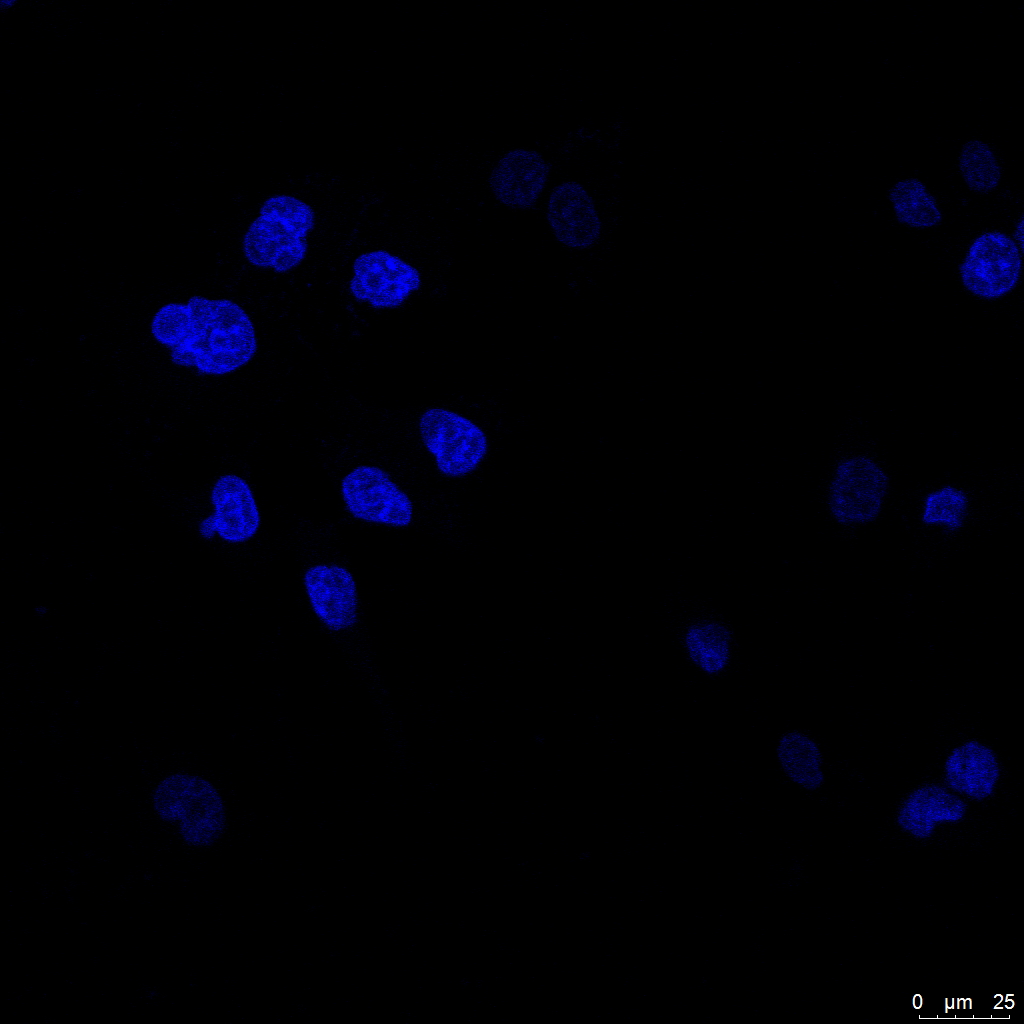

Supplement: Figure 2—figure supplement 5—source data 1. [file elife-70471-fig2-figsupp5-data1.zip › Figure 2-figure supplement 5-Source data 1/MHCC97H-normoxia/200921_17 normoxia_ch00.tif]

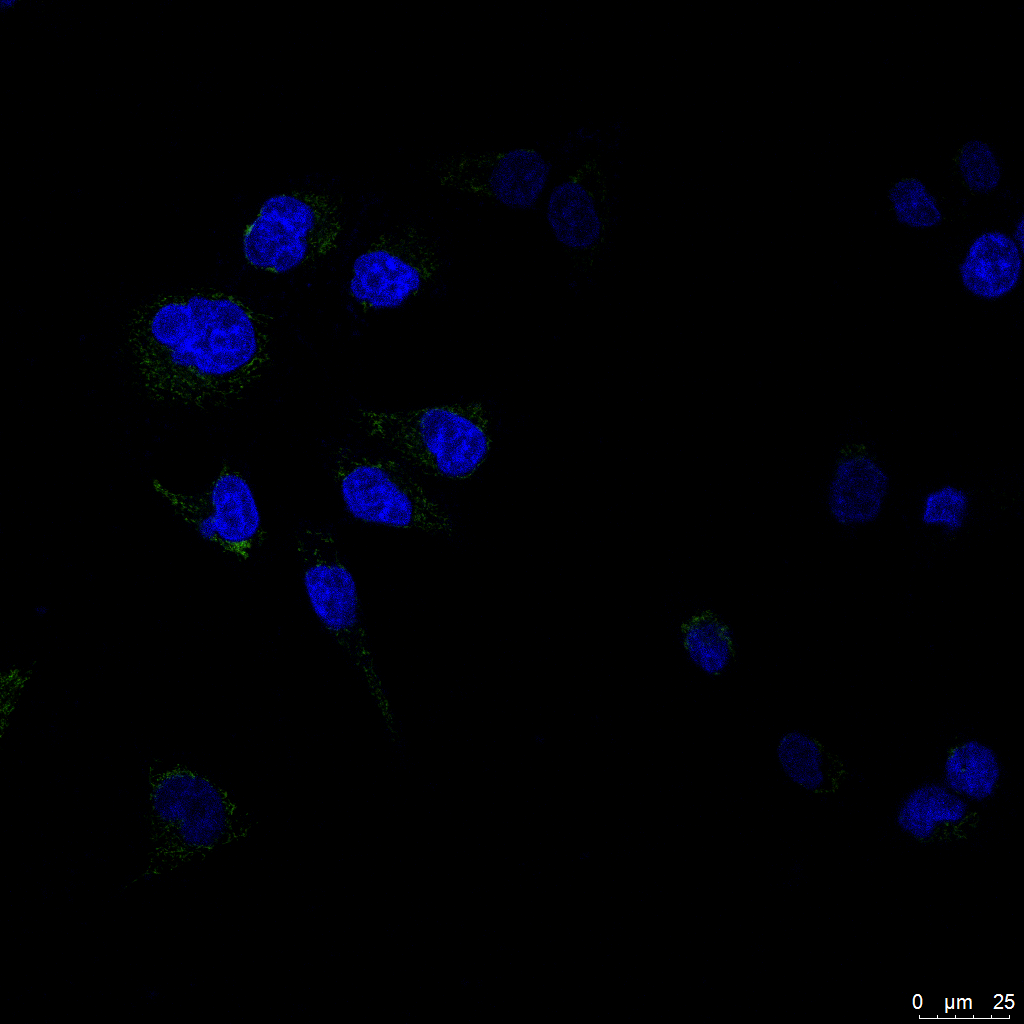

Supplement: Figure 2—figure supplement 5—source data 1. [file elife-70471-fig2-figsupp5-data1.zip › Figure 2-figure supplement 5-Source data 1/MHCC97H-normoxia/200921_17 normoxia.tif]

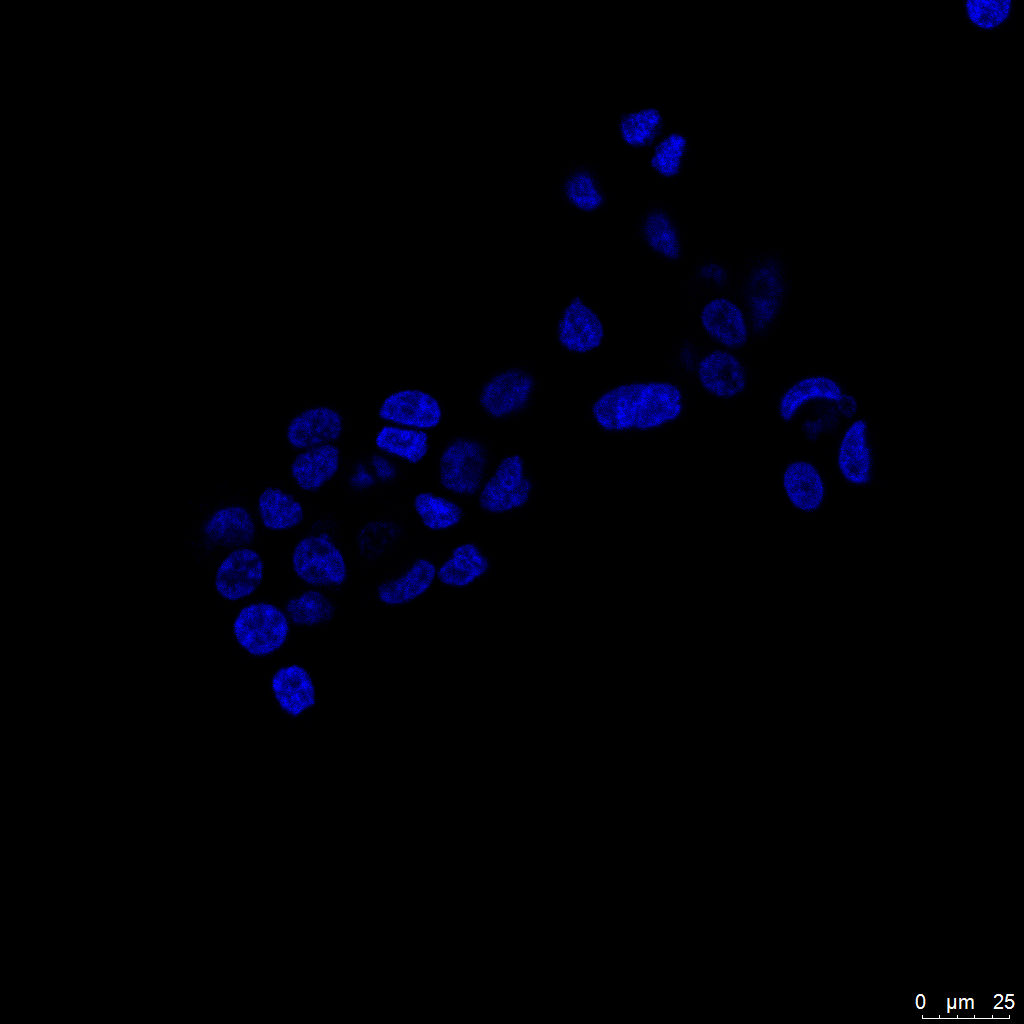

Supplement: Figure 2—figure supplement 5—source data 1. [file elife-70471-fig2-figsupp5-data1.zip › Figure 2-figure supplement 5-Source data 1/FaDu-normoxia/201019_Series081 normoxia_ch00.tif]

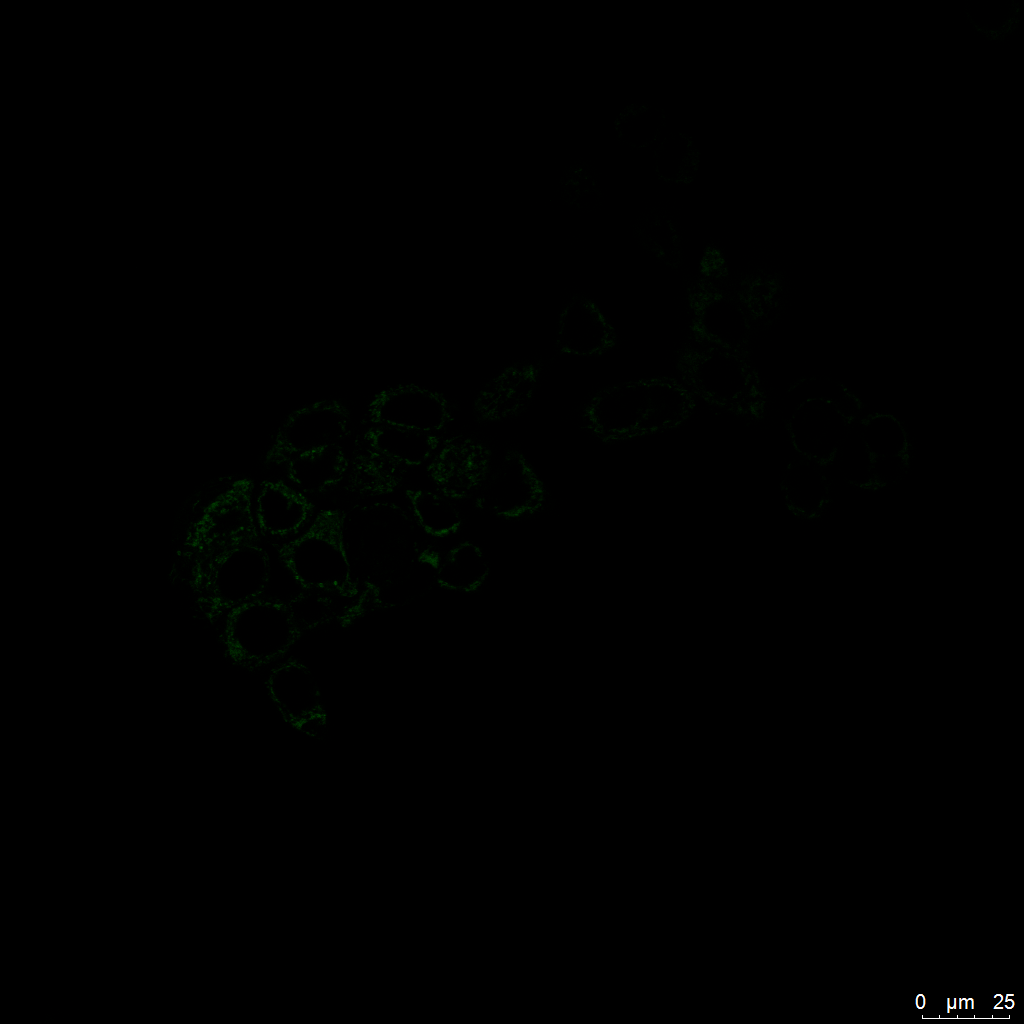

Supplement: Figure 2—figure supplement 5—source data 1. [file elife-70471-fig2-figsupp5-data1.zip › Figure 2-figure supplement 5-Source data 1/FaDu-normoxia/201019_Series081 normoxia_ch01.tif]

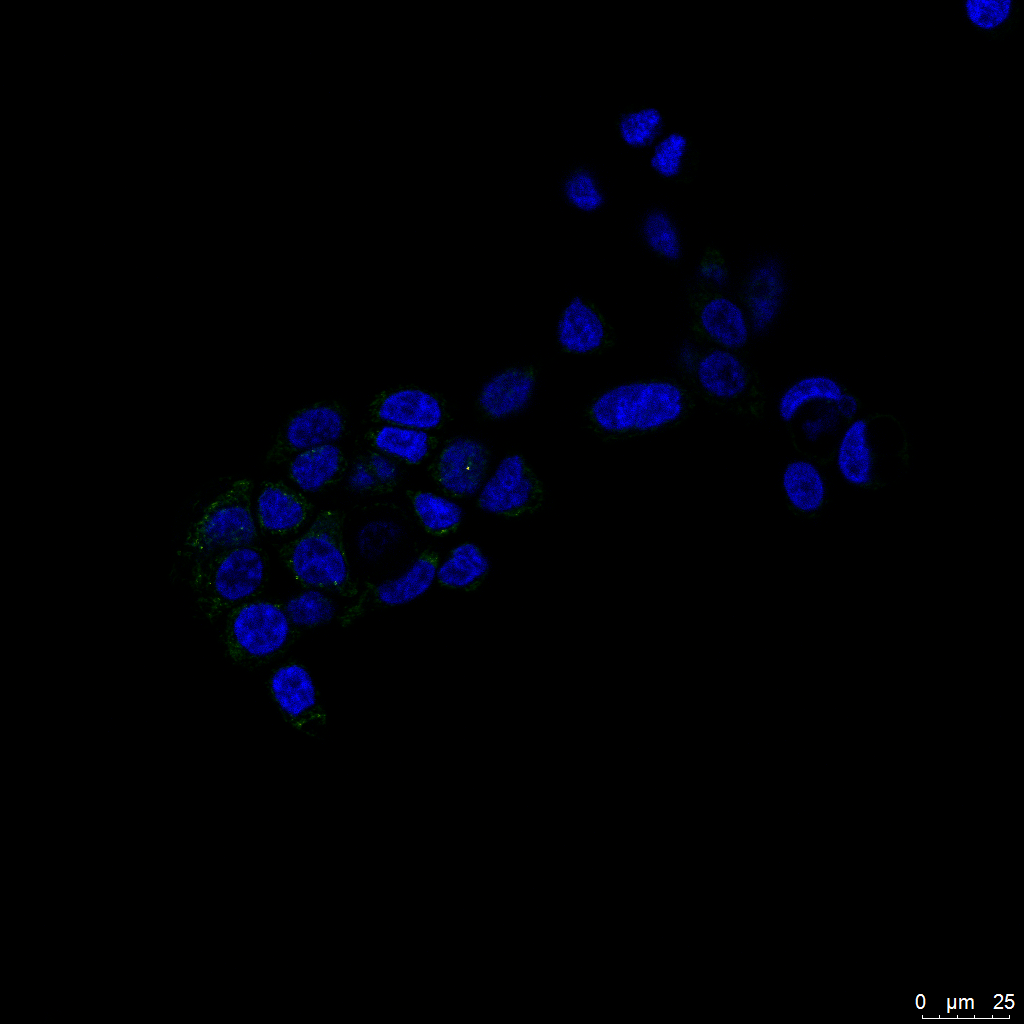

Supplement: Figure 2—figure supplement 5—source data 1. [file elife-70471-fig2-figsupp5-data1.zip › Figure 2-figure supplement 5-Source data 1/FaDu-normoxia/201019_Series081 normoxia.tif]

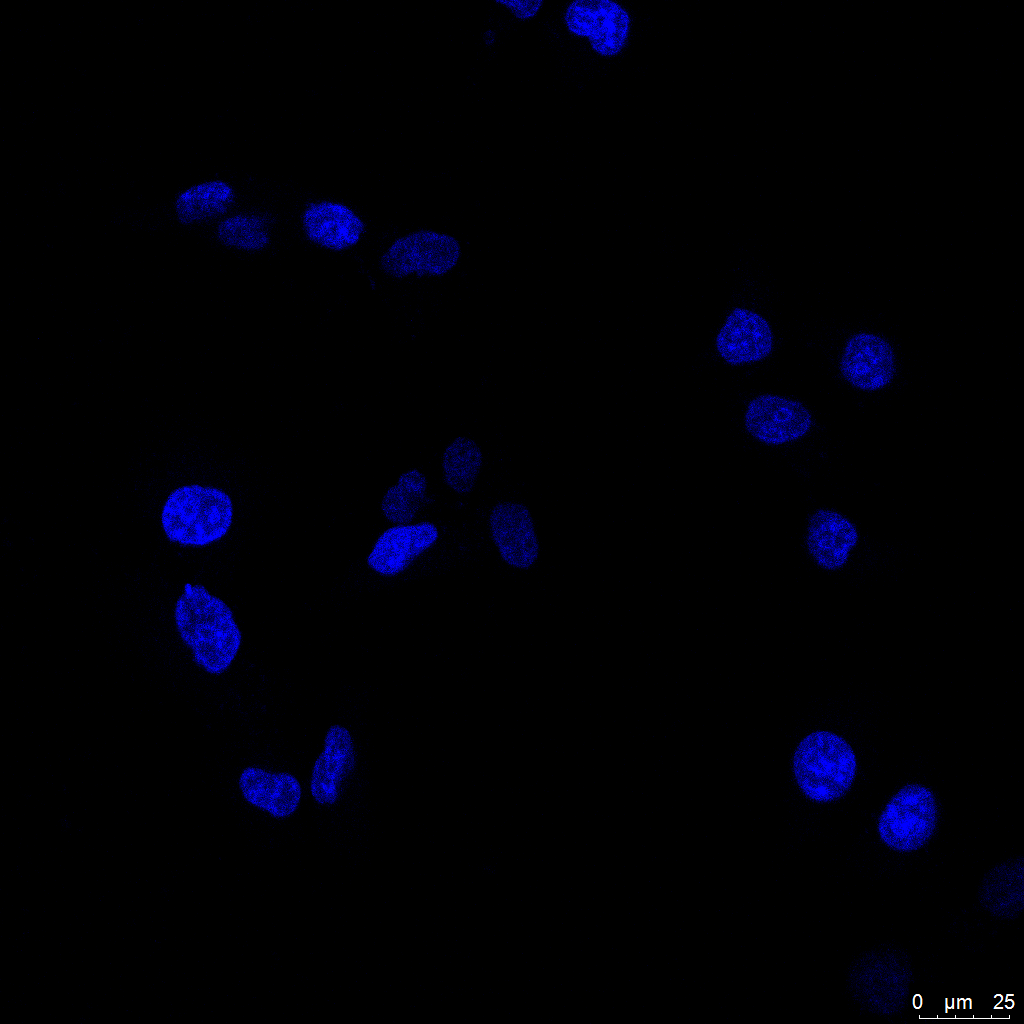

Supplement: Figure 2—figure supplement 5—source data 1. [file elife-70471-fig2-figsupp5-data1.zip › Figure 2-figure supplement 5-Source data 1/MHCC97H-hypoxia/200921_15 hypoxia_ch00.tif]

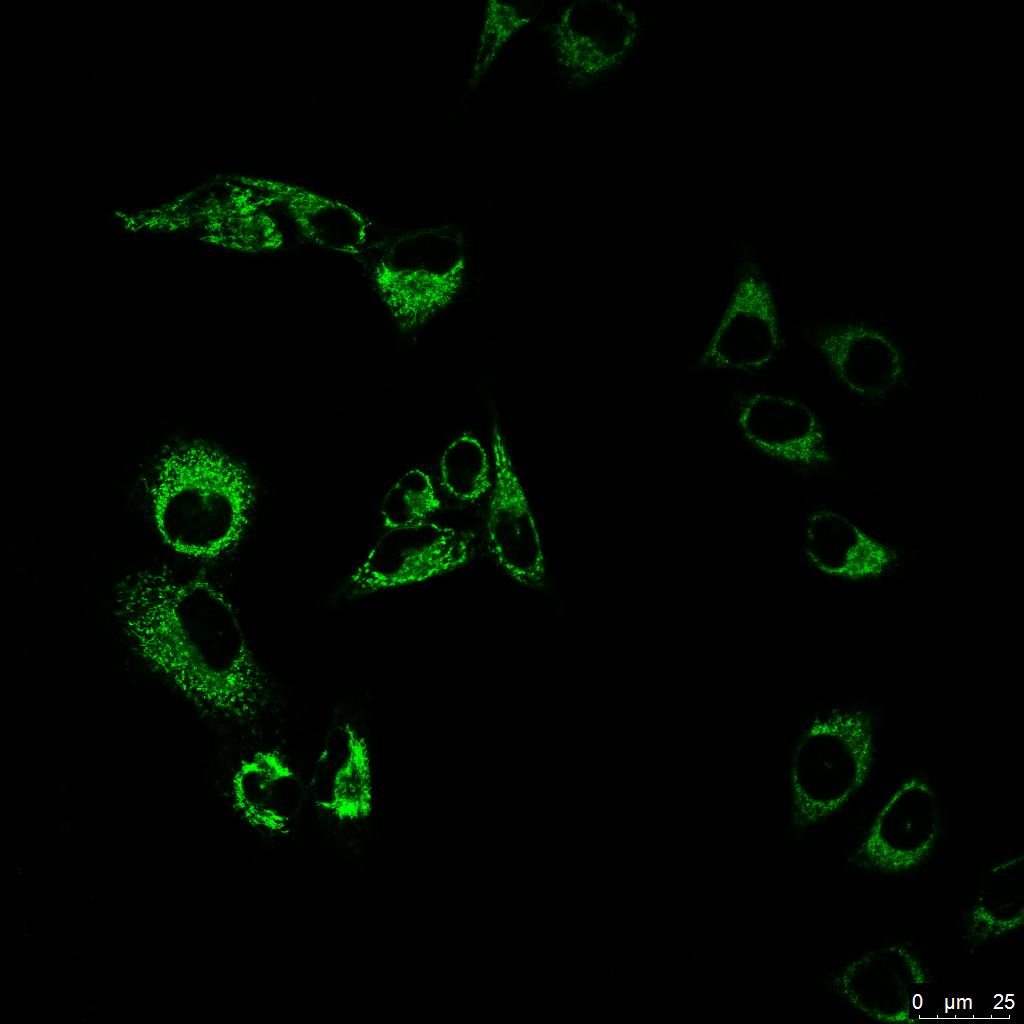

Supplement: Figure 2—figure supplement 5—source data 1. [file elife-70471-fig2-figsupp5-data1.zip › Figure 2-figure supplement 5-Source data 1/MHCC97H-hypoxia/200921_15 hypoxia_ch01.tif]

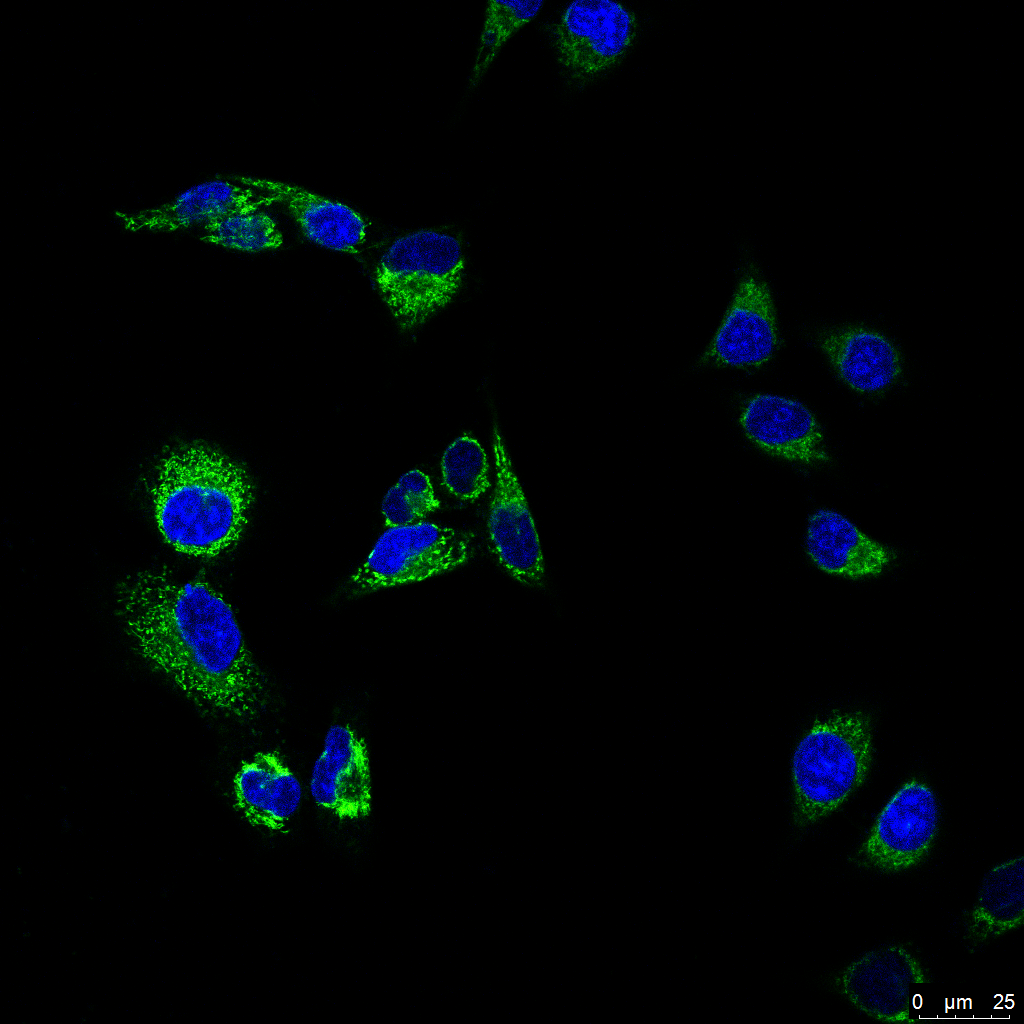

Supplement: Figure 2—figure supplement 5—source data 1. [file elife-70471-fig2-figsupp5-data1.zip › Figure 2-figure supplement 5-Source data 1/MHCC97H-hypoxia/200921_15 hypoxia.tif]

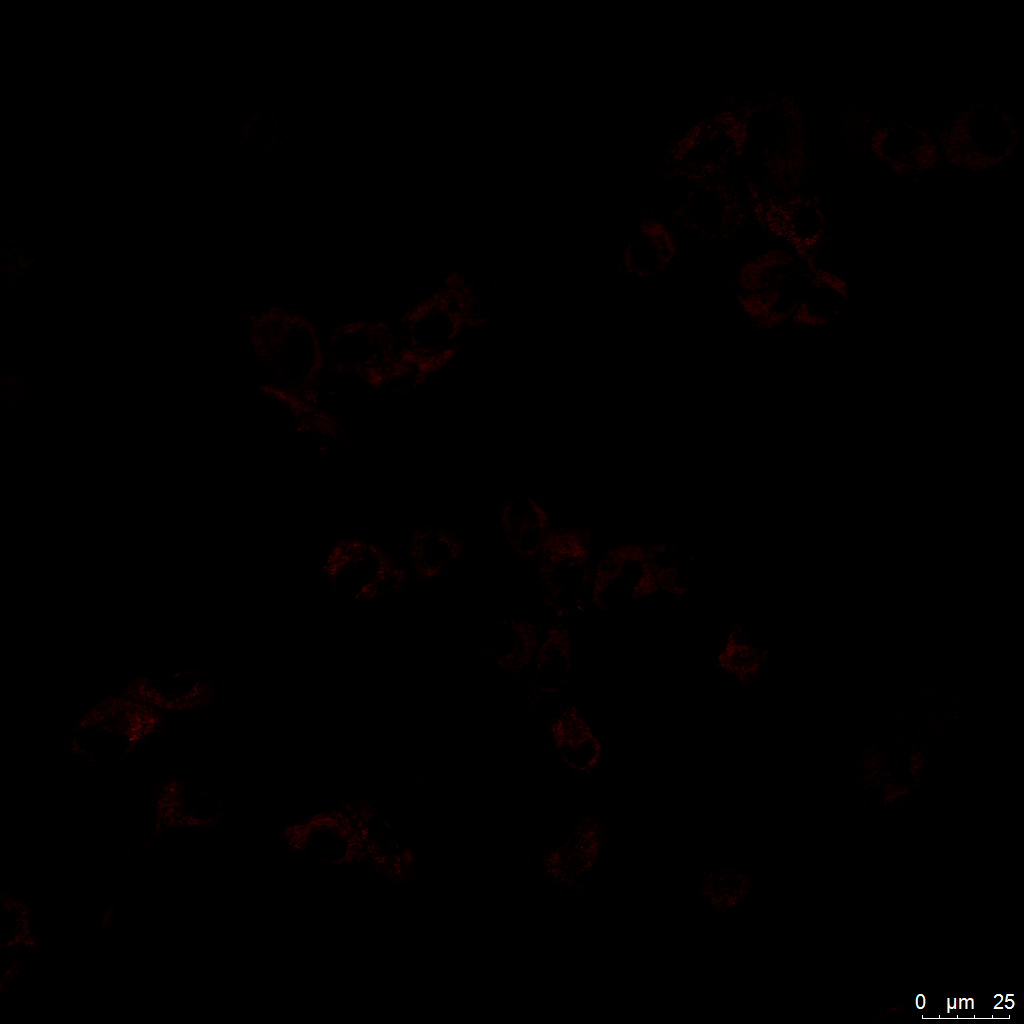

Supplement: Figure 2—figure supplement 6—source data 1. [file elife-70471-fig2-figsupp6-data1.zip › Figure 2-figure supplement 6-Source data 1/A7r5/A7r5 1_ch01.tif]

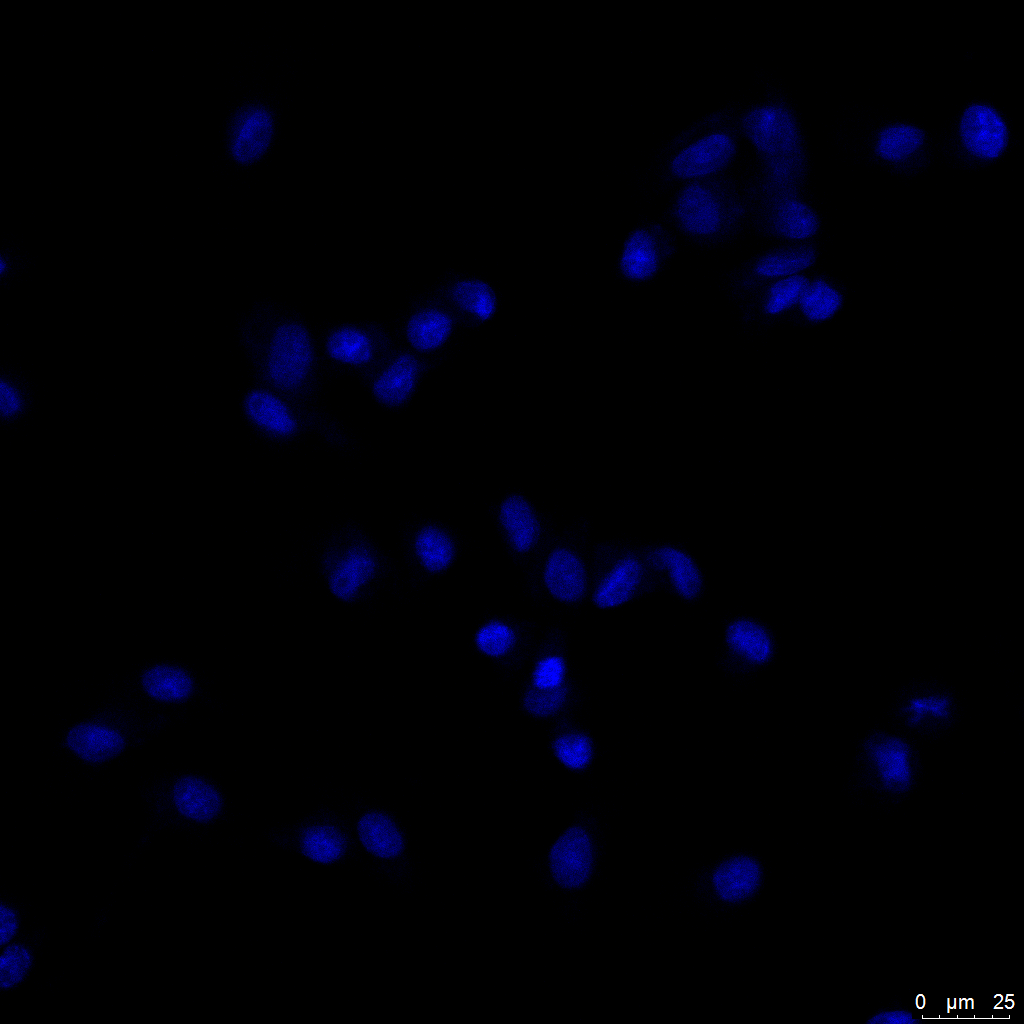

Supplement: Figure 2—figure supplement 6—source data 1. [file elife-70471-fig2-figsupp6-data1.zip › Figure 2-figure supplement 6-Source data 1/A7r5/A7r5 1_ch00.tif]

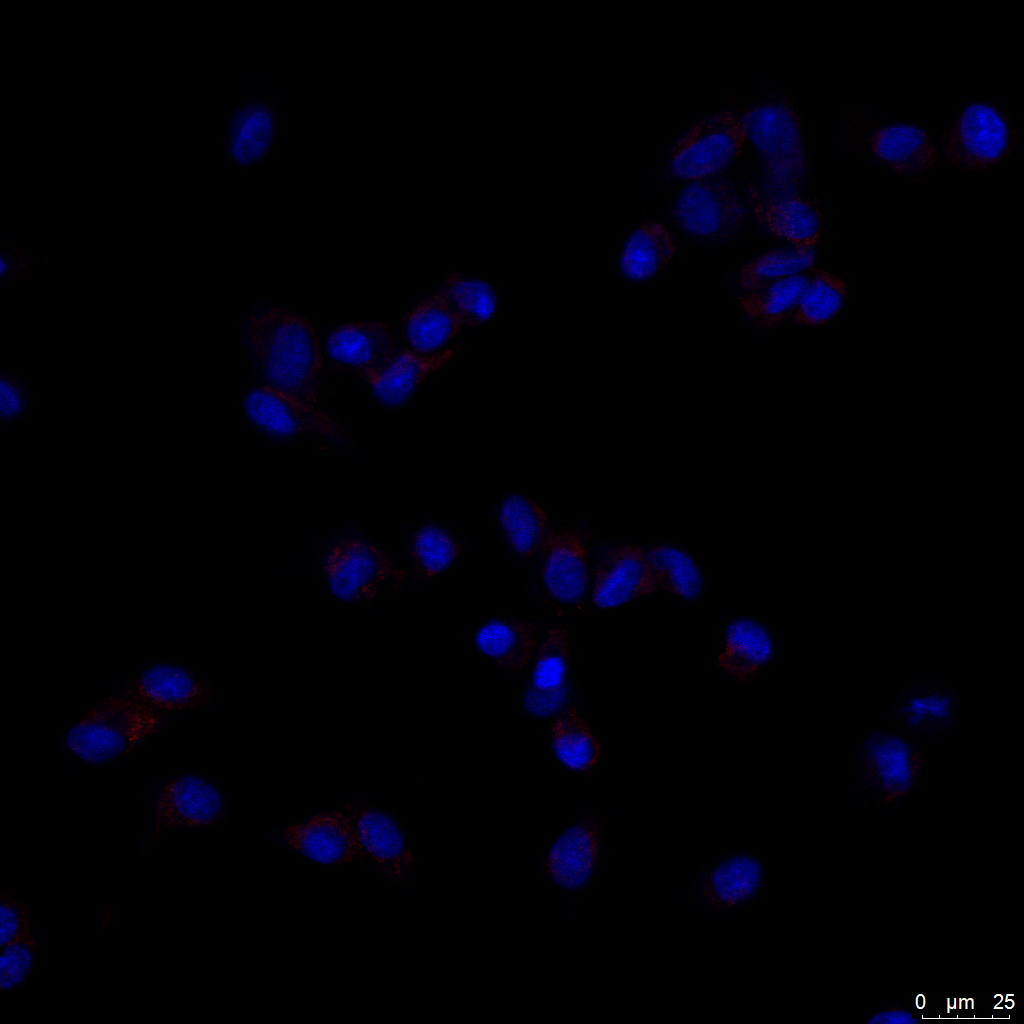

Supplement: Figure 2—figure supplement 6—source data 1. [file elife-70471-fig2-figsupp6-data1.zip › Figure 2-figure supplement 6-Source data 1/A7r5/A7r5 1.tif]

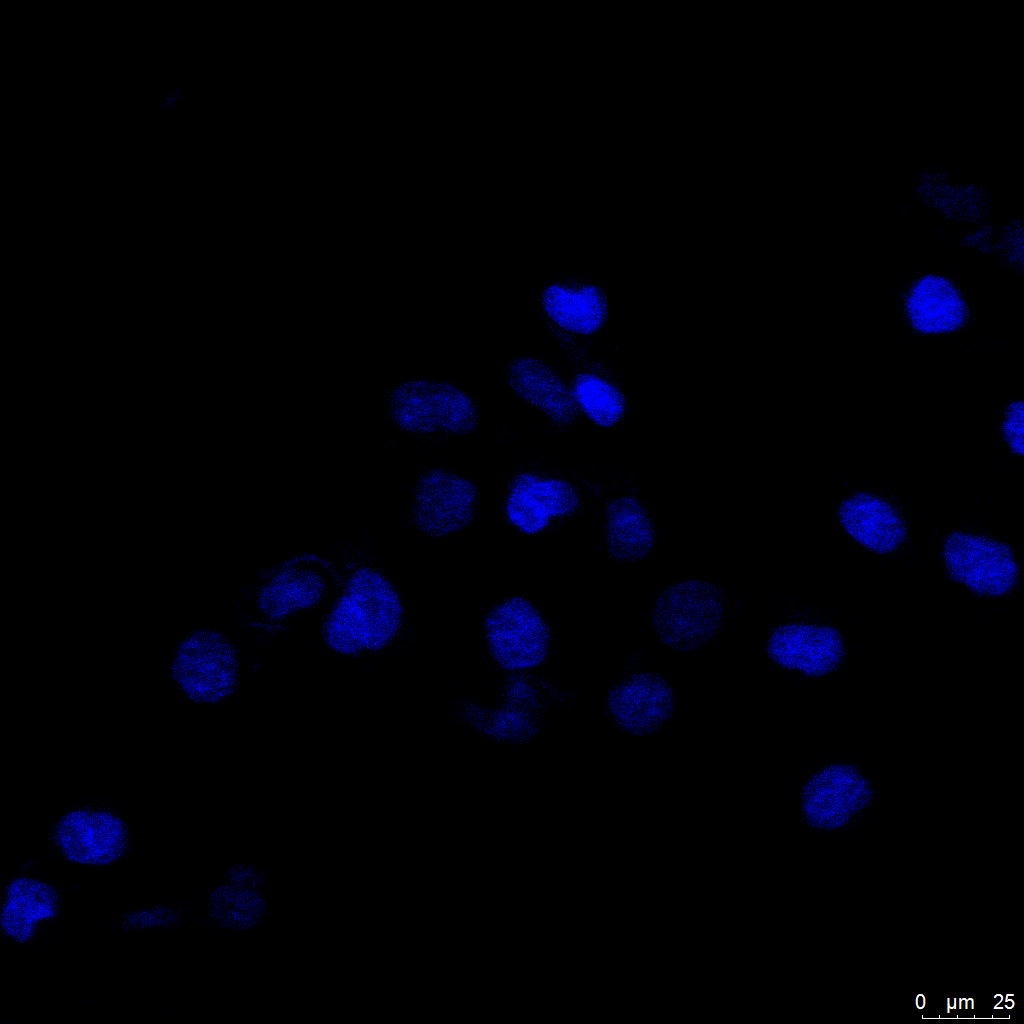

Supplement: Figure 2—figure supplement 6—source data 1. [file elife-70471-fig2-figsupp6-data1.zip › Figure 2-figure supplement 6-Source data 1/97H/97H 1_ch00.tif]

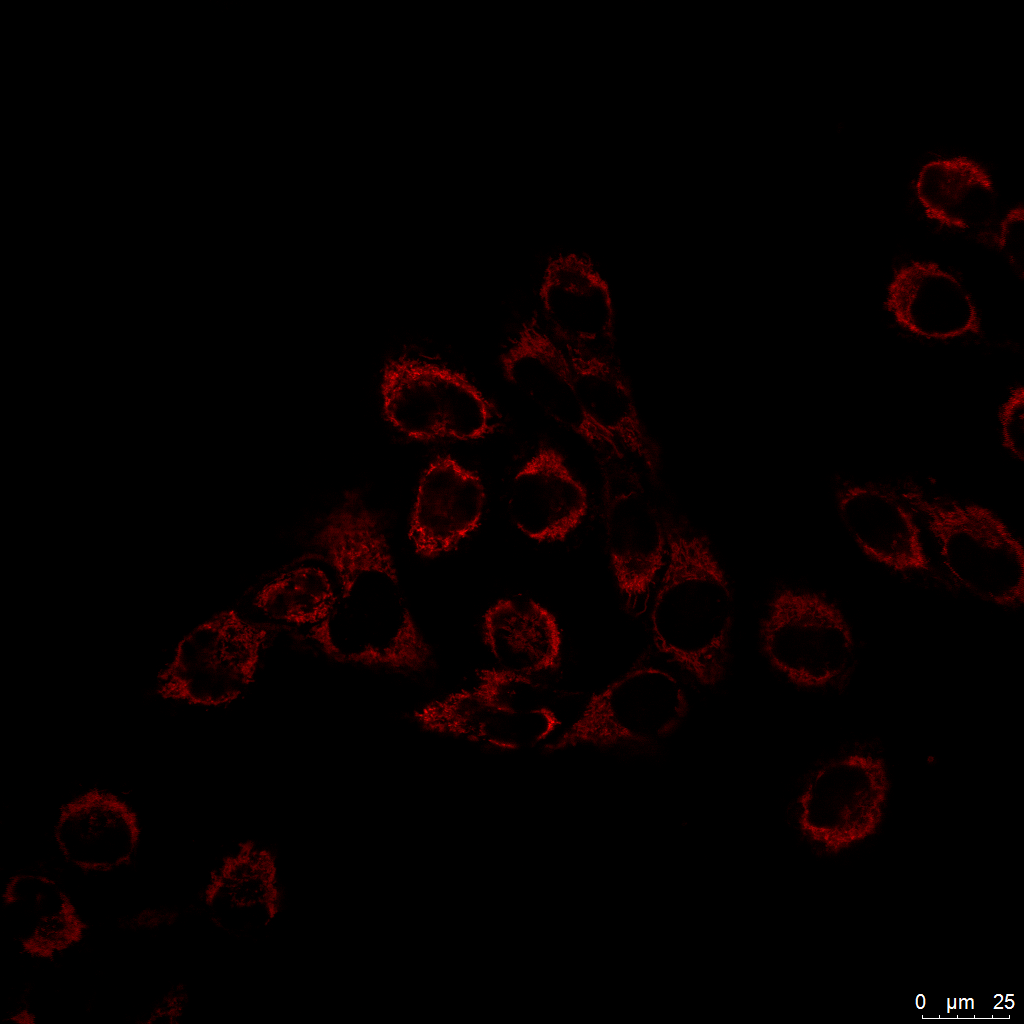

Supplement: Figure 2—figure supplement 6—source data 1. [file elife-70471-fig2-figsupp6-data1.zip › Figure 2-figure supplement 6-Source data 1/97H/97H 1_ch01.tif]

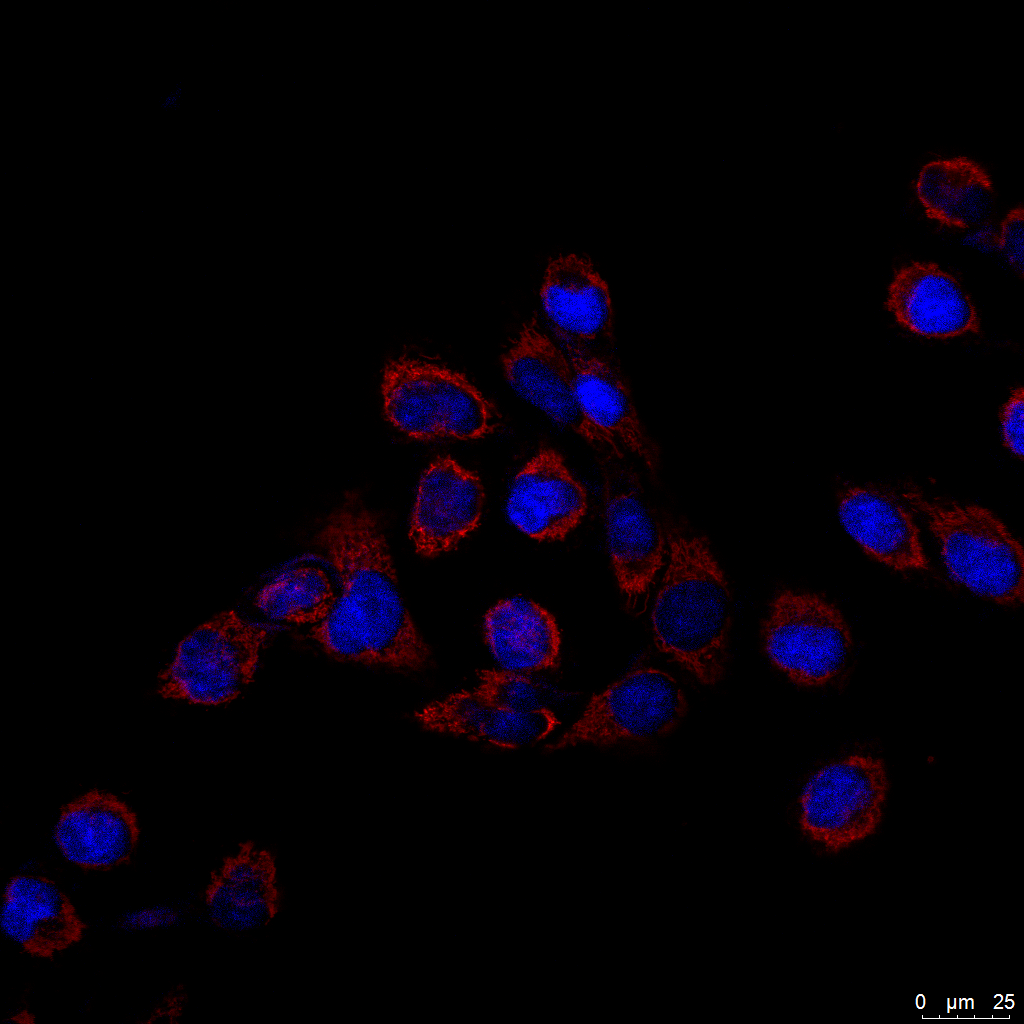

Supplement: Figure 2—figure supplement 6—source data 1. [file elife-70471-fig2-figsupp6-data1.zip › Figure 2-figure supplement 6-Source data 1/97H/97H 1.tif]

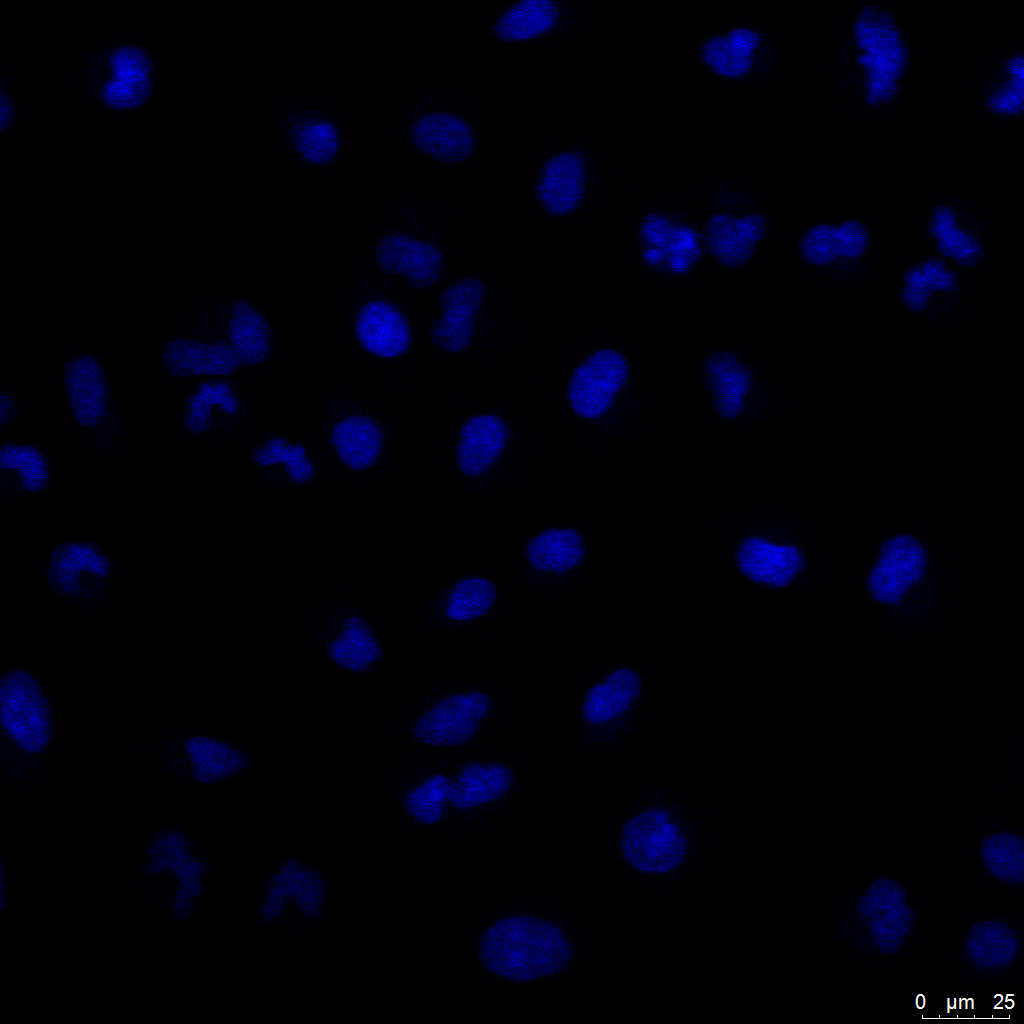

Supplement: Figure 2—figure supplement 6—source data 1. [file elife-70471-fig2-figsupp6-data1.zip › Figure 2-figure supplement 6-Source data 1/A549/A549_ch00.tif]

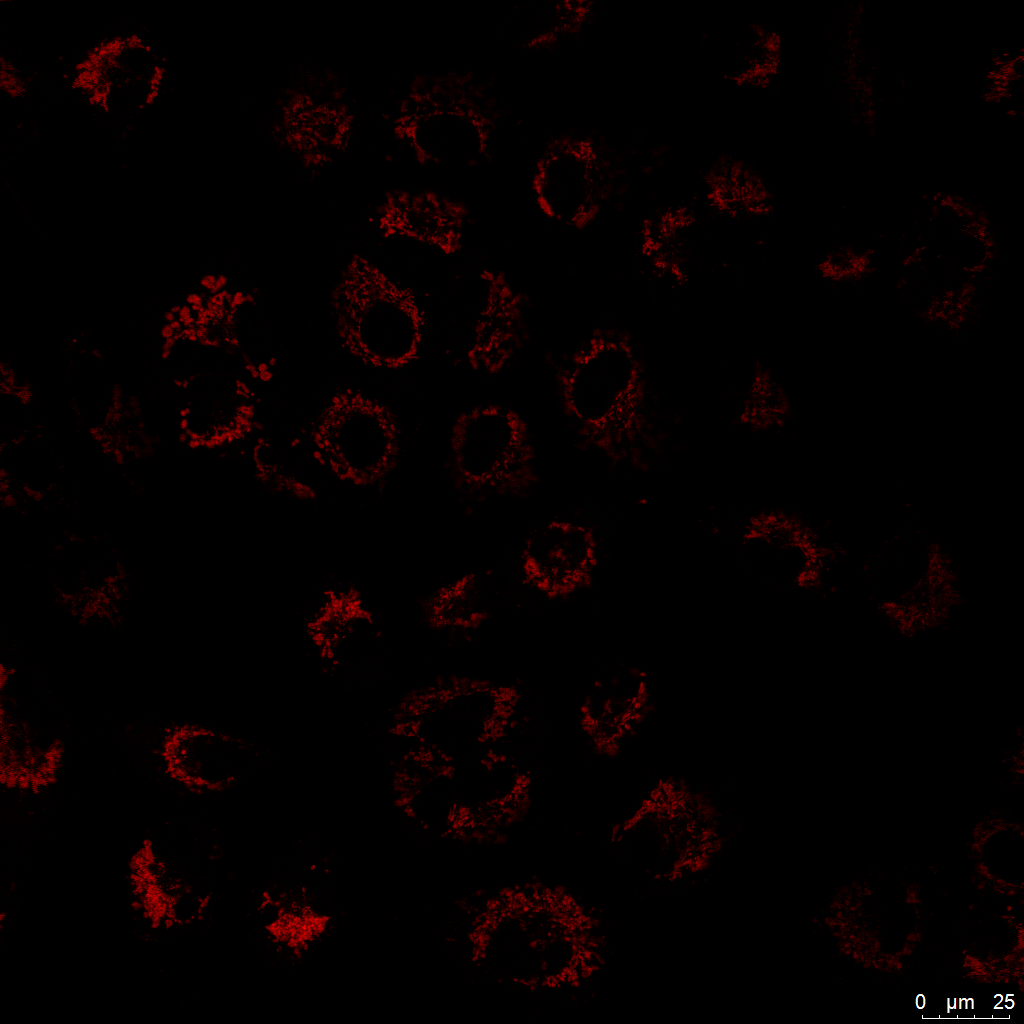

Supplement: Figure 2—figure supplement 6—source data 1. [file elife-70471-fig2-figsupp6-data1.zip › Figure 2-figure supplement 6-Source data 1/A549/A549_ch01.tif]

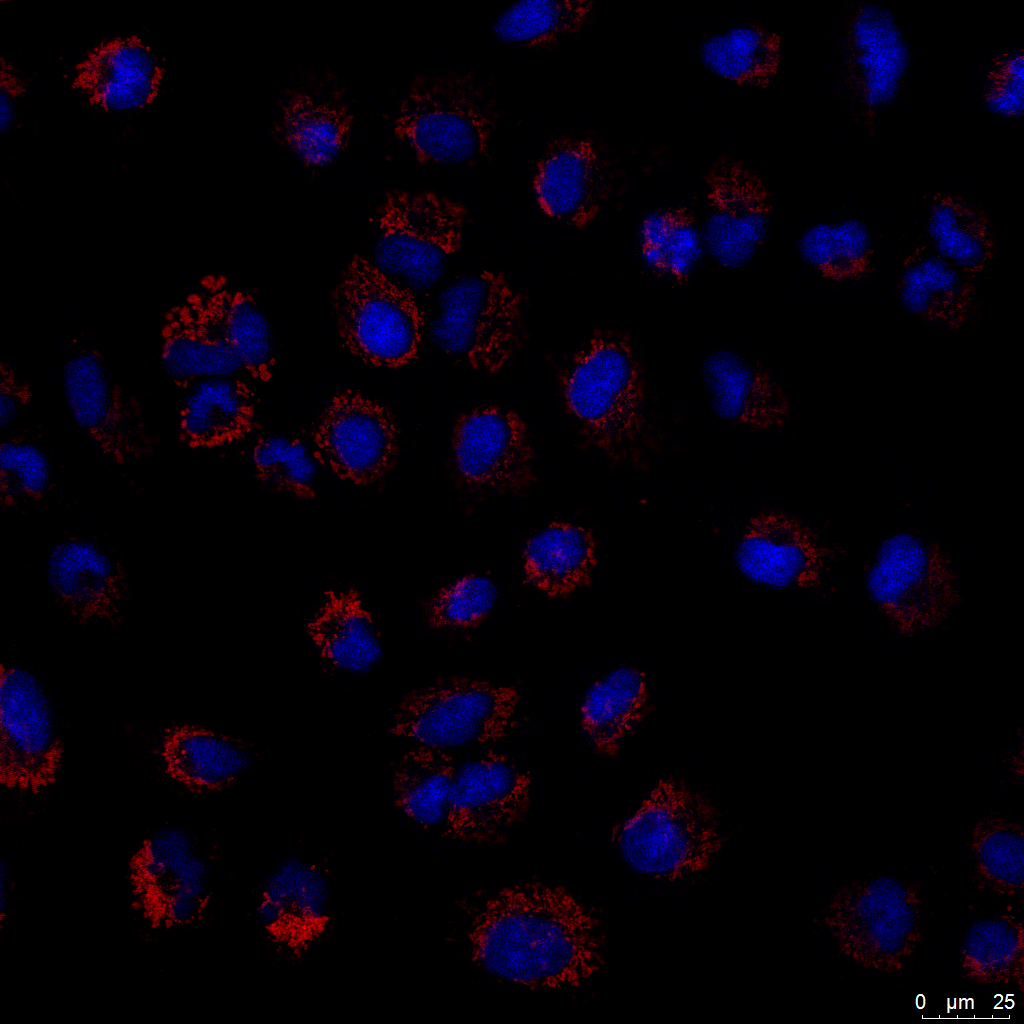

Supplement: Figure 2—figure supplement 6—source data 1. [file elife-70471-fig2-figsupp6-data1.zip › Figure 2-figure supplement 6-Source data 1/A549/A549.tif]

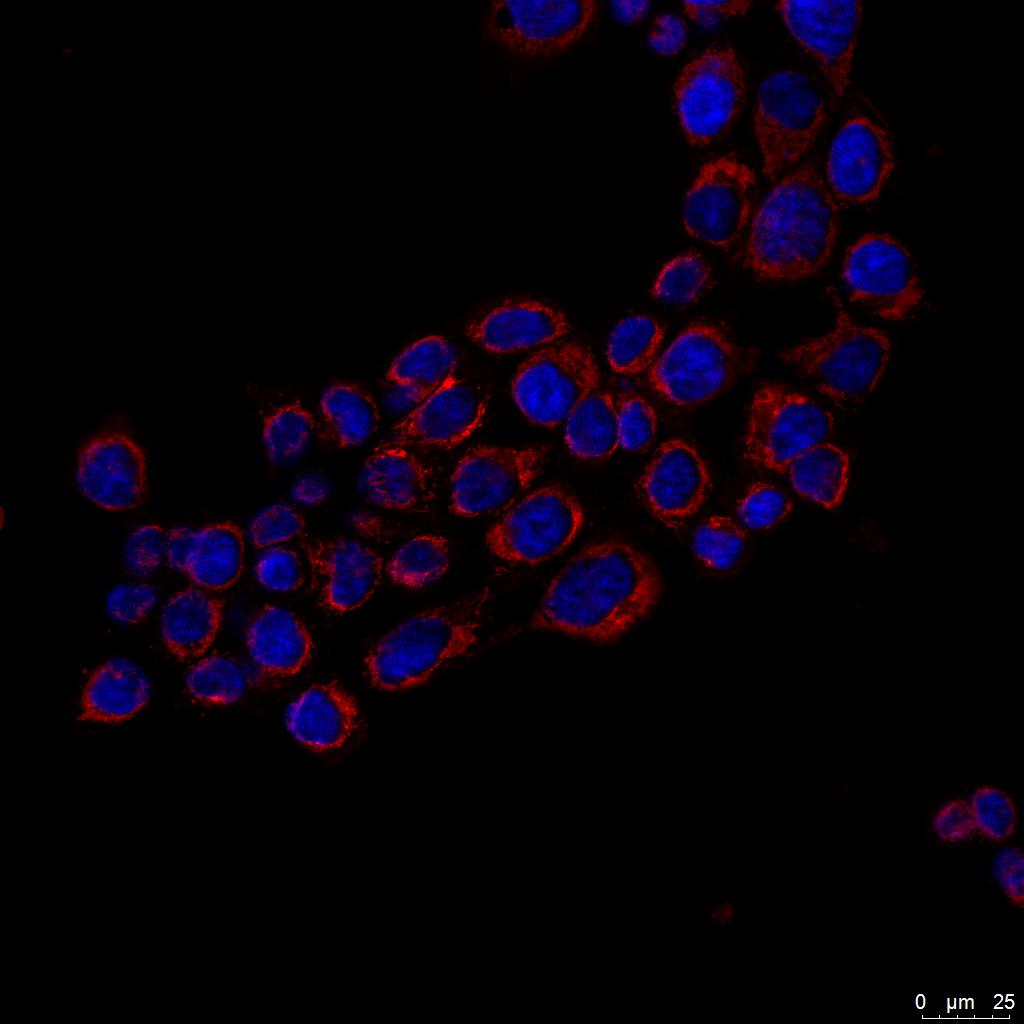

Supplement: Figure 2—figure supplement 6—source data 1. [file elife-70471-fig2-figsupp6-data1.zip › Figure 2-figure supplement 6-Source data 1/FaDu/FaDu 4.tif]

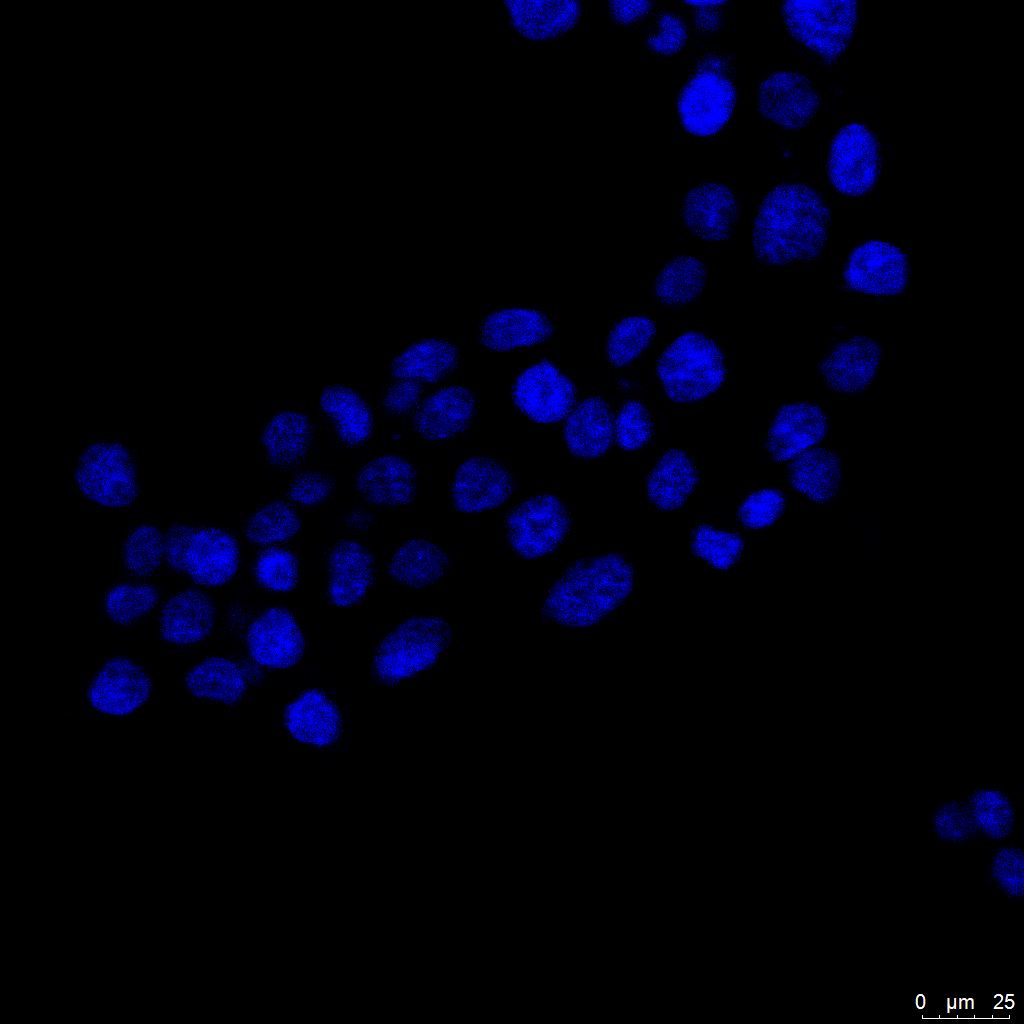

Supplement: Figure 2—figure supplement 6—source data 1. [file elife-70471-fig2-figsupp6-data1.zip › Figure 2-figure supplement 6-Source data 1/FaDu/FaDu 4_ch00.tif]

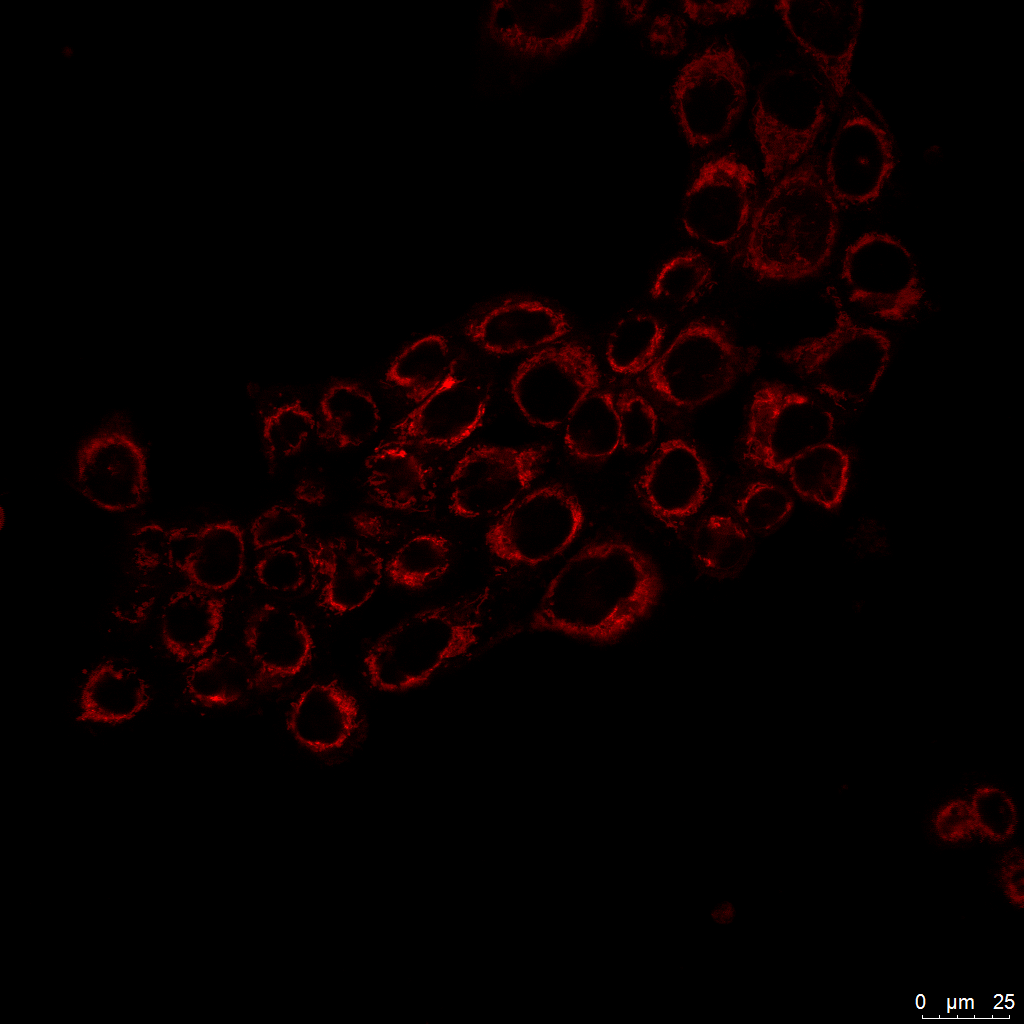

Supplement: Figure 2—figure supplement 6—source data 1. [file elife-70471-fig2-figsupp6-data1.zip › Figure 2-figure supplement 6-Source data 1/FaDu/FaDu 4_ch01.tif]

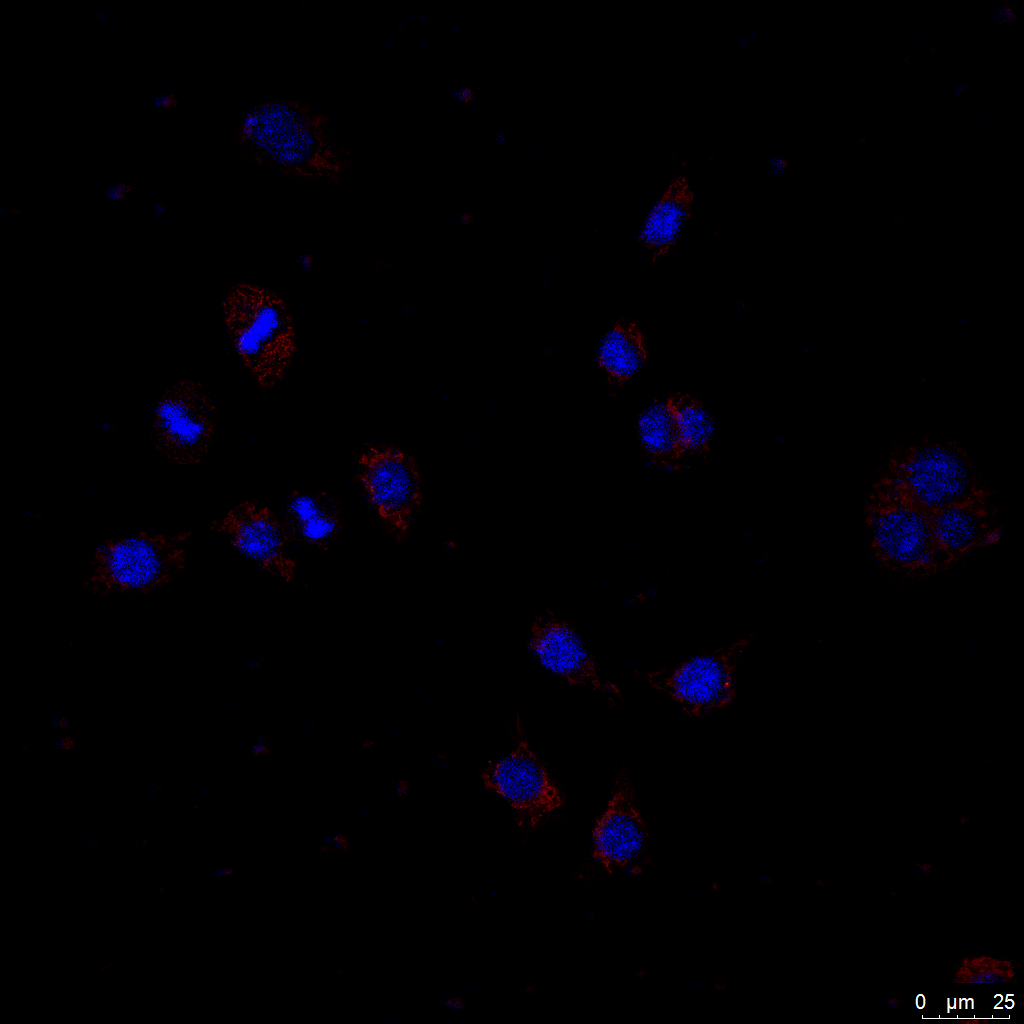

Supplement: Figure 2—figure supplement 6—source data 1. [file elife-70471-fig2-figsupp6-data1.zip › Figure 2-figure supplement 6-Source data 1/RAW264.7/RAW264.7 2.tif]

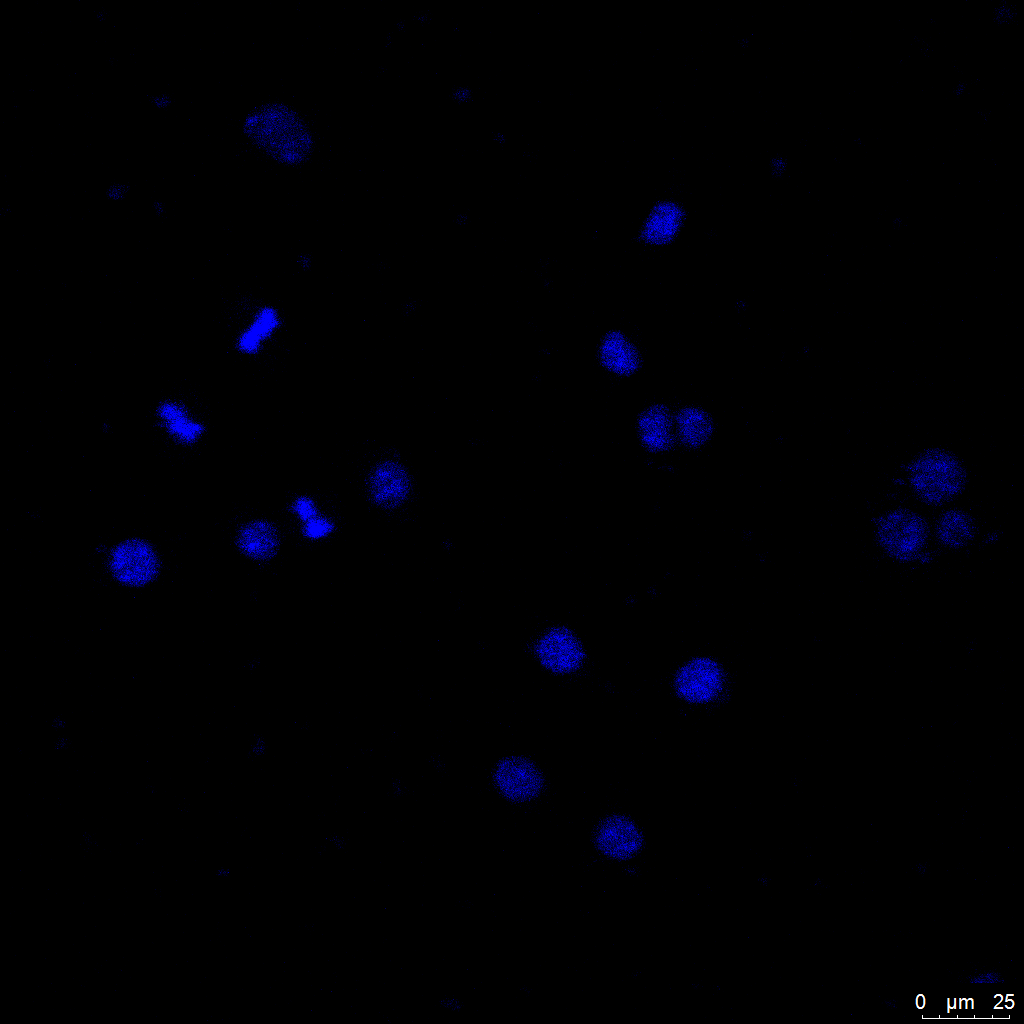

Supplement: Figure 2—figure supplement 6—source data 1. [file elife-70471-fig2-figsupp6-data1.zip › Figure 2-figure supplement 6-Source data 1/RAW264.7/RAW264.7 2_ch00.tif]

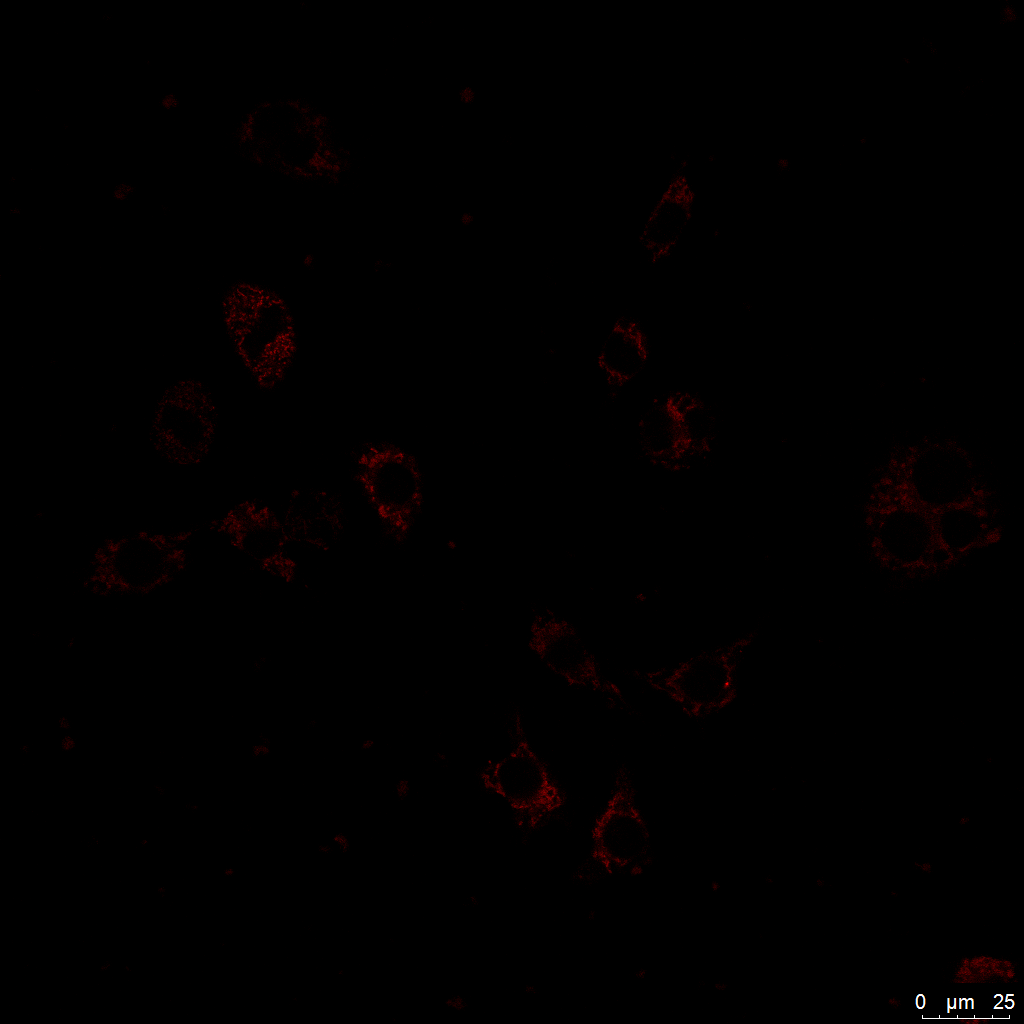

Supplement: Figure 2—figure supplement 6—source data 1. [file elife-70471-fig2-figsupp6-data1.zip › Figure 2-figure supplement 6-Source data 1/RAW264.7/RAW264.7 2_ch01.tif]

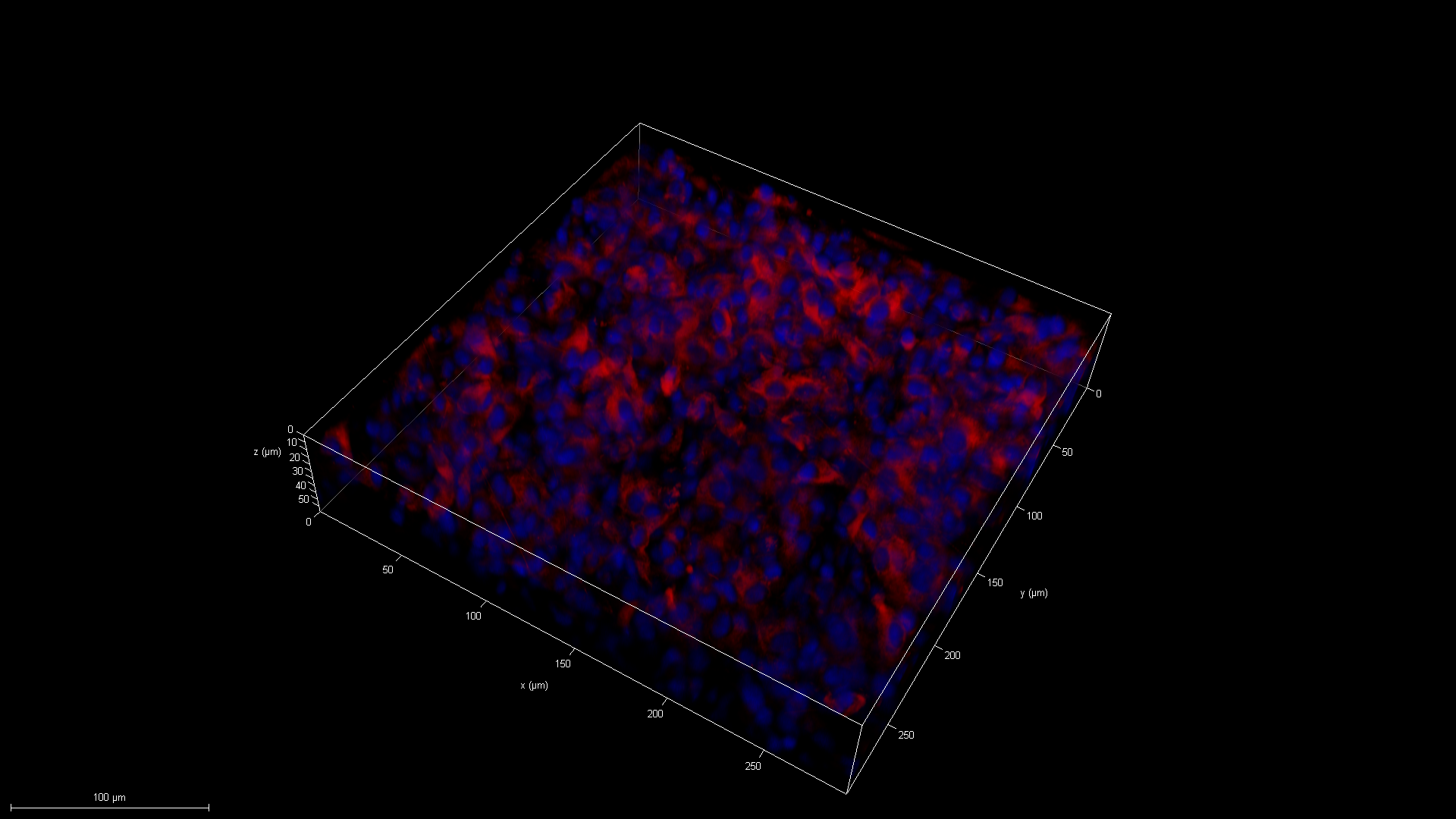

Supplement: Figure 2—figure supplement 7—source data 1. [file elife-70471-fig2-figsupp7-data1.zip › Figure 2-figure supplement 7-Source data 1/I-97H.tif]

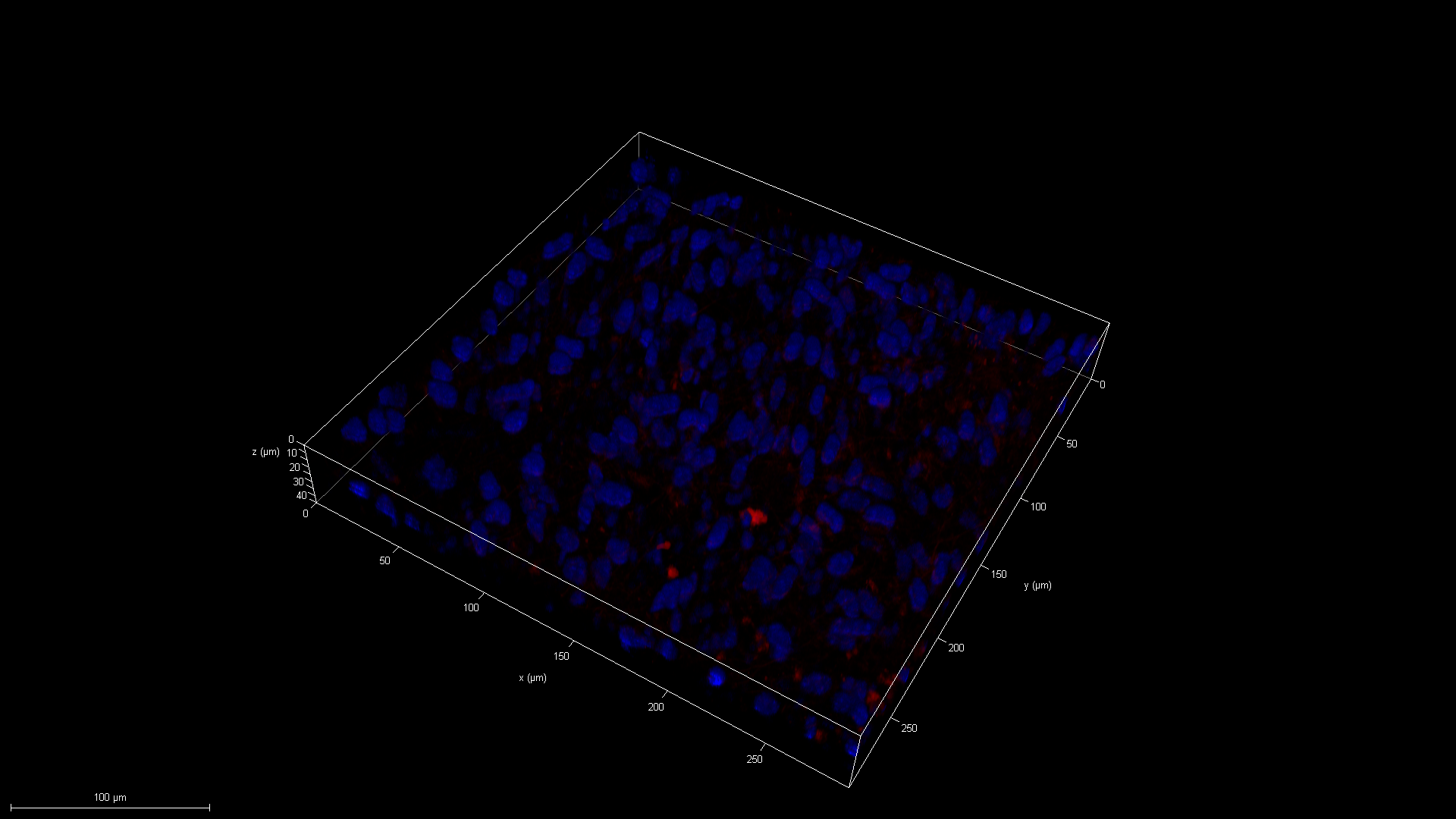

Supplement: Figure 2—figure supplement 7—source data 1. [file elife-70471-fig2-figsupp7-data1.zip › Figure 2-figure supplement 7-Source data 1/C-SPLEEN.tif]

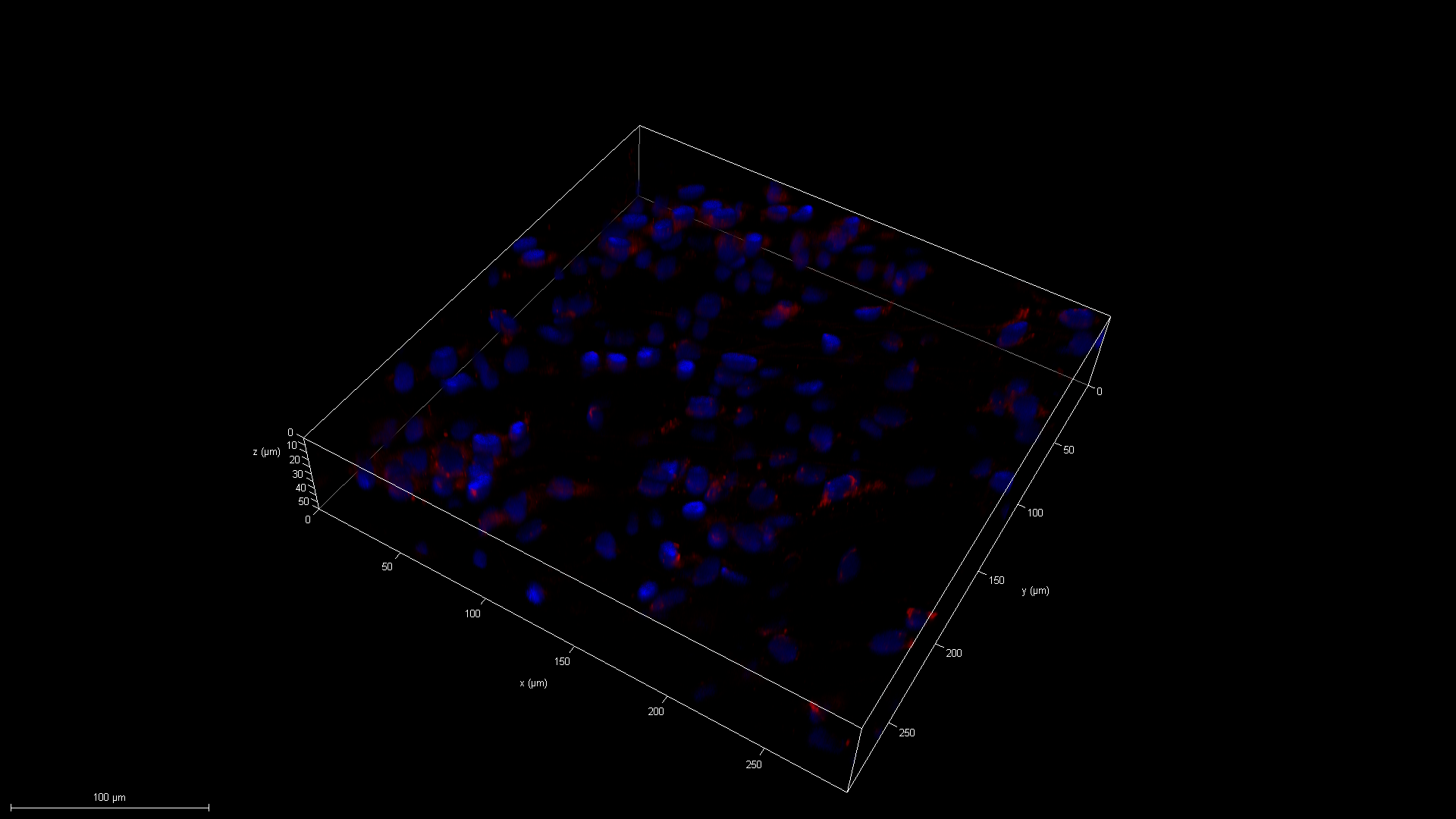

Supplement: Figure 2—figure supplement 7—source data 1. [file elife-70471-fig2-figsupp7-data1.zip › Figure 2-figure supplement 7-Source data 1/F-MUSCLE.tif]

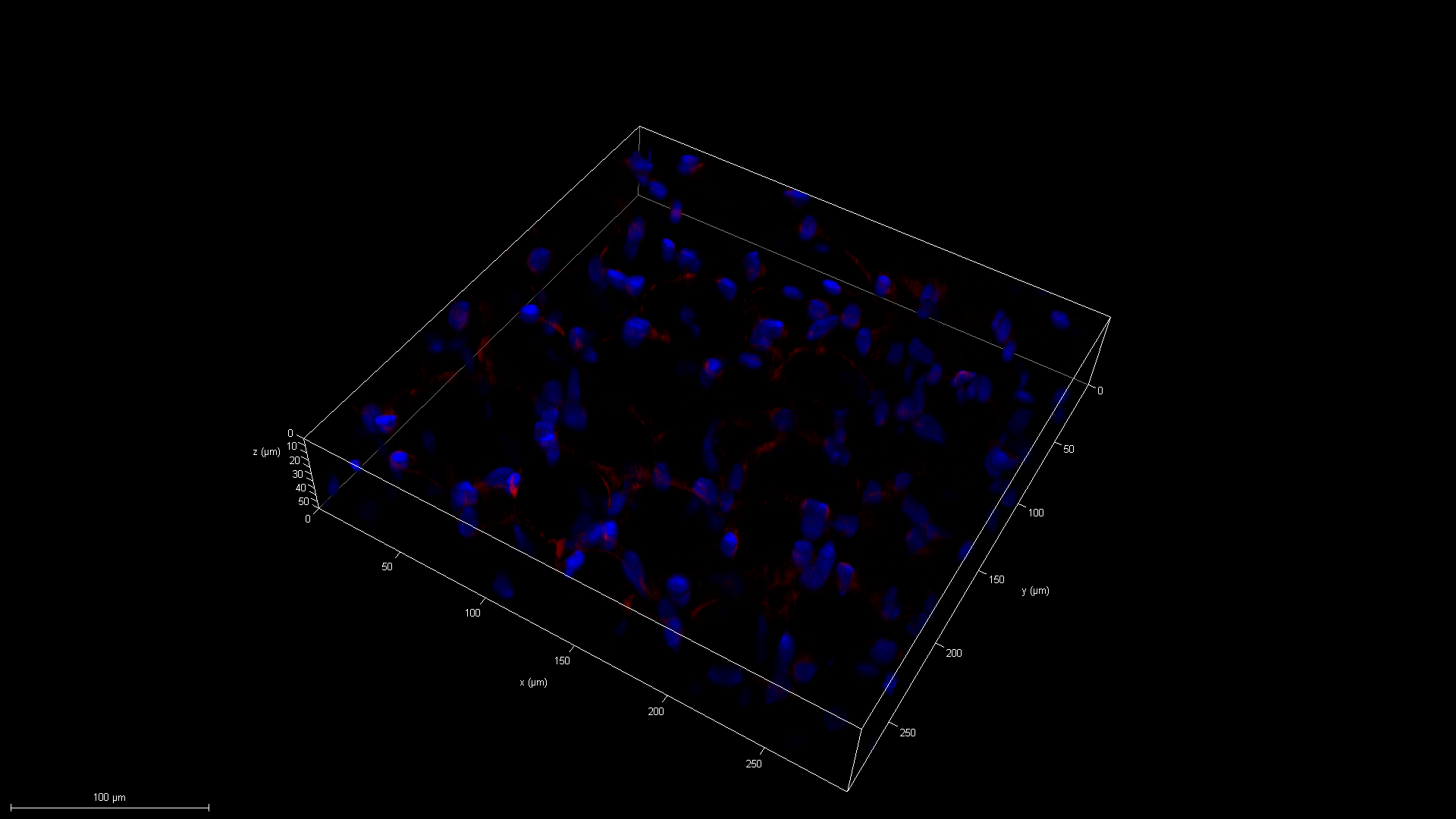

Supplement: Figure 2—figure supplement 7—source data 1. [file elife-70471-fig2-figsupp7-data1.zip › Figure 2-figure supplement 7-Source data 1/G-FAT.tif]

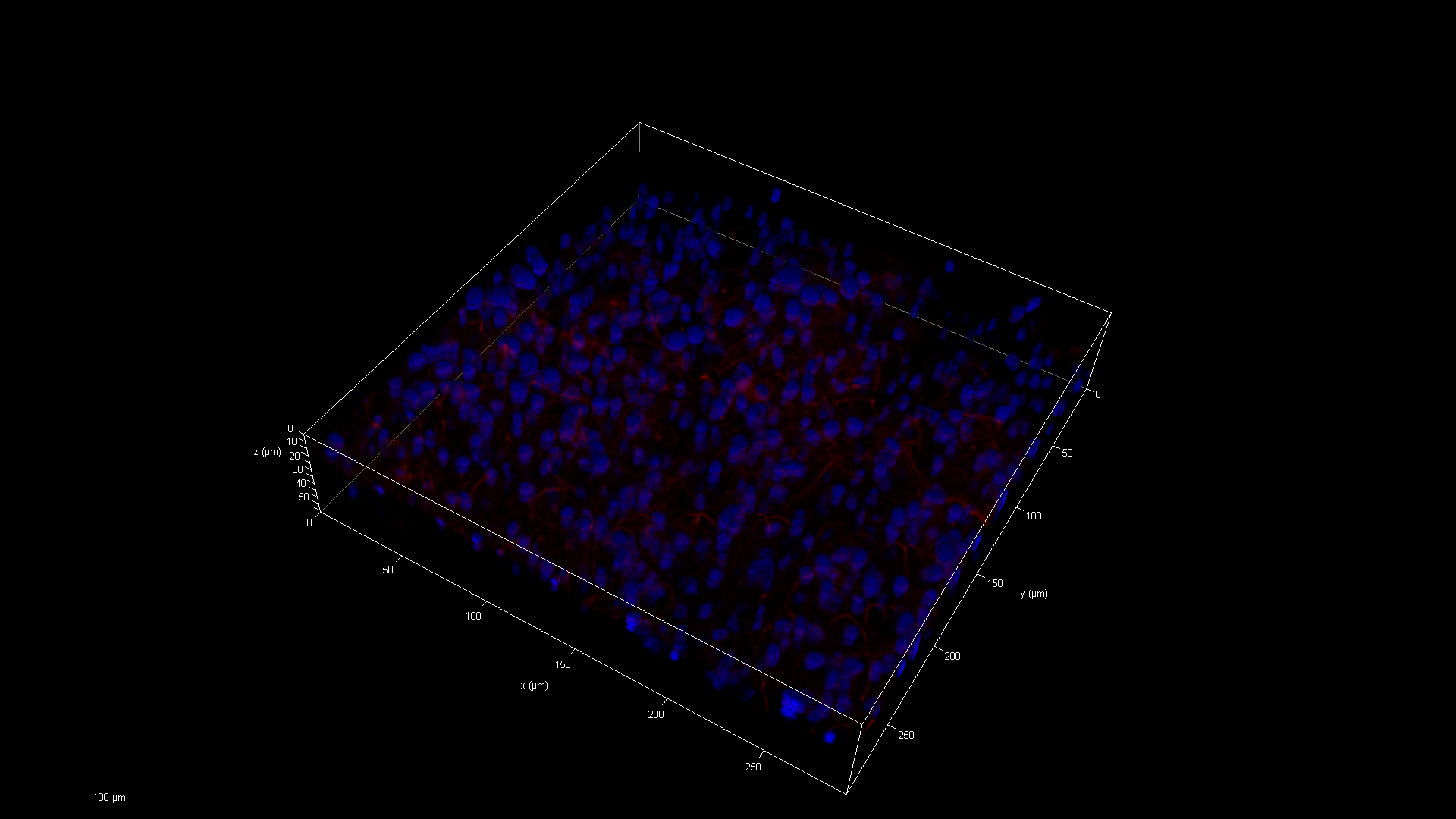

Supplement: Figure 2—figure supplement 7—source data 1. [file elife-70471-fig2-figsupp7-data1.zip › Figure 2-figure supplement 7-Source data 1/D-LUNG.tif]

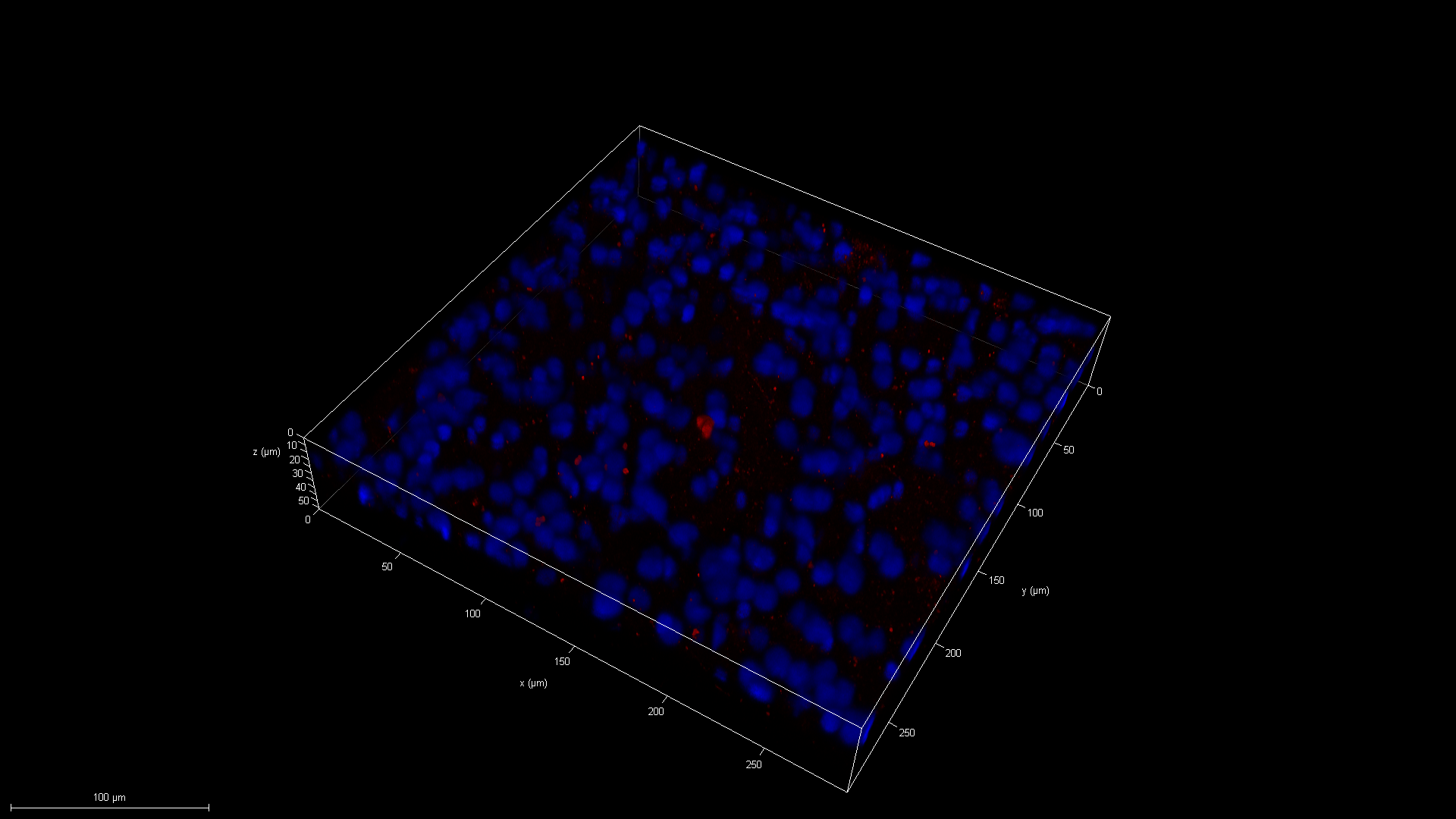

Supplement: Figure 2—figure supplement 7—source data 1. [file elife-70471-fig2-figsupp7-data1.zip › Figure 2-figure supplement 7-Source data 1/H-BRAIN.tif]

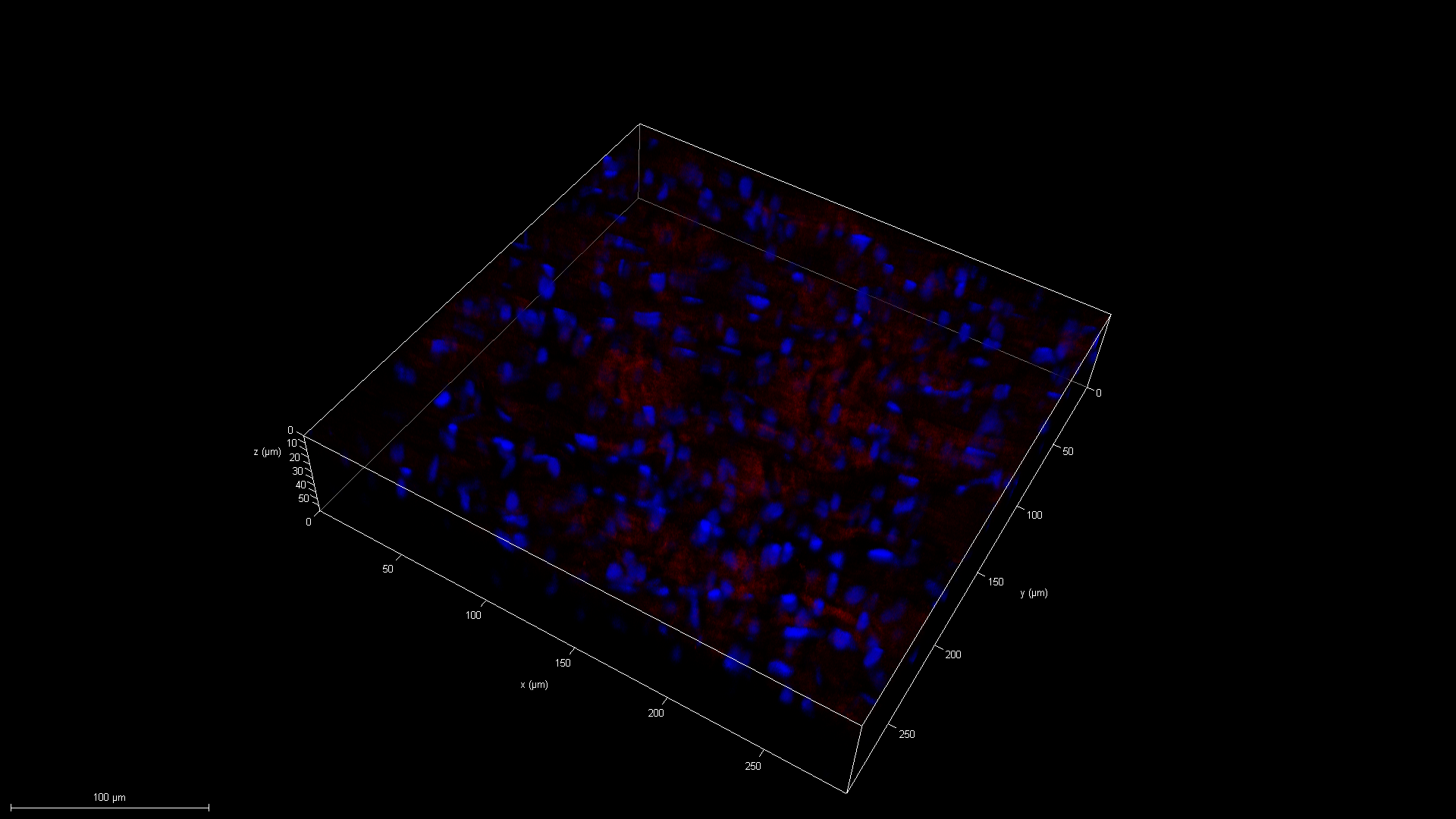

Supplement: Figure 2—figure supplement 7—source data 1. [file elife-70471-fig2-figsupp7-data1.zip › Figure 2-figure supplement 7-Source data 1/A-HEART.tif]

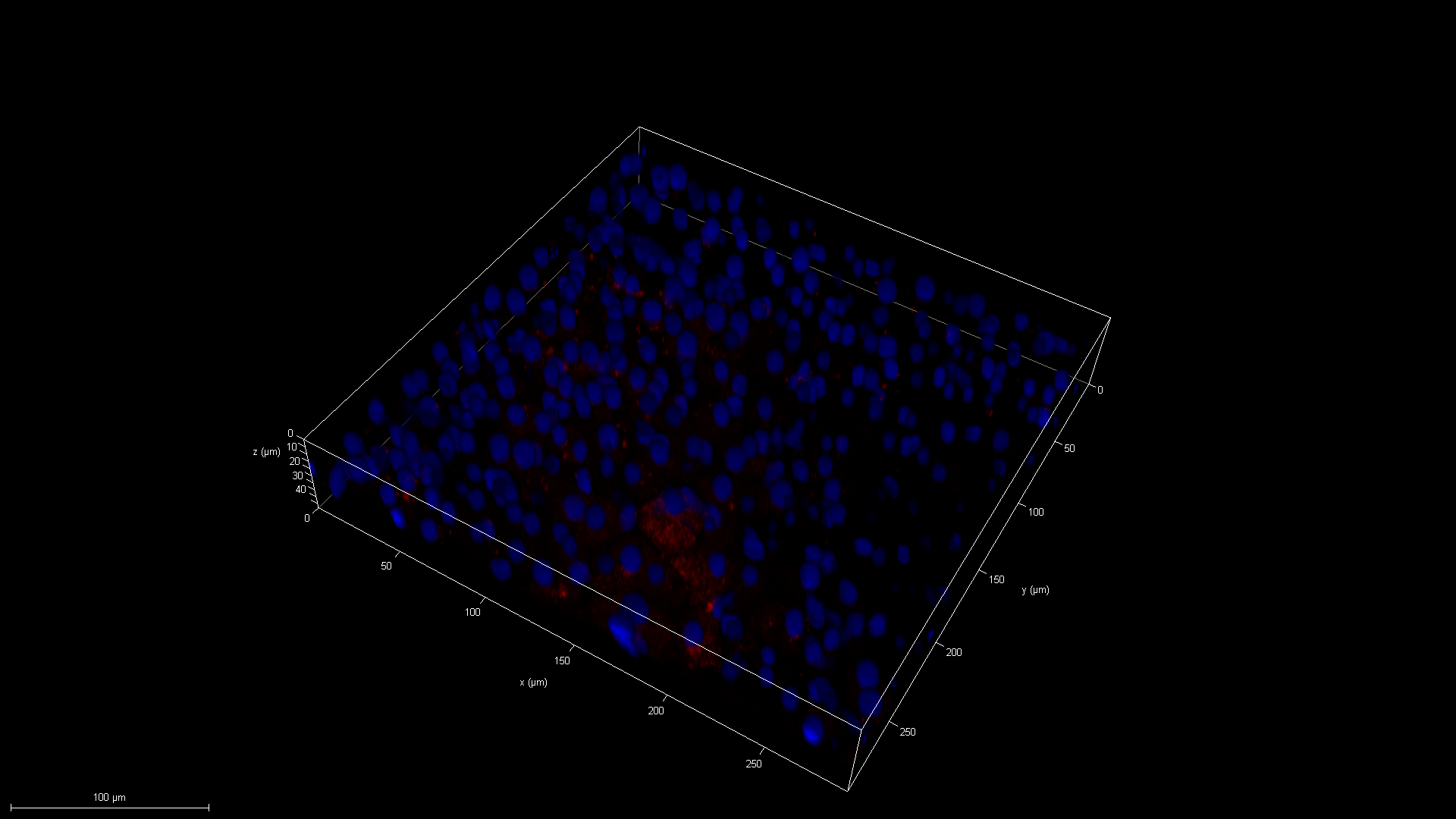

Supplement: Figure 2—figure supplement 7—source data 1. [file elife-70471-fig2-figsupp7-data1.zip › Figure 2-figure supplement 7-Source data 1/B-LIVER.tif]

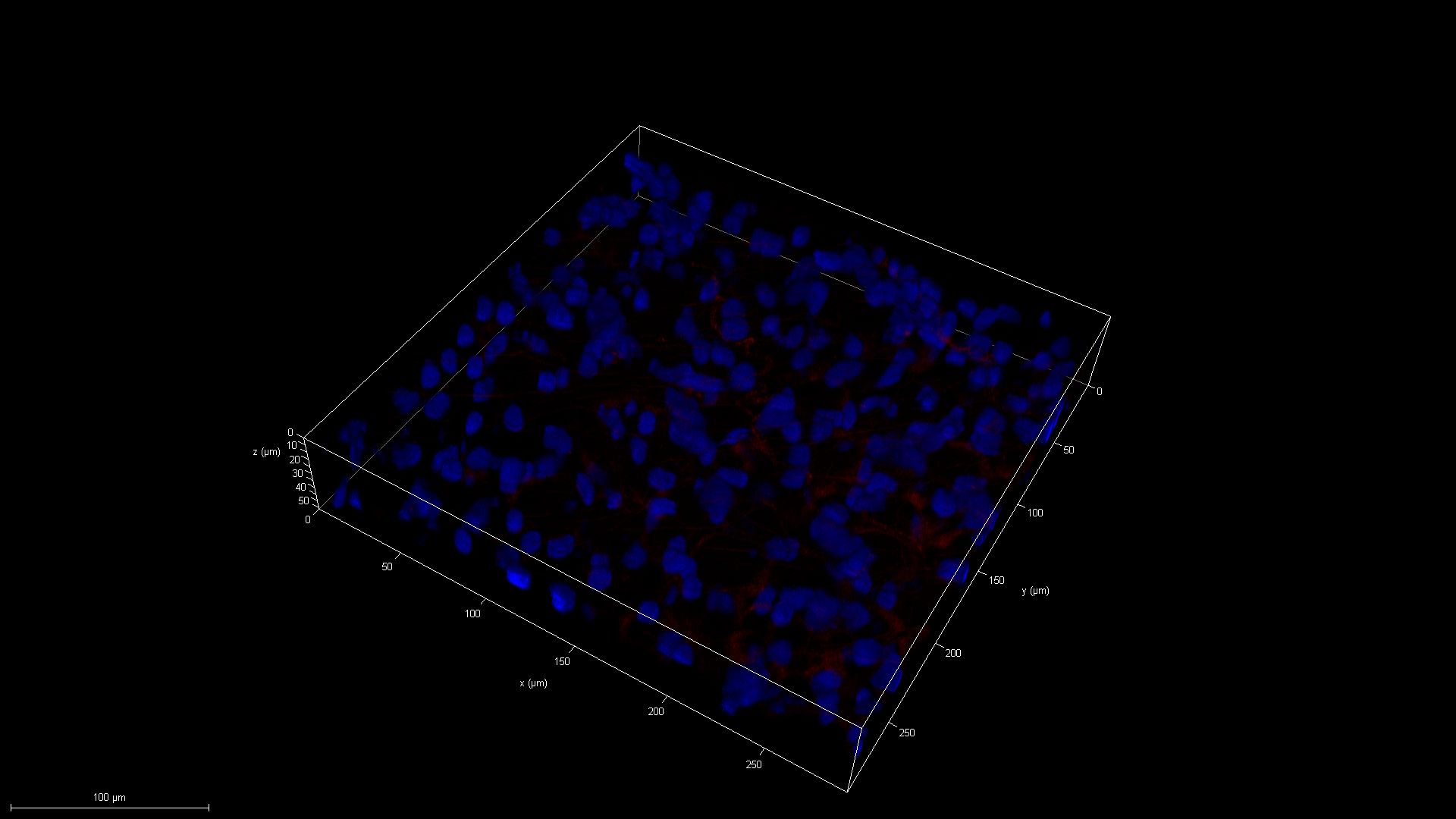

Supplement: Figure 2—figure supplement 7—source data 1. [file elife-70471-fig2-figsupp7-data1.zip › Figure 2-figure supplement 7-Source data 1/E-RENAL.tif]

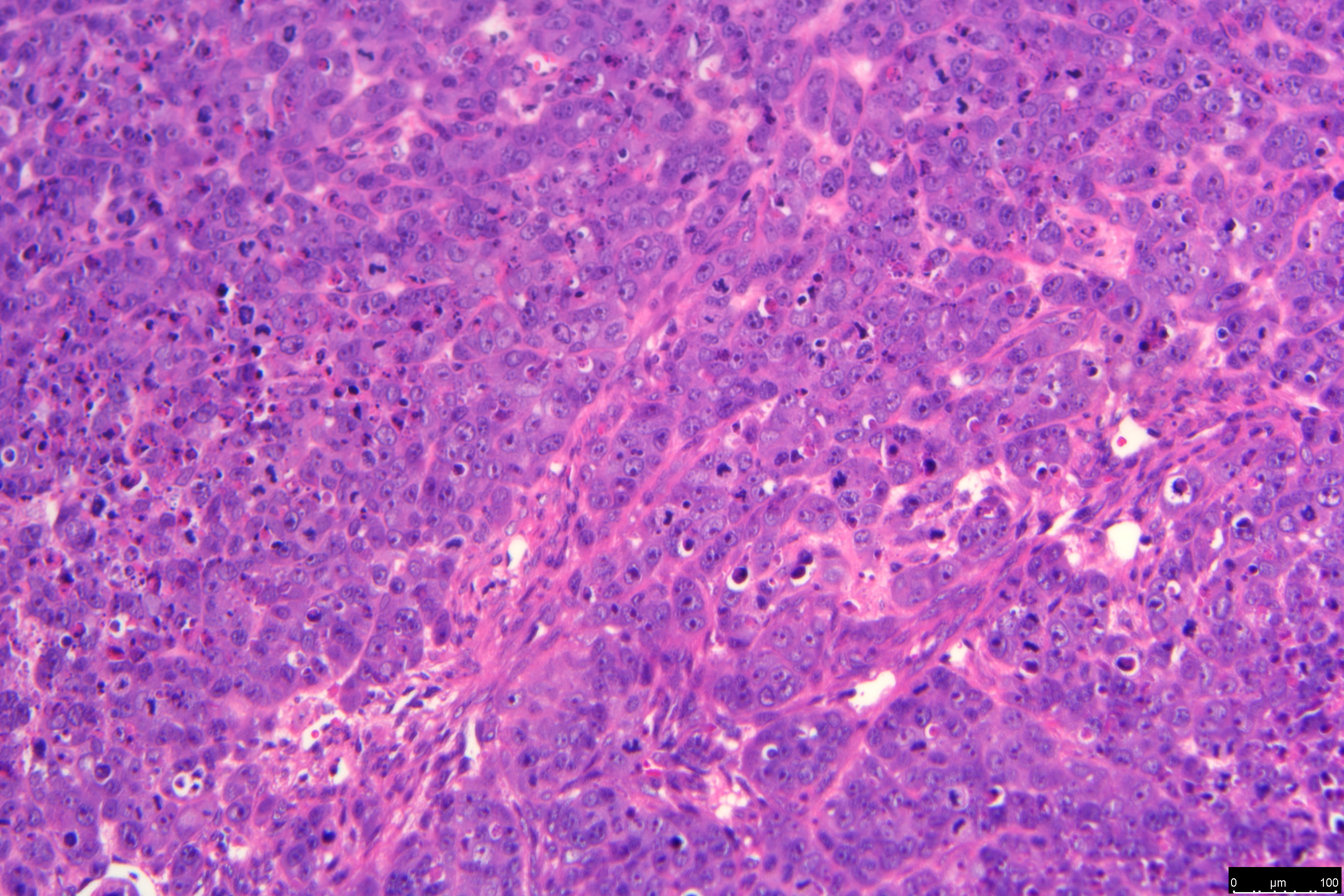

Supplement: Figure 3—source data 1. [file elife-70471-fig3-data1.zip › Figure 3-Source data/MHCC97H-bearing mice/Raw data-HE staining image in Figure 3C.tif]

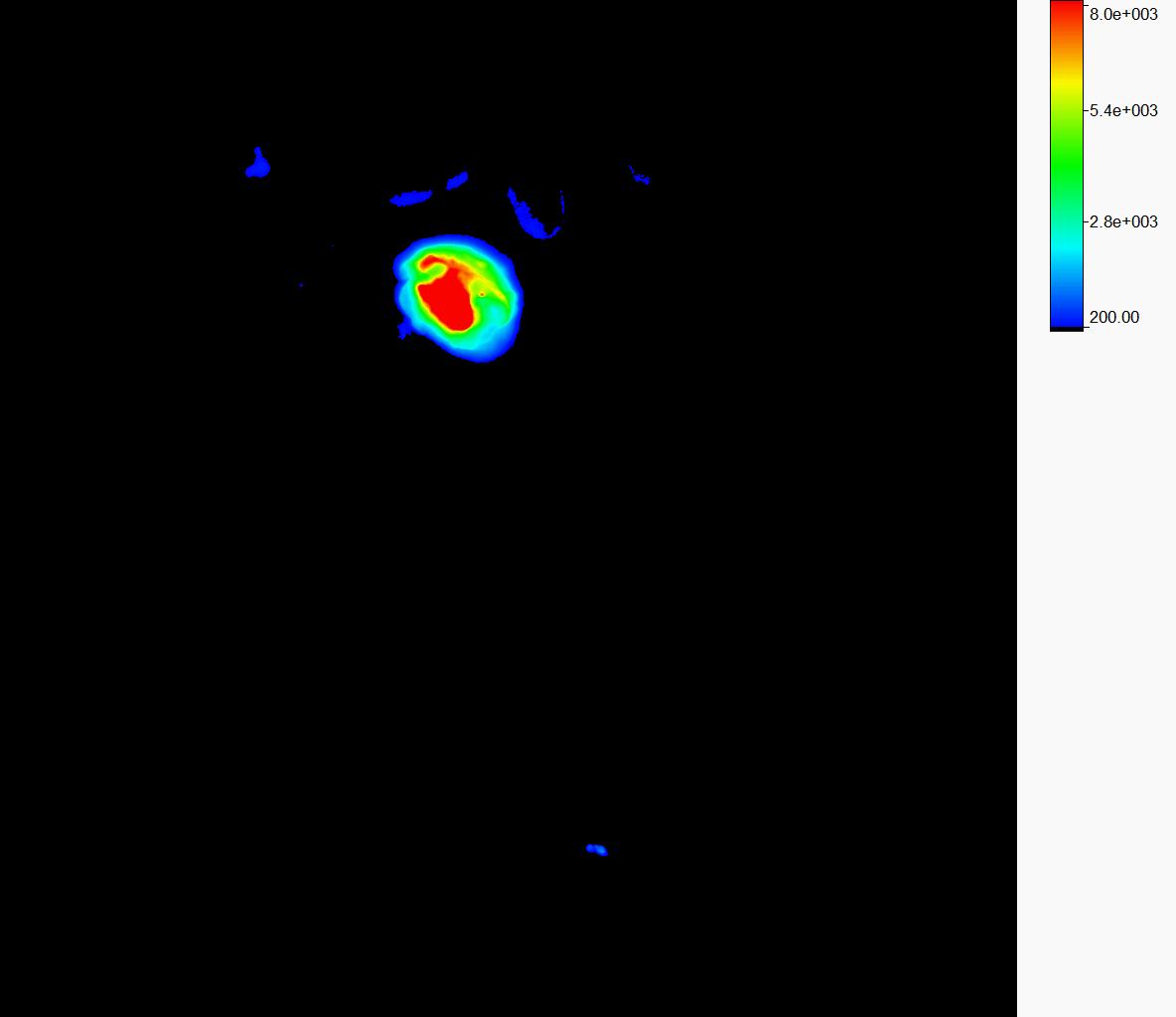

Supplement: Figure 3—source data 1. [file elife-70471-fig3-data1.zip › Figure 3-Source data/MHCC97H-bearing mice/Raw data-tissue viscosity detection image in Figure 3B.jpg]

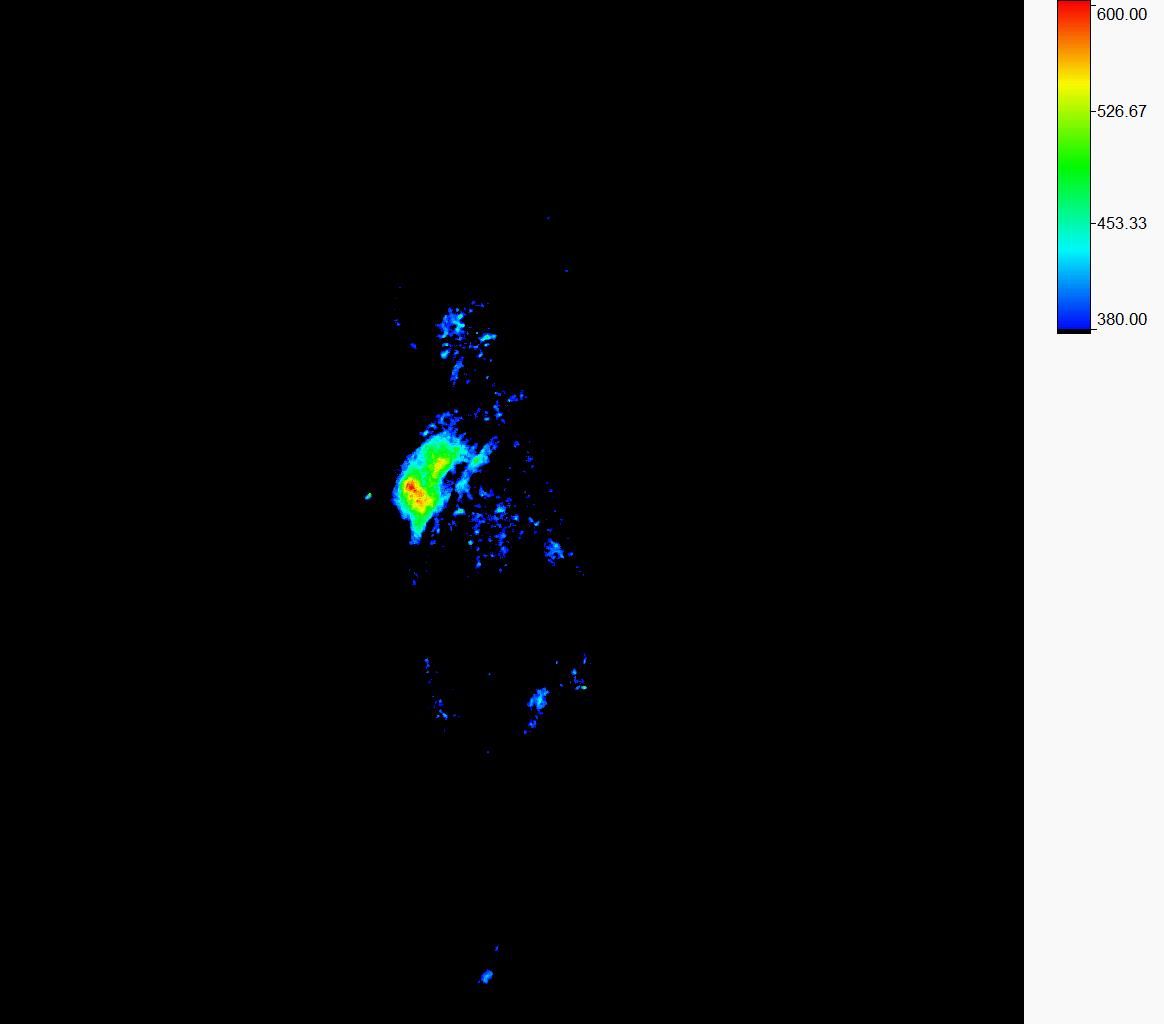

Supplement: Figure 3—source data 1. [file elife-70471-fig3-data1.zip › Figure 3-Source data/MHCC97H-bearing mice/Raw data-in vivo nitroreductase detection image in Figure 3A.jpg]

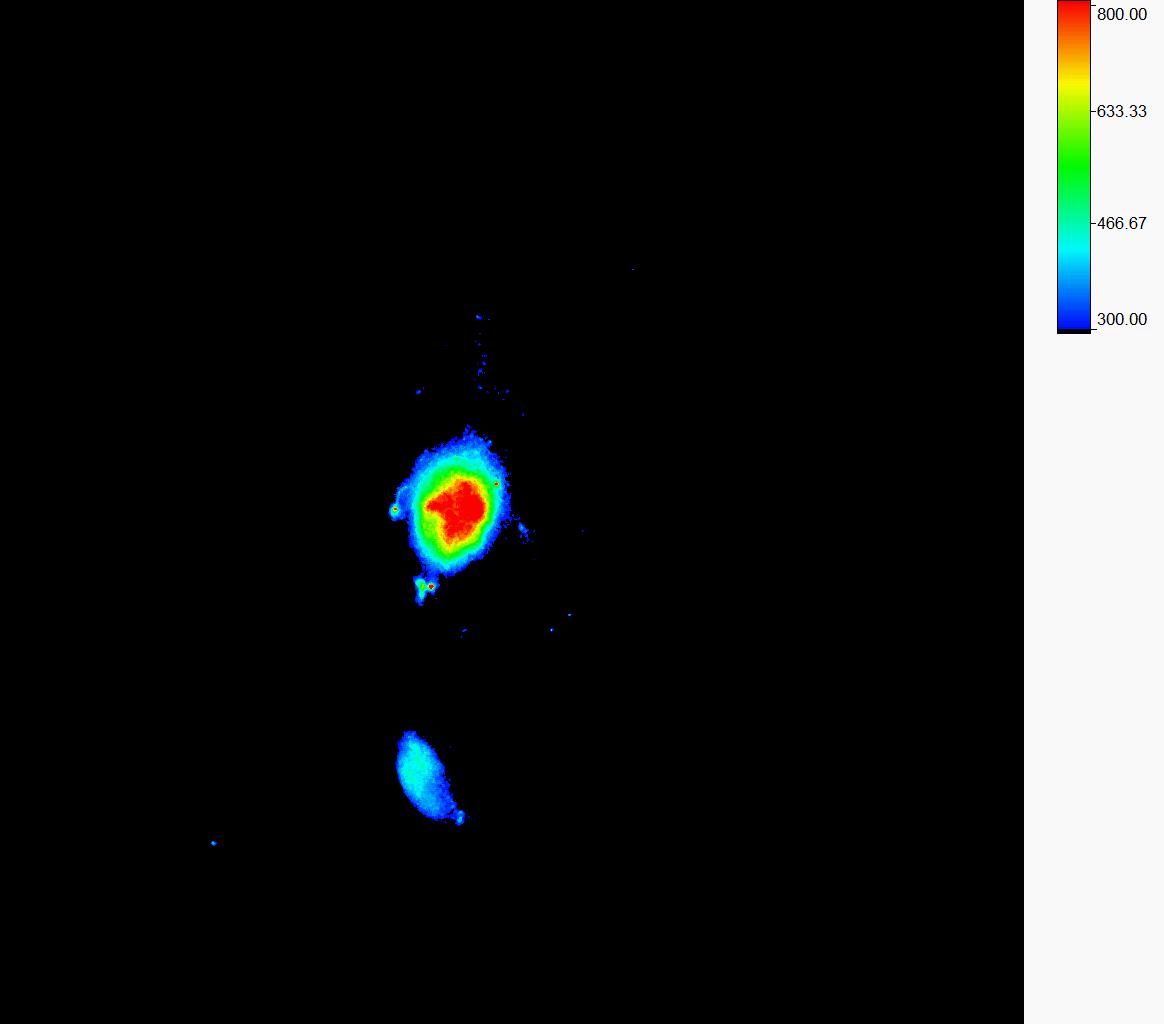

Supplement: Figure 3—source data 1. [file elife-70471-fig3-data1.zip › Figure 3-Source data/MHCC97H-bearing mice/Raw data-in vivo viscosity detection image in Figure 3A.jpg]

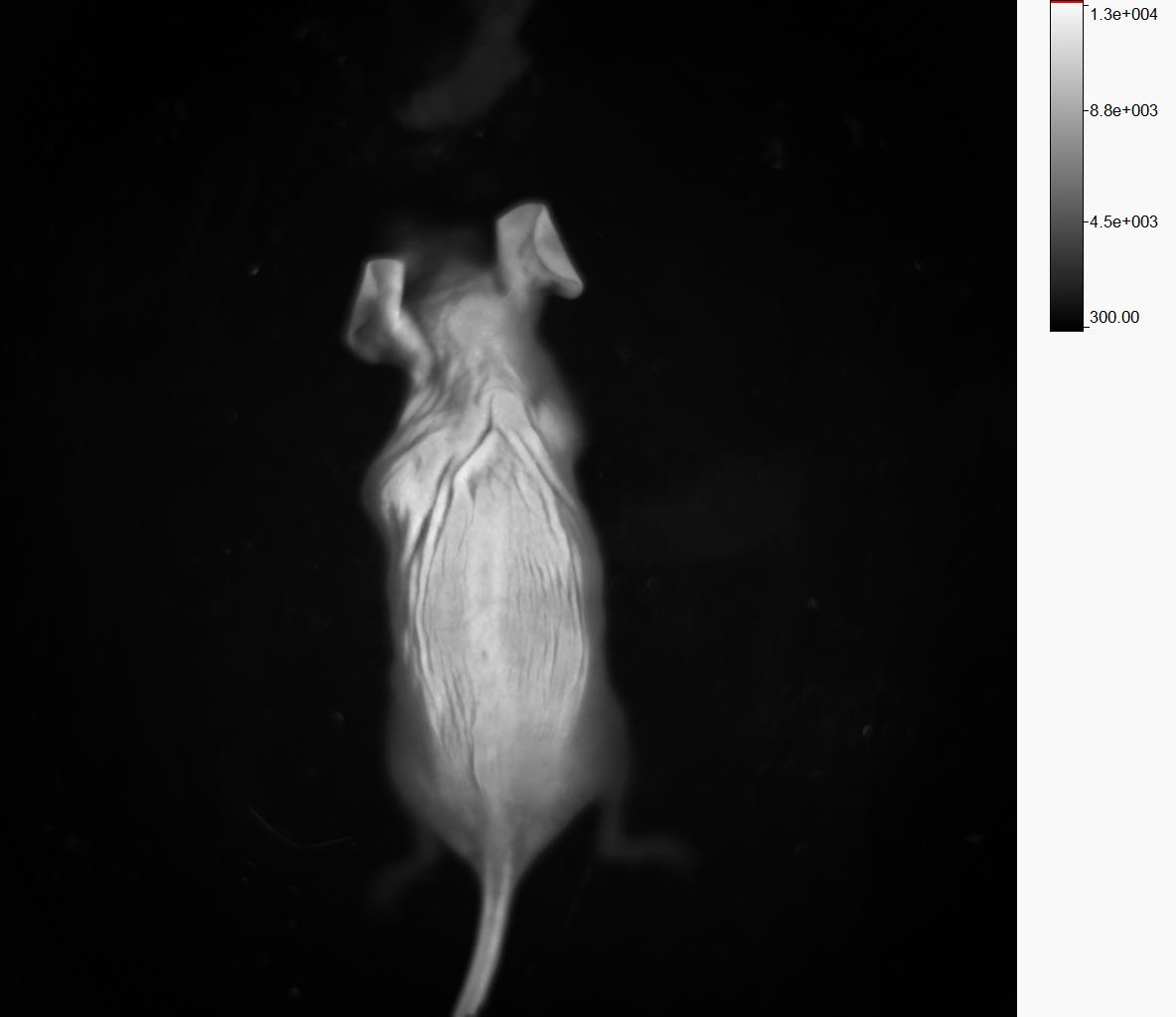

Supplement: Figure 3—source data 1. [file elife-70471-fig3-data1.zip › Figure 3-Source data/MHCC97H-bearing mice/Raw data-Reflectance image in Figure 3A.jpg]

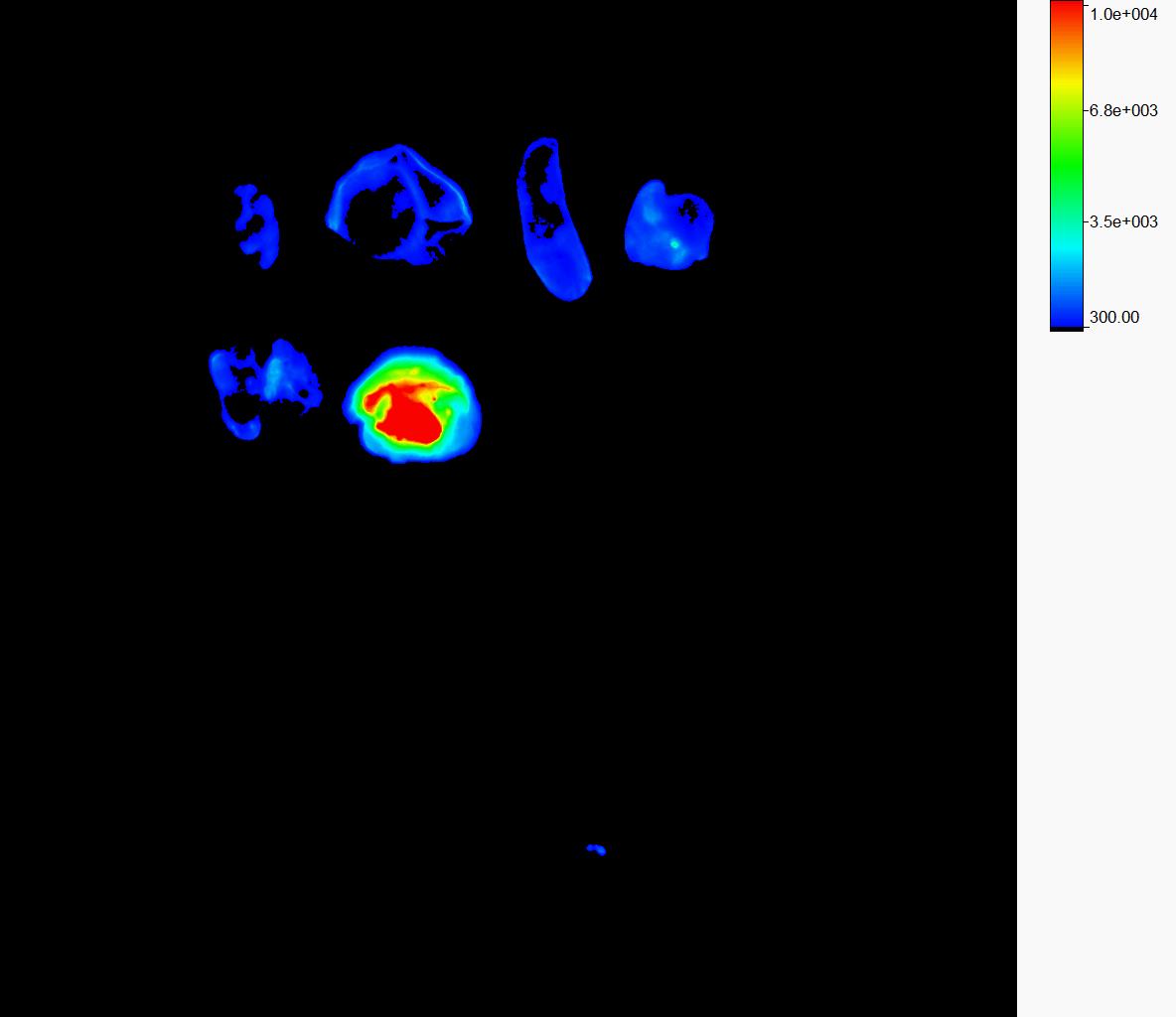

Supplement: Figure 3—source data 1. [file elife-70471-fig3-data1.zip › Figure 3-Source data/MHCC97H-bearing mice/Raw data-tissue nitroreductase detection image in Figure 3B.jpg]

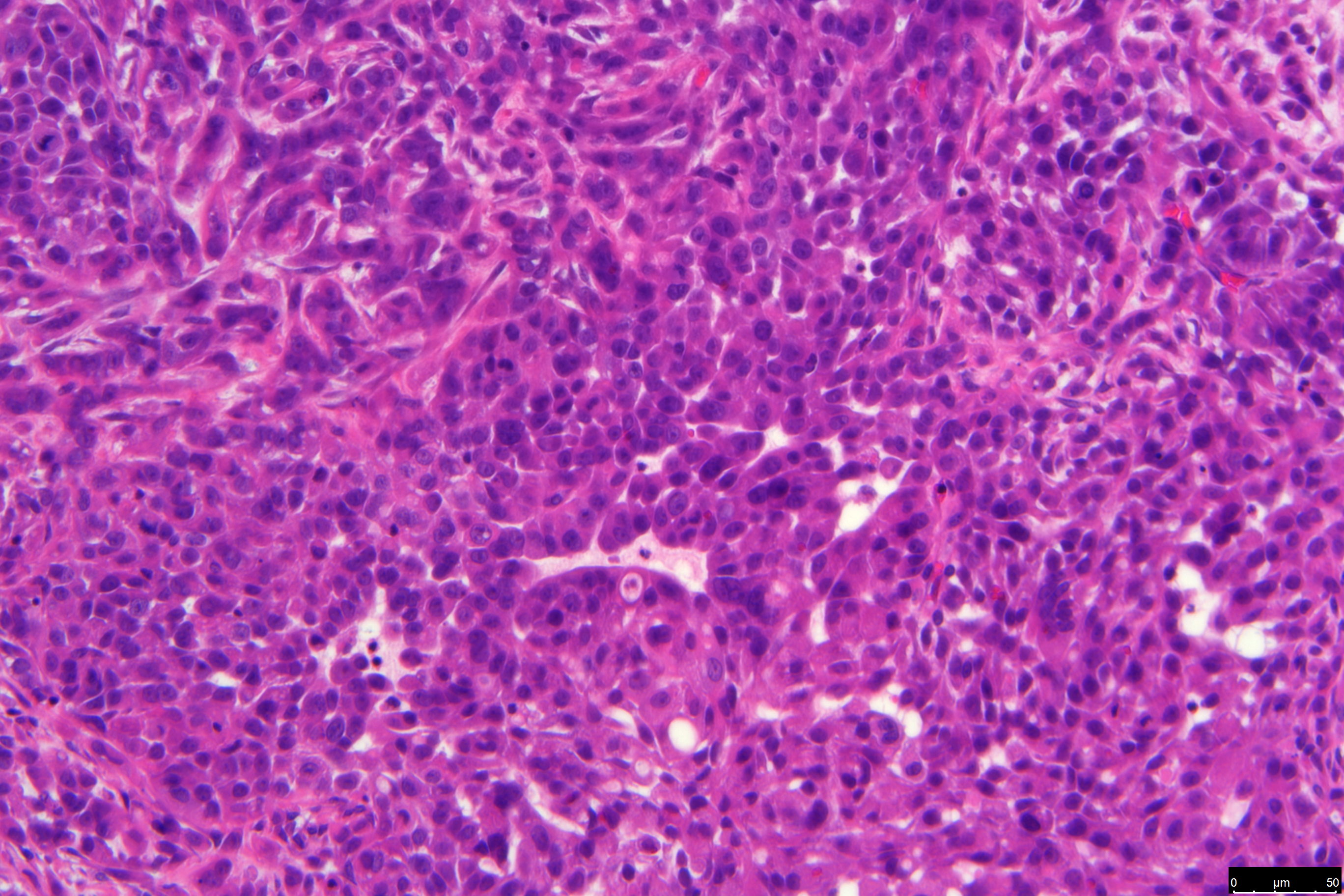

Supplement: Figure 3—source data 1. [file elife-70471-fig3-data1.zip › Figure 3-Source data/A549-bearing mice/Raw data-HE staining image in Figure 3C.tif]

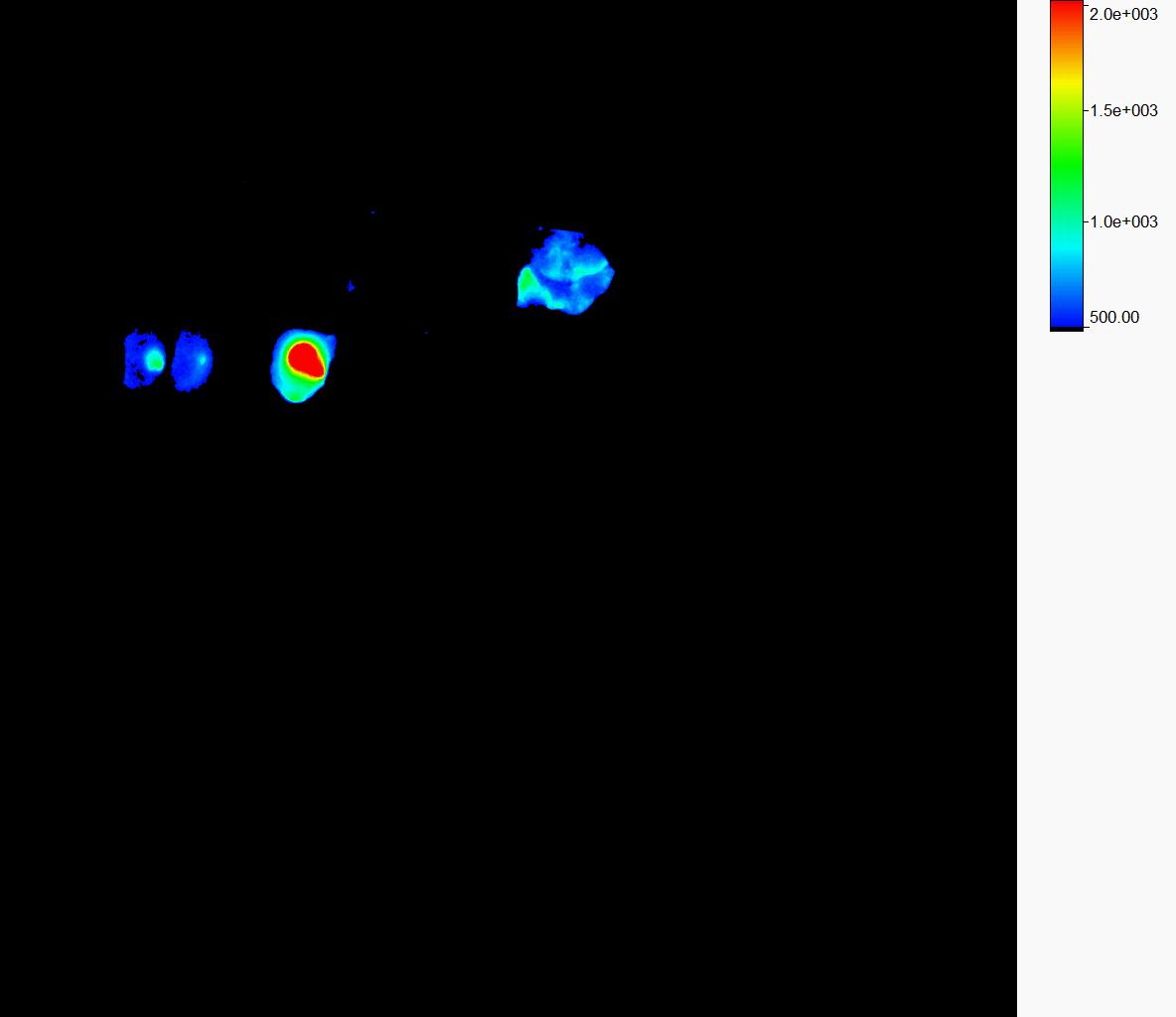

Supplement: Figure 3—source data 1. [file elife-70471-fig3-data1.zip › Figure 3-Source data/A549-bearing mice/Raw data-tissue viscosity detection image in Figure 3B.jpg]

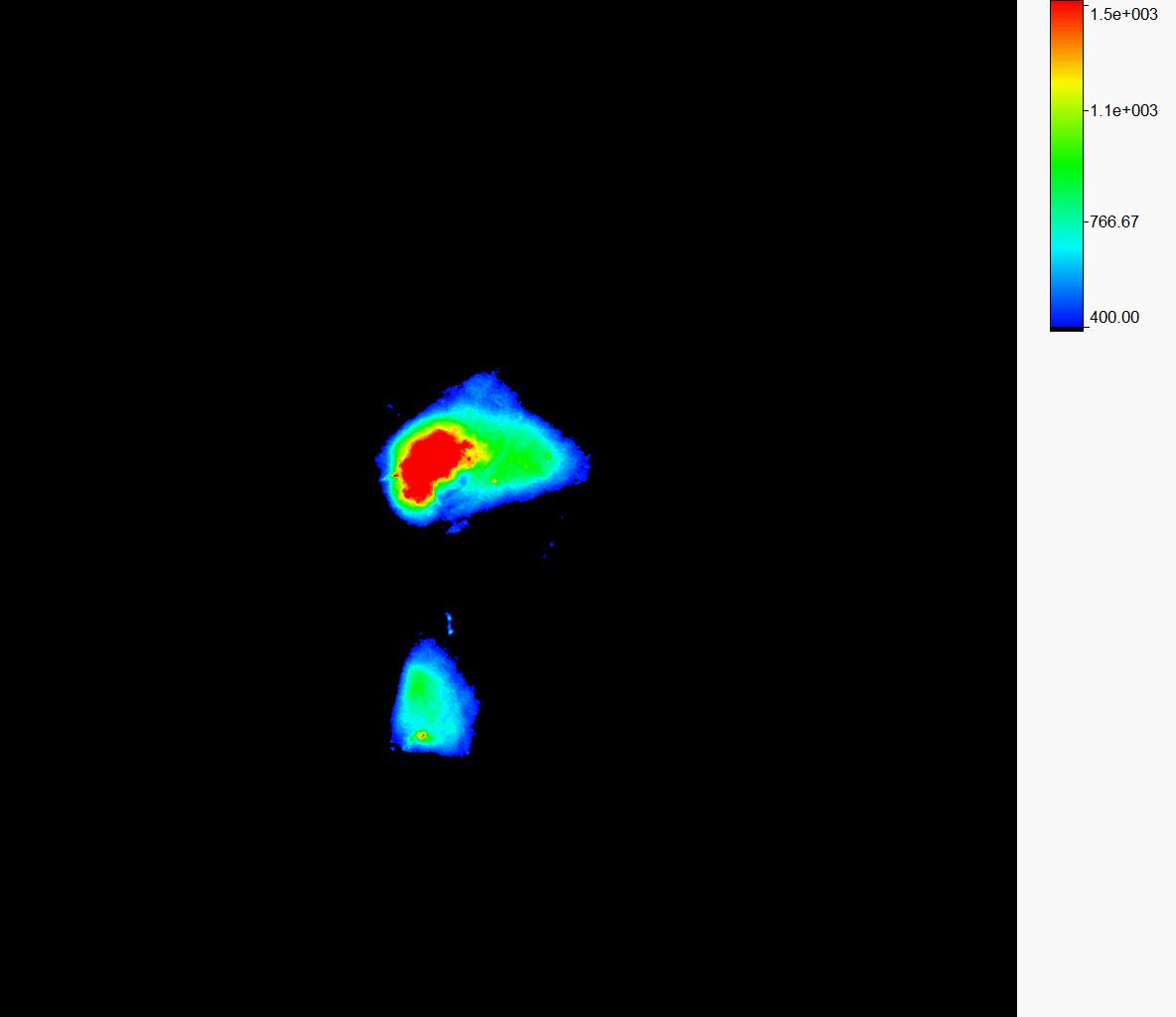

Supplement: Figure 3—source data 1. [file elife-70471-fig3-data1.zip › Figure 3-Source data/A549-bearing mice/Raw data-in vivo nitroreductase detection image in Figure 3A.jpg]

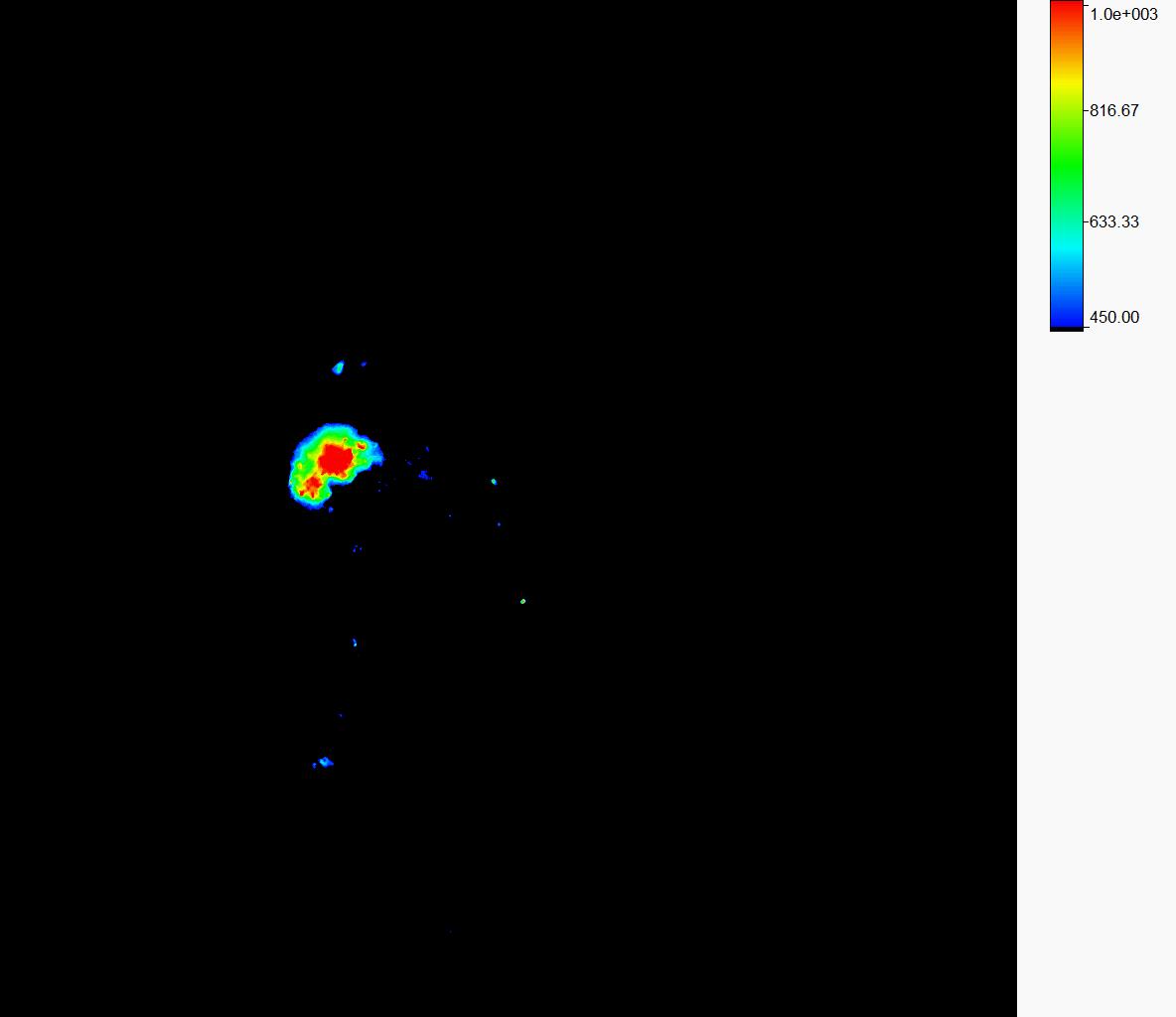

Supplement: Figure 3—source data 1. [file elife-70471-fig3-data1.zip › Figure 3-Source data/A549-bearing mice/Raw data-in vivo viscosity detection image in Figure 3A.jpg]

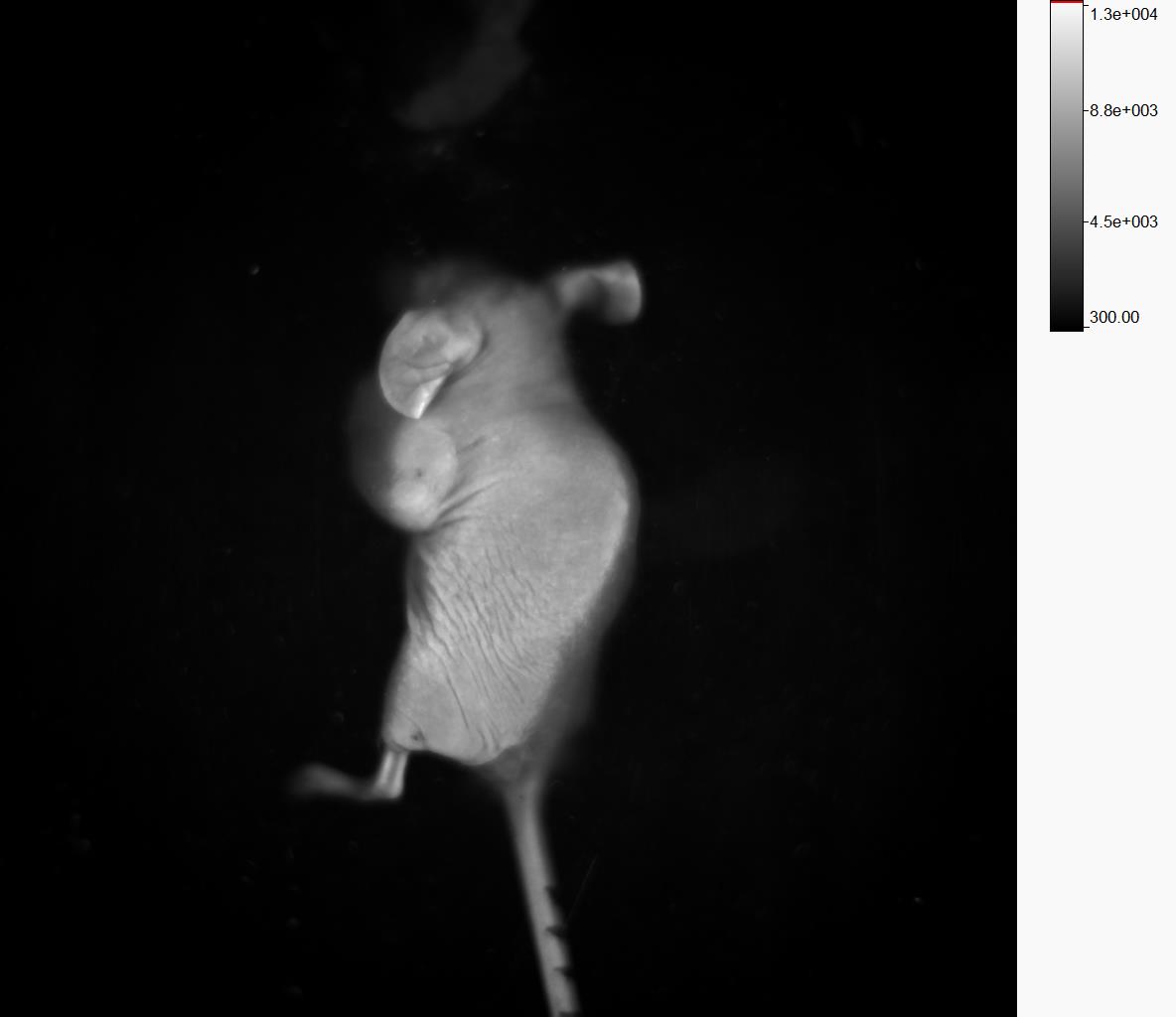

Supplement: Figure 3—source data 1. [file elife-70471-fig3-data1.zip › Figure 3-Source data/A549-bearing mice/Raw data-Reflectance image in Figure 3A.jpg]

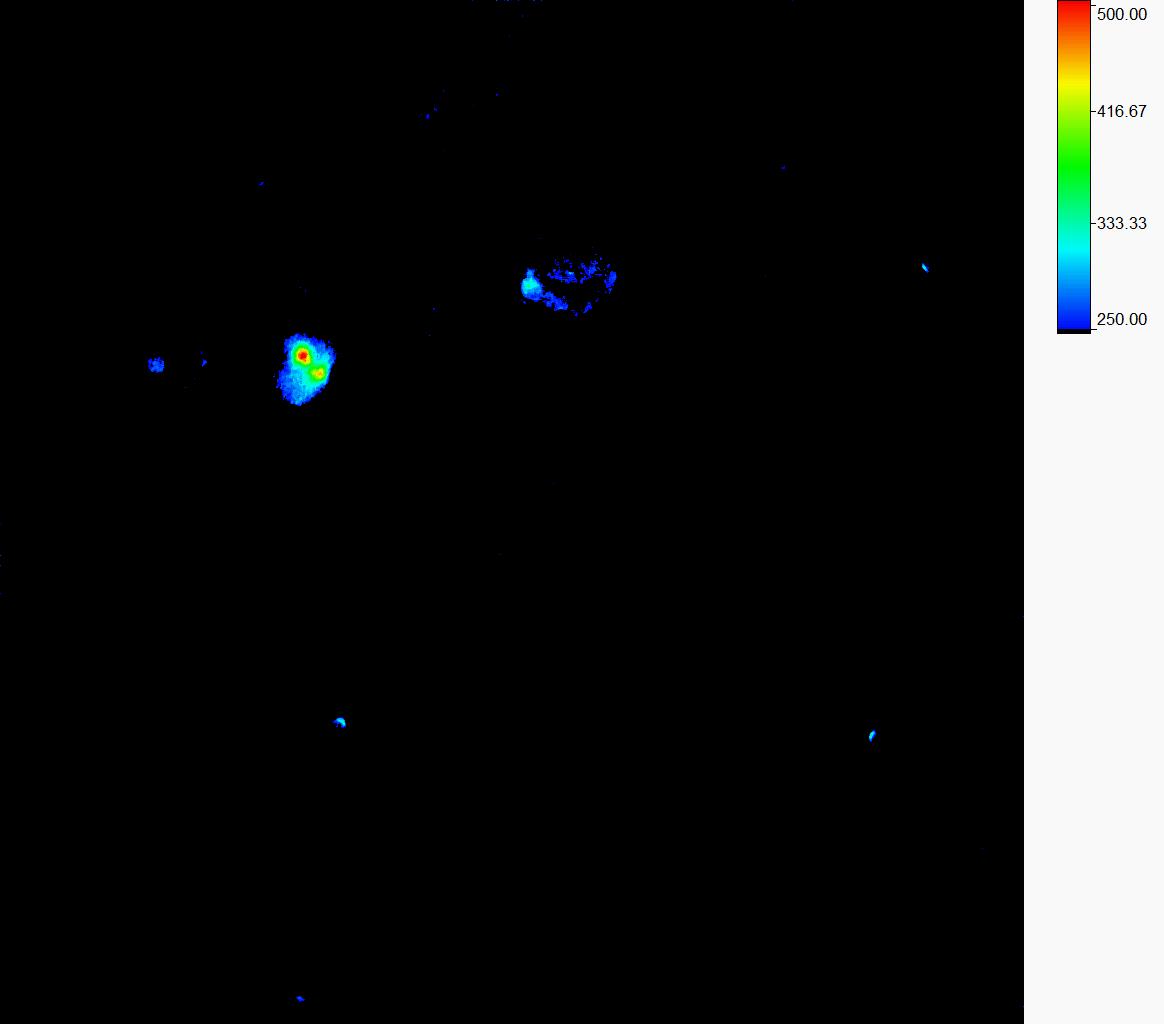

Supplement: Figure 3—source data 1. [file elife-70471-fig3-data1.zip › Figure 3-Source data/A549-bearing mice/Raw data-tissue nitroreductase detection image in Figure 3B.jpg]

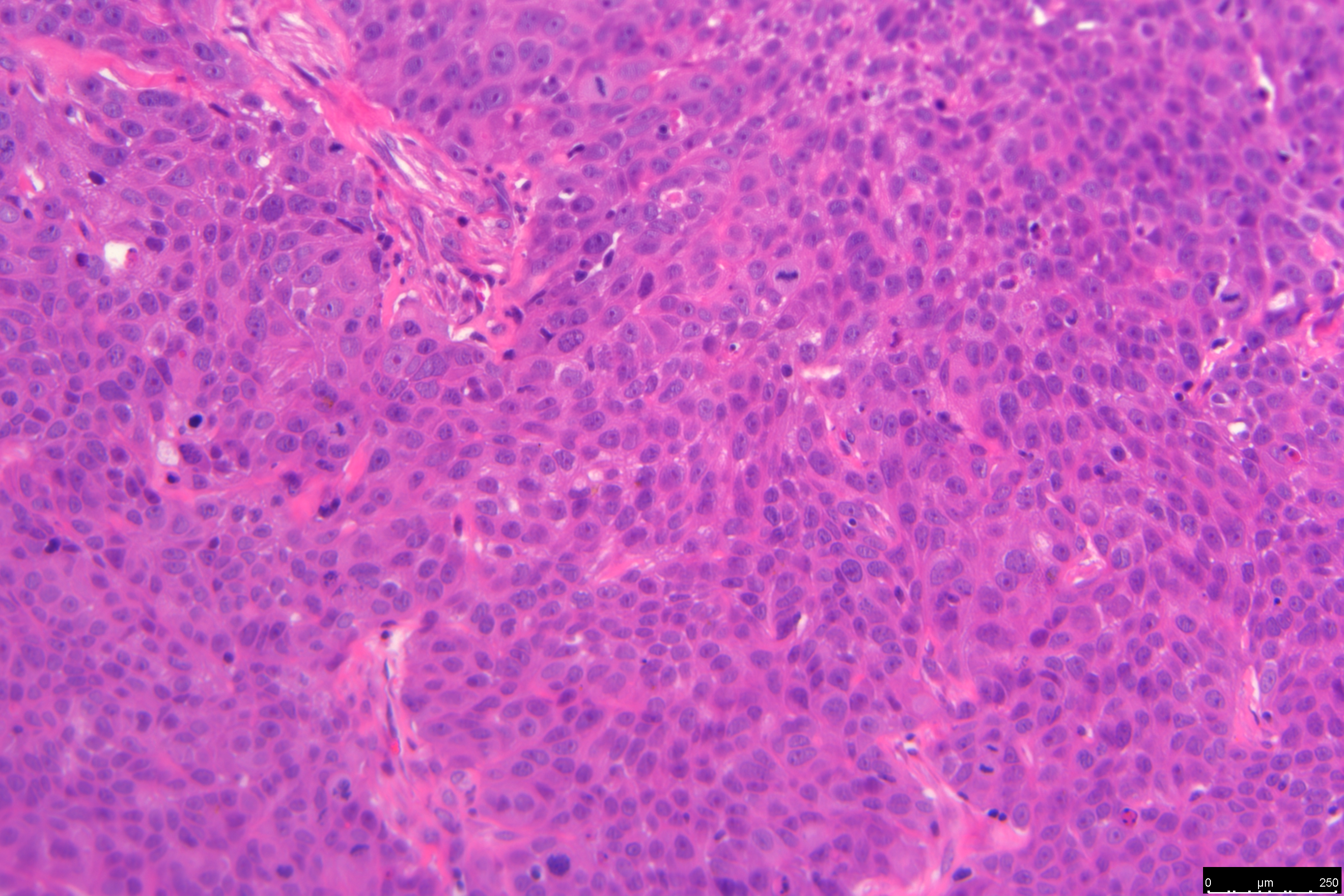

Supplement: Figure 3—source data 1. [file elife-70471-fig3-data1.zip › Figure 3-Source data/FaDu-bearing mice/Raw data-HE staining image in Figure 3C.tif]

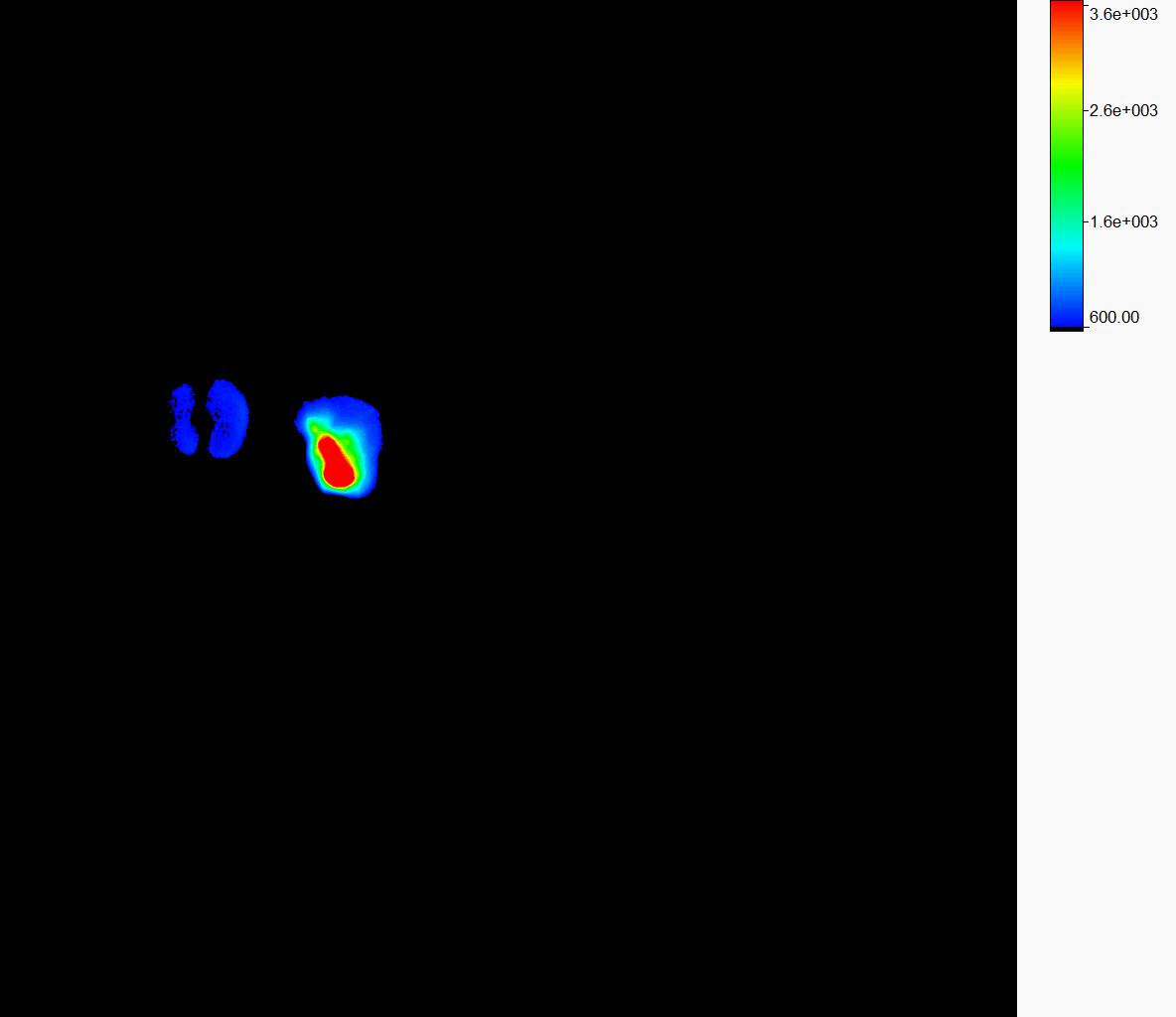

Supplement: Figure 3—source data 1. [file elife-70471-fig3-data1.zip › Figure 3-Source data/FaDu-bearing mice/Raw data-tissue viscosity detection image in Figure 3B.jpg]

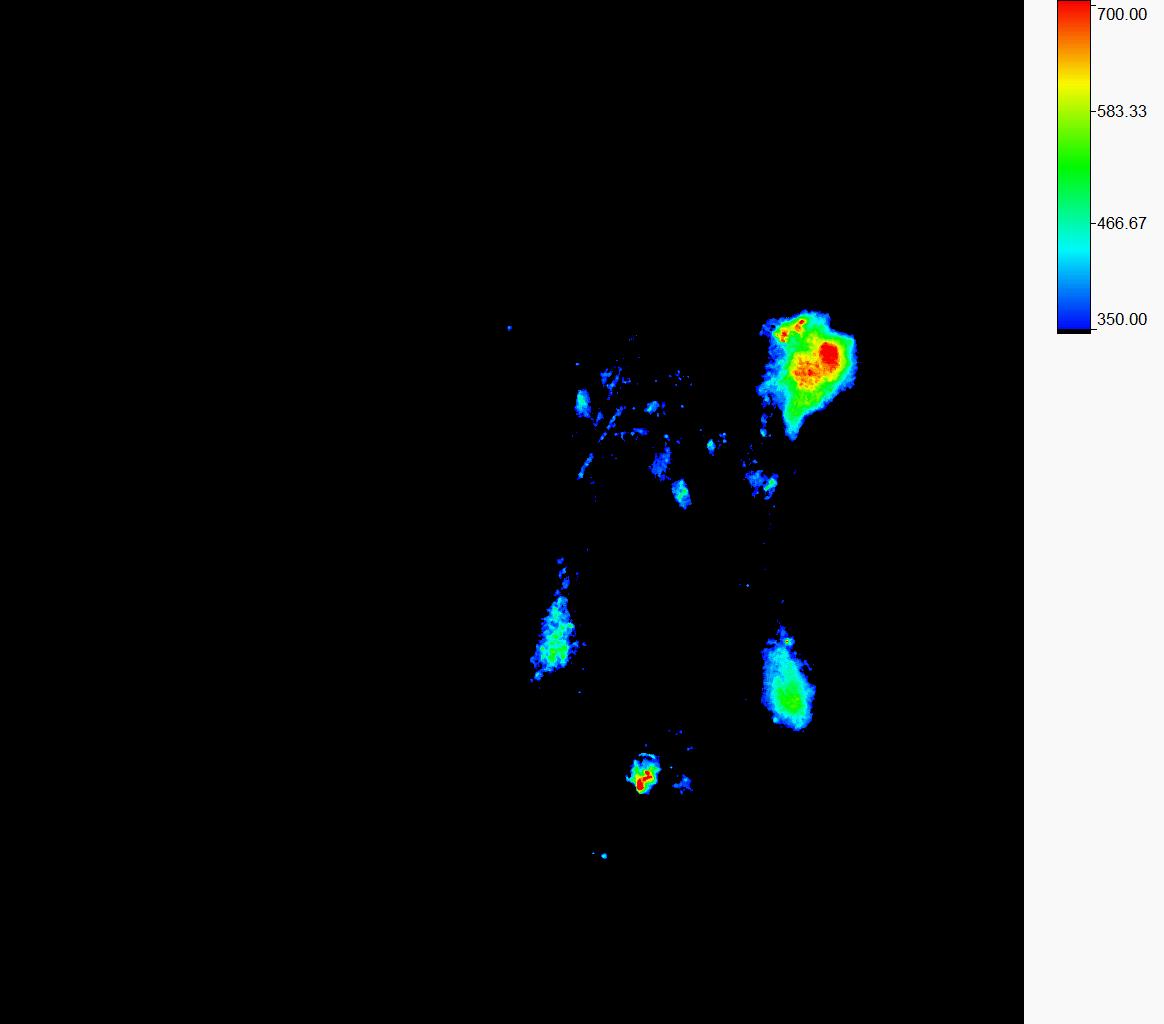

Supplement: Figure 3—source data 1. [file elife-70471-fig3-data1.zip › Figure 3-Source data/FaDu-bearing mice/Raw data-in vivo nitroreductase detection image in Figure 3A.jpg]

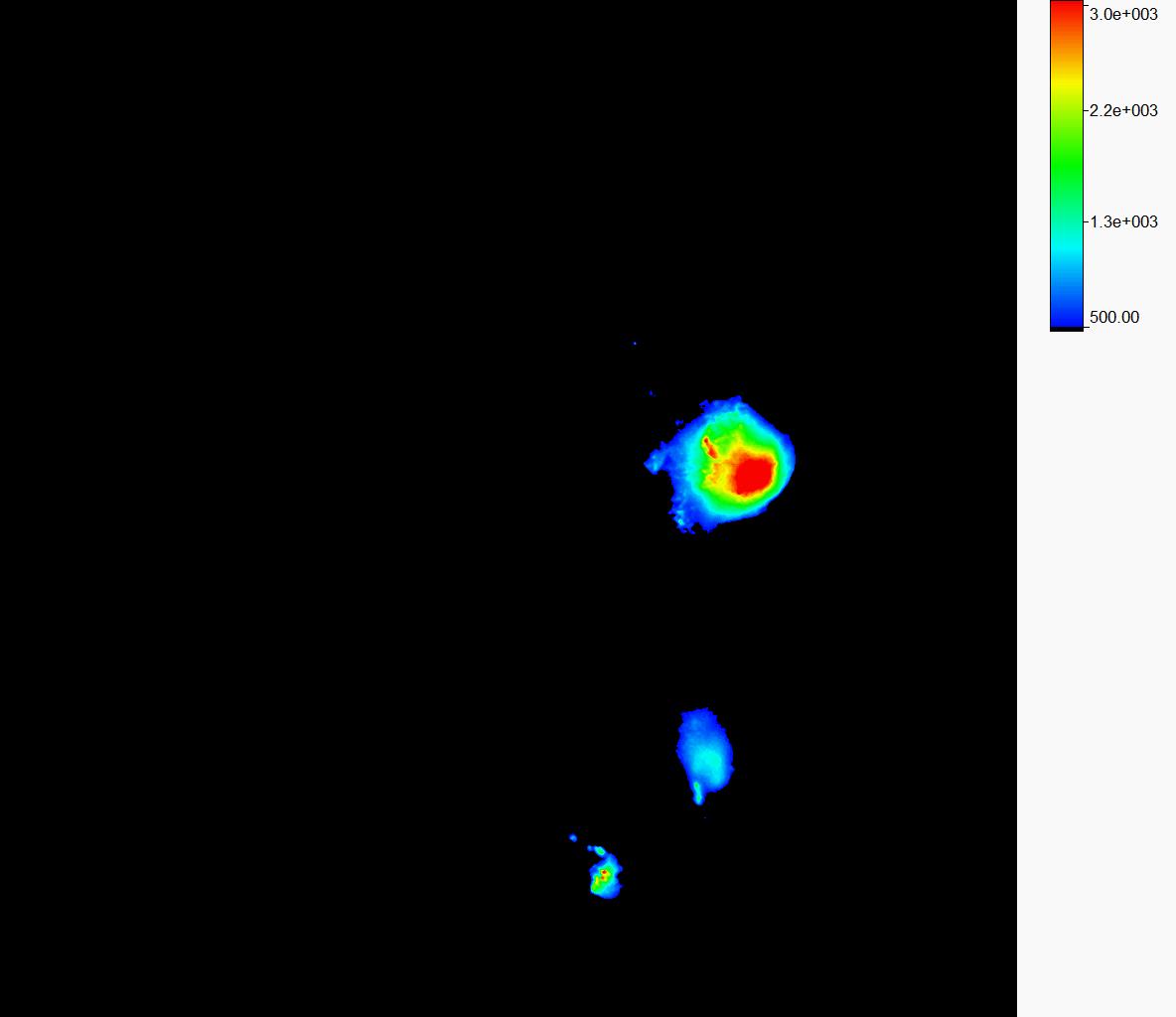

Supplement: Figure 3—source data 1. [file elife-70471-fig3-data1.zip › Figure 3-Source data/FaDu-bearing mice/Raw data-in vivo viscosity detection image in Figure 3A.jpg]

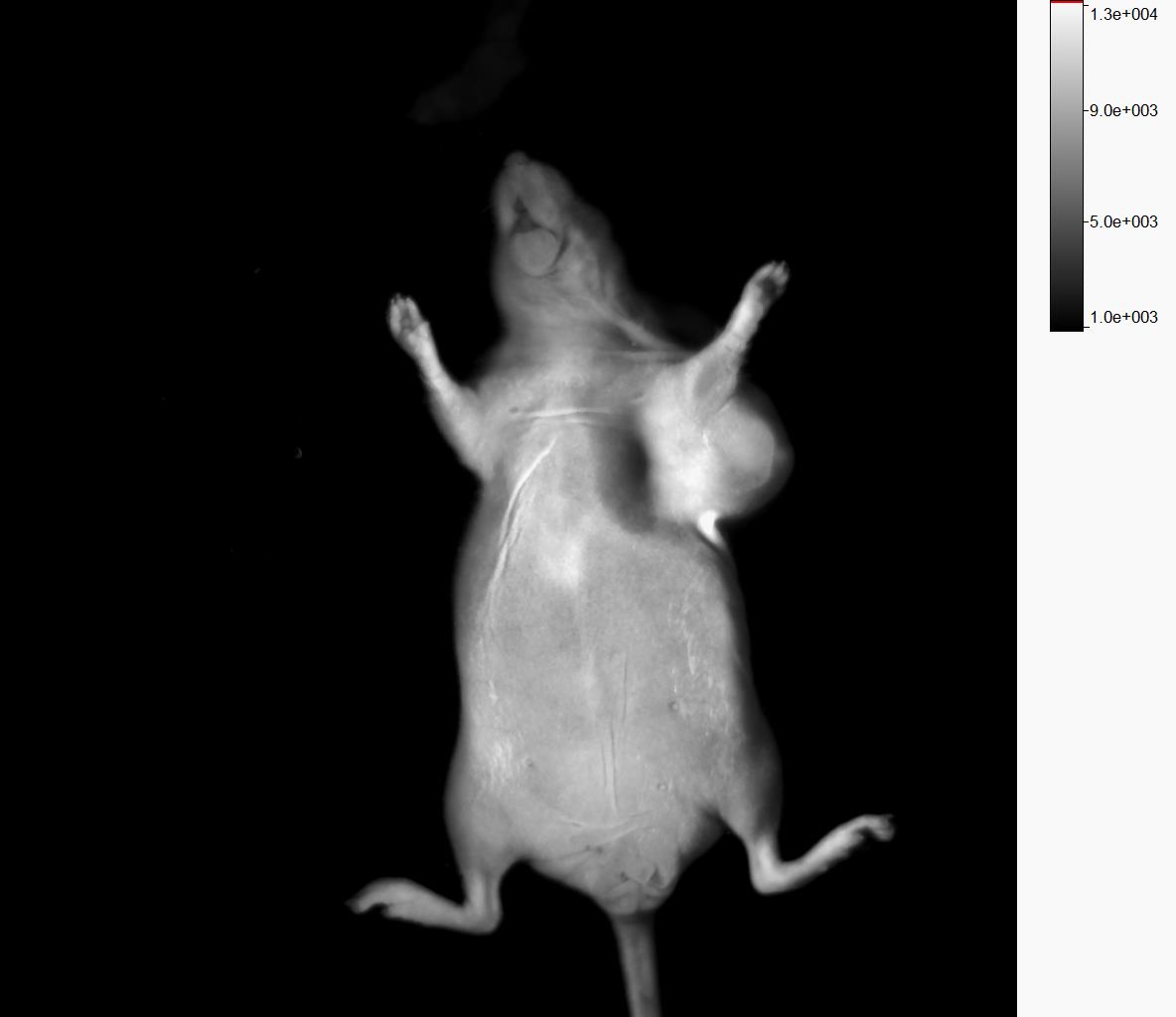

Supplement: Figure 3—source data 1. [file elife-70471-fig3-data1.zip › Figure 3-Source data/FaDu-bearing mice/Raw data-Reflectance image in Figure 3A.jpg]

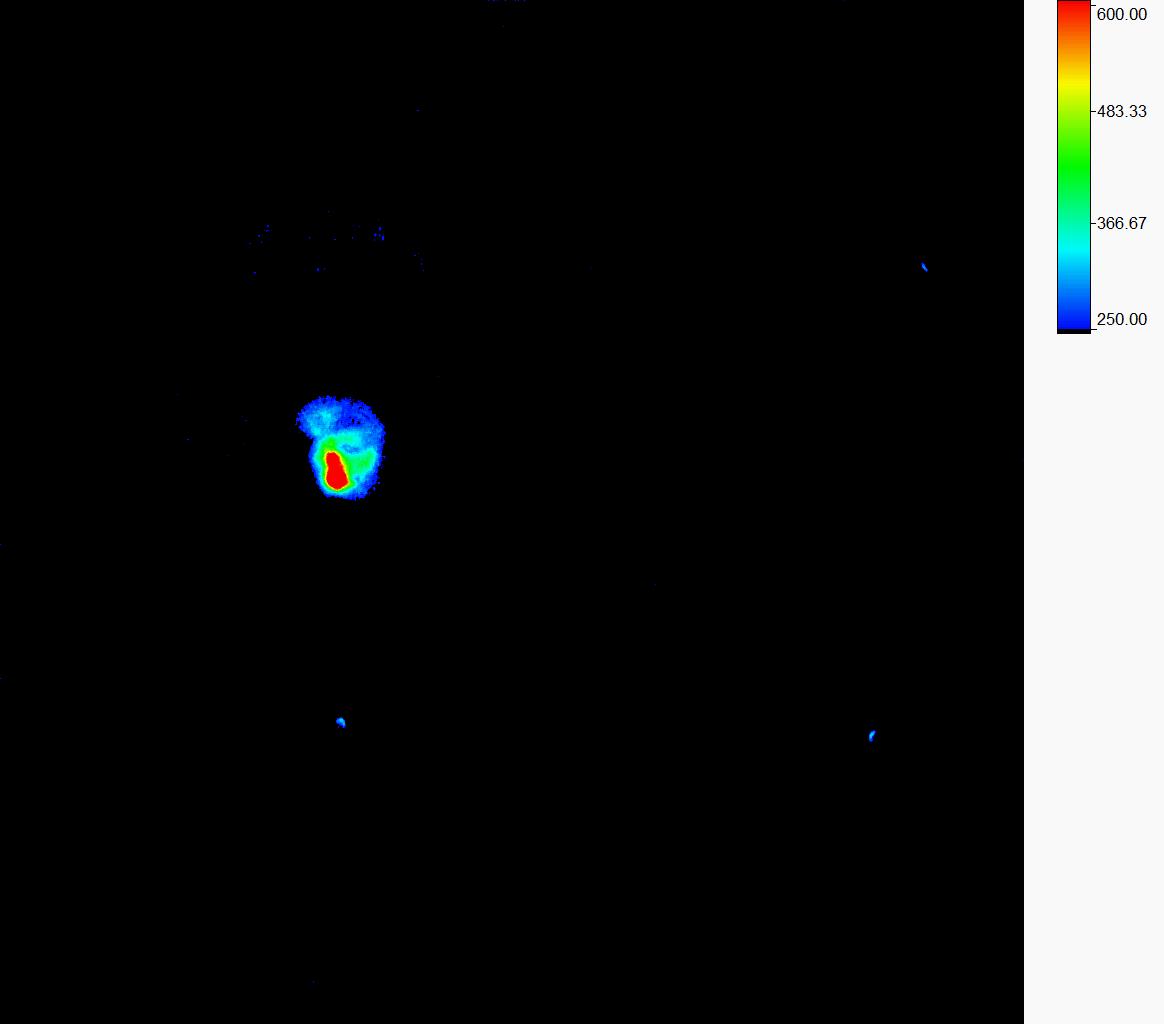

Supplement: Figure 3—source data 1. [file elife-70471-fig3-data1.zip › Figure 3-Source data/FaDu-bearing mice/Raw data-tissue nitroreductase detection image in Figure 3B.jpg]

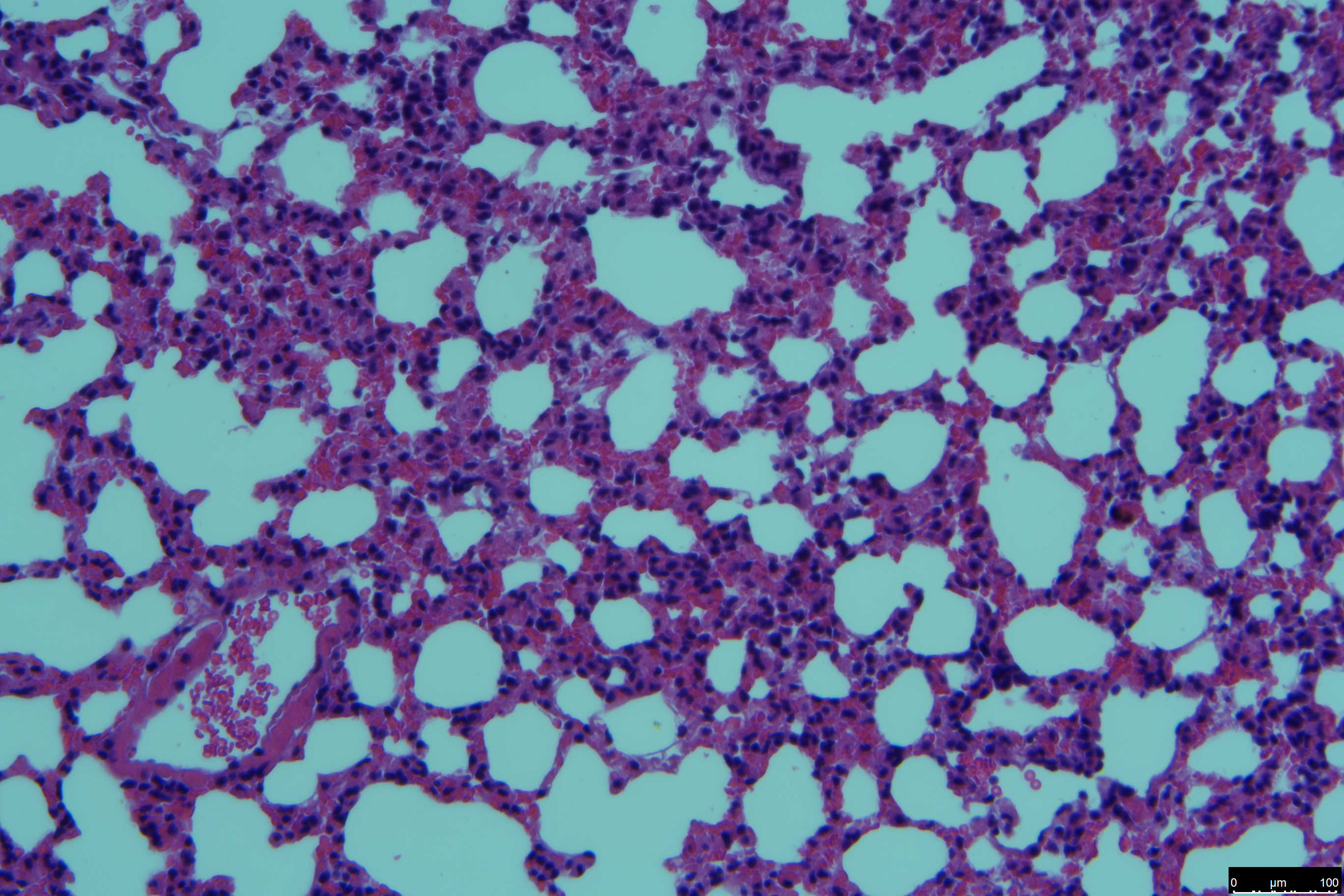

Supplement: Figure 3—source data 2. [file elife-70471-fig3-data2.zip › Figure 3-figure supplement 1-Source data /HH201111_LUNG 20X.tif]

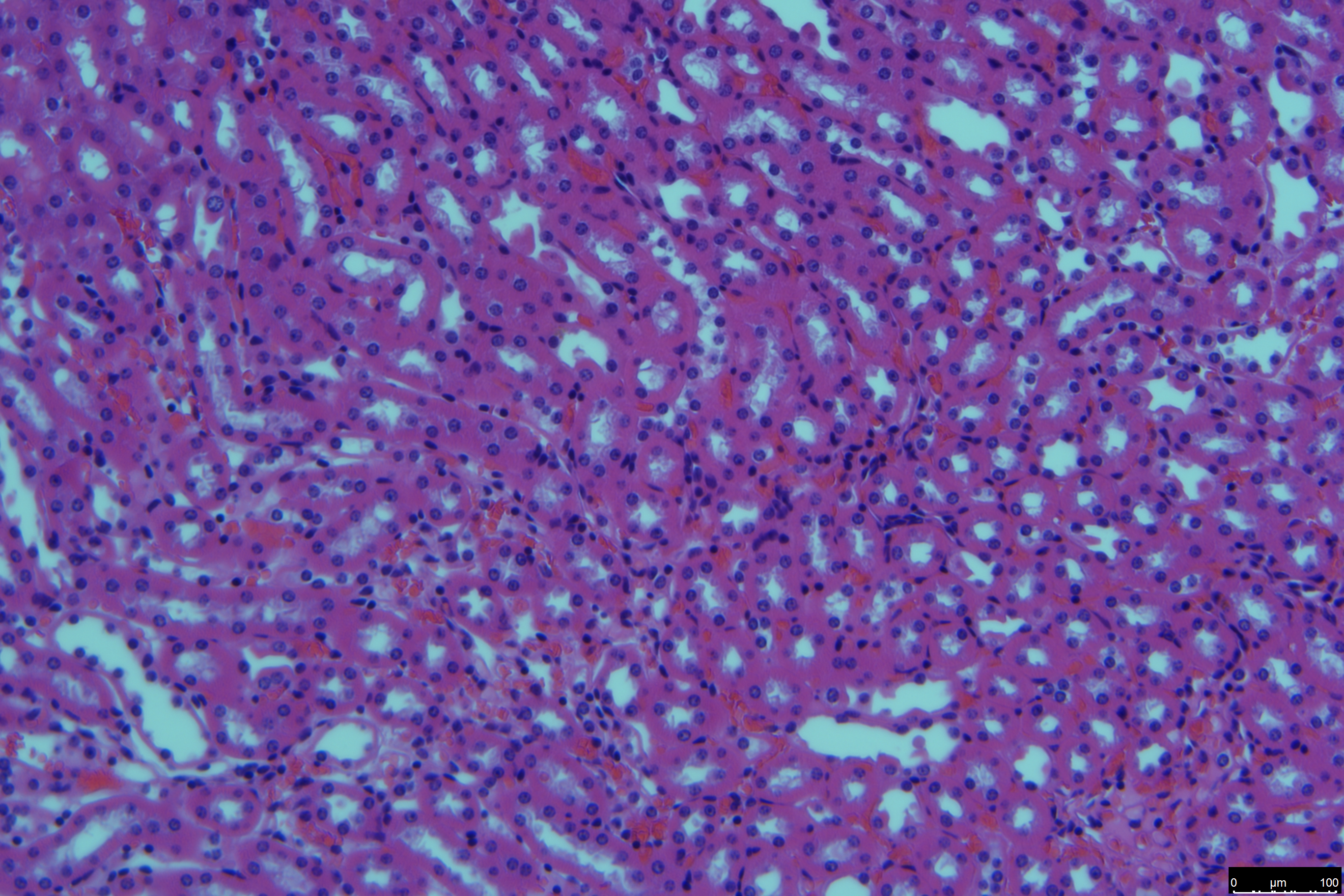

Supplement: Figure 3—source data 2. [file elife-70471-fig3-data2.zip › Figure 3-figure supplement 1-Source data /HH201111_KIDNEY 20X.tif]

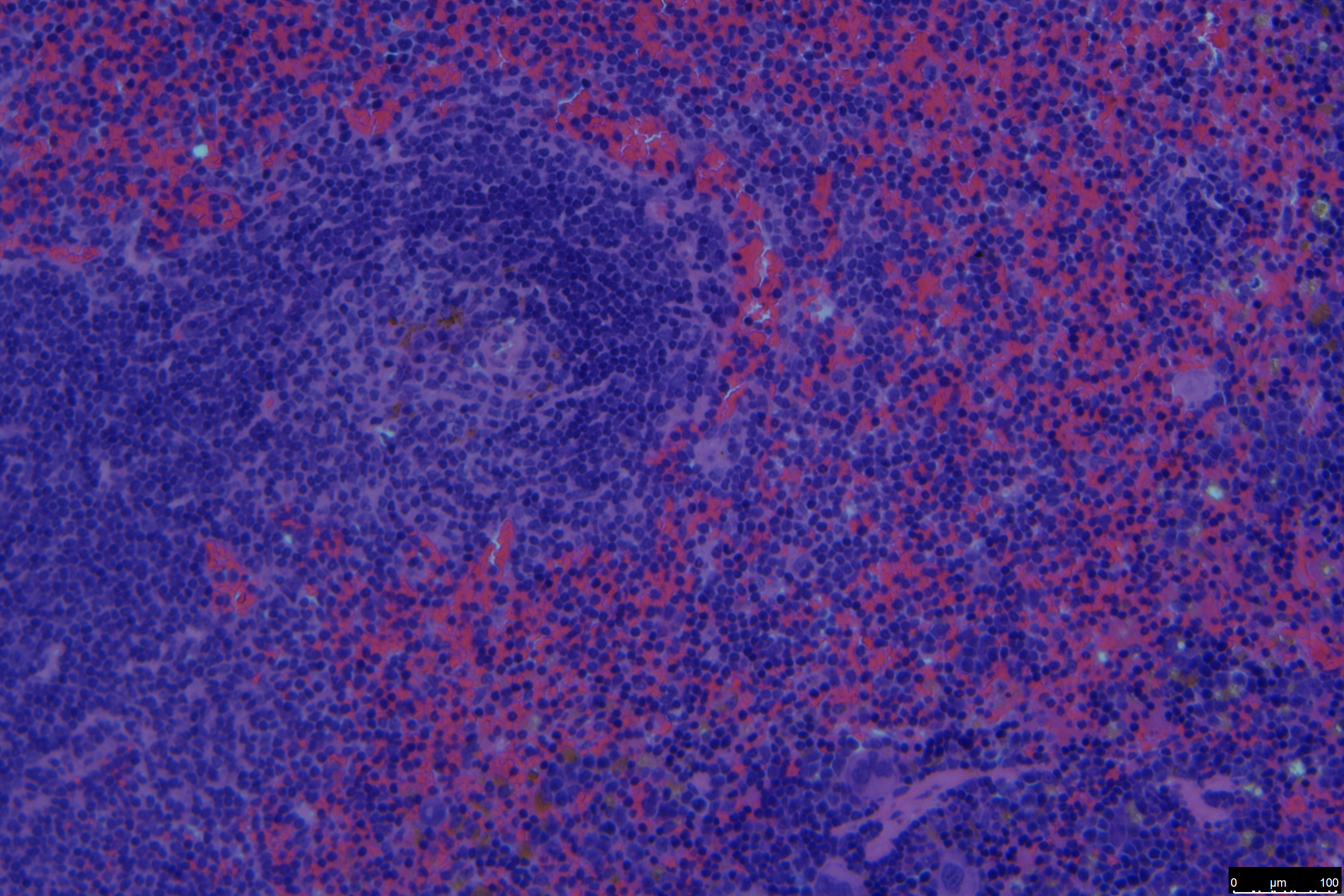

Supplement: Figure 3—source data 2. [file elife-70471-fig3-data2.zip › Figure 3-figure supplement 1-Source data /HH201111_SPLEEN 20X.tif]

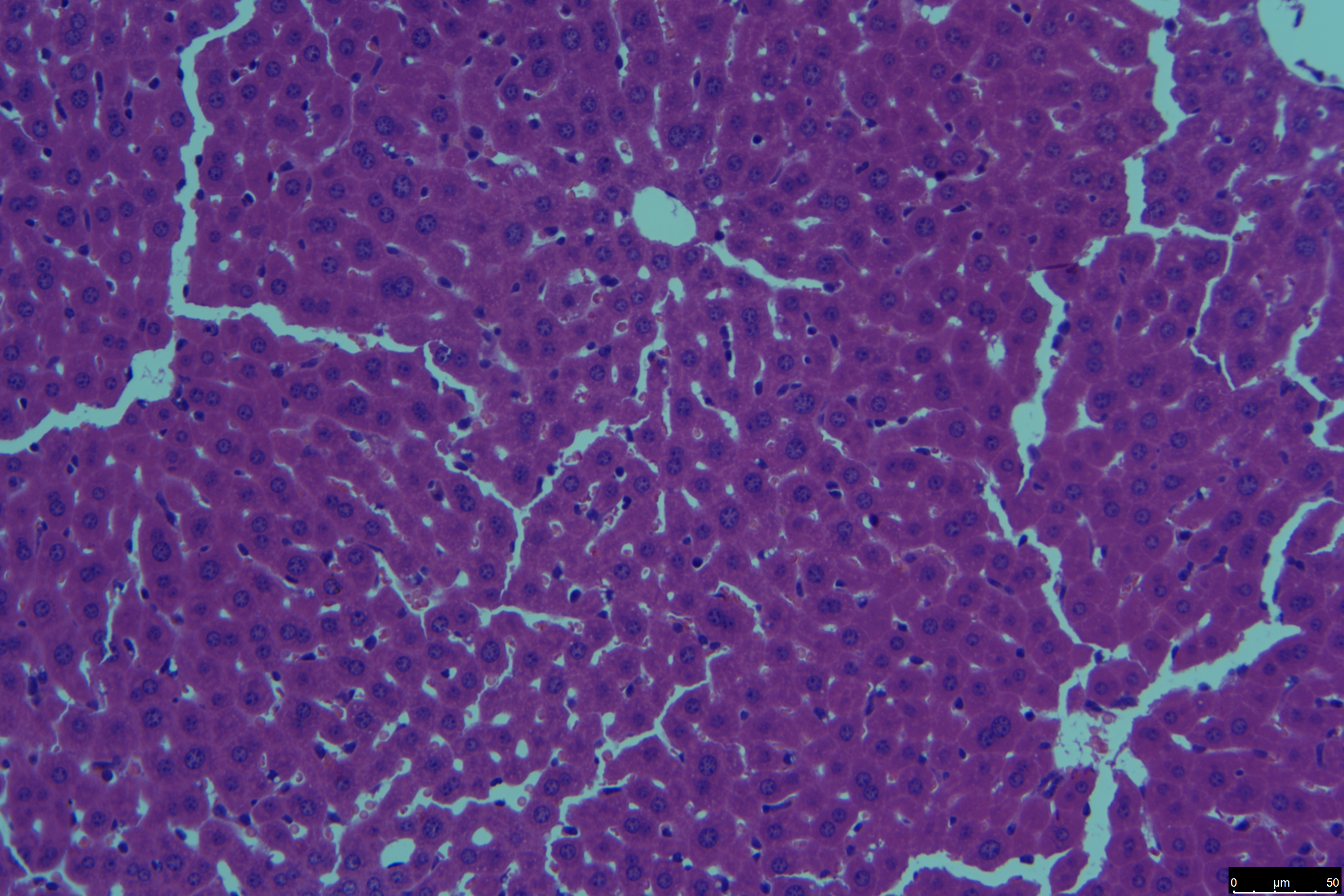

Supplement: Figure 3—source data 2. [file elife-70471-fig3-data2.zip › Figure 3-figure supplement 1-Source data /HH201111_LIVER 20X.tif]

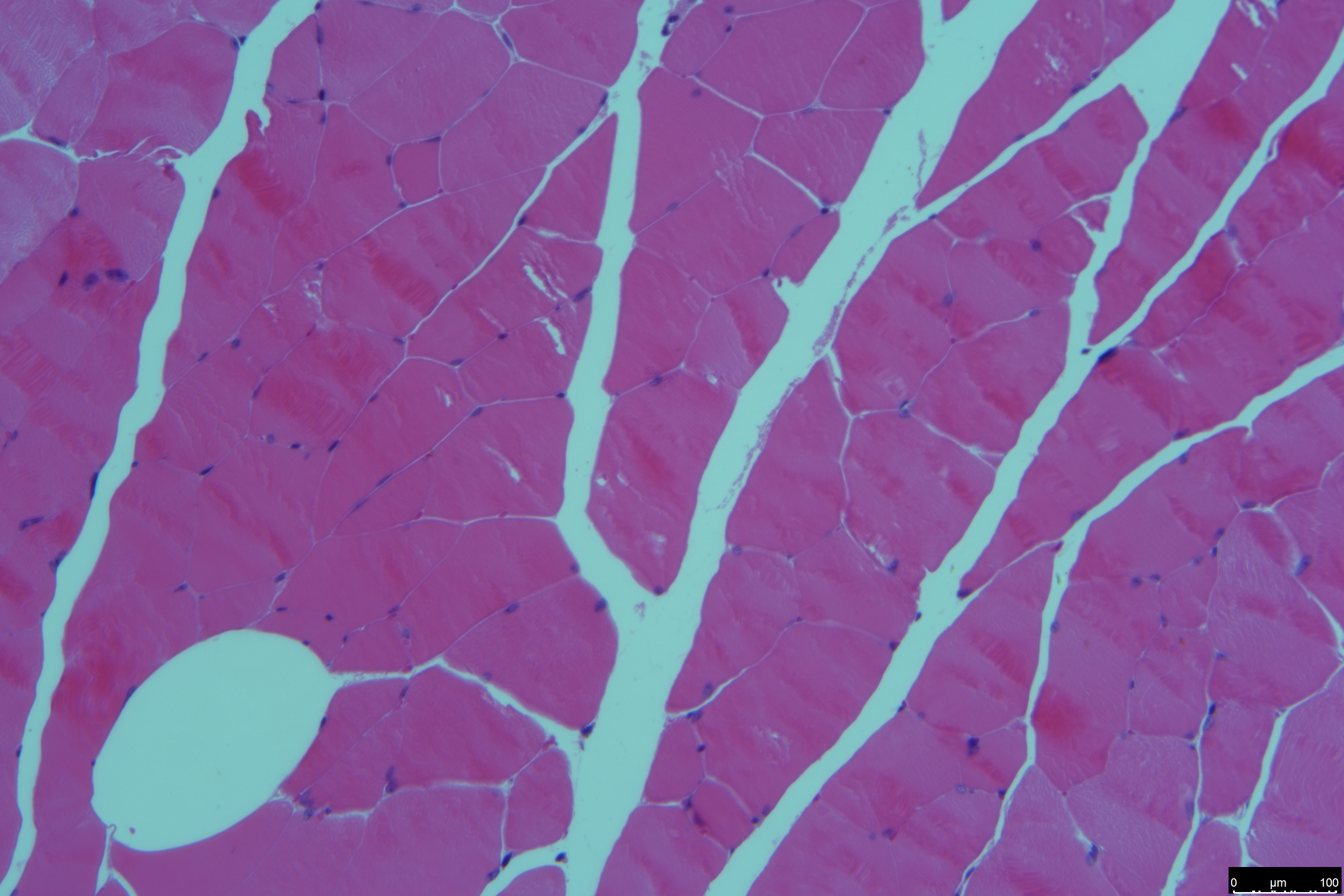

Supplement: Figure 3—source data 2. [file elife-70471-fig3-data2.zip › Figure 3-figure supplement 1-Source data /HH201111_Muscle 20X.tif]

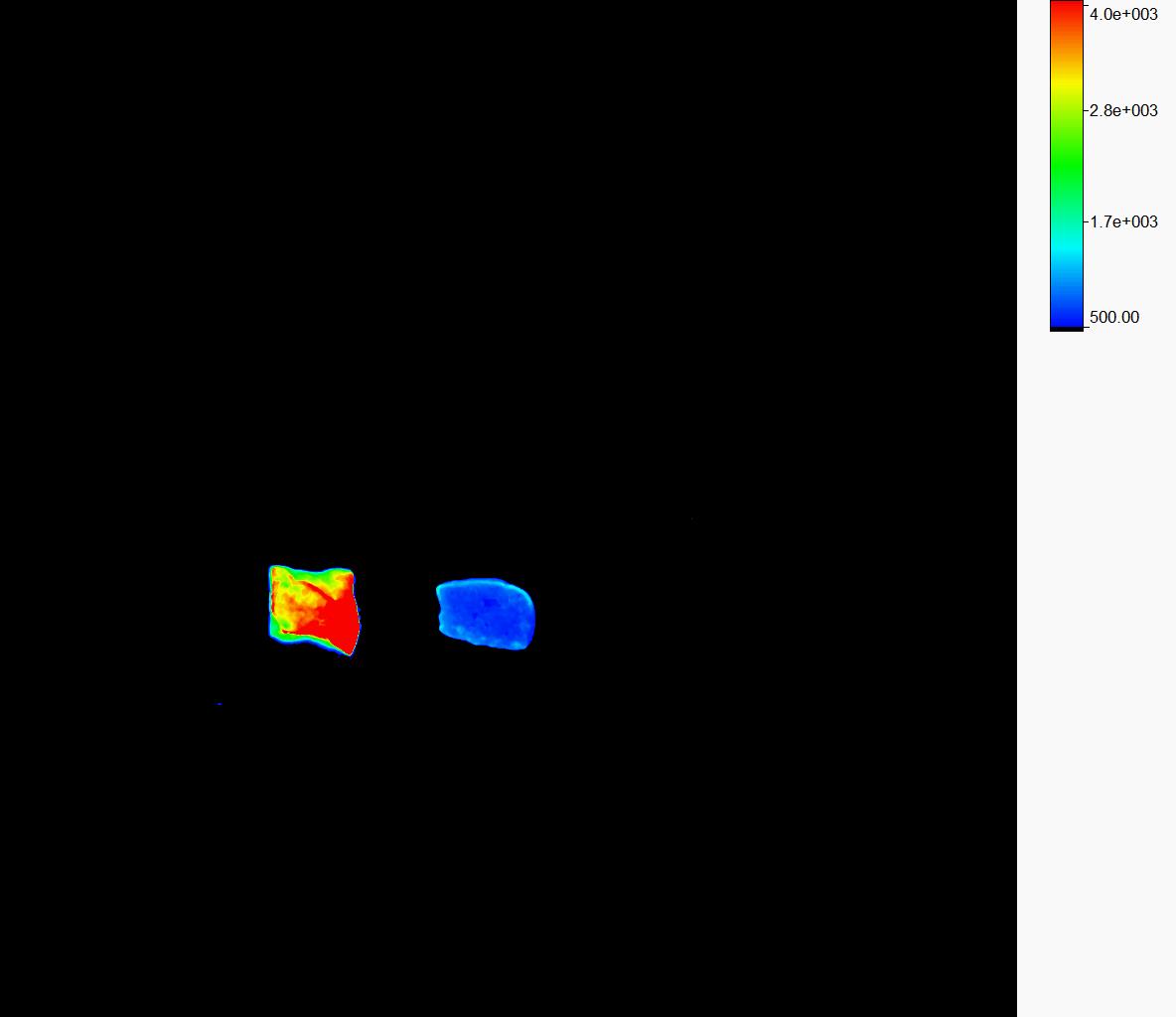

Supplement: Figure 4—source data 1. [file elife-70471-fig4-data1.zip › Figure 4-Source data/hepatocellular cancer patient 1/Raw data-viscosity detection image.jpg]
