# Supplementary figures and images for: A pre-screening strategy to assess resected tumor margins by imaging cytoplasmic viscosity and hypoxia (part 2 of 3)
Source: eLife. 2021 Oct 11;10:e70471. doi: 10.7554/eLife.70471 (PMC8553343; doi:10.7554/eLife.70471)

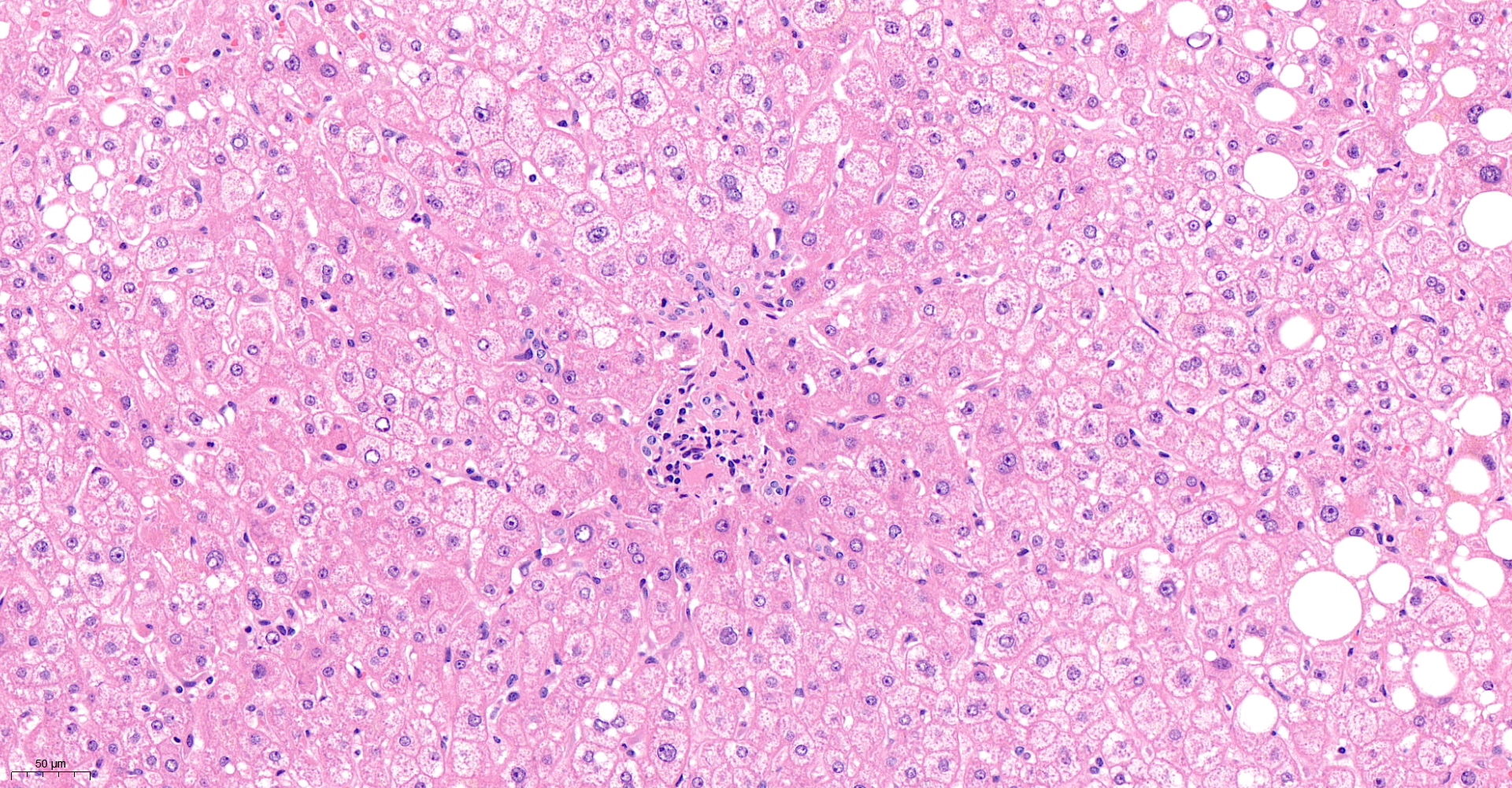

Supplement: Figure 4—source data 1. [file elife-70471-fig4-data1.zip › Figure 4-Source data/hepatocellular cancer patient 1/Raw data-HE staining image 1 of patient 1-20.0x.jpg]

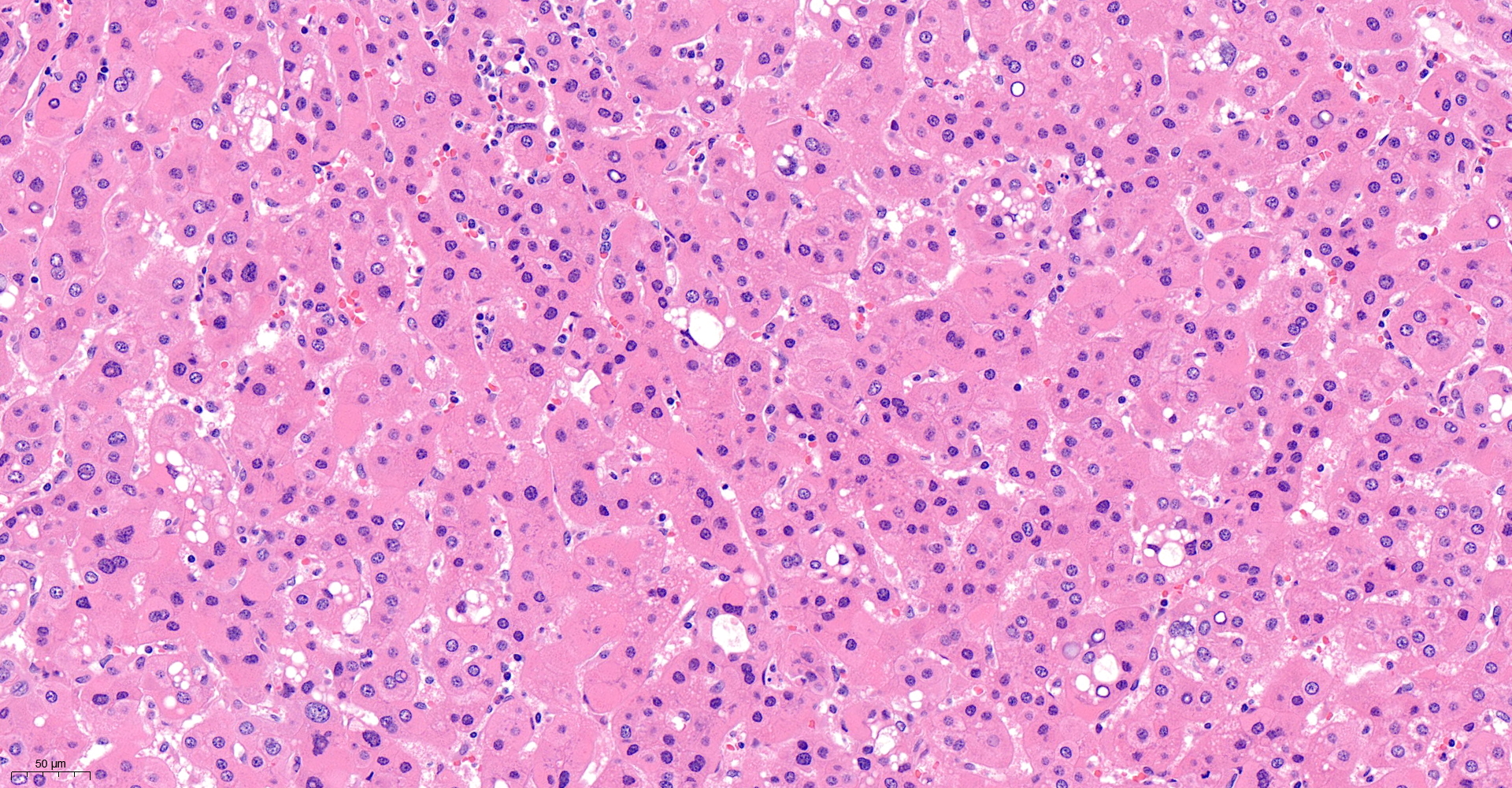

Supplement: Figure 4—source data 1. [file elife-70471-fig4-data1.zip › Figure 4-Source data/hepatocellular cancer patient 1/Raw data-HE staining image 2 of patient 1-20.0x.jpg]

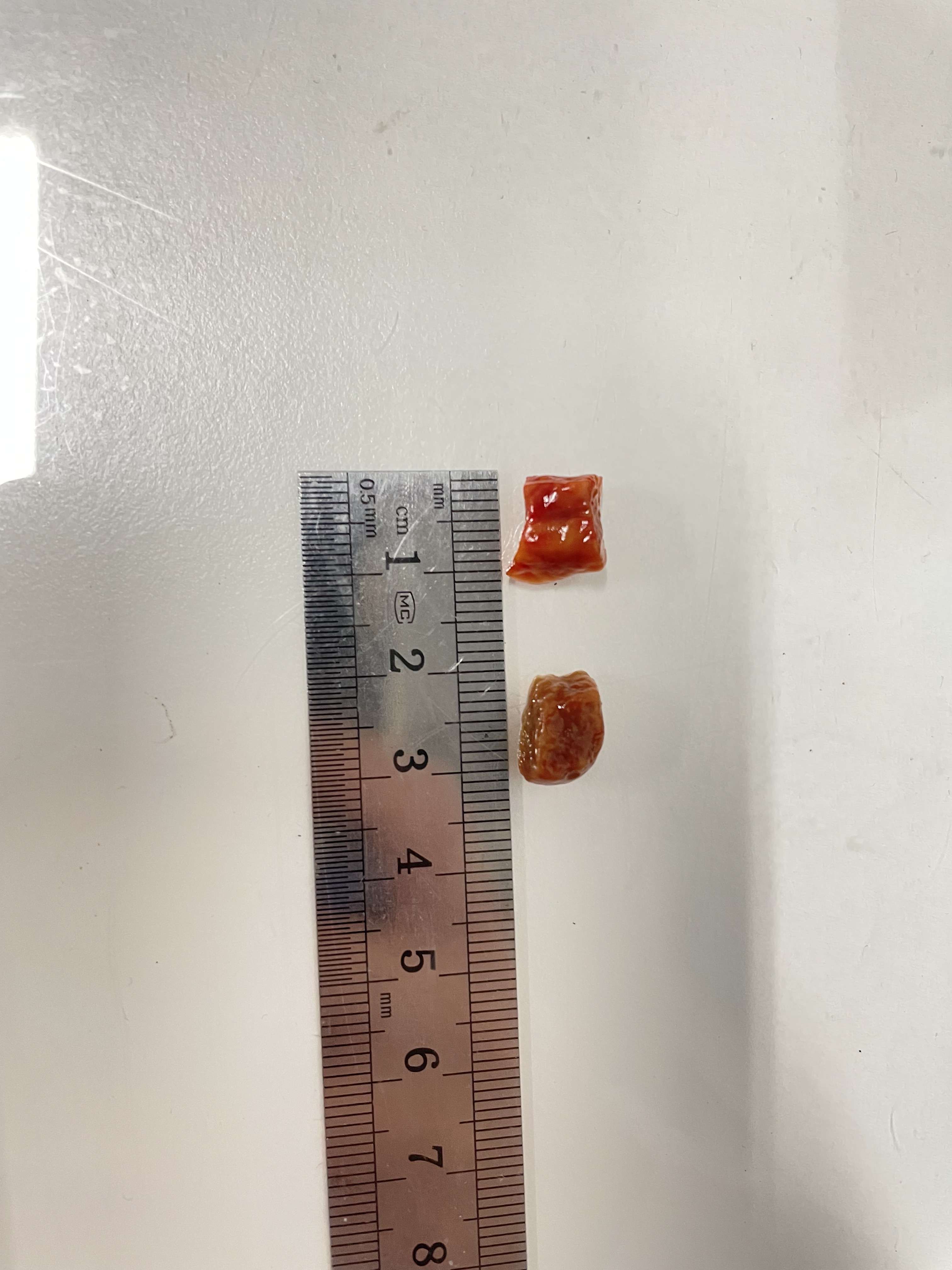

Supplement: Figure 4—source data 1. [file elife-70471-fig4-data1.zip › Figure 4-Source data/hepatocellular cancer patient 1/Raw data-photograph image.JPG]

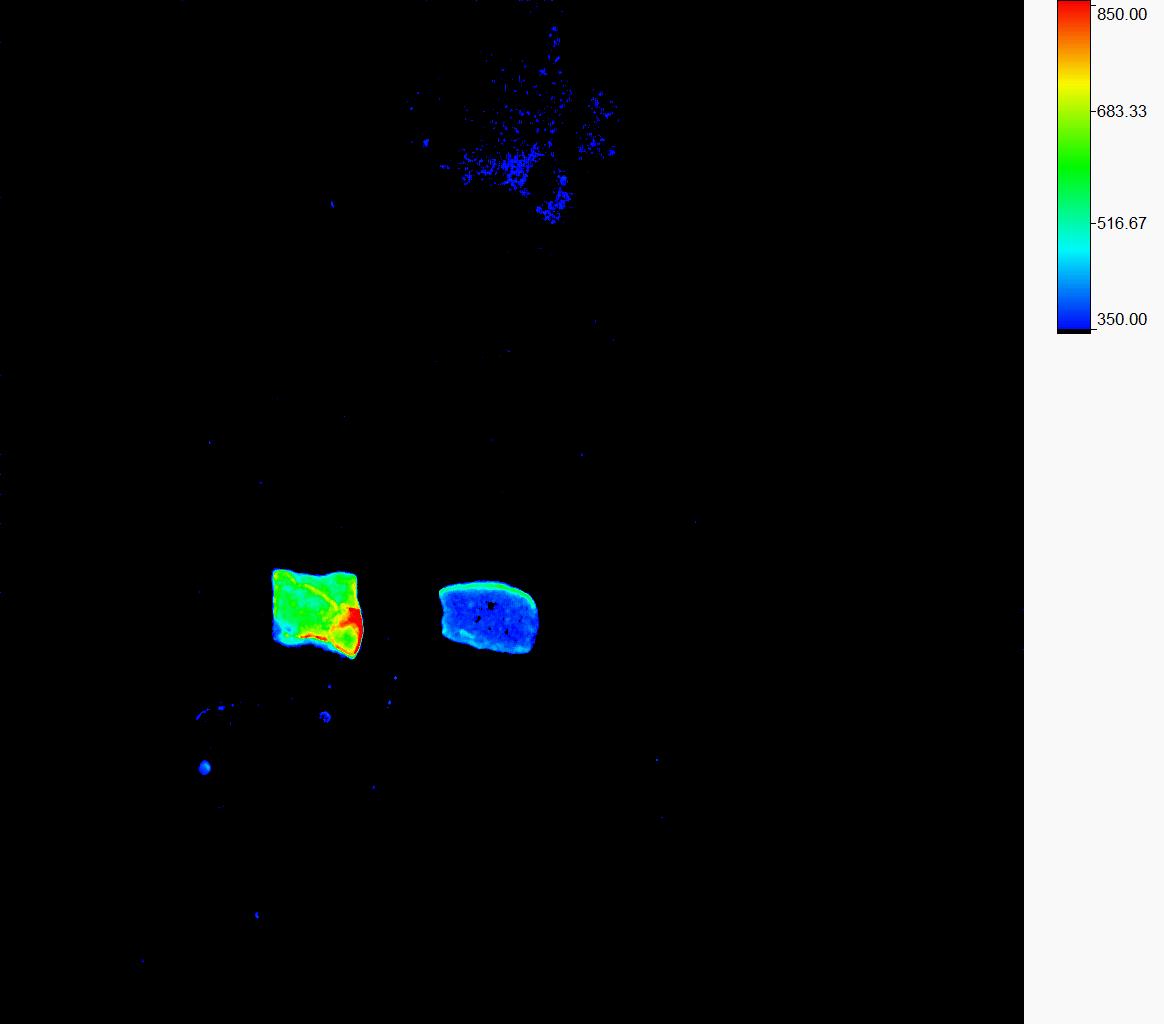

Supplement: Figure 4—source data 1. [file elife-70471-fig4-data1.zip › Figure 4-Source data/hepatocellular cancer patient 1/Raw data-nitroreductase detection image.jpg]

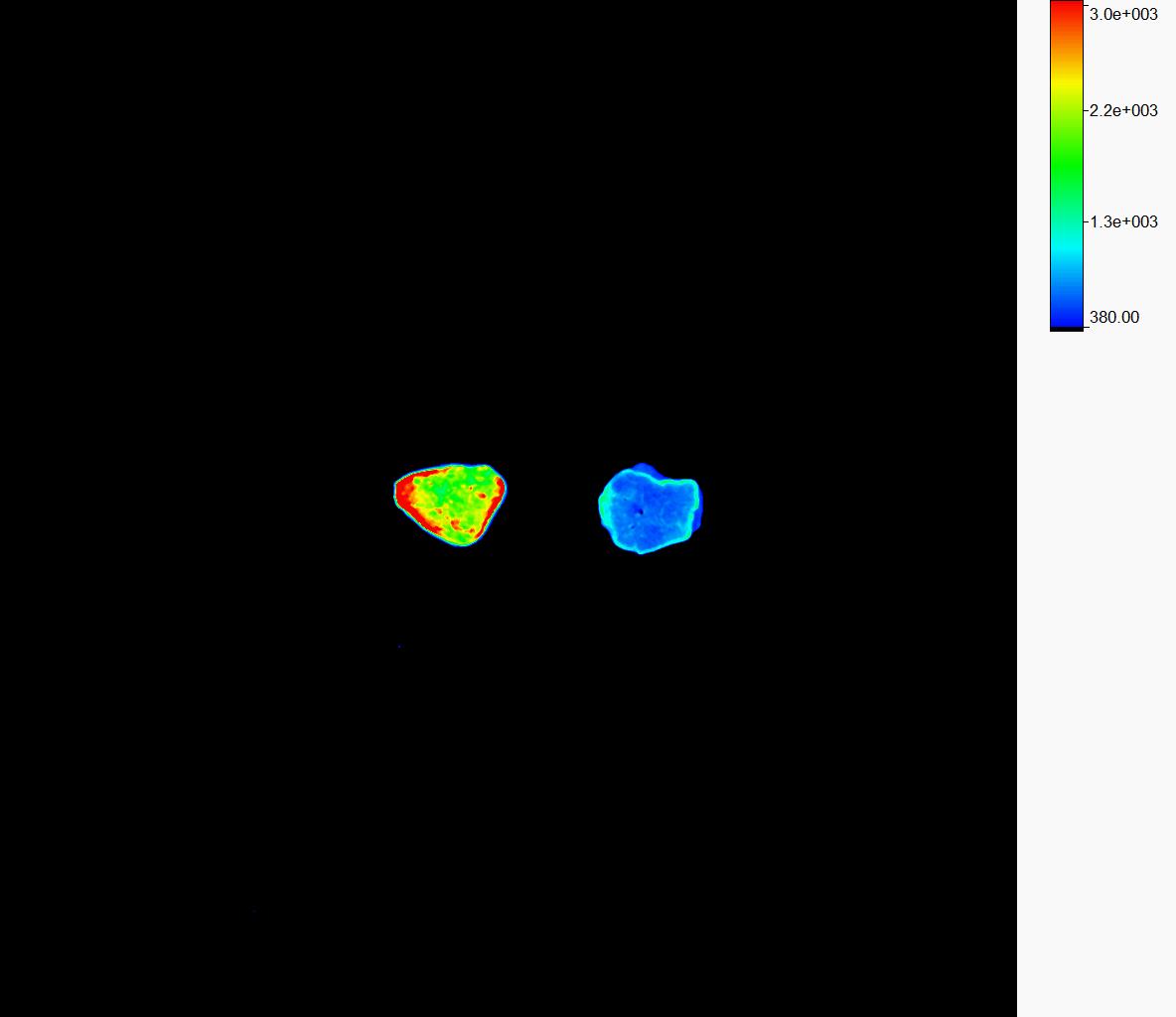

Supplement: Figure 4—source data 1. [file elife-70471-fig4-data1.zip › Figure 4-Source data/hepatocellular cancer patient 6/Raw data-viscosity detection image.jpg]

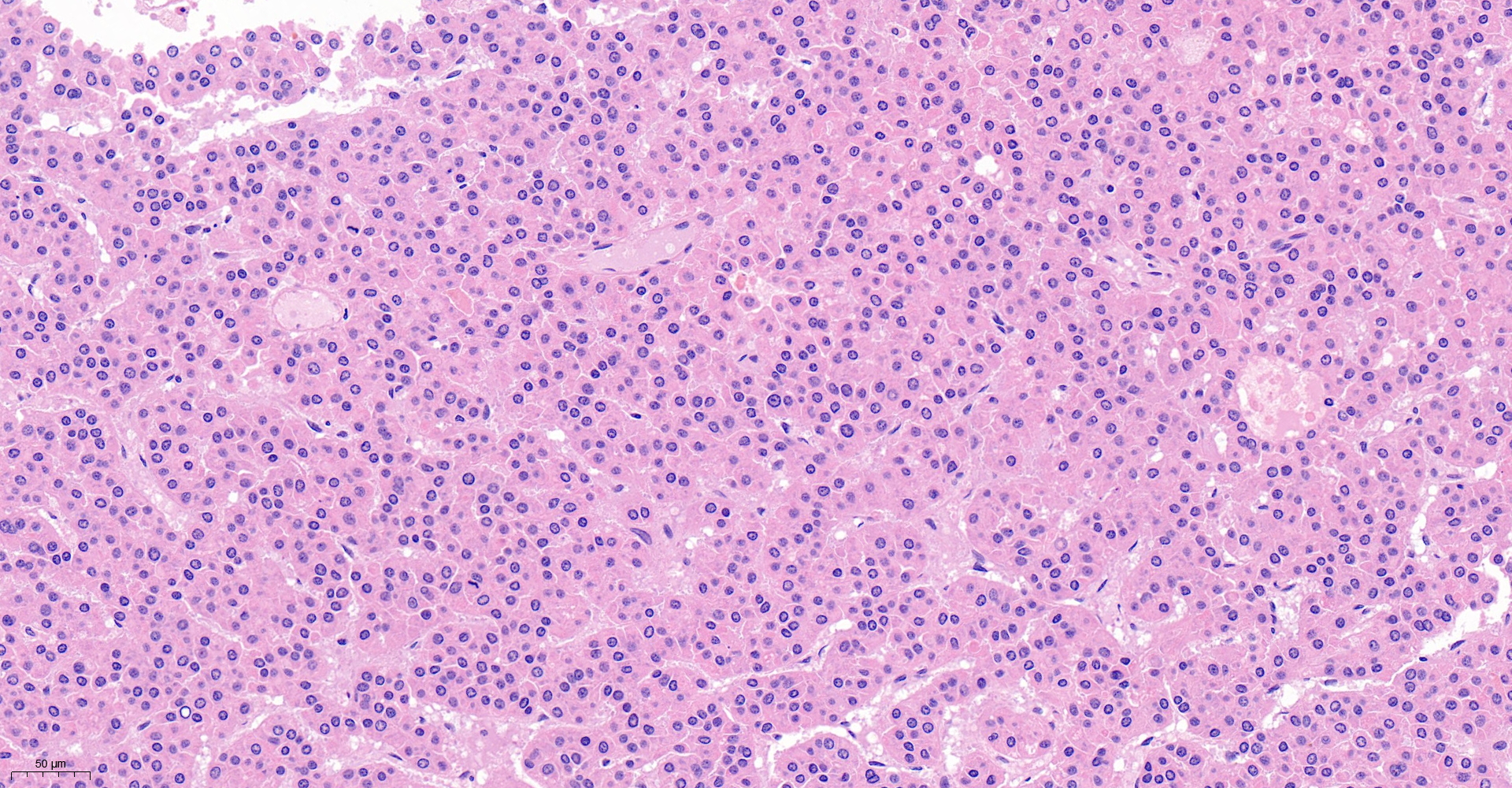

Supplement: Figure 4—source data 1. [file elife-70471-fig4-data1.zip › Figure 4-Source data/hepatocellular cancer patient 6/Raw data-HE staining image 2 of patient 6-20.0x.jpg]

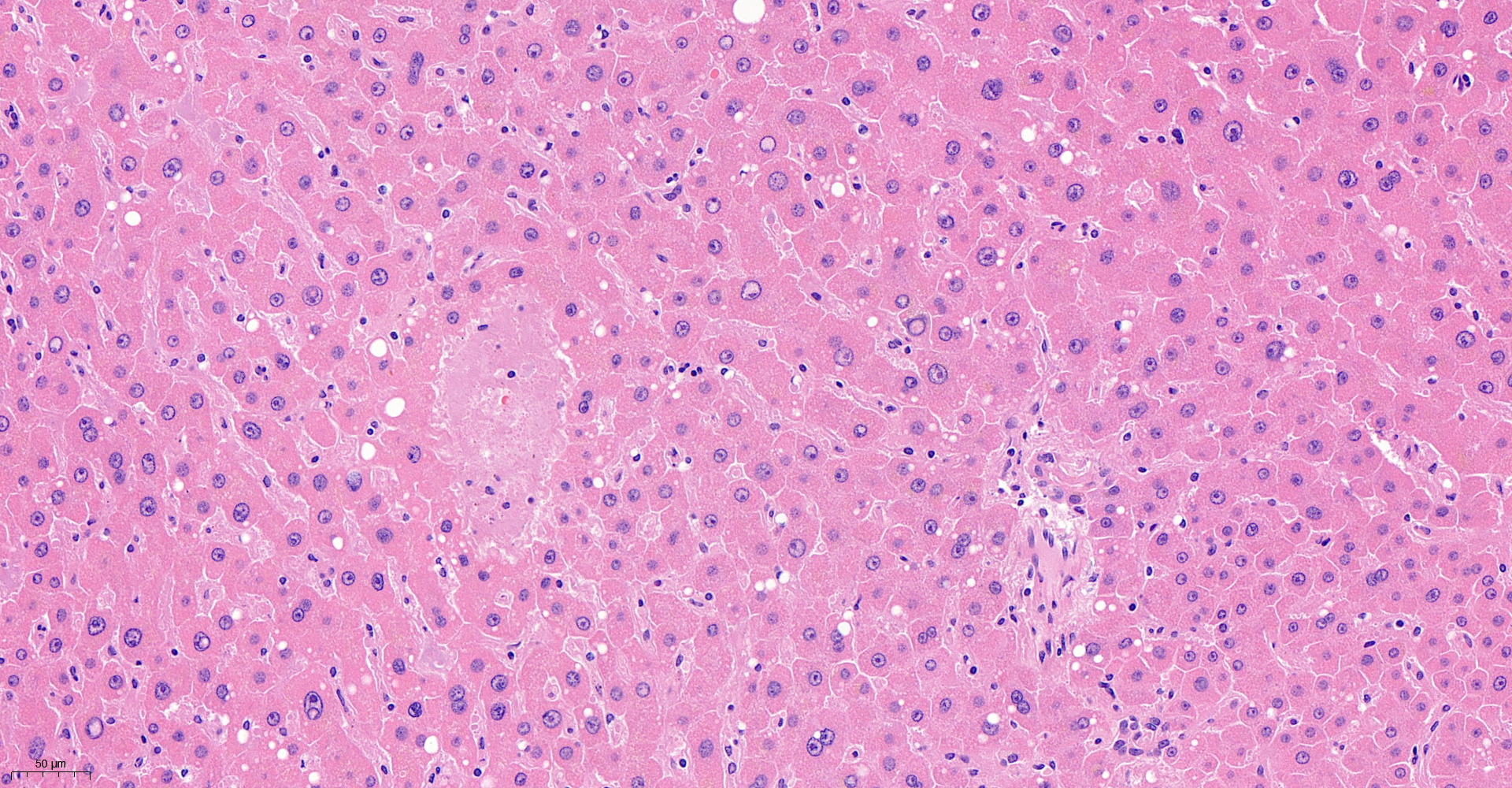

Supplement: Figure 4—source data 1. [file elife-70471-fig4-data1.zip › Figure 4-Source data/hepatocellular cancer patient 6/Raw data-HE staining image 1 of patient 6-20.0x.jpg]

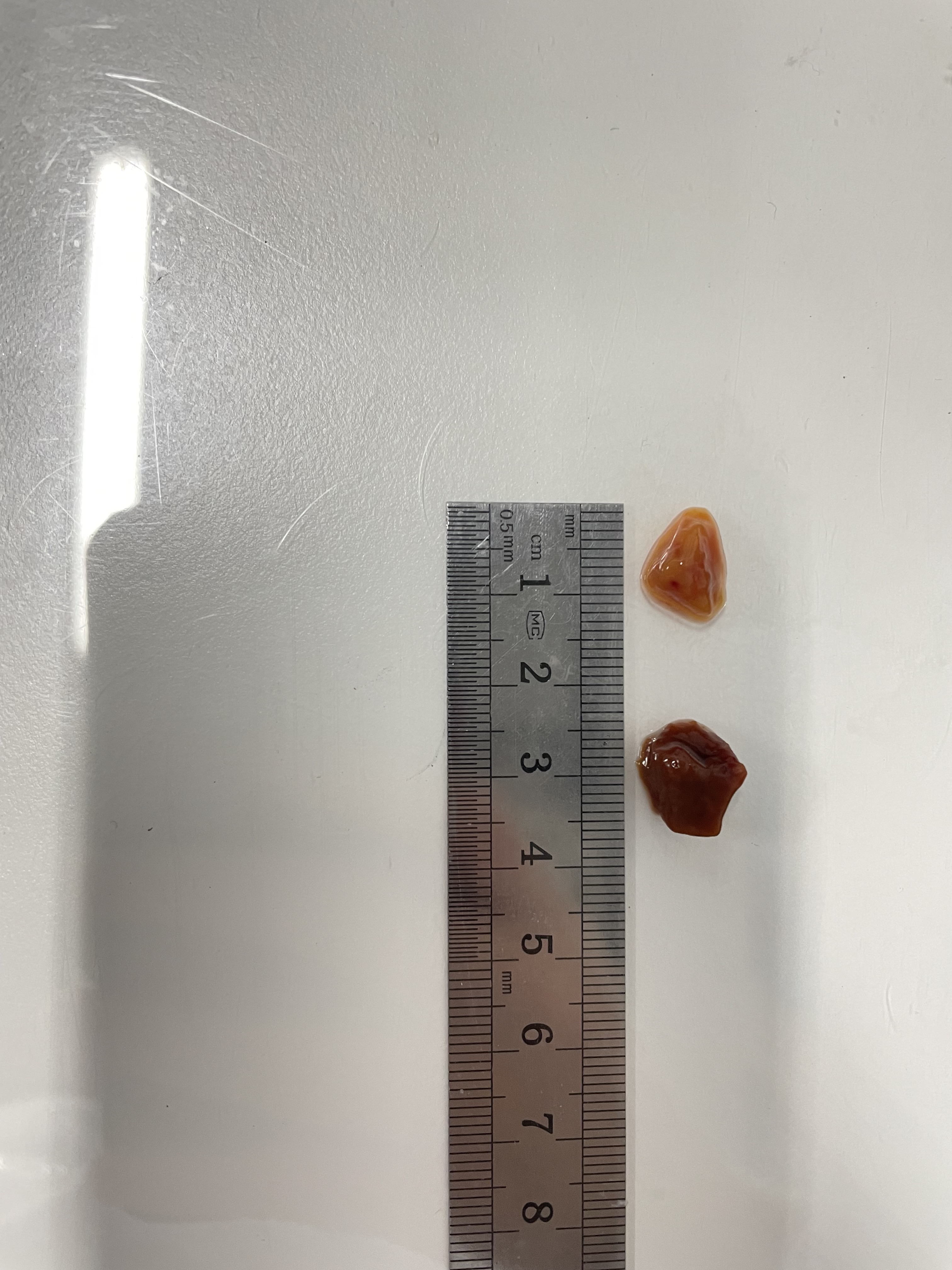

Supplement: Figure 4—source data 1. [file elife-70471-fig4-data1.zip › Figure 4-Source data/hepatocellular cancer patient 6/Raw data-photograph image.JPG]

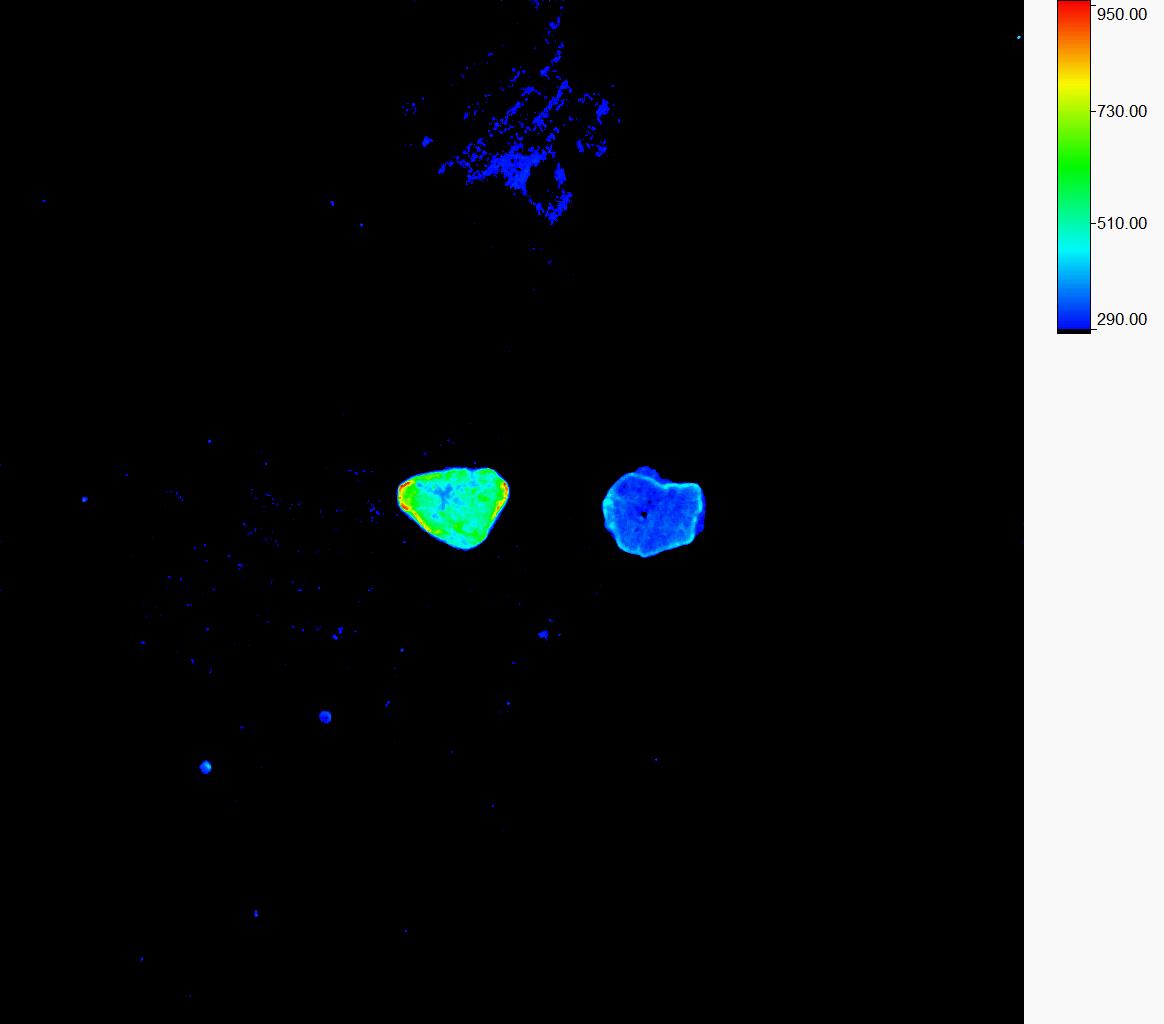

Supplement: Figure 4—source data 1. [file elife-70471-fig4-data1.zip › Figure 4-Source data/hepatocellular cancer patient 6/Raw data-nitroreductase detection image.jpg]

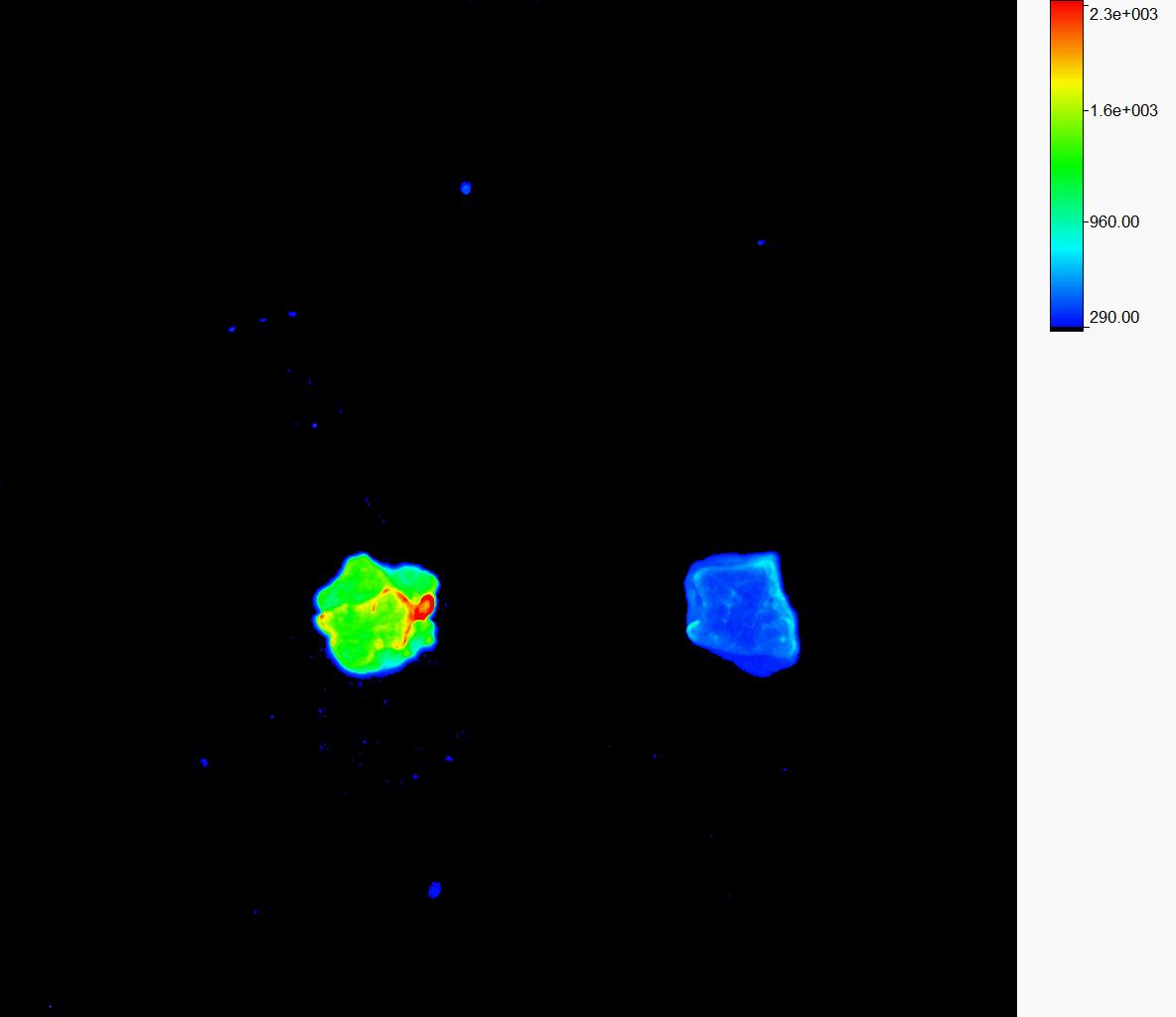

Supplement: Figure 4—source data 1. [file elife-70471-fig4-data1.zip › Figure 4-Source data/hepatocellular cancer patient 8/Raw data-viscosity detection image.jpg]

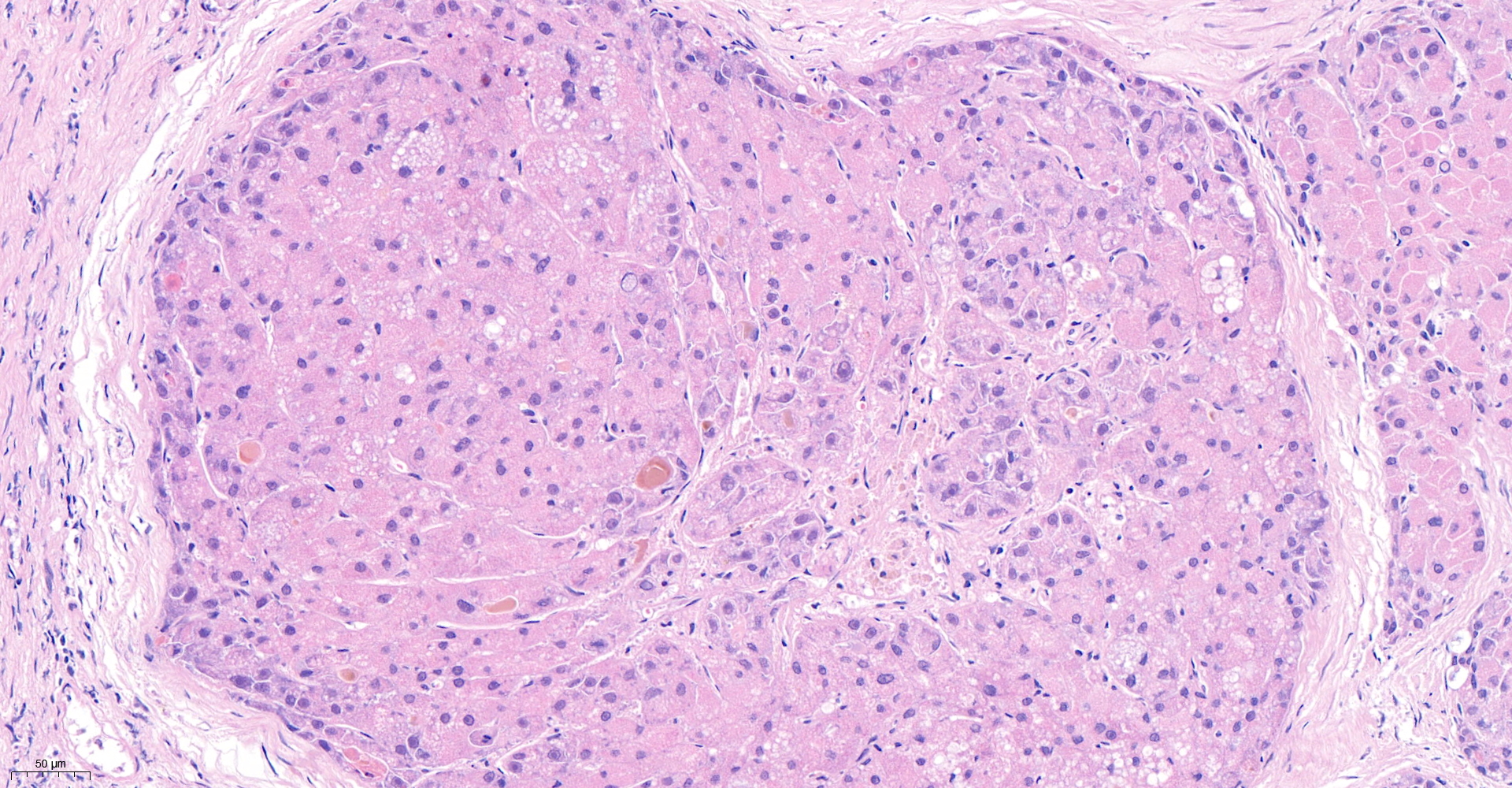

Supplement: Figure 4—source data 1. [file elife-70471-fig4-data1.zip › Figure 4-Source data/hepatocellular cancer patient 8/Raw data-HE staining image 2 of patient 8-20.0x.jpg]

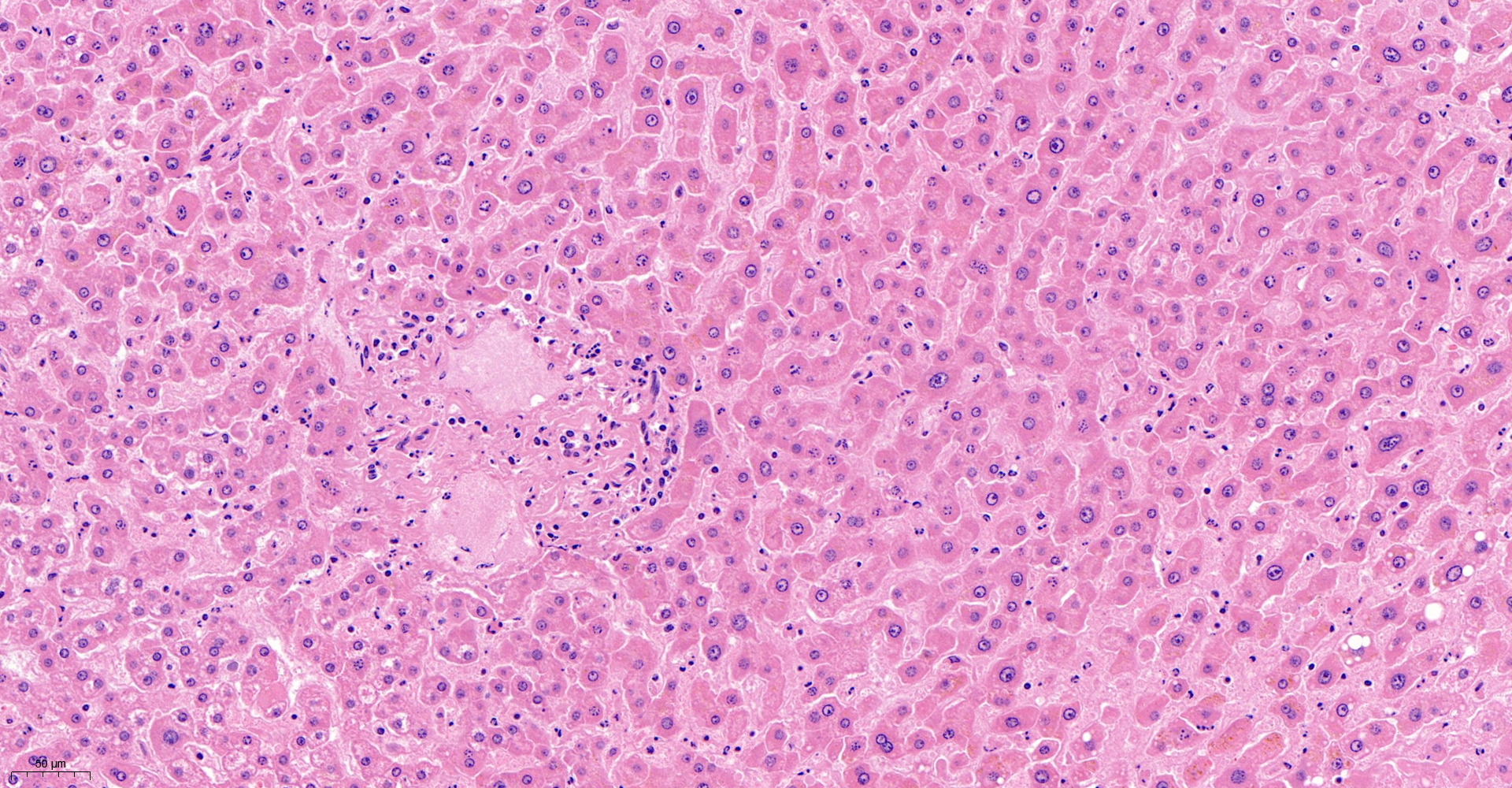

Supplement: Figure 4—source data 1. [file elife-70471-fig4-data1.zip › Figure 4-Source data/hepatocellular cancer patient 8/Raw data-HE staining image 1 of patient 8-20.0x.jpg]

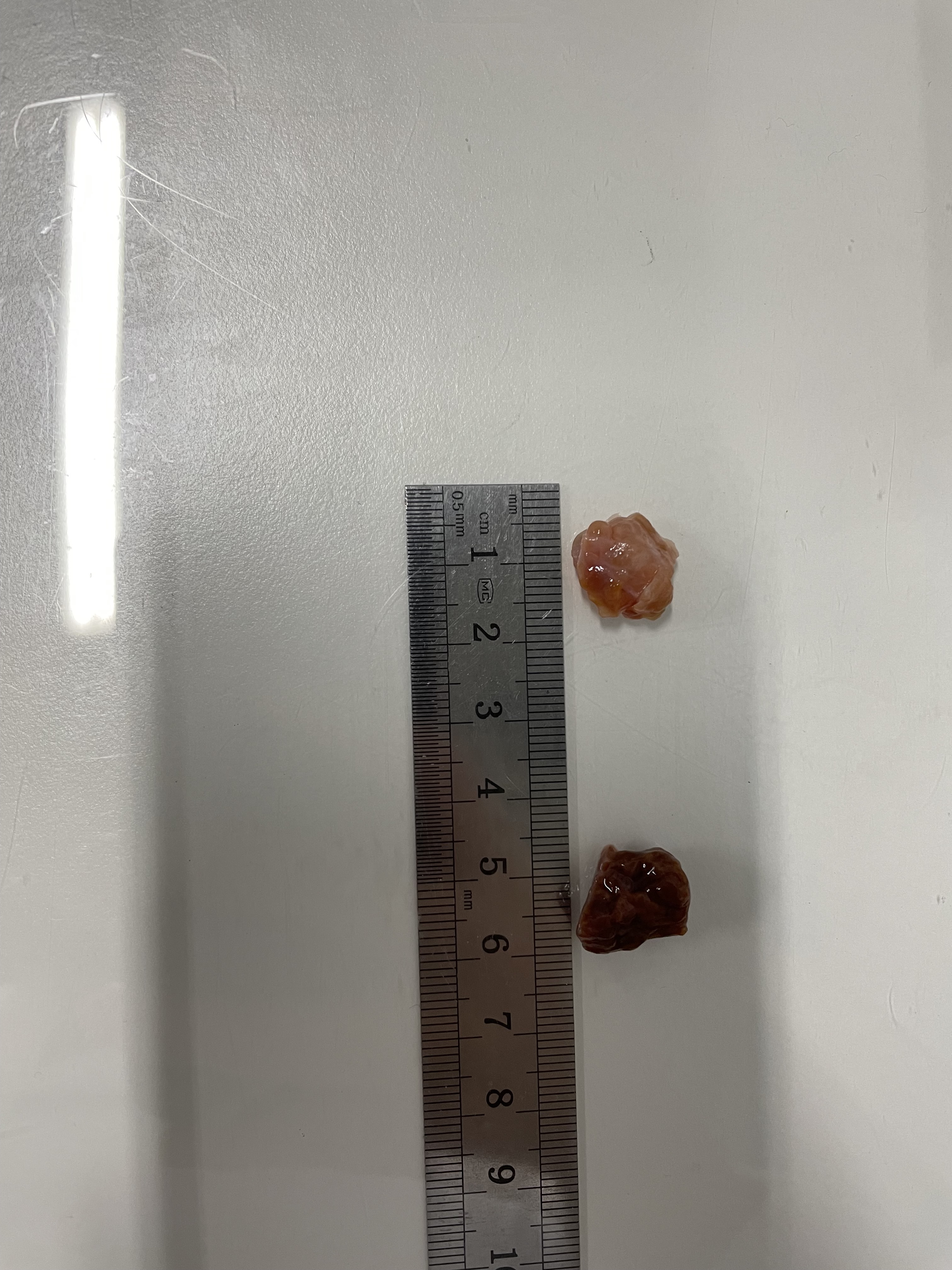

Supplement: Figure 4—source data 1. [file elife-70471-fig4-data1.zip › Figure 4-Source data/hepatocellular cancer patient 8/Raw data-photograph image.JPG]

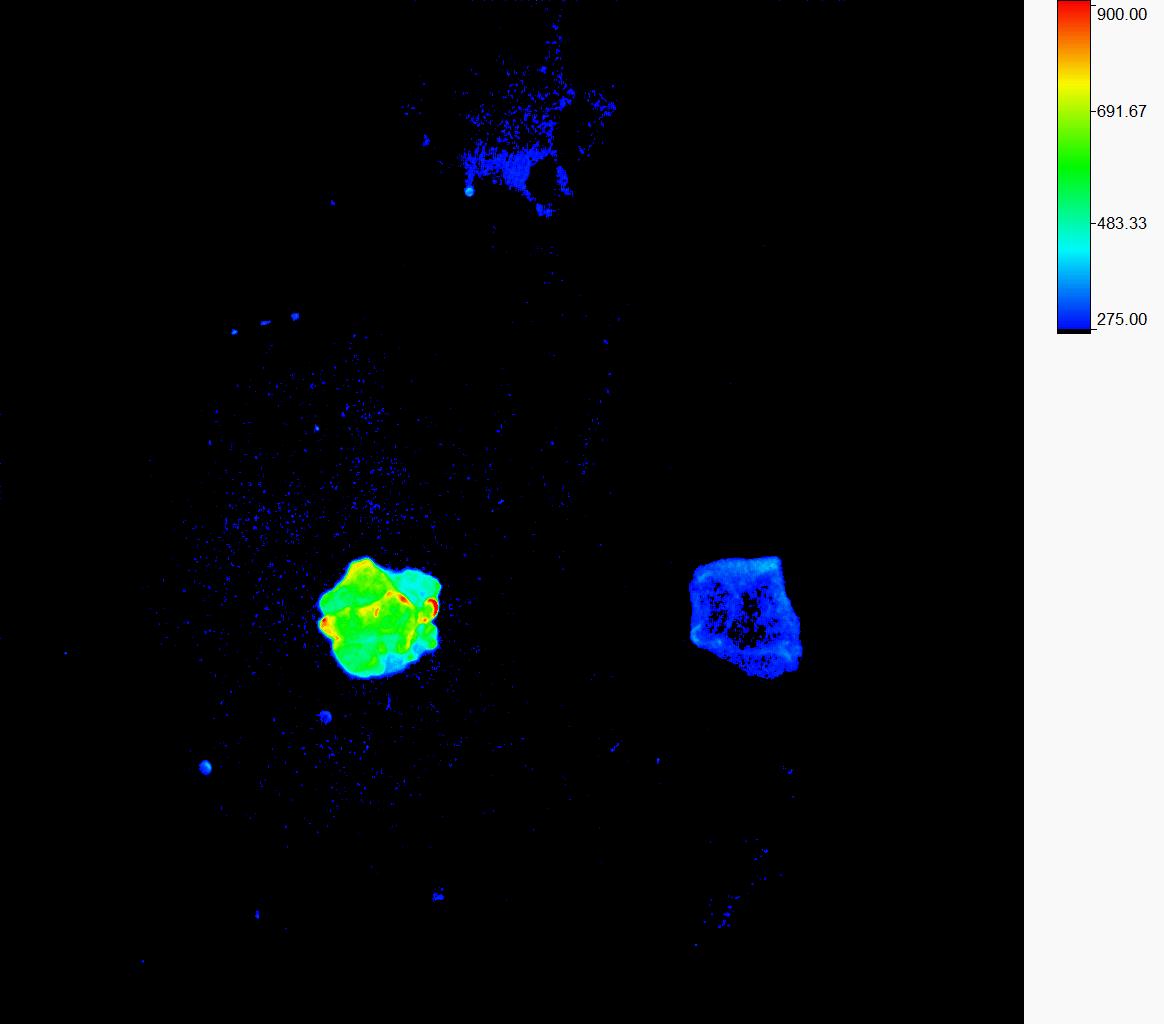

Supplement: Figure 4—source data 1. [file elife-70471-fig4-data1.zip › Figure 4-Source data/hepatocellular cancer patient 8/Raw data-nitroreductase detection image.jpg]

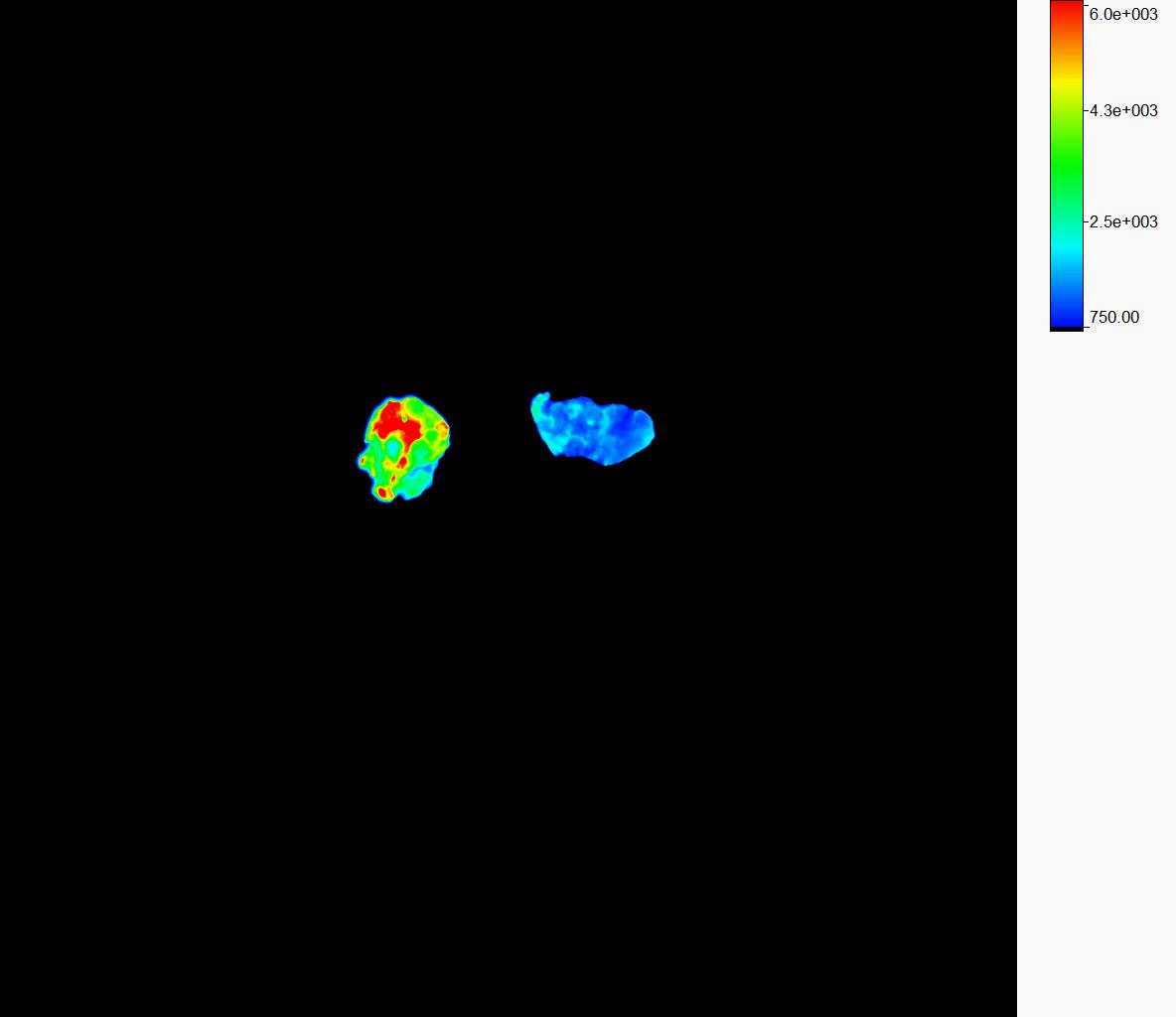

Supplement: Figure 4—source data 1. [file elife-70471-fig4-data1.zip › Figure 4-Source data/hepatocellular cancer patient 9/Raw data-viscosity detection image.jpg]

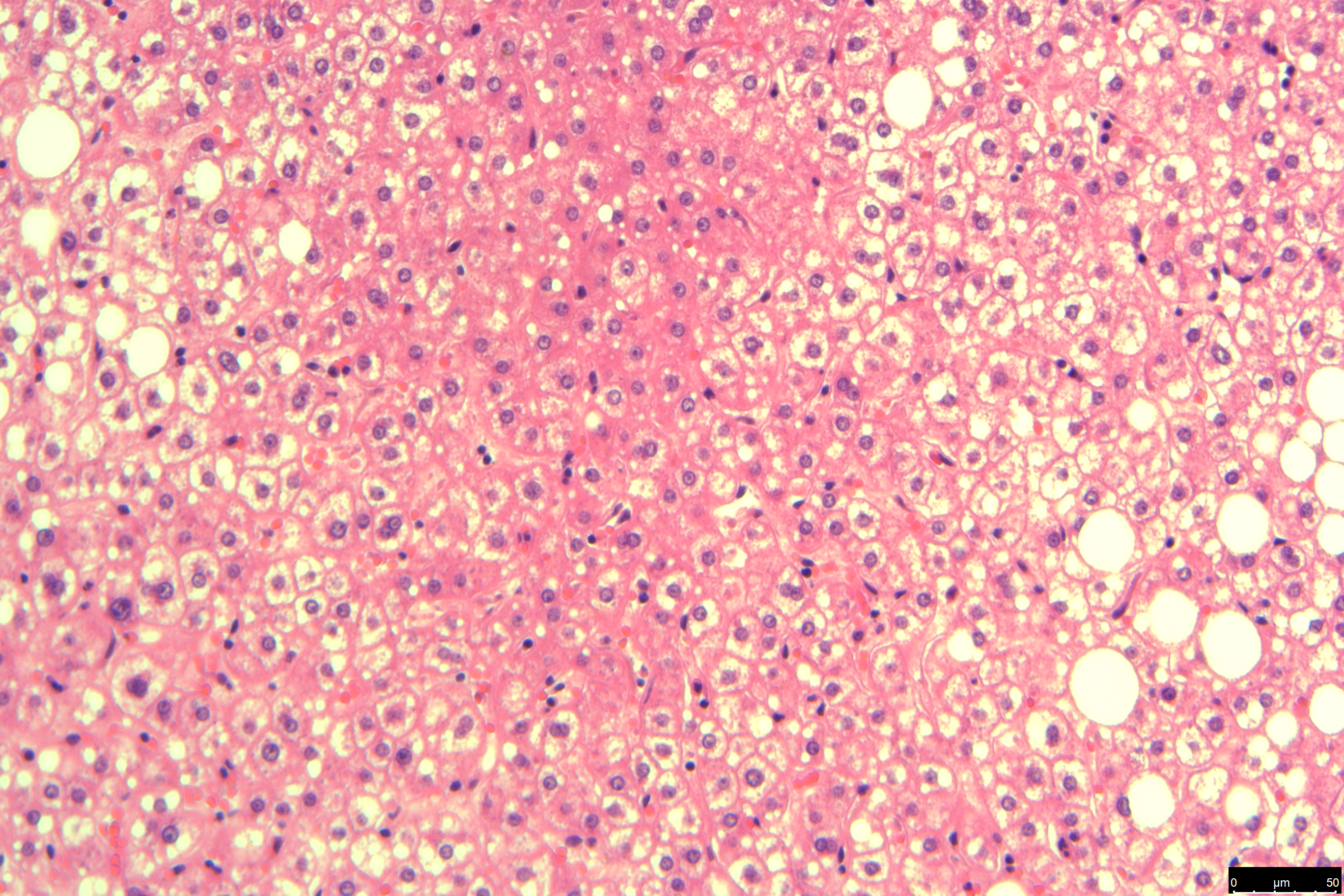

Supplement: Figure 4—source data 1. [file elife-70471-fig4-data1.zip › Figure 4-Source data/hepatocellular cancer patient 9/Raw data-HE staining image 1 of patient 9-20.0x.tif]

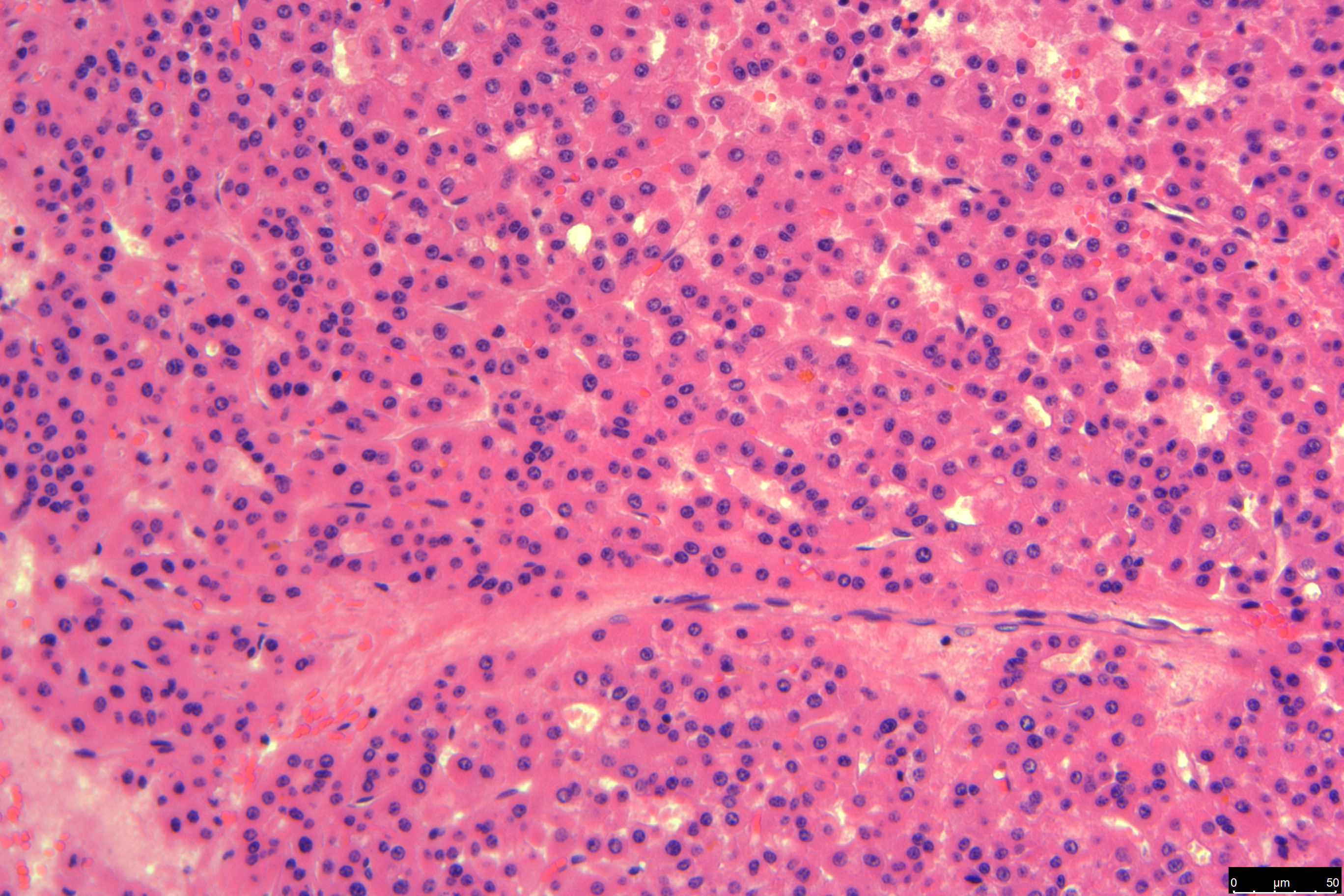

Supplement: Figure 4—source data 1. [file elife-70471-fig4-data1.zip › Figure 4-Source data/hepatocellular cancer patient 9/Raw data-HE staining image 2 of patient 9-20.0x.tif]

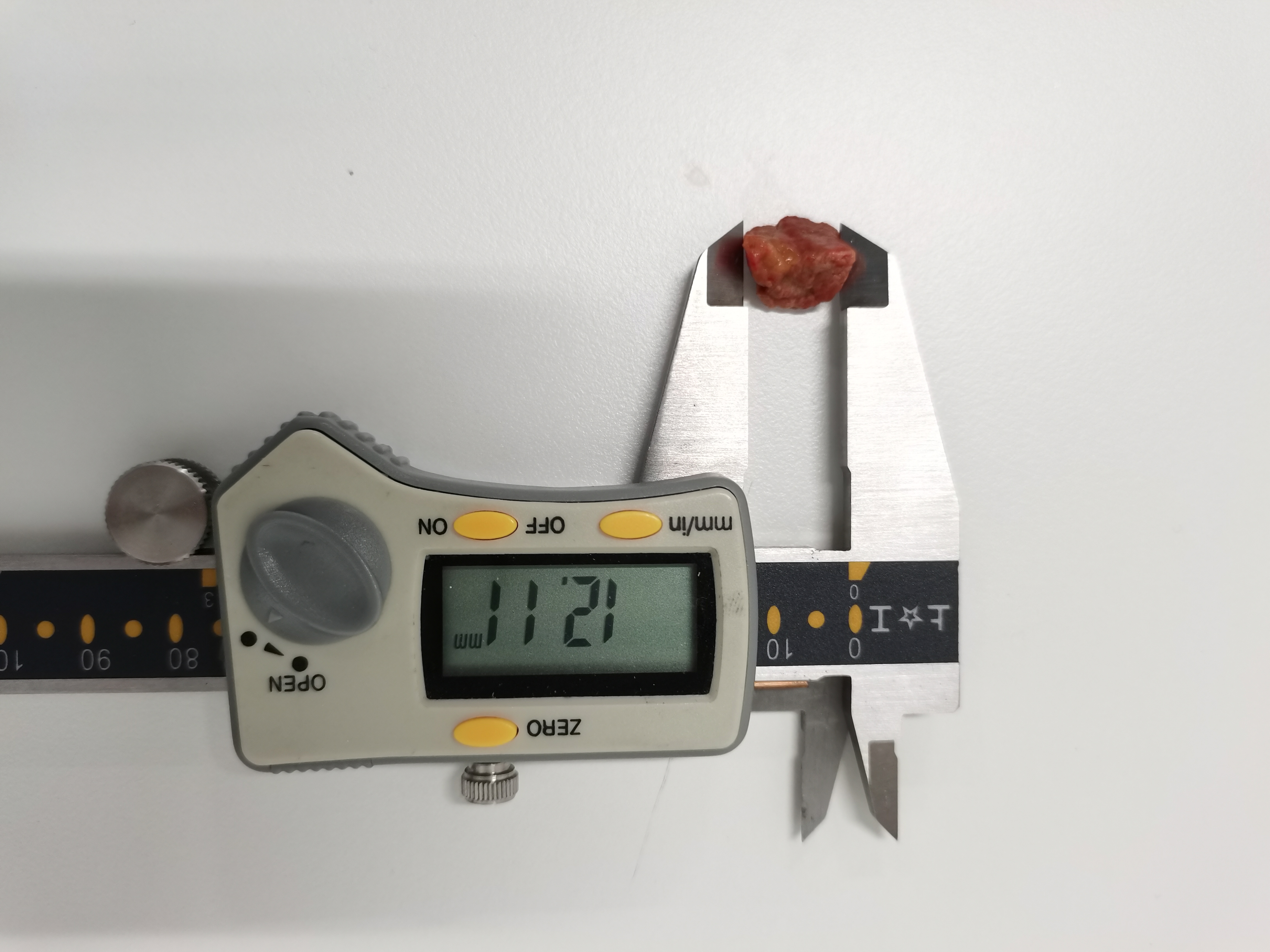

Supplement: Figure 4—source data 1. [file elife-70471-fig4-data1.zip › Figure 4-Source data/hepatocellular cancer patient 9/Raw data-photograph image 2.jpg]

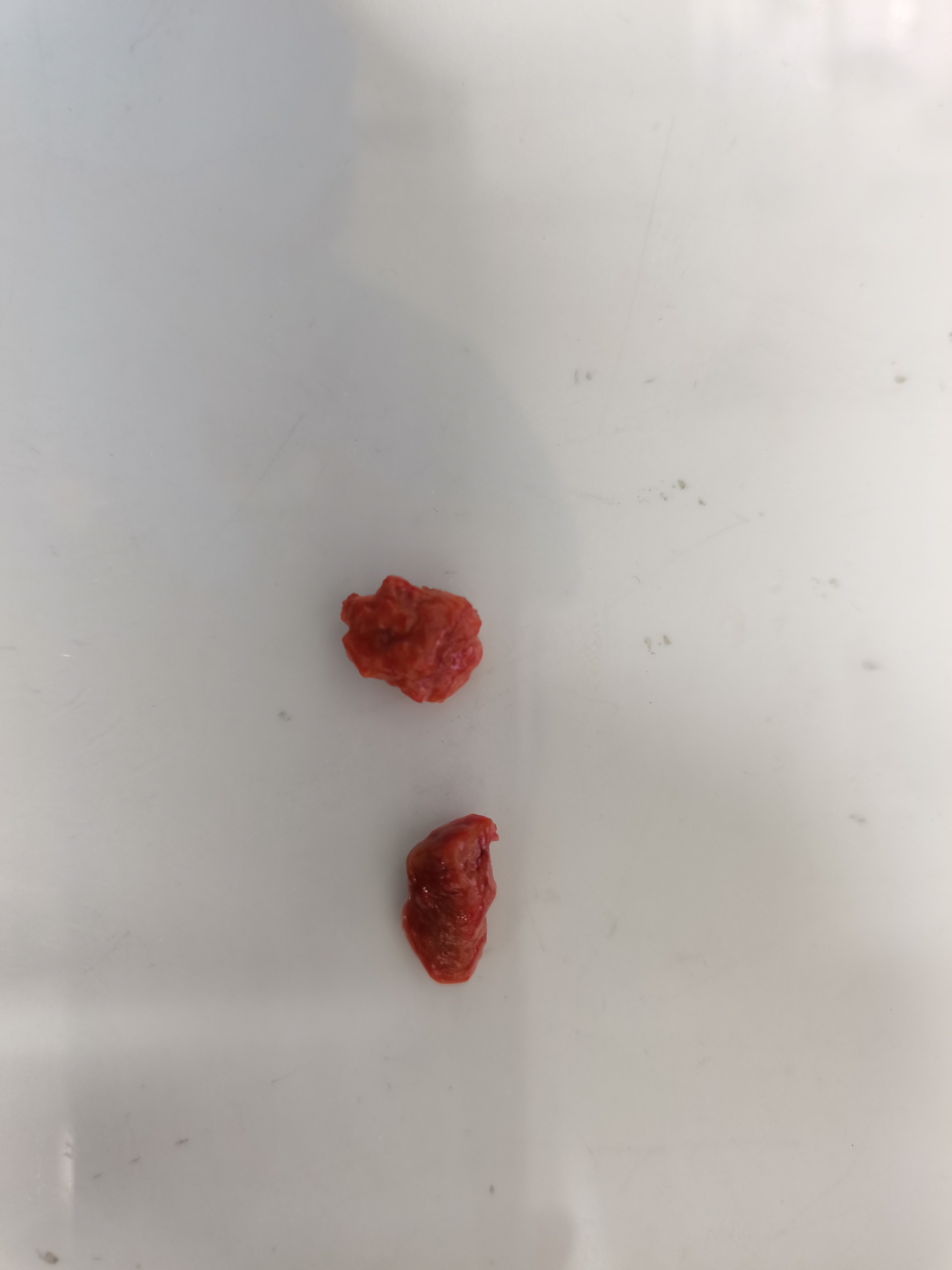

Supplement: Figure 4—source data 1. [file elife-70471-fig4-data1.zip › Figure 4-Source data/hepatocellular cancer patient 9/Raw data-photograph image 1.jpg]

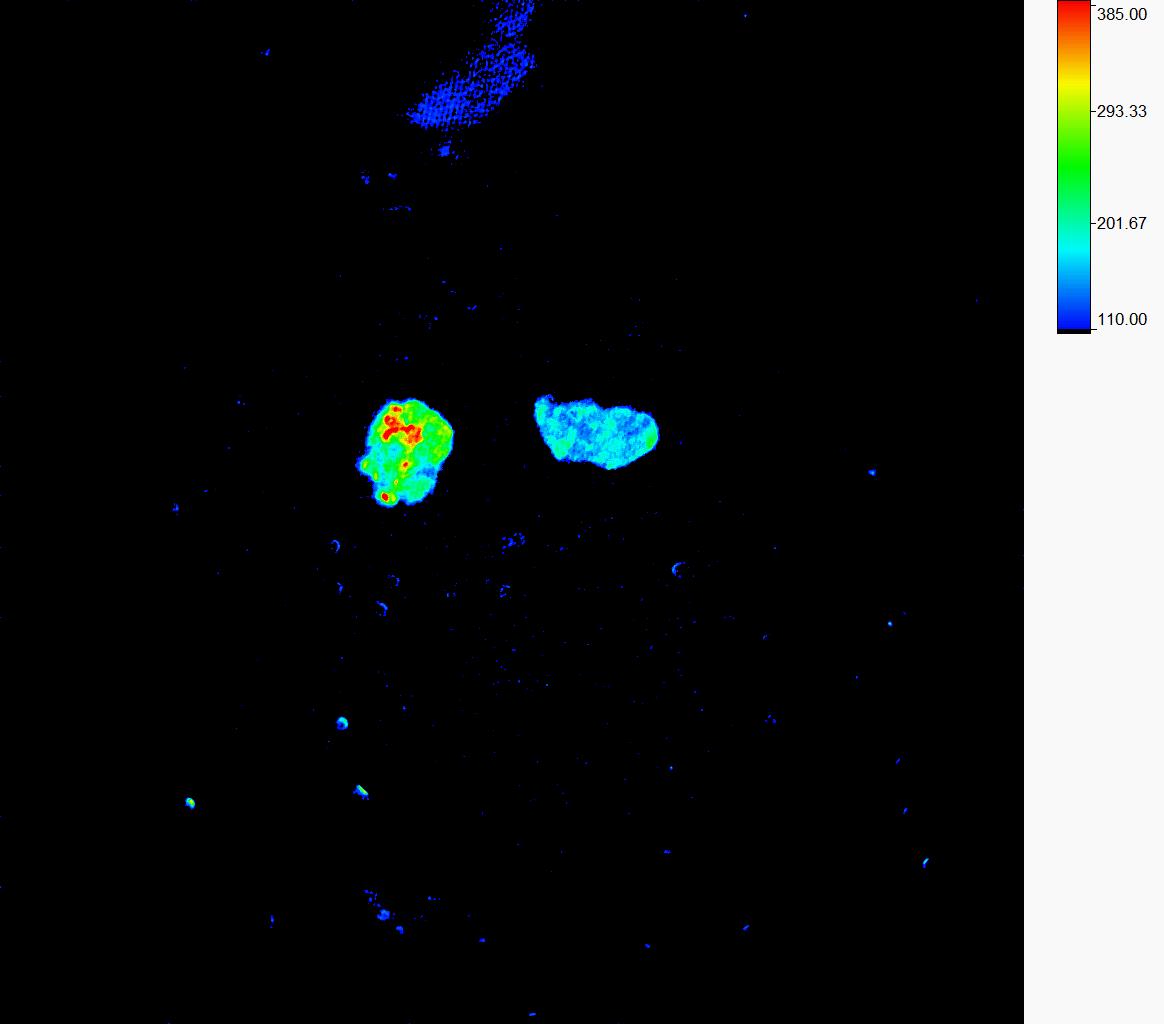

Supplement: Figure 4—source data 1. [file elife-70471-fig4-data1.zip › Figure 4-Source data/hepatocellular cancer patient 9/Raw data-nitroreductase detection image.jpg]

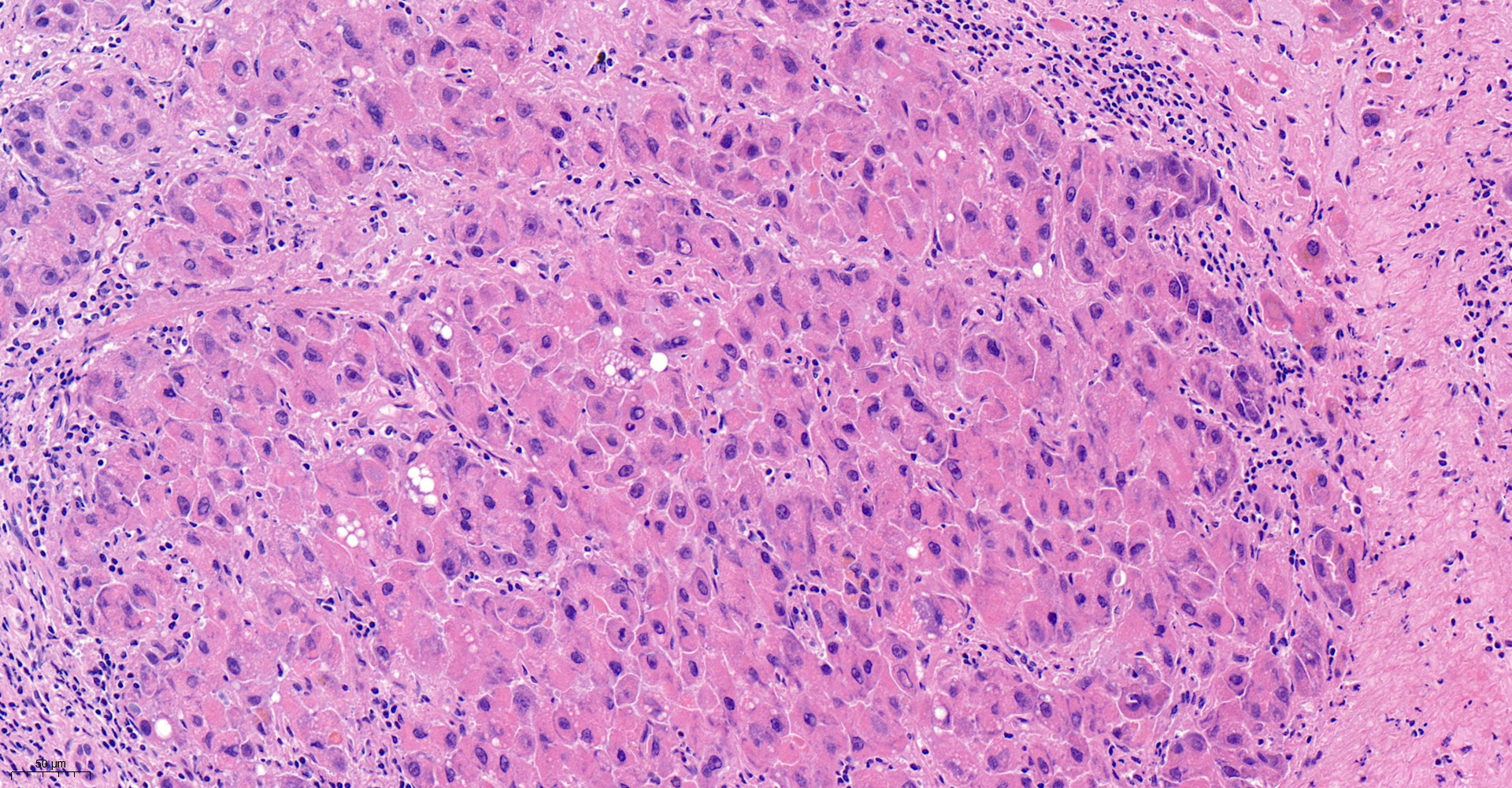

Supplement: Figure 4—source data 1. [file elife-70471-fig4-data1.zip › Figure 4-Source data/hepatocellular cancer patient 7/Raw data-HE staining image 2 of patient 7-20.0x.jpg]

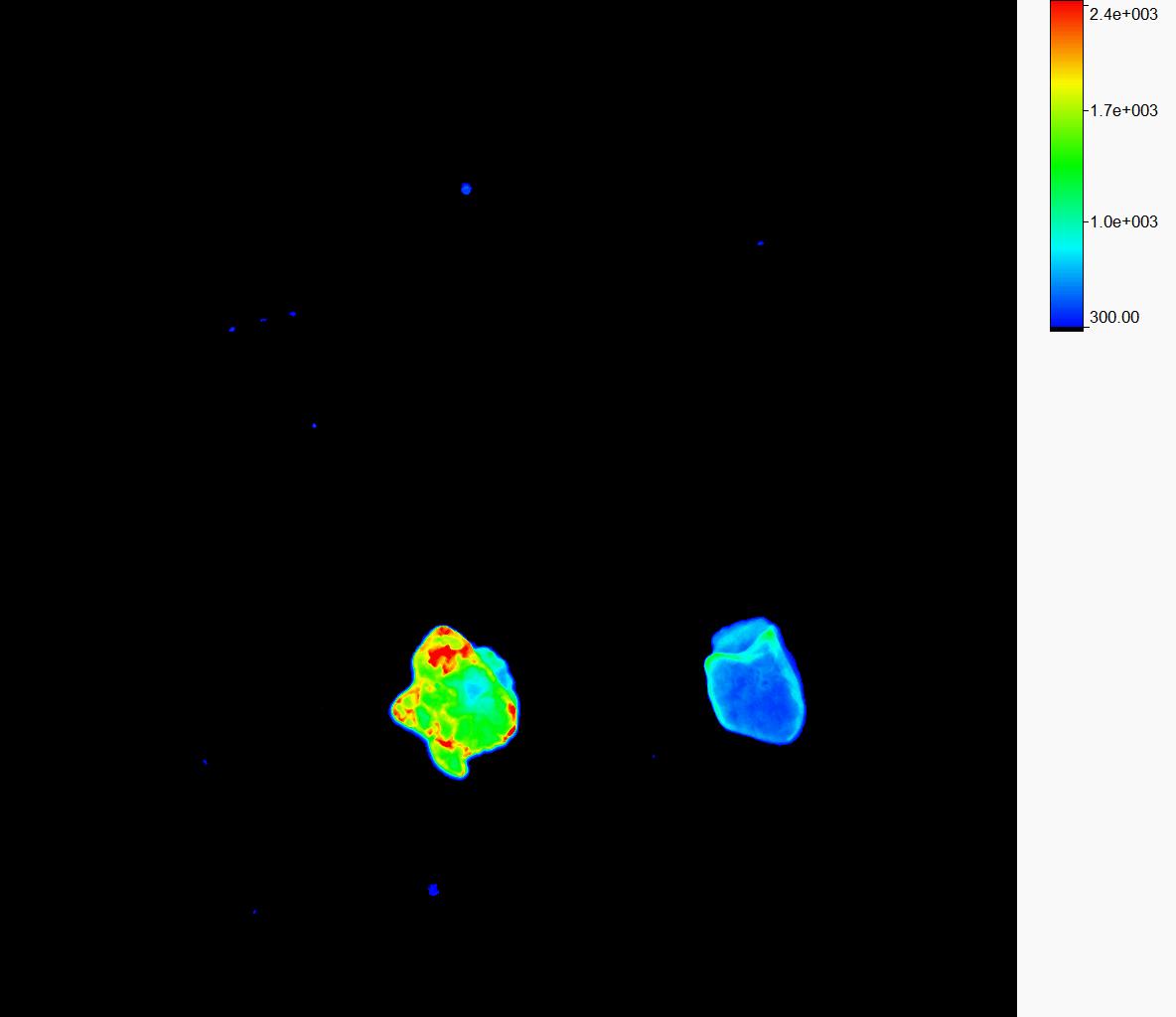

Supplement: Figure 4—source data 1. [file elife-70471-fig4-data1.zip › Figure 4-Source data/hepatocellular cancer patient 7/Raw data-viscosity detection image.jpg]

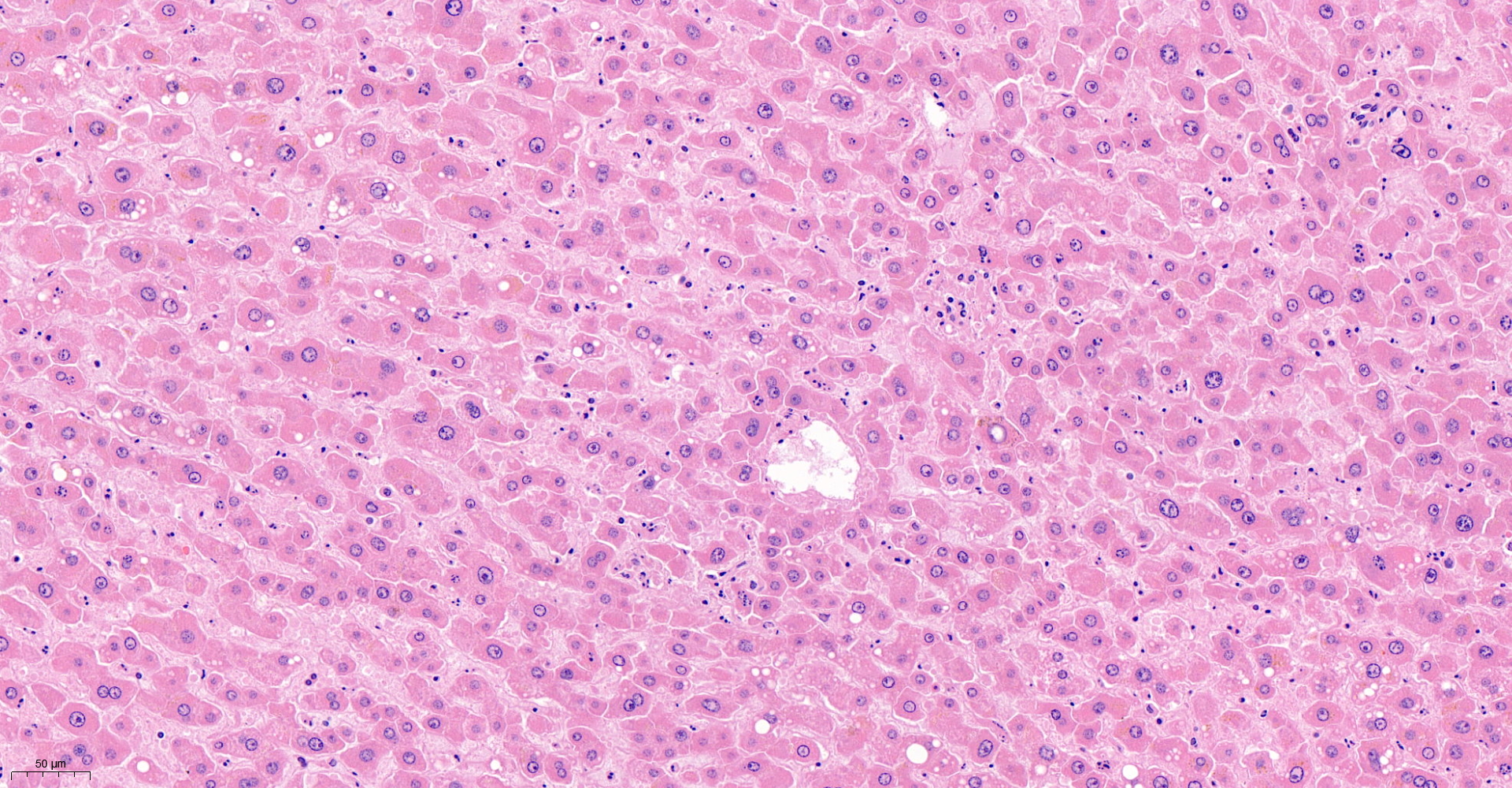

Supplement: Figure 4—source data 1. [file elife-70471-fig4-data1.zip › Figure 4-Source data/hepatocellular cancer patient 7/Raw data-HE staining image 1 of patient 7-20.0x.jpg]

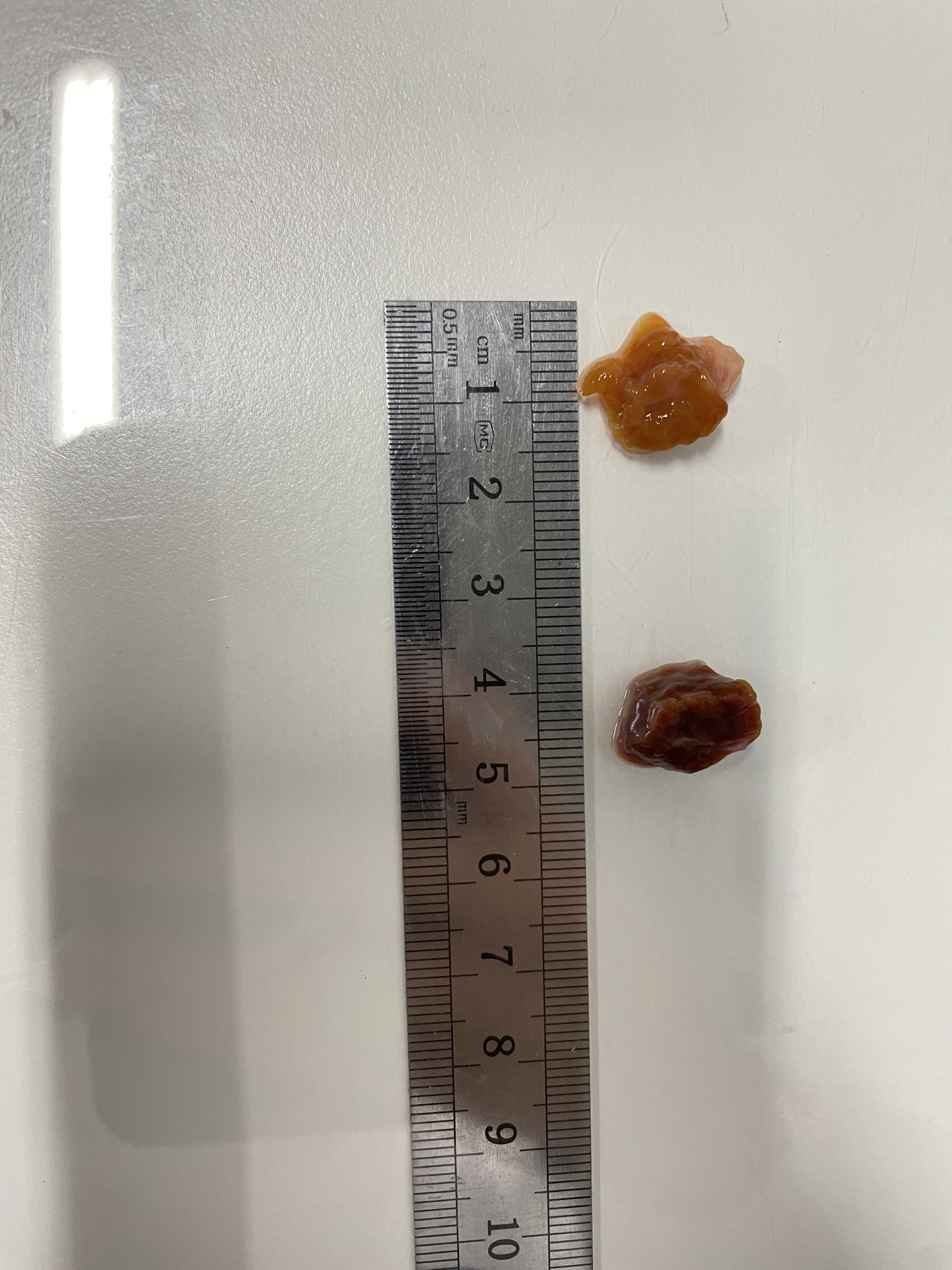

Supplement: Figure 4—source data 1. [file elife-70471-fig4-data1.zip › Figure 4-Source data/hepatocellular cancer patient 7/Raw data-photograph image.JPG]

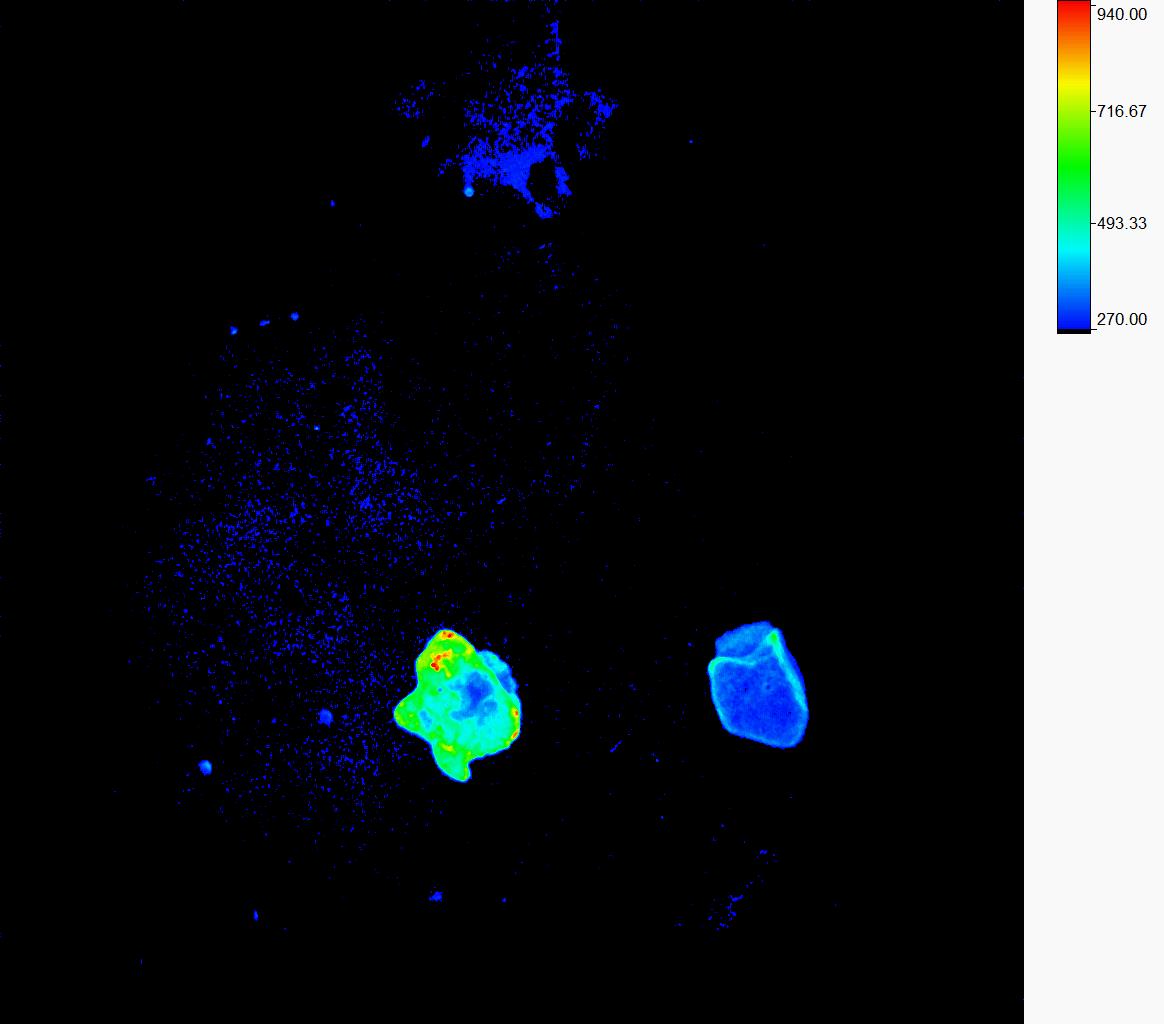

Supplement: Figure 4—source data 1. [file elife-70471-fig4-data1.zip › Figure 4-Source data/hepatocellular cancer patient 7/Raw data-nitroreductase detection image.jpg]

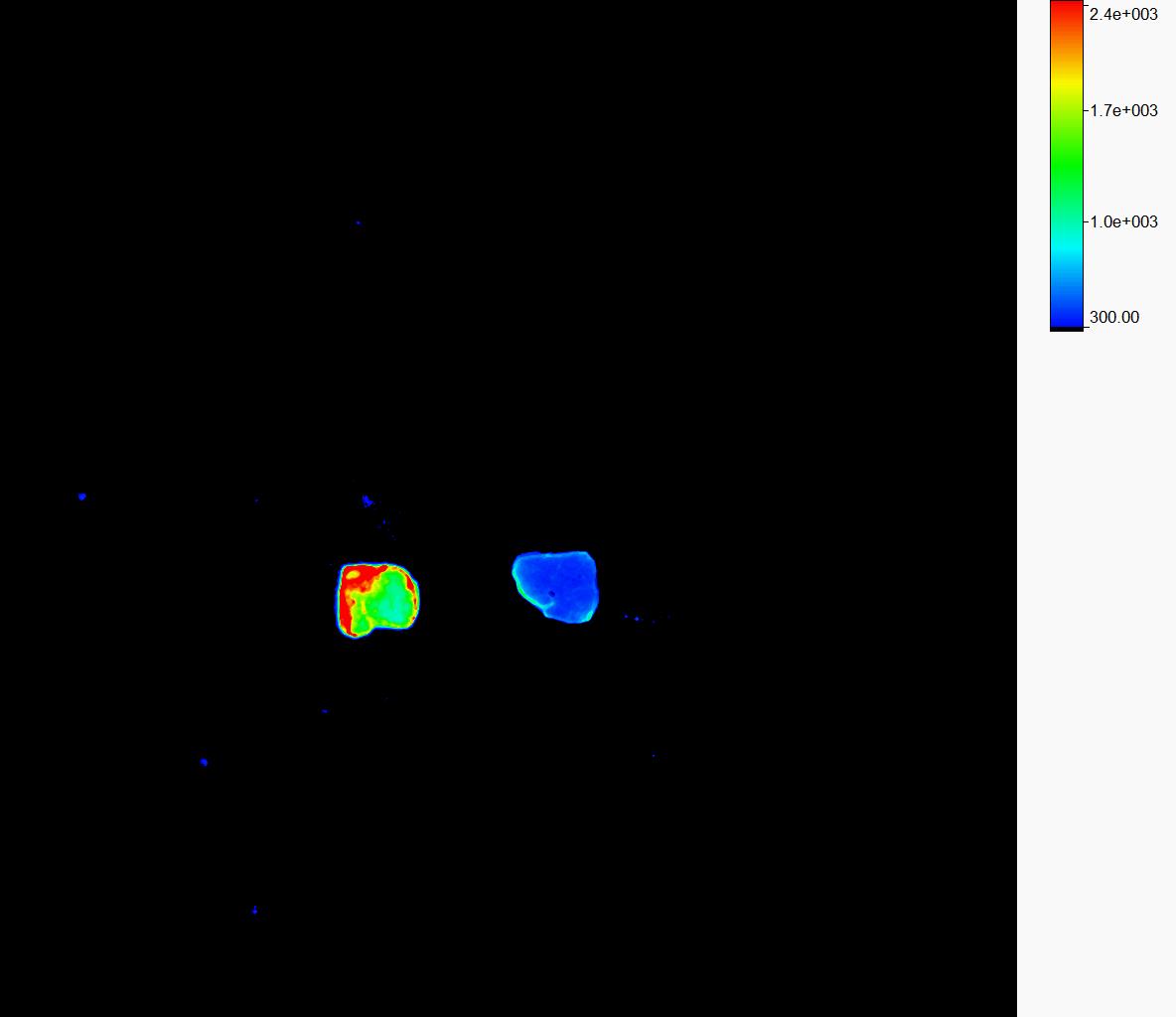

Supplement: Figure 4—source data 1. [file elife-70471-fig4-data1.zip › Figure 4-Source data/hepatocellular cancer patient 5/Raw data-viscosity detection image.jpg]

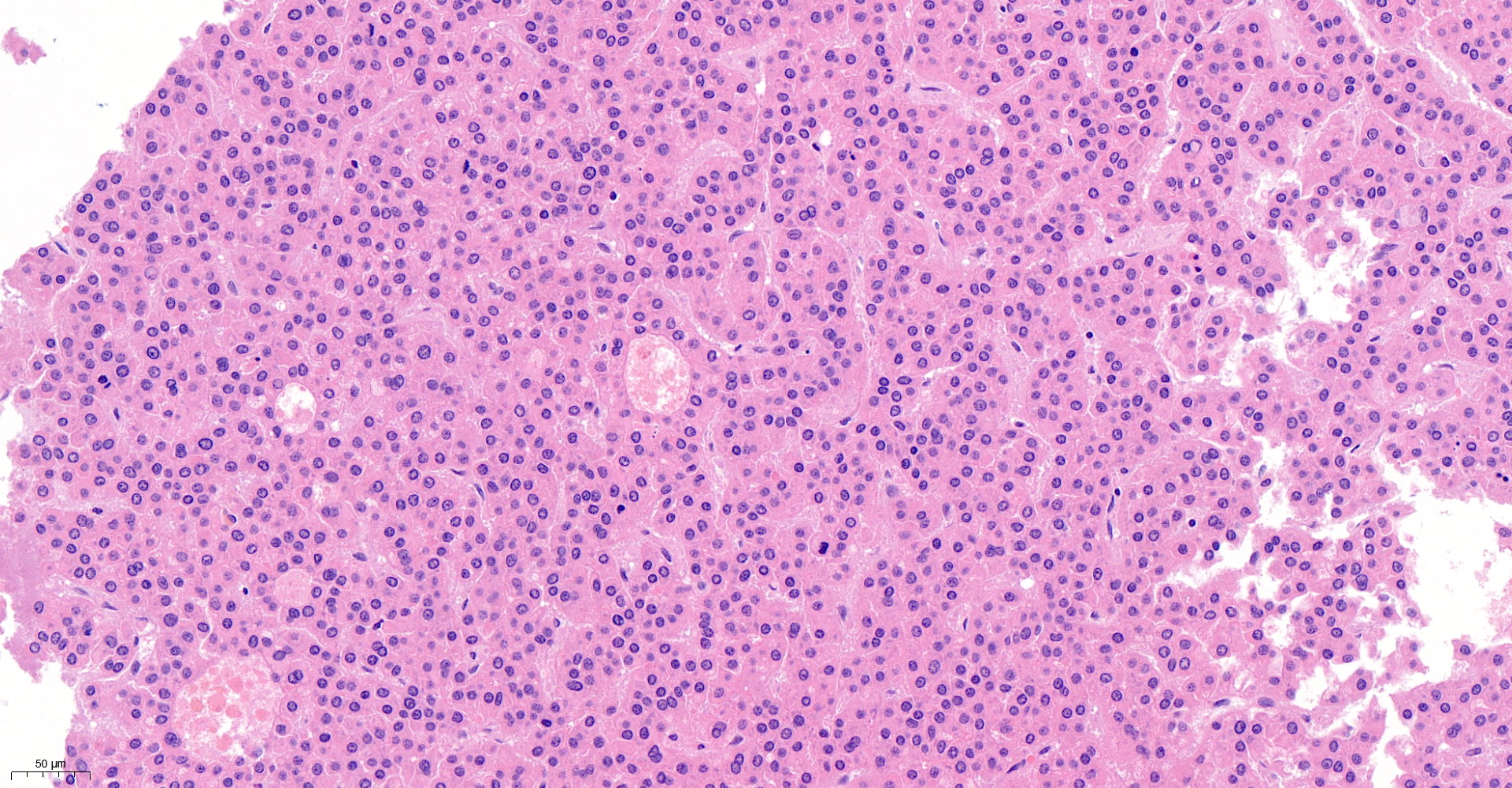

Supplement: Figure 4—source data 1. [file elife-70471-fig4-data1.zip › Figure 4-Source data/hepatocellular cancer patient 5/Raw data-HE staining image 2 of patient 5-20.0x.jpg]

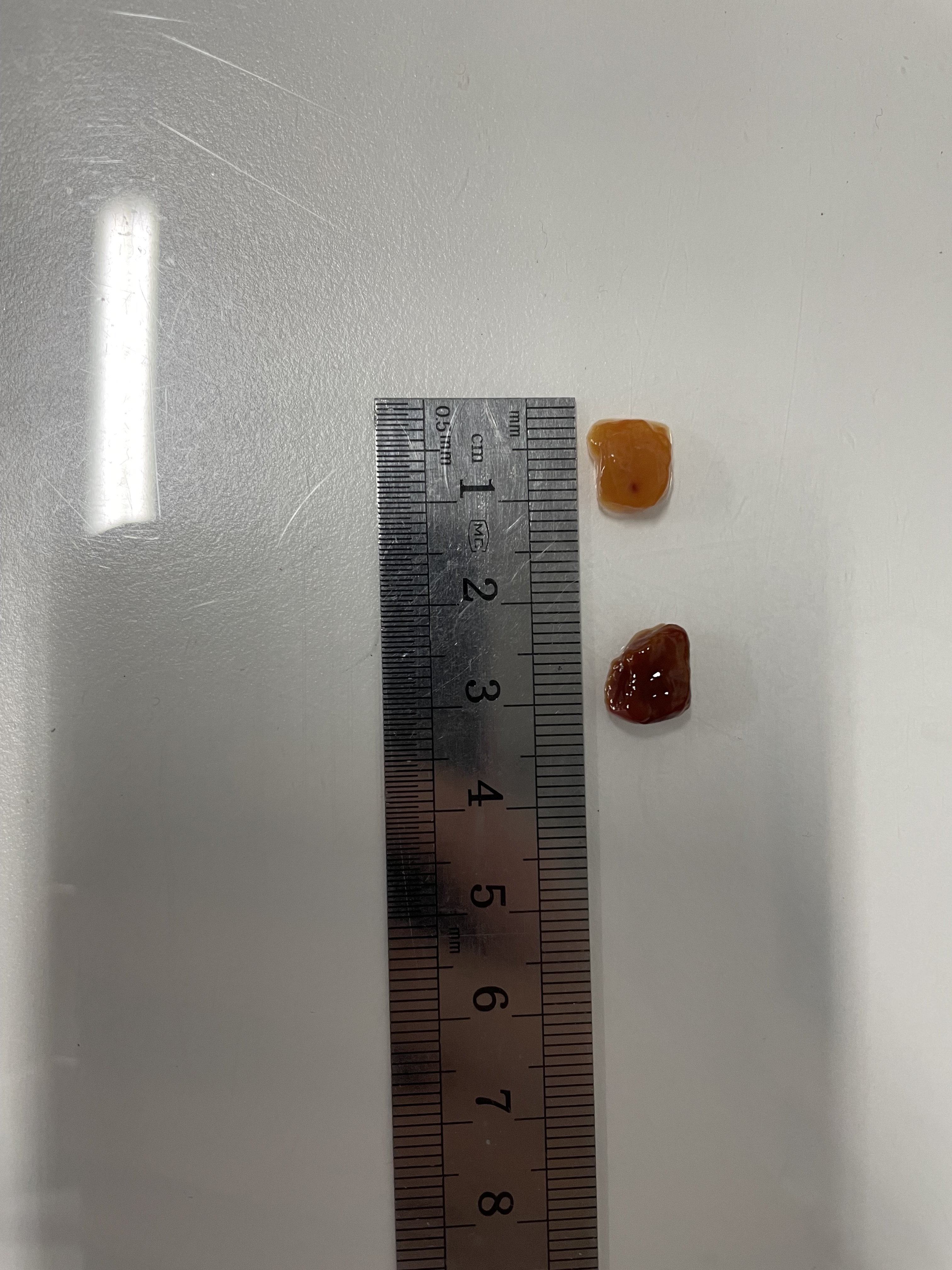

Supplement: Figure 4—source data 1. [file elife-70471-fig4-data1.zip › Figure 4-Source data/hepatocellular cancer patient 5/Raw data-photograph image.JPG]

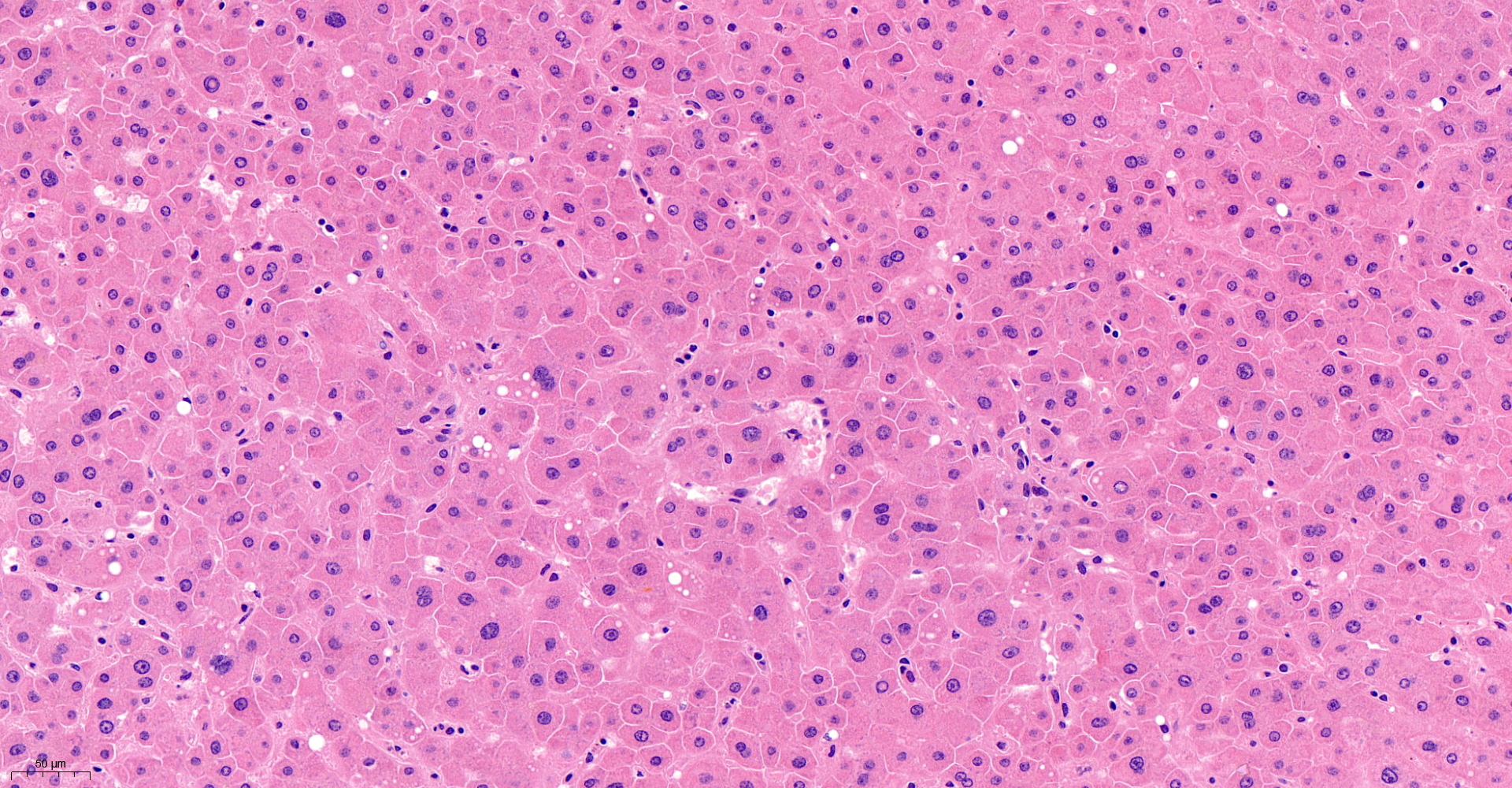

Supplement: Figure 4—source data 1. [file elife-70471-fig4-data1.zip › Figure 4-Source data/hepatocellular cancer patient 5/Raw data-HE staining image 1 of patient 5-20.0x.jpg]

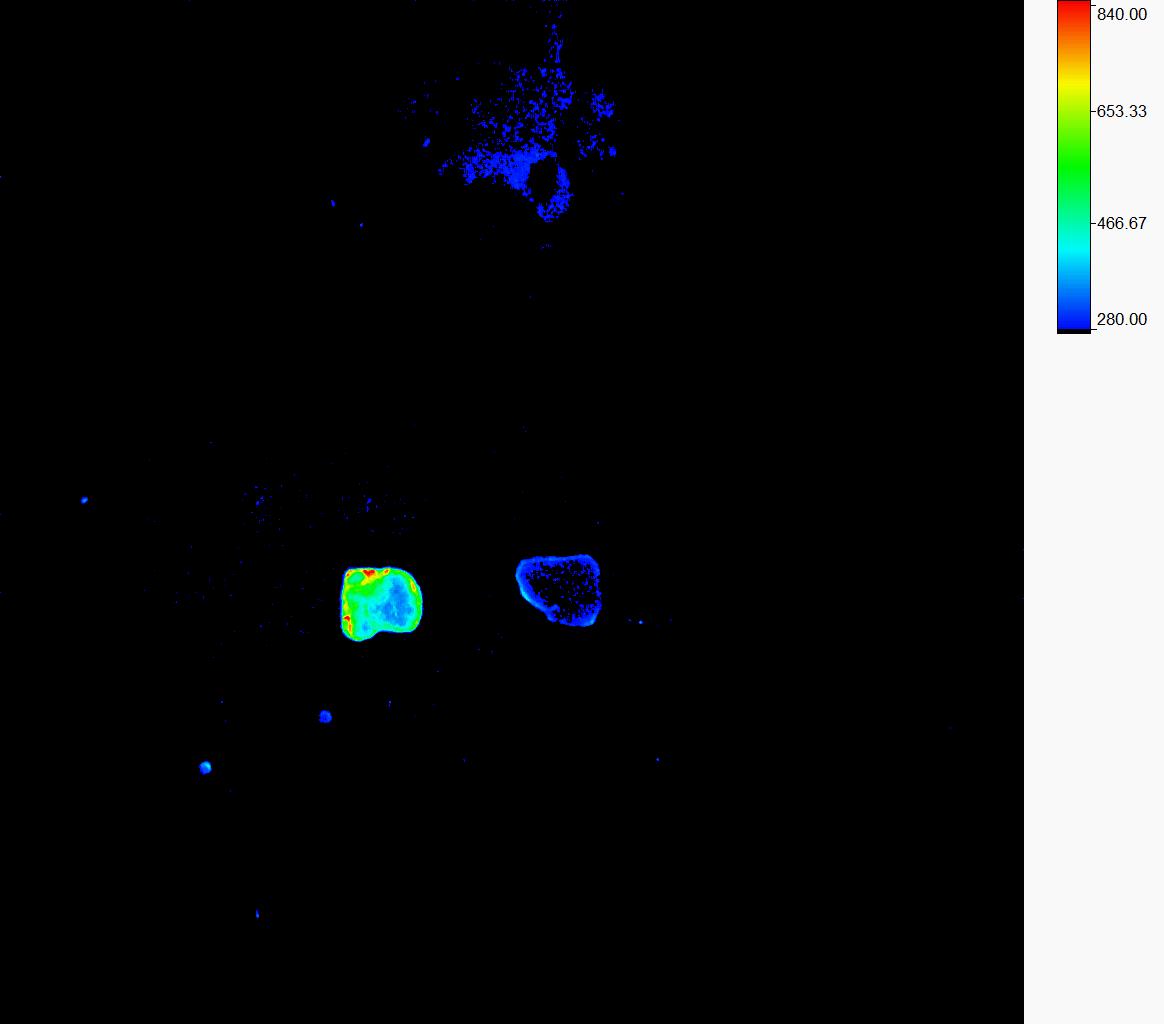

Supplement: Figure 4—source data 1. [file elife-70471-fig4-data1.zip › Figure 4-Source data/hepatocellular cancer patient 5/Raw data-nitroreductase detection image.jpg]

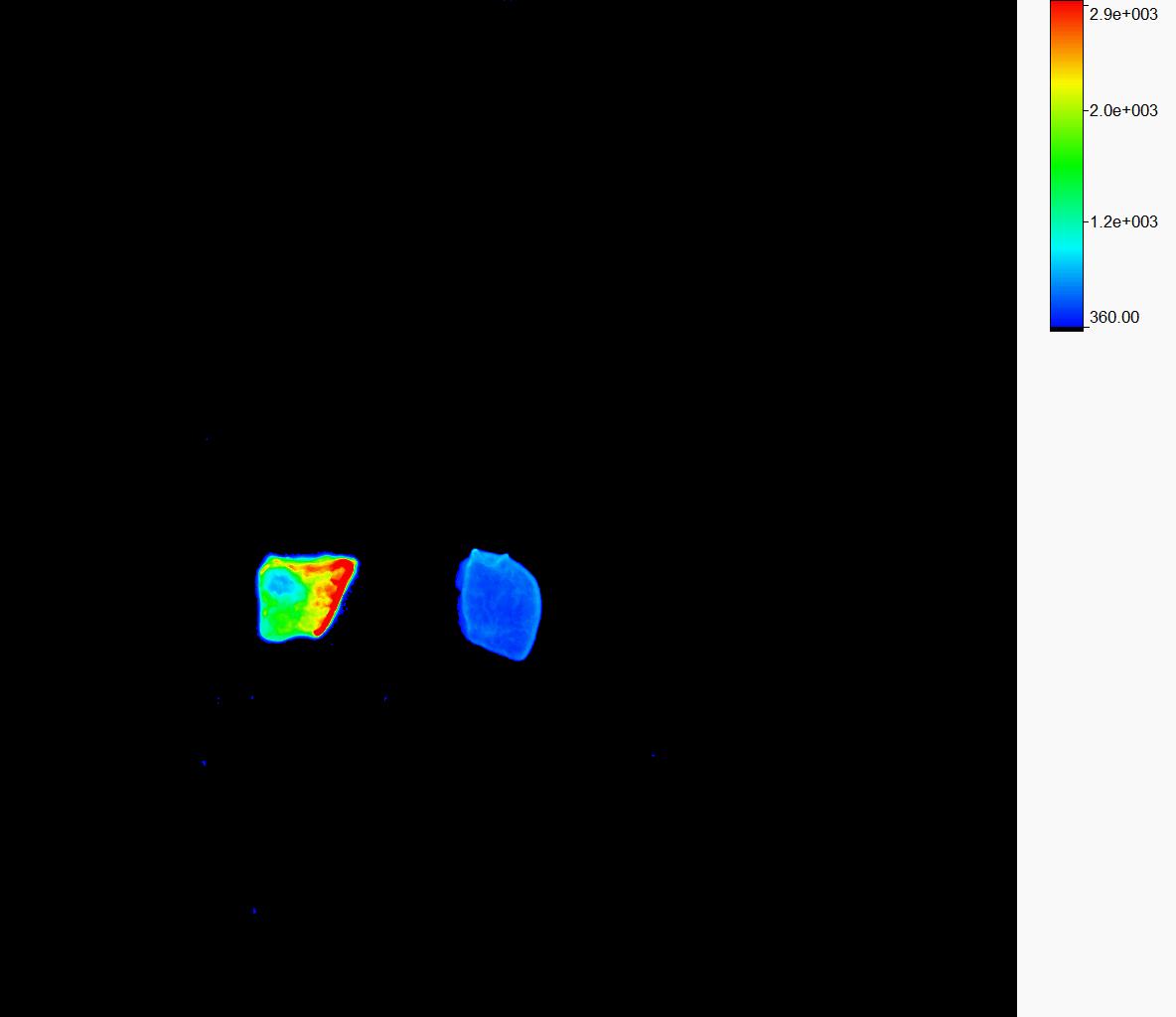

Supplement: Figure 4—source data 1. [file elife-70471-fig4-data1.zip › Figure 4-Source data/hepatocellular cancer patient 2/Raw data-viscosity detection image.jpg]

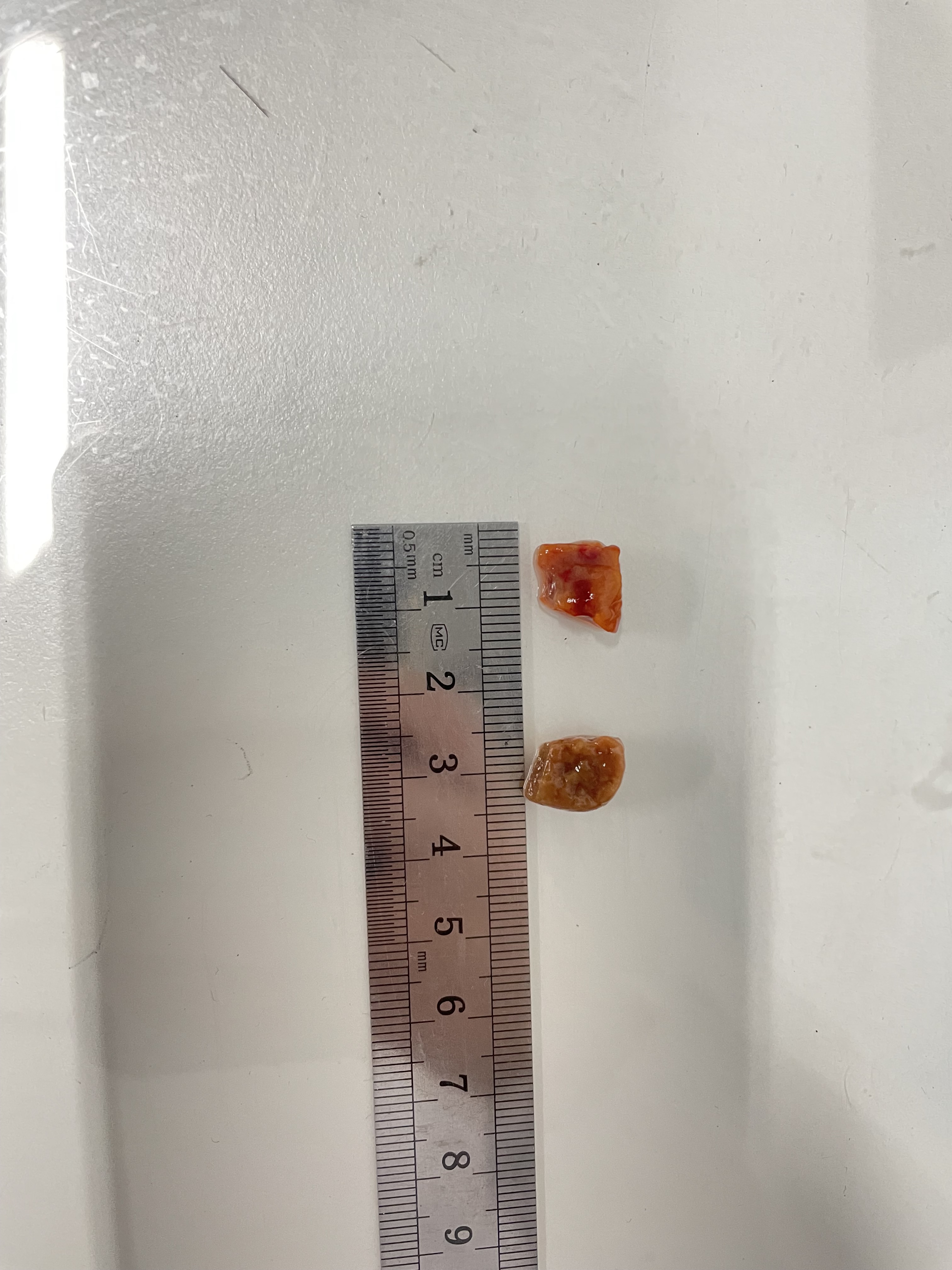

Supplement: Figure 4—source data 1. [file elife-70471-fig4-data1.zip › Figure 4-Source data/hepatocellular cancer patient 2/Raw data-photograph image.JPG]

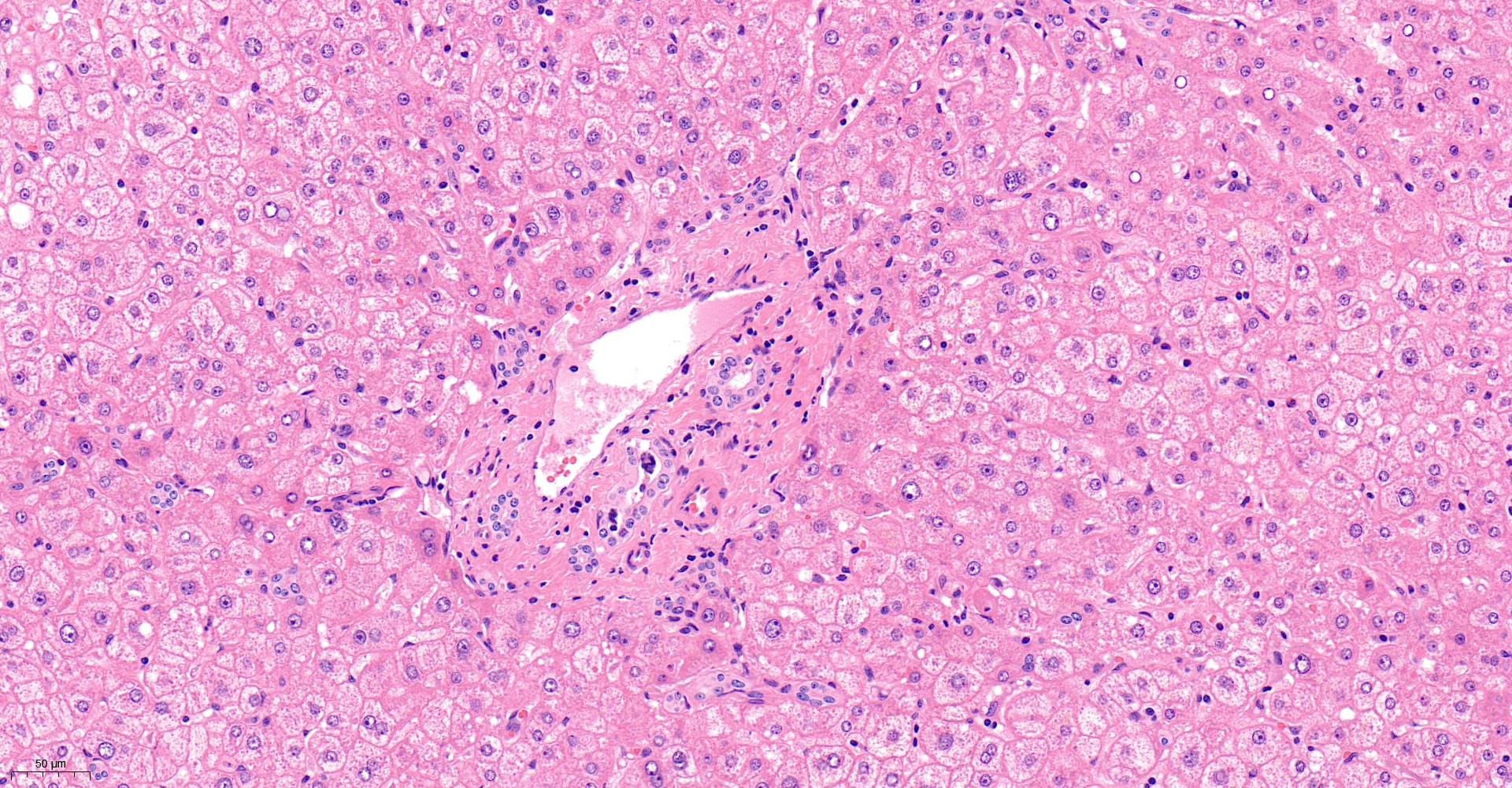

Supplement: Figure 4—source data 1. [file elife-70471-fig4-data1.zip › Figure 4-Source data/hepatocellular cancer patient 2/Raw data-HE staining image 1 of patient 2-20.0x.jpg]

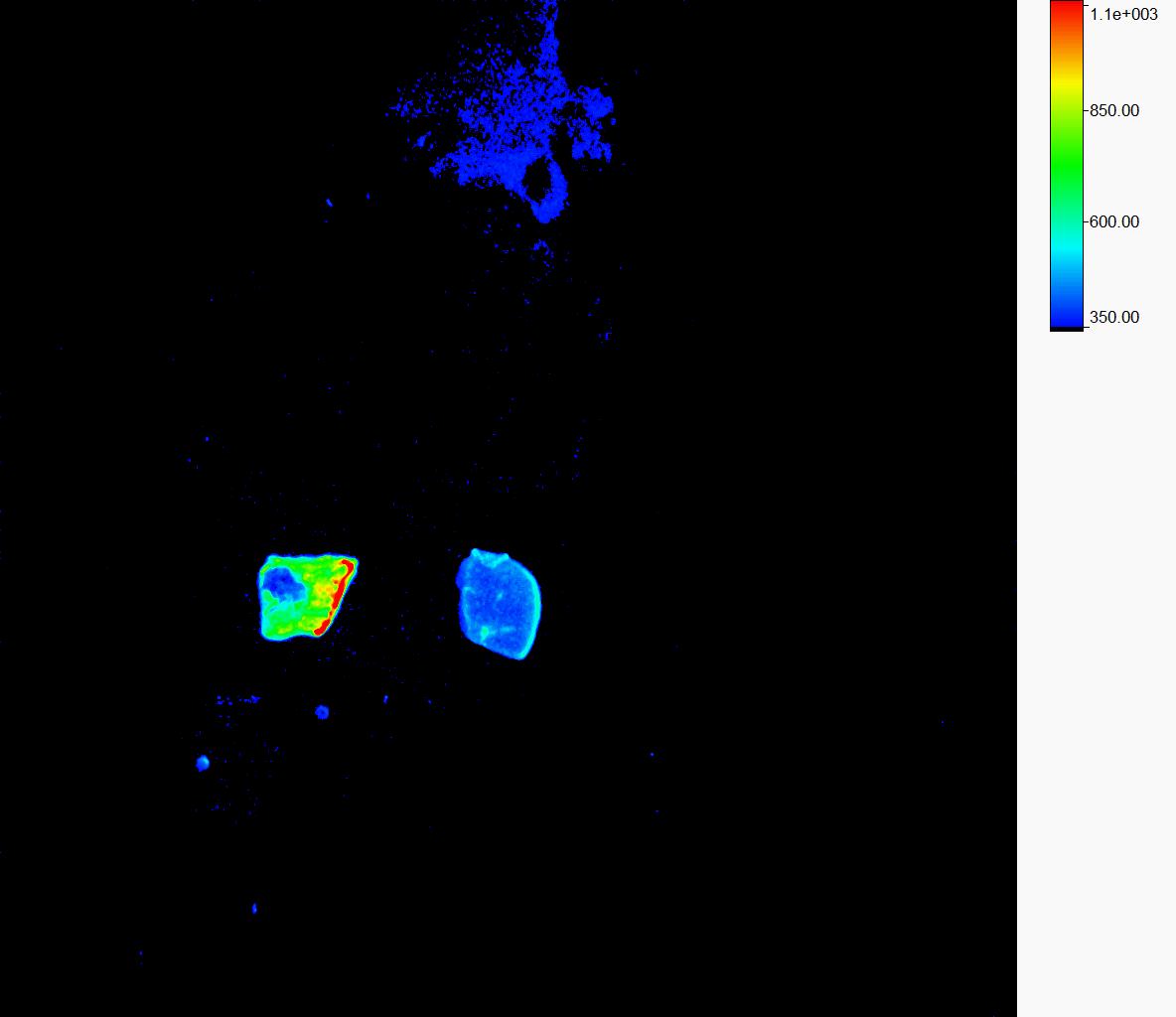

Supplement: Figure 4—source data 1. [file elife-70471-fig4-data1.zip › Figure 4-Source data/hepatocellular cancer patient 2/Raw data-nitroreductase detection image.jpg]

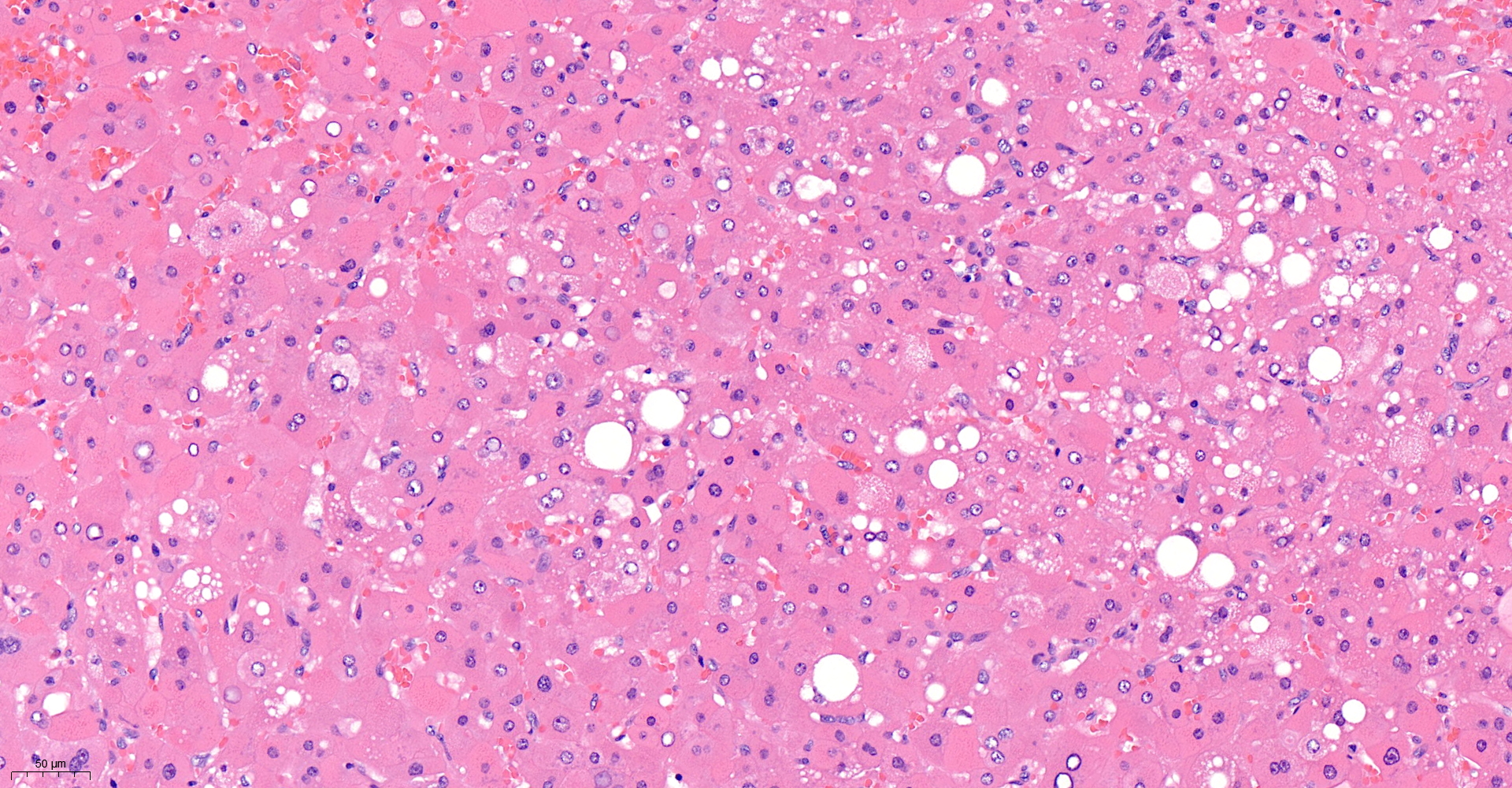

Supplement: Figure 4—source data 1. [file elife-70471-fig4-data1.zip › Figure 4-Source data/hepatocellular cancer patient 2/Raw data-HE staining image 2 of patient 2-20.0x.jpg]

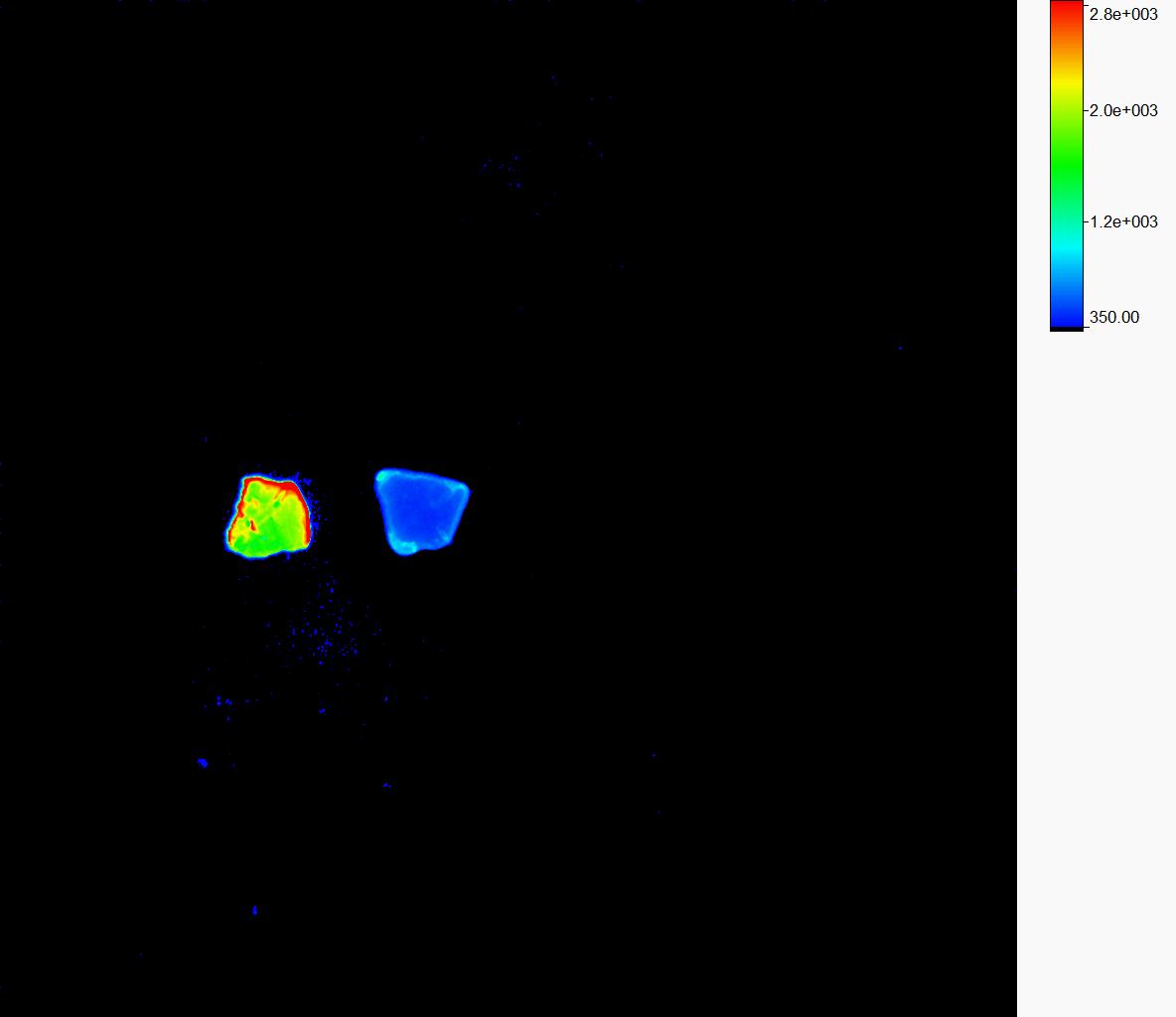

Supplement: Figure 4—source data 1. [file elife-70471-fig4-data1.zip › Figure 4-Source data/hepatocellular cancer patient 3/Raw data-viscosity detection image.jpg]

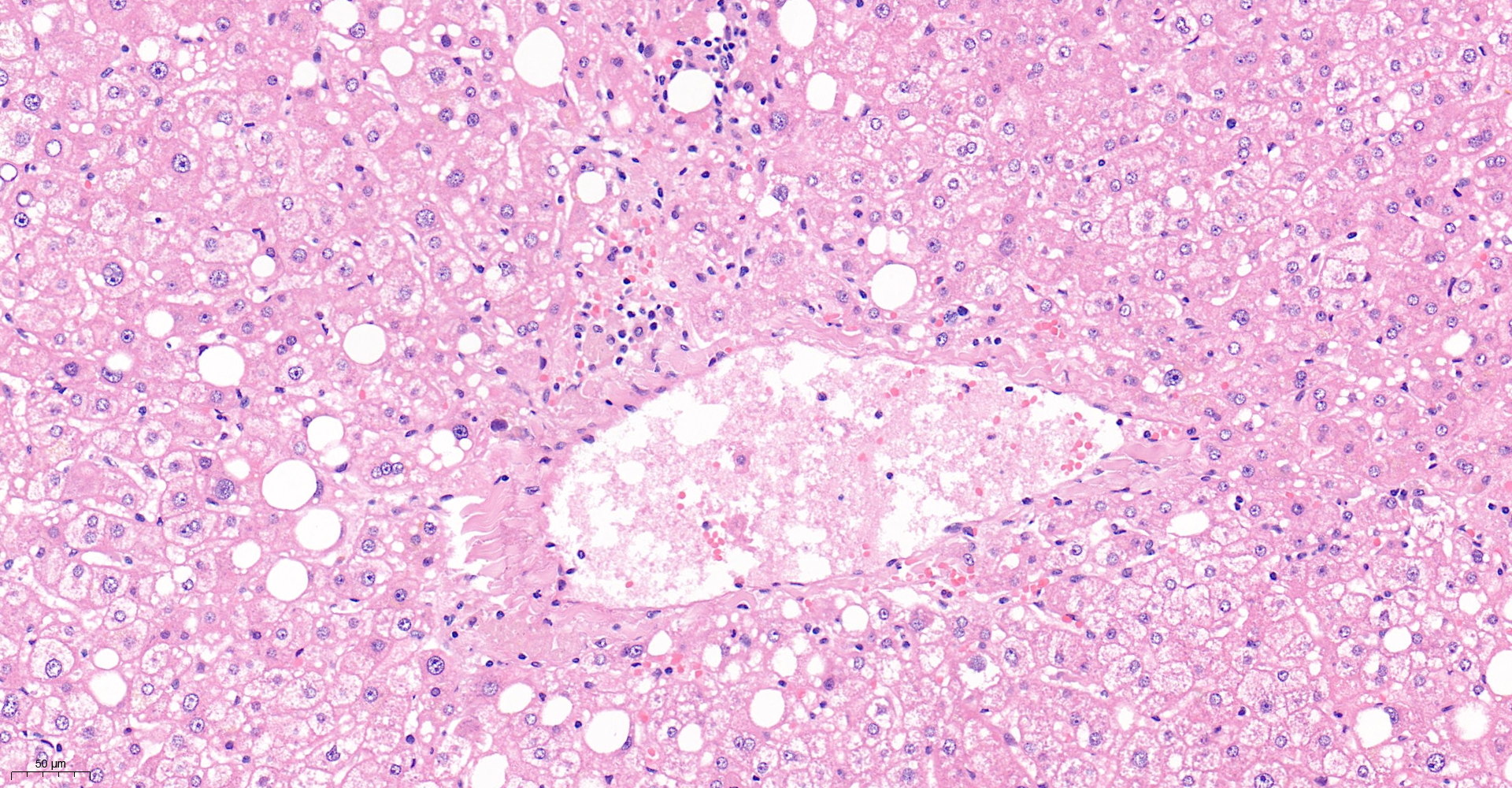

Supplement: Figure 4—source data 1. [file elife-70471-fig4-data1.zip › Figure 4-Source data/hepatocellular cancer patient 3/Raw data-HE staining image 1 of patient 3-20.0x.jpg]

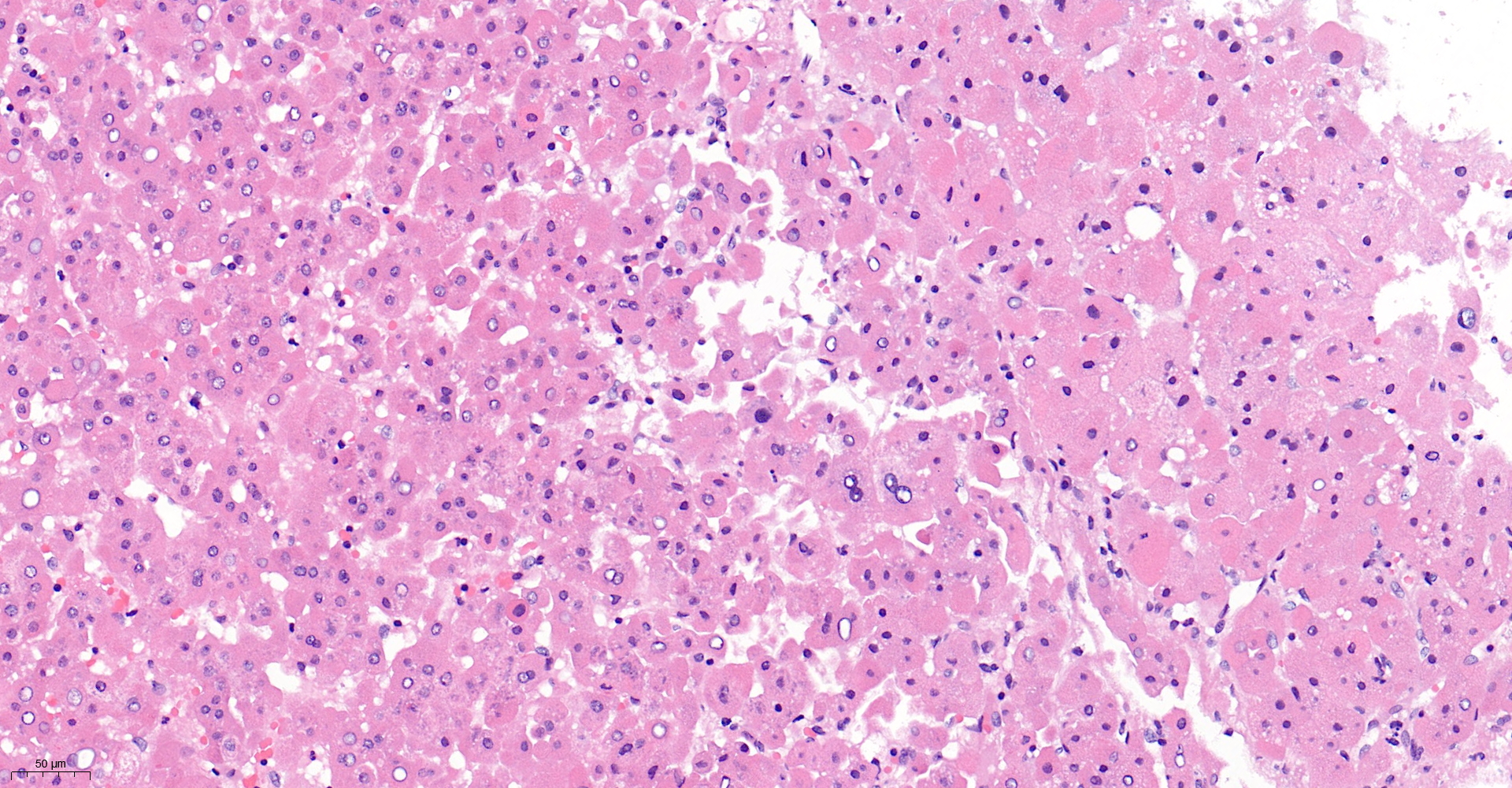

Supplement: Figure 4—source data 1. [file elife-70471-fig4-data1.zip › Figure 4-Source data/hepatocellular cancer patient 3/Raw data-HE staining image 2 of patient 3-20.0x.jpg]

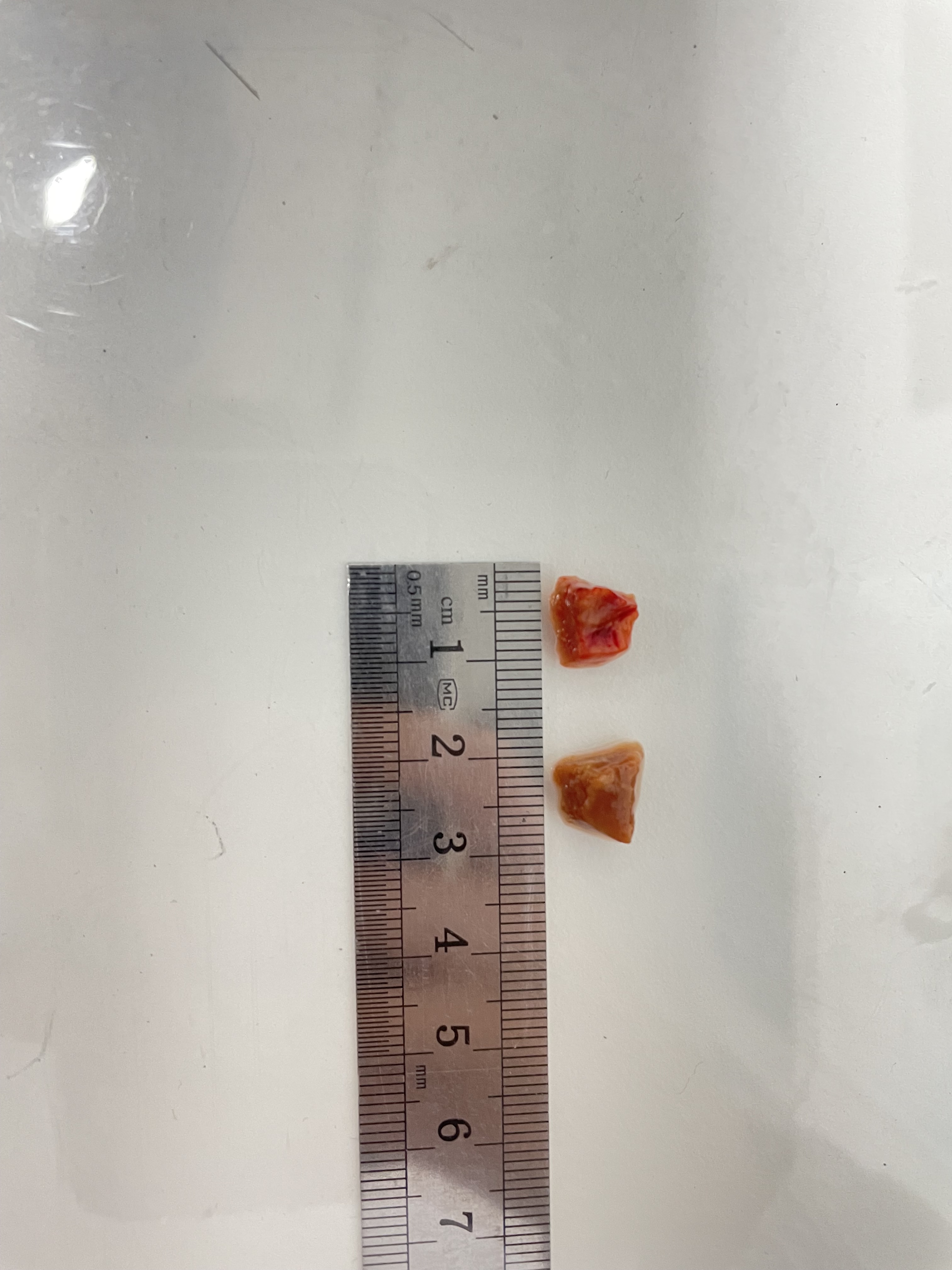

Supplement: Figure 4—source data 1. [file elife-70471-fig4-data1.zip › Figure 4-Source data/hepatocellular cancer patient 3/Raw data-photograph image.JPG]

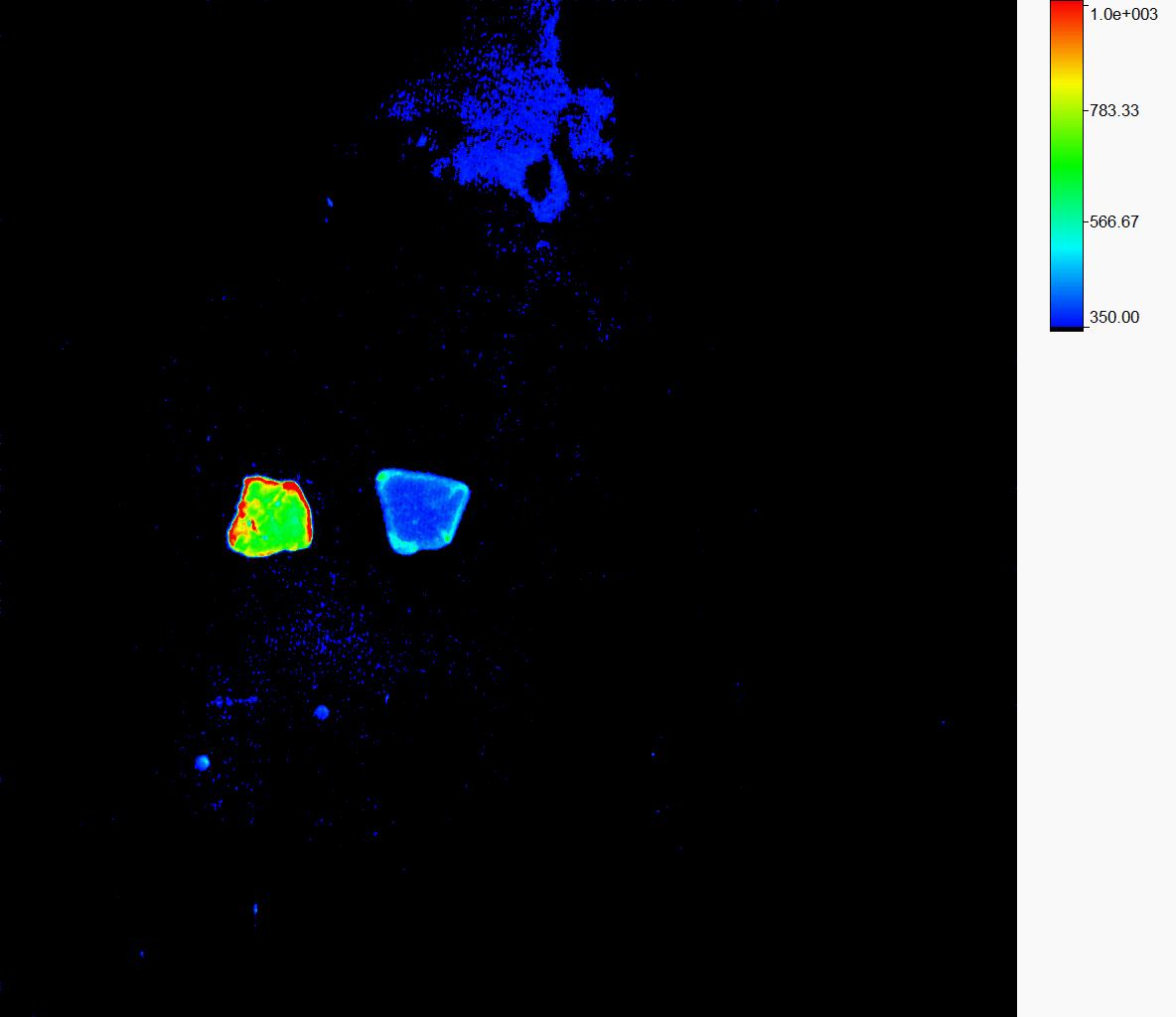

Supplement: Figure 4—source data 1. [file elife-70471-fig4-data1.zip › Figure 4-Source data/hepatocellular cancer patient 3/Raw data-nitroreductase detection image.jpg]

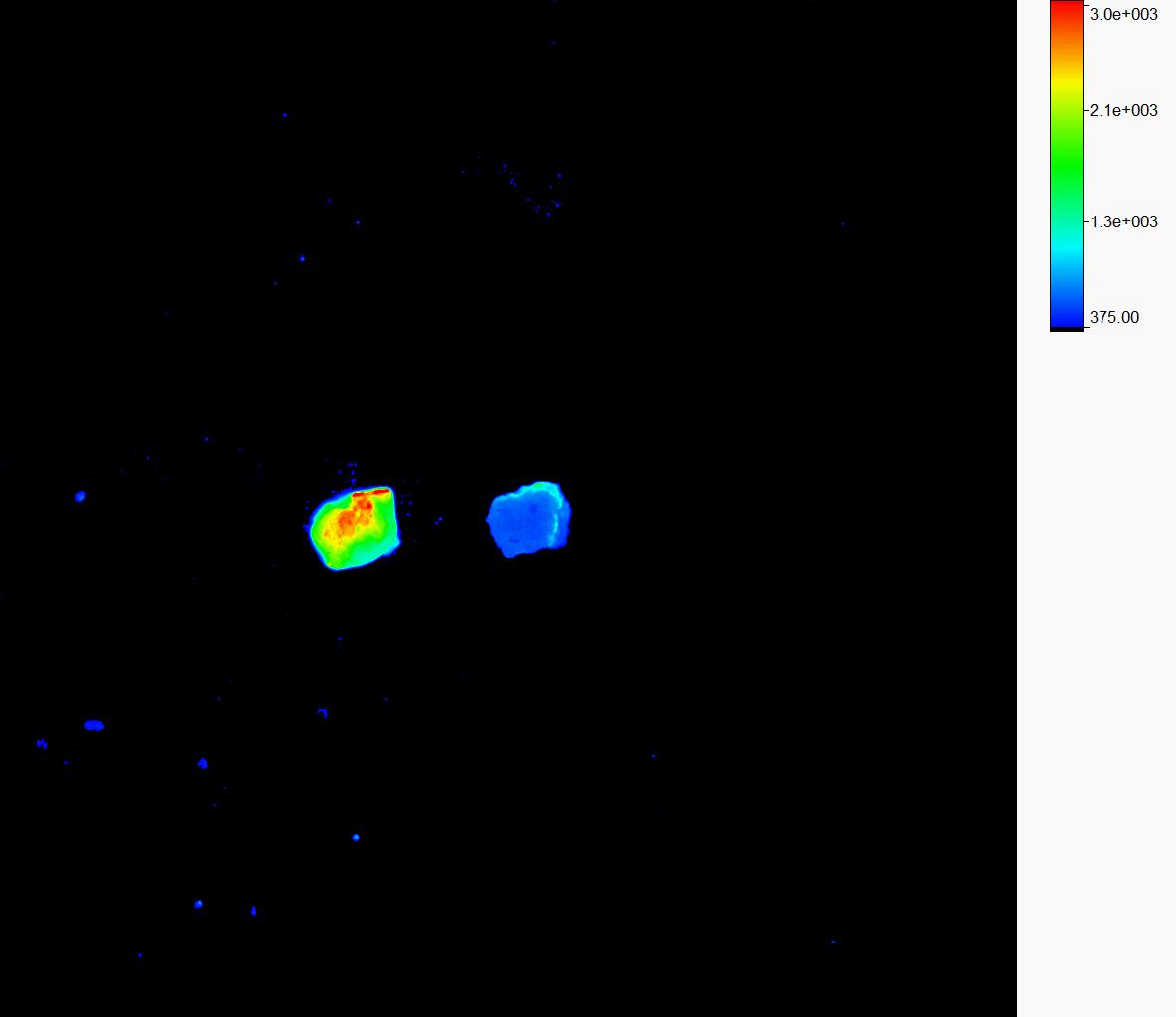

Supplement: Figure 4—source data 1. [file elife-70471-fig4-data1.zip › Figure 4-Source data/hepatocellular cancer patient 4/Raw data-viscosity detection image.jpg]

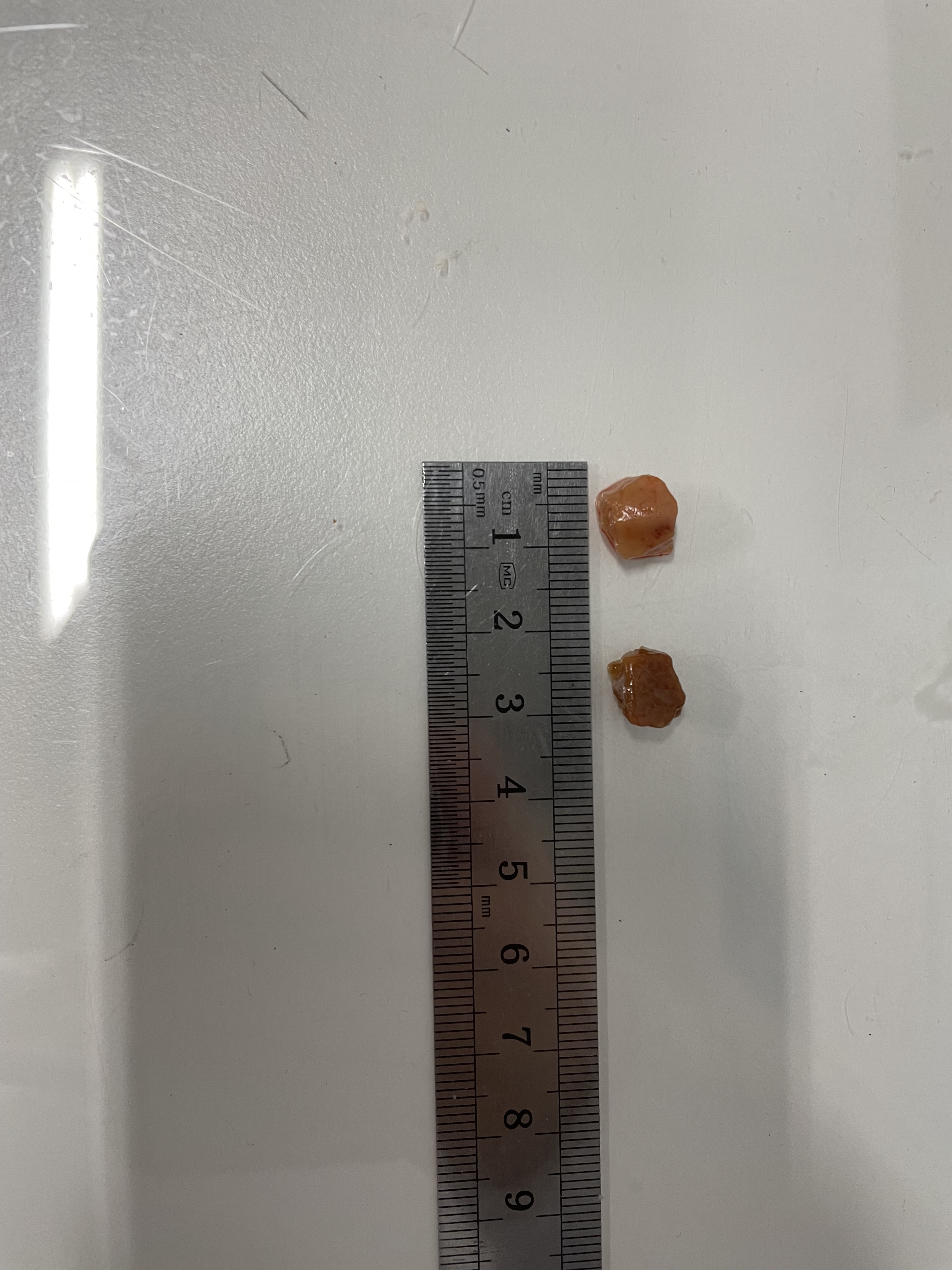

Supplement: Figure 4—source data 1. [file elife-70471-fig4-data1.zip › Figure 4-Source data/hepatocellular cancer patient 4/Raw data-photograph image.JPG]

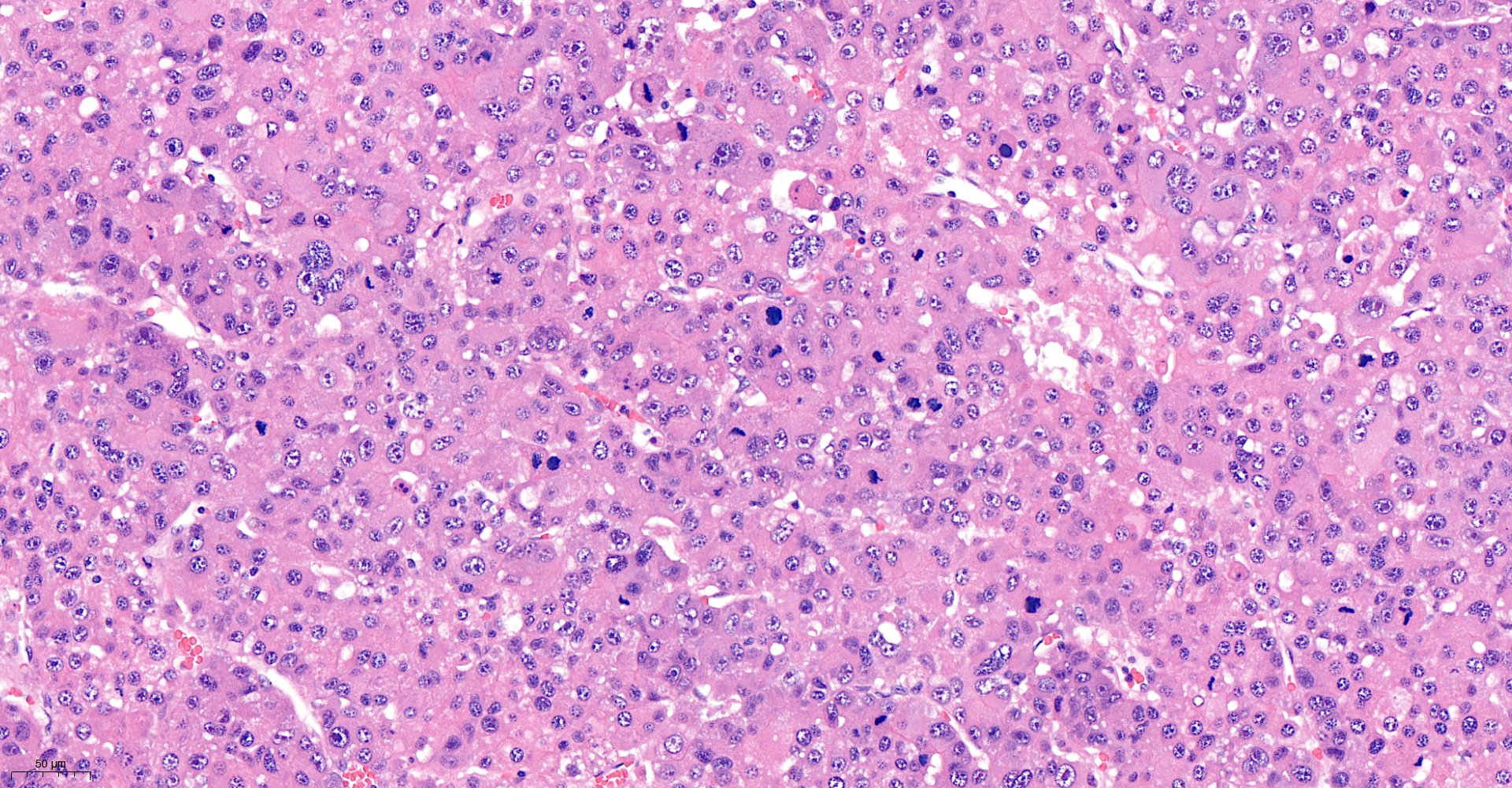

Supplement: Figure 4—source data 1. [file elife-70471-fig4-data1.zip › Figure 4-Source data/hepatocellular cancer patient 4/Raw data-HE staining image 2 of patient 4-20.0x.jpg]

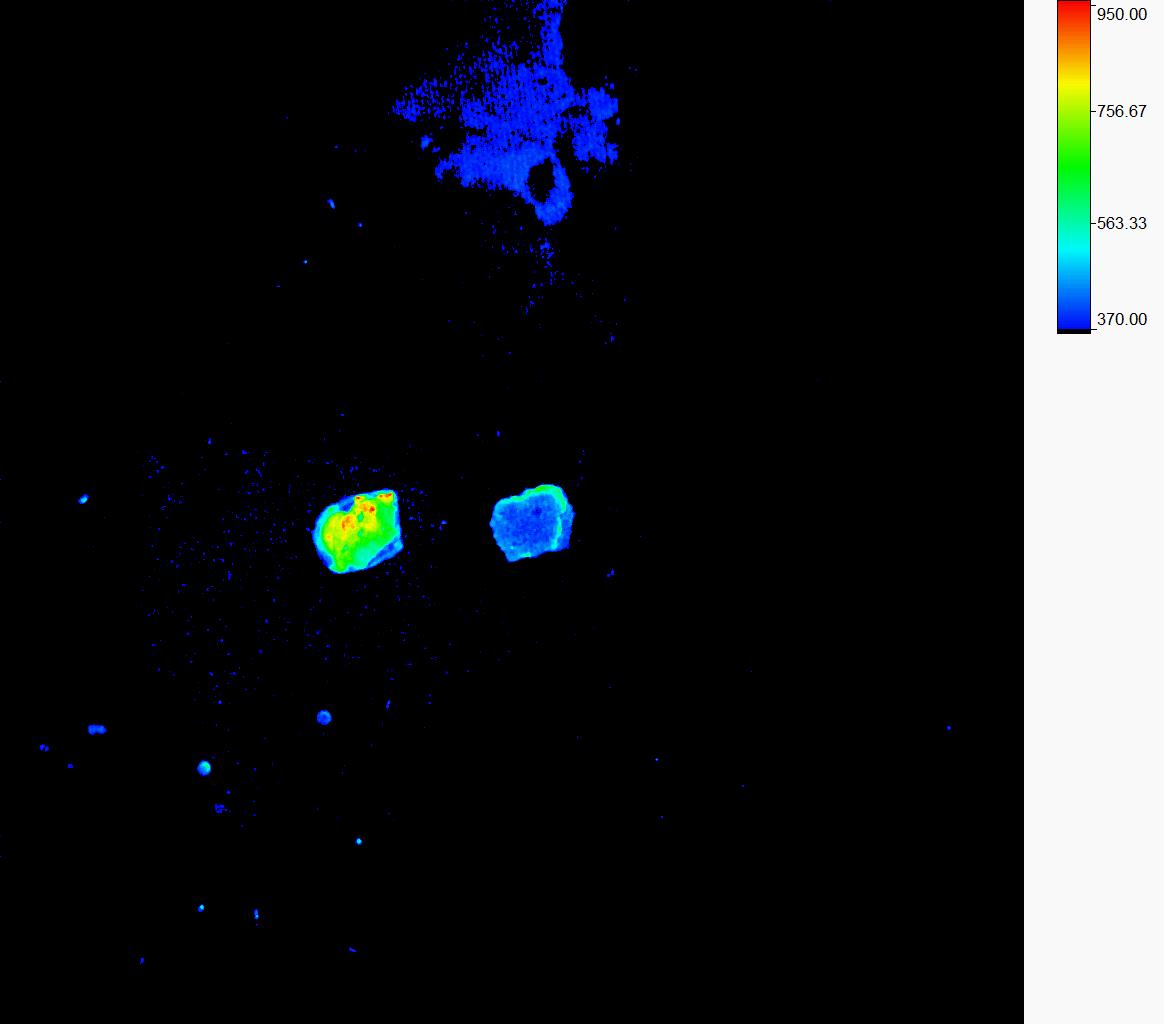

Supplement: Figure 4—source data 1. [file elife-70471-fig4-data1.zip › Figure 4-Source data/hepatocellular cancer patient 4/Raw data-nitroreductase detection image.jpg]

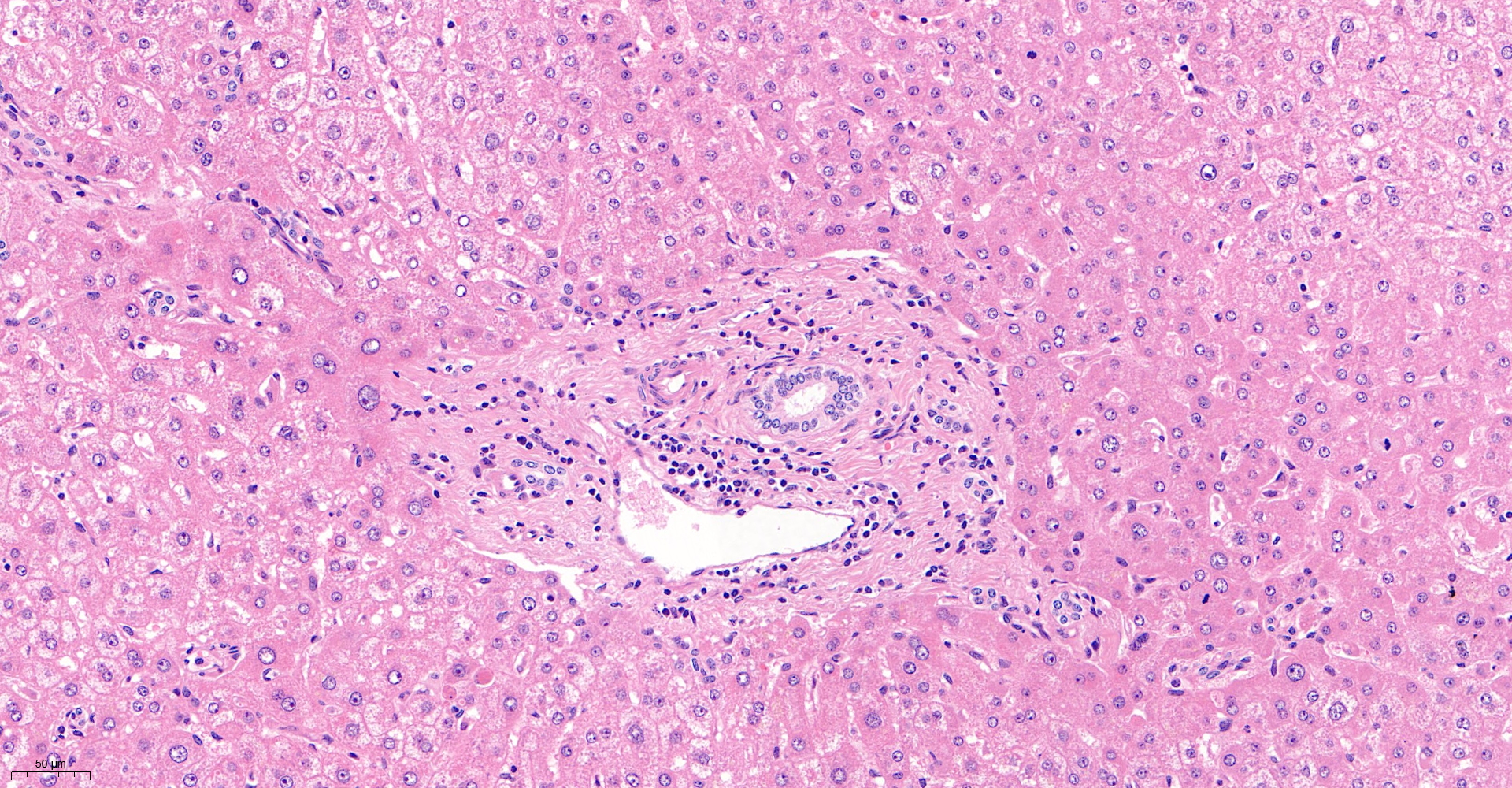

Supplement: Figure 4—source data 1. [file elife-70471-fig4-data1.zip › Figure 4-Source data/hepatocellular cancer patient 4/Raw data-HE staining image 1 of patient 4-20.0x.jpg]

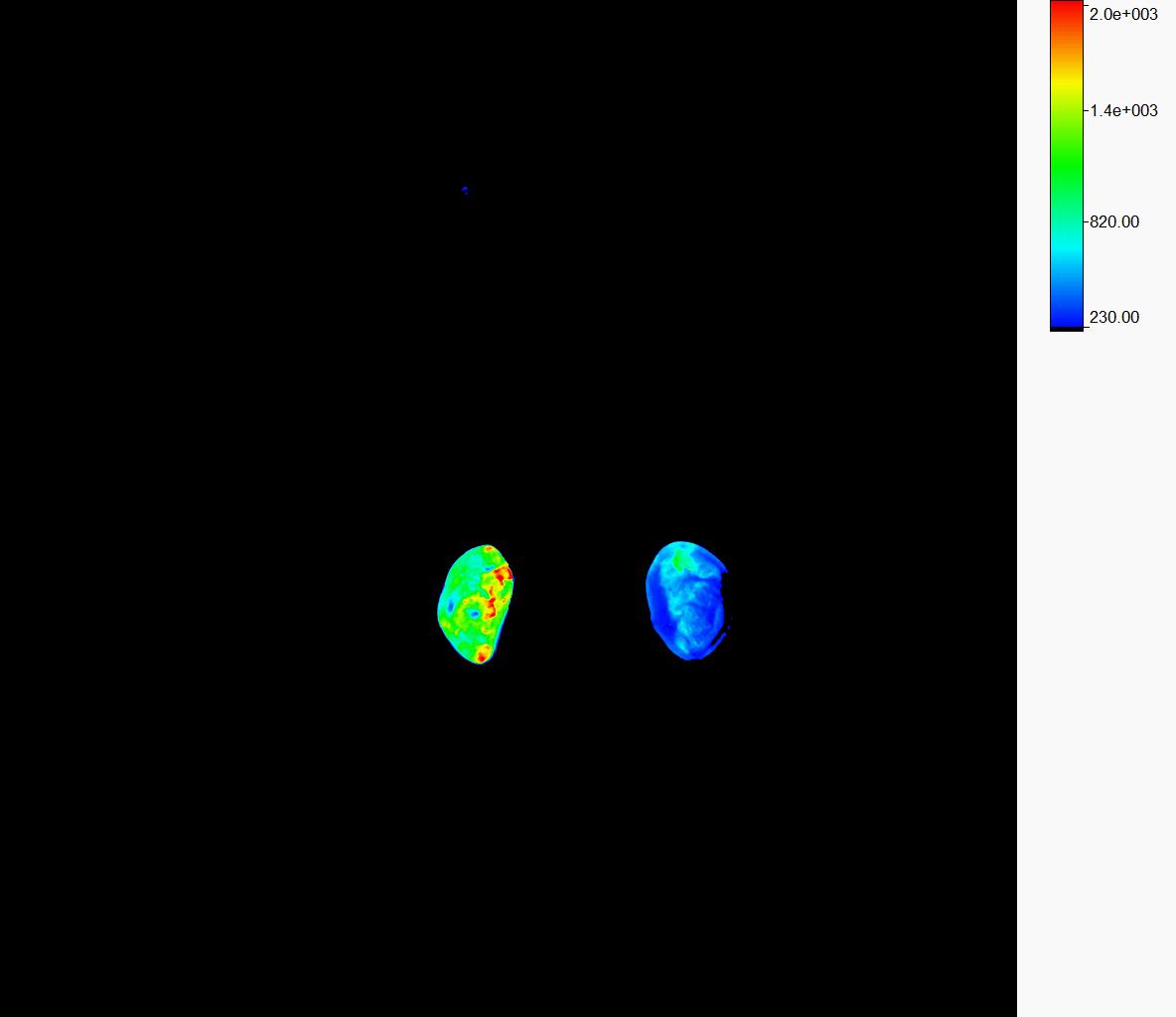

Supplement: Figure 5—source data 1. [file elife-70471-fig5-data1.zip › Figure 5-Source data/lung cancer patient 2/Raw data-viscosity detection image.jpg]

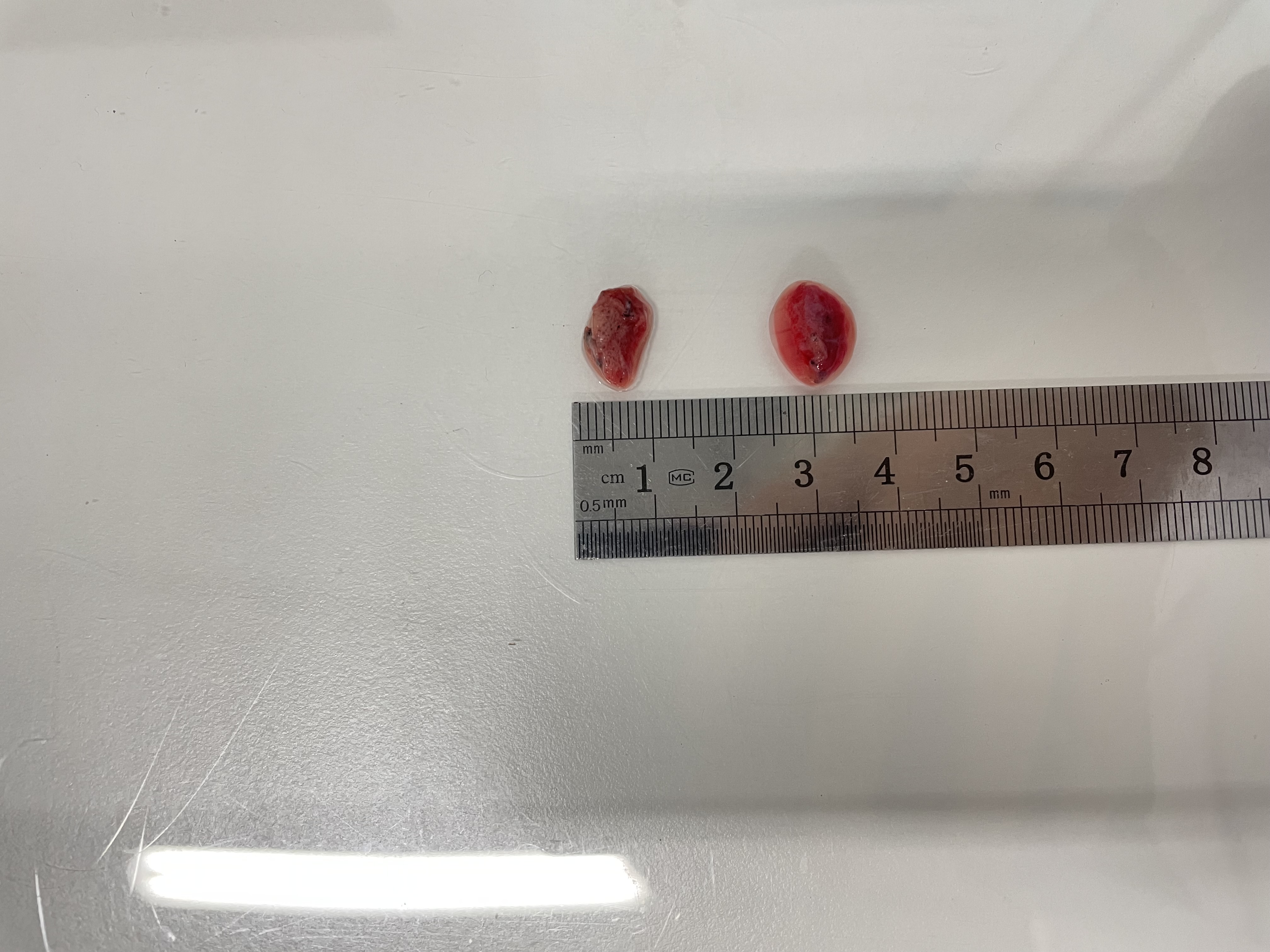

Supplement: Figure 5—source data 1. [file elife-70471-fig5-data1.zip › Figure 5-Source data/lung cancer patient 2/Raw data-photograph image.png]

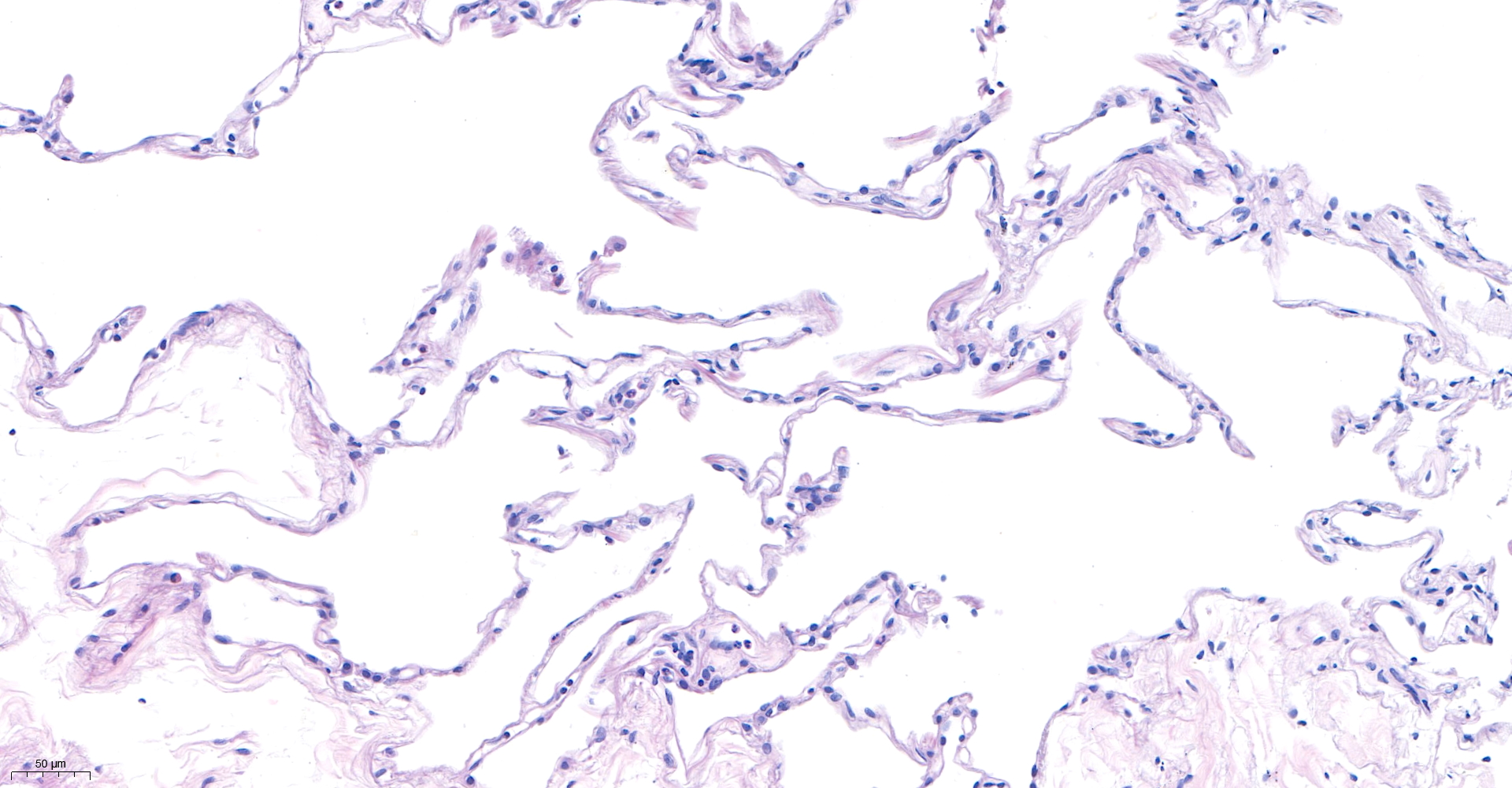

Supplement: Figure 5—source data 1. [file elife-70471-fig5-data1.zip › Figure 5-Source data/lung cancer patient 2/Raw data-HE staining image 1 of patient 2-20.0x.jpg]

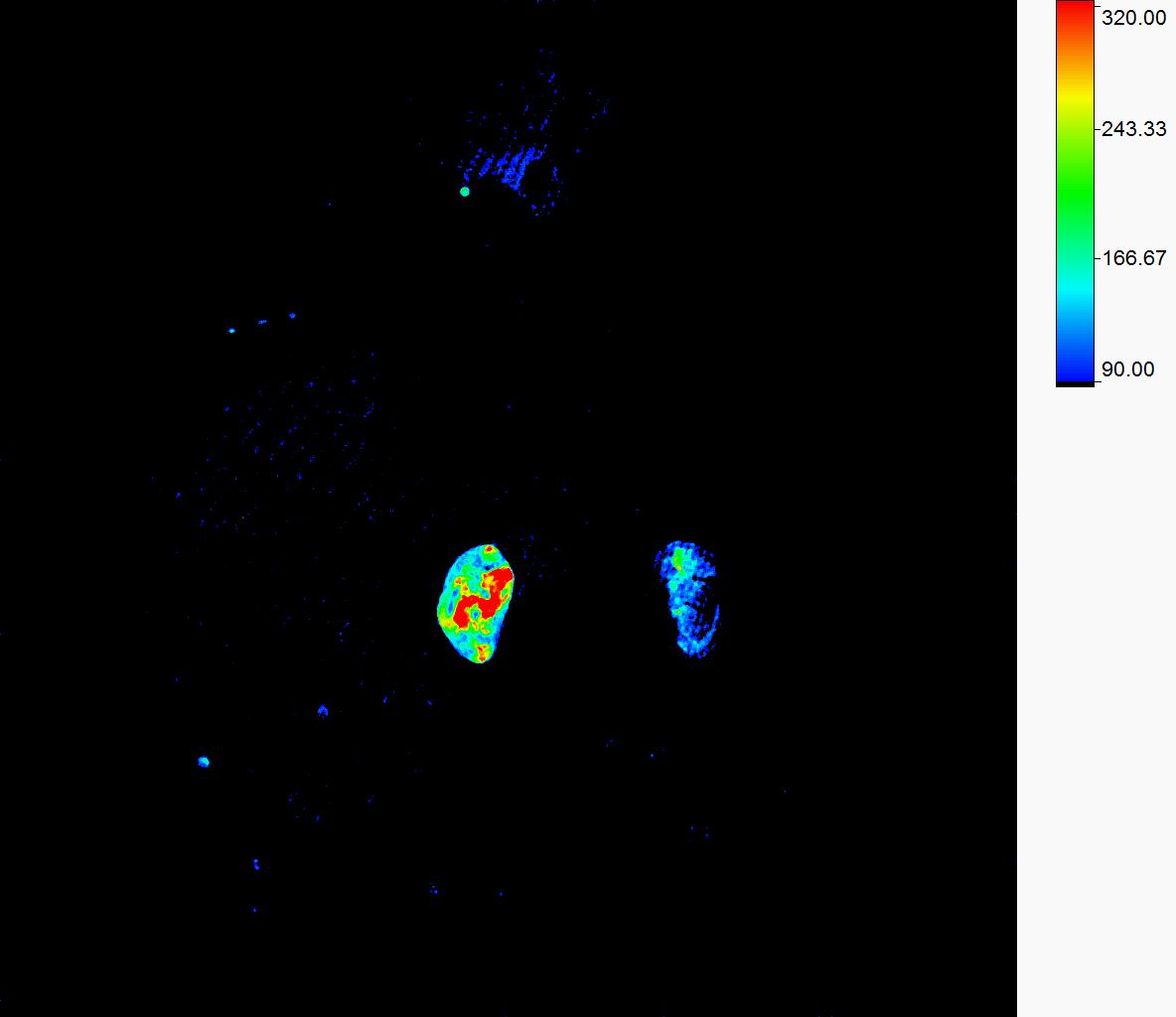

Supplement: Figure 5—source data 1. [file elife-70471-fig5-data1.zip › Figure 5-Source data/lung cancer patient 2/Raw data-nitroreductase detection image.jpg]

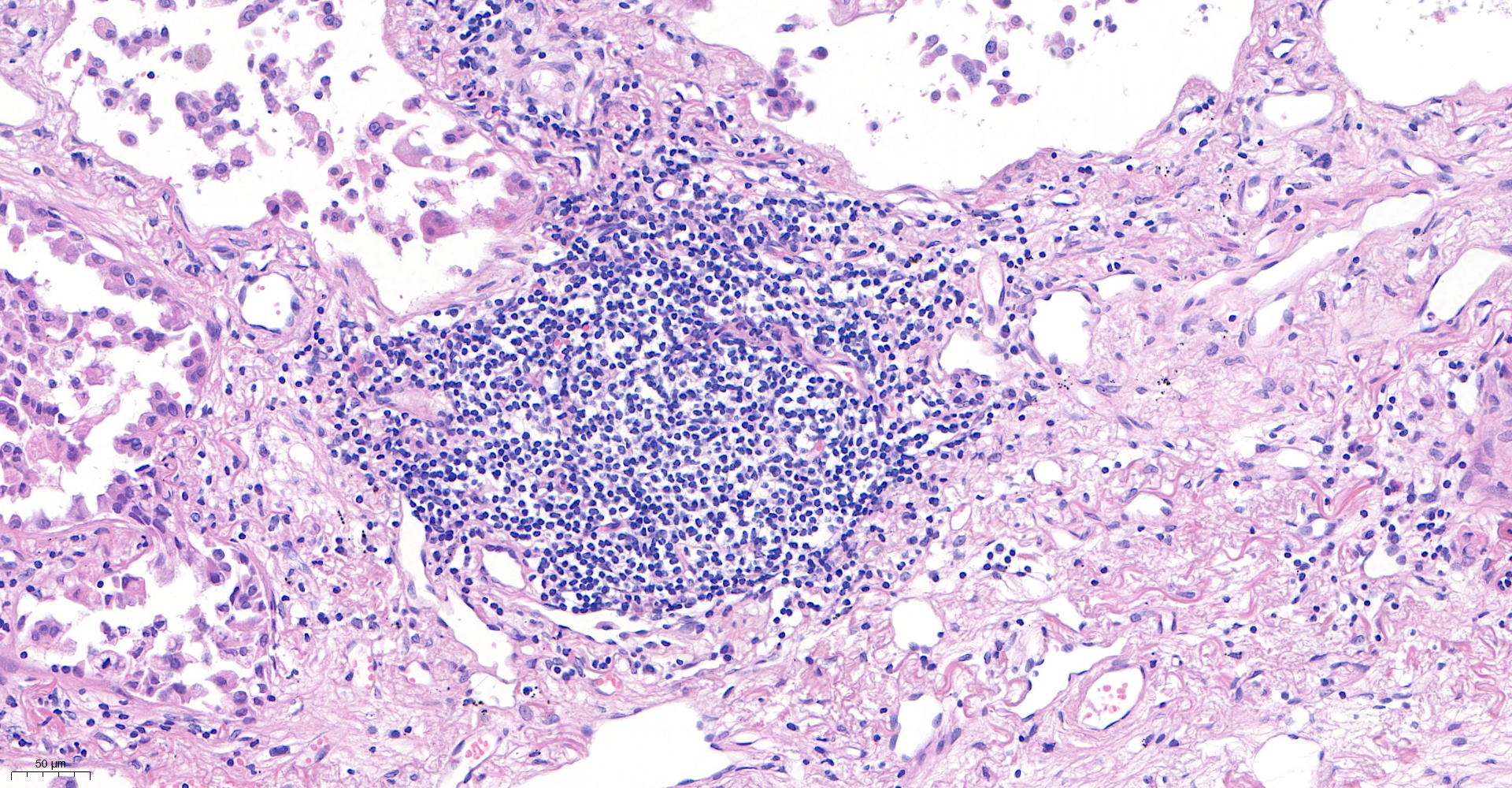

Supplement: Figure 5—source data 1. [file elife-70471-fig5-data1.zip › Figure 5-Source data/lung cancer patient 2/Raw data-HE staining image 2 of patient 2-20.0x.jpg]

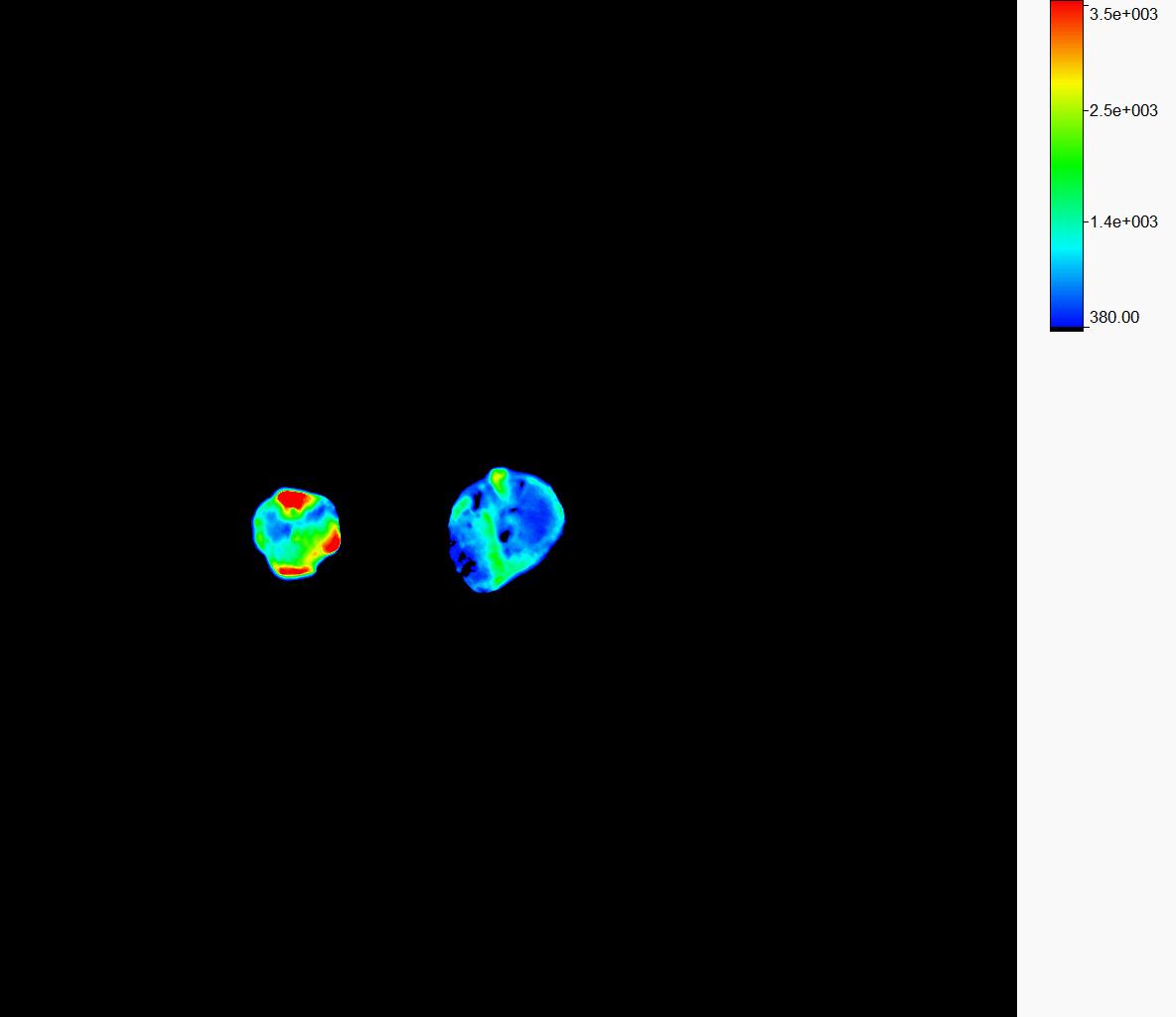

Supplement: Figure 5—source data 1. [file elife-70471-fig5-data1.zip › Figure 5-Source data/lung cancer patient 5/Raw data-viscosity detection image.jpg]

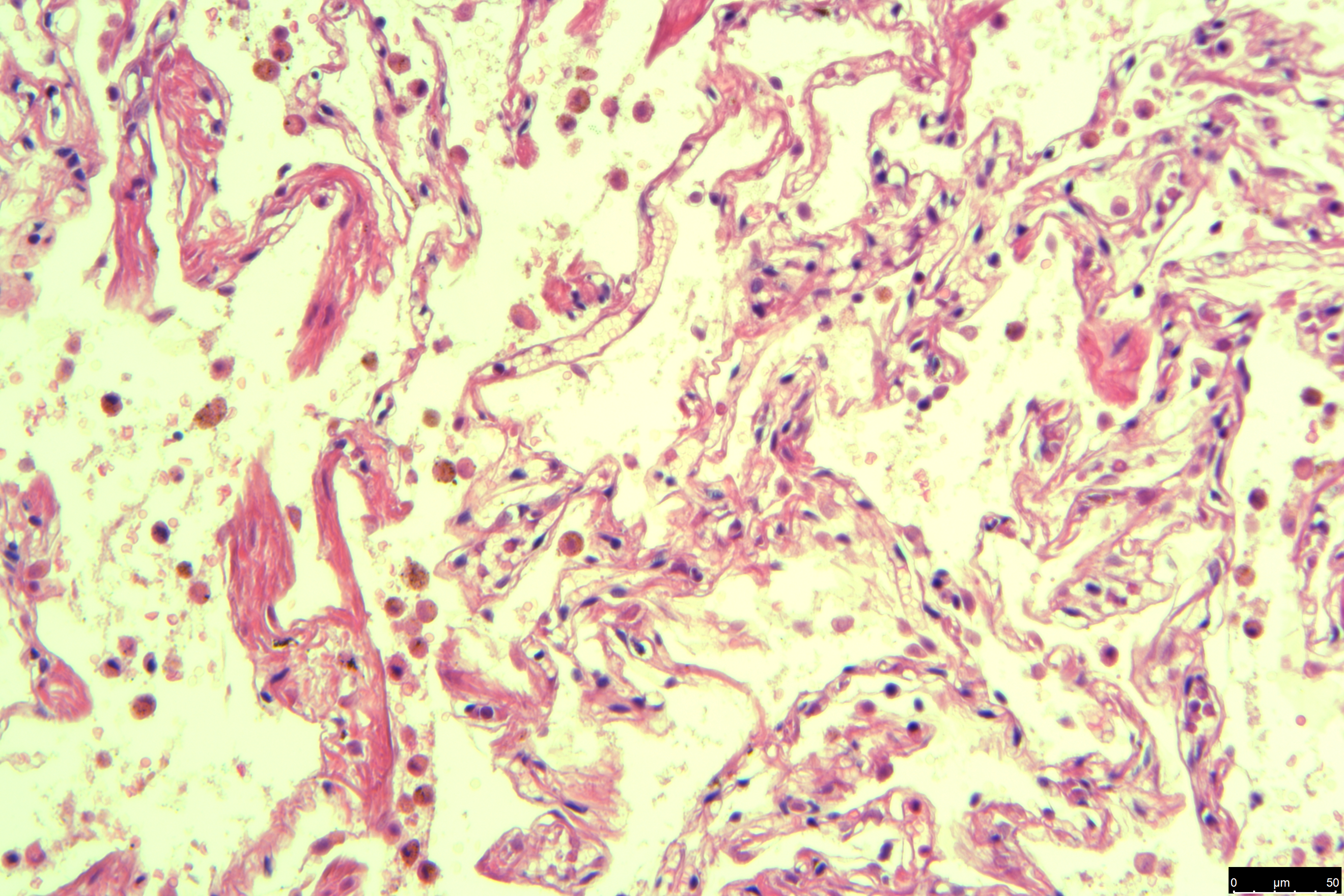

Supplement: Figure 5—source data 1. [file elife-70471-fig5-data1.zip › Figure 5-Source data/lung cancer patient 5/Raw data-HE staining image 1 of patient 4-20.0x.tif]

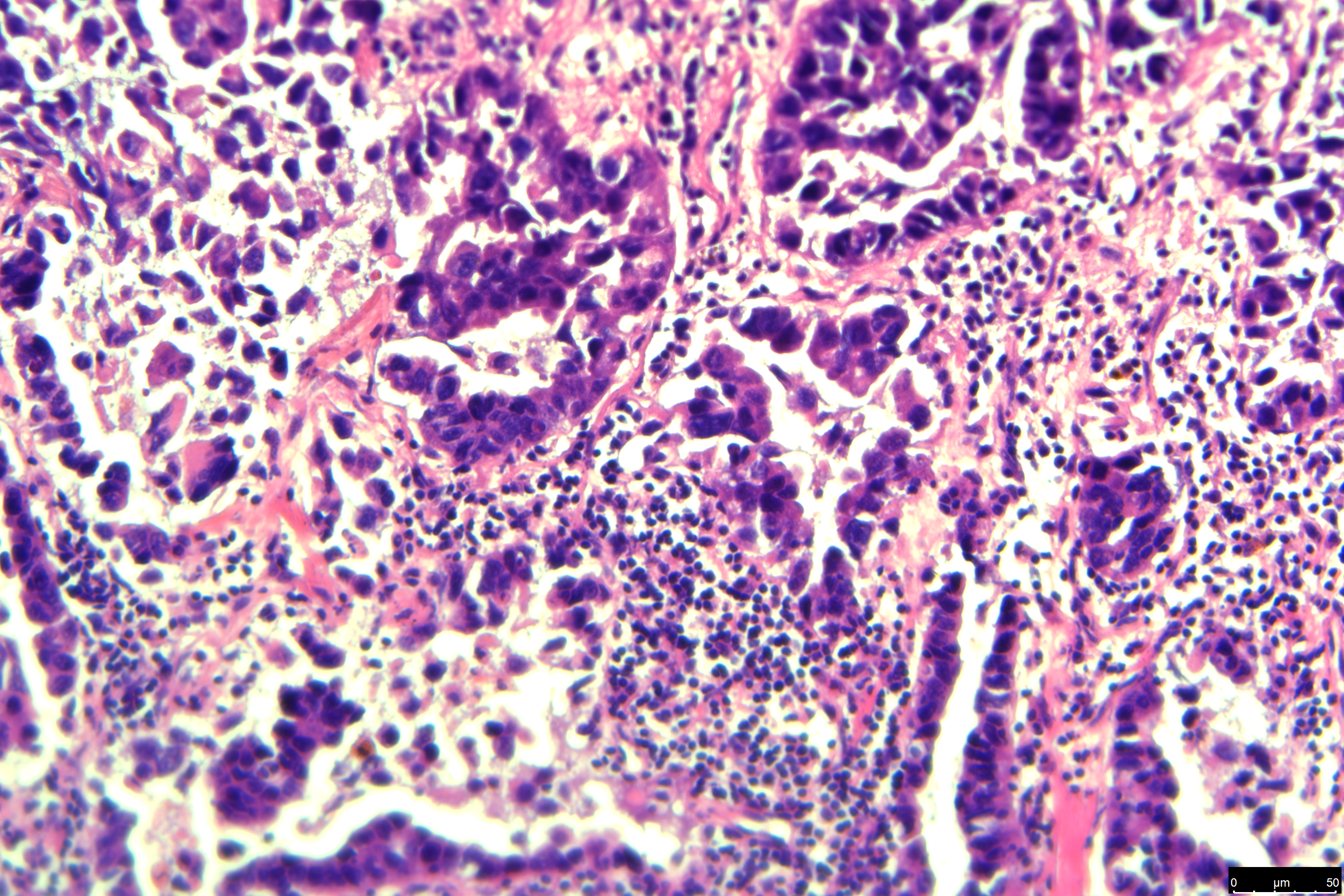

Supplement: Figure 5—source data 1. [file elife-70471-fig5-data1.zip › Figure 5-Source data/lung cancer patient 5/Raw data-HE staining image 2 of patient 4-20.0x.tif]

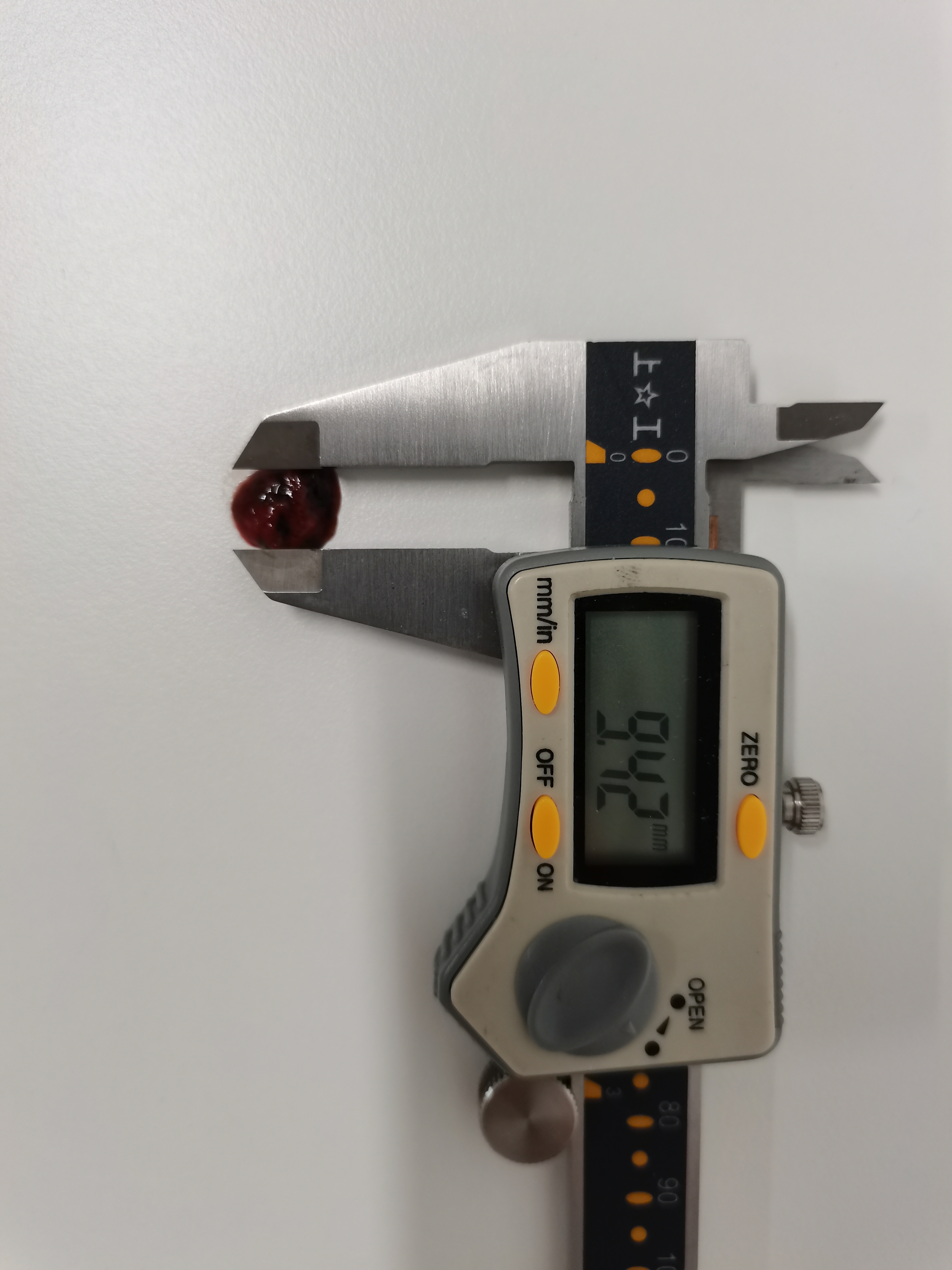

Supplement: Figure 5—source data 1. [file elife-70471-fig5-data1.zip › Figure 5-Source data/lung cancer patient 5/Raw data-photograph image 2.jpg]

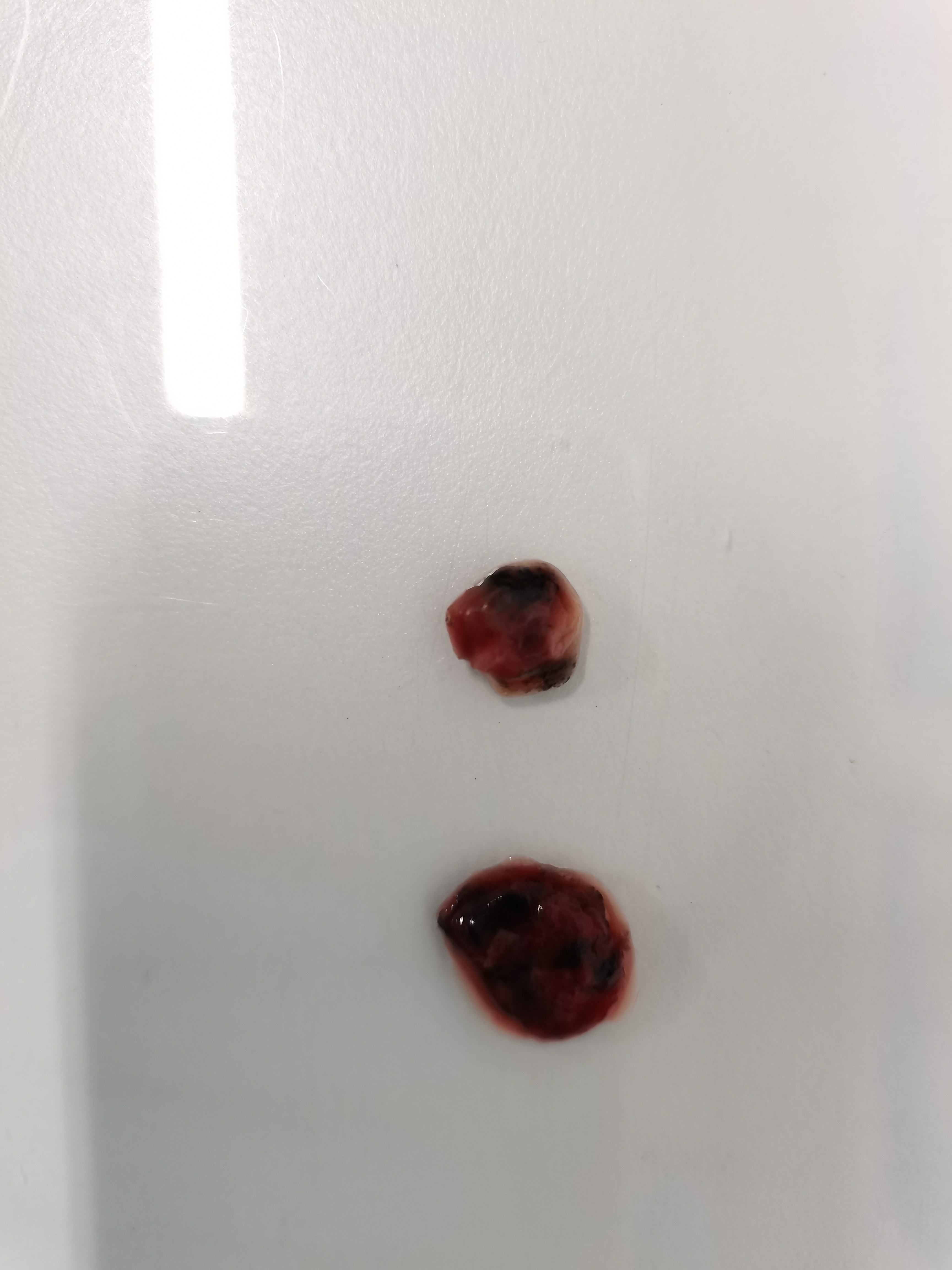

Supplement: Figure 5—source data 1. [file elife-70471-fig5-data1.zip › Figure 5-Source data/lung cancer patient 5/Raw data-photograph image 1.jpg]

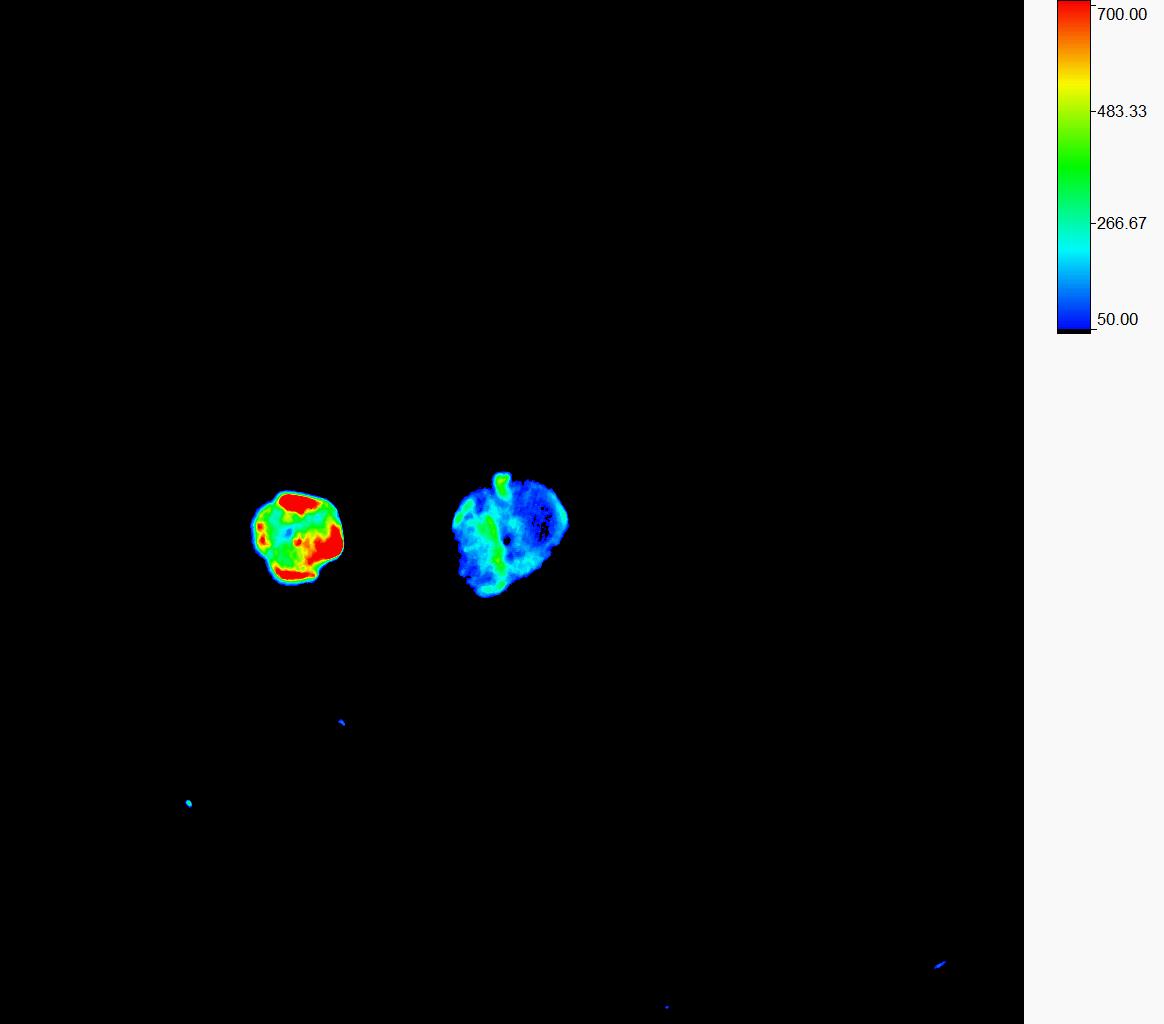

Supplement: Figure 5—source data 1. [file elife-70471-fig5-data1.zip › Figure 5-Source data/lung cancer patient 5/Raw data-nitroreductase detection image.jpg]

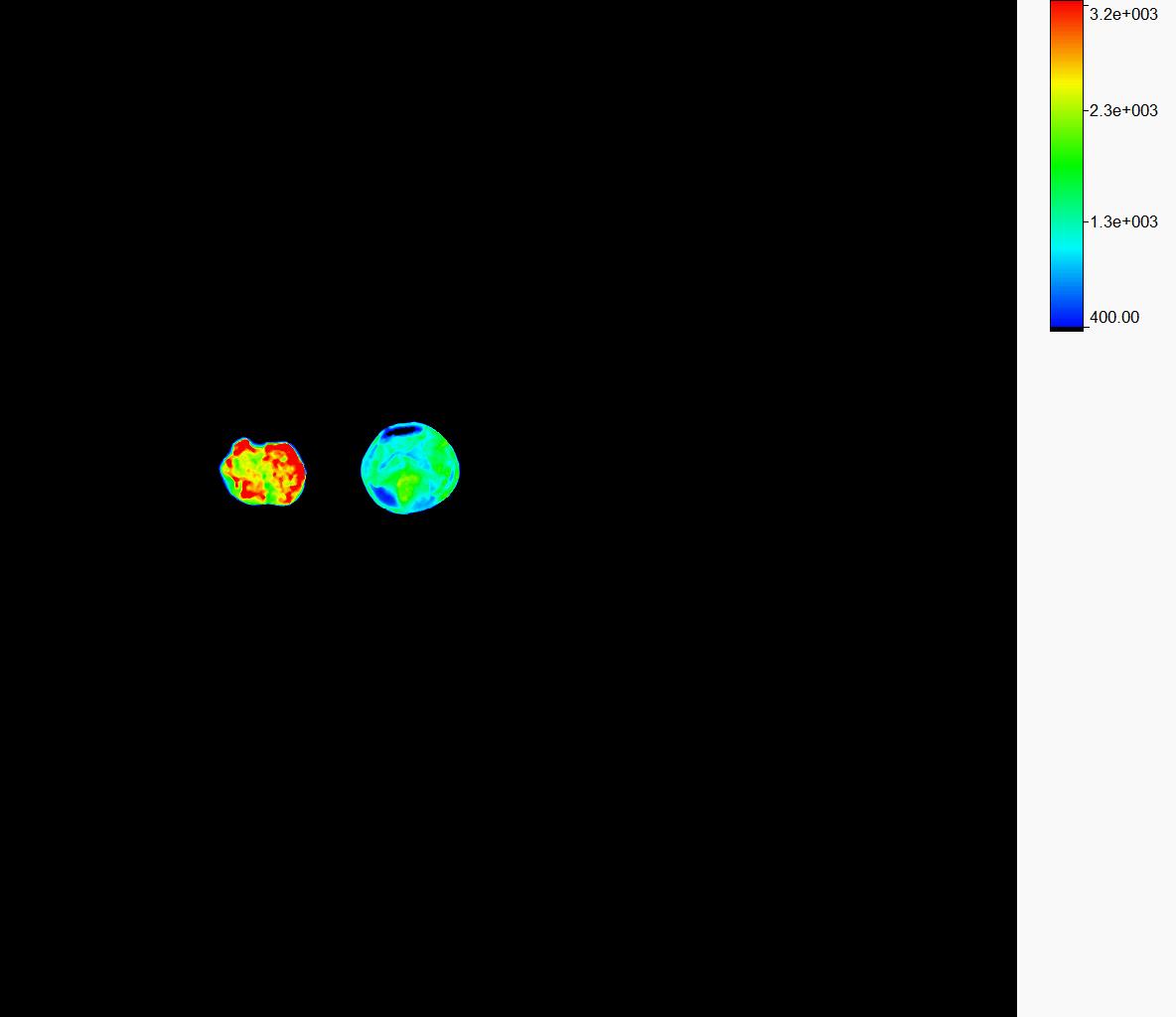

Supplement: Figure 5—source data 1. [file elife-70471-fig5-data1.zip › Figure 5-Source data/lung cancer patient 4/Raw data-viscosity detection image.jpg]

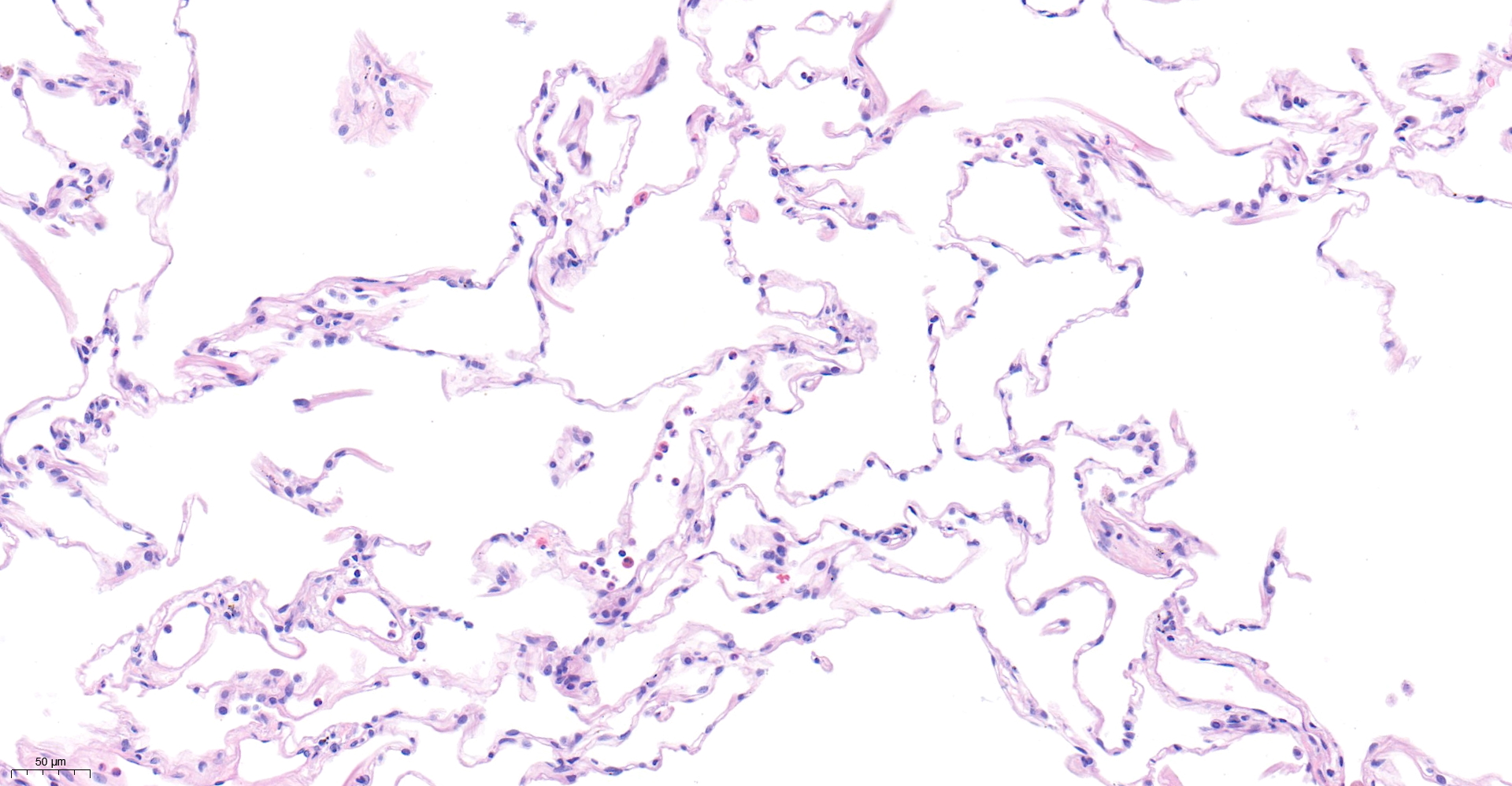

Supplement: Figure 5—source data 1. [file elife-70471-fig5-data1.zip › Figure 5-Source data/lung cancer patient 4/Raw data-HE staining image 2 of patient 5-20.0x.jpg]

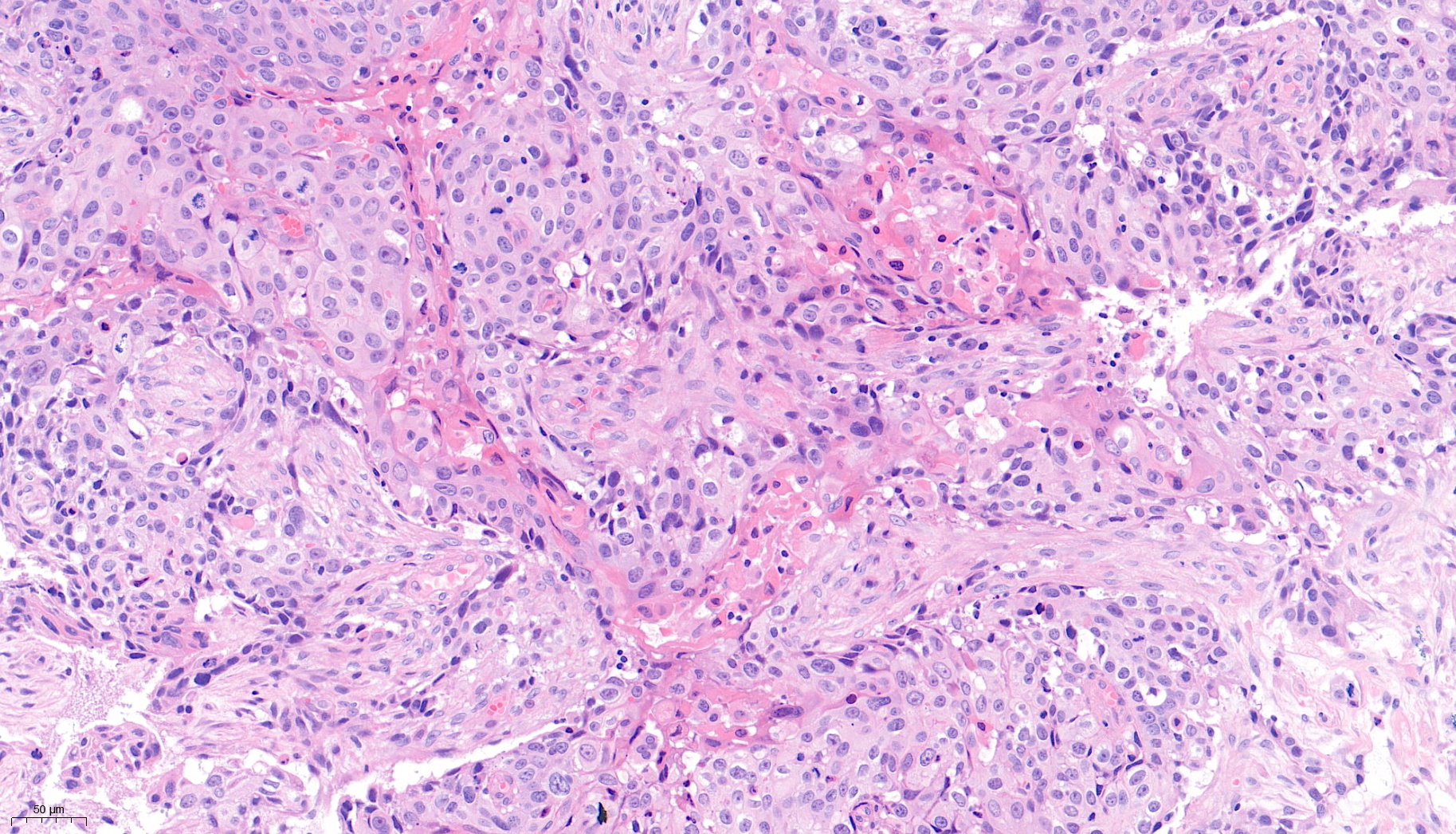

Supplement: Figure 5—source data 1. [file elife-70471-fig5-data1.zip › Figure 5-Source data/lung cancer patient 4/Raw data-HE staining image 1 of patient 5-20.0x.jpg]

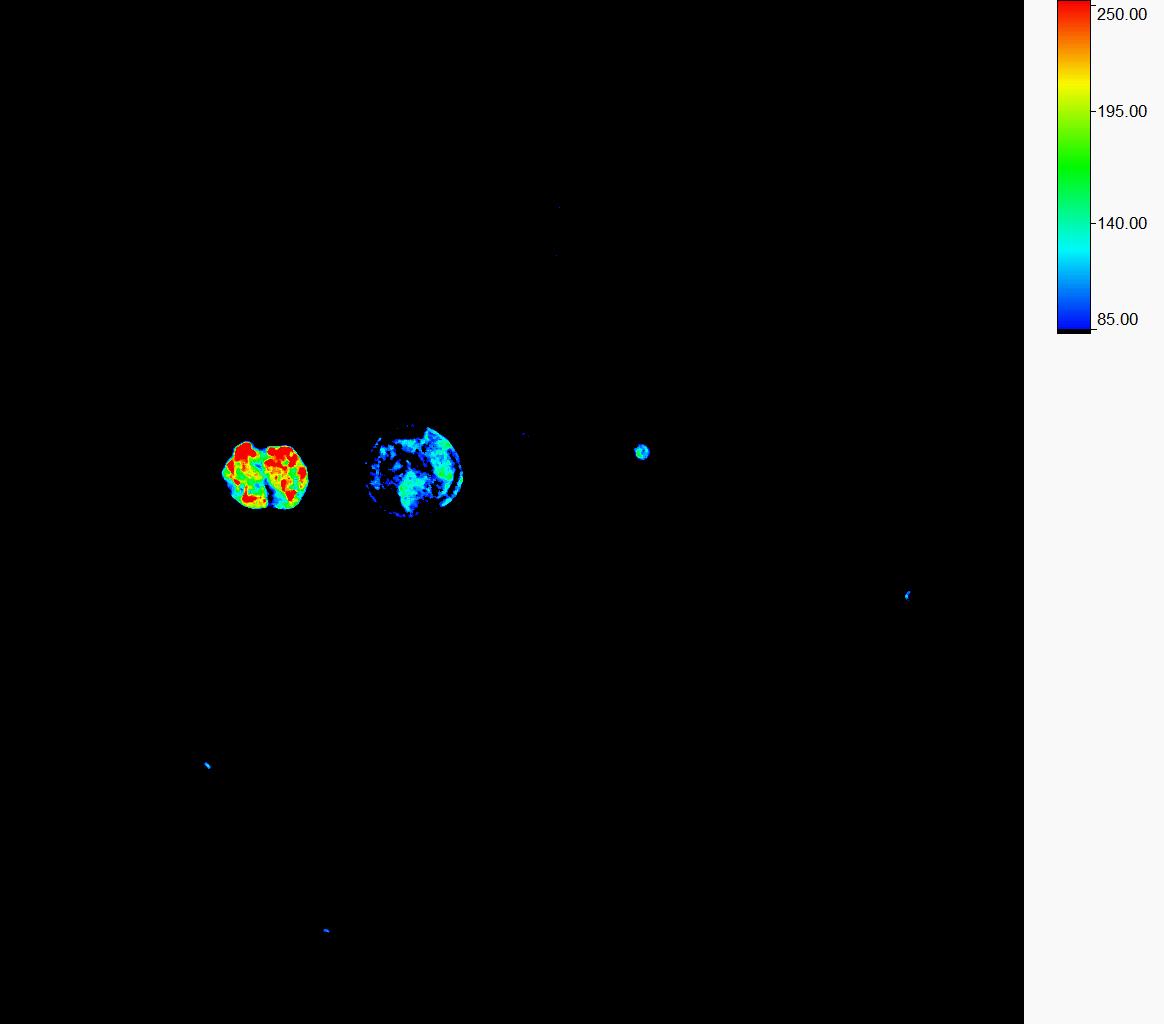

Supplement: Figure 5—source data 1. [file elife-70471-fig5-data1.zip › Figure 5-Source data/lung cancer patient 4/Raw data-nitroreductase detection image.jpg]

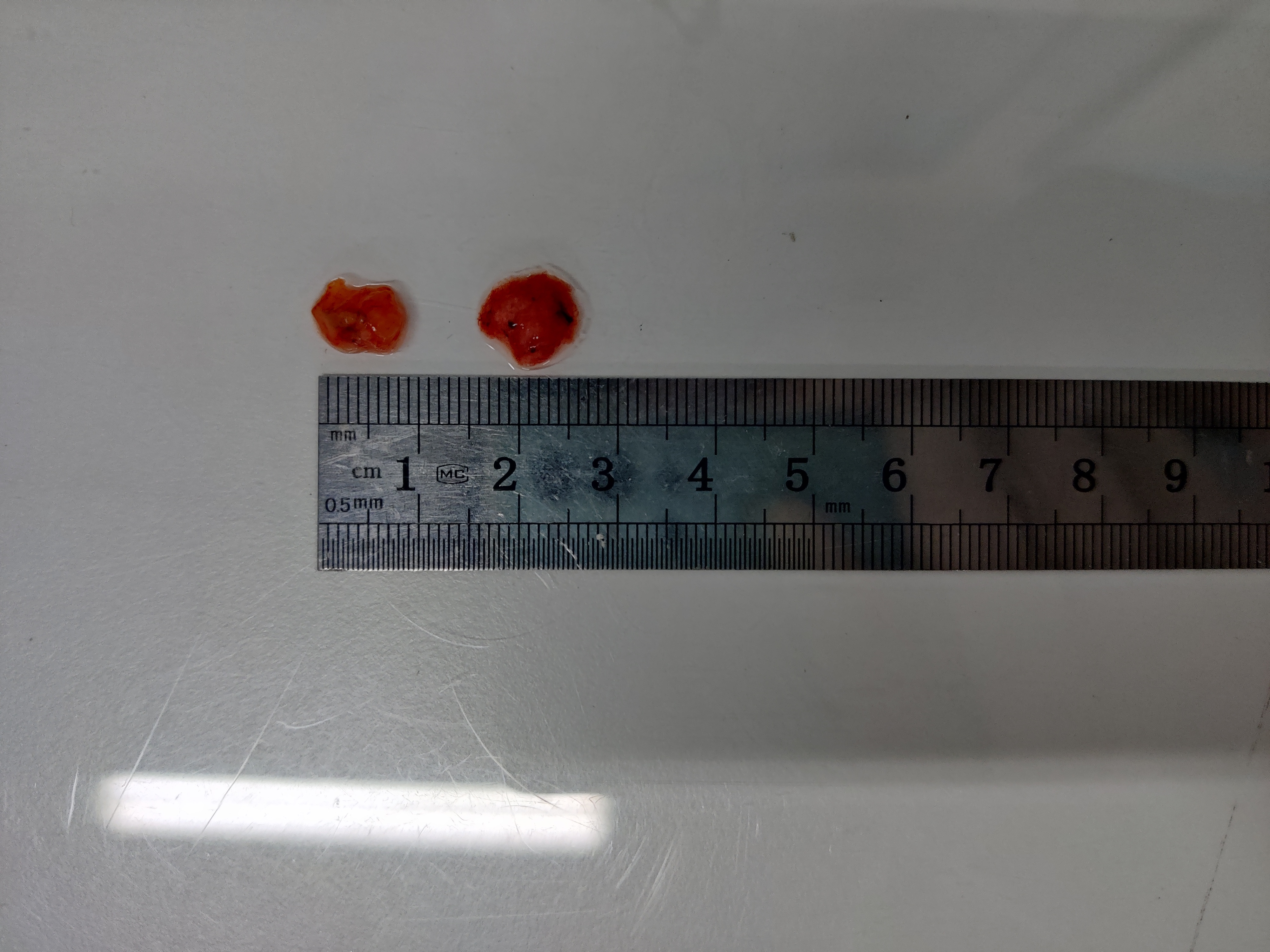

Supplement: Figure 5—source data 1. [file elife-70471-fig5-data1.zip › Figure 5-Source data/lung cancer patient 4/Raw data-photograph image.jpeg]

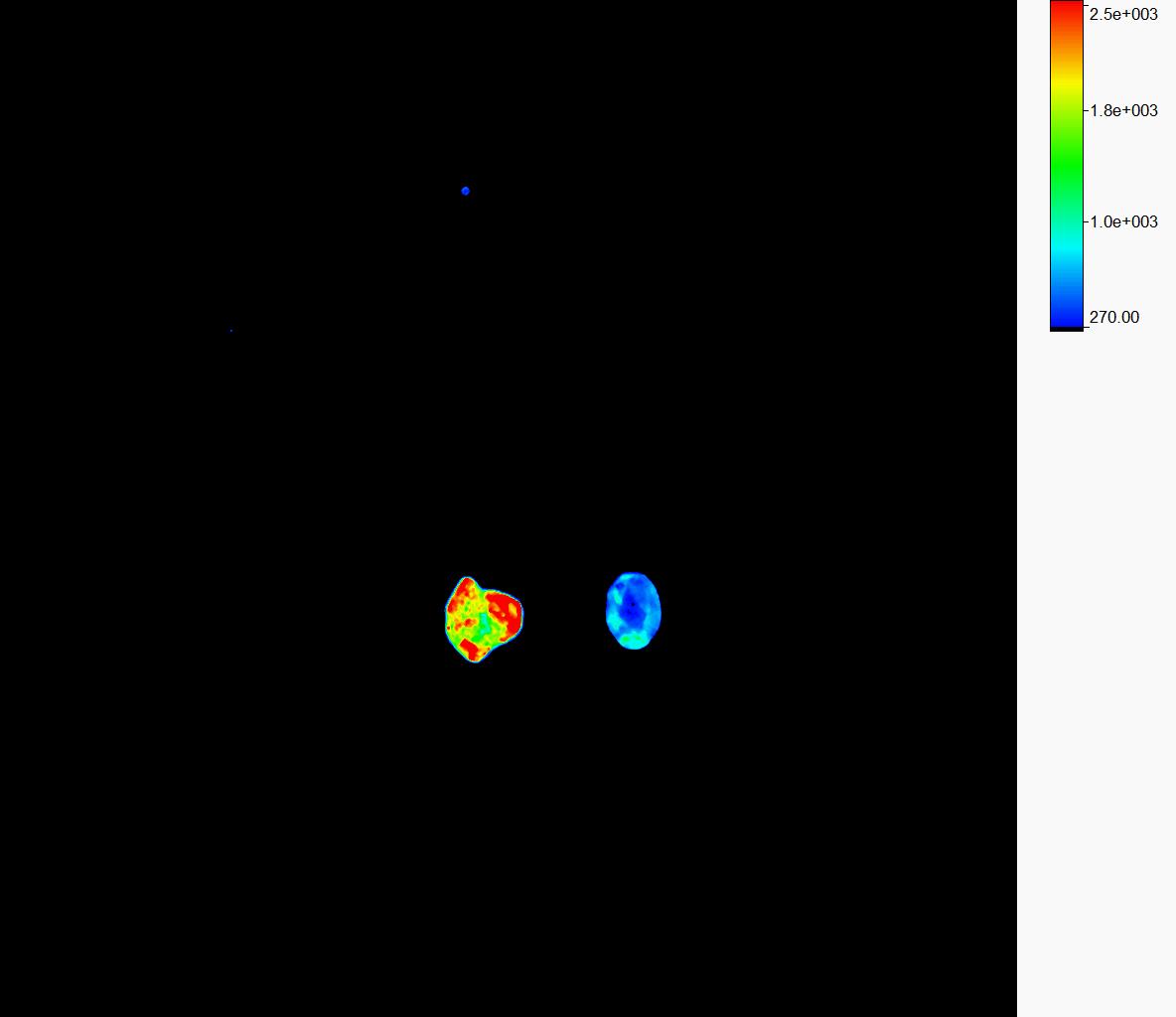

Supplement: Figure 5—source data 1. [file elife-70471-fig5-data1.zip › Figure 5-Source data/lung cancer patient 3/Raw data-viscosity detection image.jpg]

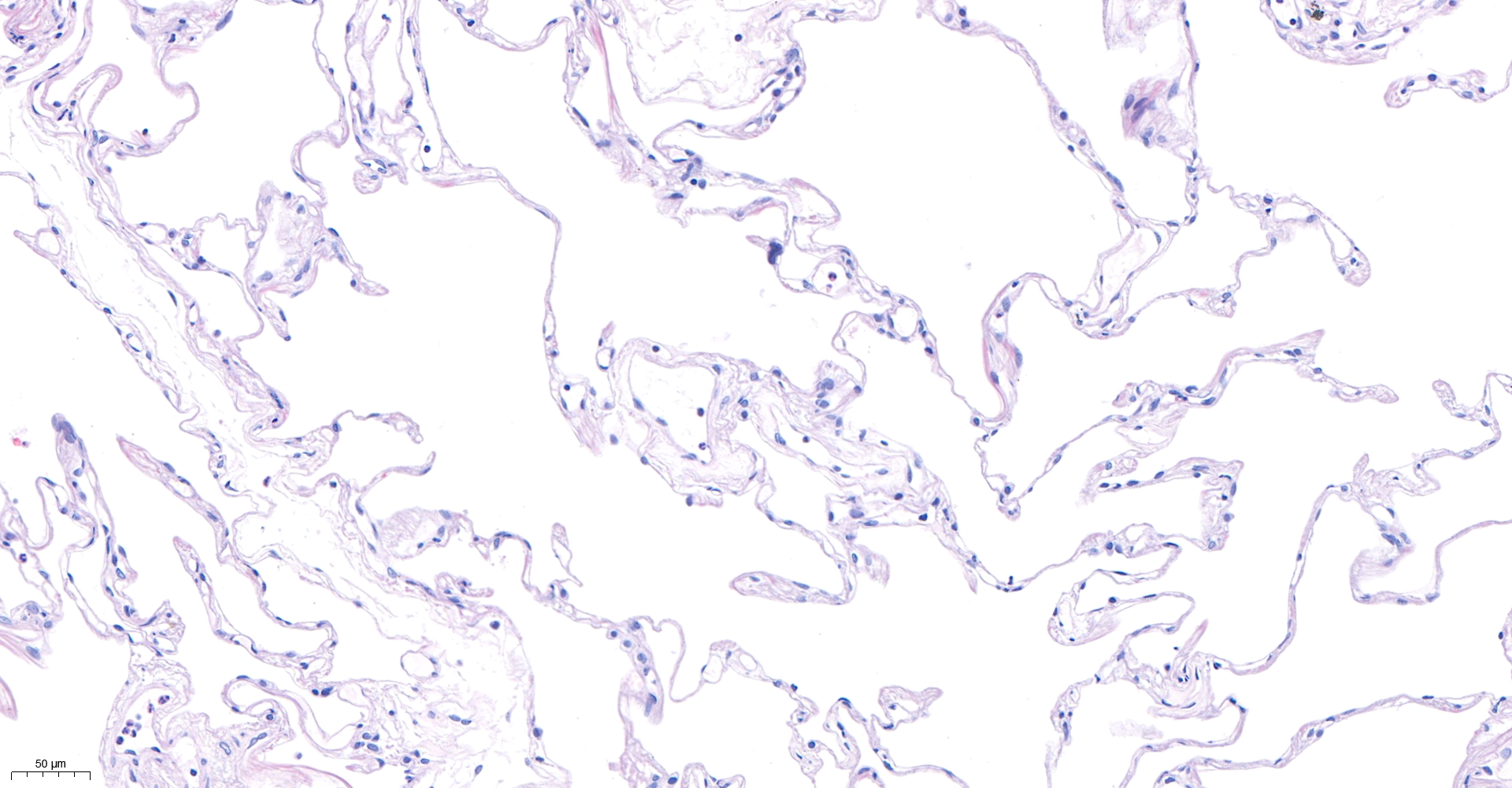

Supplement: Figure 5—source data 1. [file elife-70471-fig5-data1.zip › Figure 5-Source data/lung cancer patient 3/Raw data-HE staining image 1 of patient 3-20.0x.jpg]

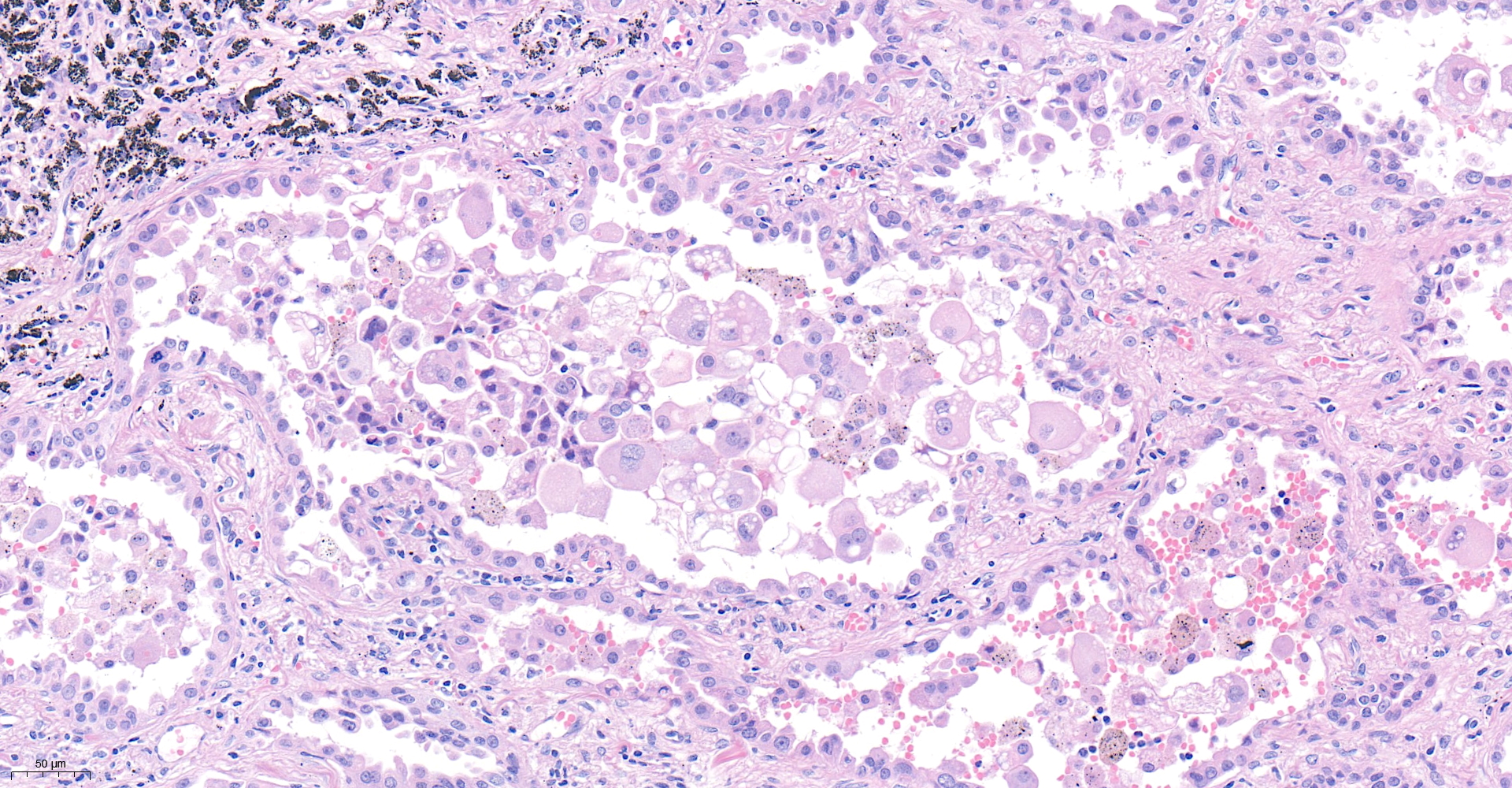

Supplement: Figure 5—source data 1. [file elife-70471-fig5-data1.zip › Figure 5-Source data/lung cancer patient 3/Raw data-HE staining image 2 of patient 3-20.0x.jpg]

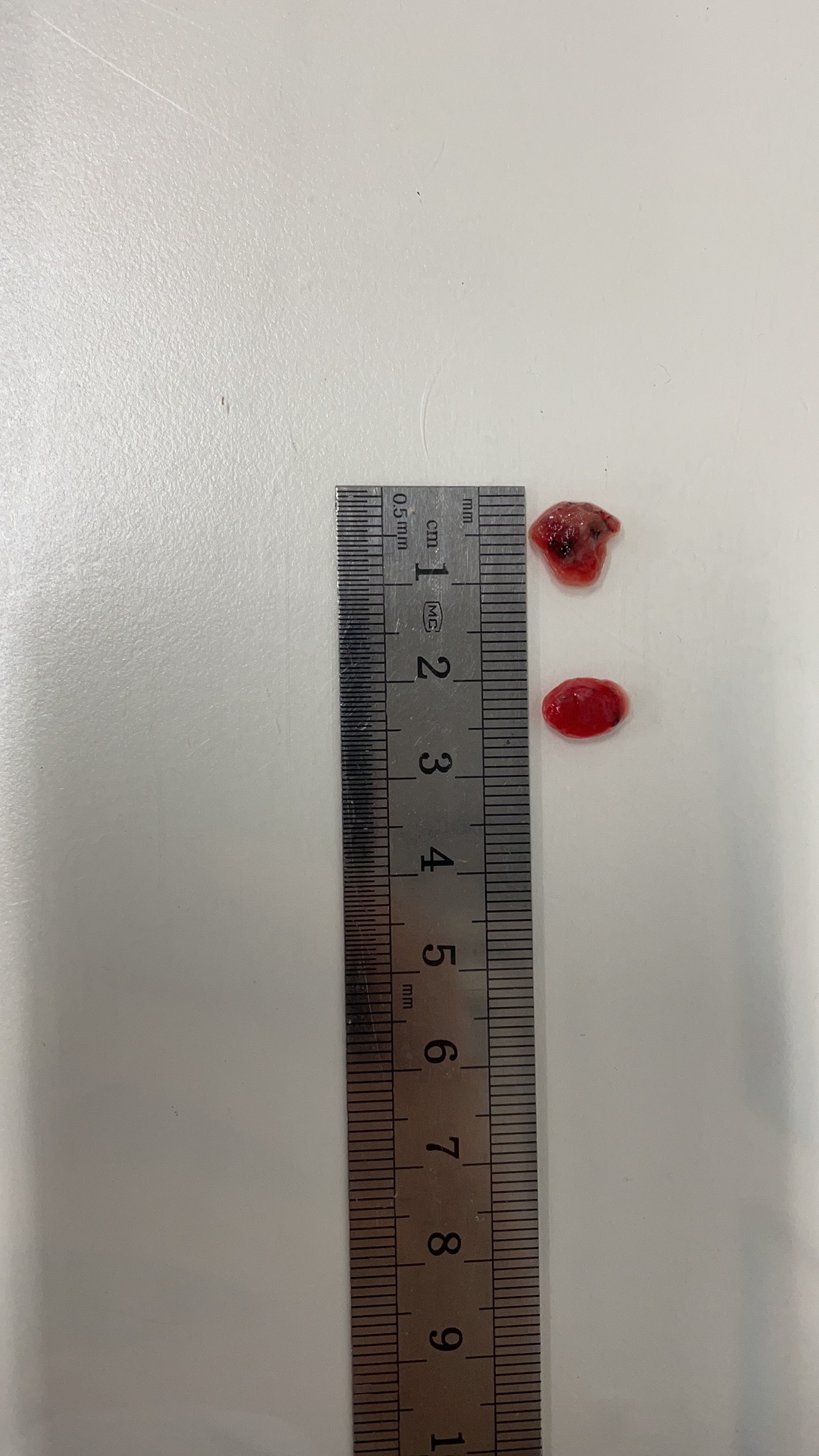

Supplement: Figure 5—source data 1. [file elife-70471-fig5-data1.zip › Figure 5-Source data/lung cancer patient 3/Raw data-photograph image.png]

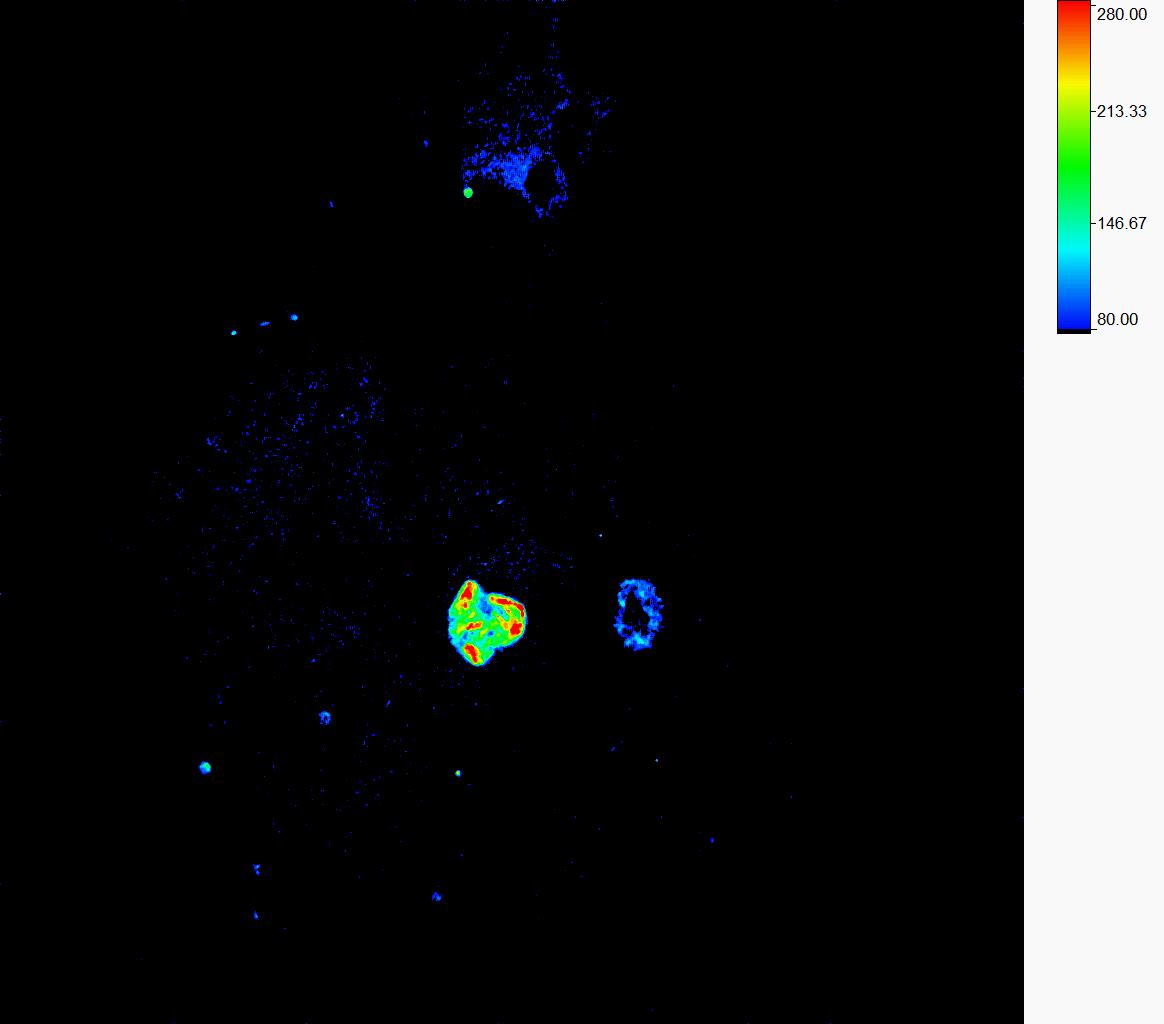

Supplement: Figure 5—source data 1. [file elife-70471-fig5-data1.zip › Figure 5-Source data/lung cancer patient 3/Raw data-nitroreductase detection image.jpg]

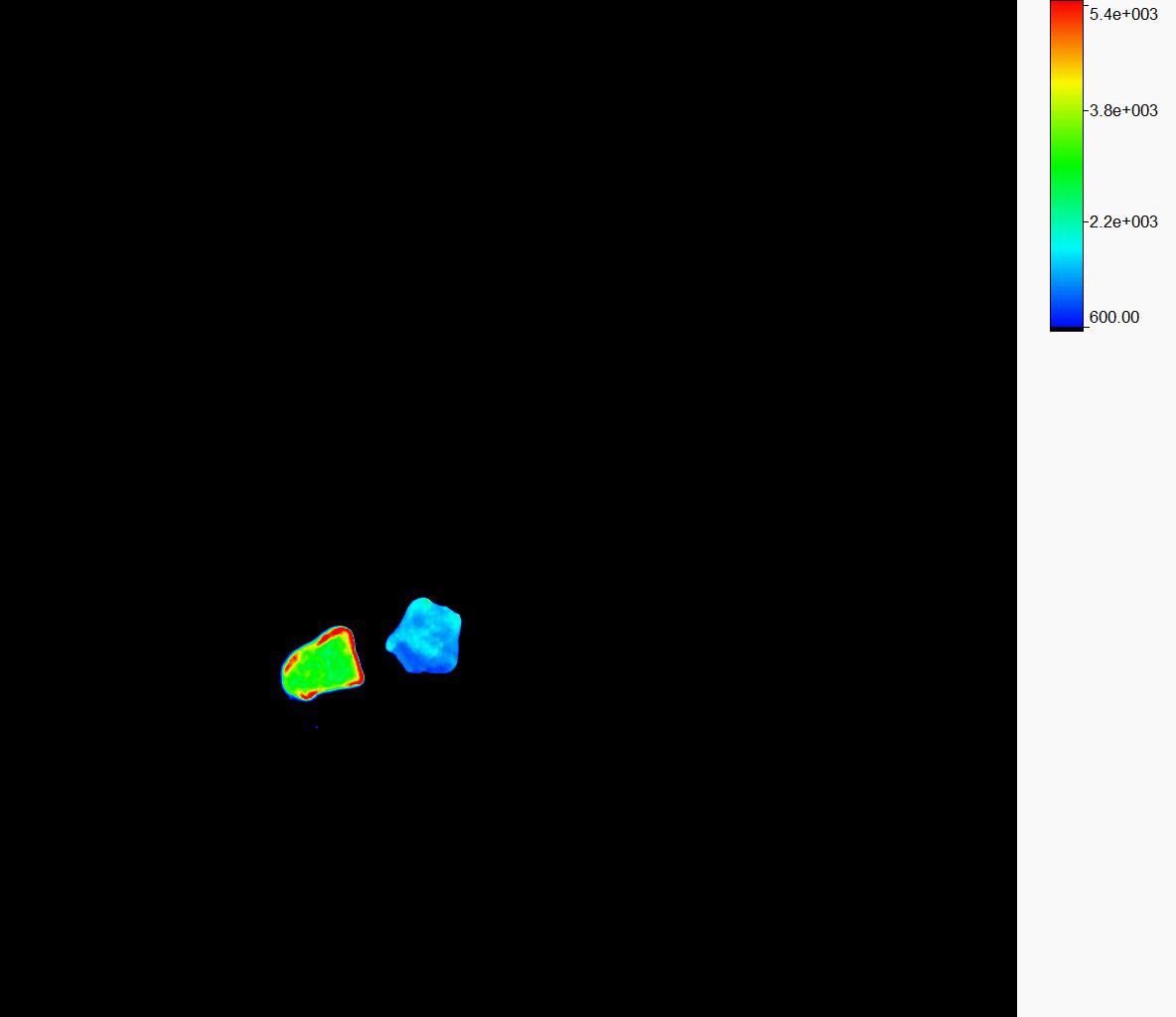

Supplement: Figure 5—source data 1. [file elife-70471-fig5-data1.zip › Figure 5-Source data/lung cancer patient 1/Raw data-viscosity detection image.jpg]

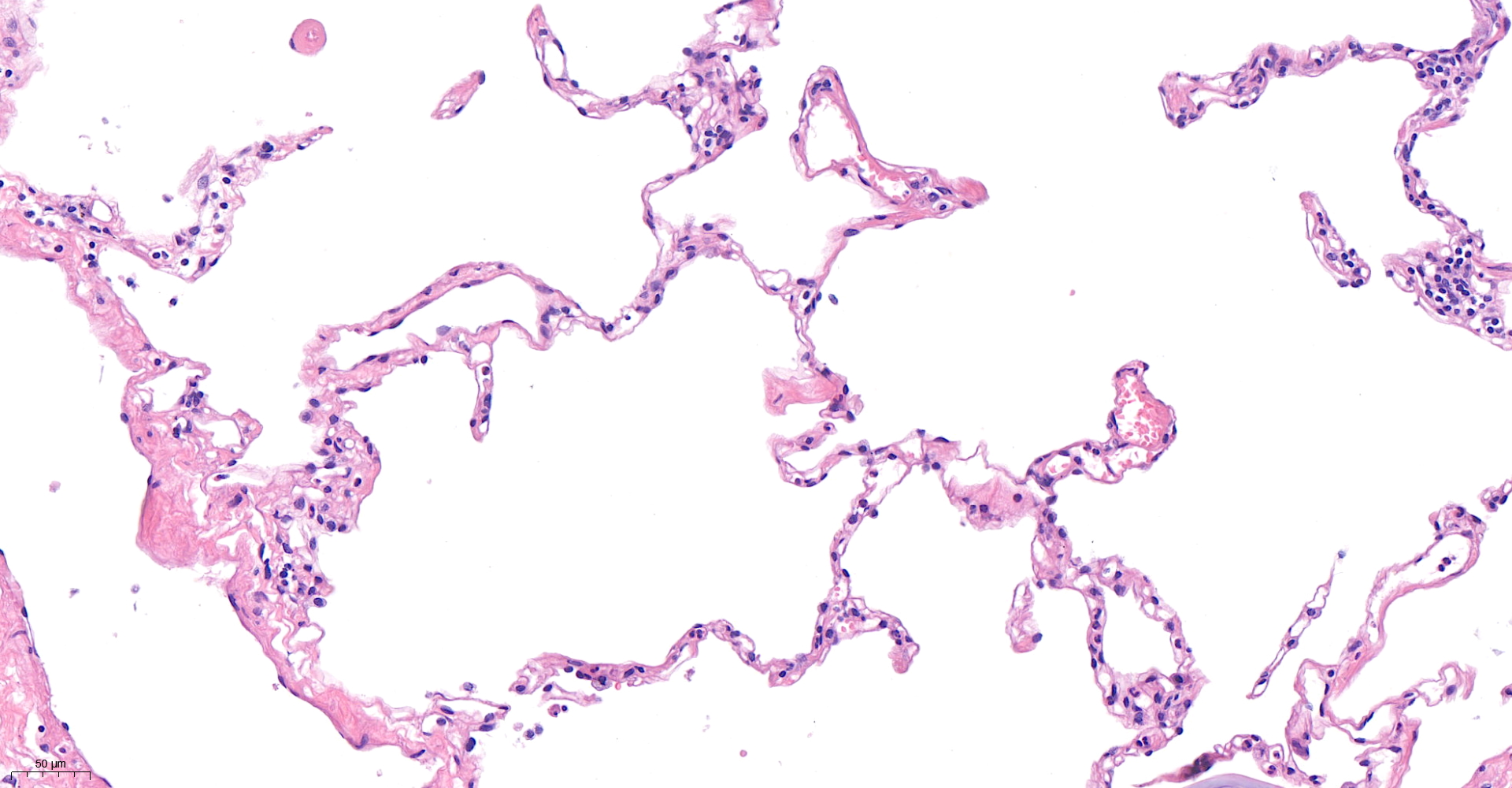

Supplement: Figure 5—source data 1. [file elife-70471-fig5-data1.zip › Figure 5-Source data/lung cancer patient 1/Raw data-HE staining image 1 of patient 1-20.0x.jpg]

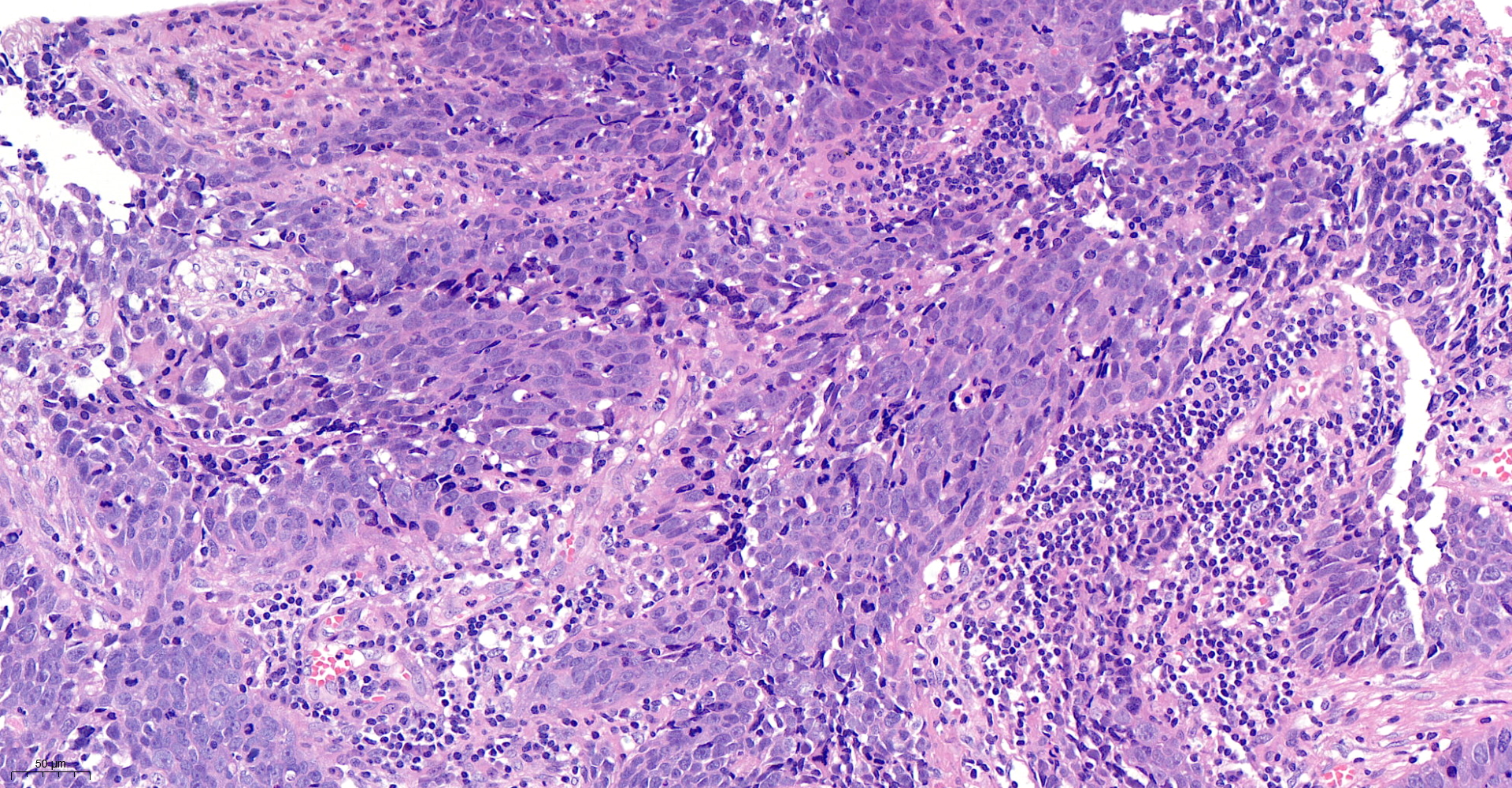

Supplement: Figure 5—source data 1. [file elife-70471-fig5-data1.zip › Figure 5-Source data/lung cancer patient 1/Raw data-HE staining image 2 of patient 1-20.0x.jpg]

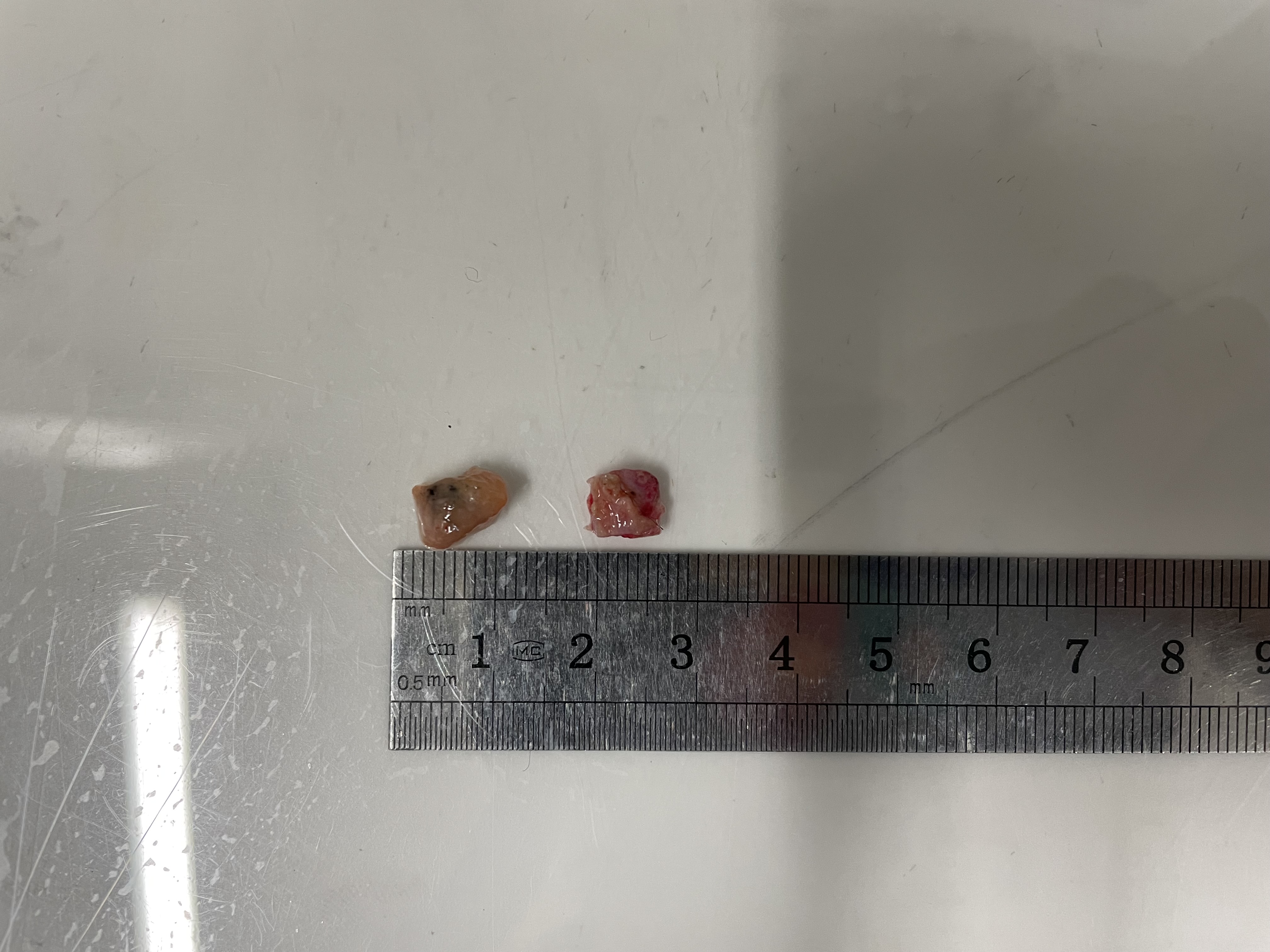

Supplement: Figure 5—source data 1. [file elife-70471-fig5-data1.zip › Figure 5-Source data/lung cancer patient 1/Raw data-photograph image.png]

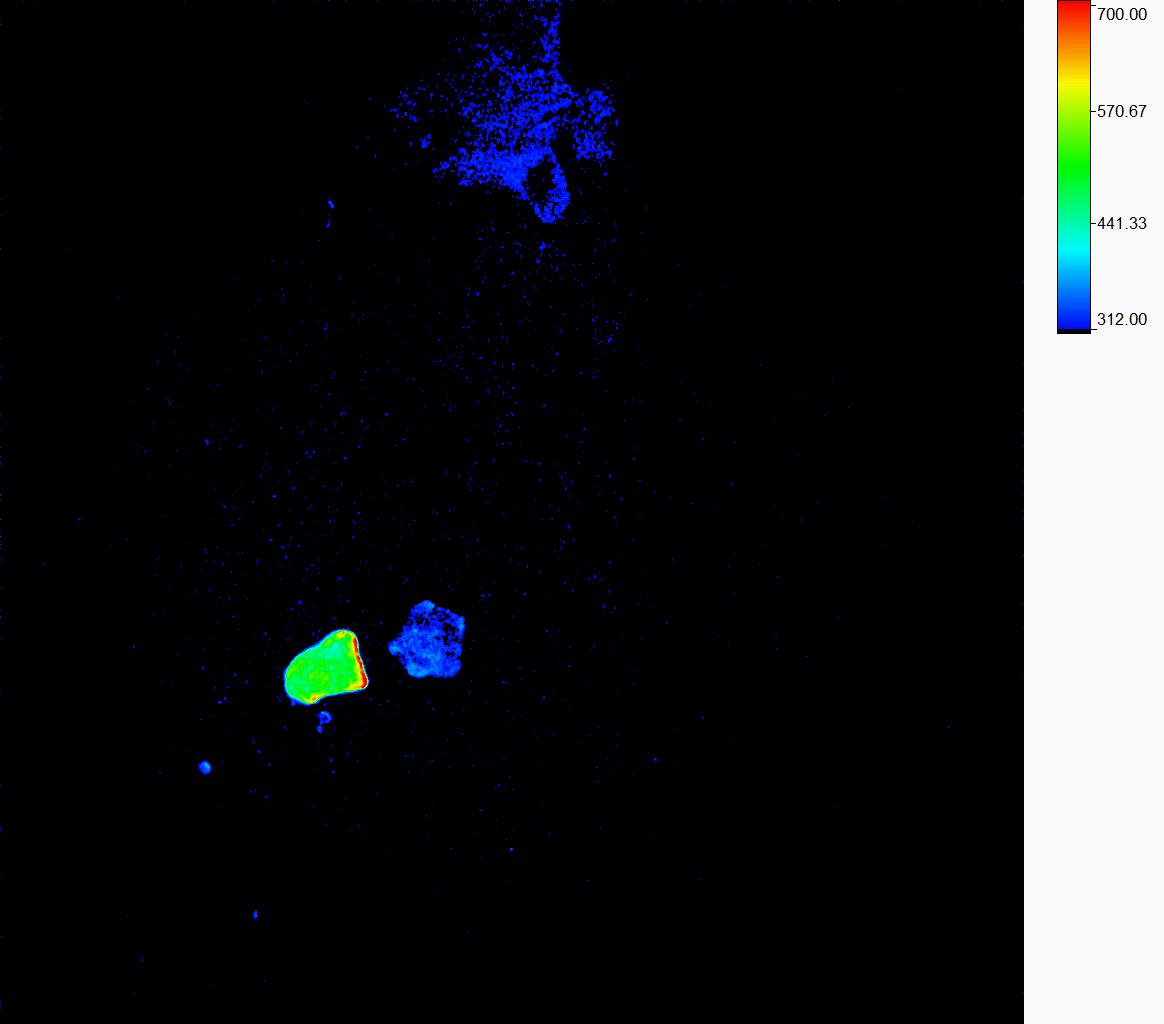

Supplement: Figure 5—source data 1. [file elife-70471-fig5-data1.zip › Figure 5-Source data/lung cancer patient 1/Raw data-nitroreductase detection image.jpg]

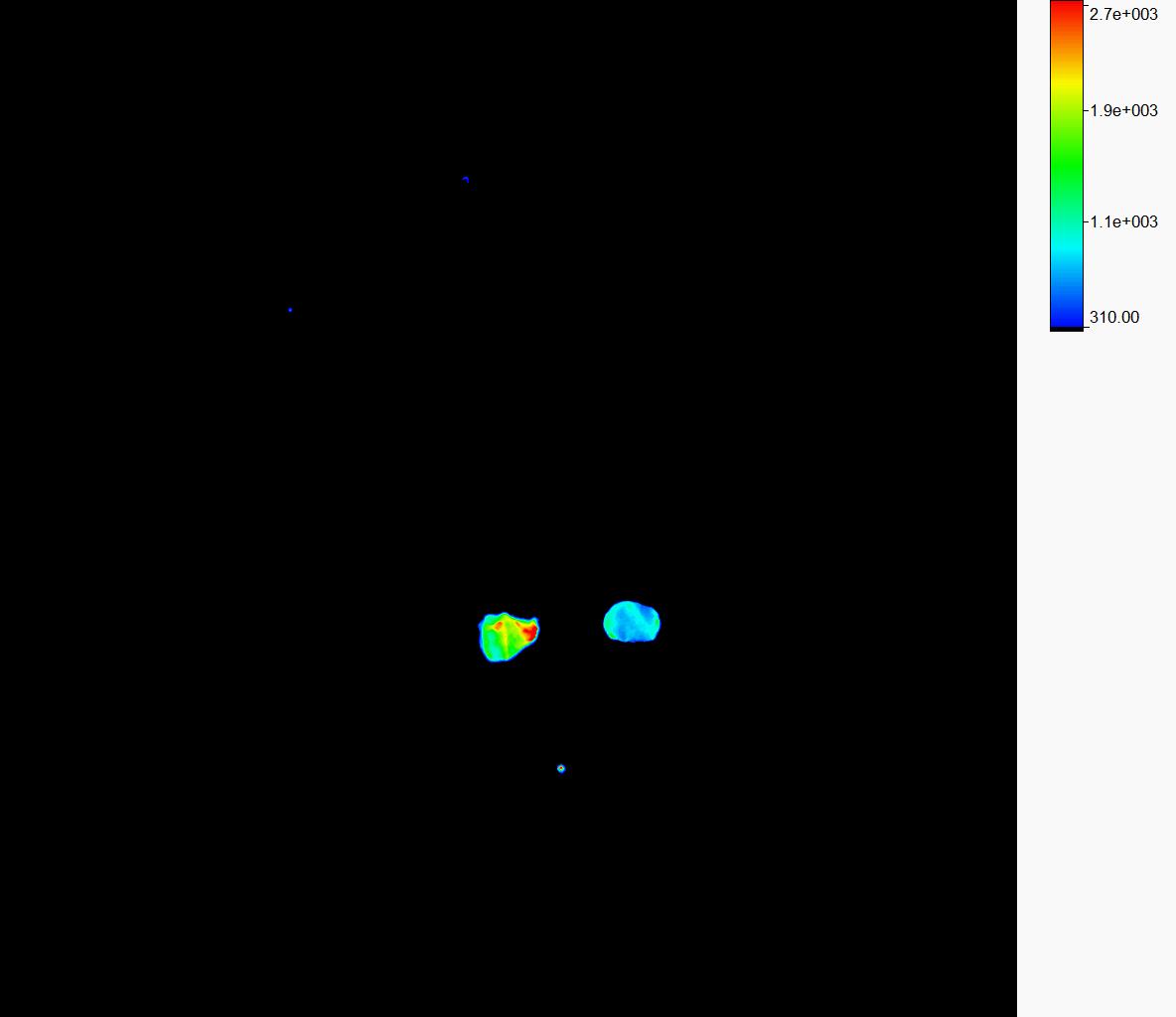

Supplement: Figure 5—figure supplement 2—source data 1. [file elife-70471-fig5-figsupp2-data1.zip › Figure 5-figure supplement 2-Source data 1/oral cancer patient 3/Raw data-viscosity detection image.jpg]

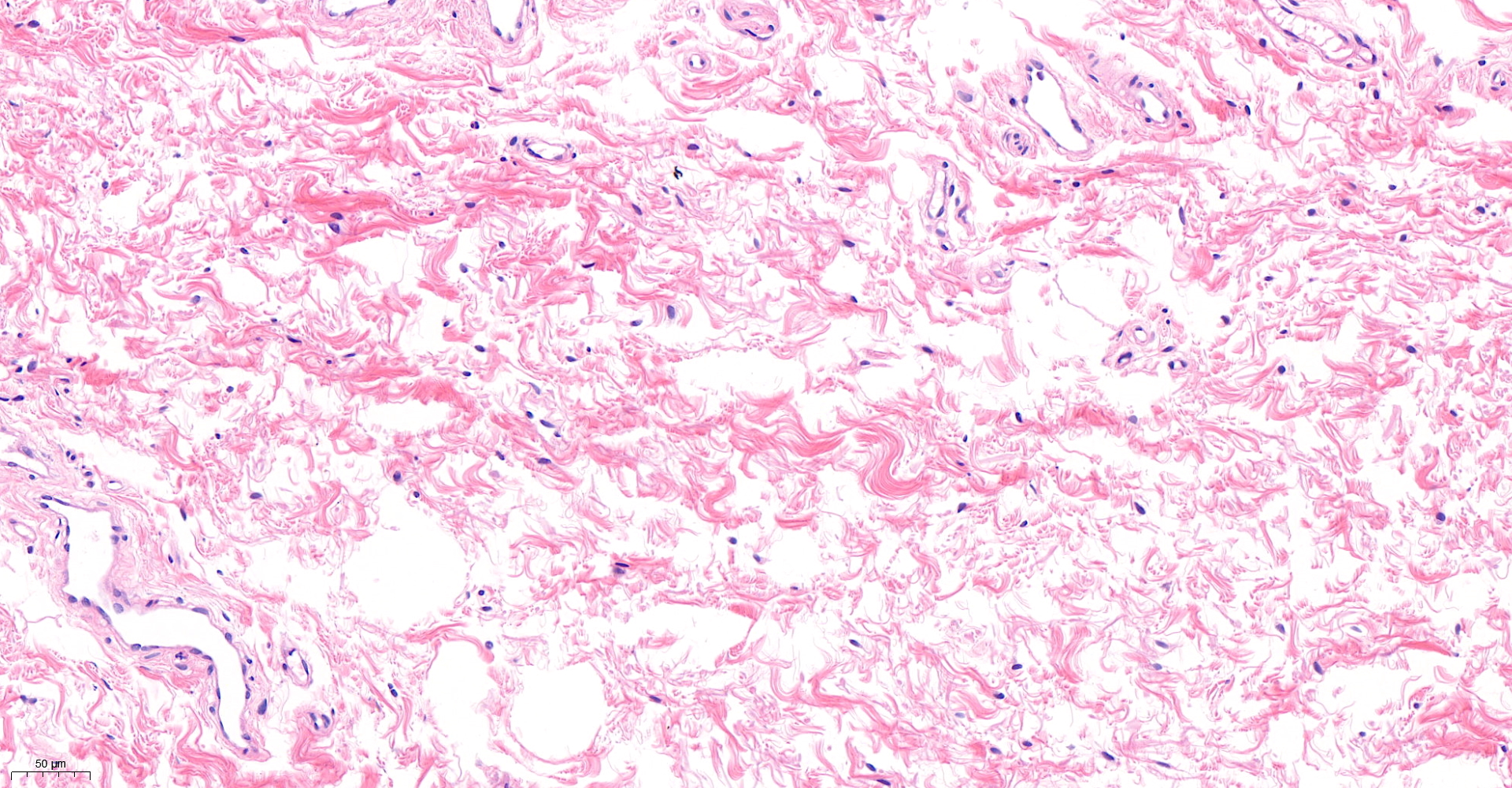

Supplement: Figure 5—figure supplement 2—source data 1. [file elife-70471-fig5-figsupp2-data1.zip › Figure 5-figure supplement 2-Source data 1/oral cancer patient 3/Raw data-HE staining image 1 of patient 3-20.0x.jpg]

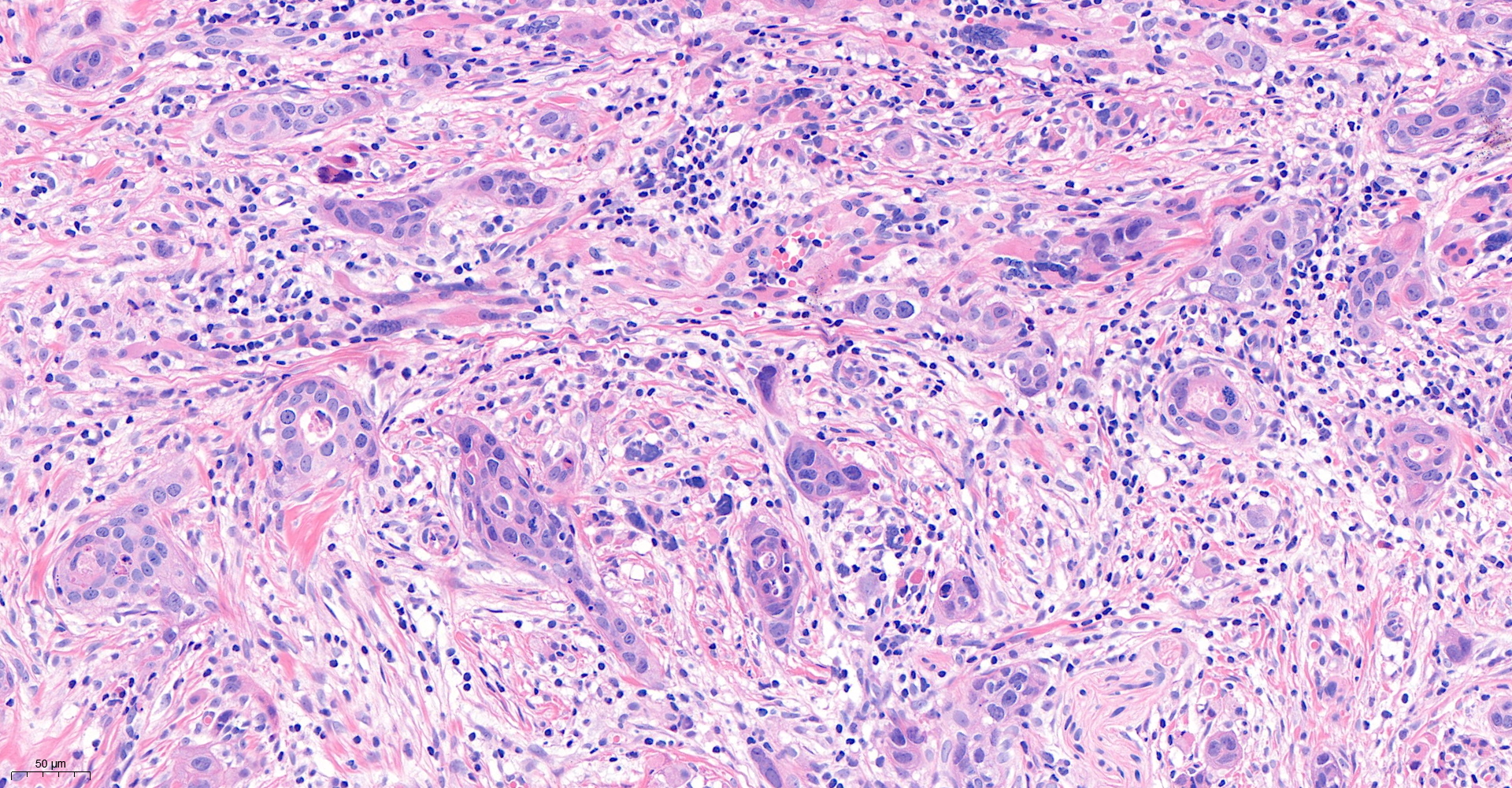

Supplement: Figure 5—figure supplement 2—source data 1. [file elife-70471-fig5-figsupp2-data1.zip › Figure 5-figure supplement 2-Source data 1/oral cancer patient 3/Raw data-HE staining image 2 of patient 3-20.0x.jpg]

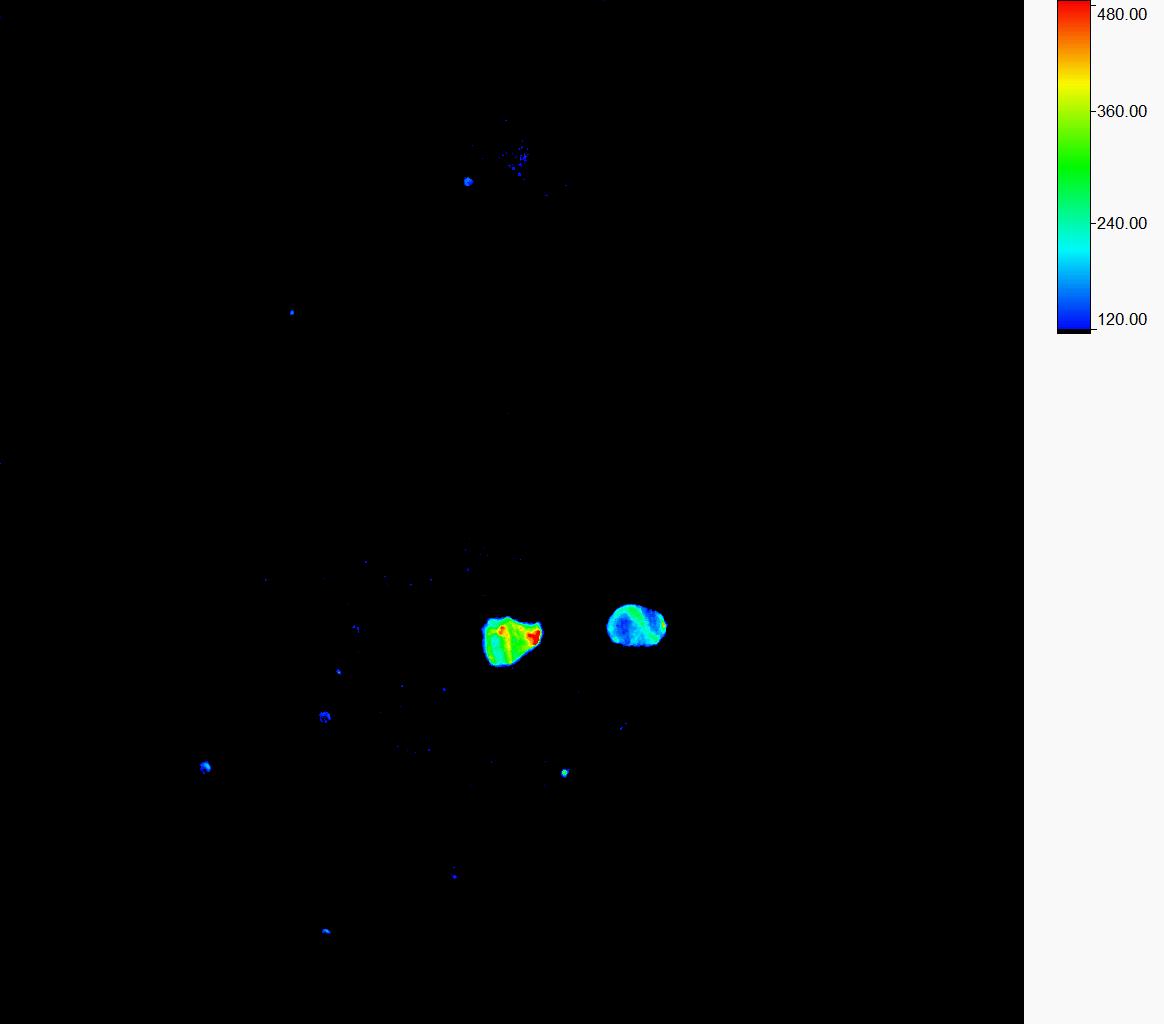

Supplement: Figure 5—figure supplement 2—source data 1. [file elife-70471-fig5-figsupp2-data1.zip › Figure 5-figure supplement 2-Source data 1/oral cancer patient 3/Raw data-nitroreductase detection image.jpg]

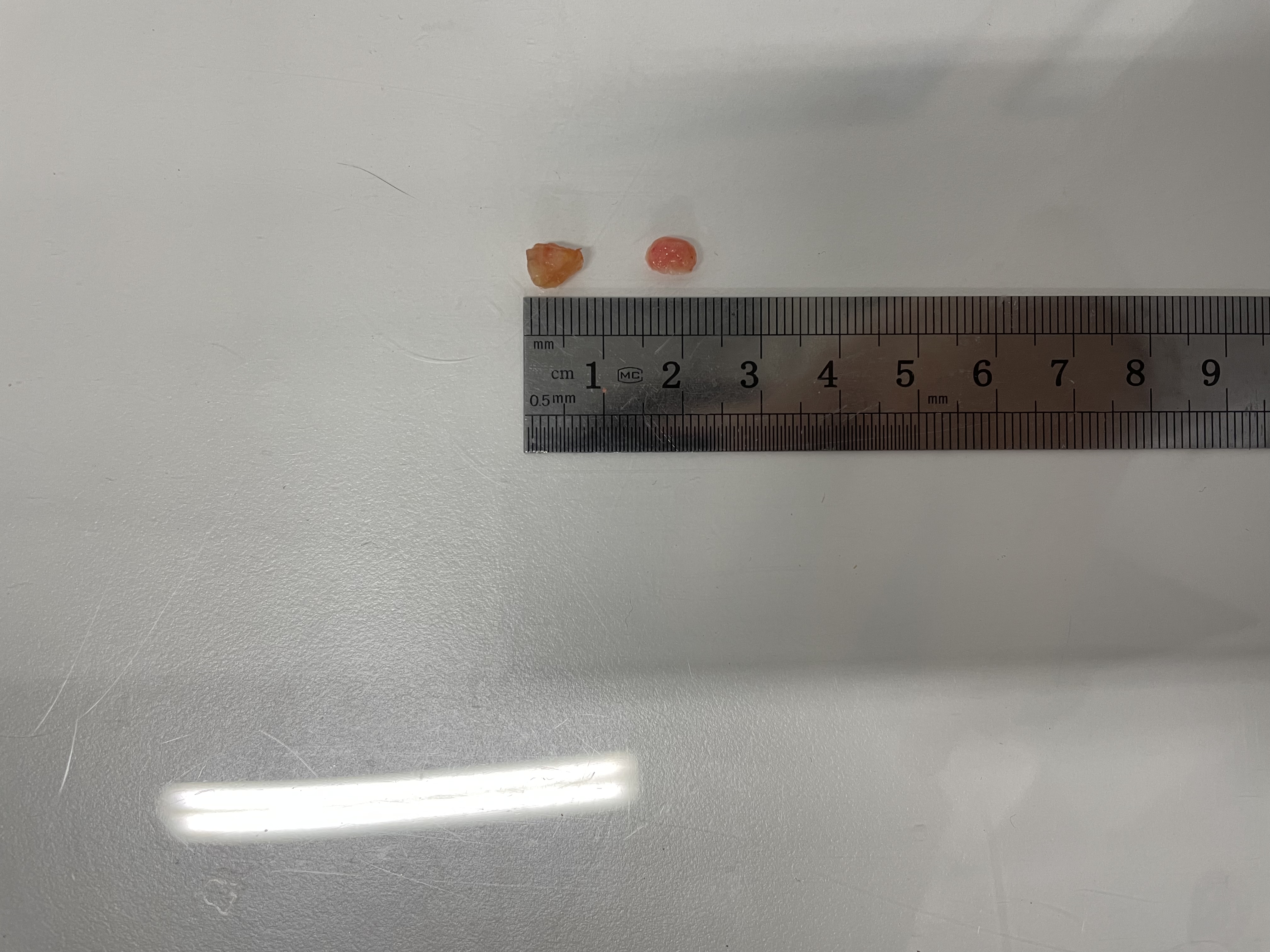

Supplement: Figure 5—figure supplement 2—source data 1. [file elife-70471-fig5-figsupp2-data1.zip › Figure 5-figure supplement 2-Source data 1/oral cancer patient 3/Raw data-photograph image.jpeg]

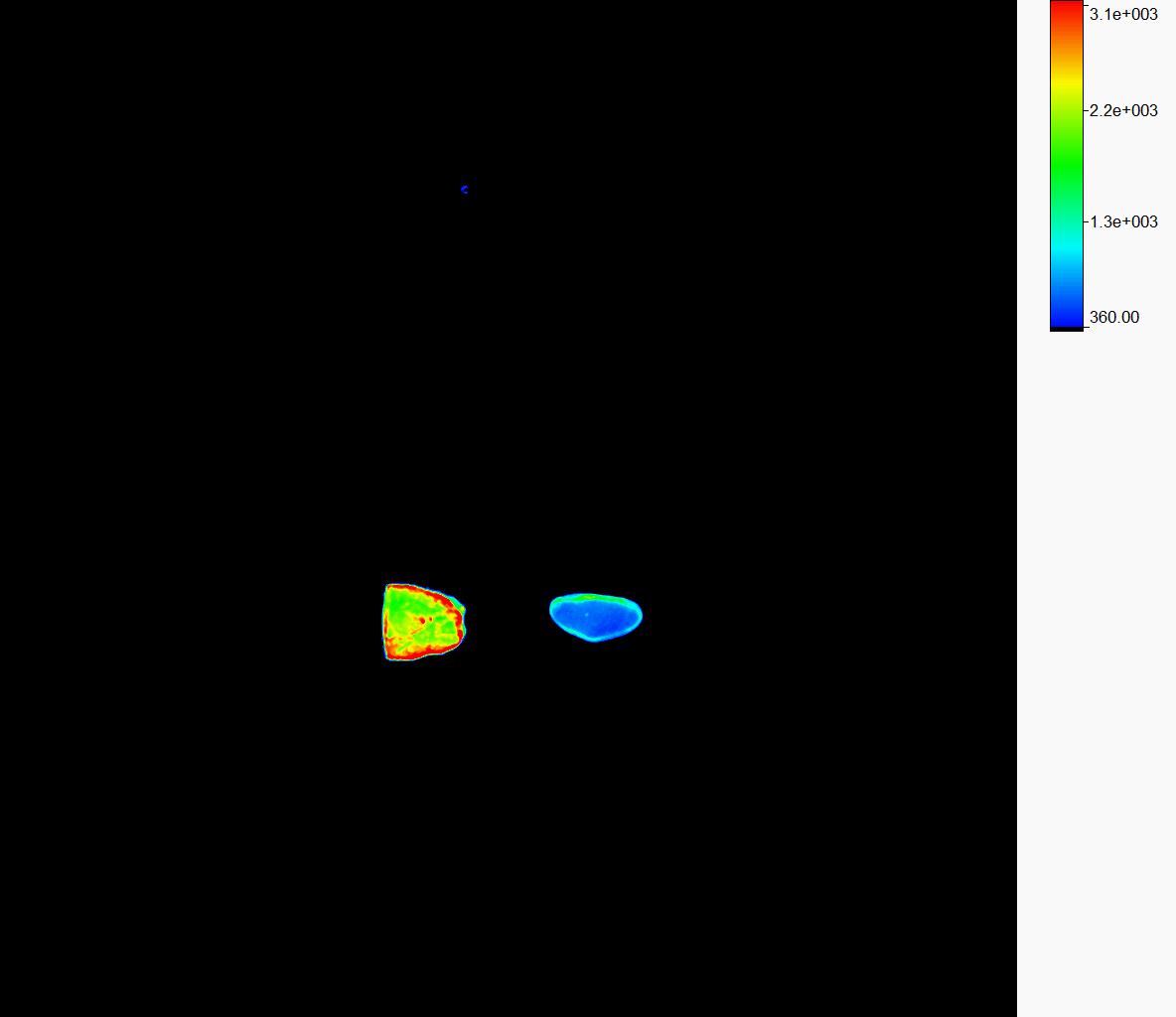

Supplement: Figure 5—figure supplement 2—source data 1. [file elife-70471-fig5-figsupp2-data1.zip › Figure 5-figure supplement 2-Source data 1/oral cancer patient 4/Raw data-viscosity detection image.jpg]

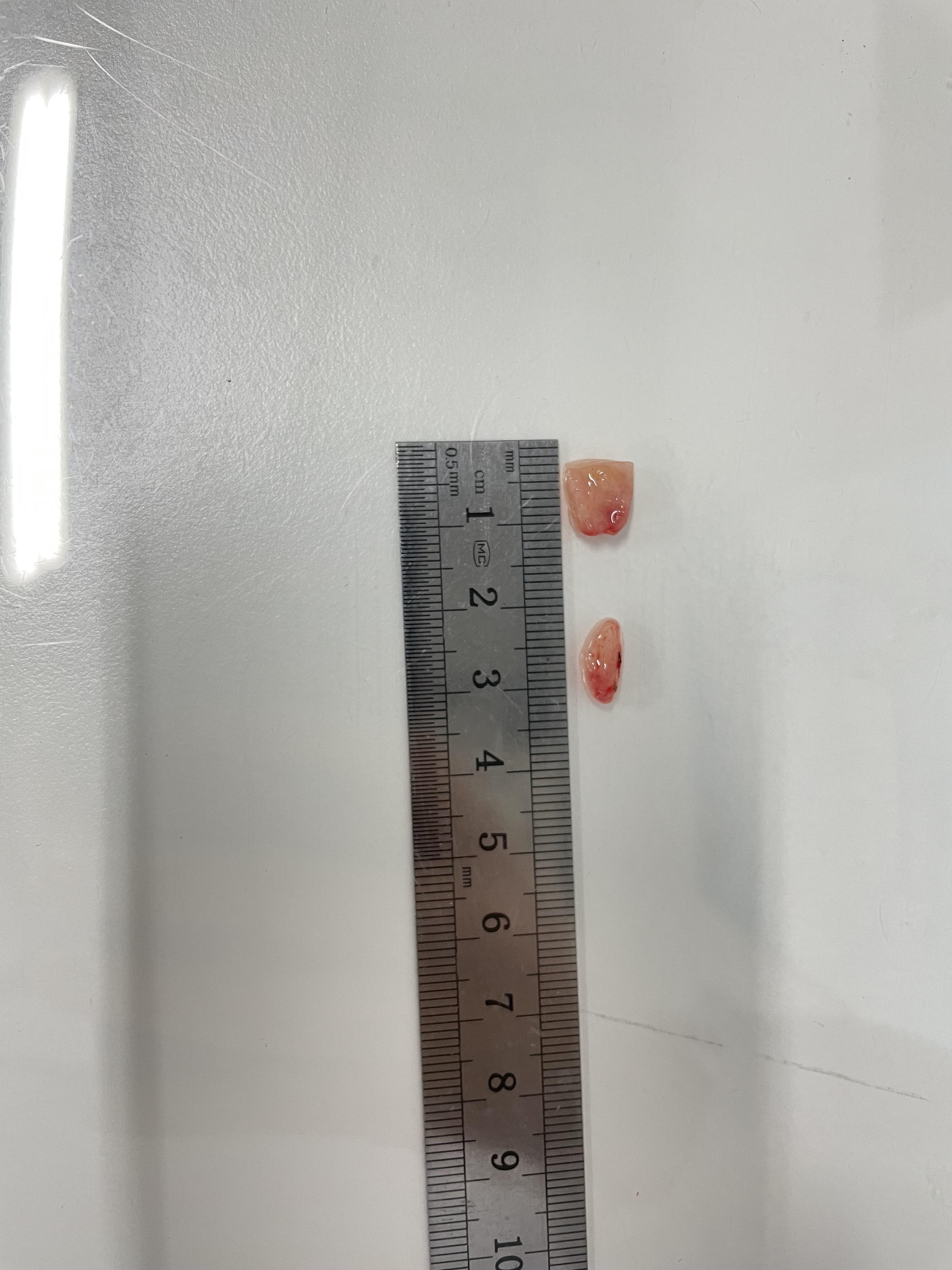

Supplement: Figure 5—figure supplement 2—source data 1. [file elife-70471-fig5-figsupp2-data1.zip › Figure 5-figure supplement 2-Source data 1/oral cancer patient 4/Raw data-photograph image.JPG]

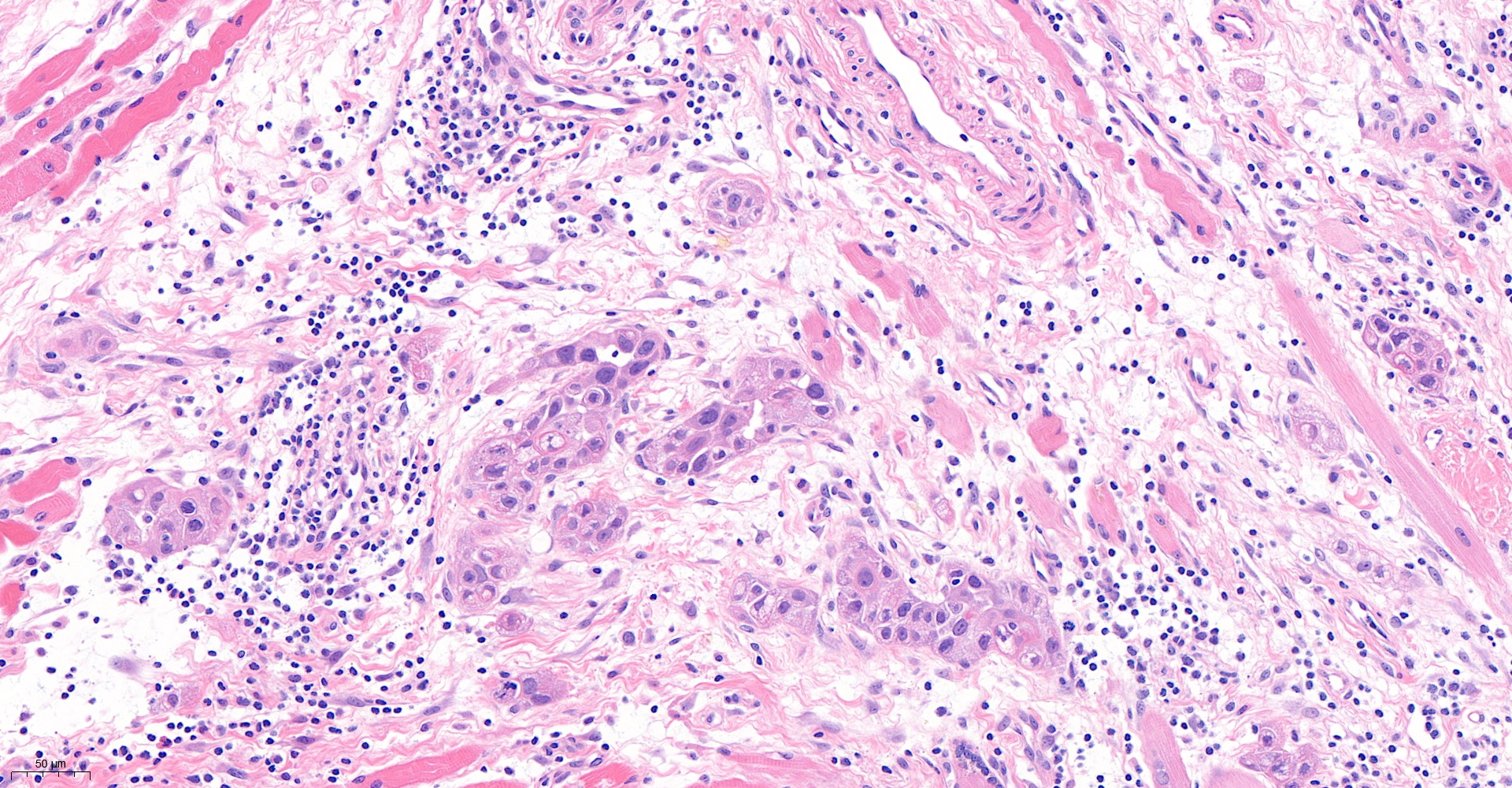

Supplement: Figure 5—figure supplement 2—source data 1. [file elife-70471-fig5-figsupp2-data1.zip › Figure 5-figure supplement 2-Source data 1/oral cancer patient 4/Raw data-HE staining image 2 of patient 4-20.0x.jpg]

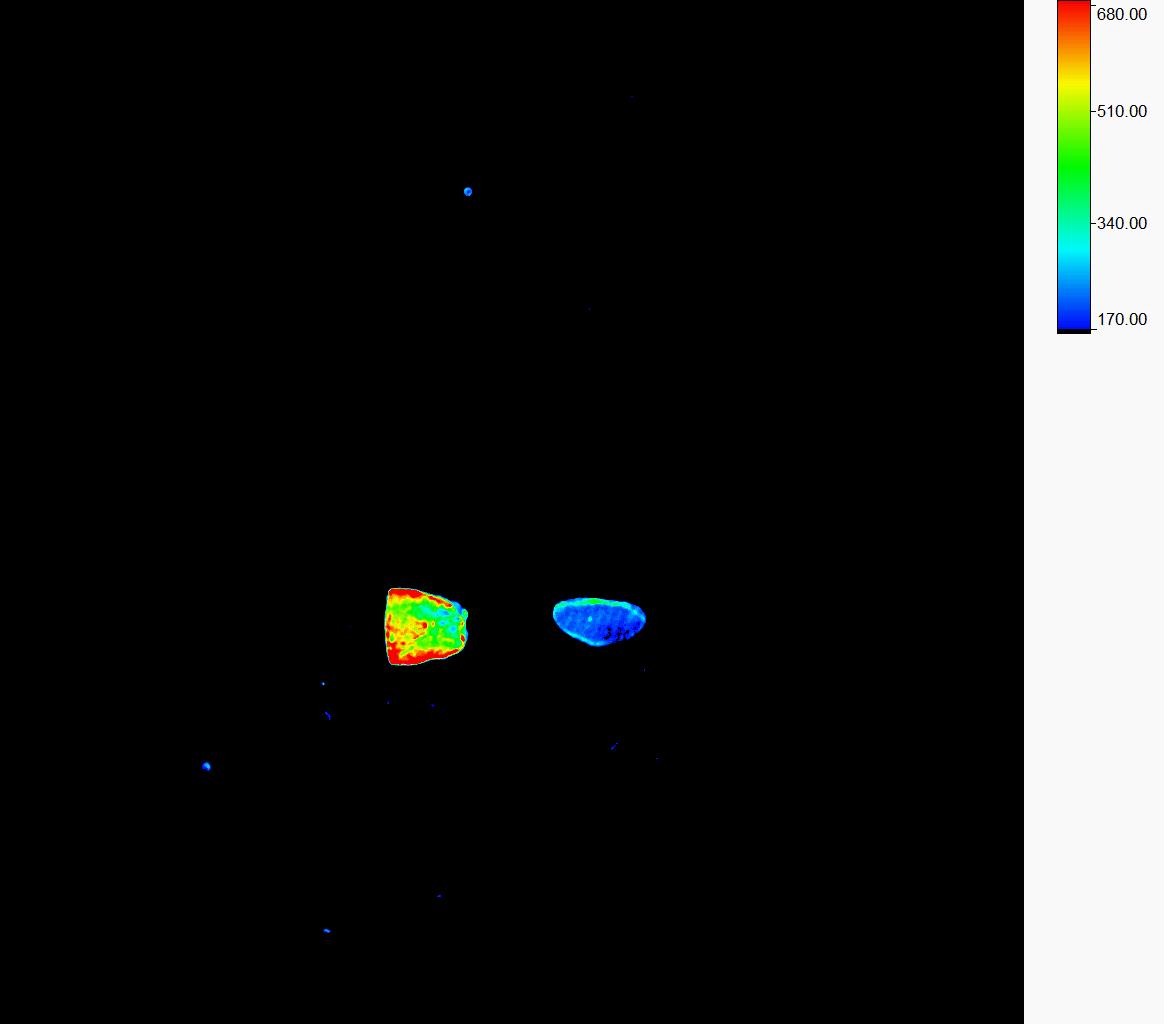

Supplement: Figure 5—figure supplement 2—source data 1. [file elife-70471-fig5-figsupp2-data1.zip › Figure 5-figure supplement 2-Source data 1/oral cancer patient 4/Raw data-nitroreductase detection image.jpg]

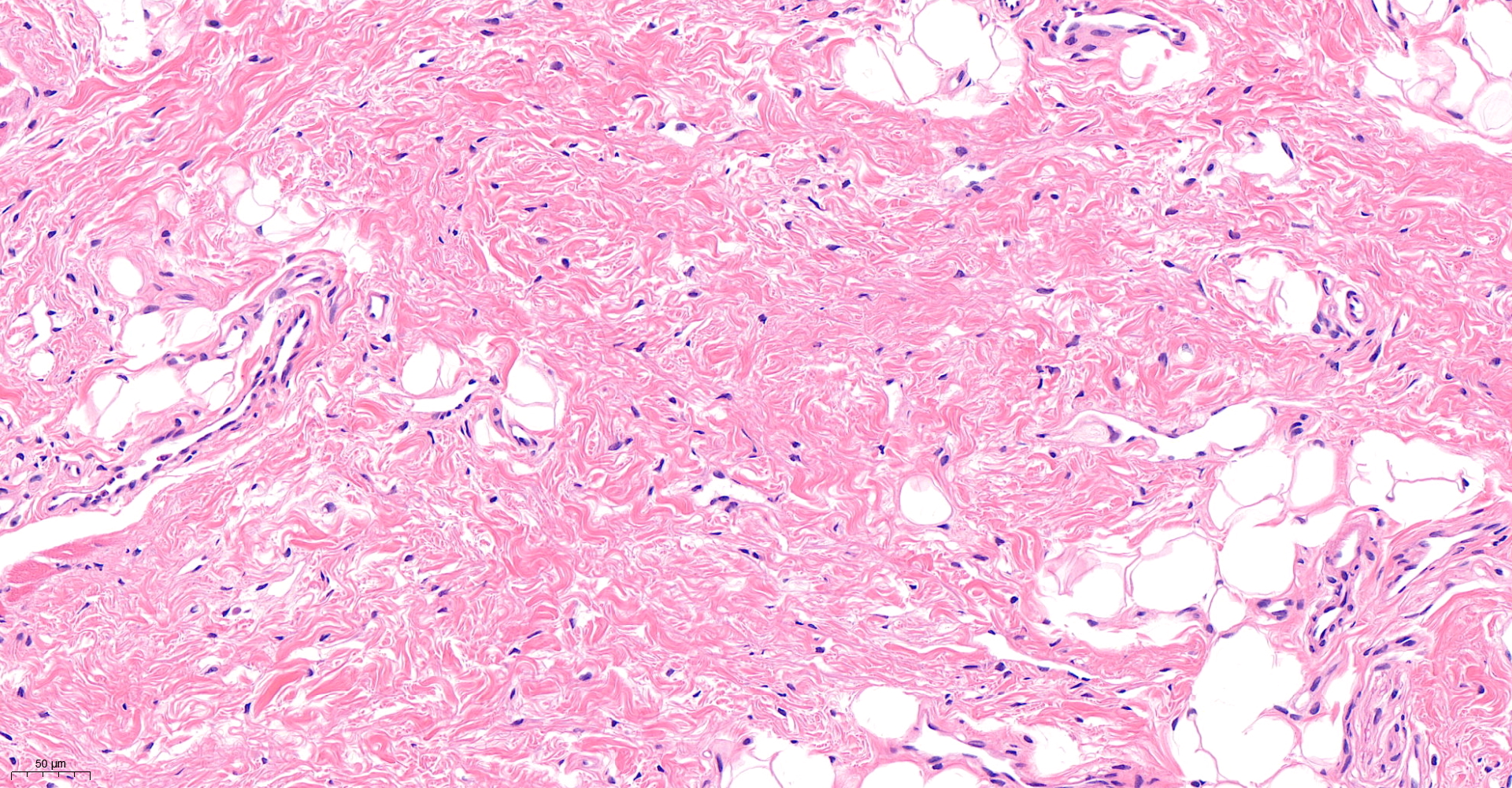

Supplement: Figure 5—figure supplement 2—source data 1. [file elife-70471-fig5-figsupp2-data1.zip › Figure 5-figure supplement 2-Source data 1/oral cancer patient 4/Raw data-HE staining image 1 of patient 4-20.0x.jpg]

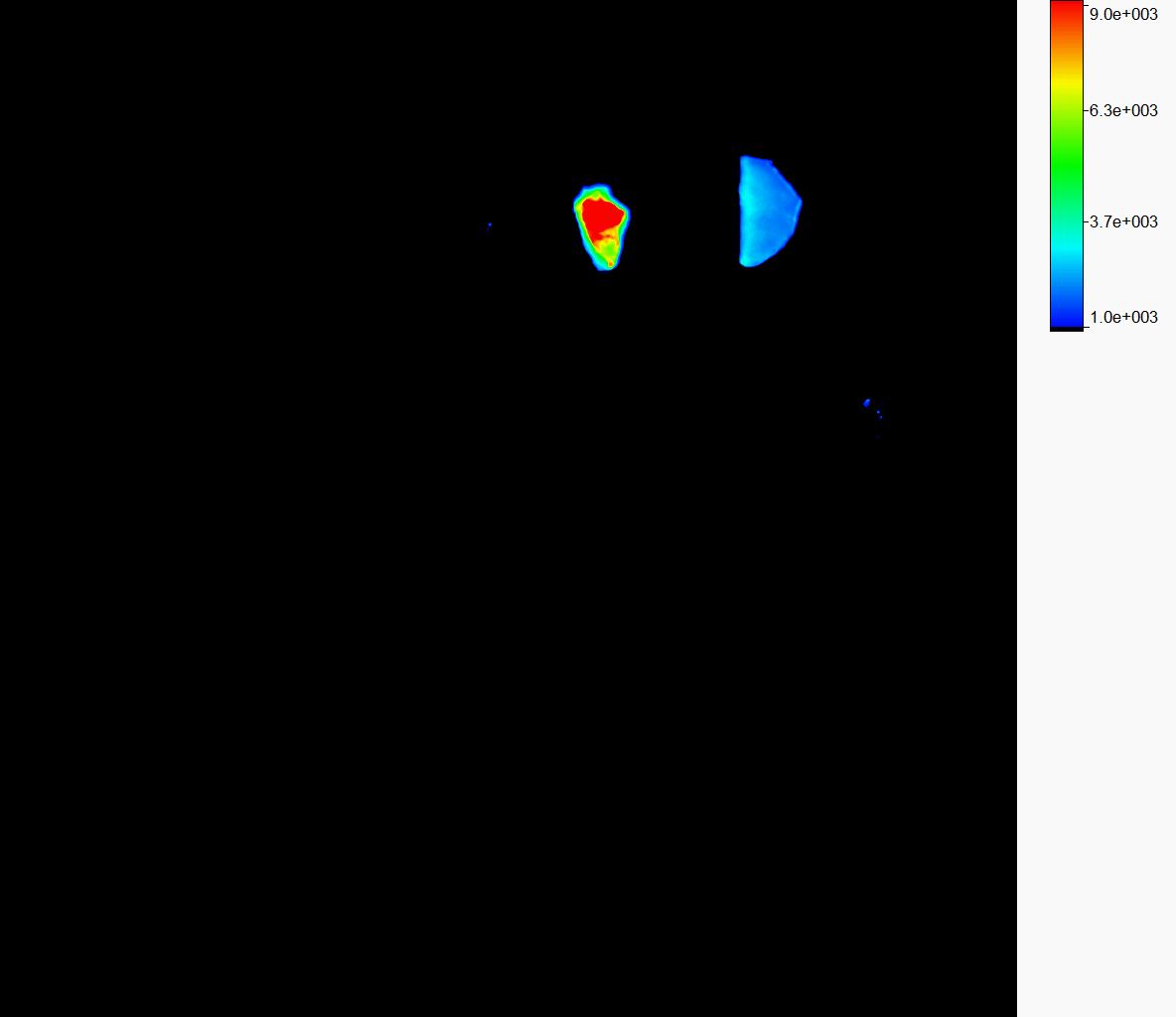

Supplement: Figure 5—figure supplement 2—source data 1. [file elife-70471-fig5-figsupp2-data1.zip › Figure 5-figure supplement 2-Source data 1/oral cancer patient 5/Raw data-viscosity detection image.jpg]

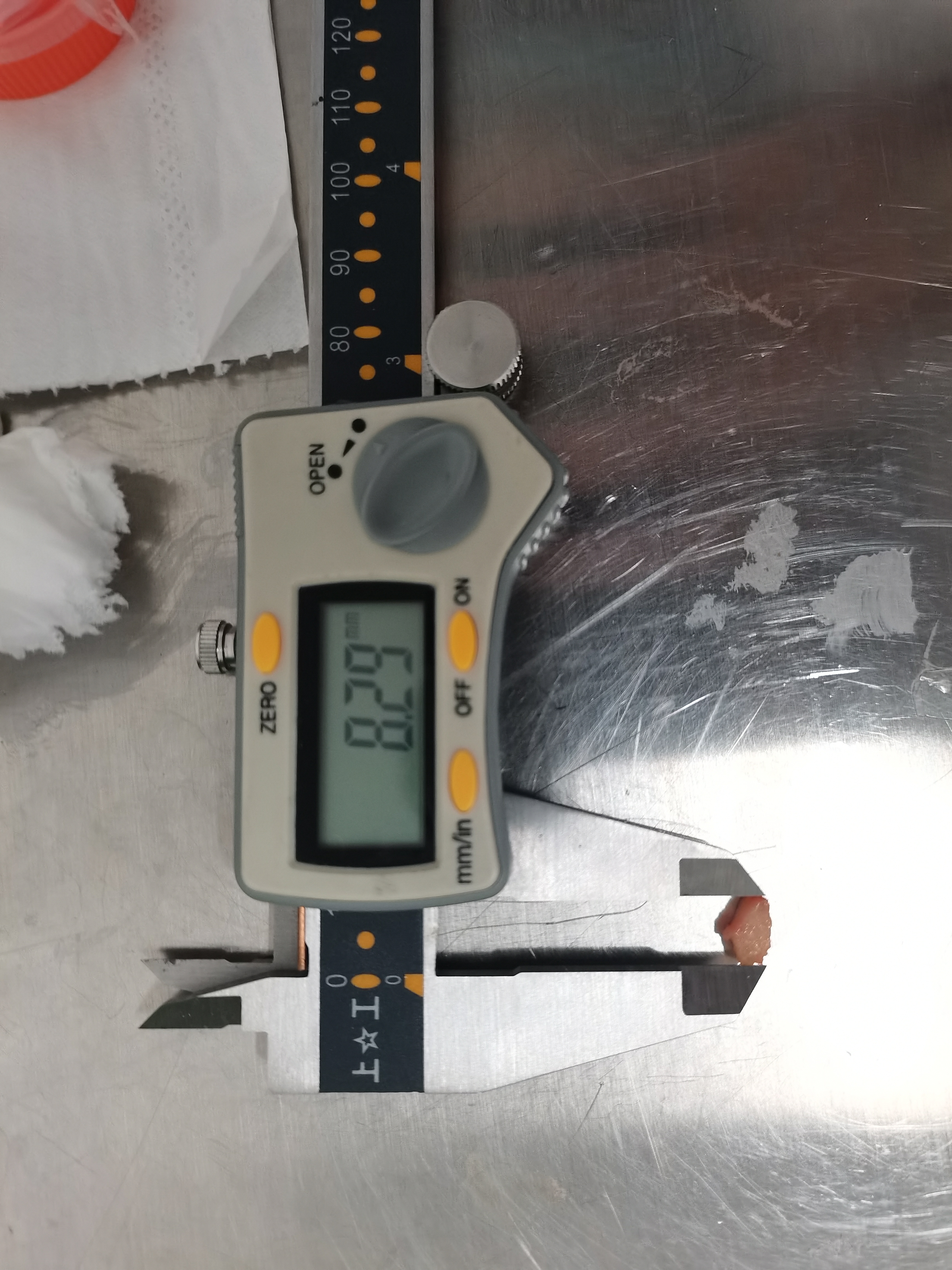

Supplement: Figure 5—figure supplement 2—source data 1. [file elife-70471-fig5-figsupp2-data1.zip › Figure 5-figure supplement 2-Source data 1/oral cancer patient 5/Raw data-photograph image 2.jpg]

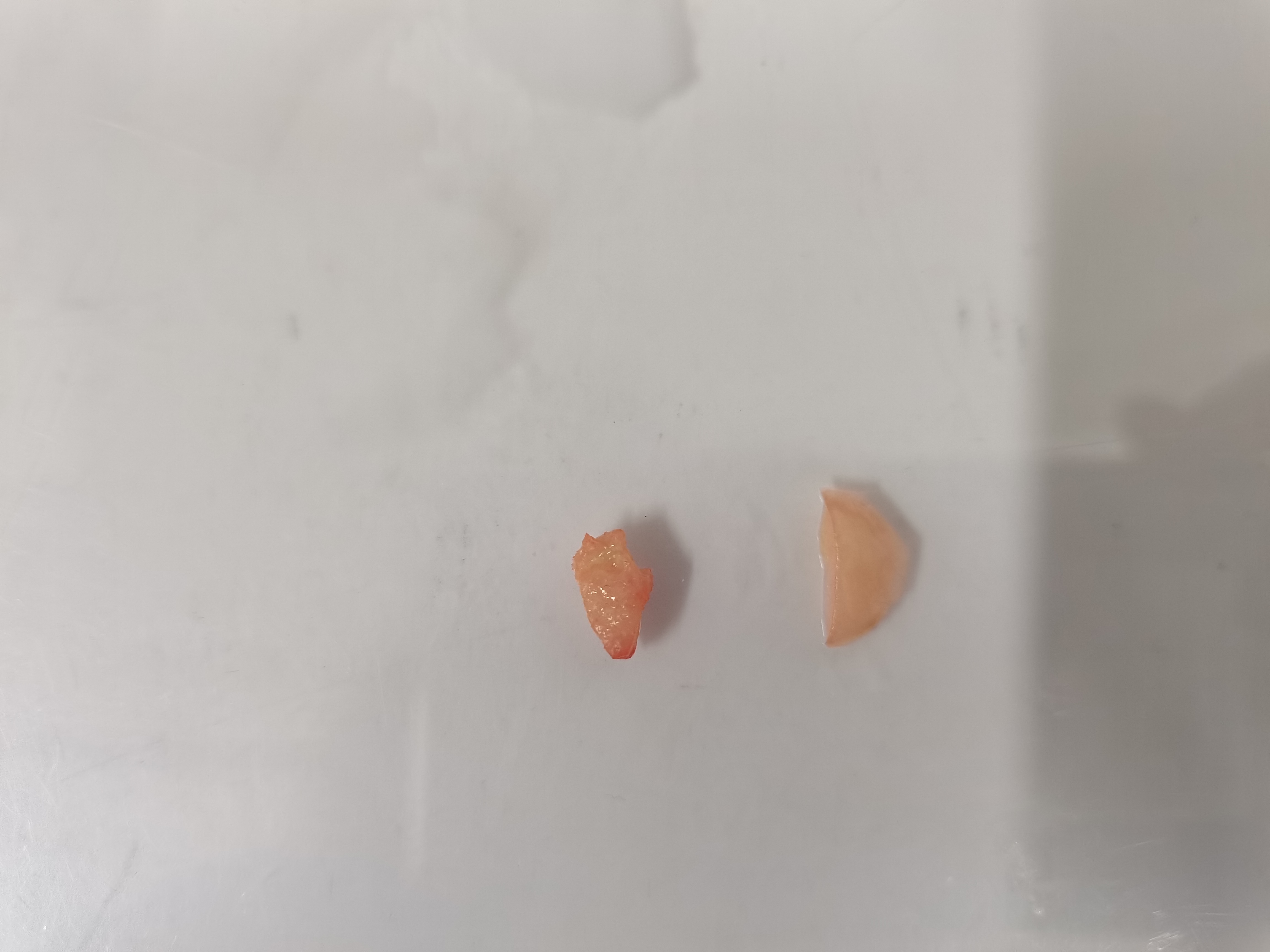

Supplement: Figure 5—figure supplement 2—source data 1. [file elife-70471-fig5-figsupp2-data1.zip › Figure 5-figure supplement 2-Source data 1/oral cancer patient 5/Raw data-photograph image 1.jpg]

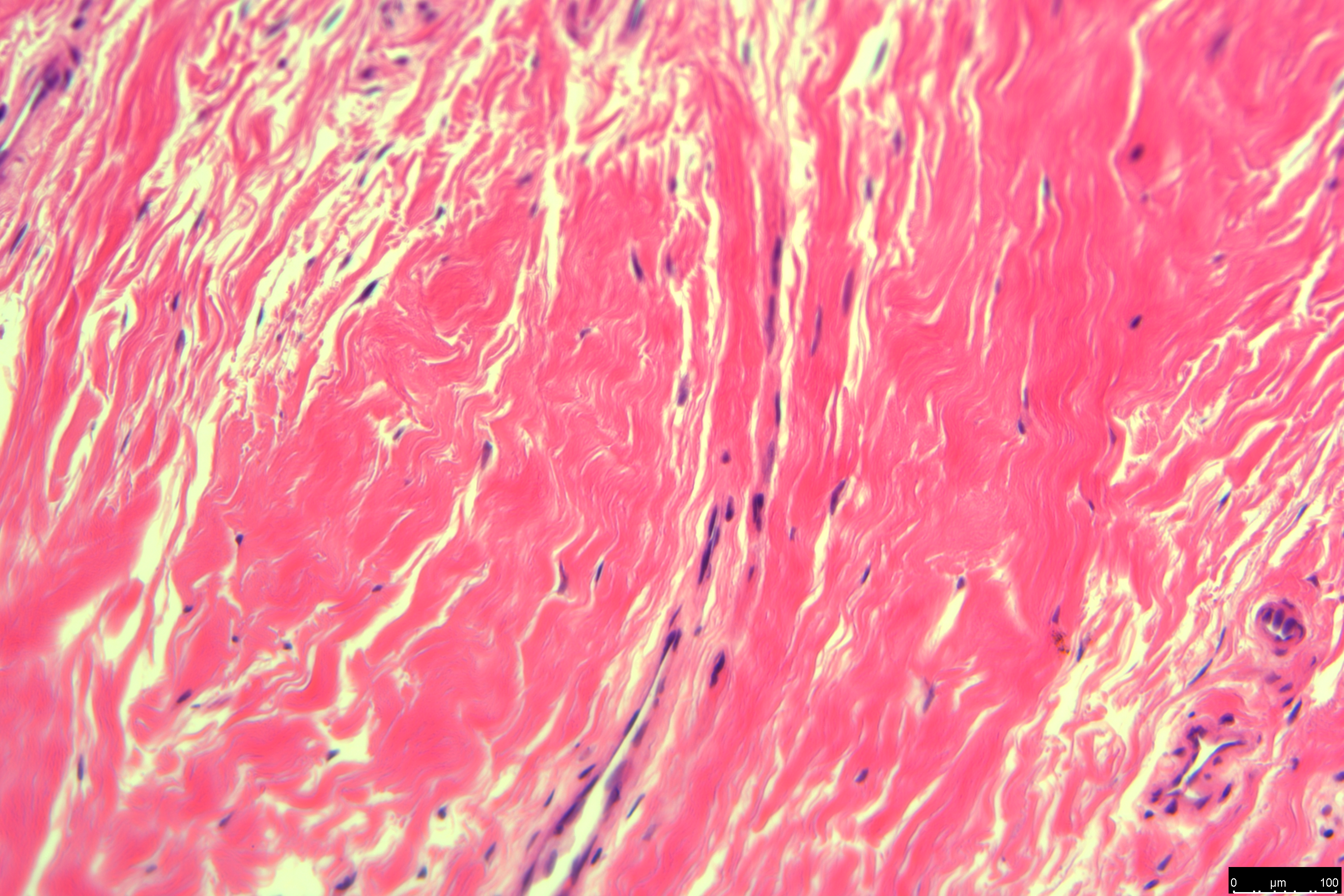

Supplement: Figure 5—figure supplement 2—source data 1. [file elife-70471-fig5-figsupp2-data1.zip › Figure 5-figure supplement 2-Source data 1/oral cancer patient 5/Raw data-HE staining image 1 of patient 5-20.0x.tif]

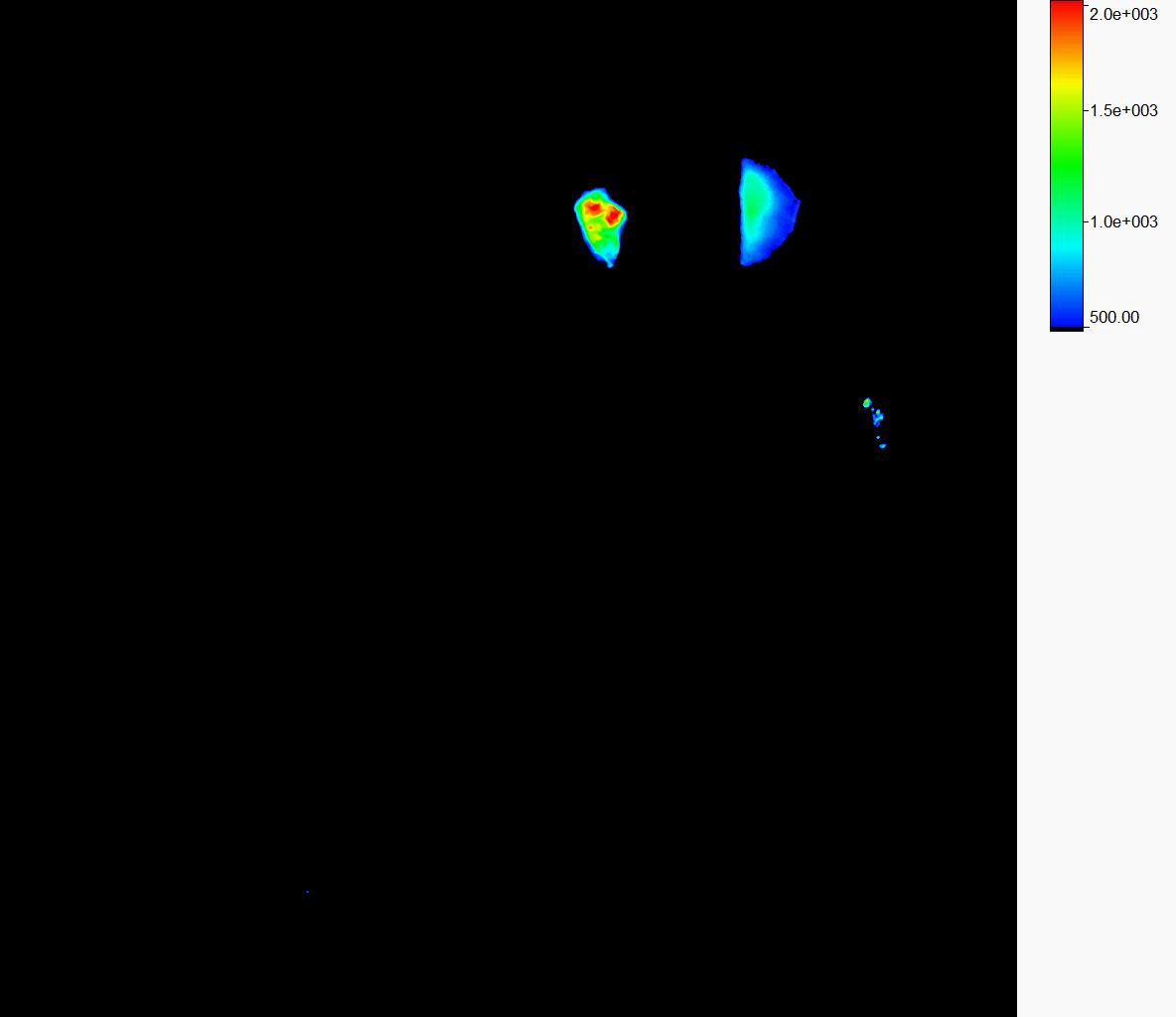

Supplement: Figure 5—figure supplement 2—source data 1. [file elife-70471-fig5-figsupp2-data1.zip › Figure 5-figure supplement 2-Source data 1/oral cancer patient 5/Raw data-nitroreductase detection image.jpg]

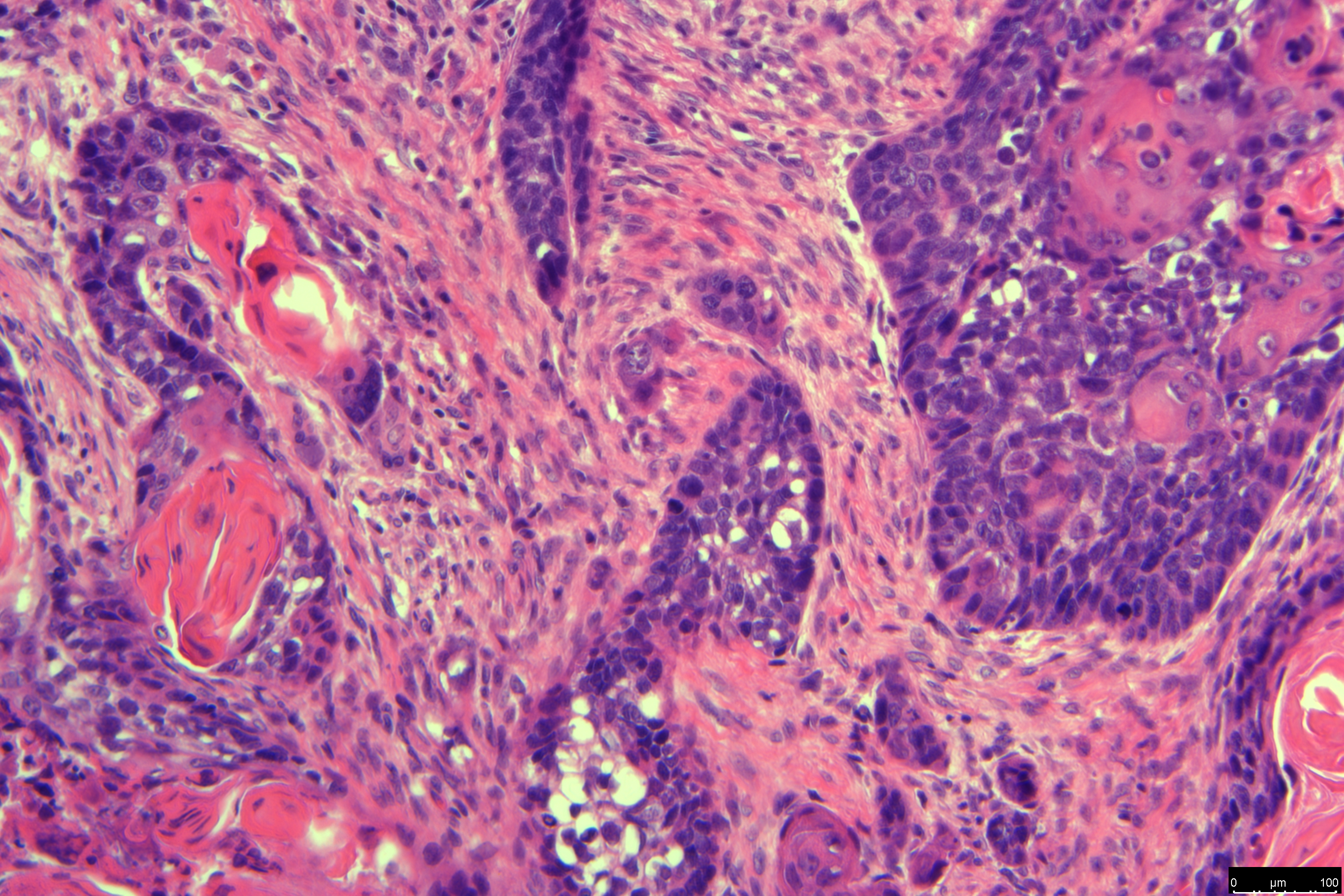

Supplement: Figure 5—figure supplement 2—source data 1. [file elife-70471-fig5-figsupp2-data1.zip › Figure 5-figure supplement 2-Source data 1/oral cancer patient 5/Raw data-HE staining image 2 of patient 5-20.0x.tif]

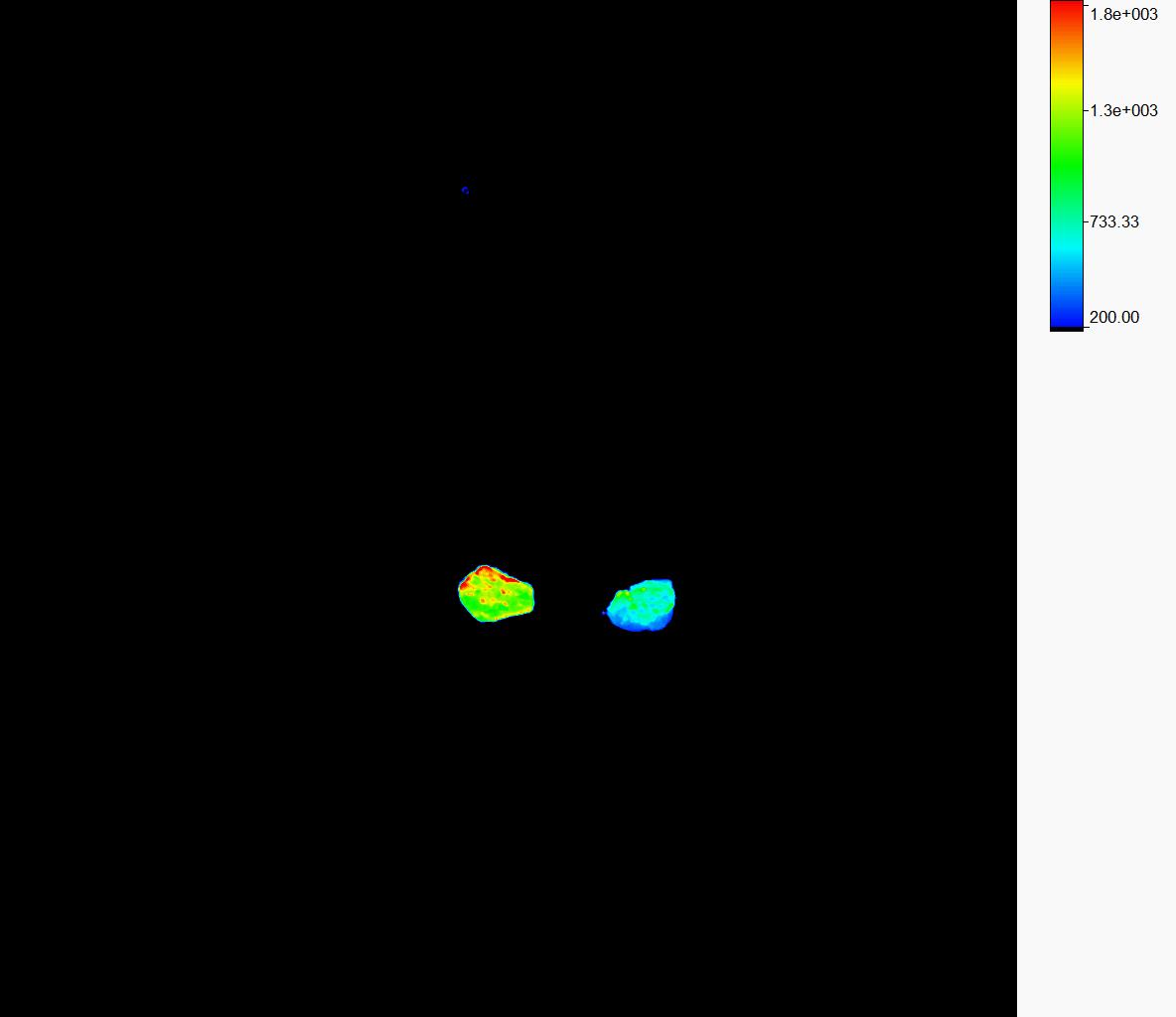

Supplement: Figure 5—figure supplement 2—source data 1. [file elife-70471-fig5-figsupp2-data1.zip › Figure 5-figure supplement 2-Source data 1/oral cancer patient 2/Raw data-viscosity detection image.jpg]

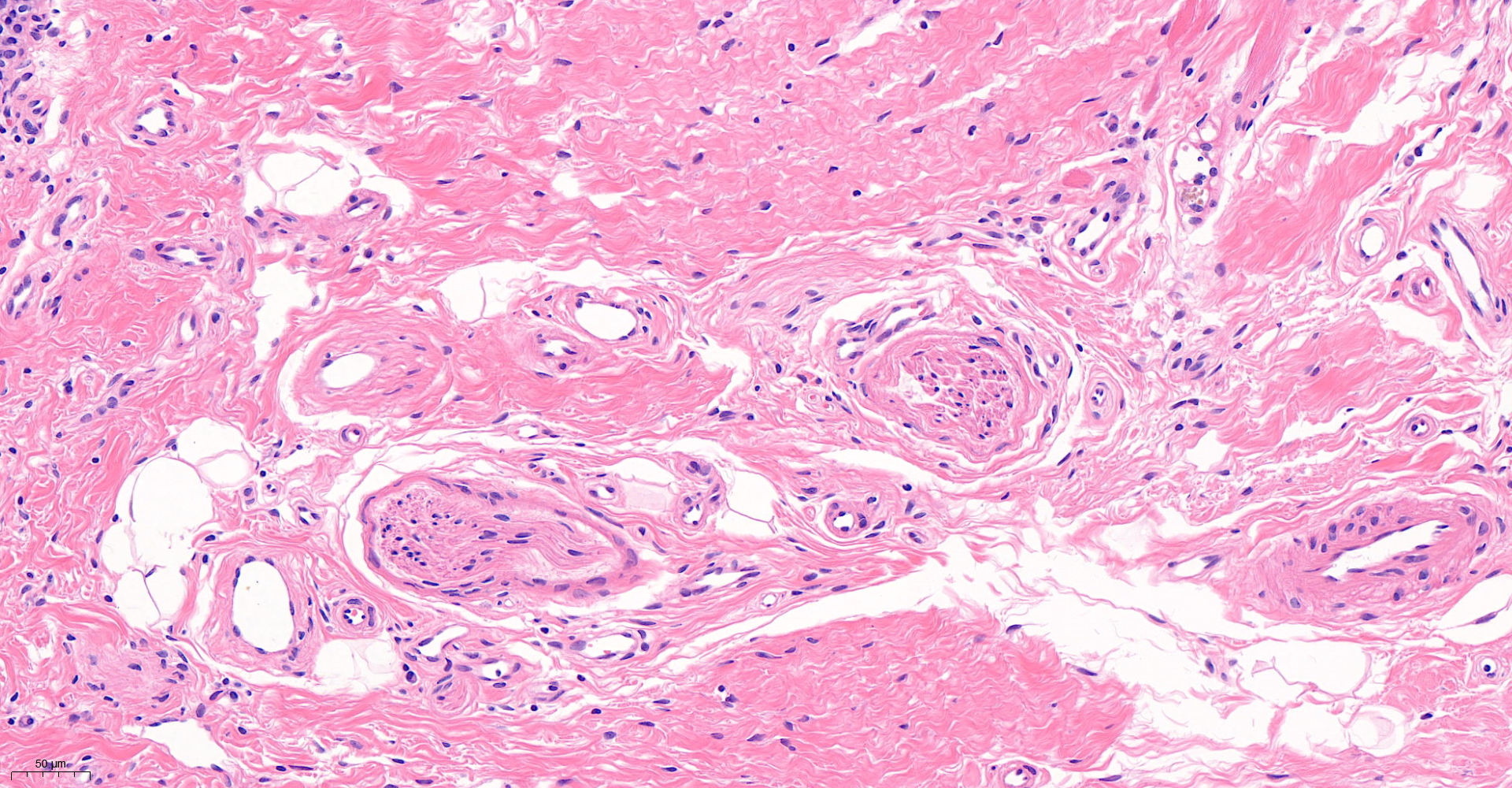

Supplement: Figure 5—figure supplement 2—source data 1. [file elife-70471-fig5-figsupp2-data1.zip › Figure 5-figure supplement 2-Source data 1/oral cancer patient 2/Raw data-HE staining image 1 of patient 2-20.0x.jpg]

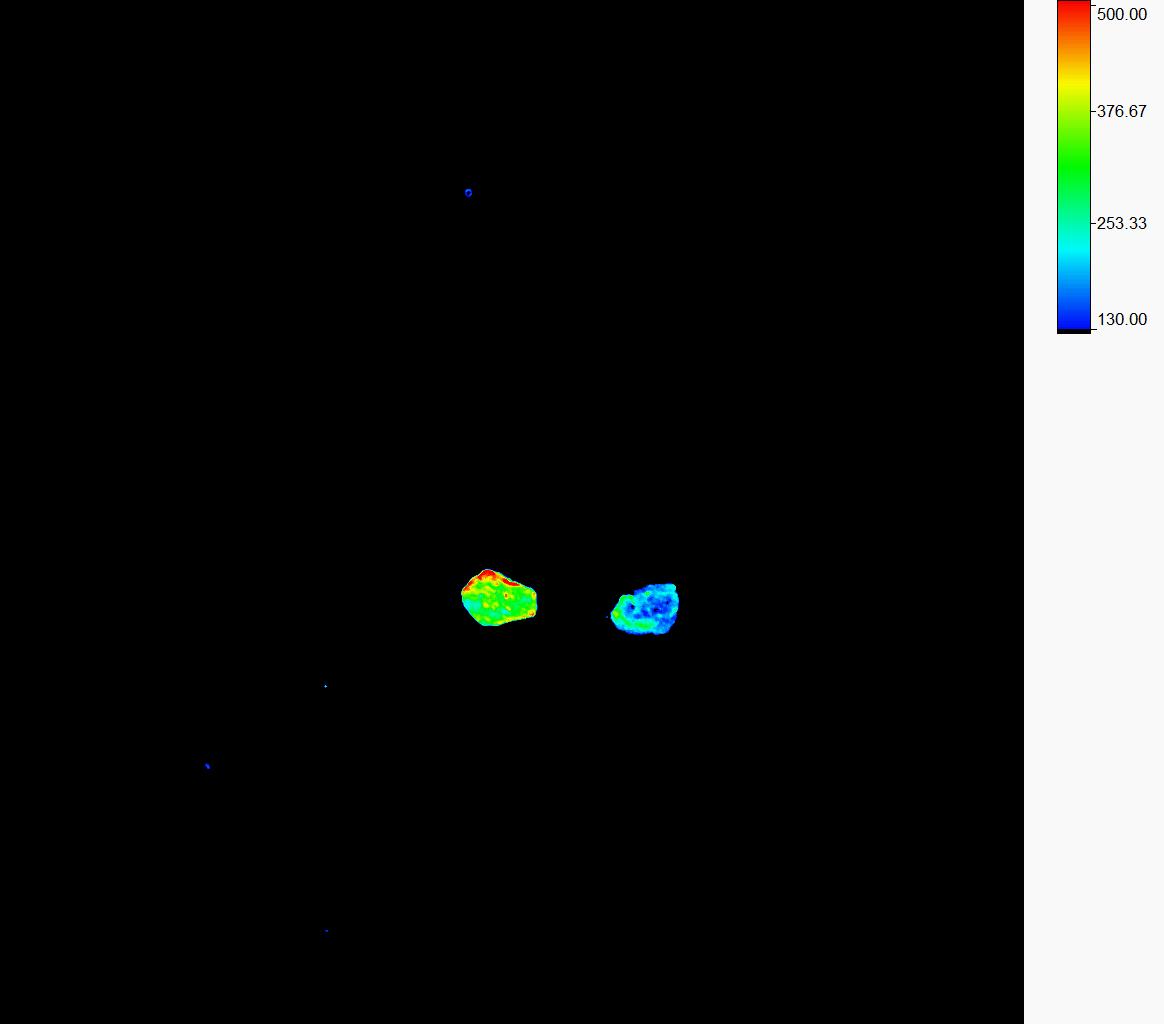

Supplement: Figure 5—figure supplement 2—source data 1. [file elife-70471-fig5-figsupp2-data1.zip › Figure 5-figure supplement 2-Source data 1/oral cancer patient 2/Raw data-nitroreductase detection image.jpg]

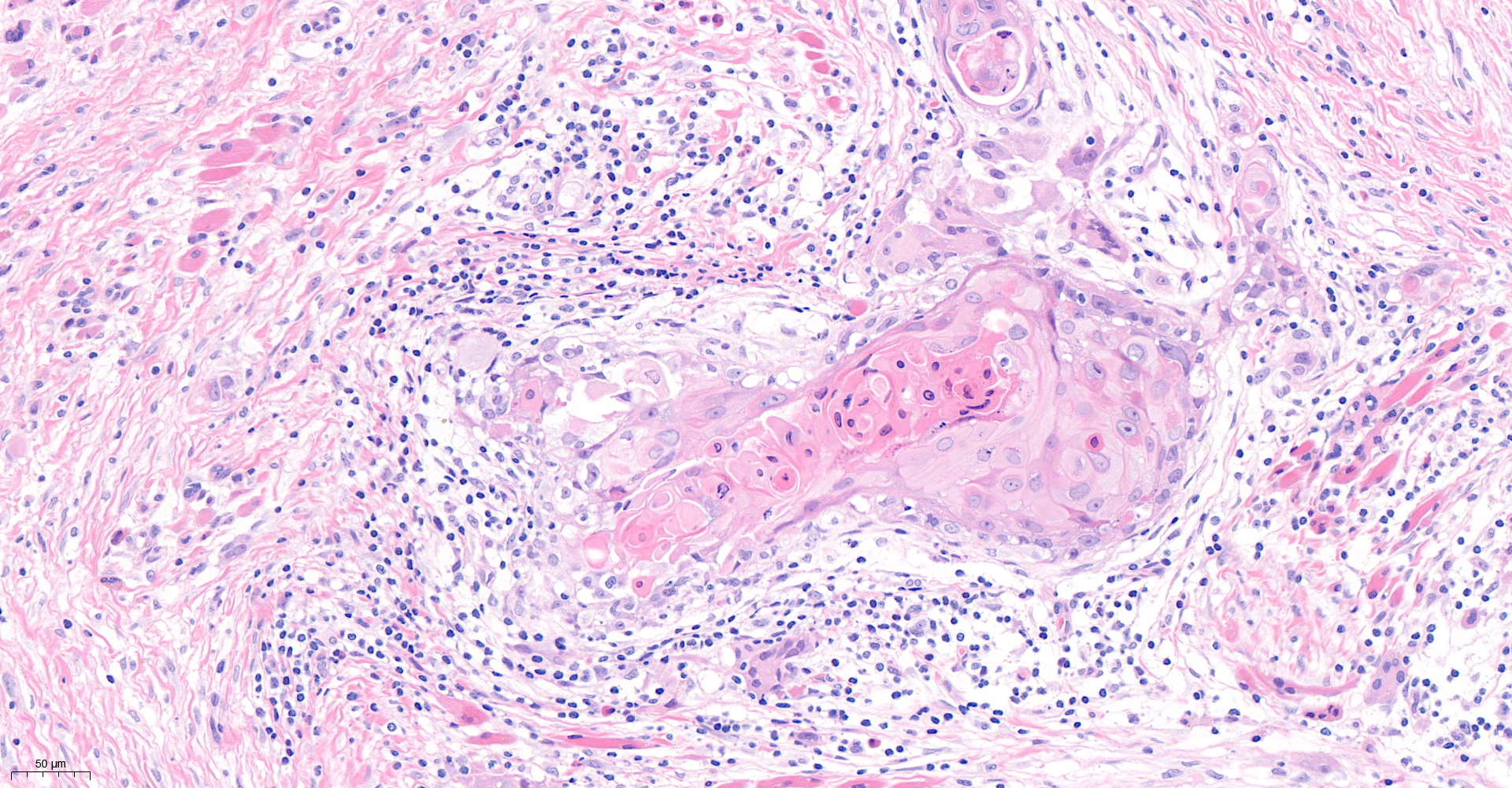

Supplement: Figure 5—figure supplement 2—source data 1. [file elife-70471-fig5-figsupp2-data1.zip › Figure 5-figure supplement 2-Source data 1/oral cancer patient 2/Raw data-HE staining image 2 of patient 2-20.0x.jpg]

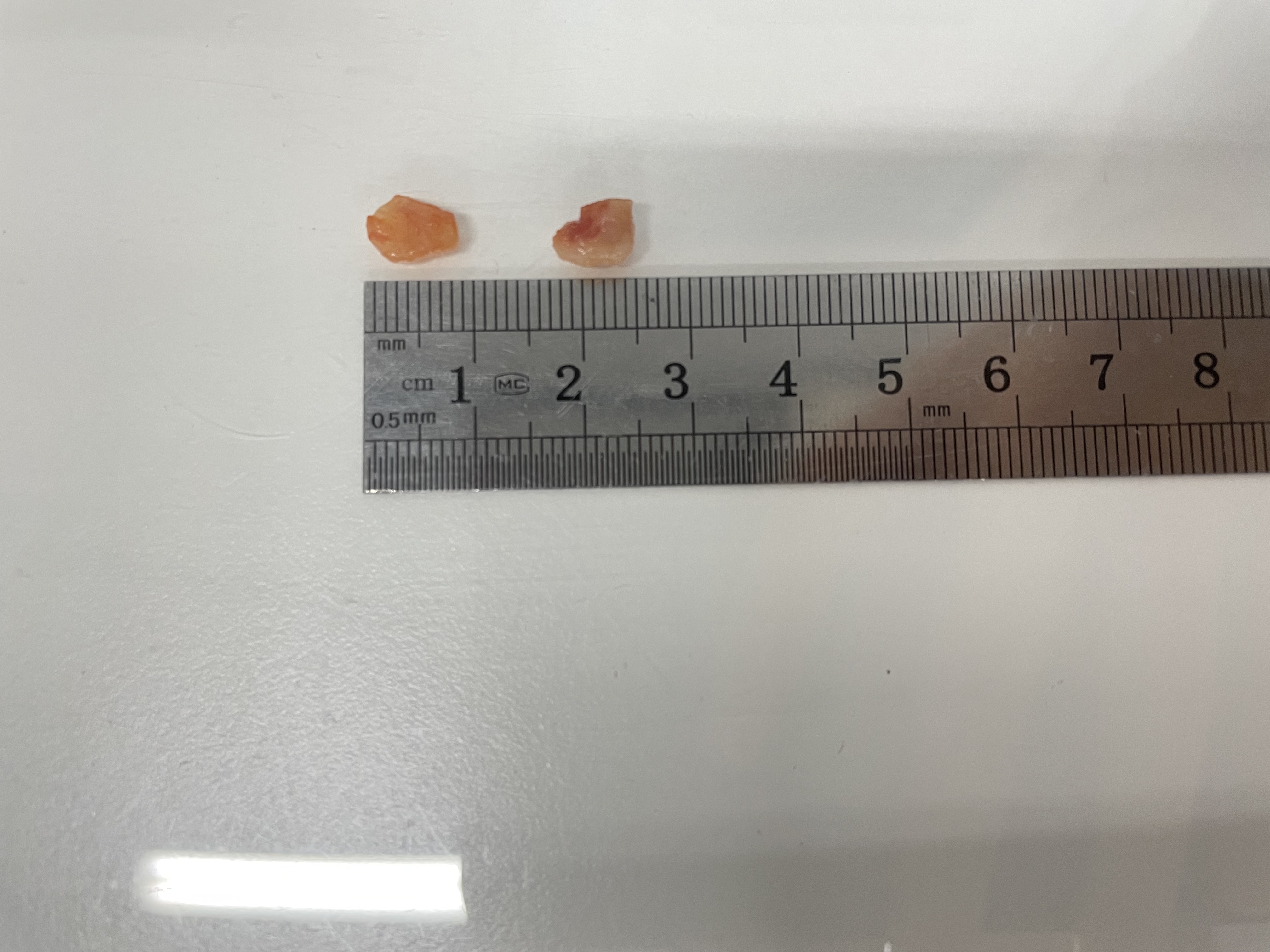

Supplement: Figure 5—figure supplement 2—source data 1. [file elife-70471-fig5-figsupp2-data1.zip › Figure 5-figure supplement 2-Source data 1/oral cancer patient 2/Raw data-photograph image.jpeg]

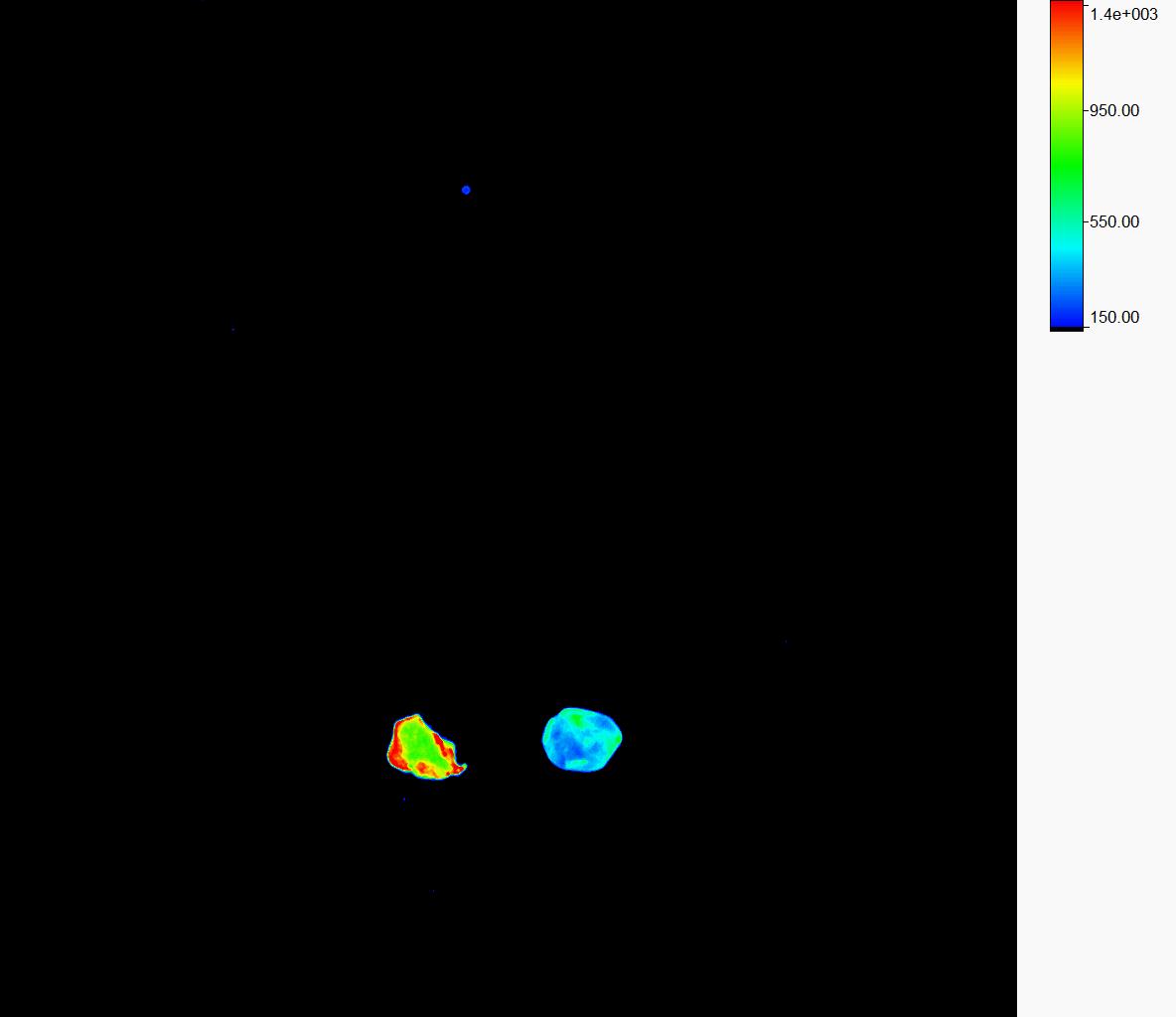

Supplement: Figure 5—figure supplement 2—source data 1. [file elife-70471-fig5-figsupp2-data1.zip › Figure 5-figure supplement 2-Source data 1/oral cancer patient 1/Raw data-viscosity detection image.jpg]

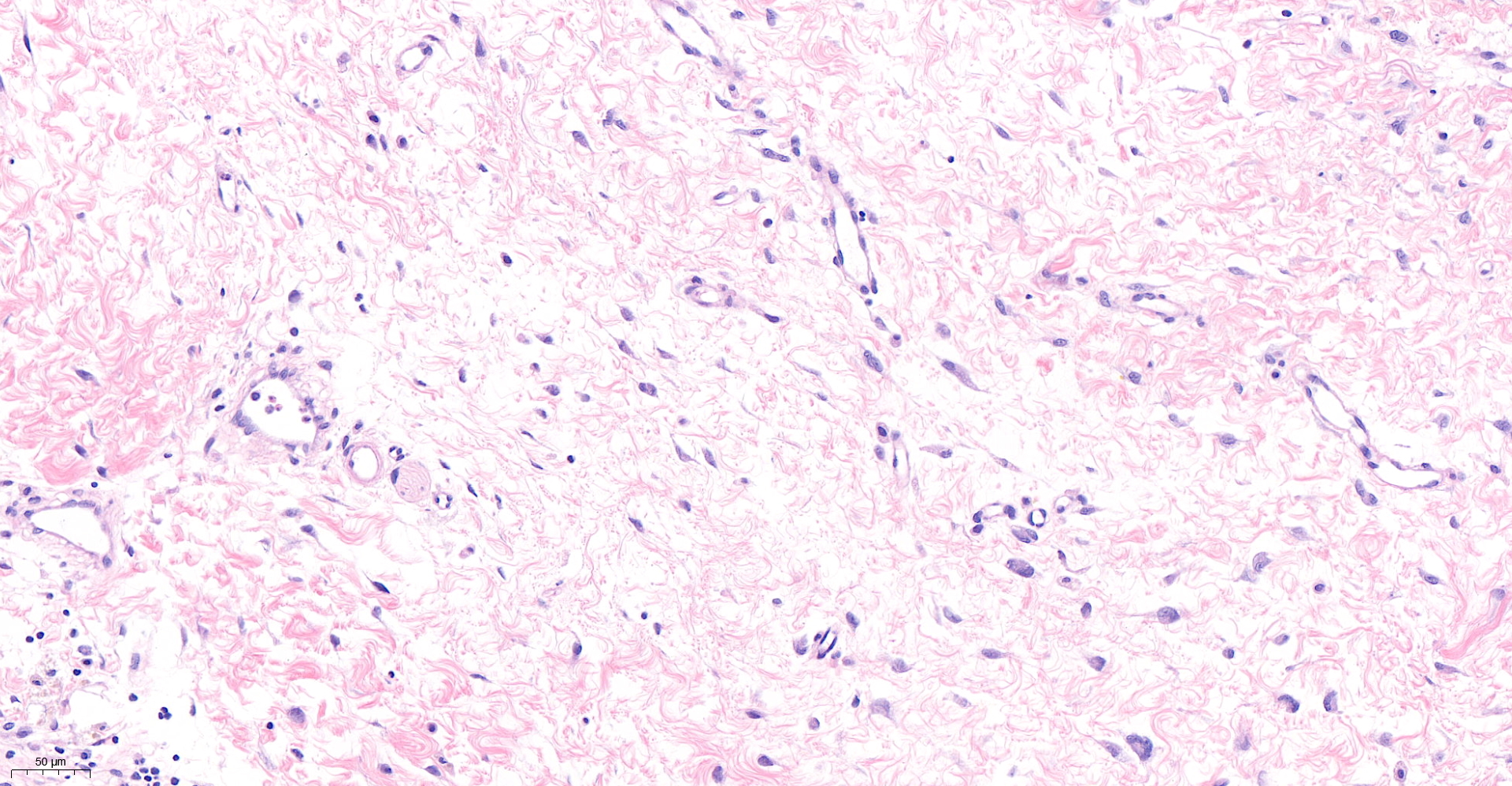

Supplement: Figure 5—figure supplement 2—source data 1. [file elife-70471-fig5-figsupp2-data1.zip › Figure 5-figure supplement 2-Source data 1/oral cancer patient 1/Raw data-HE staining image 1 of patient 1-20.0x.jpg]

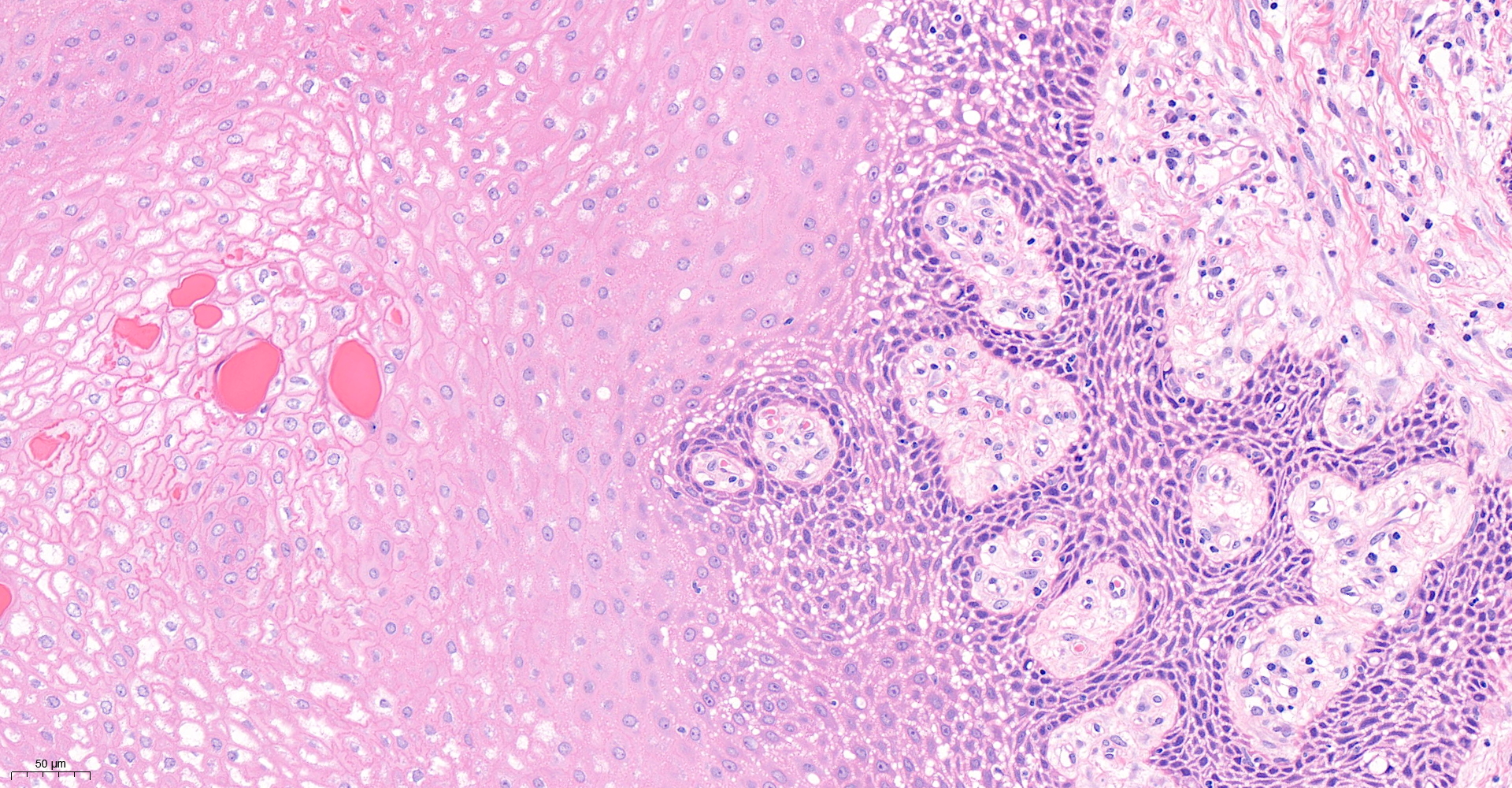

Supplement: Figure 5—figure supplement 2—source data 1. [file elife-70471-fig5-figsupp2-data1.zip › Figure 5-figure supplement 2-Source data 1/oral cancer patient 1/Raw data-HE staining image 2 of patient 1-20.0x.jpg]

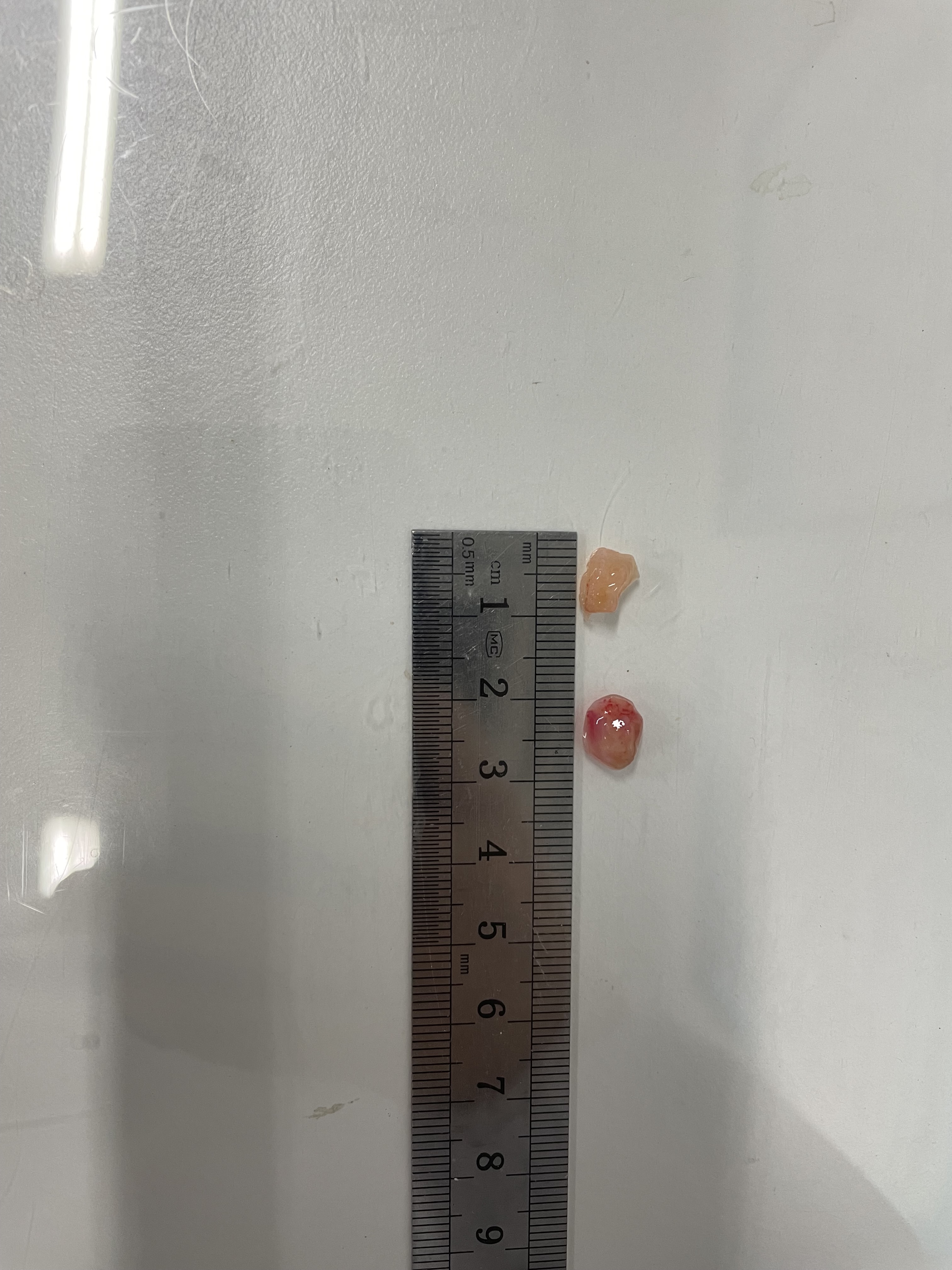

Supplement: Figure 5—figure supplement 2—source data 1. [file elife-70471-fig5-figsupp2-data1.zip › Figure 5-figure supplement 2-Source data 1/oral cancer patient 1/Raw data-photograph image.JPG]

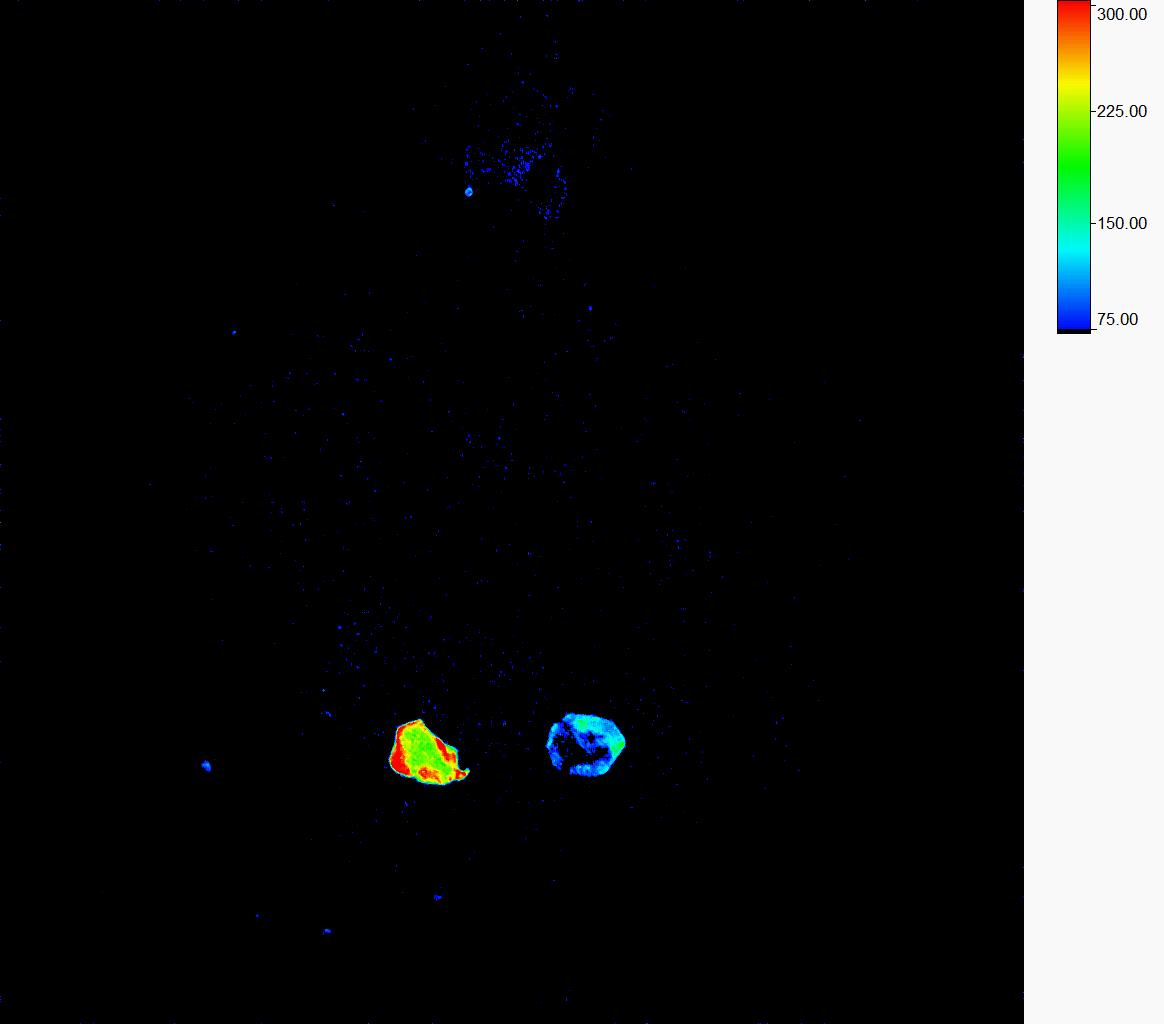

Supplement: Figure 5—figure supplement 2—source data 1. [file elife-70471-fig5-figsupp2-data1.zip › Figure 5-figure supplement 2-Source data 1/oral cancer patient 1/Raw data-nitroreductase detection image.jpg]

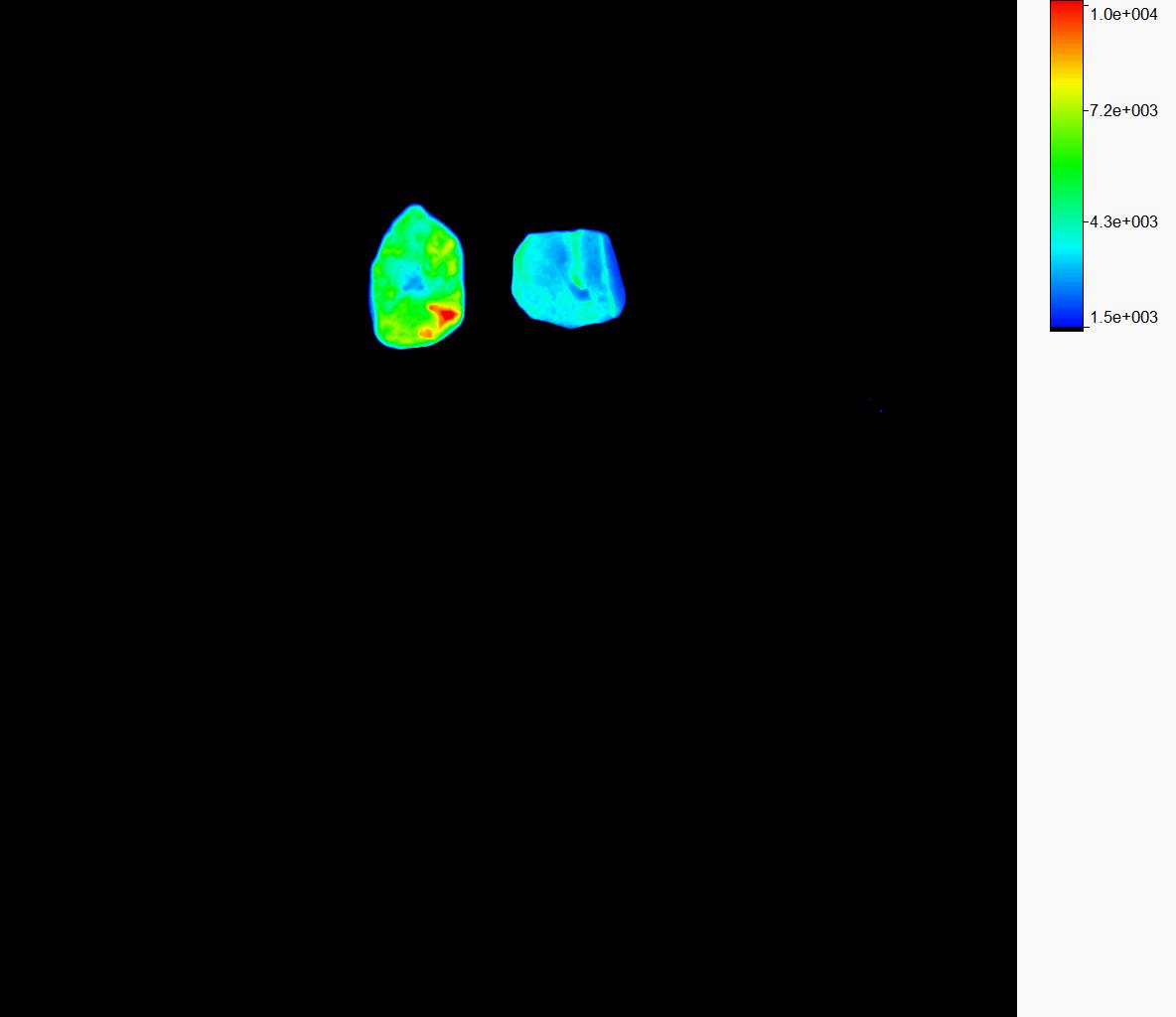

Supplement: Figure 5—figure supplement 3—source data 1. [file elife-70471-fig5-figsupp3-data1.zip › Figure 5-figure supplement 3-Source data 1/renal cancer patient 6/Raw data-viscosity detection image.jpg]

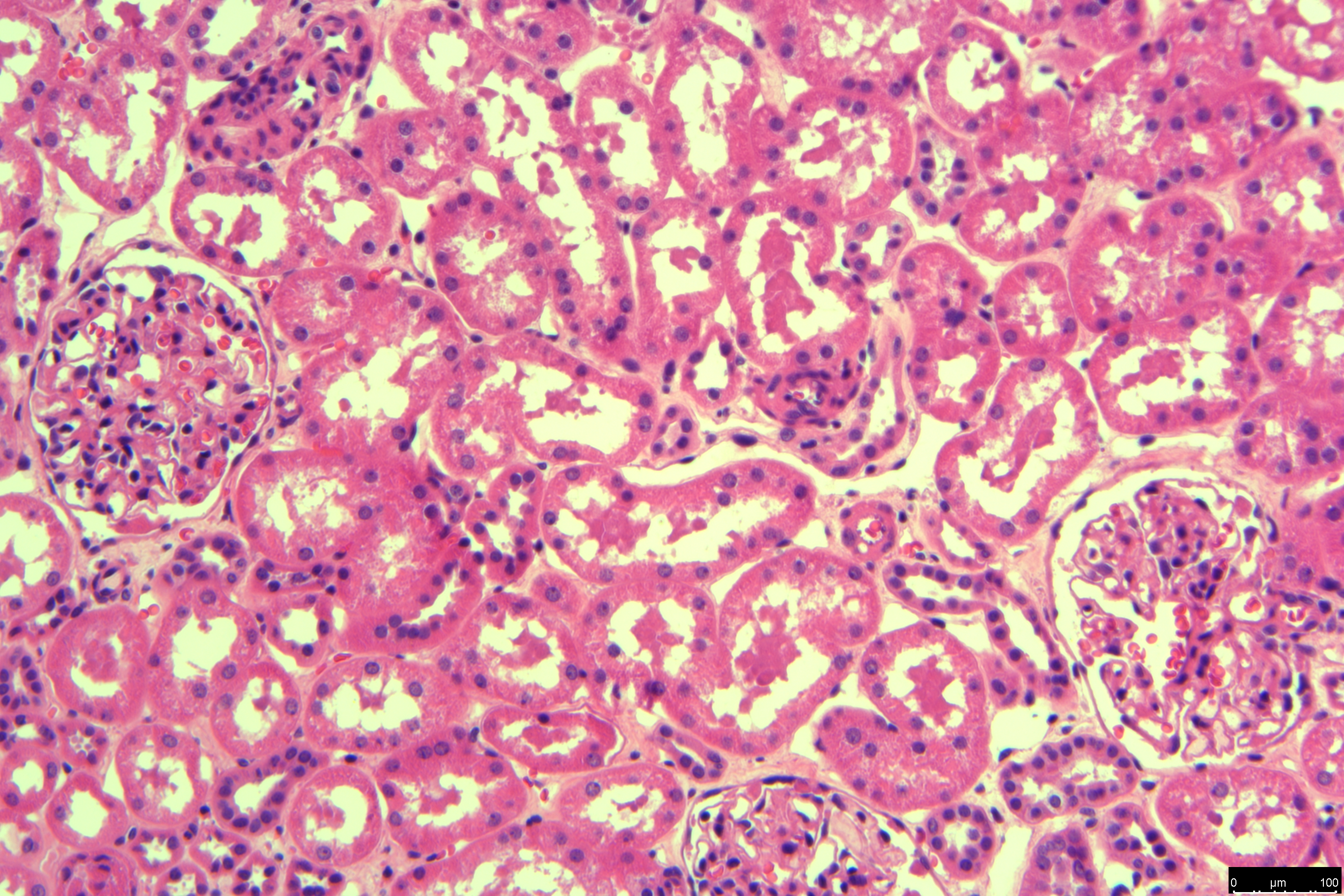

Supplement: Figure 5—figure supplement 3—source data 1. [file elife-70471-fig5-figsupp3-data1.zip › Figure 5-figure supplement 3-Source data 1/renal cancer patient 6/Raw data-HE staining image 1 of patient 6-20.0x.tif]

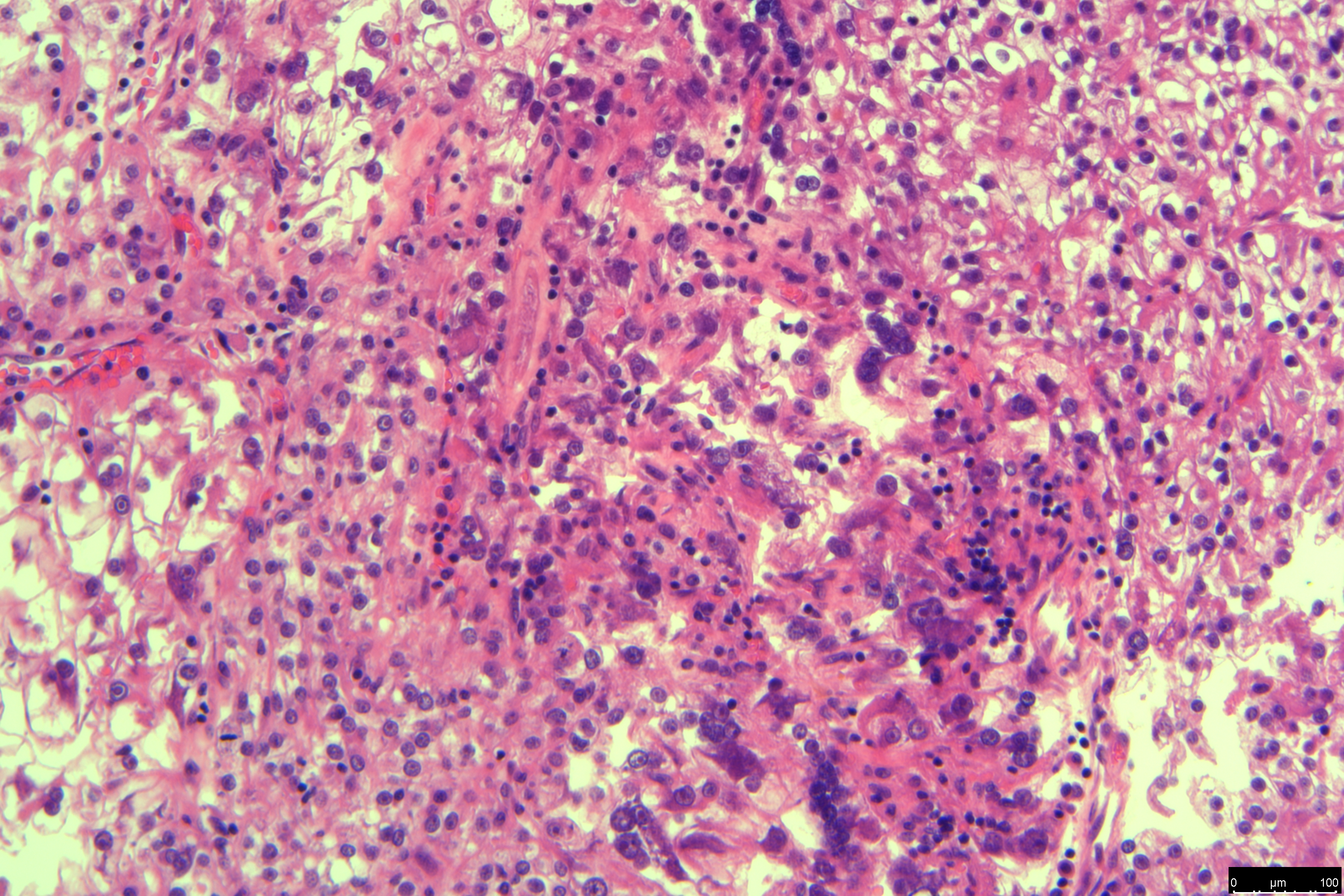

Supplement: Figure 5—figure supplement 3—source data 1. [file elife-70471-fig5-figsupp3-data1.zip › Figure 5-figure supplement 3-Source data 1/renal cancer patient 6/Raw data-HE staining image 2 of patient 6-20.0x.tif]
