# Supplementary figures and images for: A pre-screening strategy to assess resected tumor margins by imaging cytoplasmic viscosity and hypoxia (part 3 of 3)
Source: eLife. 2021 Oct 11;10:e70471. doi: 10.7554/eLife.70471 (PMC8553343; doi:10.7554/eLife.70471)

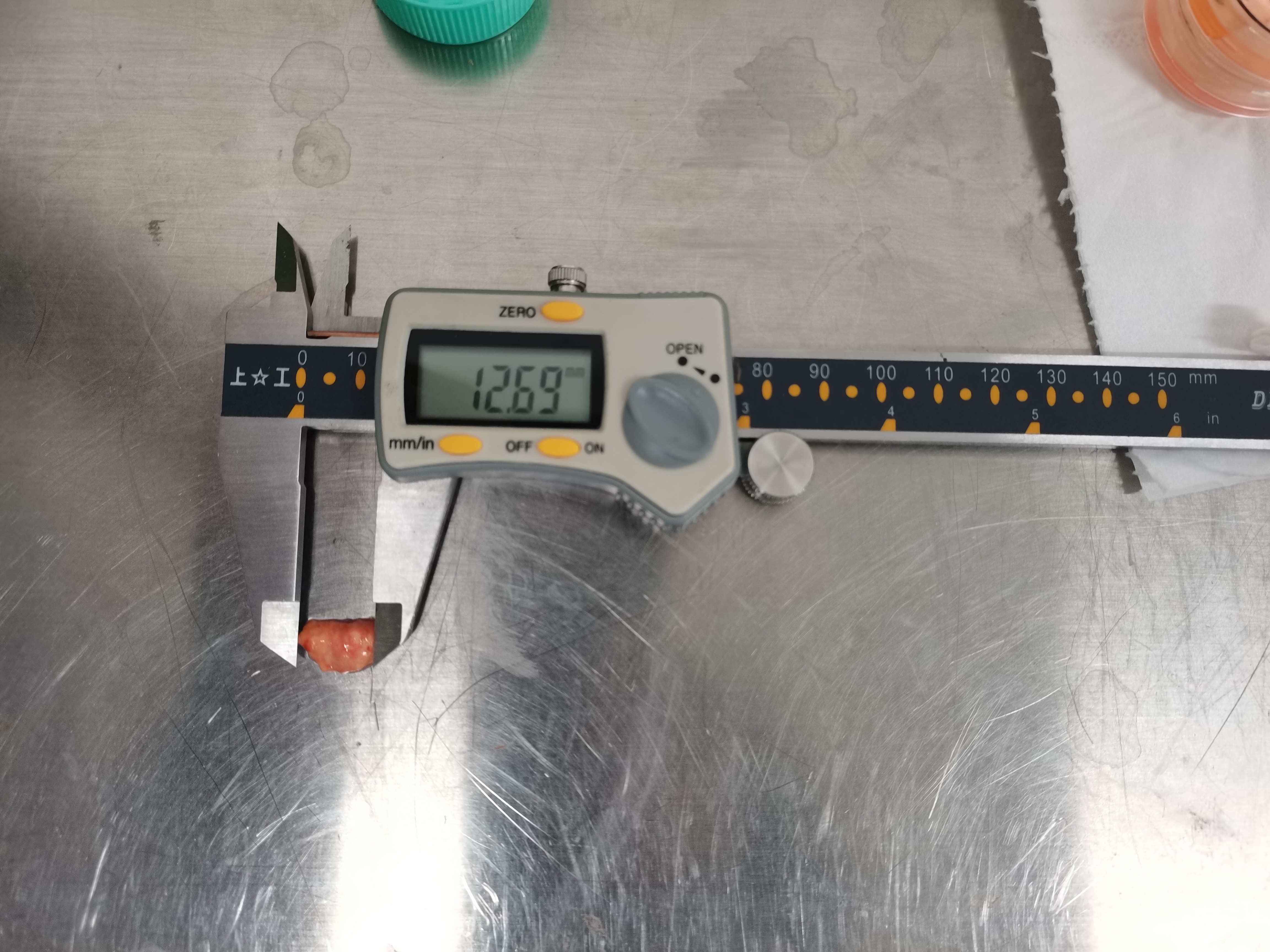

Supplement: Figure 5—figure supplement 3—source data 1. [file elife-70471-fig5-figsupp3-data1.zip › Figure 5-figure supplement 3-Source data 1/renal cancer patient 6/Raw data-photograph image 2.jpg]

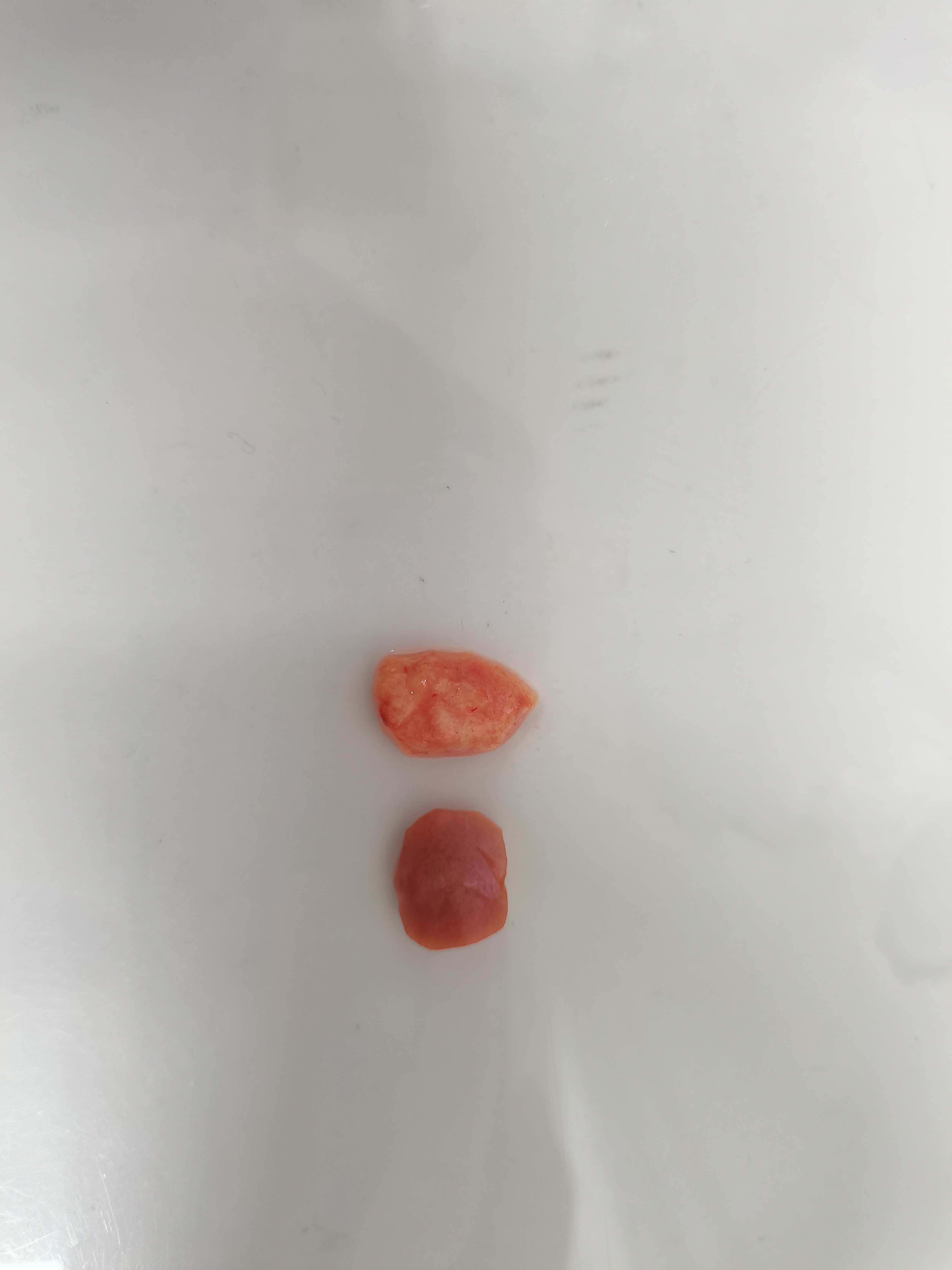

Supplement: Figure 5—figure supplement 3—source data 1. [file elife-70471-fig5-figsupp3-data1.zip › Figure 5-figure supplement 3-Source data 1/renal cancer patient 6/Raw data-photograph image 1.jpg]

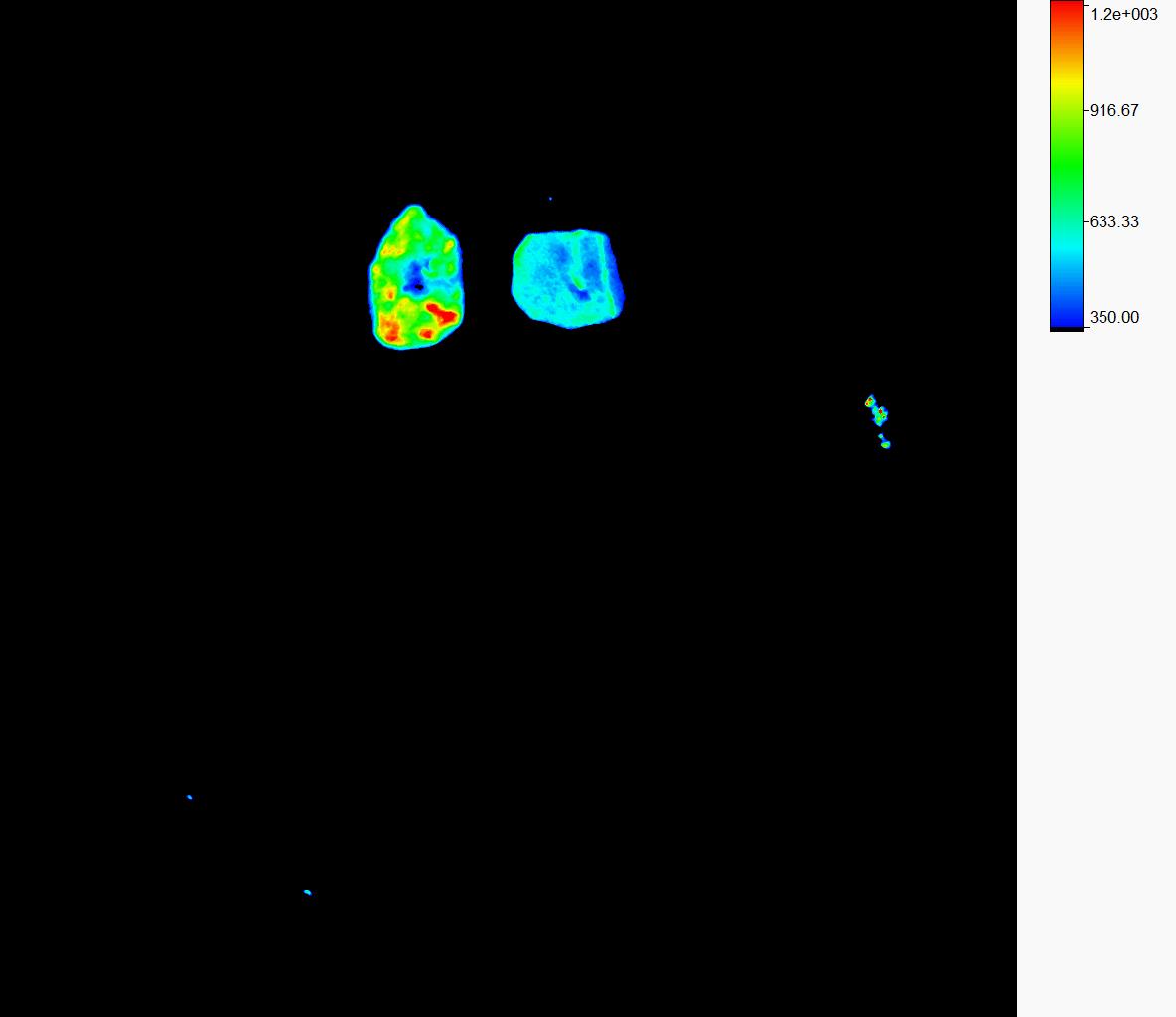

Supplement: Figure 5—figure supplement 3—source data 1. [file elife-70471-fig5-figsupp3-data1.zip › Figure 5-figure supplement 3-Source data 1/renal cancer patient 6/Raw data-nitroreductase detection image.jpg]

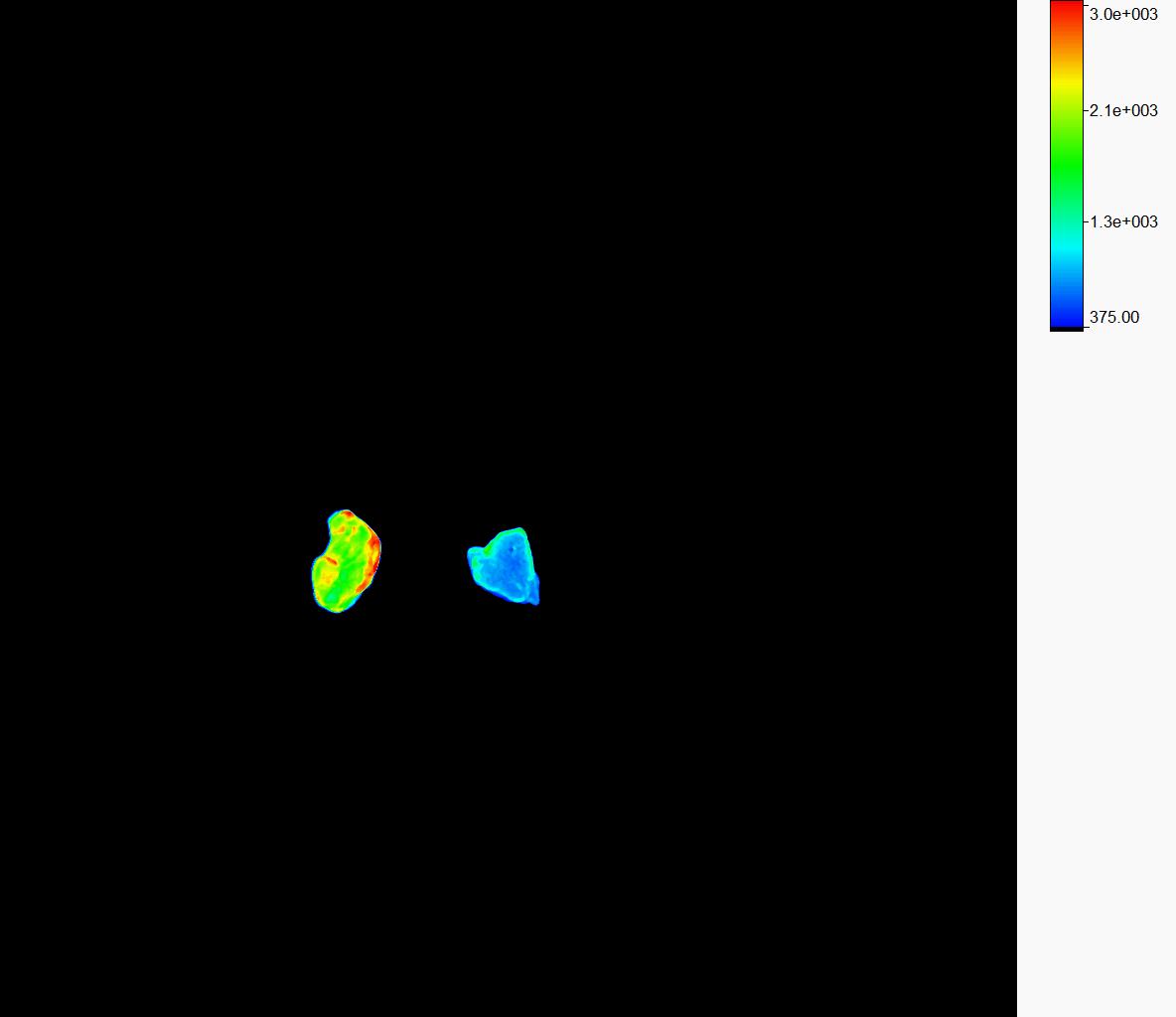

Supplement: Figure 5—figure supplement 3—source data 1. [file elife-70471-fig5-figsupp3-data1.zip › Figure 5-figure supplement 3-Source data 1/renal cancer patient 1/Raw data-viscosity detection image.jpg]

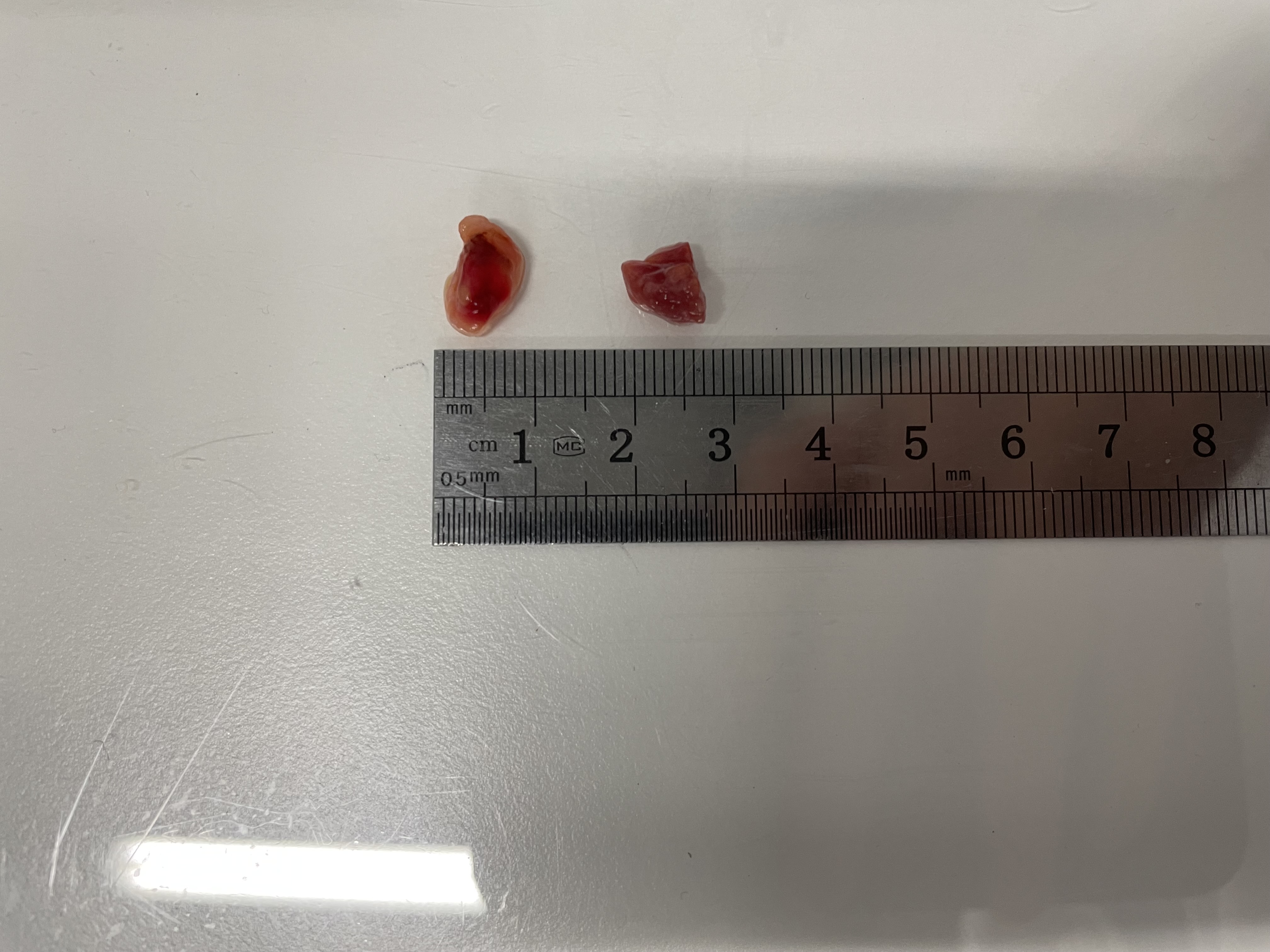

Supplement: Figure 5—figure supplement 3—source data 1. [file elife-70471-fig5-figsupp3-data1.zip › Figure 5-figure supplement 3-Source data 1/renal cancer patient 1/Raw data-photograph image.png]

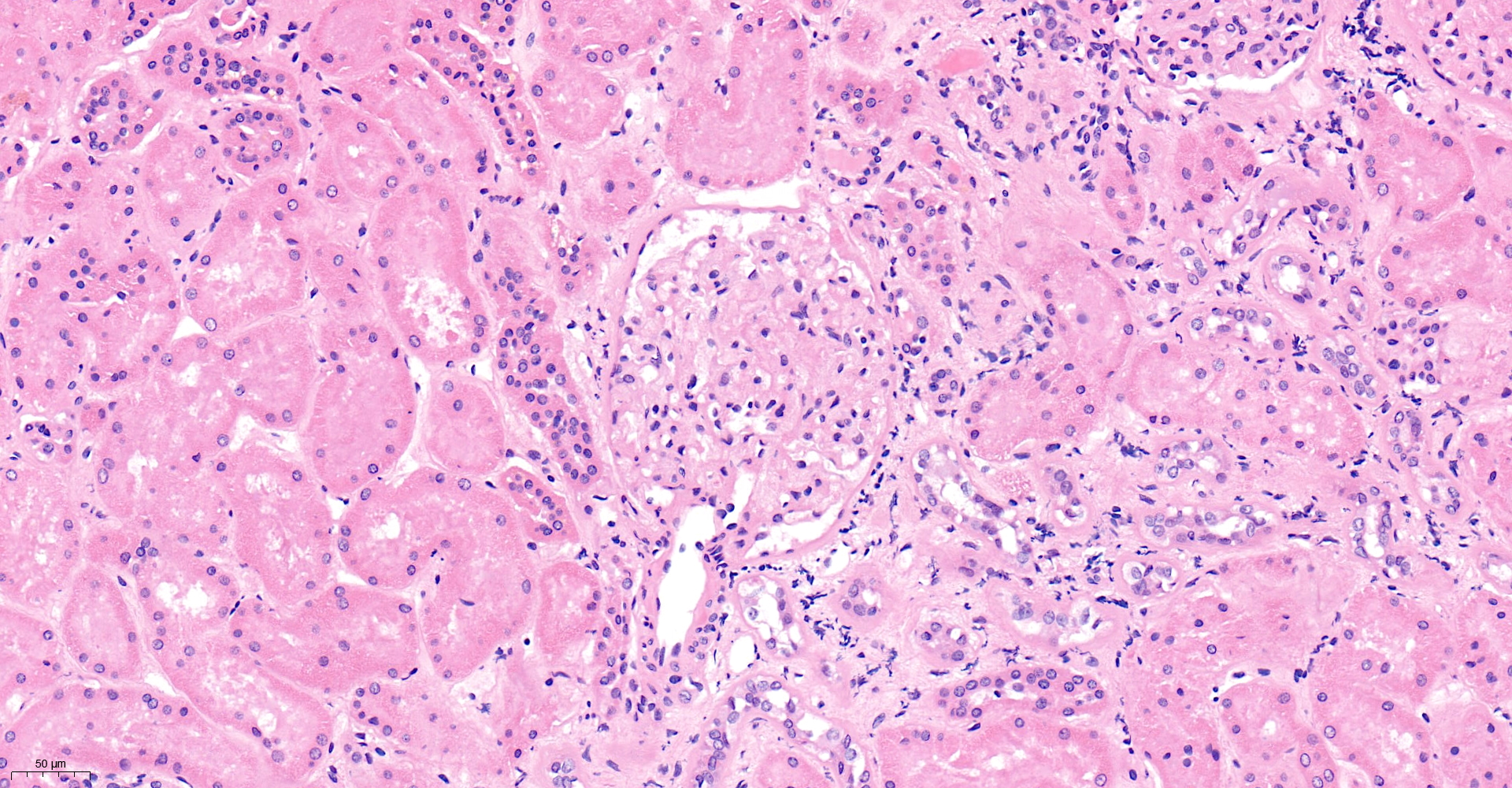

Supplement: Figure 5—figure supplement 3—source data 1. [file elife-70471-fig5-figsupp3-data1.zip › Figure 5-figure supplement 3-Source data 1/renal cancer patient 1/Raw data-HE staining image 1 of patient 2-20.0x.jpg]

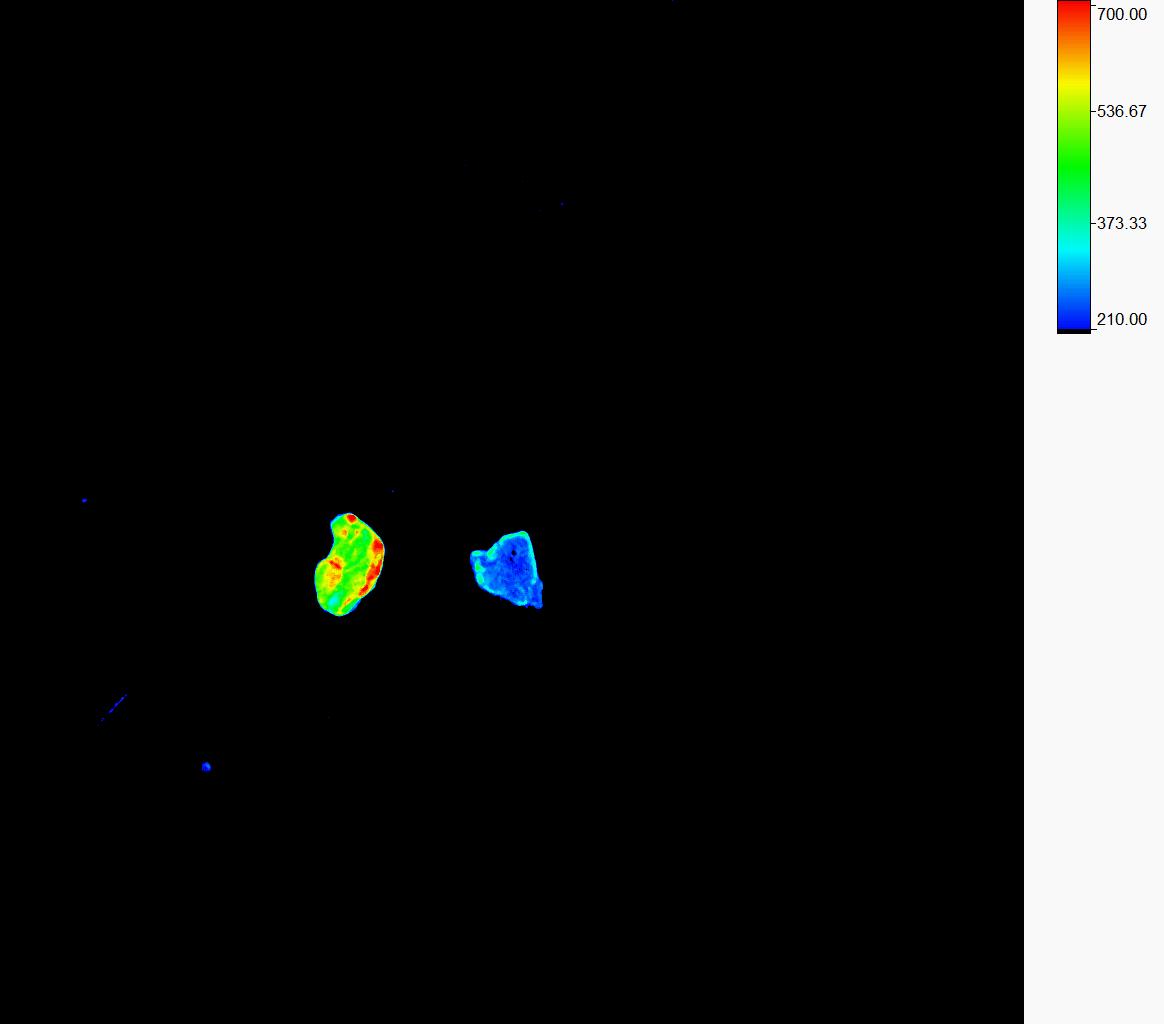

Supplement: Figure 5—figure supplement 3—source data 1. [file elife-70471-fig5-figsupp3-data1.zip › Figure 5-figure supplement 3-Source data 1/renal cancer patient 1/Raw data-nitroreductase detection image.jpg]

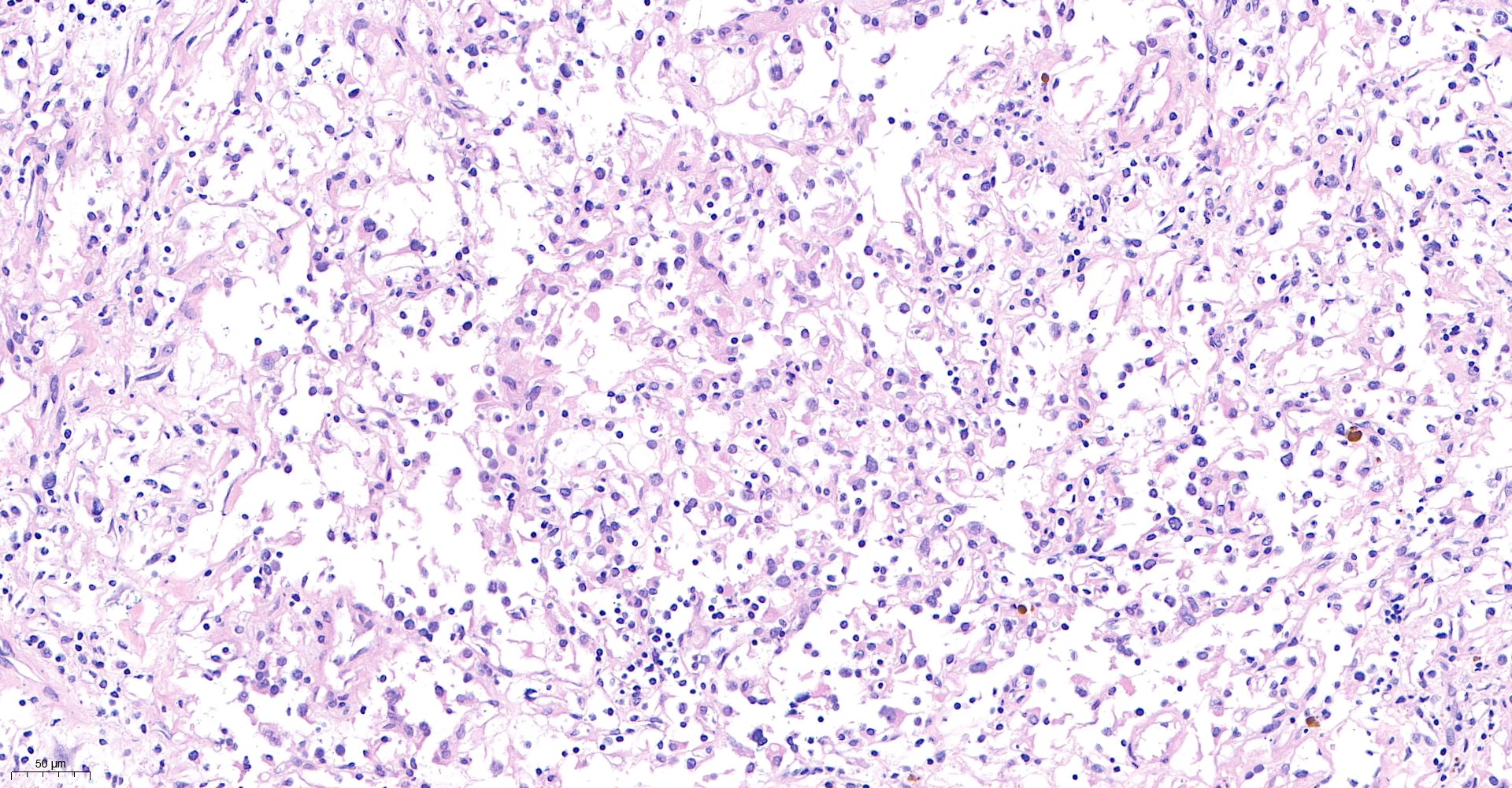

Supplement: Figure 5—figure supplement 3—source data 1. [file elife-70471-fig5-figsupp3-data1.zip › Figure 5-figure supplement 3-Source data 1/renal cancer patient 1/Raw data-HE staining image 2 of patient 2-20.0x.jpg]

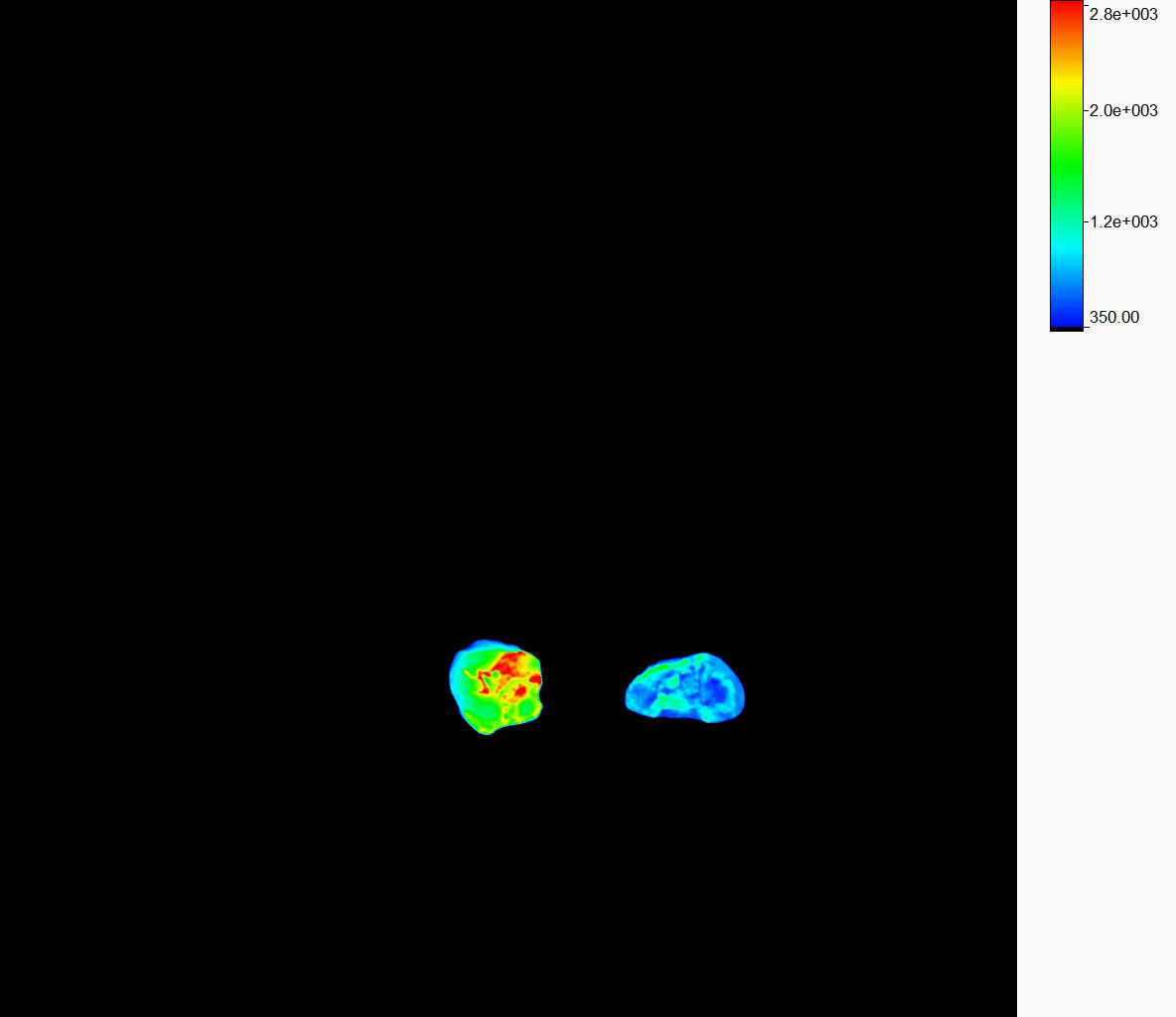

Supplement: Figure 5—figure supplement 3—source data 1. [file elife-70471-fig5-figsupp3-data1.zip › Figure 5-figure supplement 3-Source data 1/renal cancer patient 4/Raw data-viscosity detection image.jpg]

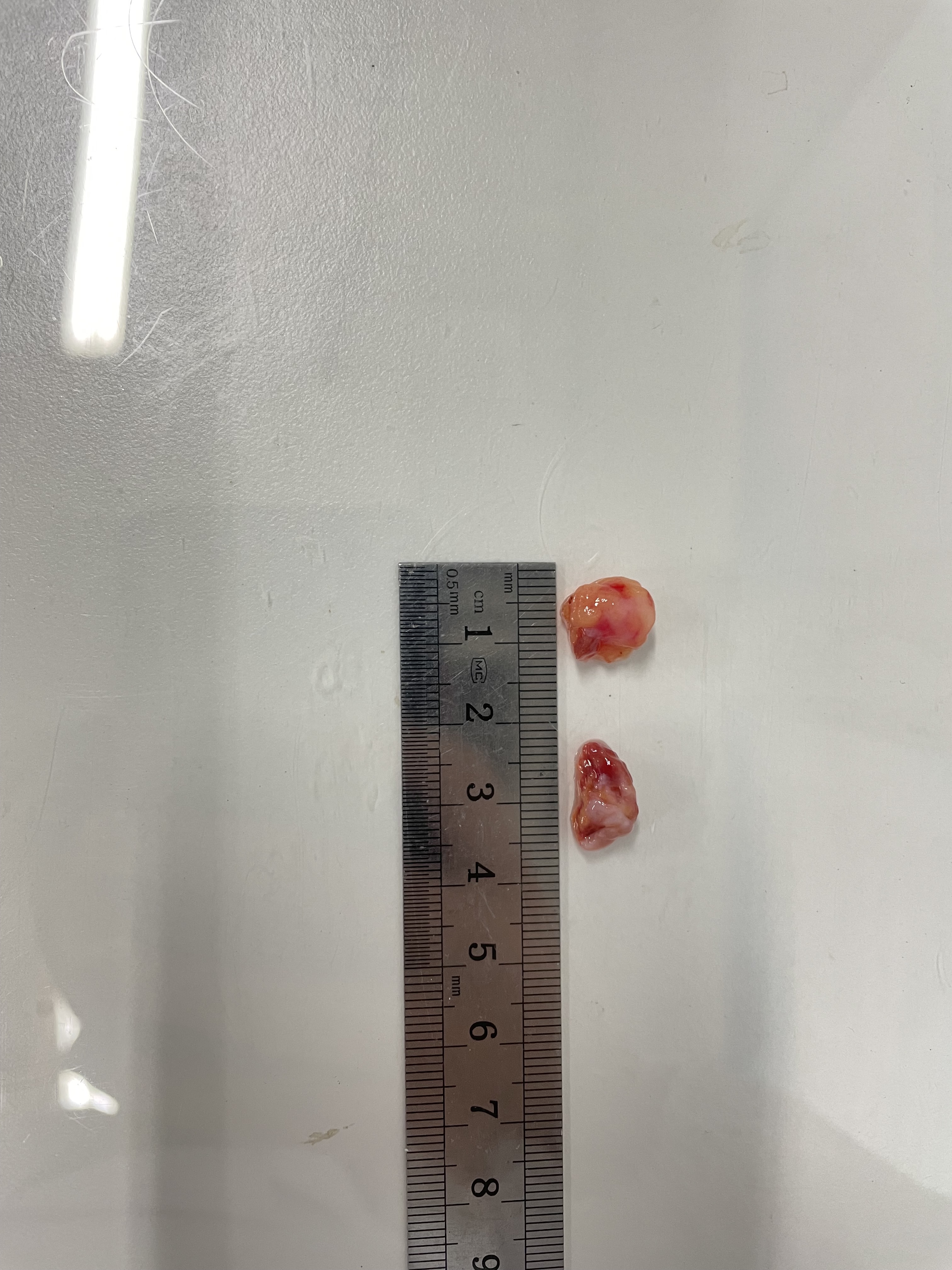

Supplement: Figure 5—figure supplement 3—source data 1. [file elife-70471-fig5-figsupp3-data1.zip › Figure 5-figure supplement 3-Source data 1/renal cancer patient 4/Raw data-photograph image.JPG]

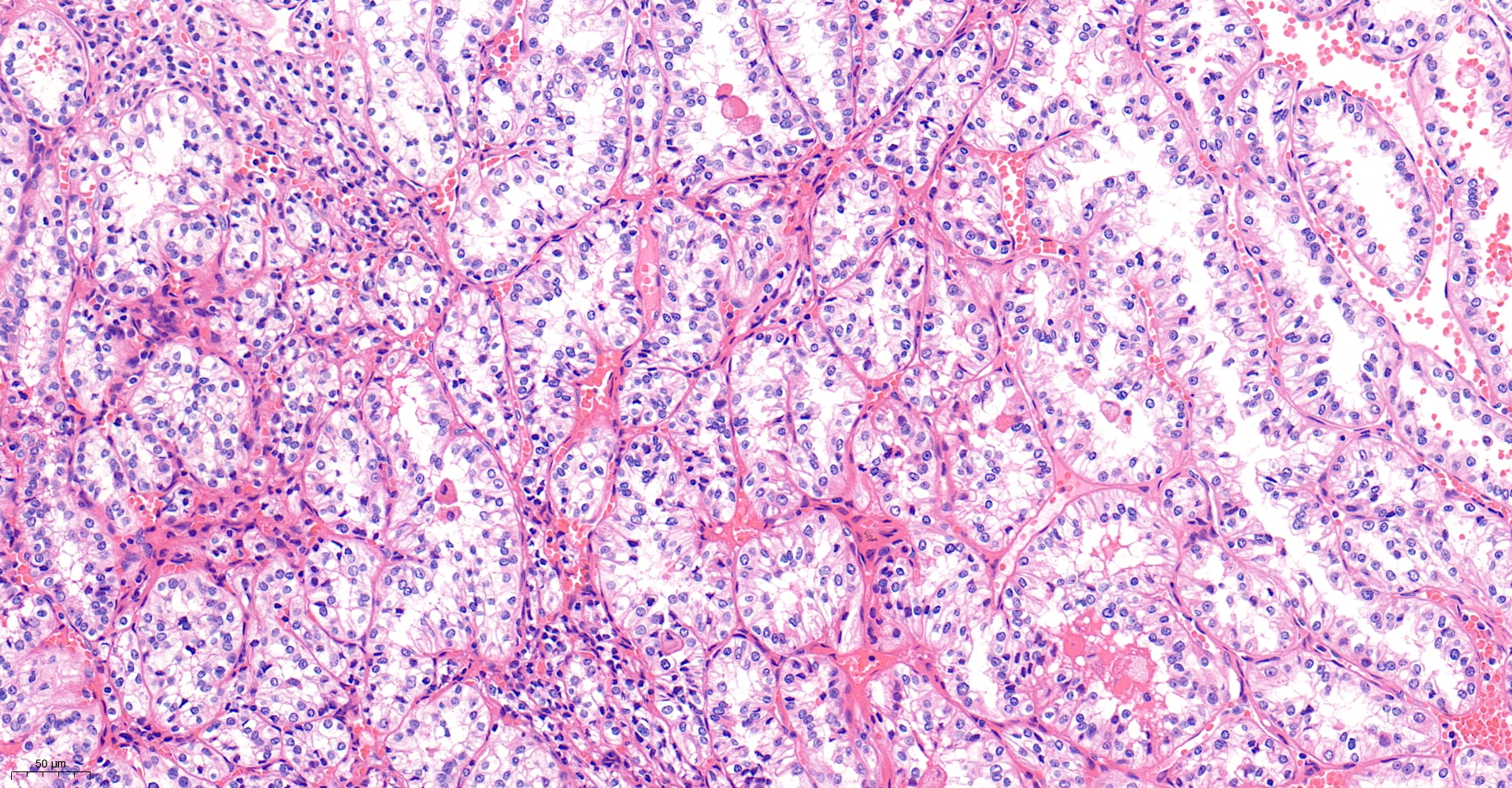

Supplement: Figure 5—figure supplement 3—source data 1. [file elife-70471-fig5-figsupp3-data1.zip › Figure 5-figure supplement 3-Source data 1/renal cancer patient 4/Raw data-HE staining image 2 of patient 4-20.0x.jpg]

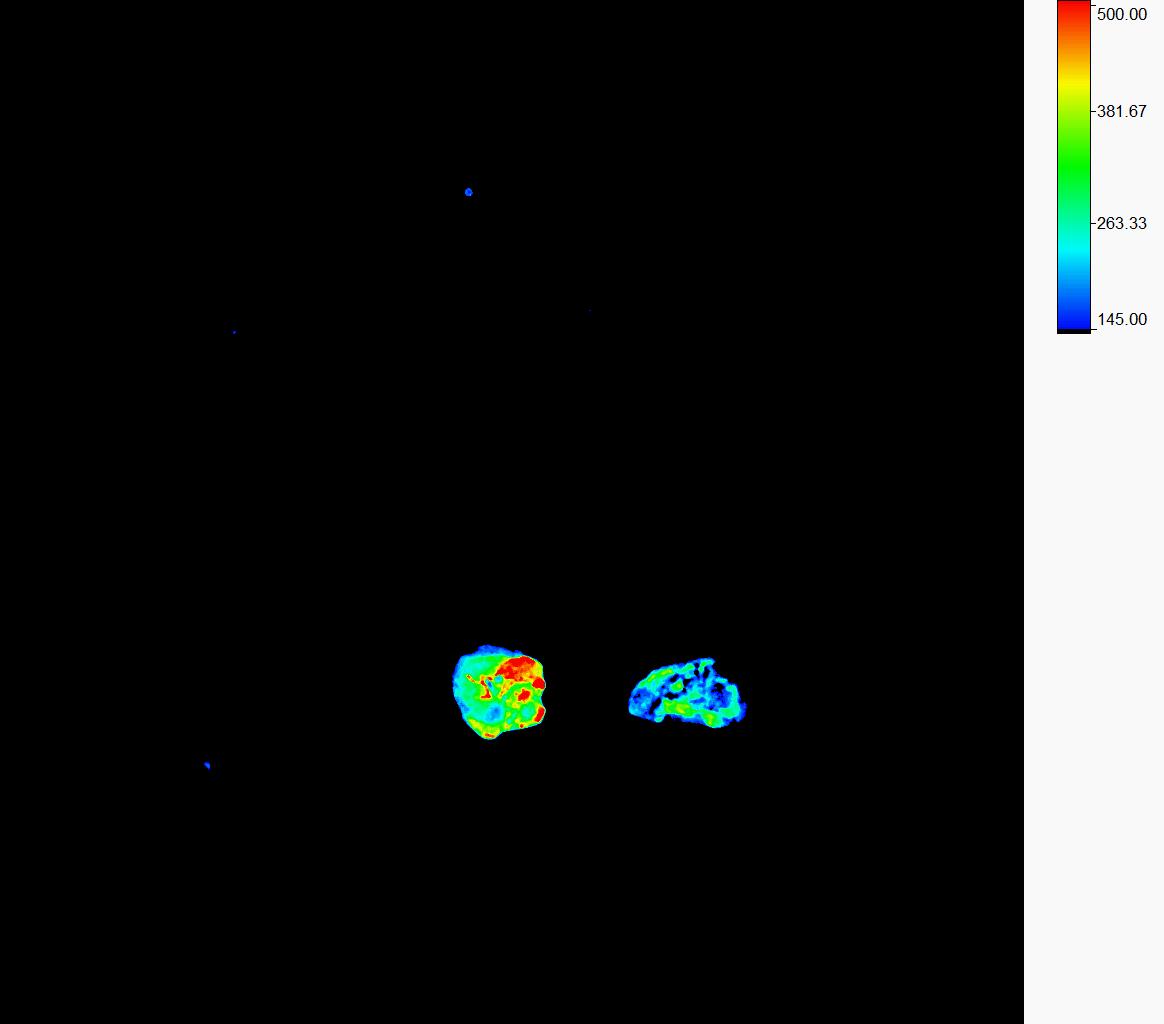

Supplement: Figure 5—figure supplement 3—source data 1. [file elife-70471-fig5-figsupp3-data1.zip › Figure 5-figure supplement 3-Source data 1/renal cancer patient 4/Raw data-nitroreductase detection image.jpg]

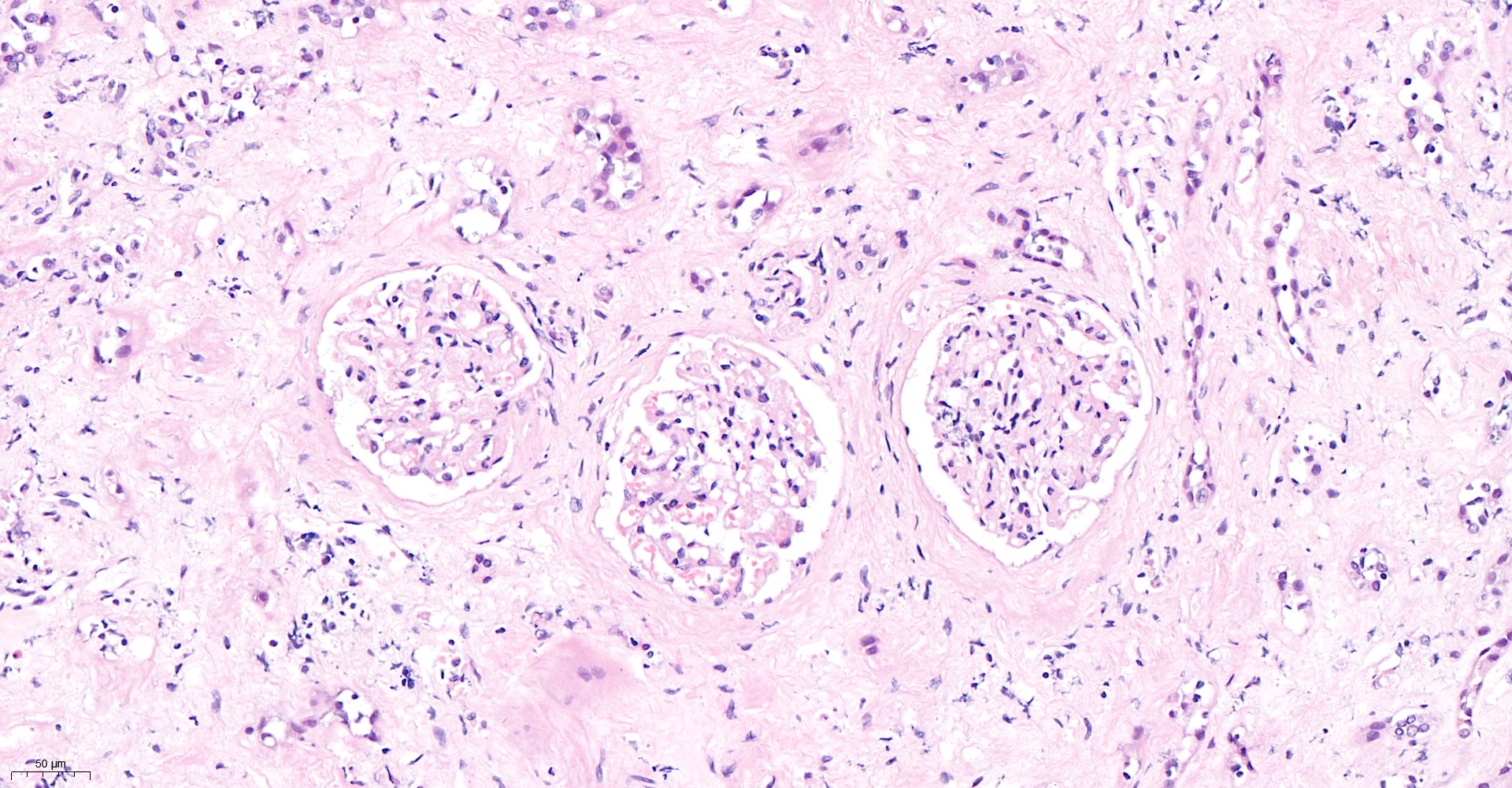

Supplement: Figure 5—figure supplement 3—source data 1. [file elife-70471-fig5-figsupp3-data1.zip › Figure 5-figure supplement 3-Source data 1/renal cancer patient 4/Raw data-HE staining image 1 of patient 4-20.0x.jpg]

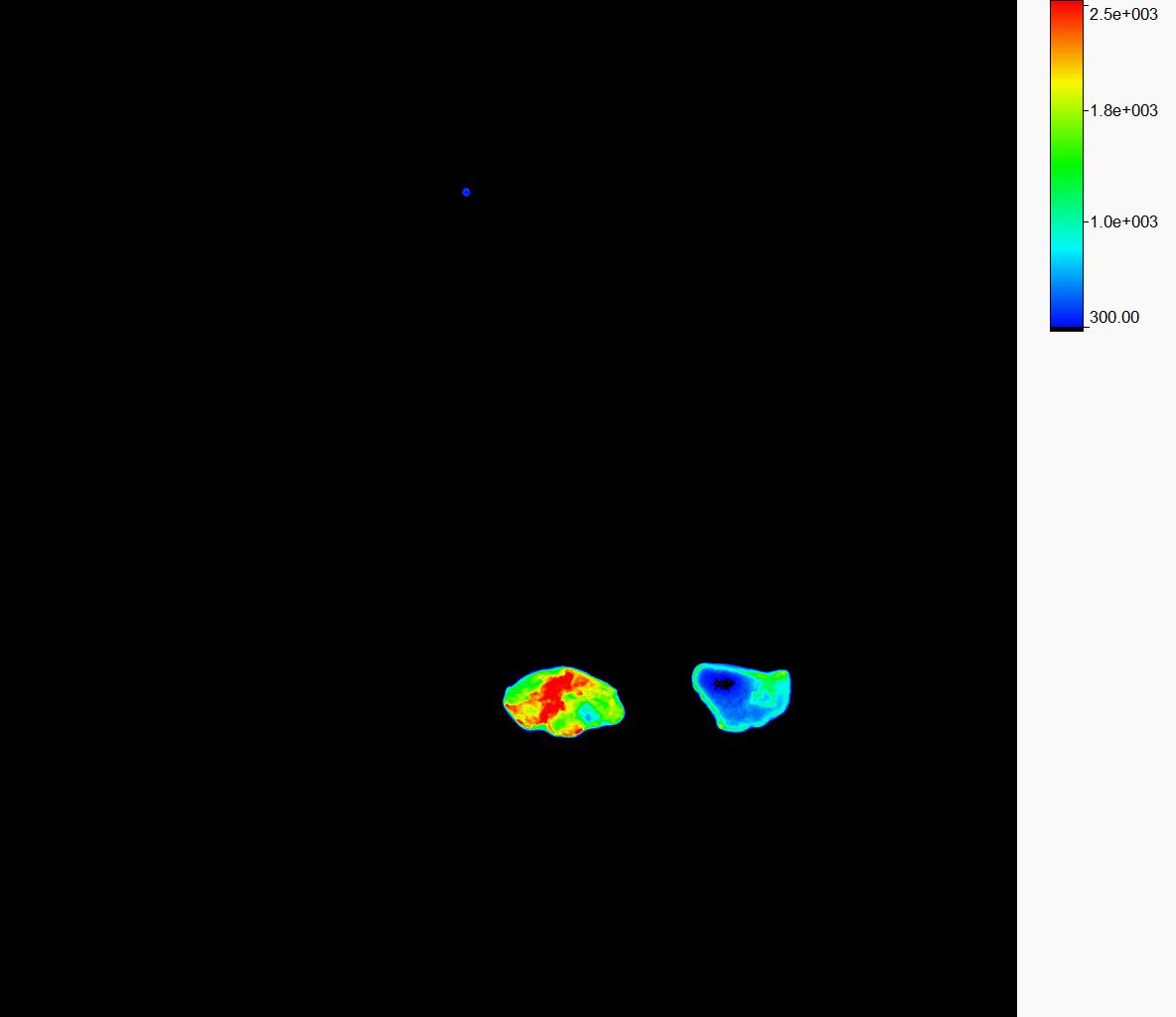

Supplement: Figure 5—figure supplement 3—source data 1. [file elife-70471-fig5-figsupp3-data1.zip › Figure 5-figure supplement 3-Source data 1/renal cancer patient 3/Raw data-viscosity detection image.jpg]

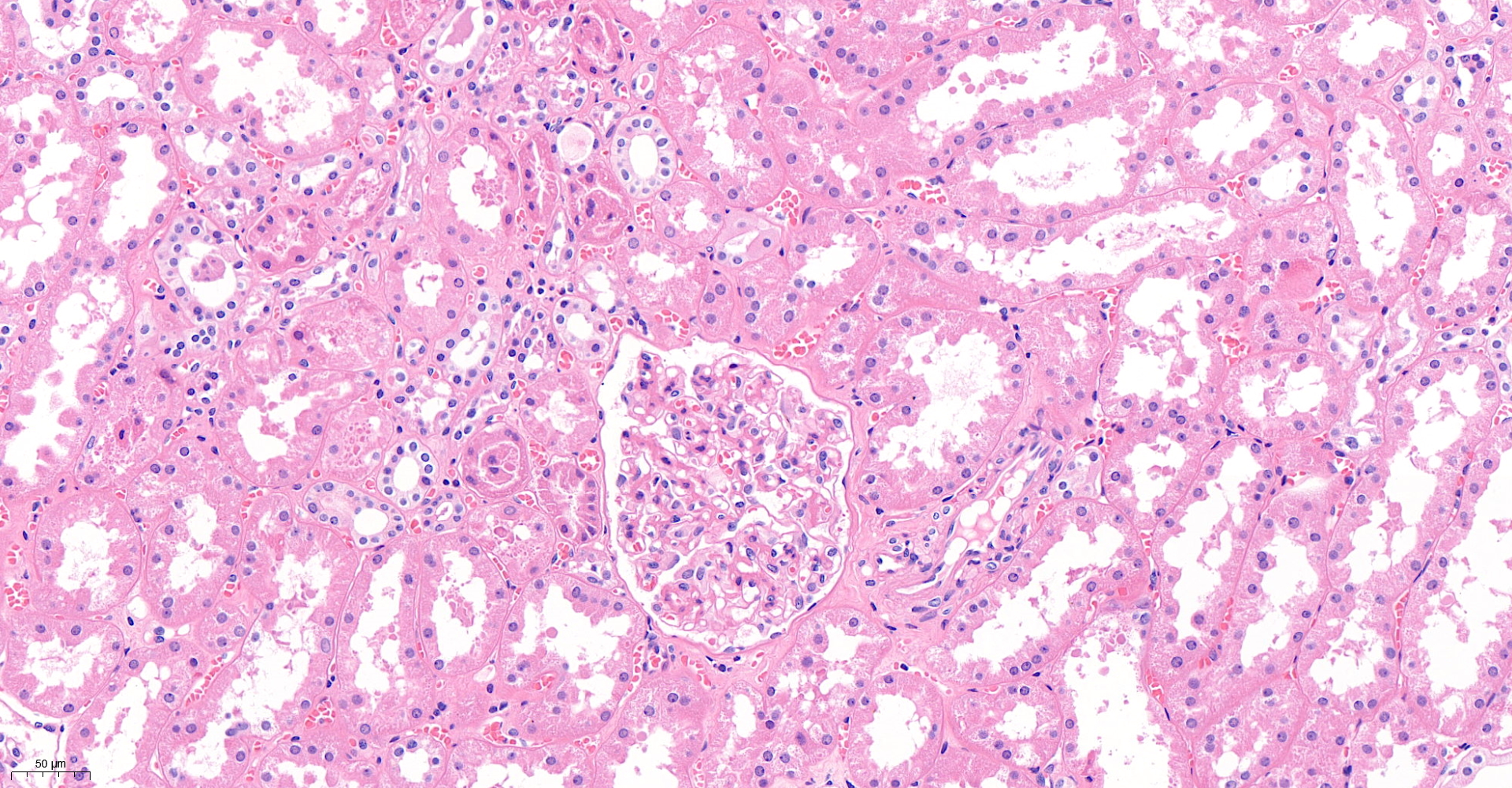

Supplement: Figure 5—figure supplement 3—source data 1. [file elife-70471-fig5-figsupp3-data1.zip › Figure 5-figure supplement 3-Source data 1/renal cancer patient 3/Raw data-HE staining image 1 of patient 3-20.0x.jpg]

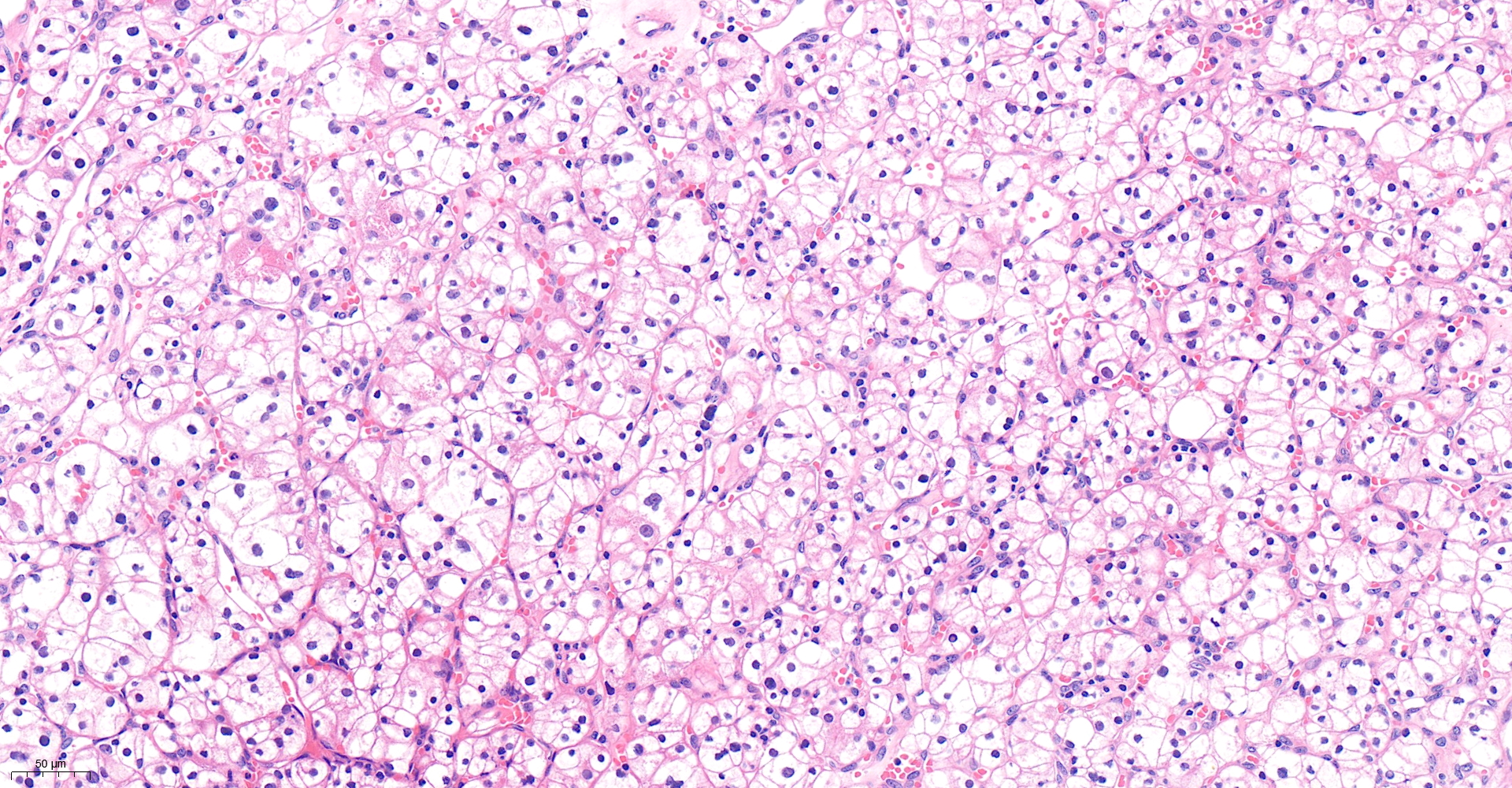

Supplement: Figure 5—figure supplement 3—source data 1. [file elife-70471-fig5-figsupp3-data1.zip › Figure 5-figure supplement 3-Source data 1/renal cancer patient 3/Raw data-HE staining image 2 of patient 3-20.0x.jpg]

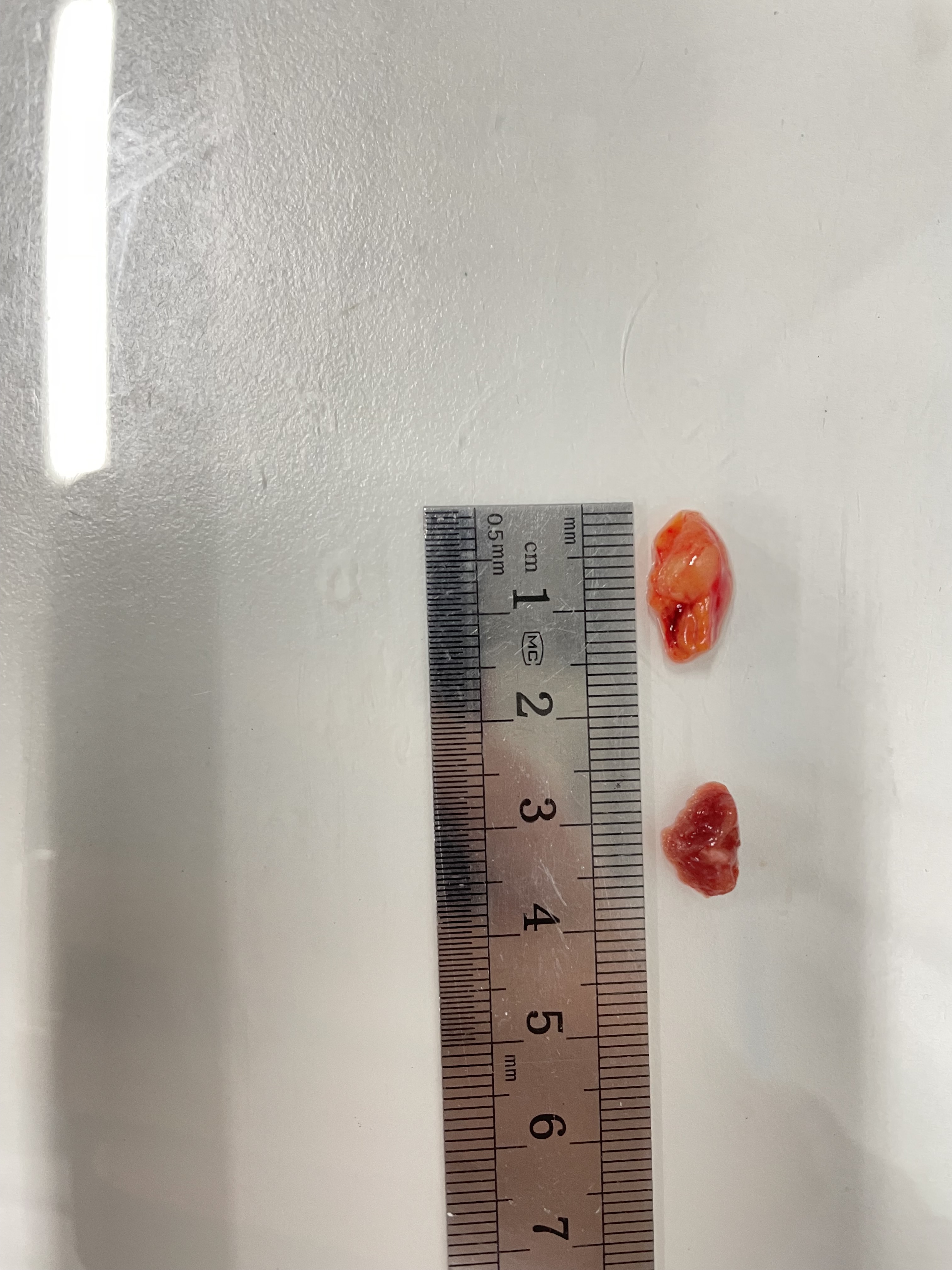

Supplement: Figure 5—figure supplement 3—source data 1. [file elife-70471-fig5-figsupp3-data1.zip › Figure 5-figure supplement 3-Source data 1/renal cancer patient 3/Raw data-photograph image.JPG]

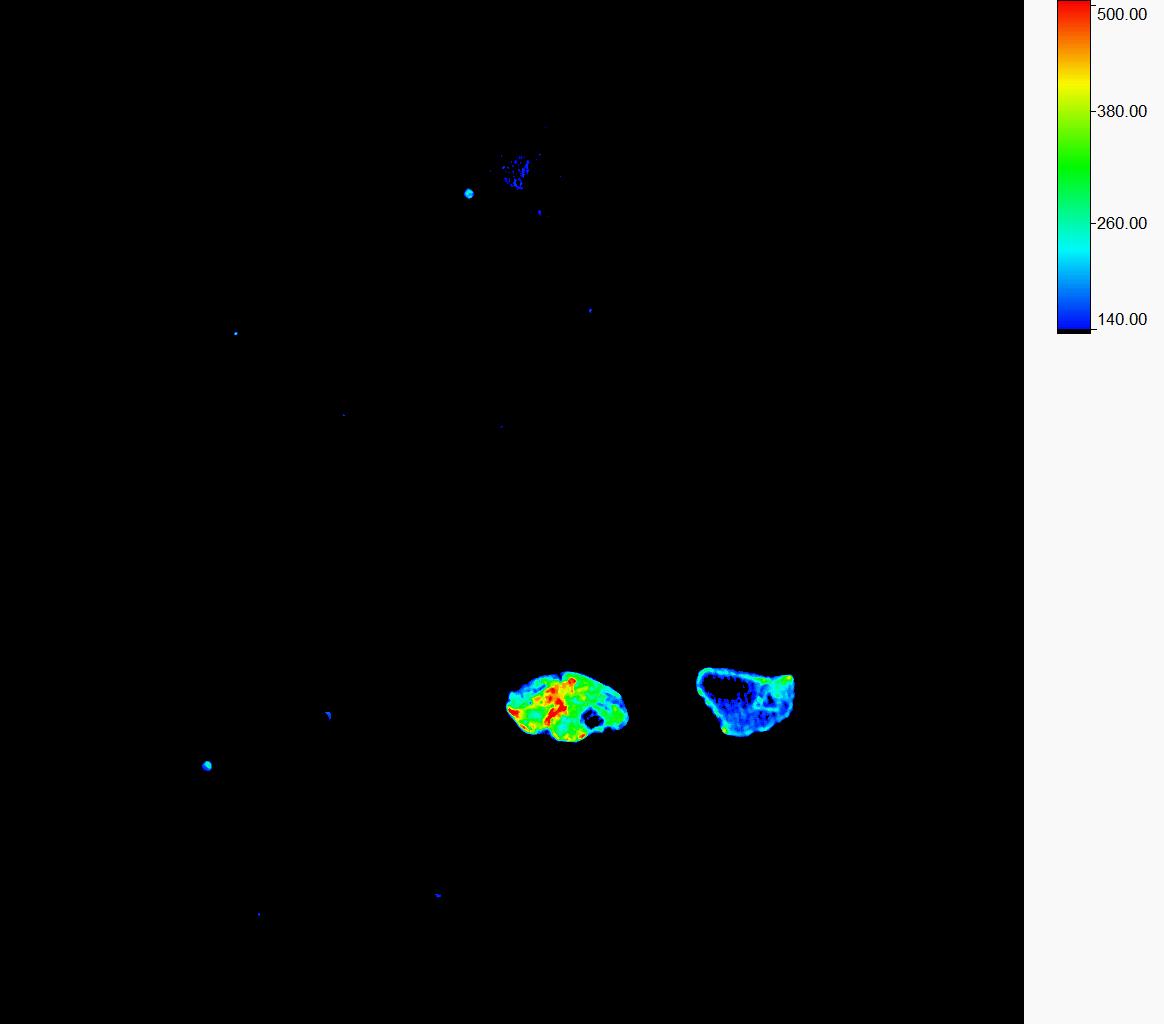

Supplement: Figure 5—figure supplement 3—source data 1. [file elife-70471-fig5-figsupp3-data1.zip › Figure 5-figure supplement 3-Source data 1/renal cancer patient 3/Raw data-nitroreductase detection image.jpg]

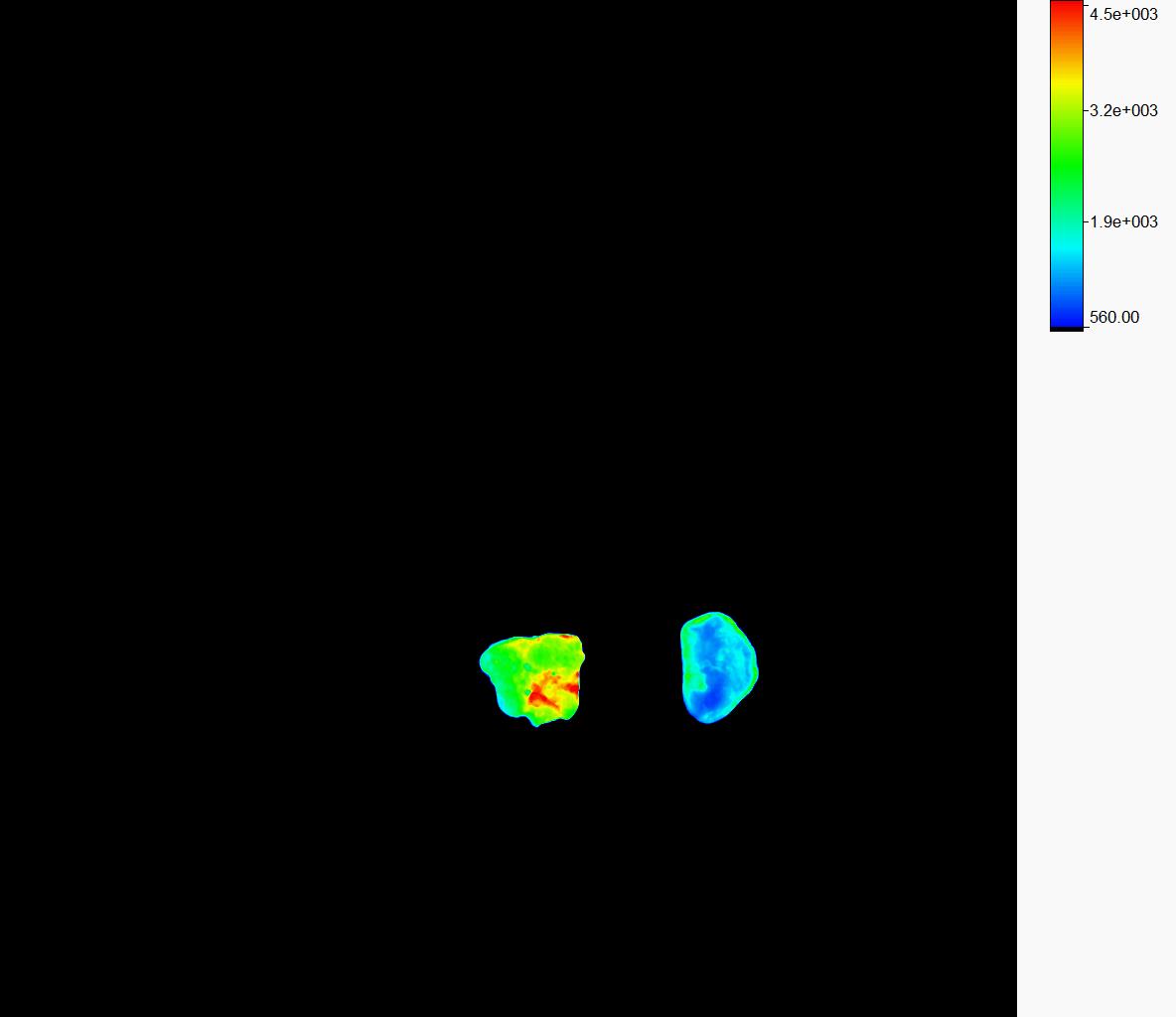

Supplement: Figure 5—figure supplement 3—source data 1. [file elife-70471-fig5-figsupp3-data1.zip › Figure 5-figure supplement 3-Source data 1/renal cancer patient 2/Raw data-viscosity detection image.jpg]

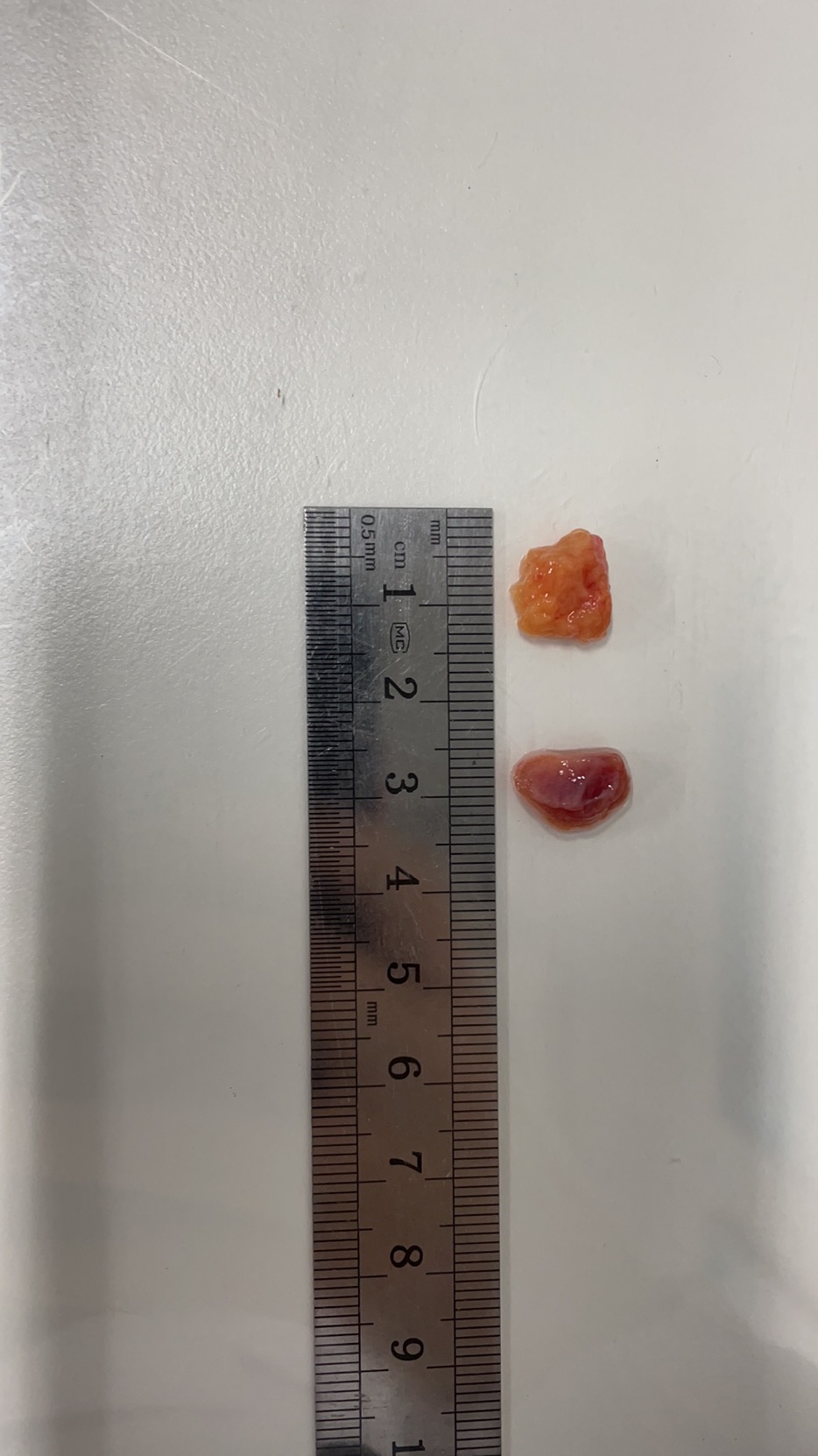

Supplement: Figure 5—figure supplement 3—source data 1. [file elife-70471-fig5-figsupp3-data1.zip › Figure 5-figure supplement 3-Source data 1/renal cancer patient 2/Raw data-photograph image.png]

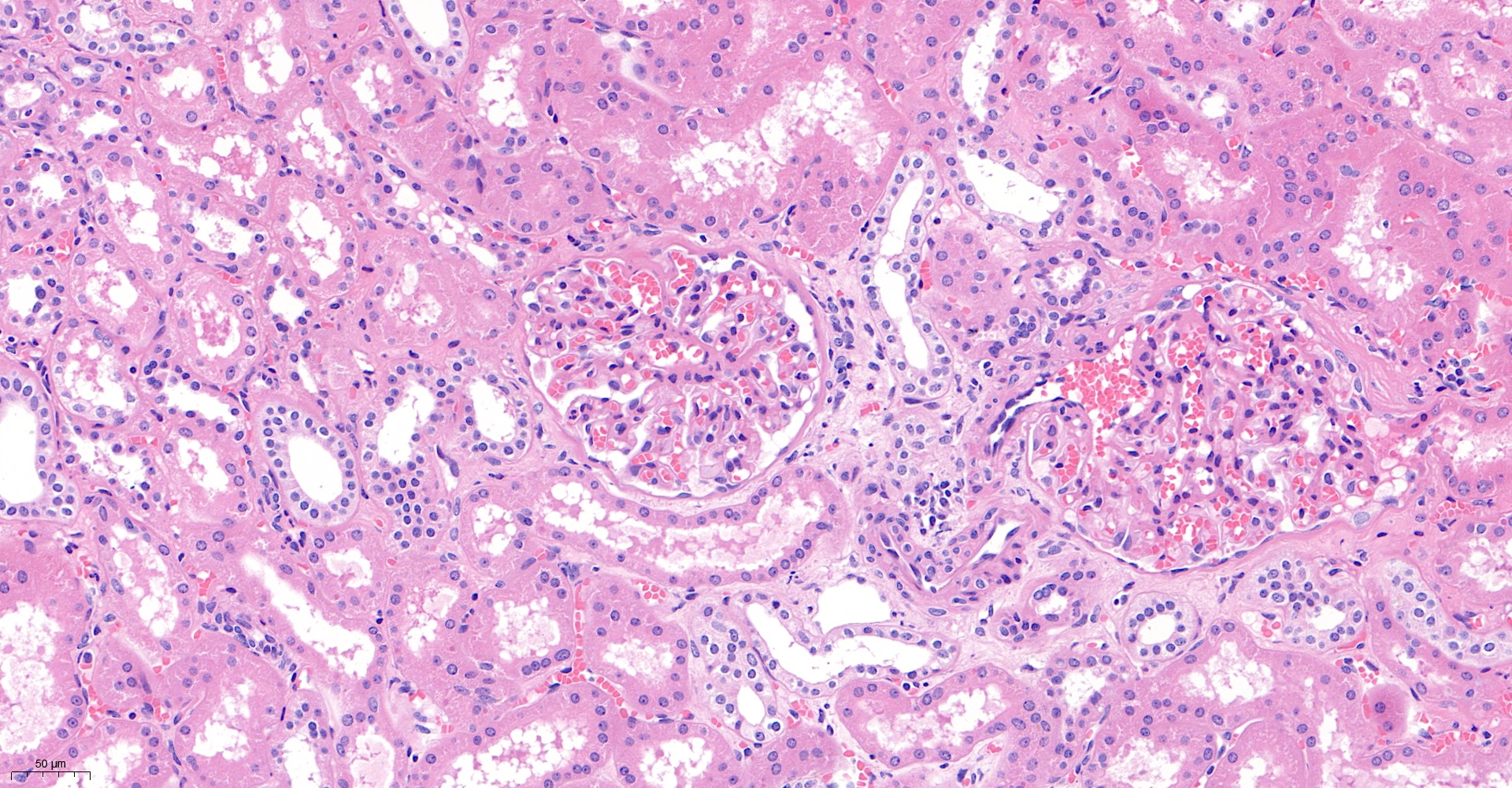

Supplement: Figure 5—figure supplement 3—source data 1. [file elife-70471-fig5-figsupp3-data1.zip › Figure 5-figure supplement 3-Source data 1/renal cancer patient 2/Raw data-HE staining image 1 of patient 2-20.0x.jpg]

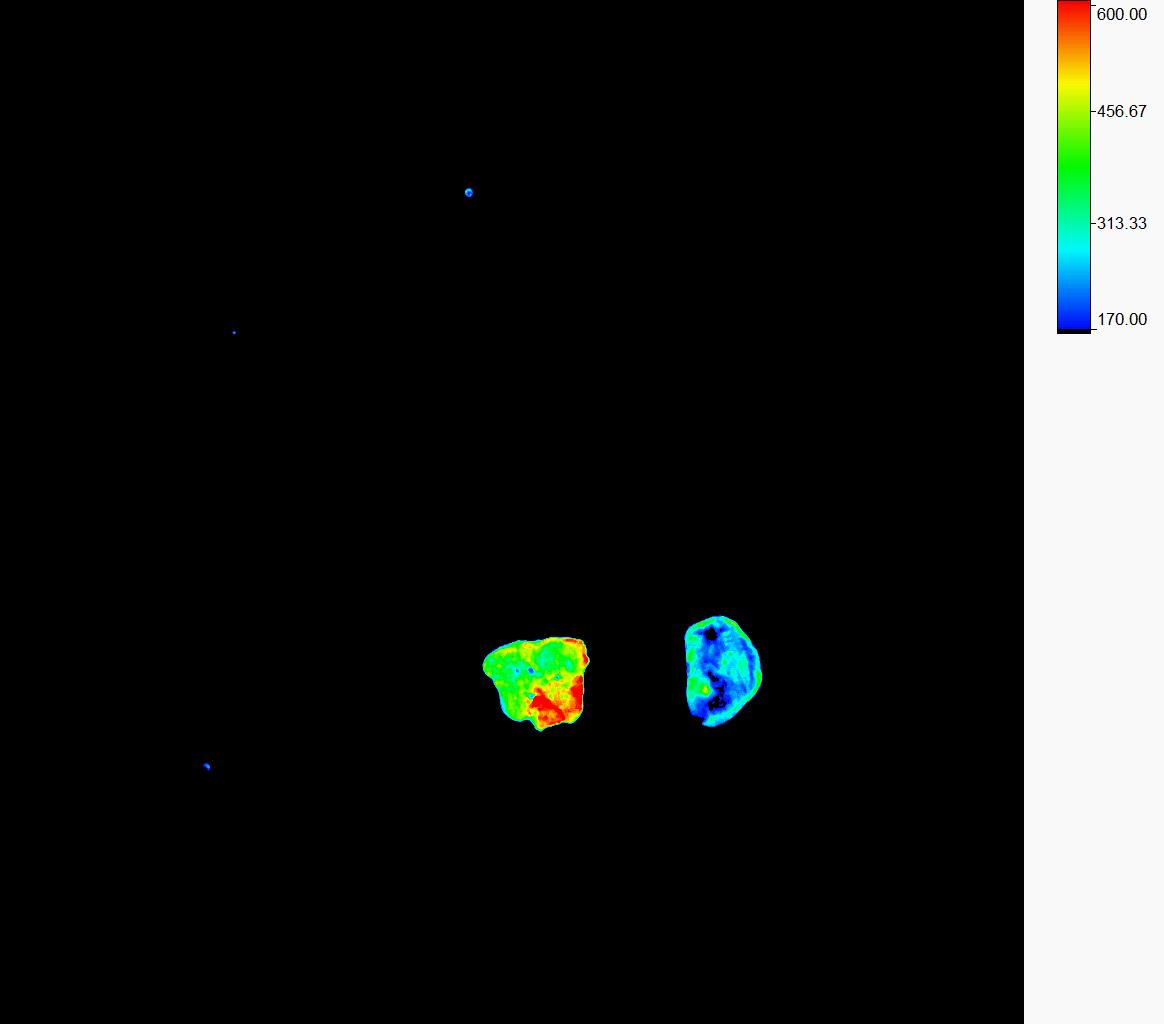

Supplement: Figure 5—figure supplement 3—source data 1. [file elife-70471-fig5-figsupp3-data1.zip › Figure 5-figure supplement 3-Source data 1/renal cancer patient 2/Raw data-nitroreductase detection image.jpg]

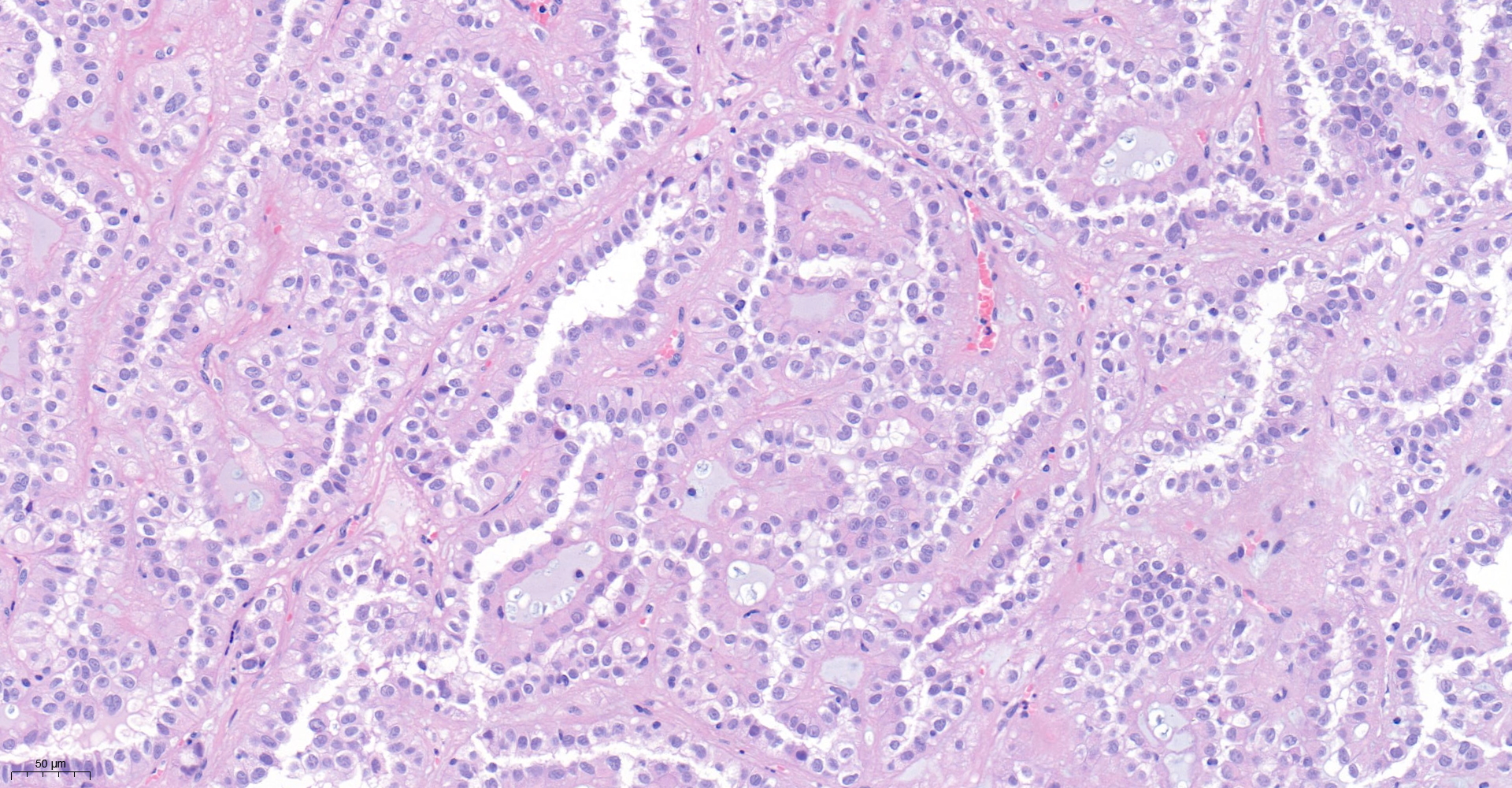

Supplement: Figure 5—figure supplement 3—source data 1. [file elife-70471-fig5-figsupp3-data1.zip › Figure 5-figure supplement 3-Source data 1/renal cancer patient 2/Raw data-HE staining image 2 of patient 2-20.0x.jpg]

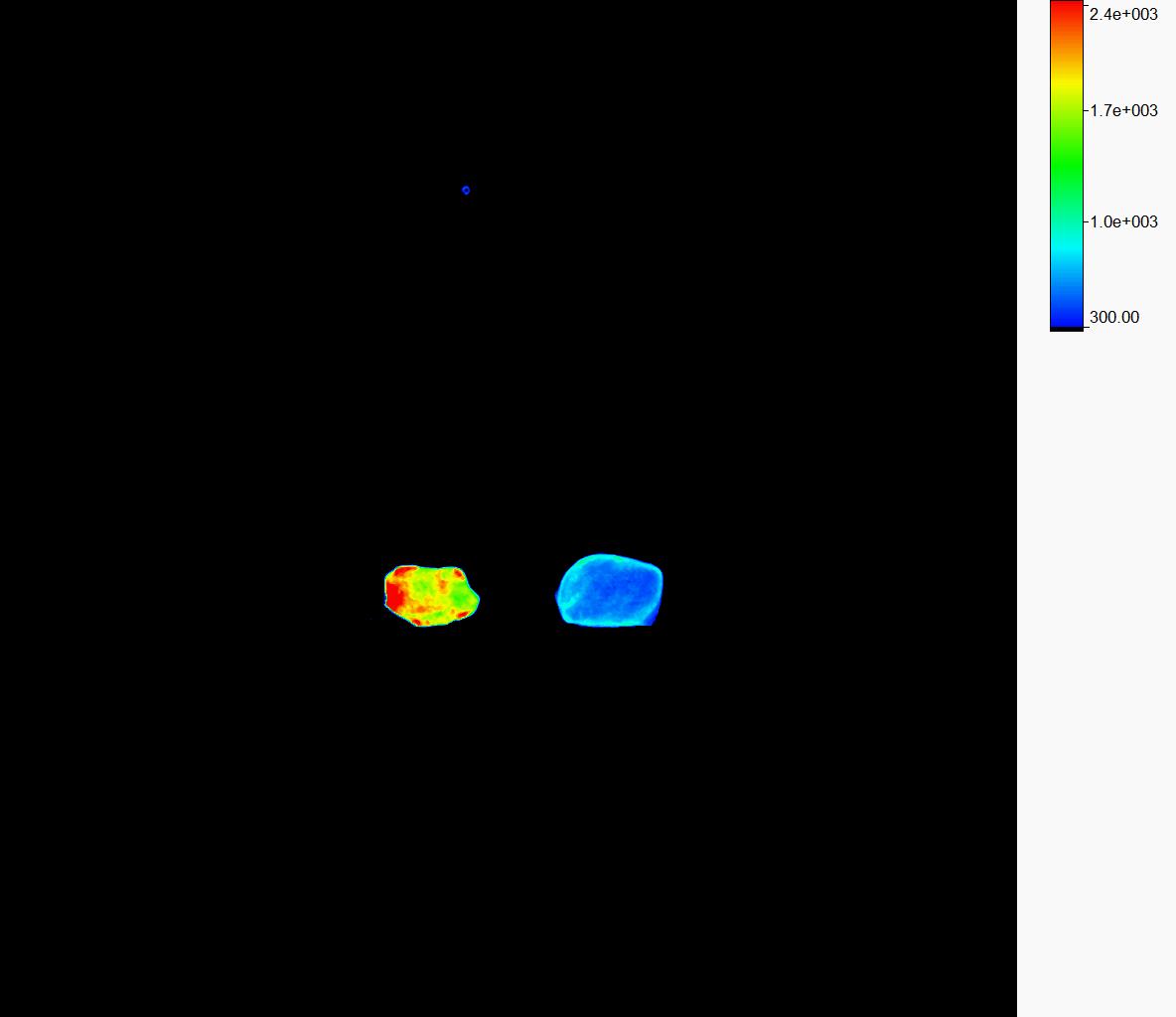

Supplement: Figure 5—figure supplement 3—source data 1. [file elife-70471-fig5-figsupp3-data1.zip › Figure 5-figure supplement 3-Source data 1/renal cancer patient 5/Raw data-viscosity detection image.jpg]

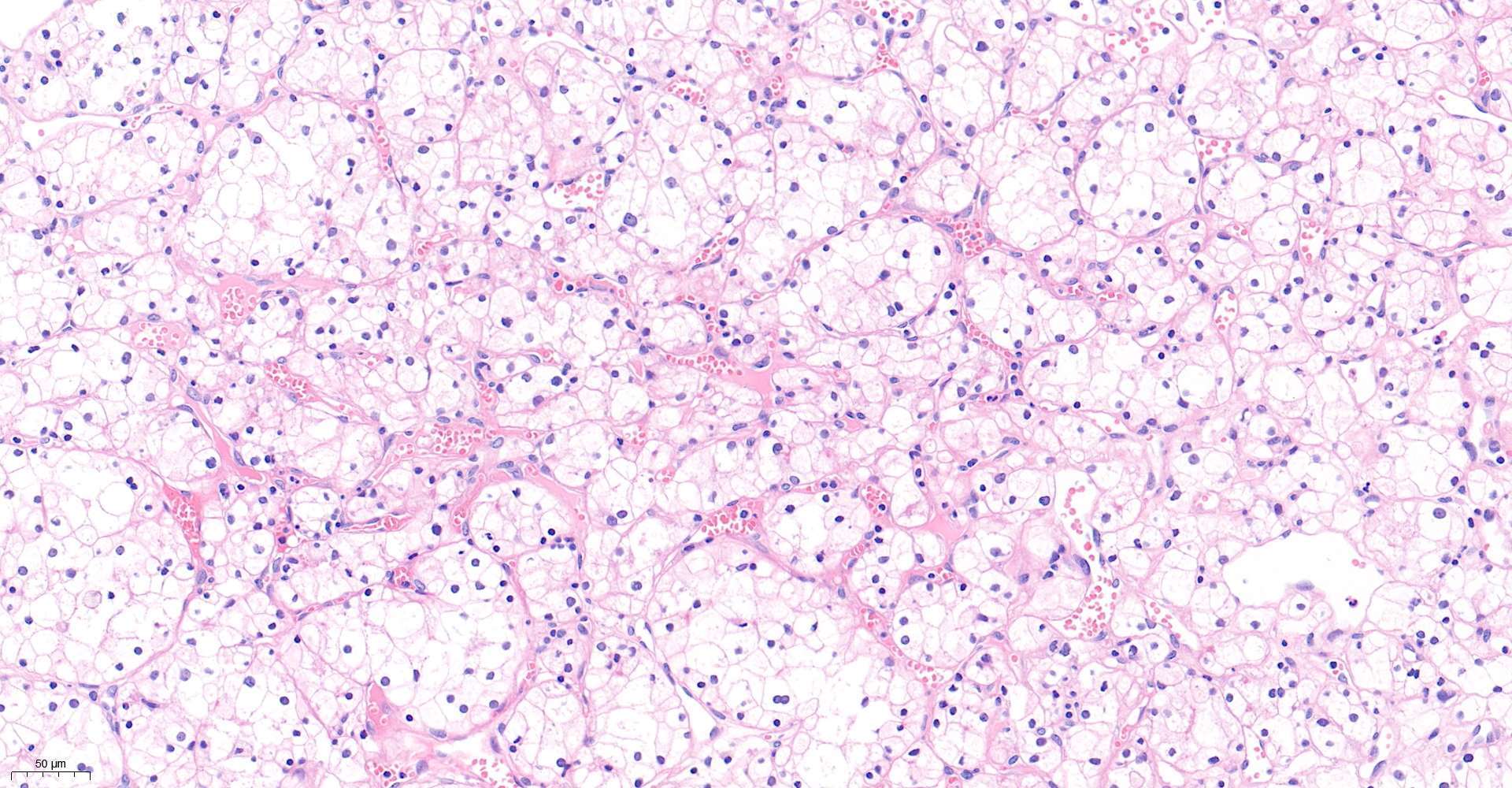

Supplement: Figure 5—figure supplement 3—source data 1. [file elife-70471-fig5-figsupp3-data1.zip › Figure 5-figure supplement 3-Source data 1/renal cancer patient 5/Raw data-HE staining image 2 of patient 5-20.0x.jpg]

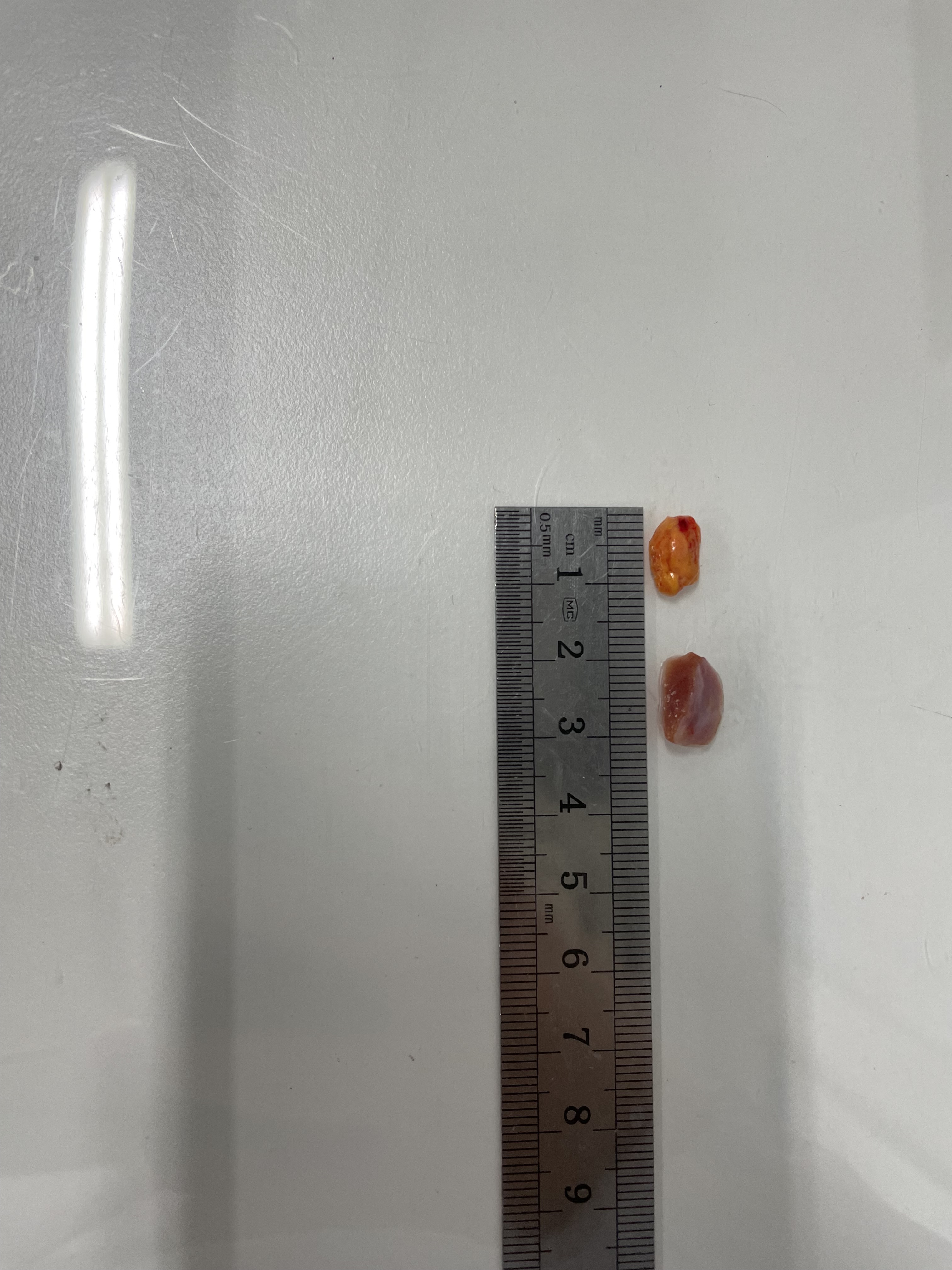

Supplement: Figure 5—figure supplement 3—source data 1. [file elife-70471-fig5-figsupp3-data1.zip › Figure 5-figure supplement 3-Source data 1/renal cancer patient 5/Raw data-photograph image.JPG]

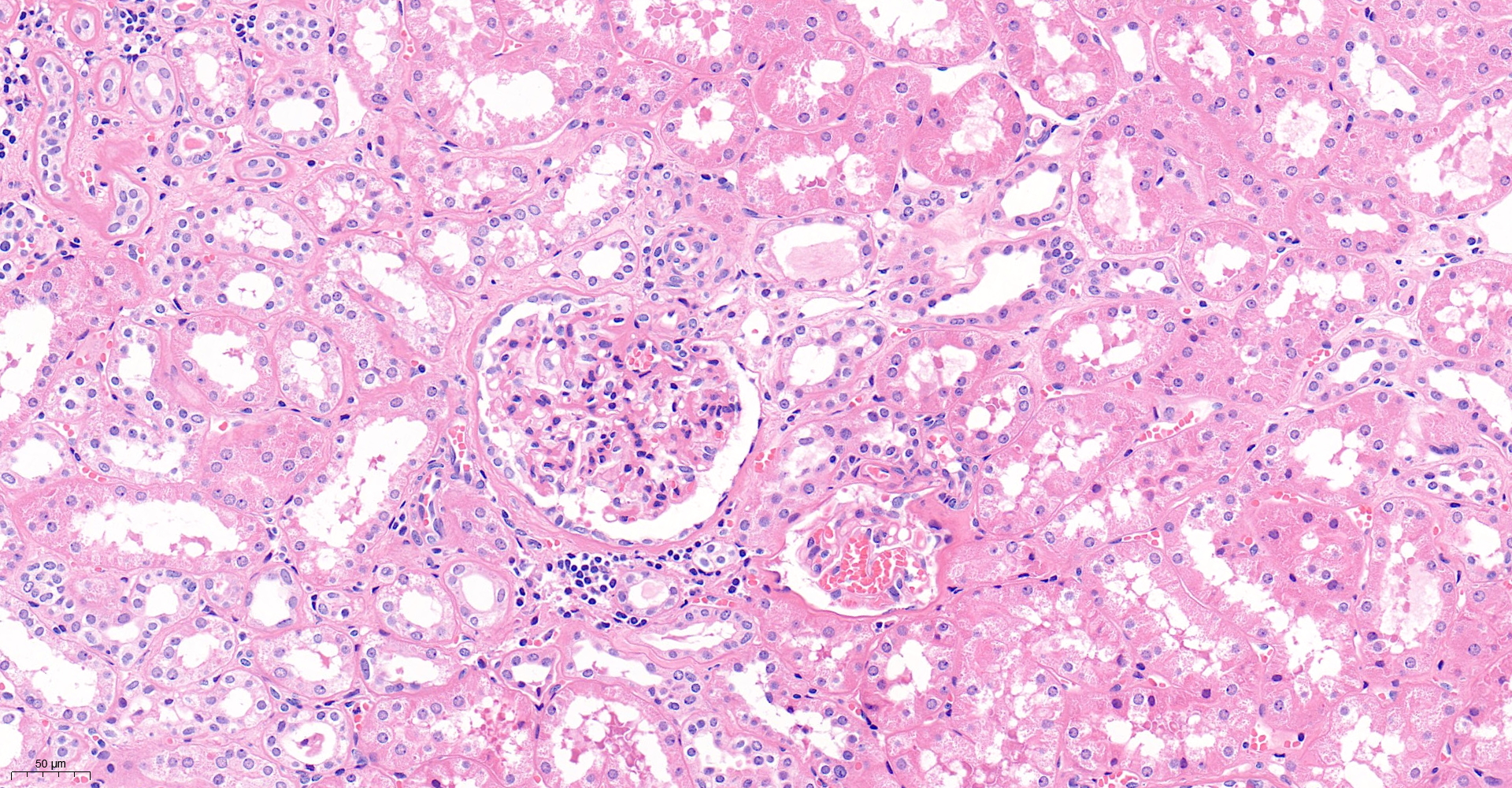

Supplement: Figure 5—figure supplement 3—source data 1. [file elife-70471-fig5-figsupp3-data1.zip › Figure 5-figure supplement 3-Source data 1/renal cancer patient 5/Raw data-HE staining image 1 of patient 5-20.0x.jpg]

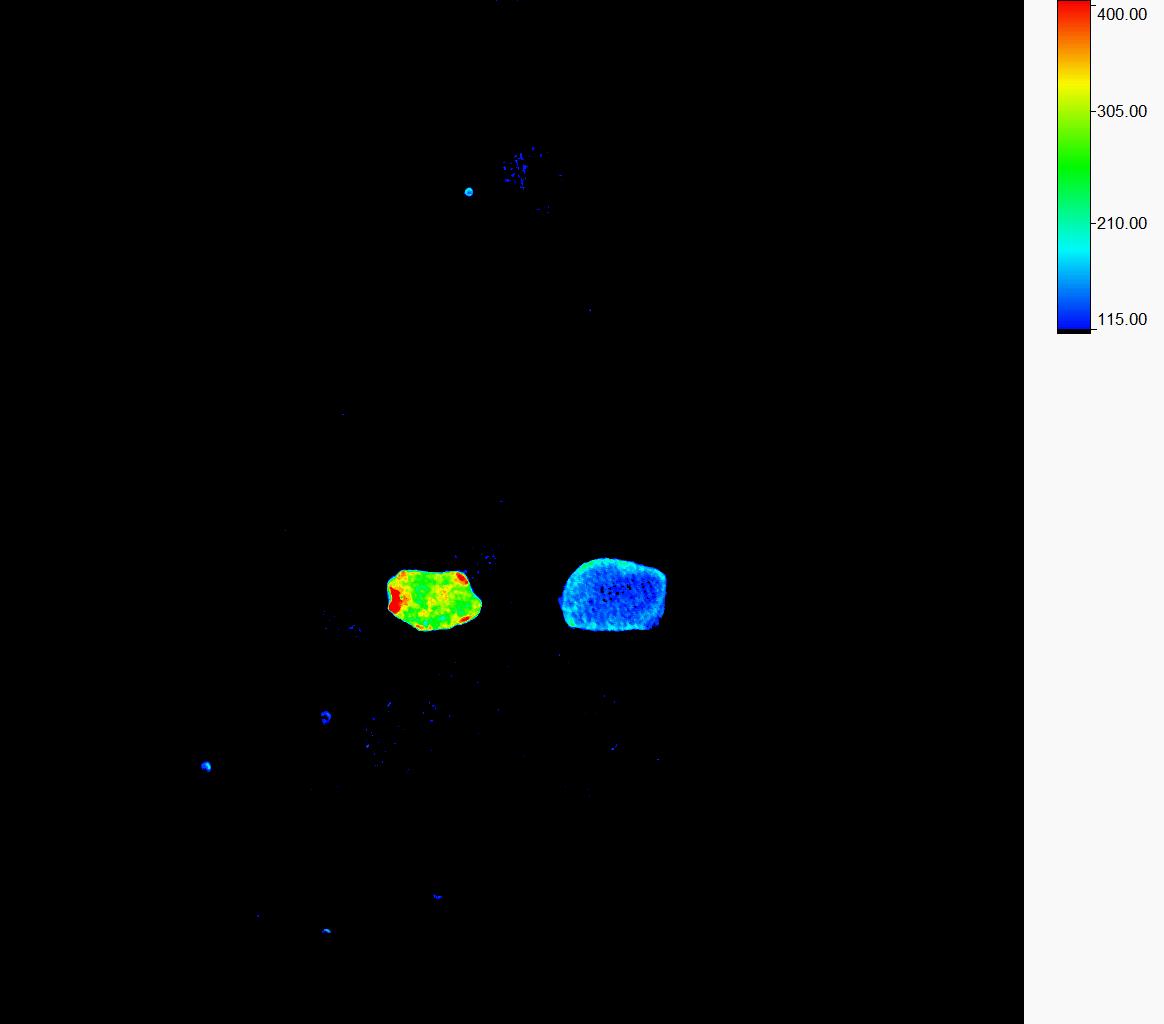

Supplement: Figure 5—figure supplement 3—source data 1. [file elife-70471-fig5-figsupp3-data1.zip › Figure 5-figure supplement 3-Source data 1/renal cancer patient 5/Raw data-nitroreductase detection image.jpg]

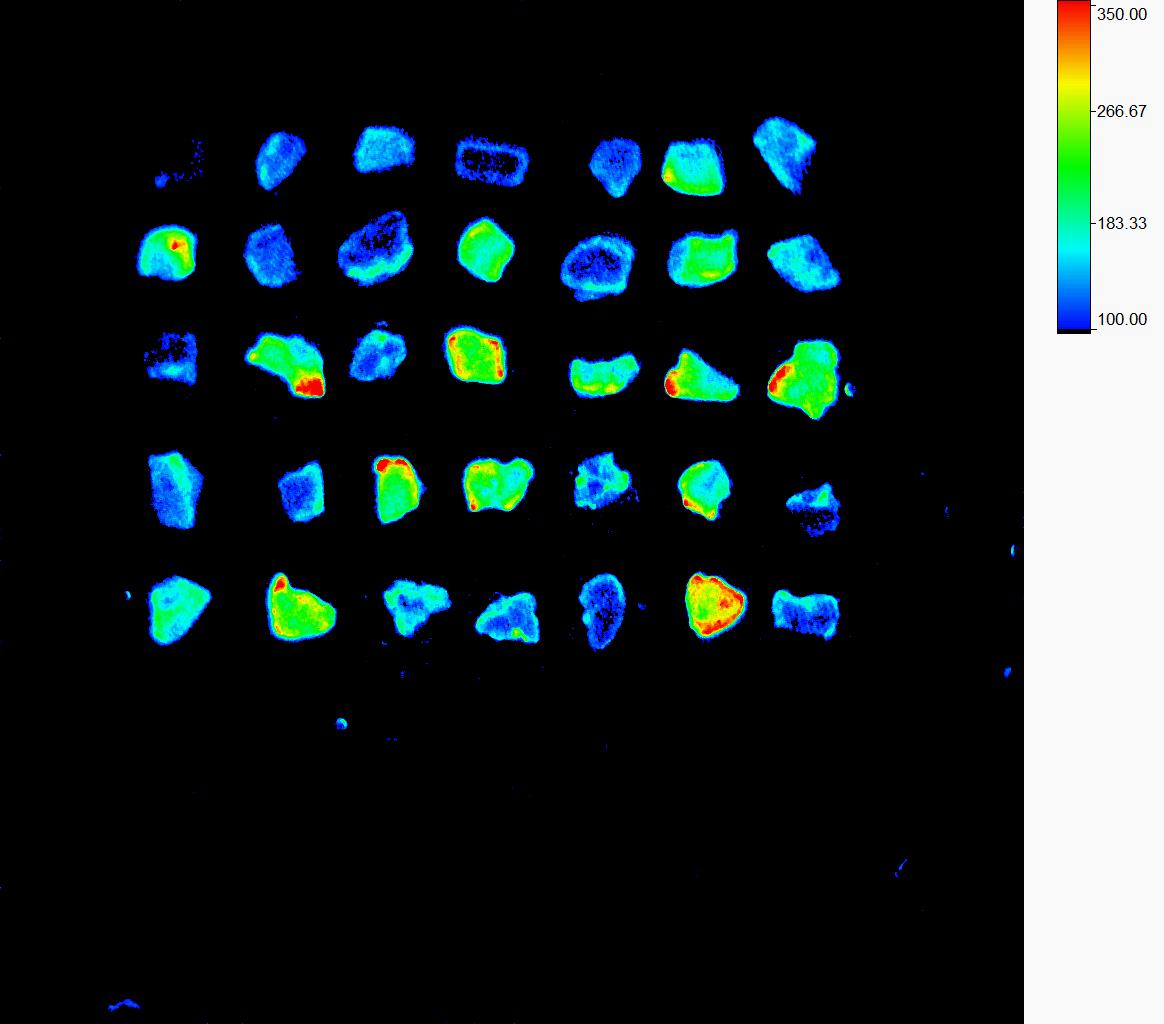

Supplement: Figure 6—source data 1. [file elife-70471-fig6-data1.zip › Figure 6-Source data/Raw data-nitroreductase detection image in Figure 8A.jpg]

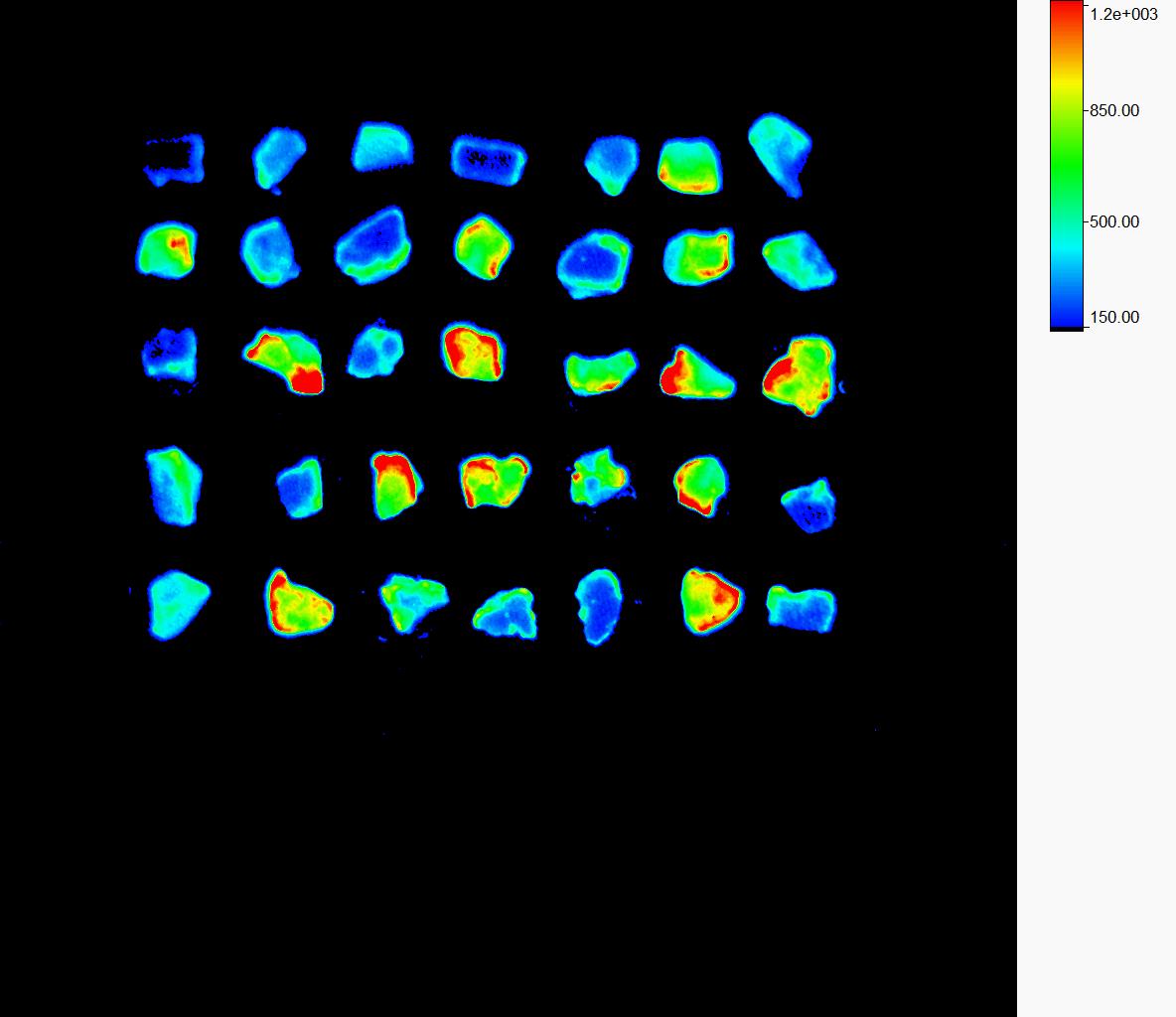

Supplement: Figure 6—source data 1. [file elife-70471-fig6-data1.zip › Figure 6-Source data/Raw data-viscosity detection image in Figure 8A.jpg]

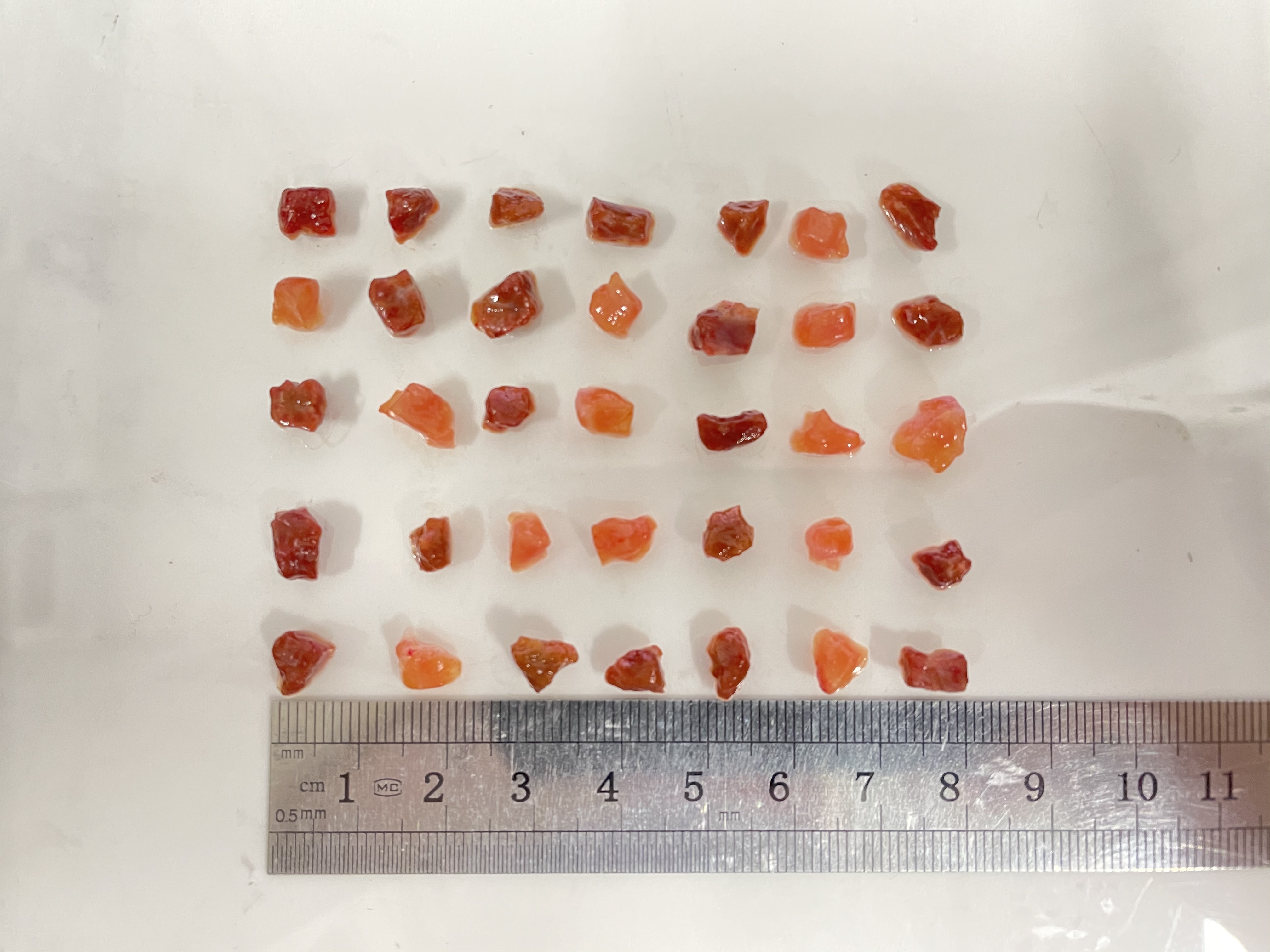

Supplement: Figure 6—source data 1. [file elife-70471-fig6-data1.zip › Figure 6-Source data/Raw data-photograph image in Figure 8A.jpeg]

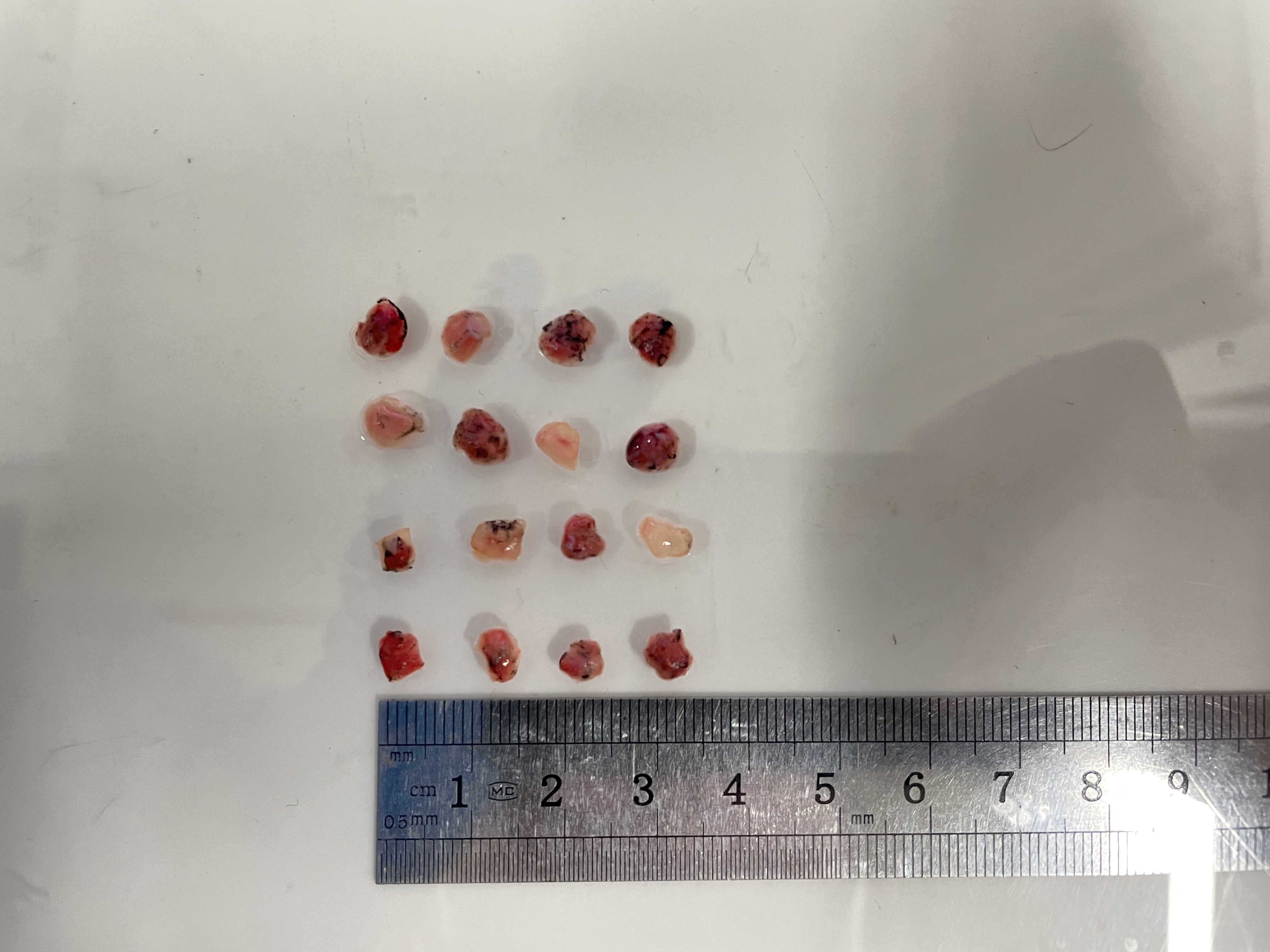

Supplement: Figure 7—source data 1. [file elife-70471-fig7-data1.zip › Figure 7-Source data/Raw data-photograph image in Figure 7A.jpeg]

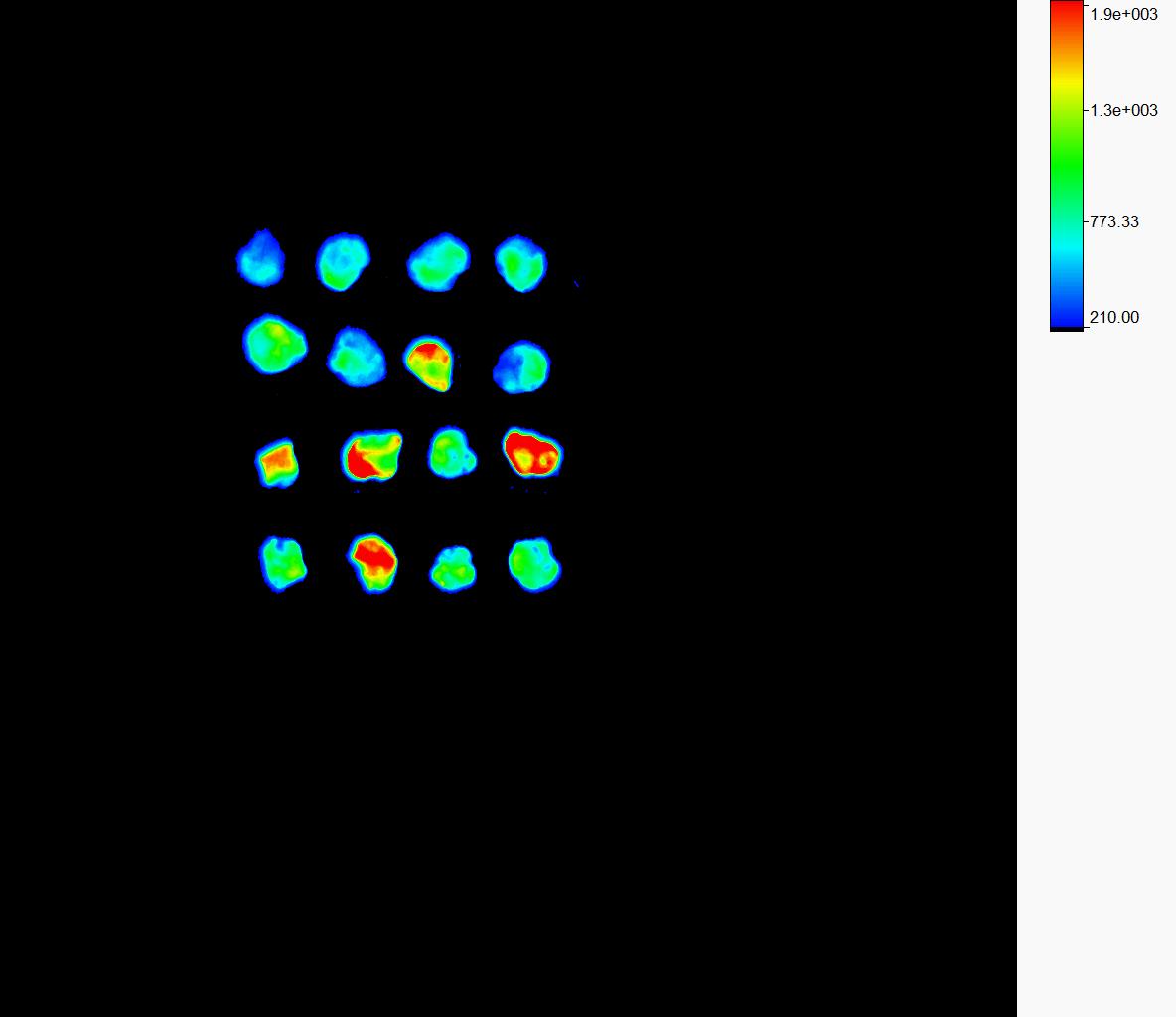

Supplement: Figure 7—source data 1. [file elife-70471-fig7-data1.zip › Figure 7-Source data/Raw data-viscosity detection image in Figure 7A.jpg]

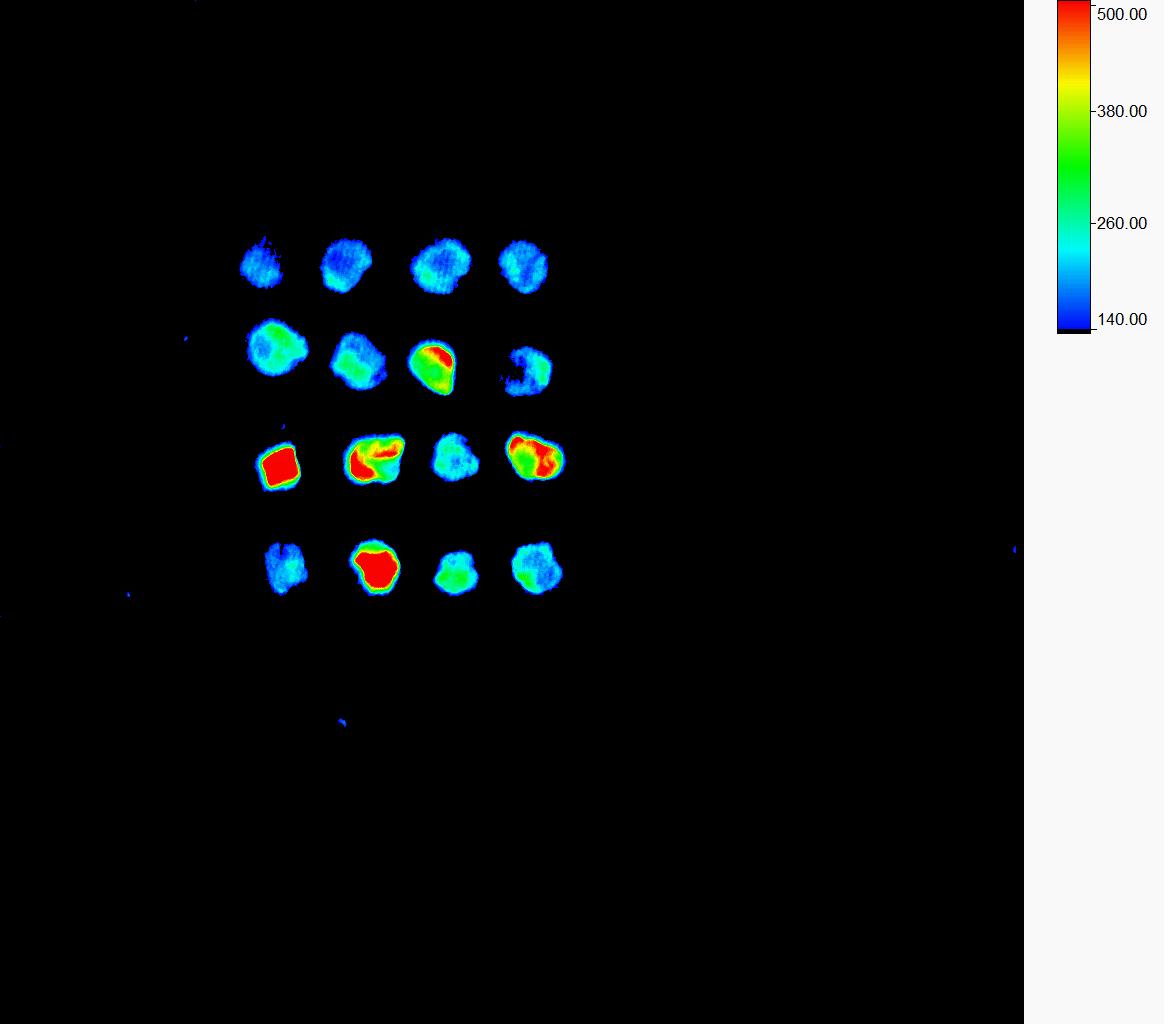

Supplement: Figure 7—source data 1. [file elife-70471-fig7-data1.zip › Figure 7-Source data/Raw data-nitroreductase detection image in Figure 7A.jpg]

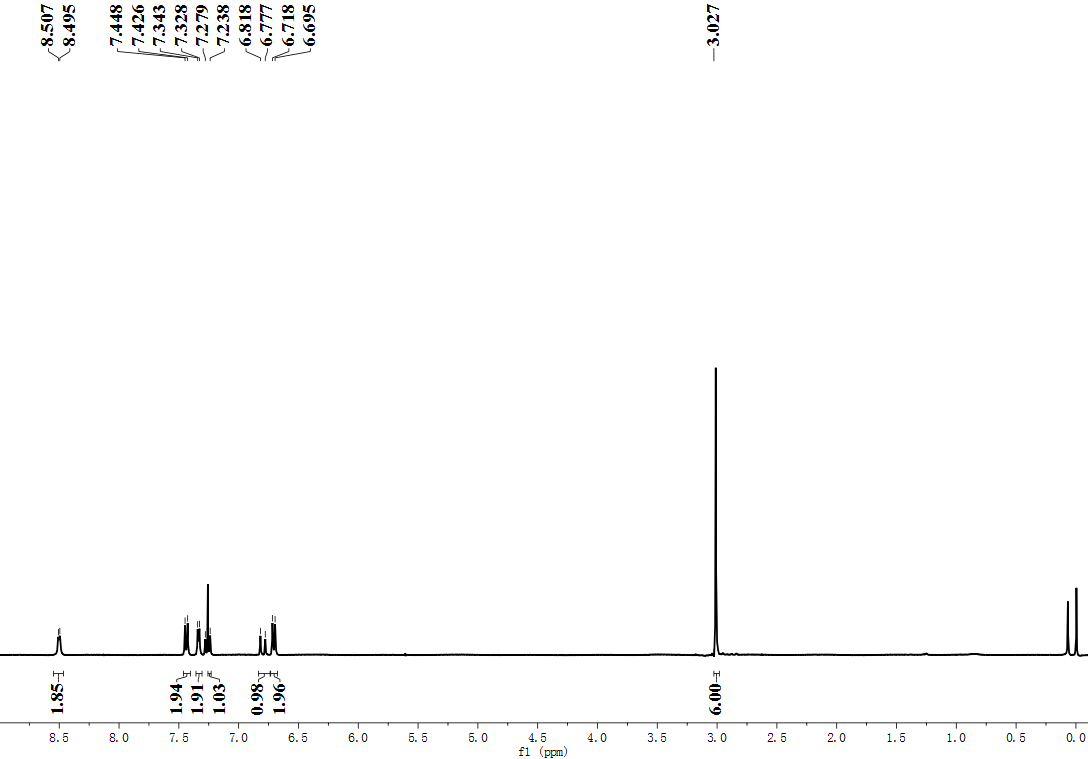

Supplement: Source data 1. [file elife-70471-supp2.zip › Supplementary File 1-Source data/IBS224-H.tiff]

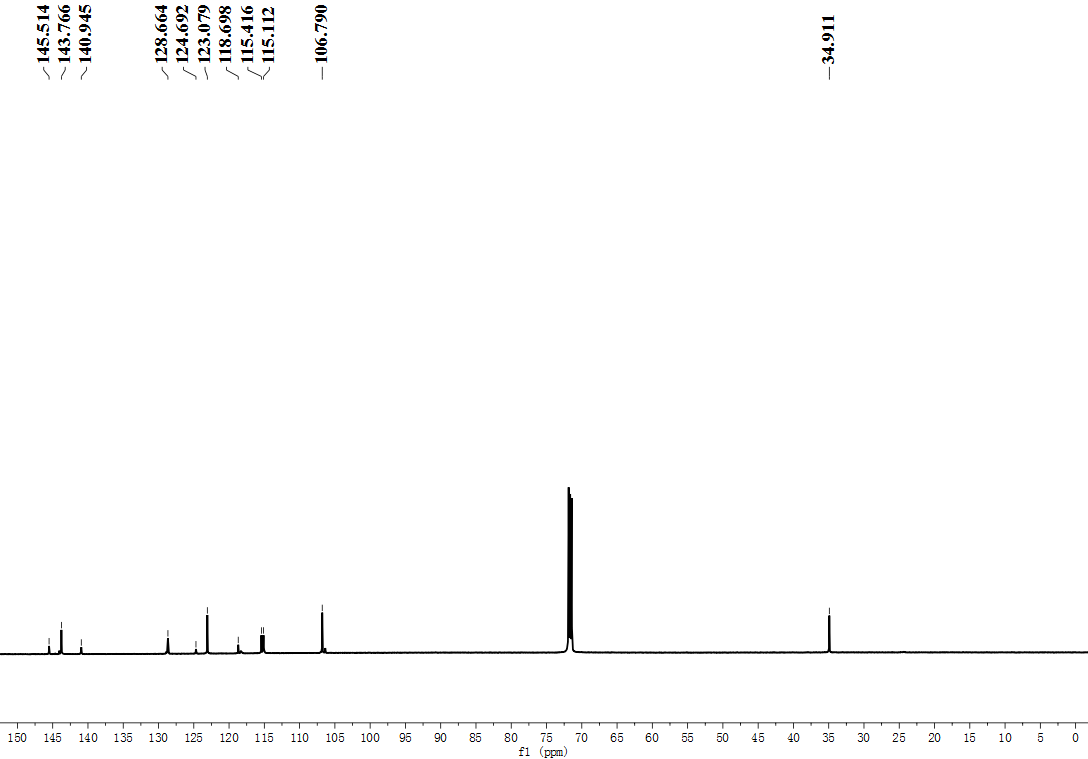

Supplement: Source data 1. [file elife-70471-supp2.zip › Supplementary File 1-Source data/IBS224-C.tiff]

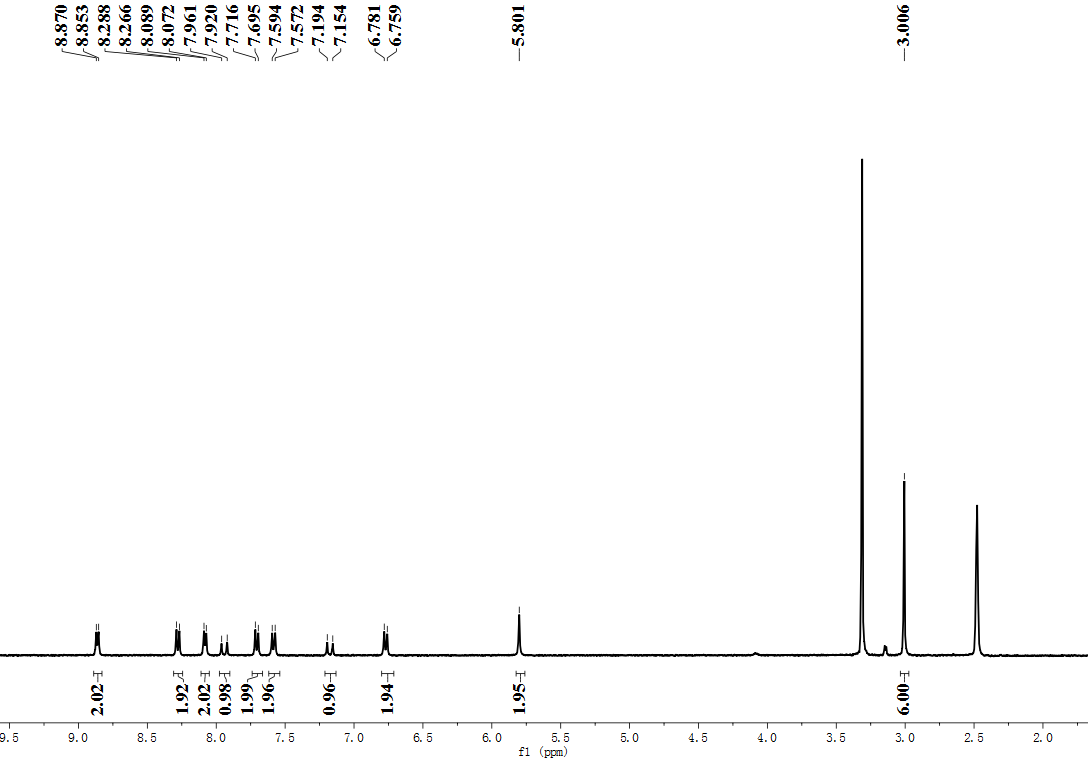

Supplement: Source data 1. [file elife-70471-supp2.zip › Supplementary File 1-Source data/IBS440-H.tiff]

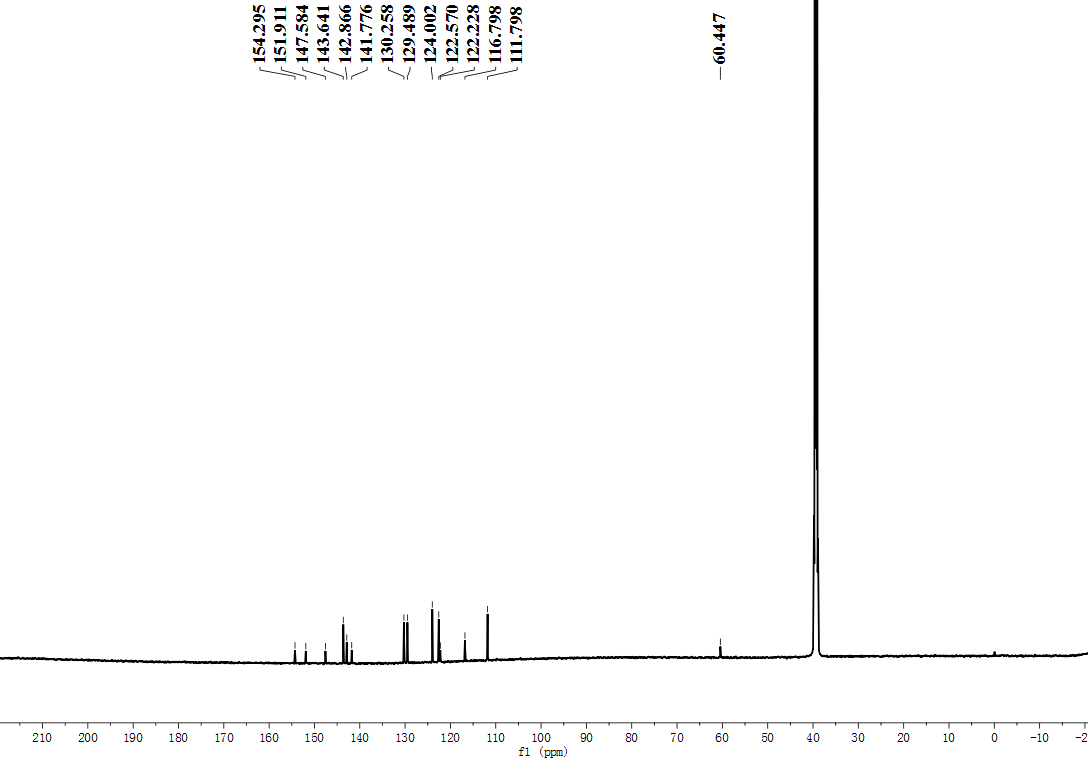

Supplement: Source data 1. [file elife-70471-supp2.zip › Supplementary File 1-Source data/IBS440-c.tiff]
