# Supplementary material for: A pre-screening strategy to assess resected tumor margins by imaging cytoplasmic viscosity and hypoxia
Source: eLife. 2021 Oct 11;10:e70471. doi: 10.7554/eLife.70471 (PMC8553343; doi:10.7554/eLife.70471)

Raw data of the hematoxylin and eosin scanning images (12G, 35 files) are available through the following links.

Microsoft one drive:

<https://emailcpcc-my.sharepoint.com/:u:/g/personal/mjohn123_email_cpcc_edu/EeIbNAErCqFPlsEFyYuny48BnxD12vmzCP0rHgusda6joA?e=xBUq0Y> Password:8545

Or personal cloud drive:

<http://IBSlab.quickconnect.cn/d/s/621772215557338803/J0BX7Cc3pOAaZacr-6zTtK6IaSZqHkW4-lbwA-8T6oAg_> Password:8545

Raw data files can be open by the software (Pannoramic Scanner).

Thumbnail images of all 35 samples are presented below.

Scale bar: 500 μm.

Sample 1.


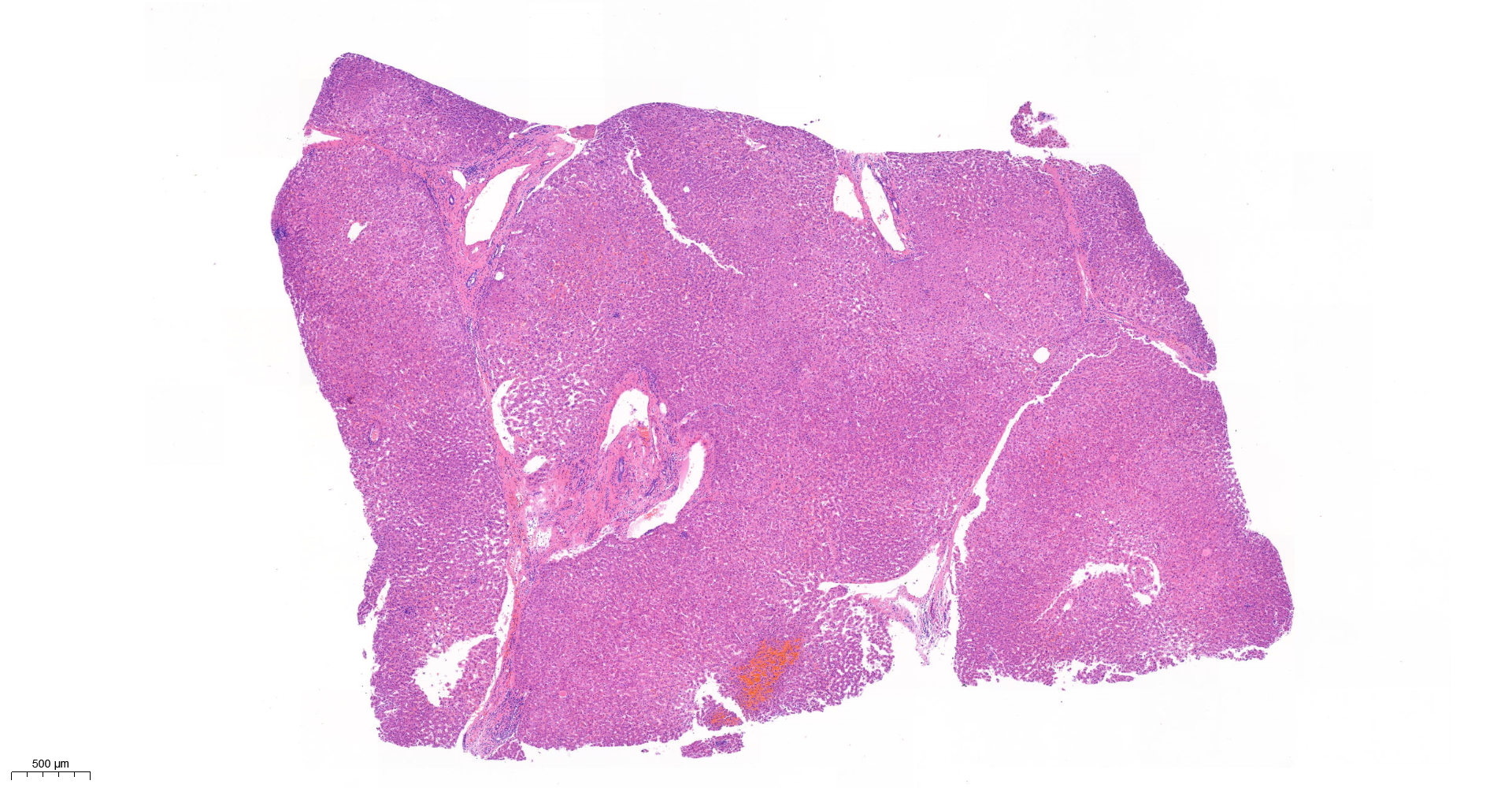


Sample 2.


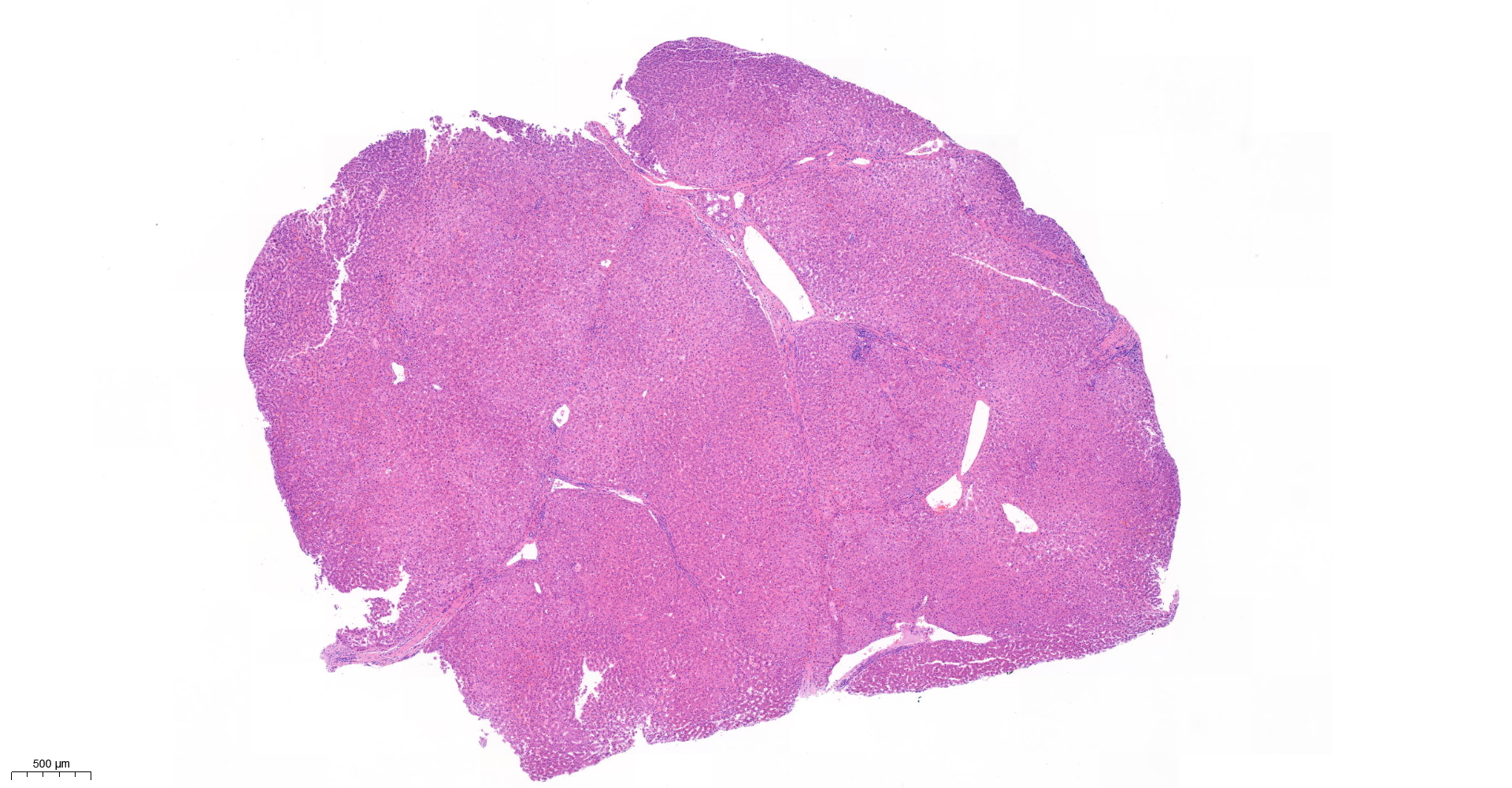


Sample 3.


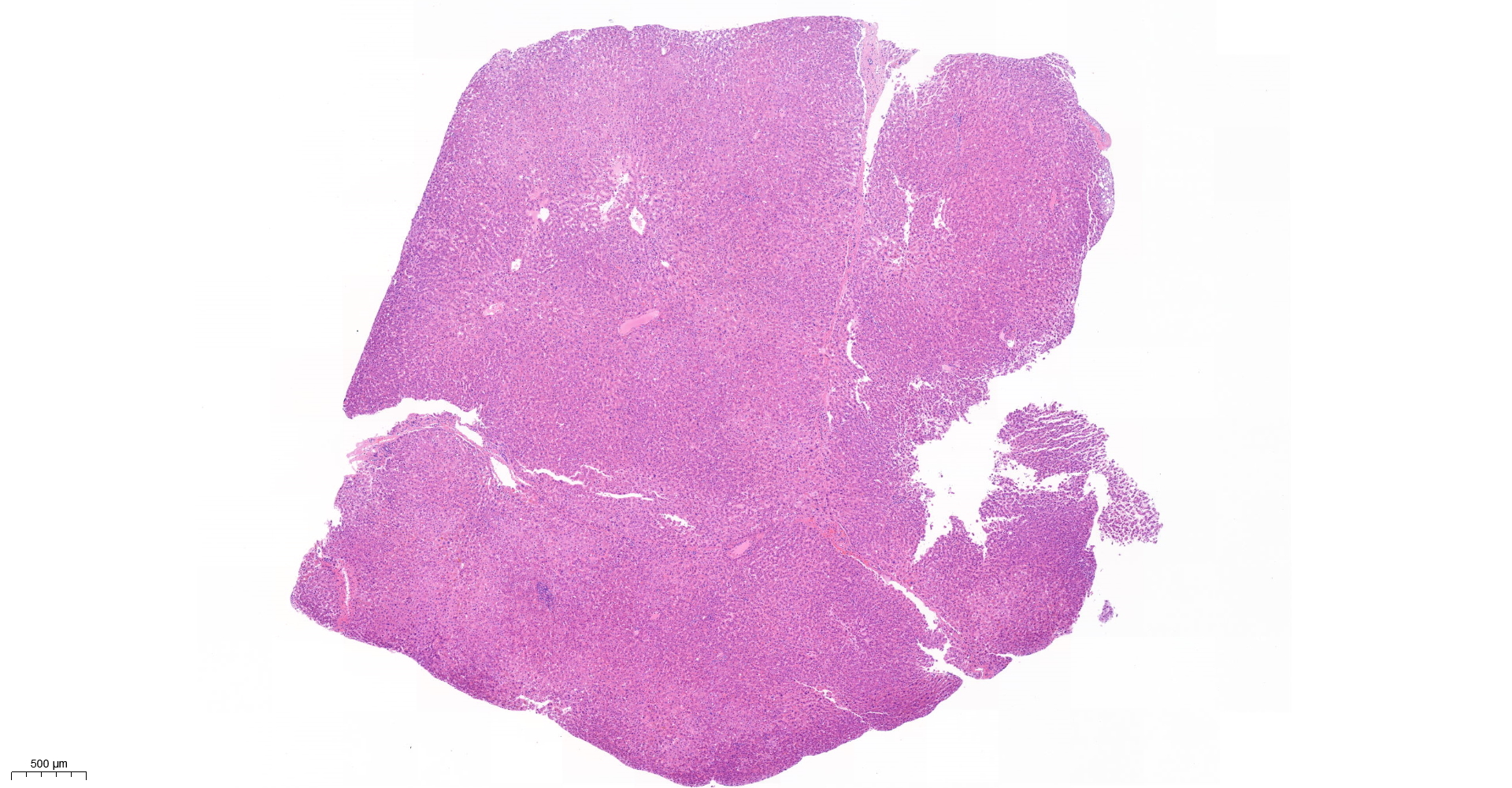


Sample 4.


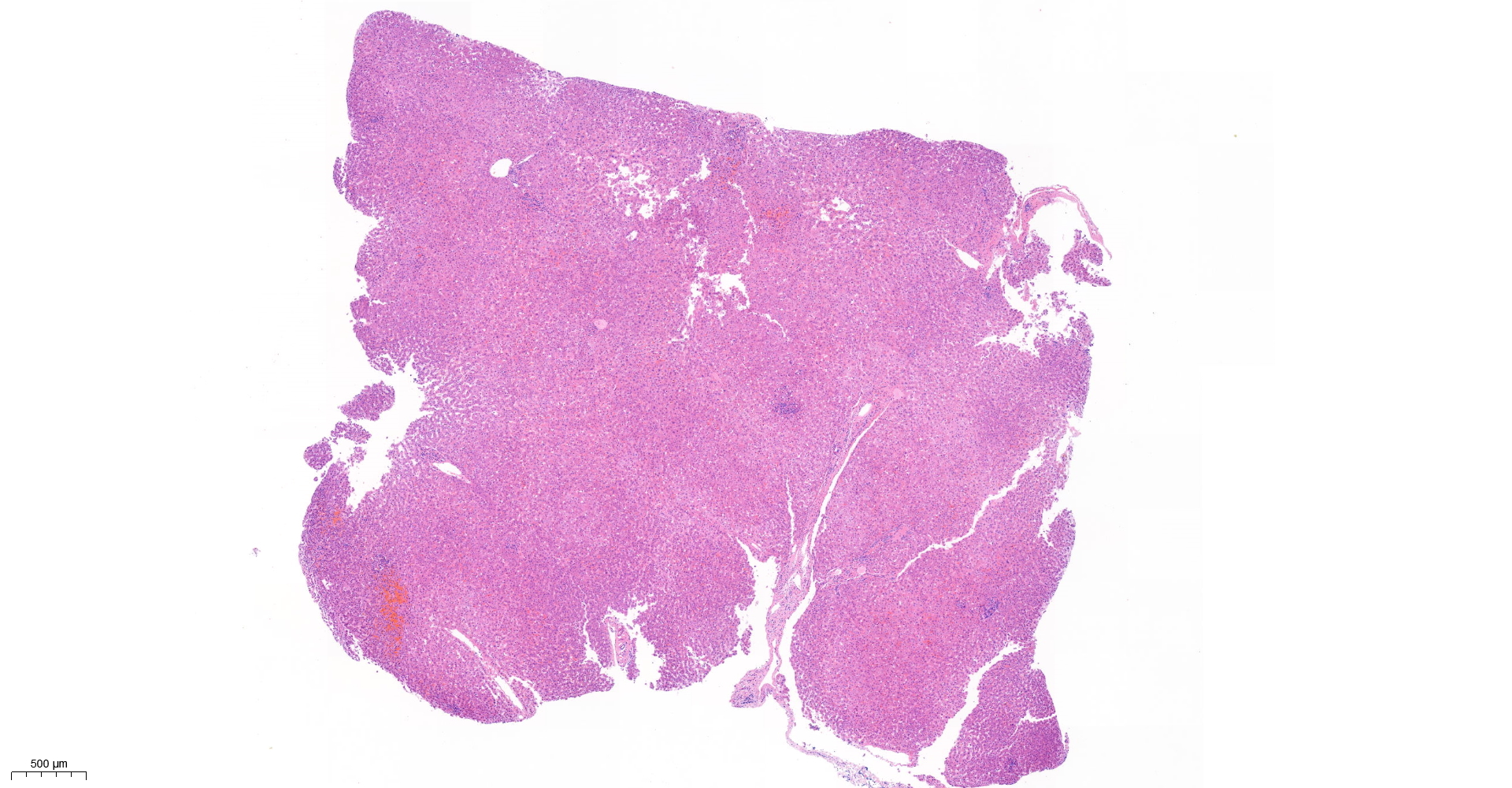


Sample 5.


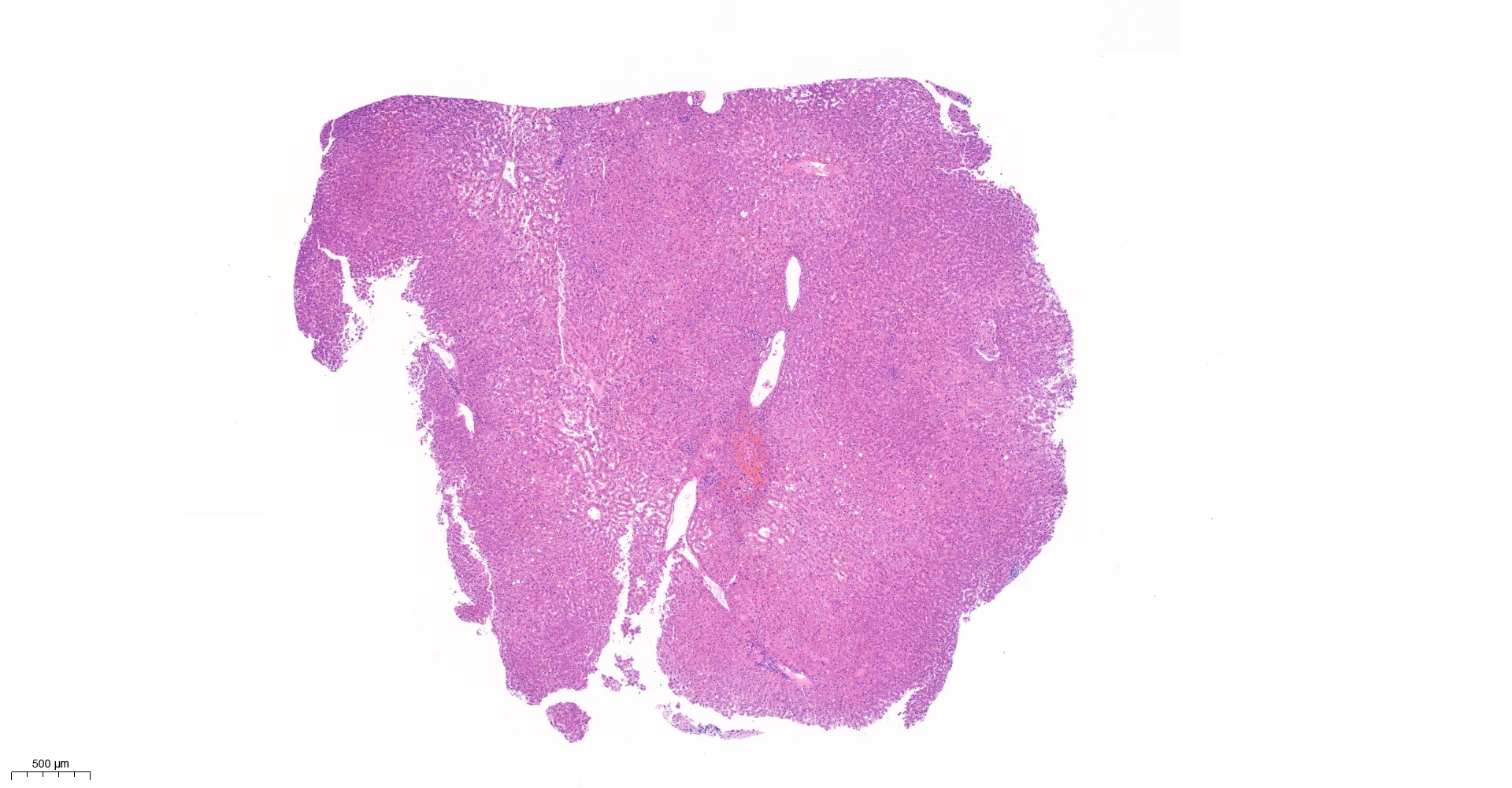


Sample 6.


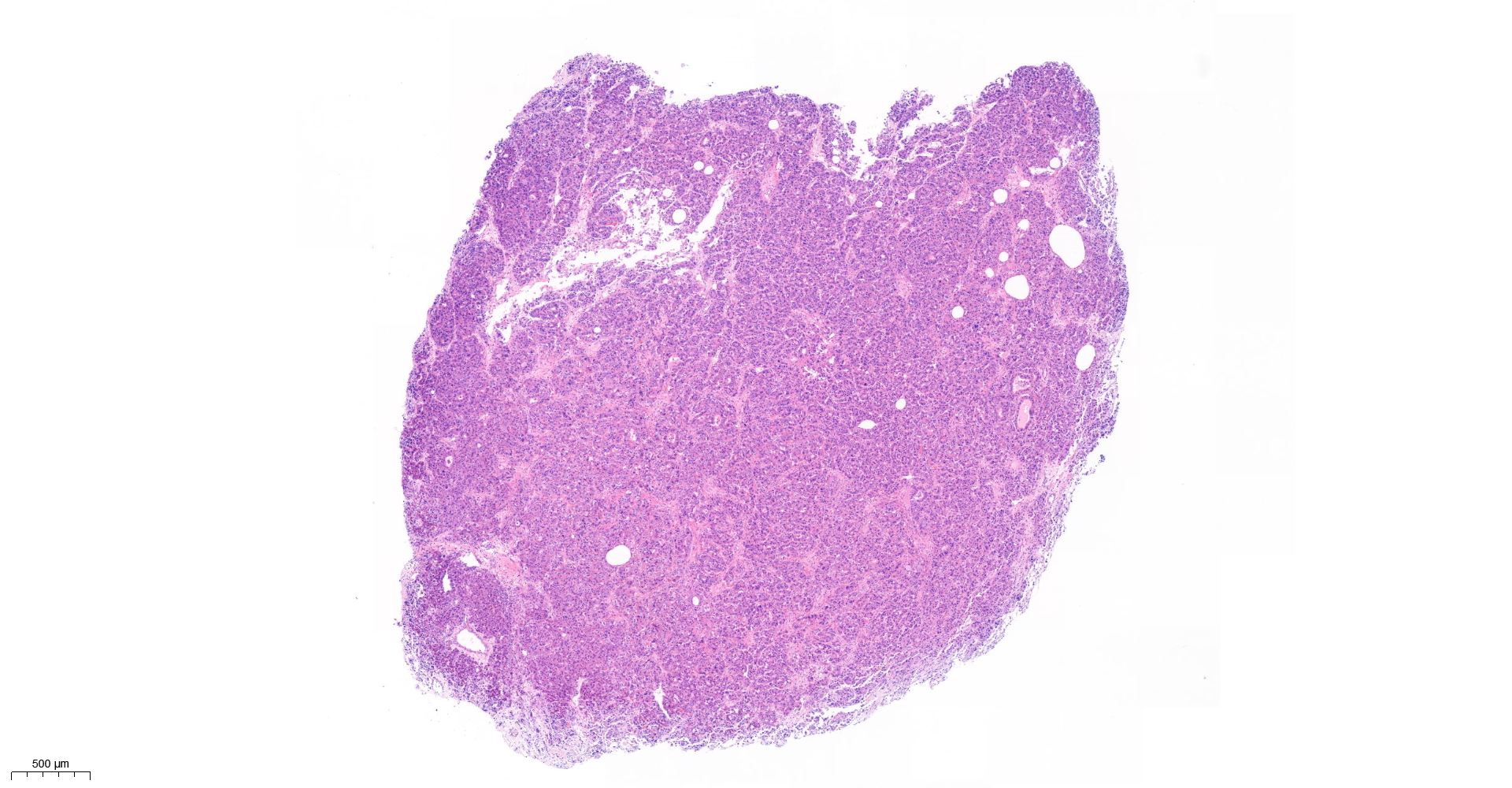


Sample 7.


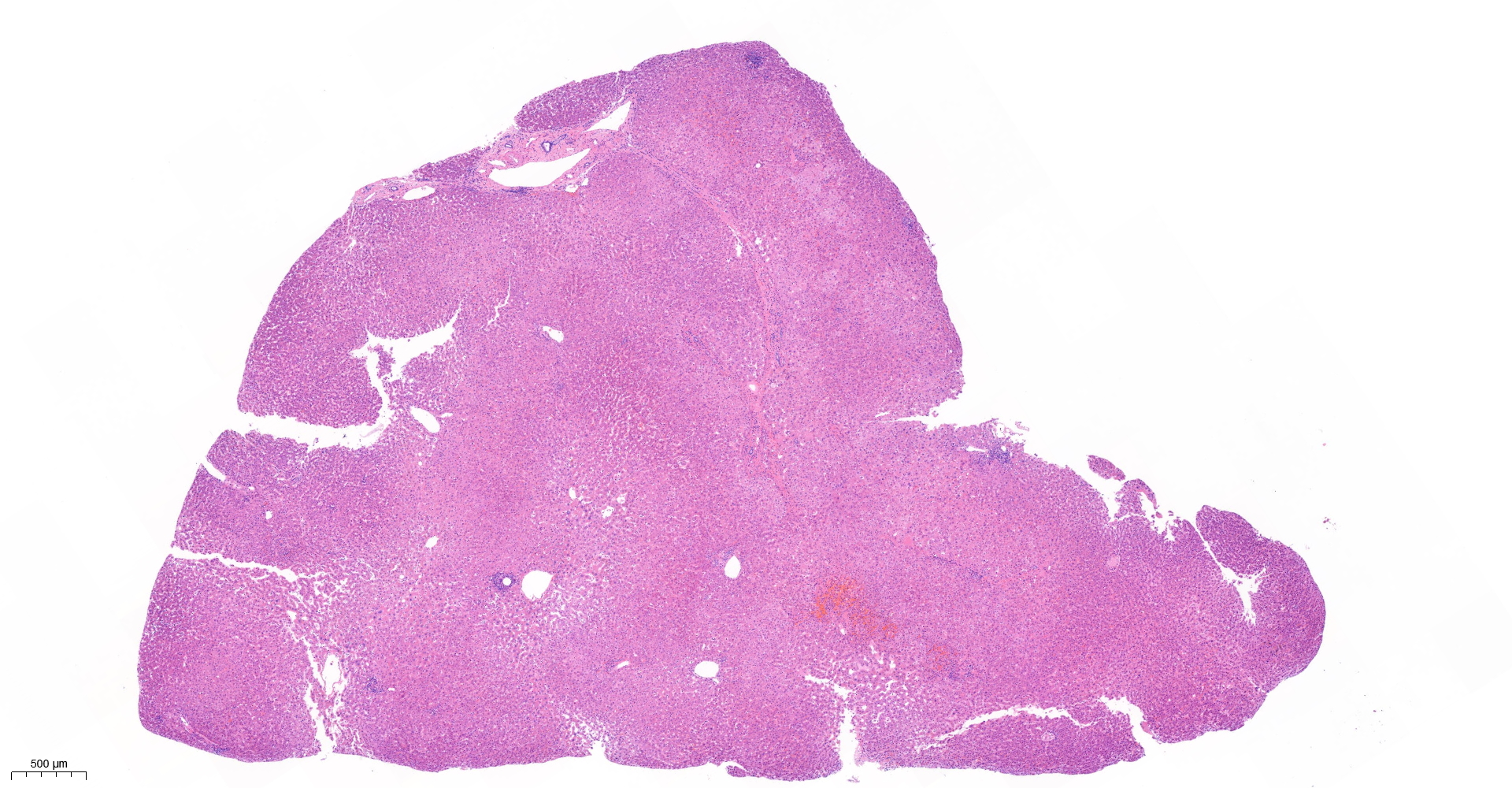


Sample 8.


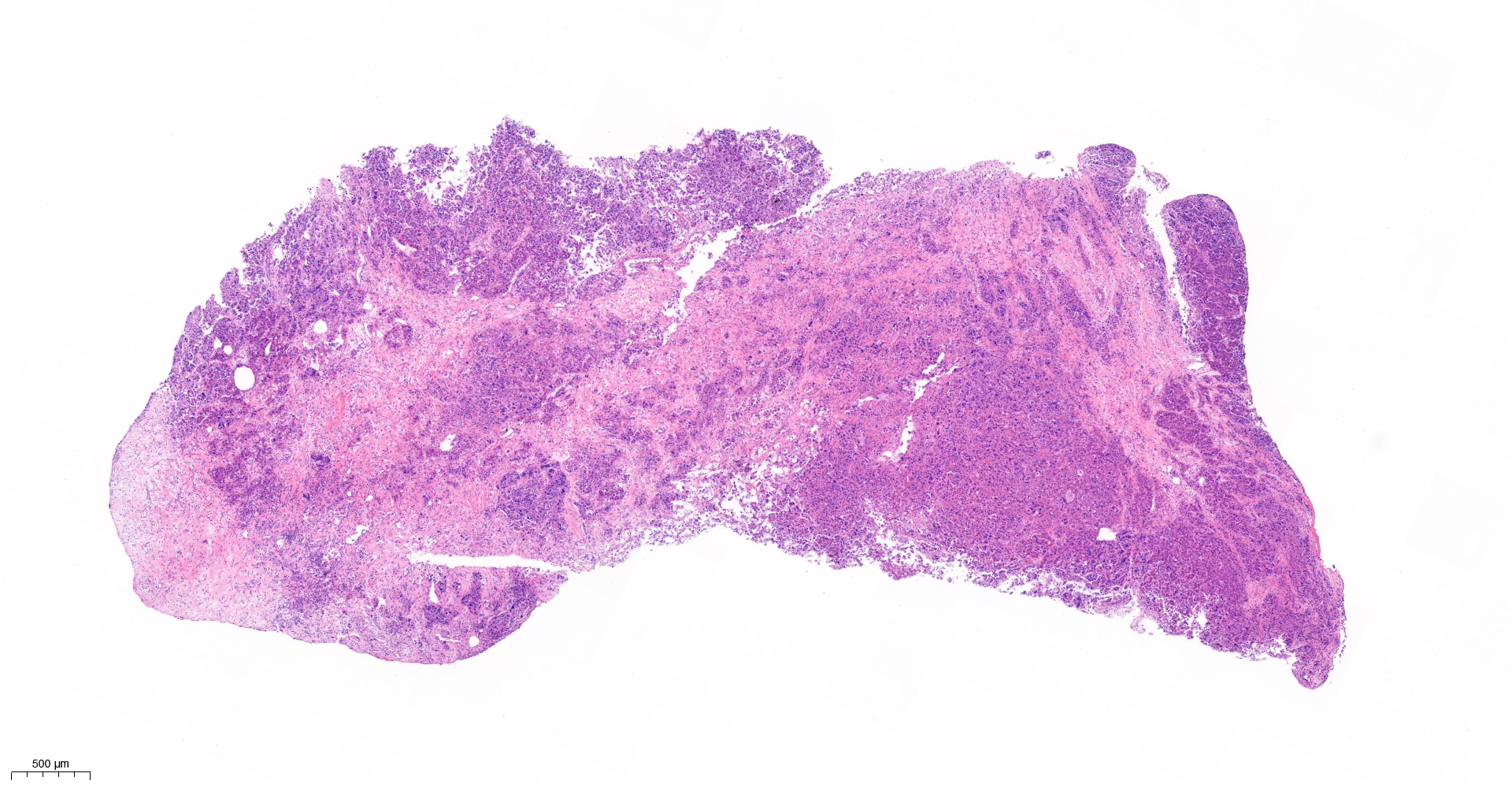


Sample 9.


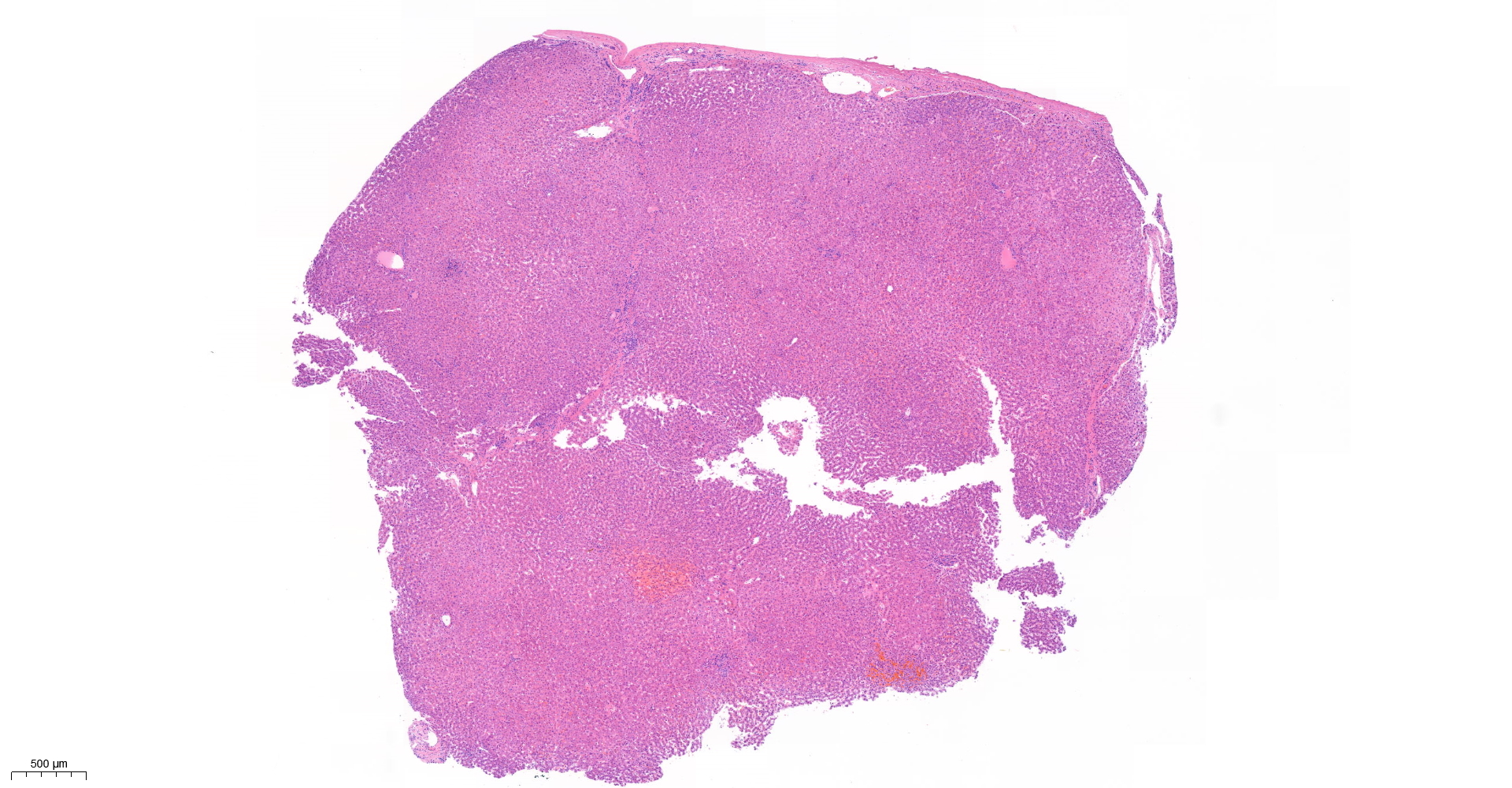


Sample 10.


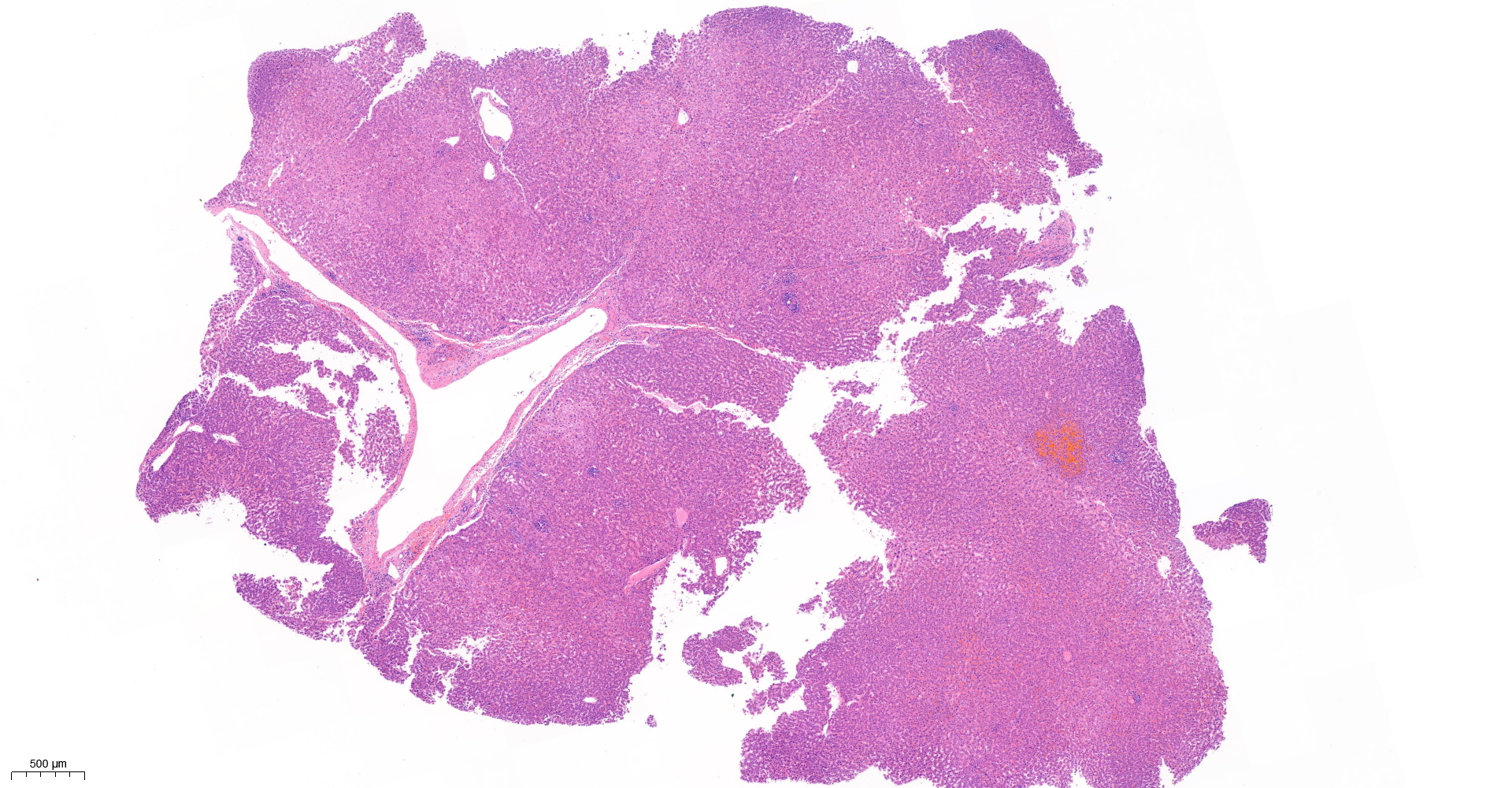


Sample 11.


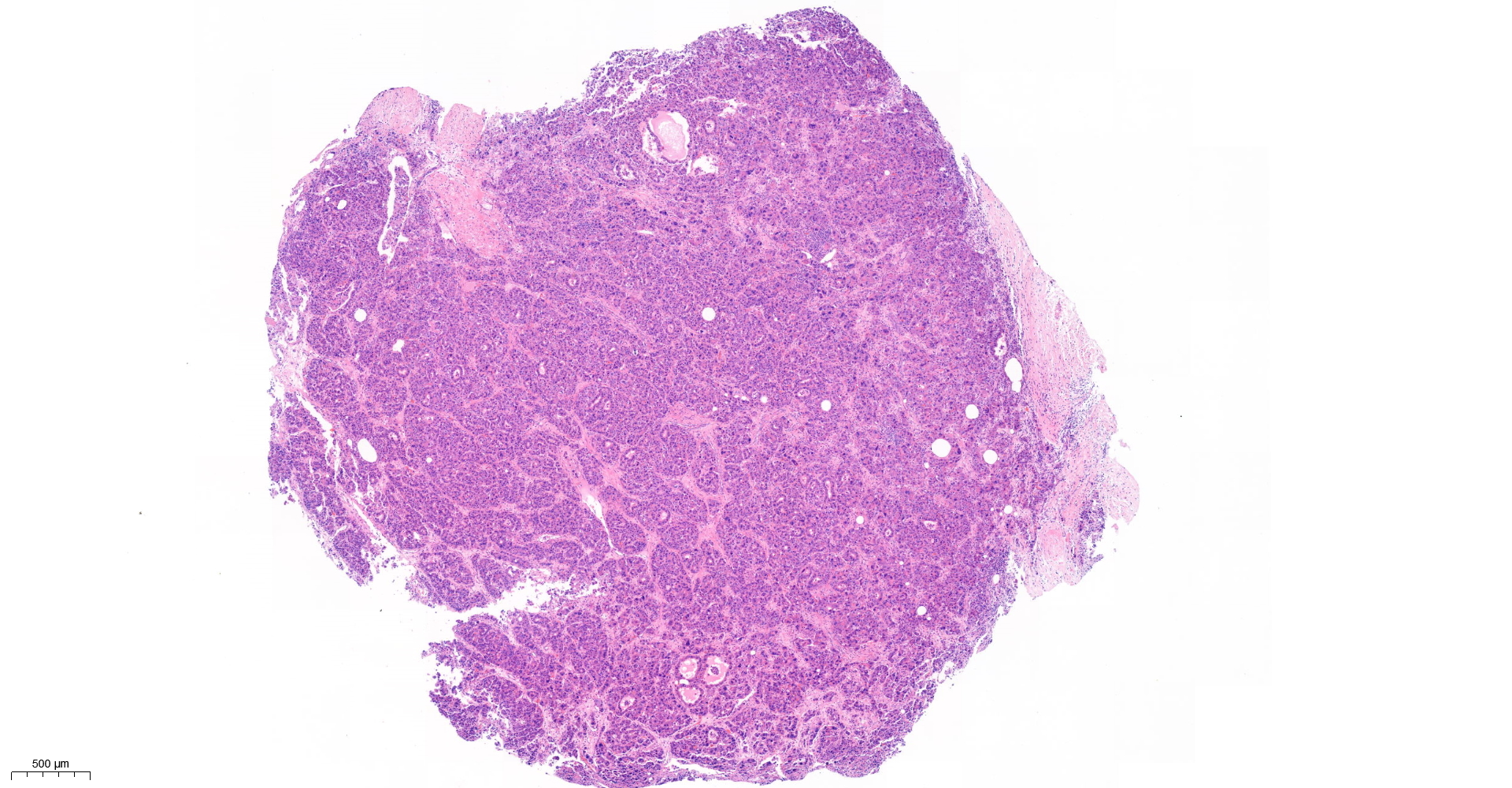


Sample 12.


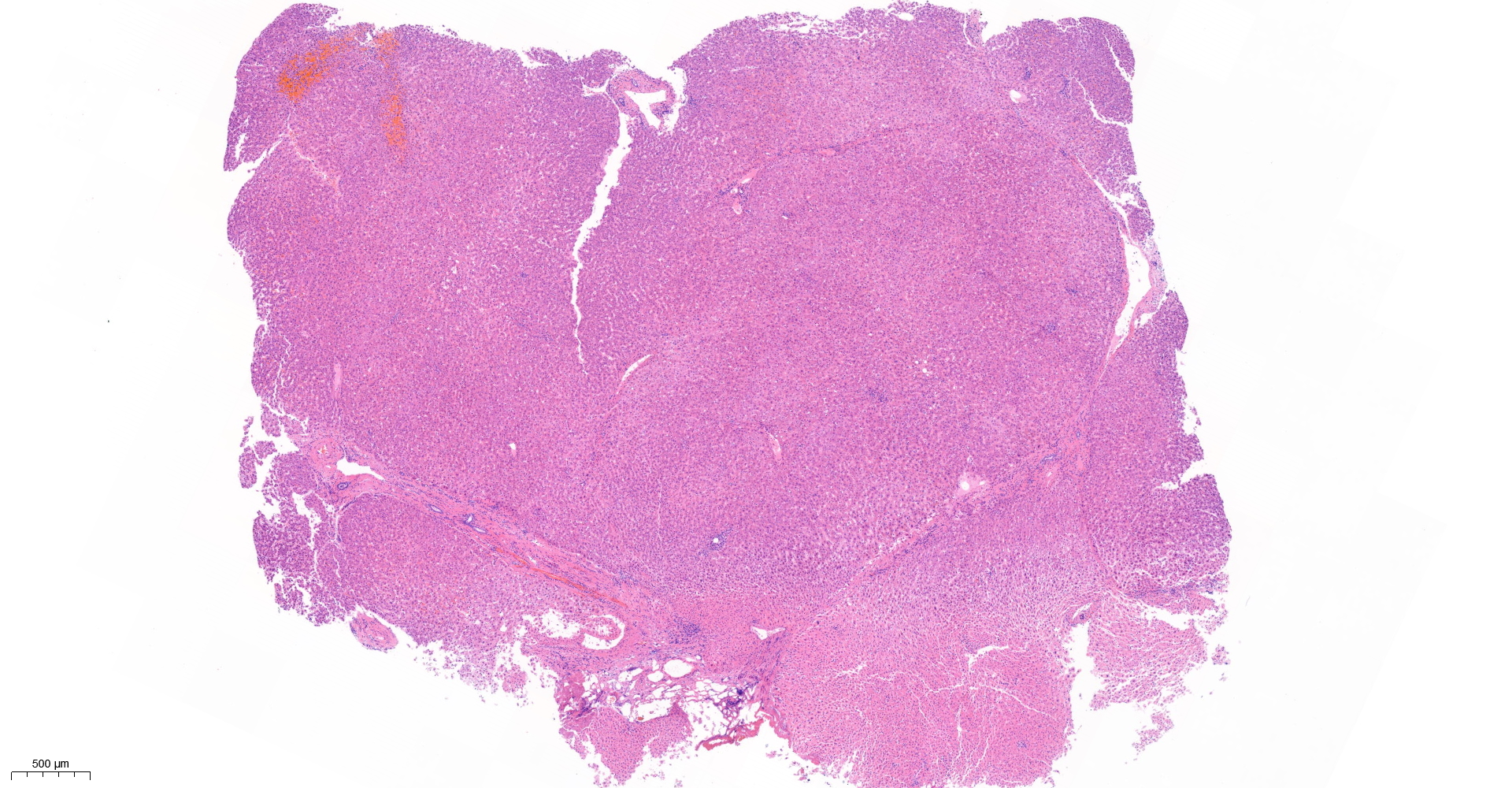


Sample 13.


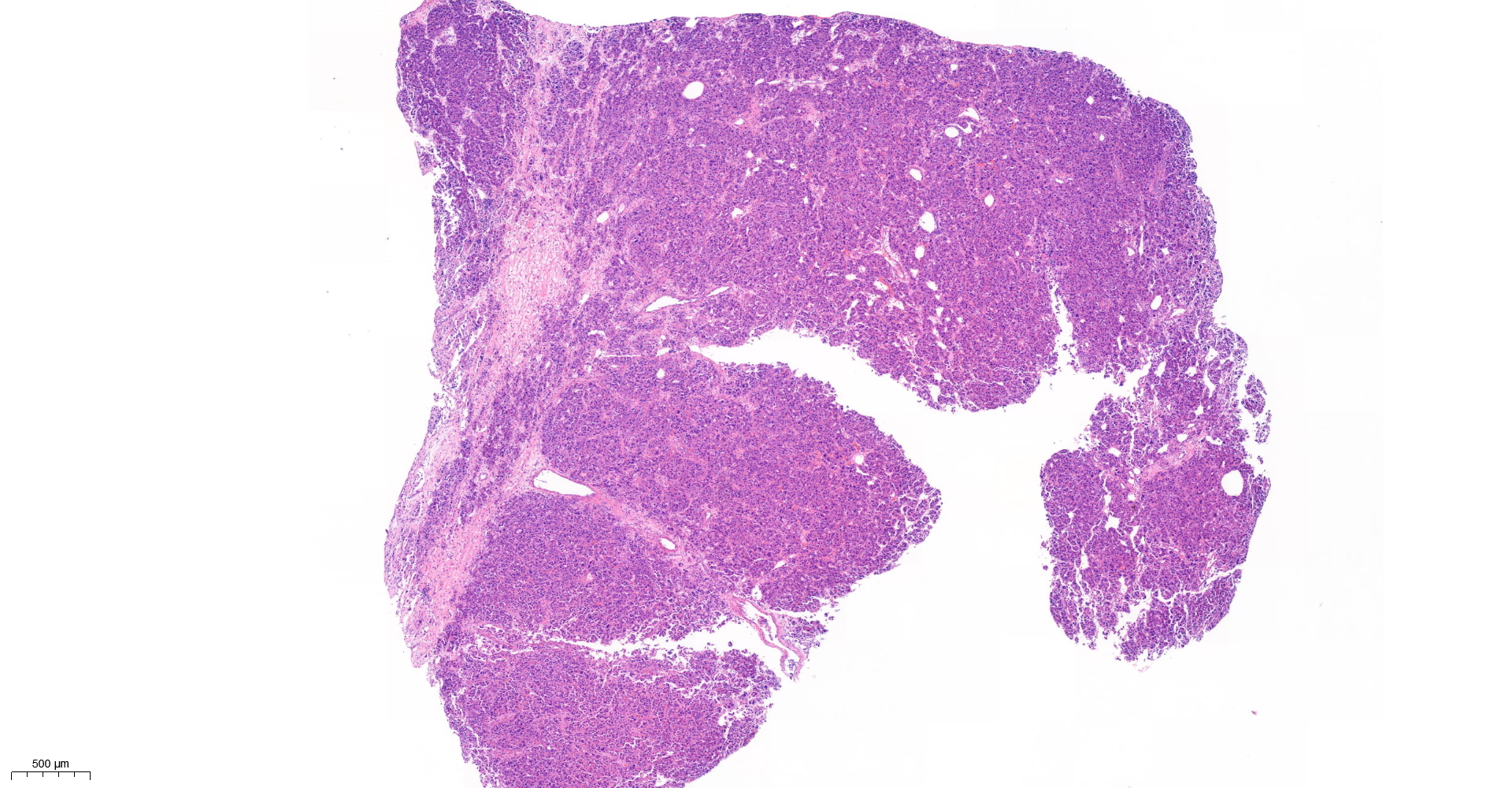


Sample 14.


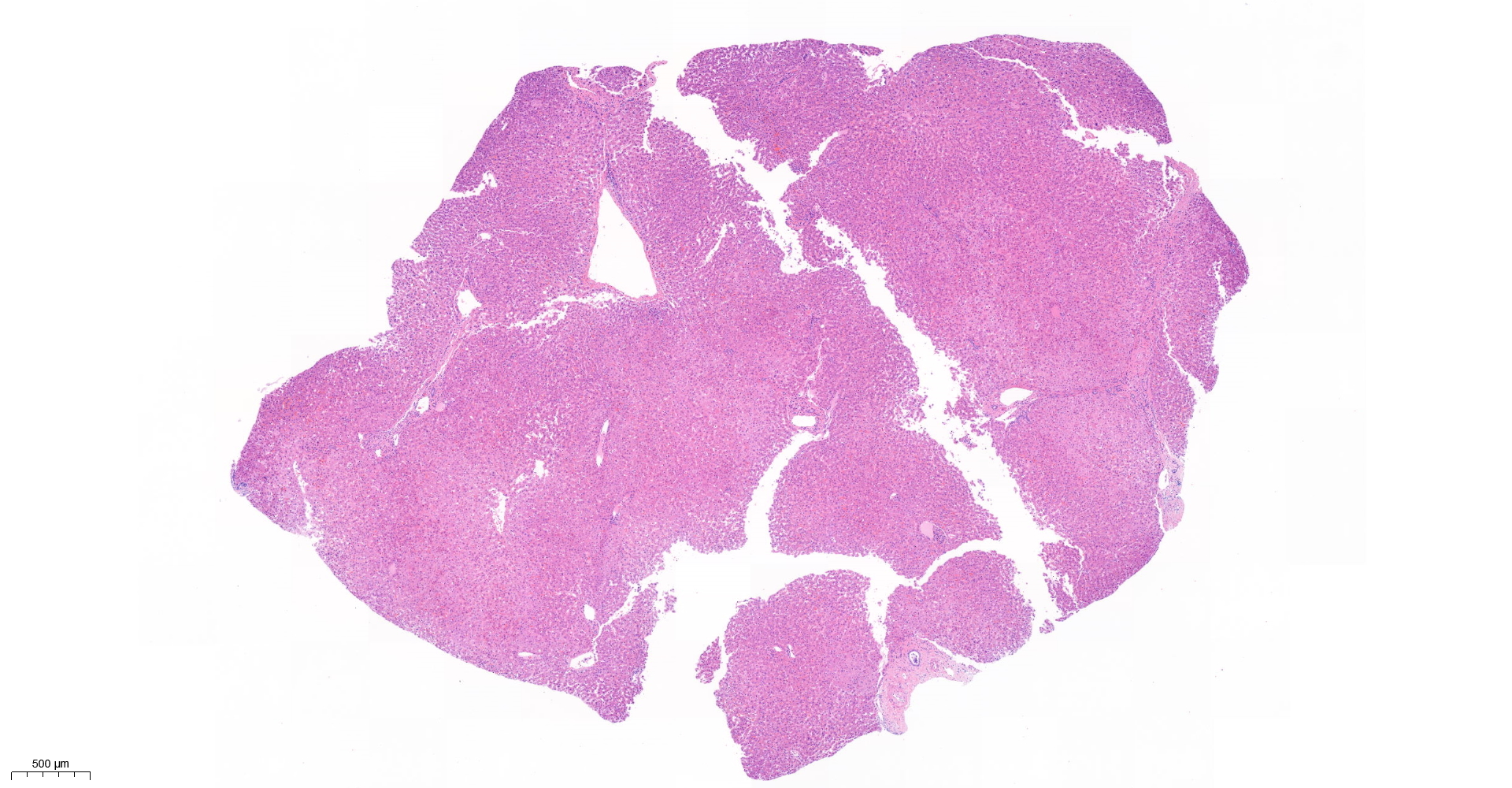


Sample 15.


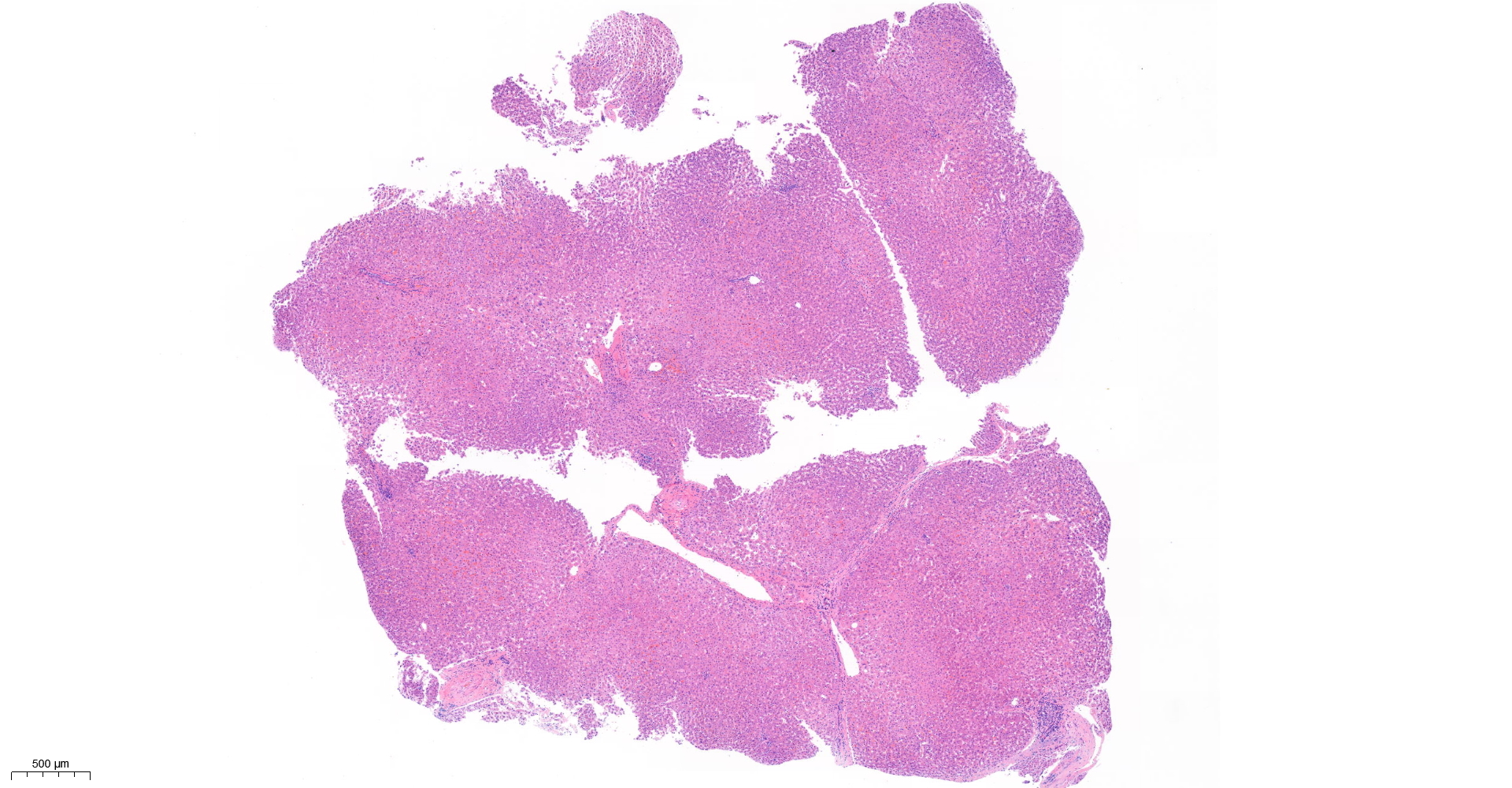


Sample 16.


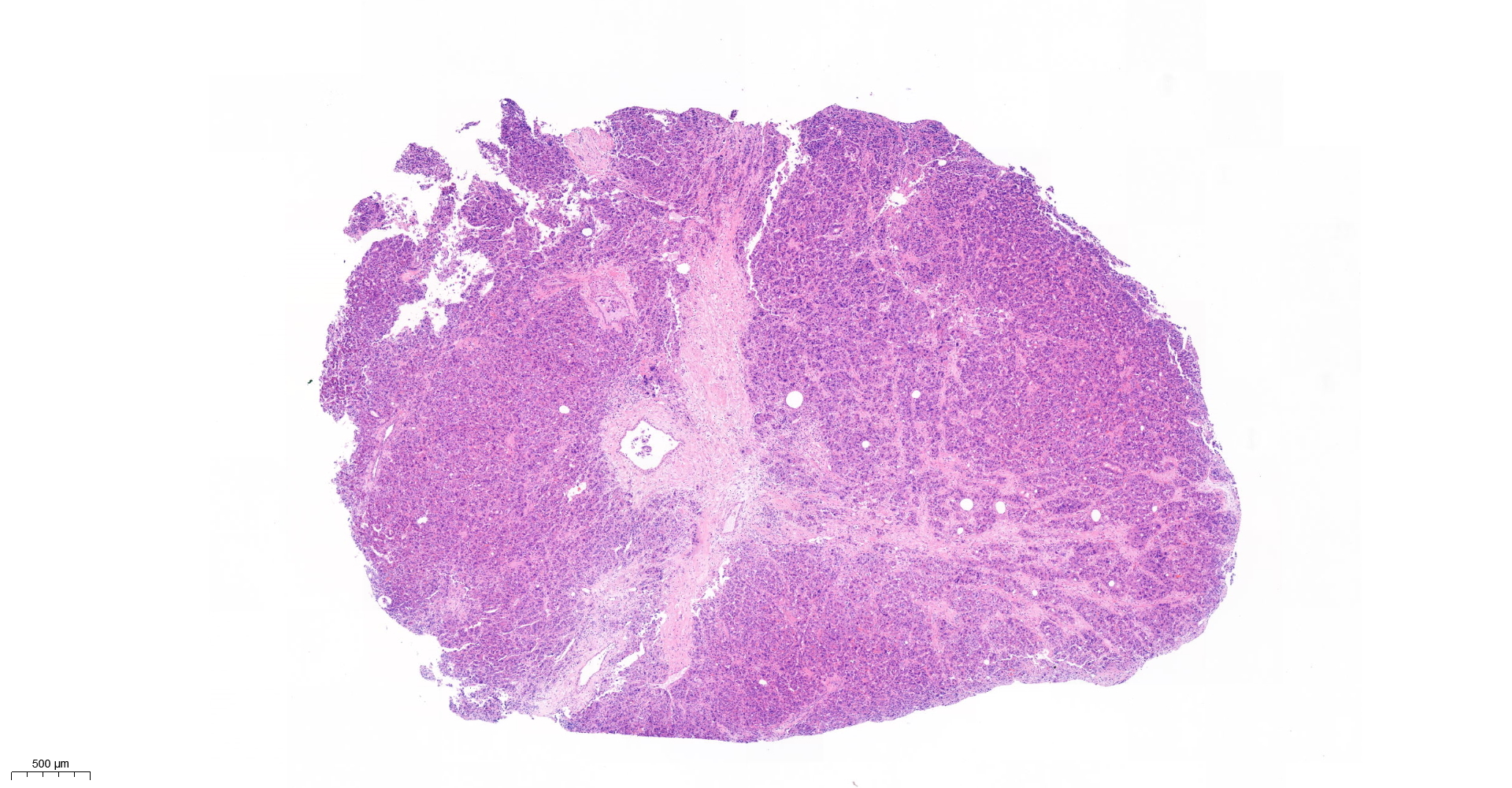


Sample 17.


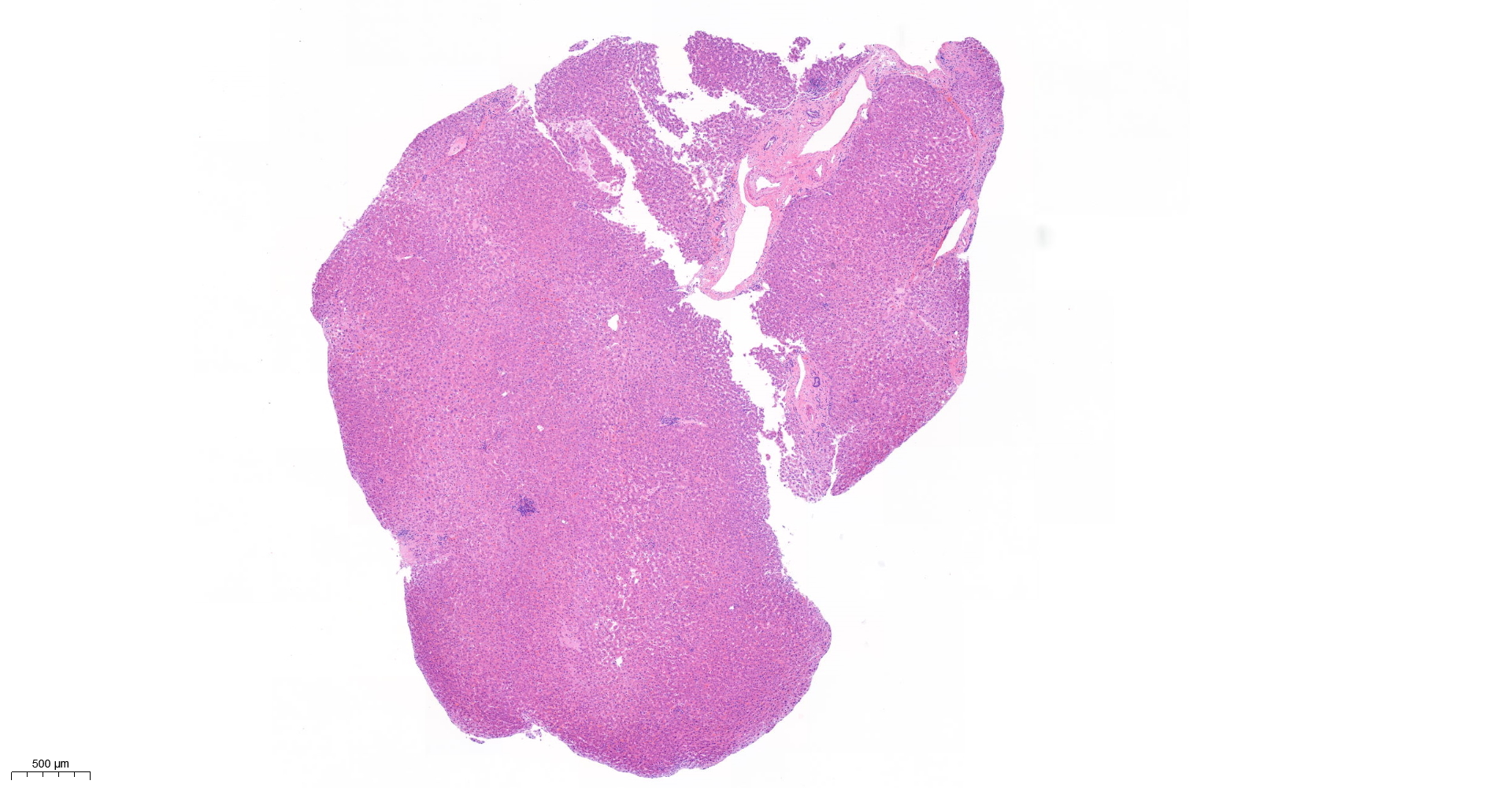


Sample 18.


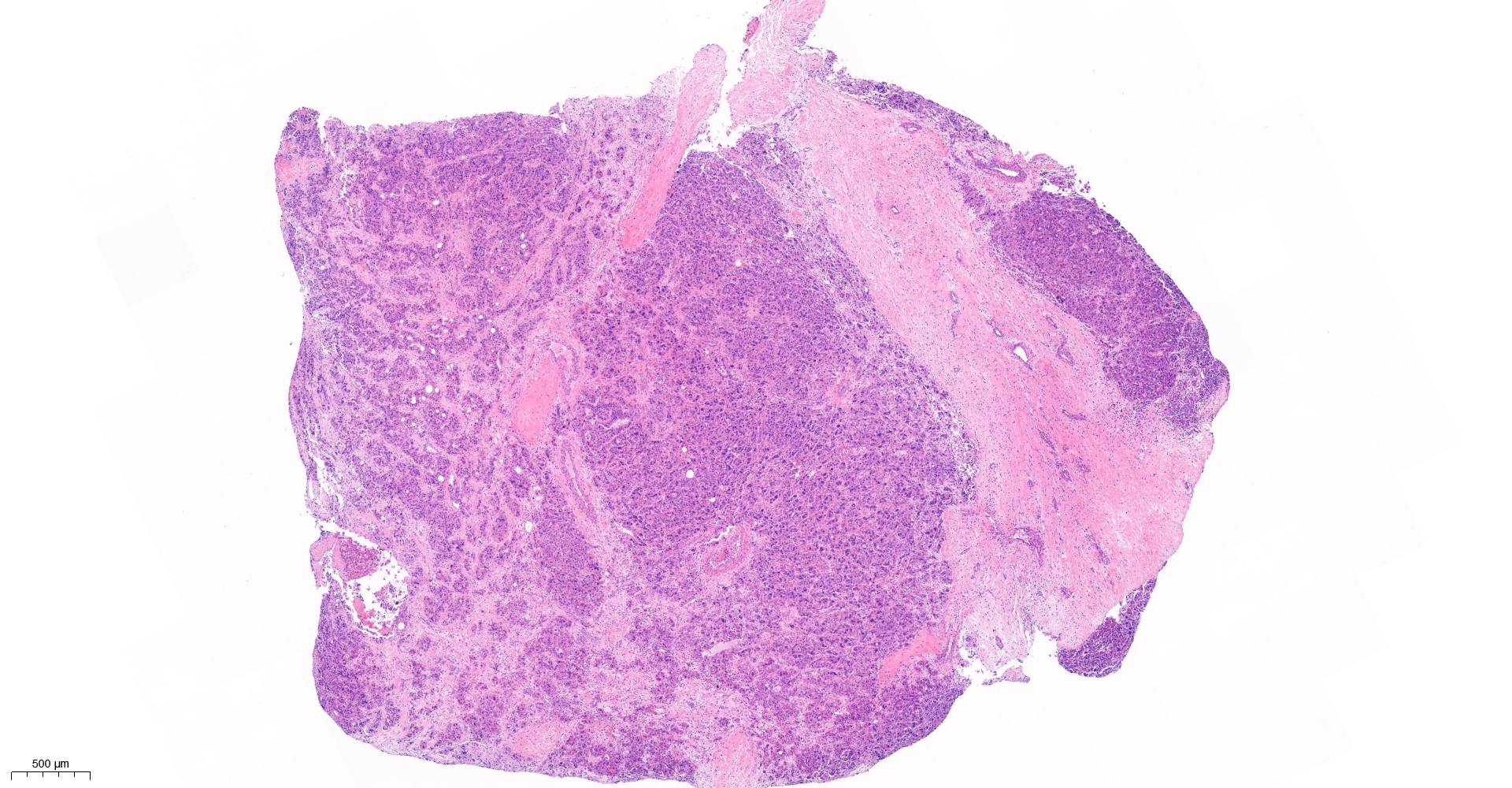


Sample 19.


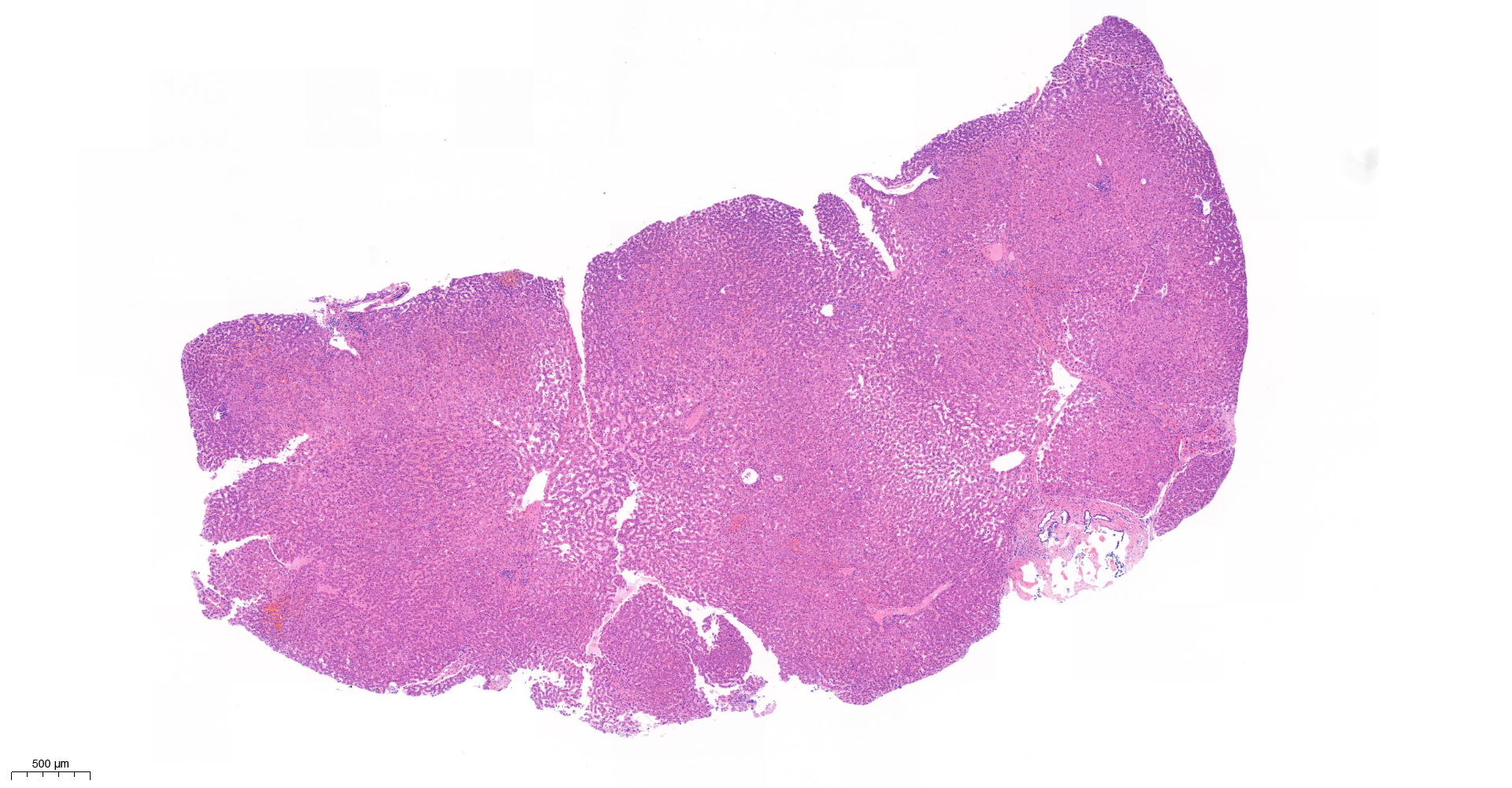


Sample 20.


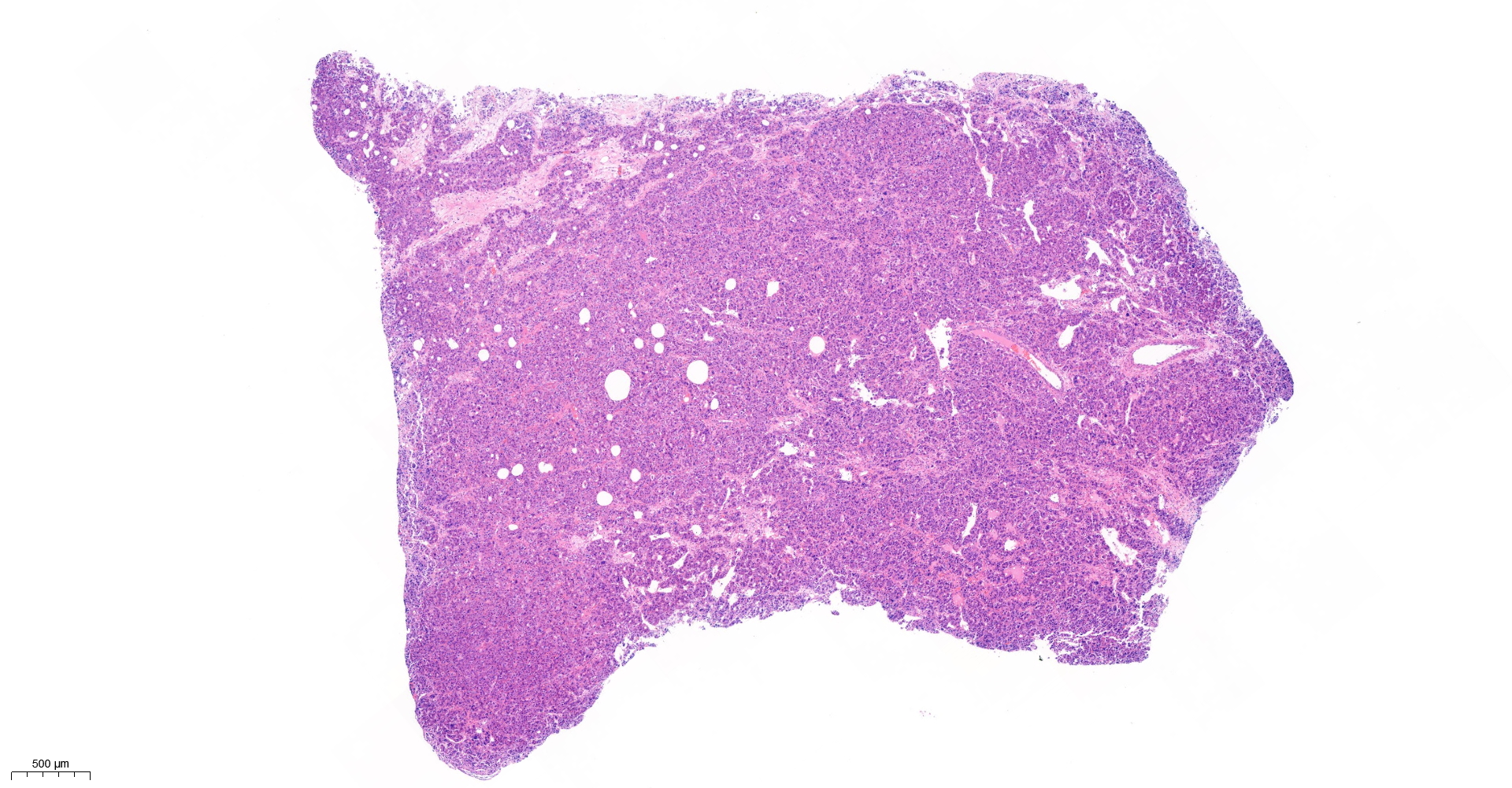


Sample 21.


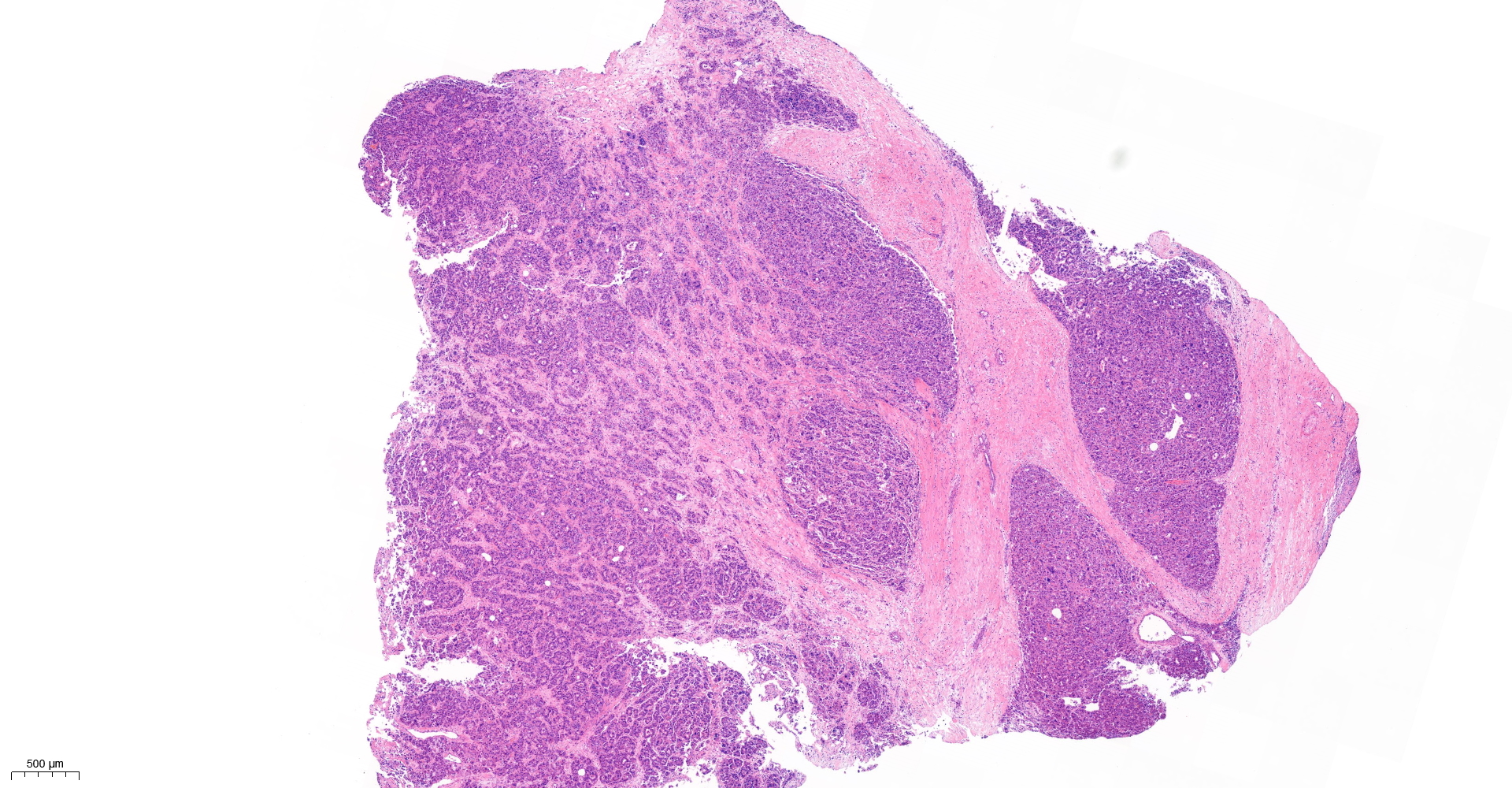


Sample 22.


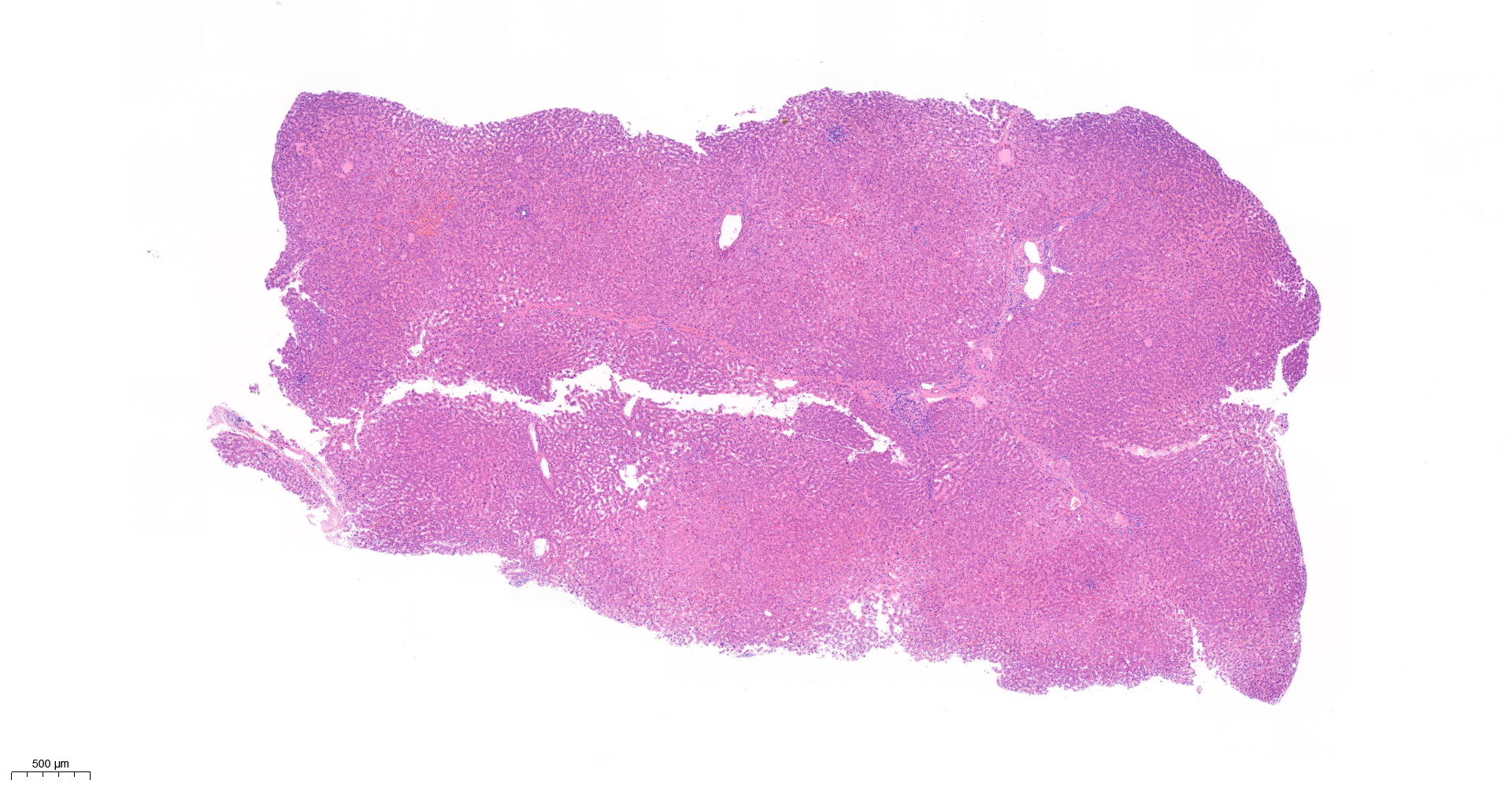


Sample 23.


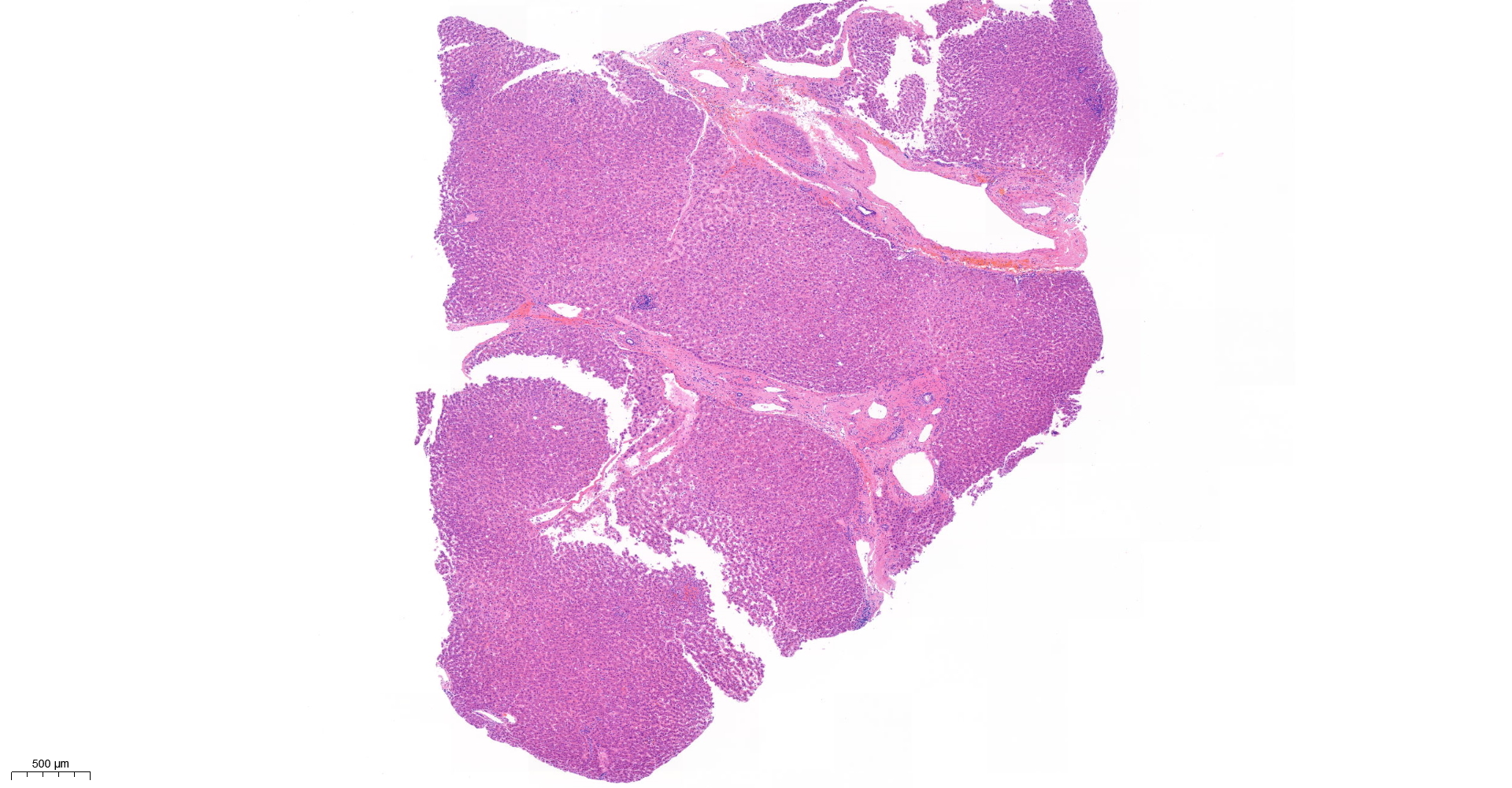


Sample 24.


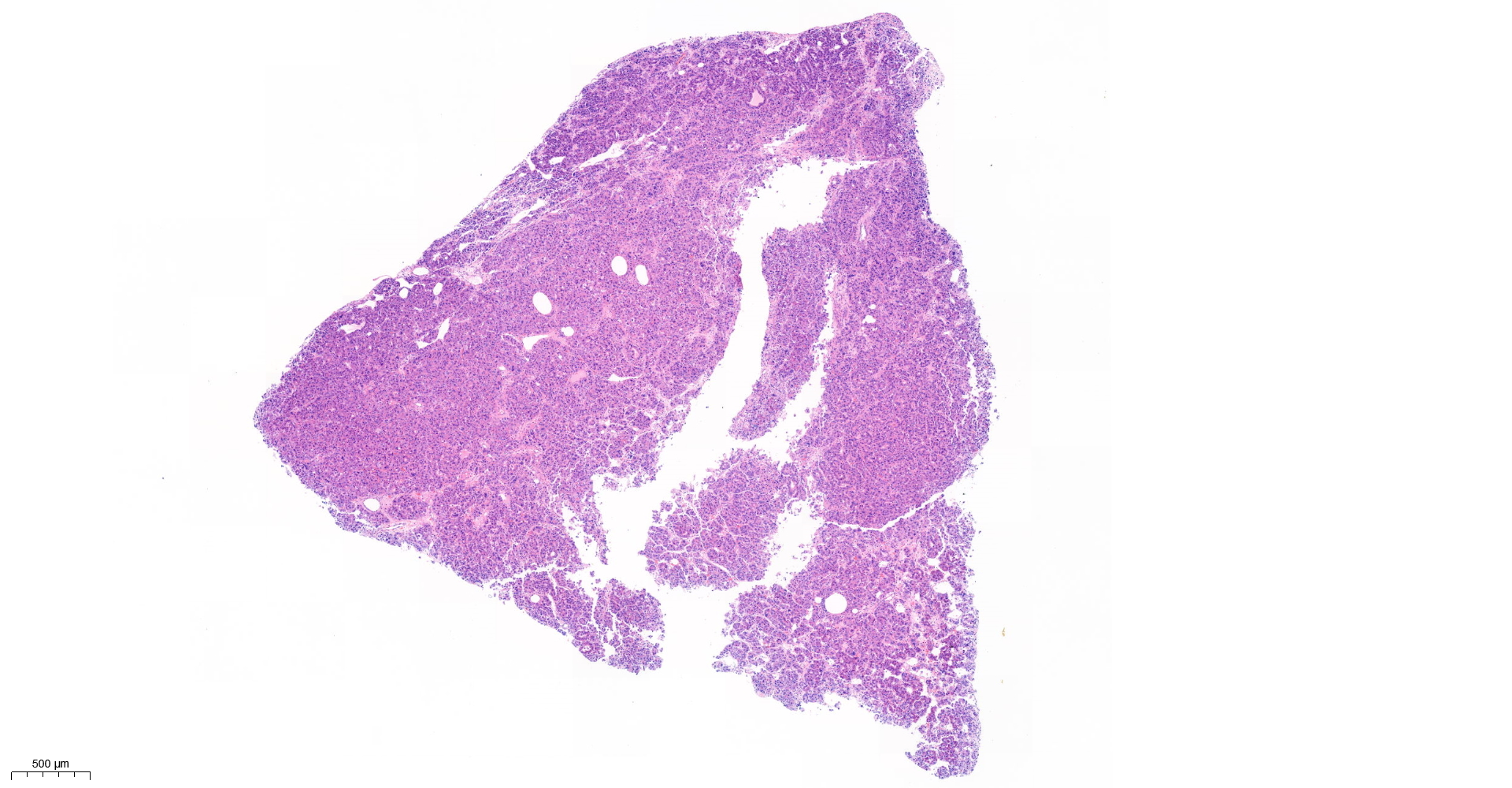


Sample 25.


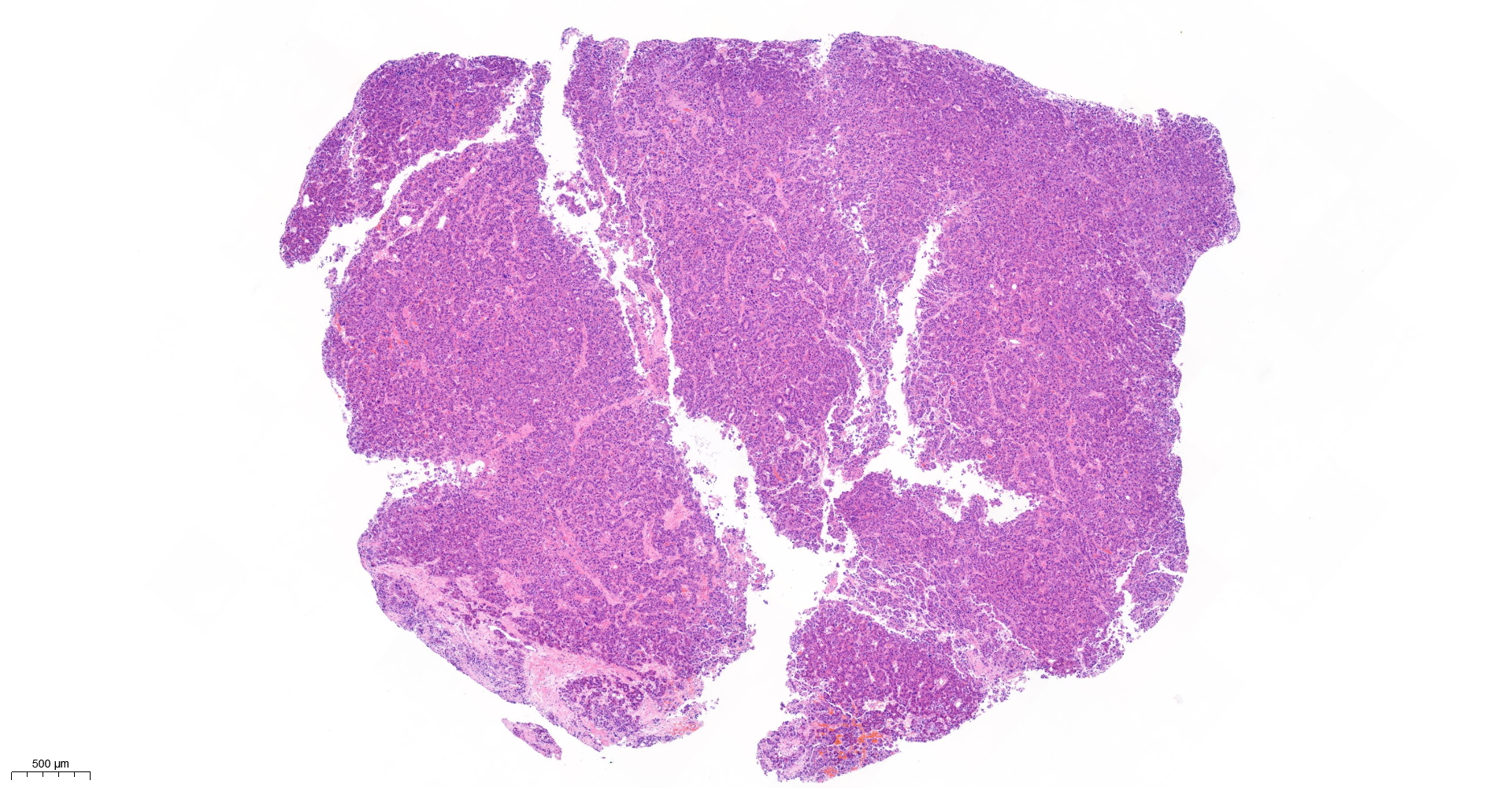


Sample 26.


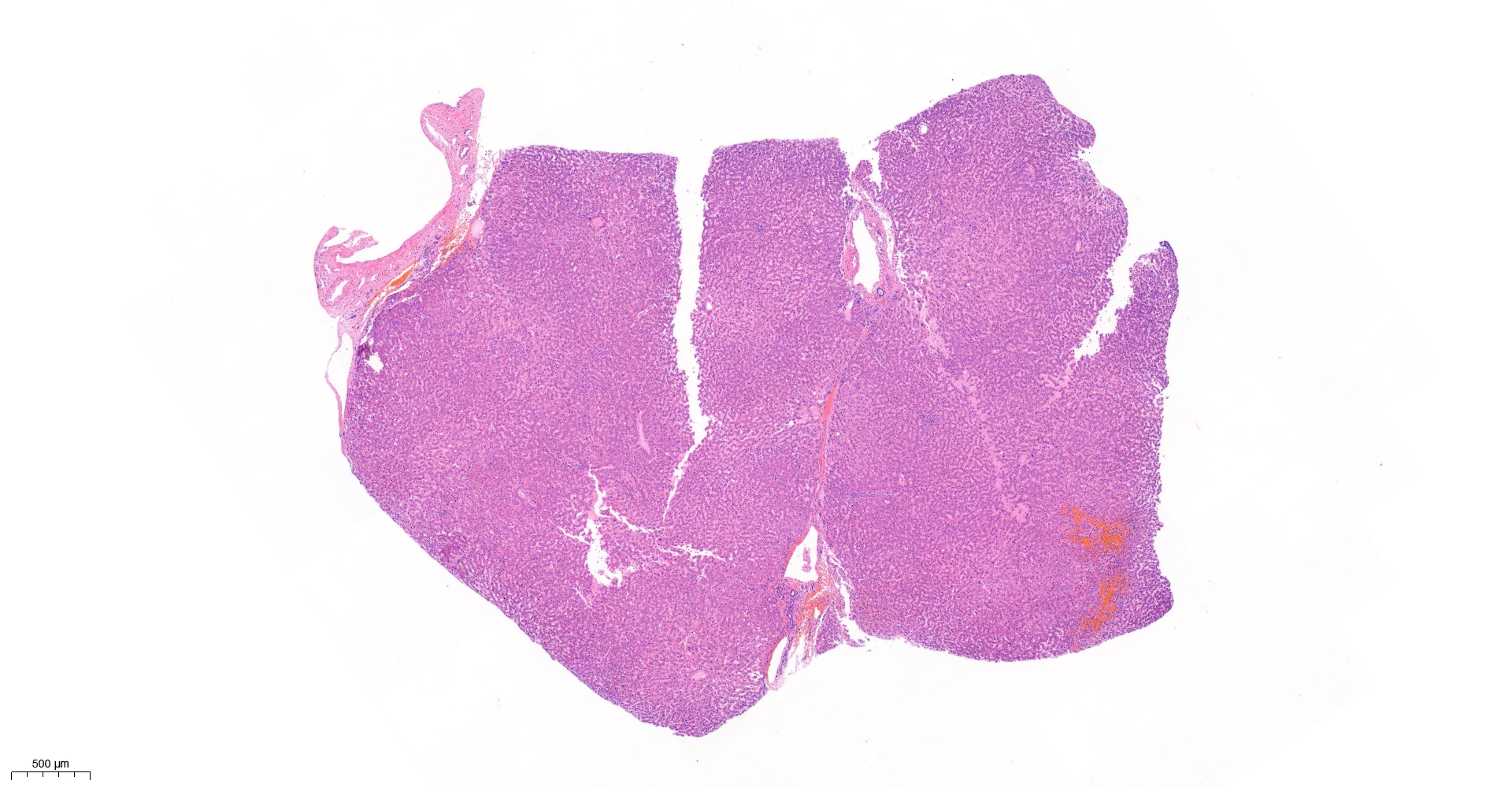


Sample 27.


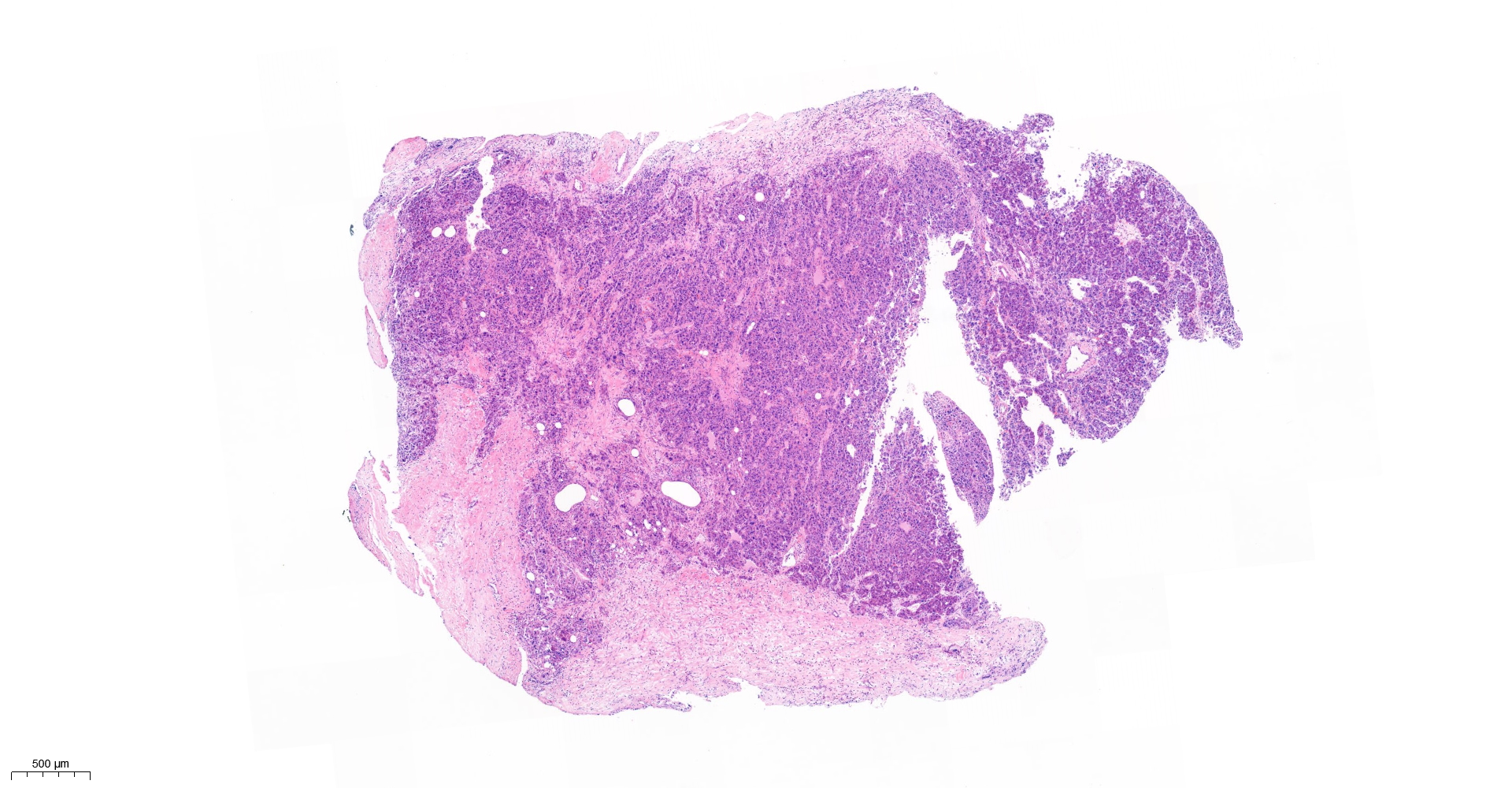


Sample 28.


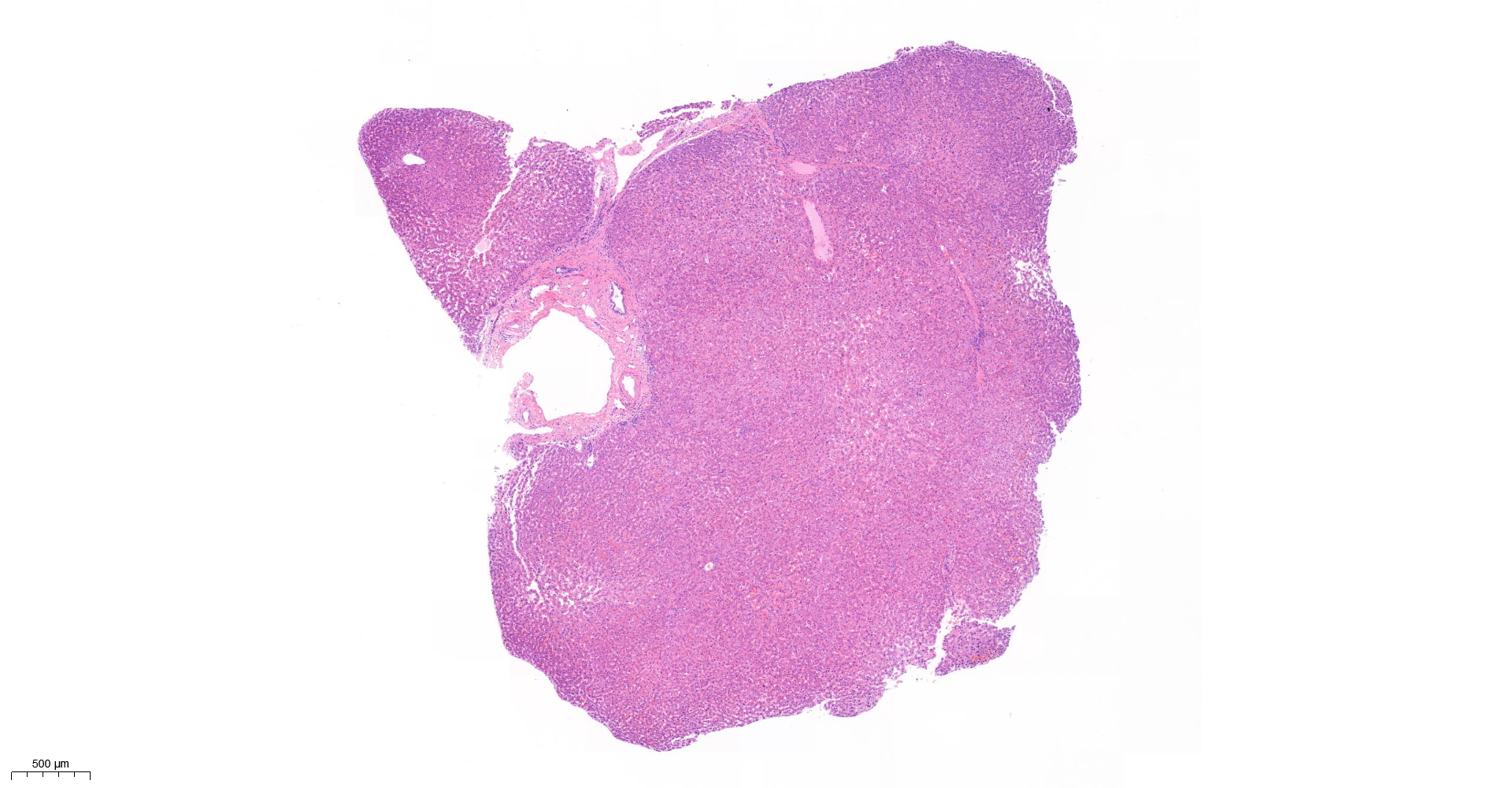


Sample 29.


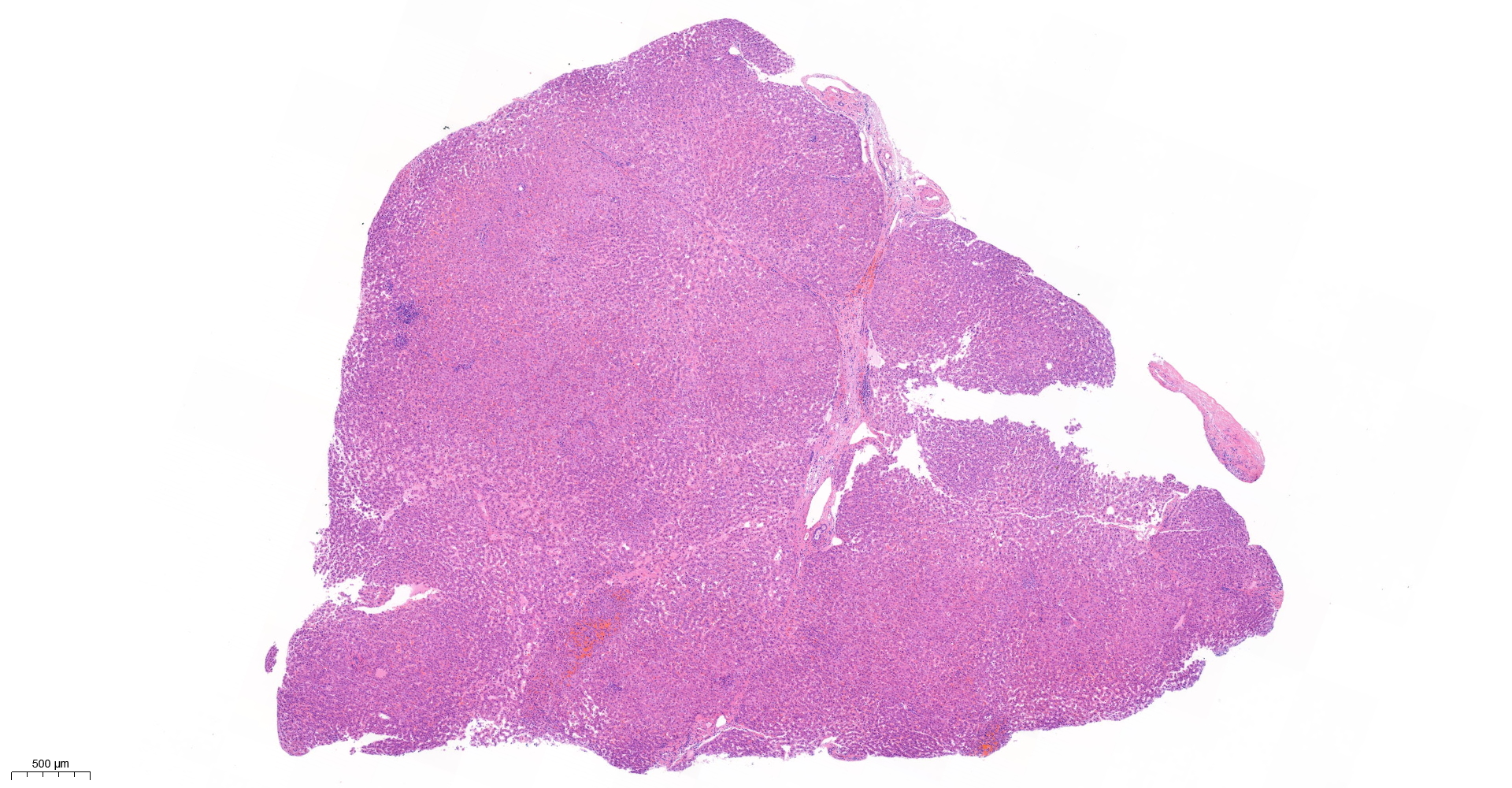


Sample 30.


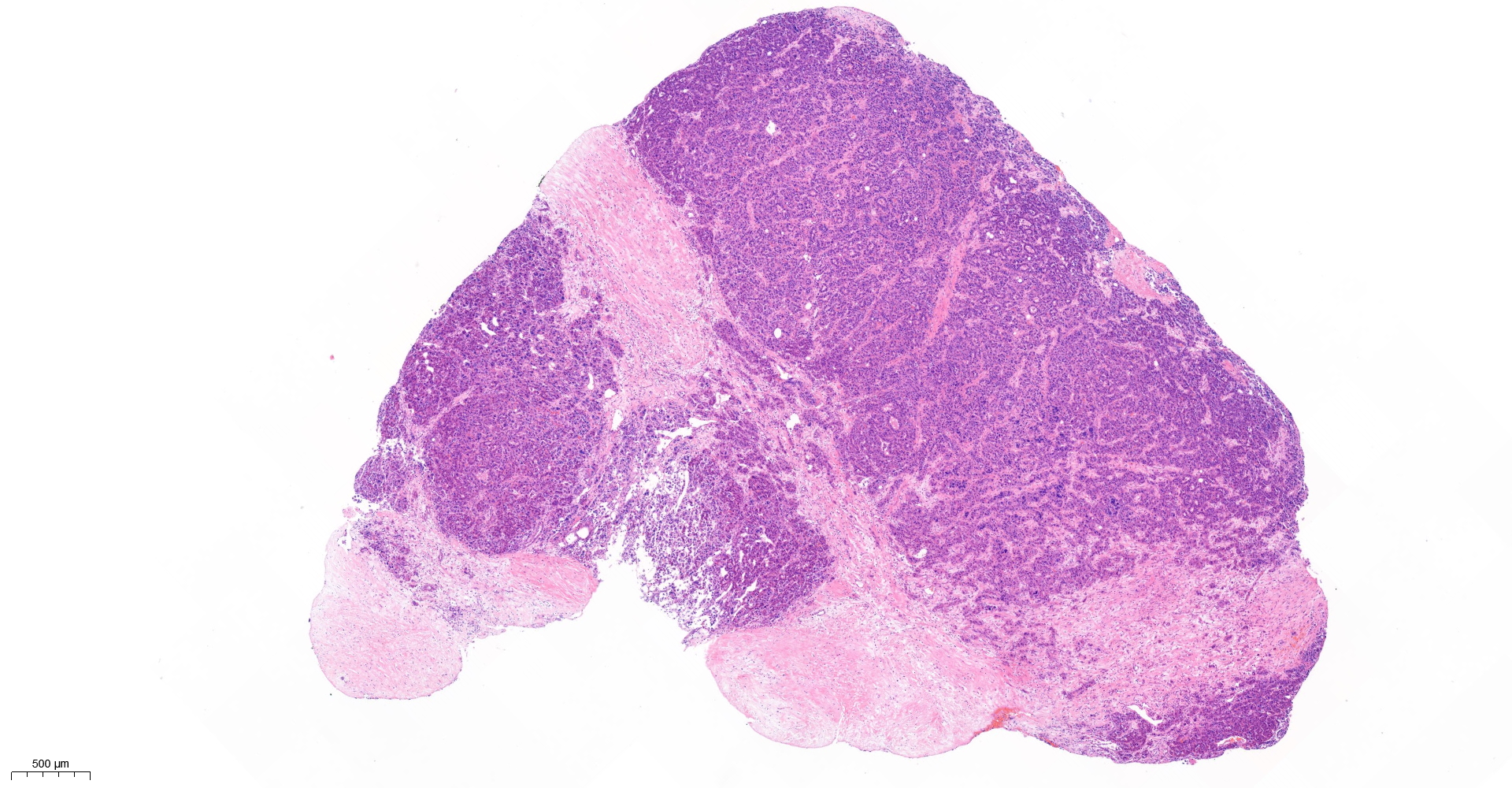


Sample 31.


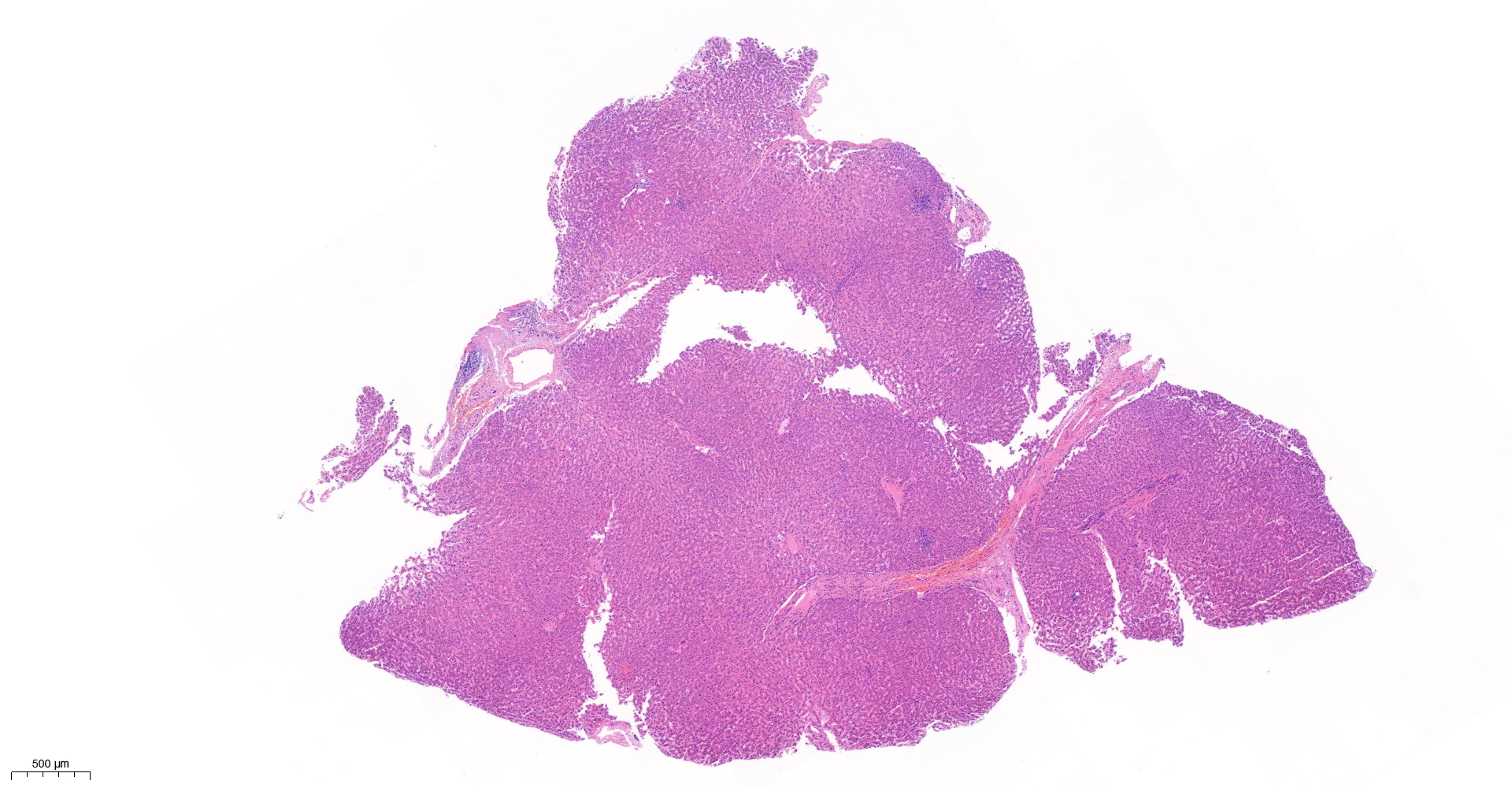


Sample 32.


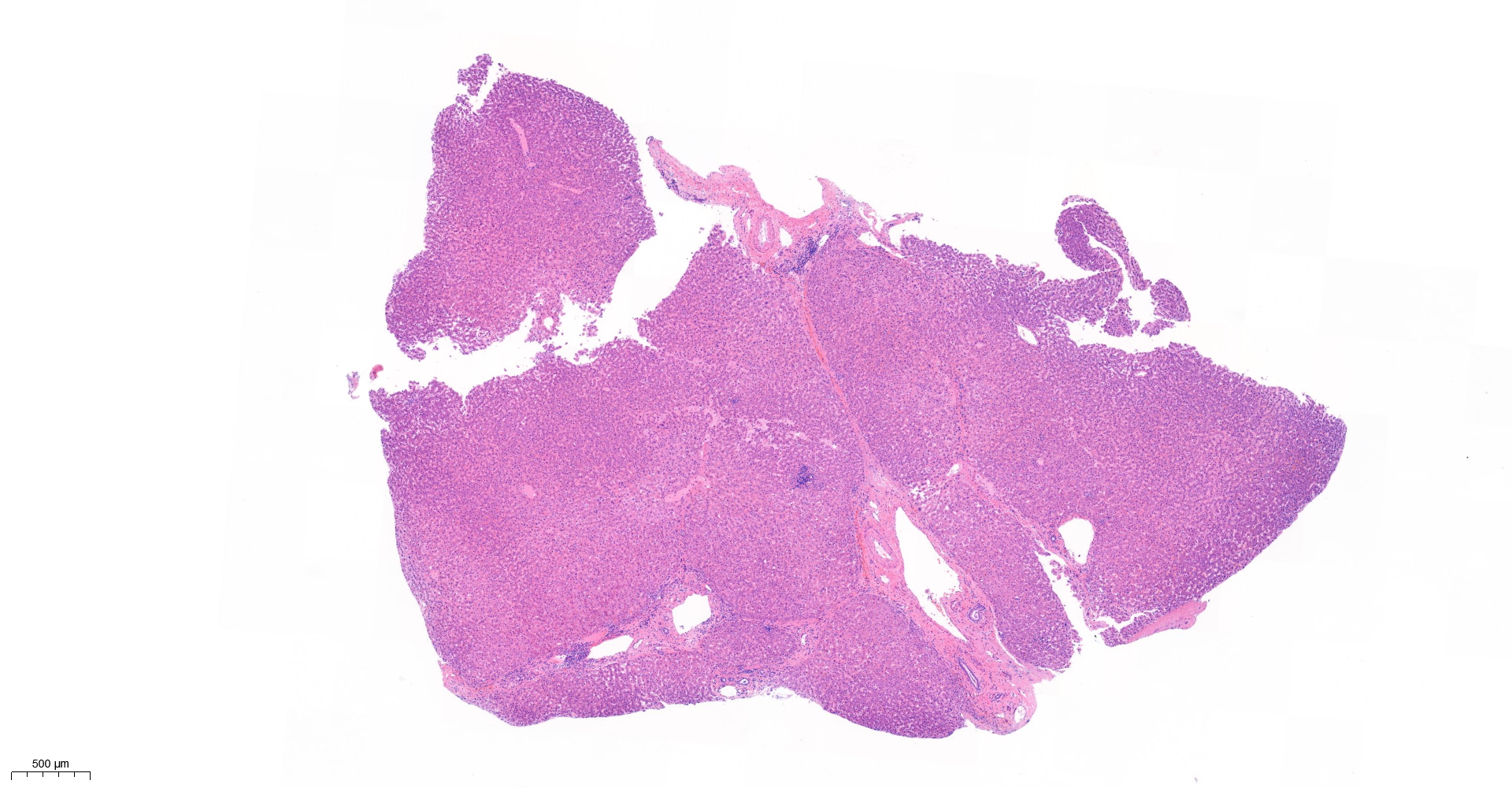


Sample 33.


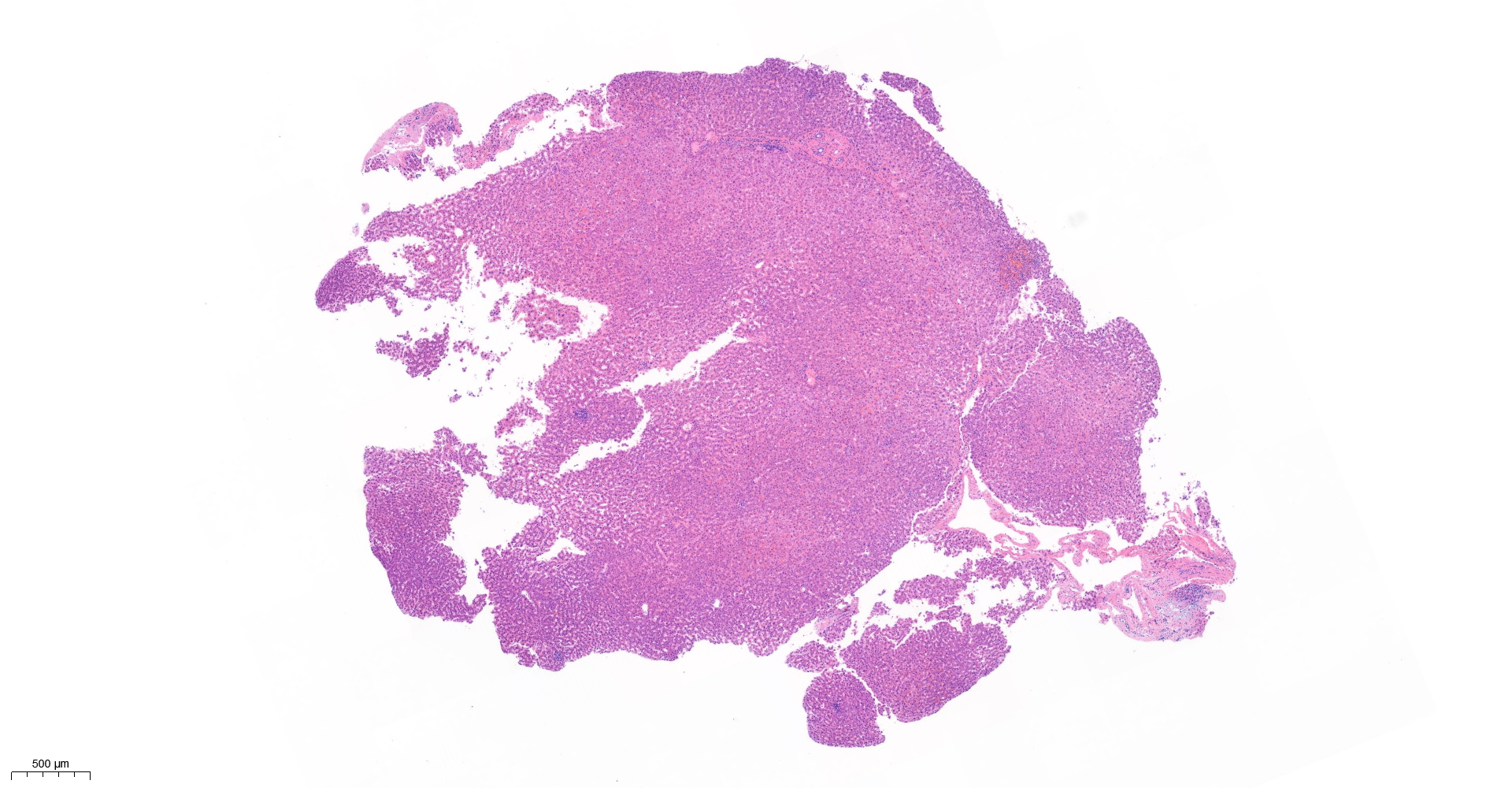


Sample 34.


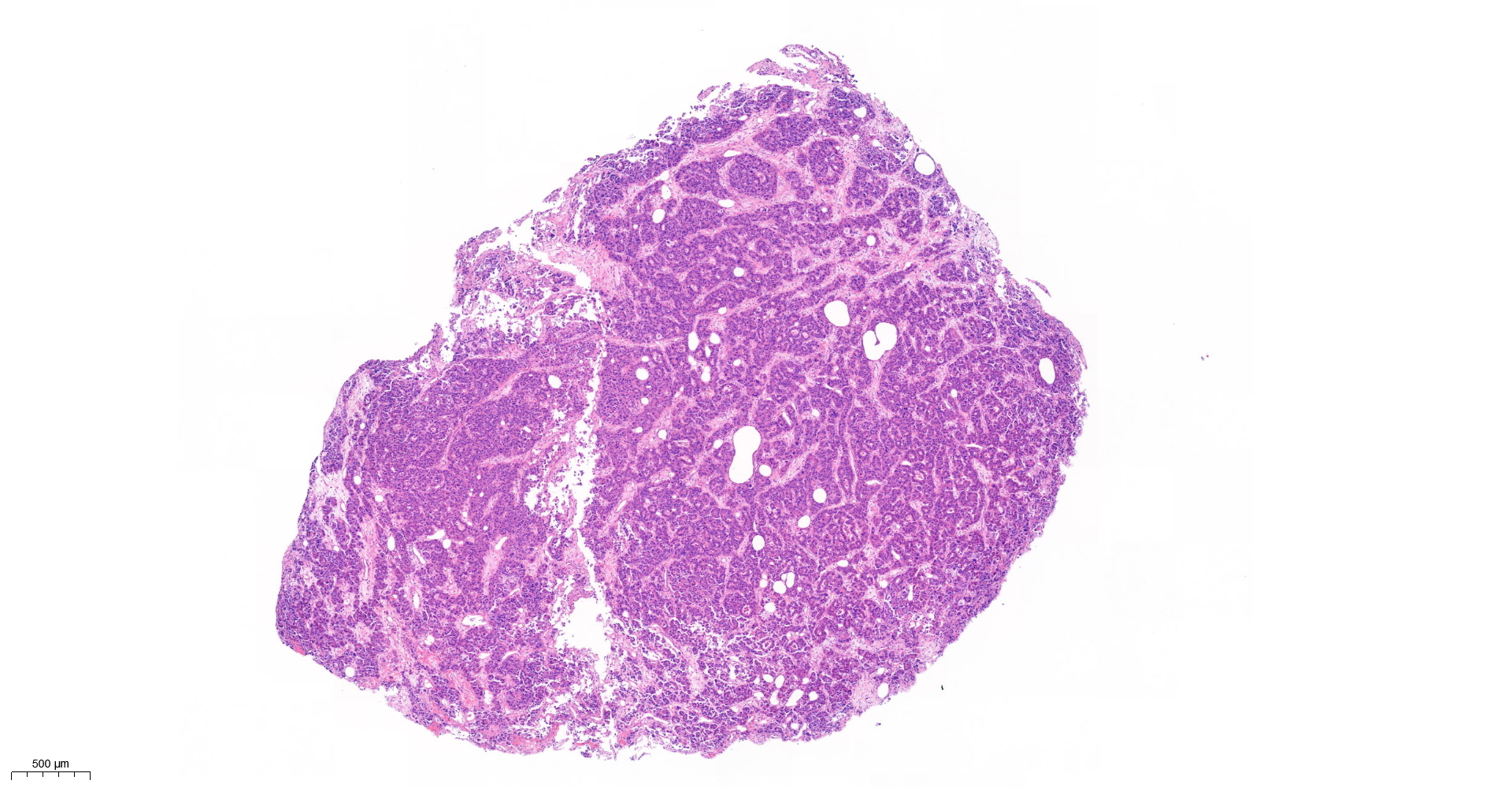


Sample 35.


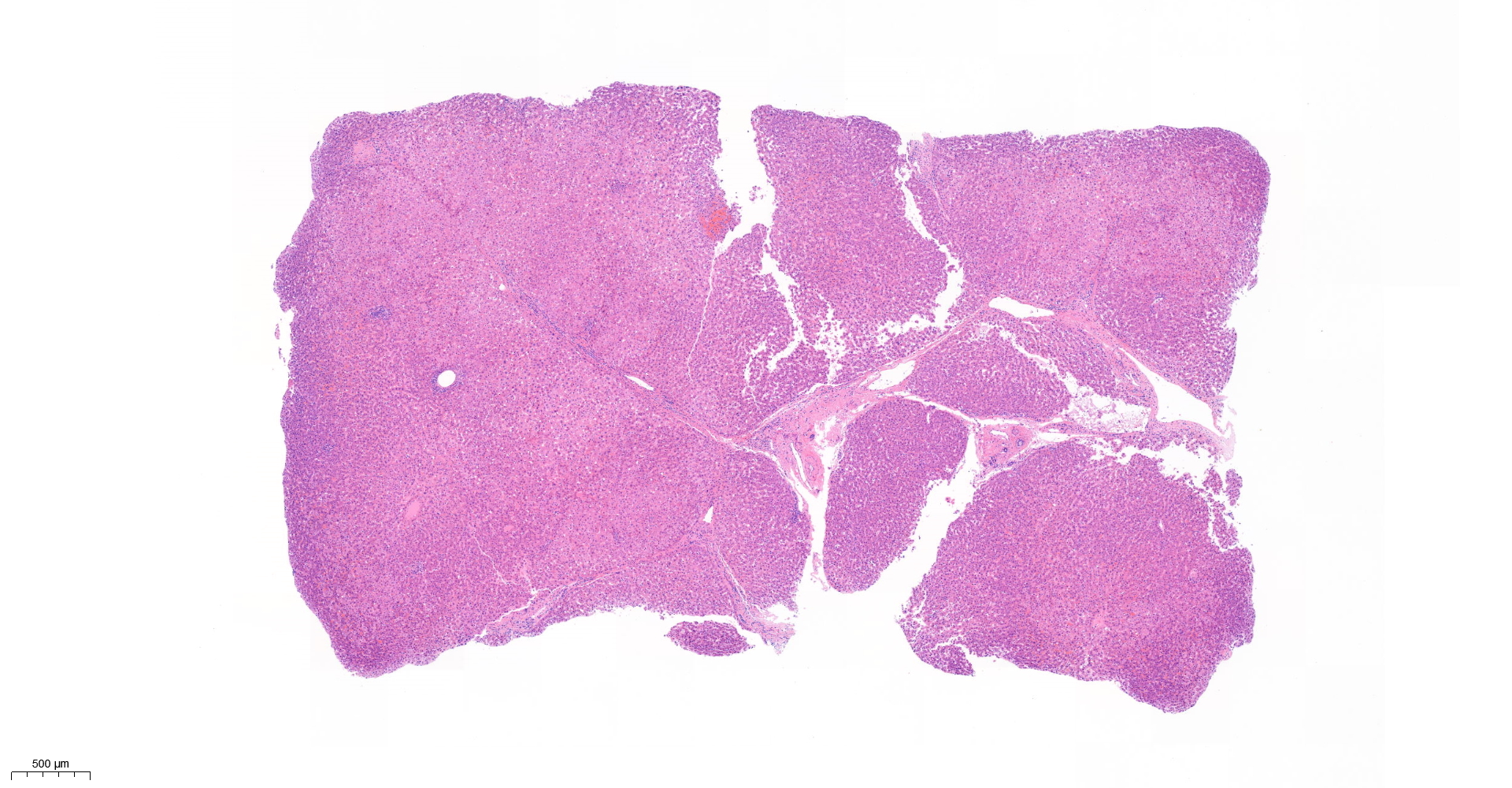

Supplement: Figure 6—source data 1. [file elife-70471-fig6-data1.zip › Figure 6-Source data/Raw data and Thumbnail images of HE staining images in Figure 8.docx]
