# Supplementary material for: A pre-screening strategy to assess resected tumor margins by imaging cytoplasmic viscosity and hypoxia
Source: eLife. 2021 Oct 11;10:e70471. doi: 10.7554/eLife.70471 (PMC8553343; doi:10.7554/eLife.70471)

Raw data of the hematoxylin and eosin scanning images (12G, 35 files) are available through the following links.

Microsoft one drive:

<https://emailcpcc-my.sharepoint.com/:u:/g/personal/mjohn123_email_cpcc_edu/ESwiAZiXnvNJphXaiJtFPAcBqKke4eH8qTth7Jye5rhBwg?e=HL8HMO> Password:8545

Or personal cloud drive:

<http://IBSlab.quickconnect.cn/d/s/621769054052466634/yk8SxiNkcZrhQWLocXFesuuaGGZhyJ7Z-Ob7gv9T6oAg_> Password:8545

Thumbnail images of all 16 samples are presented below.

Scale bar: 500 μm.

Sample 1.


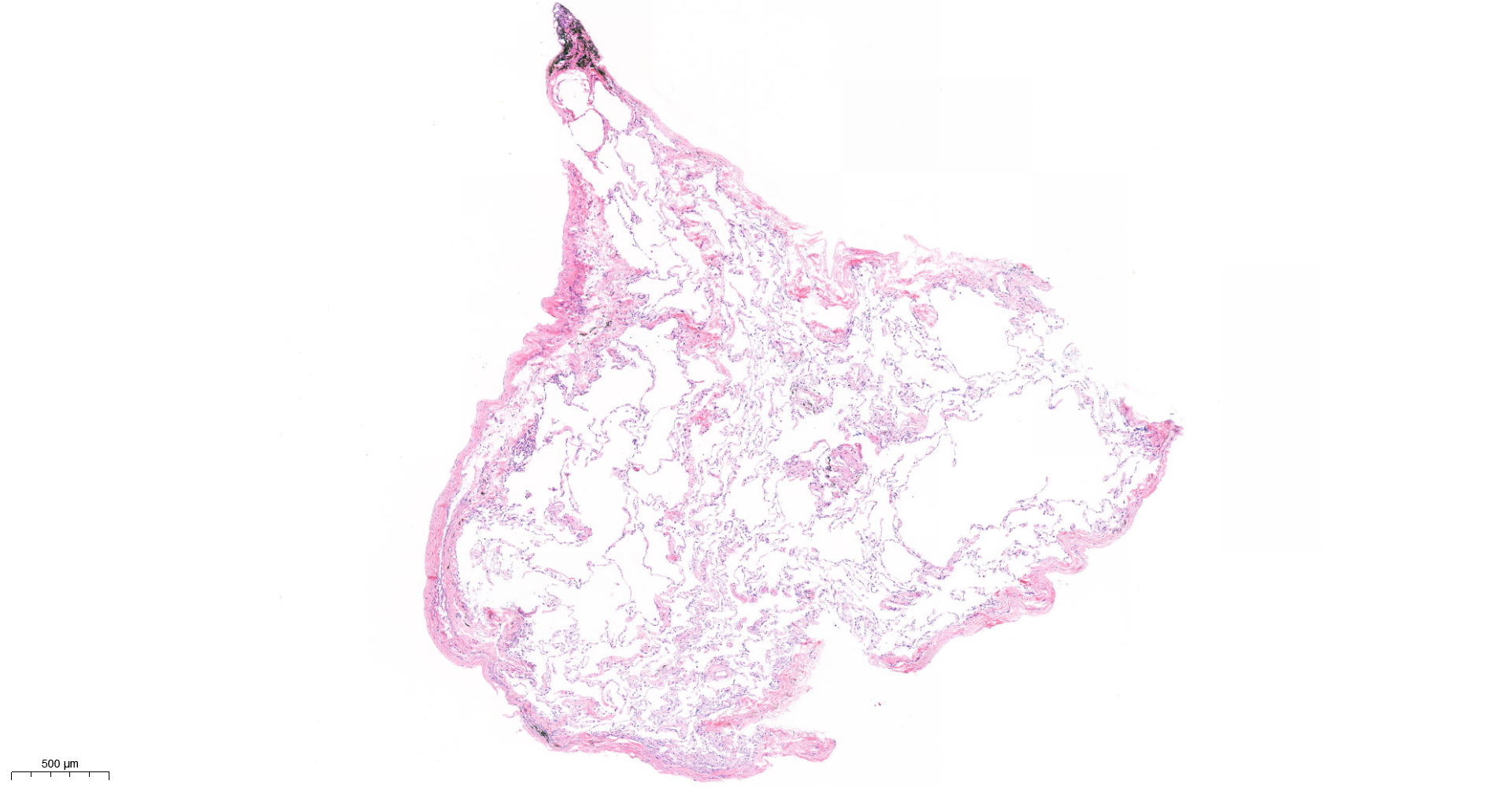


Sample 2.


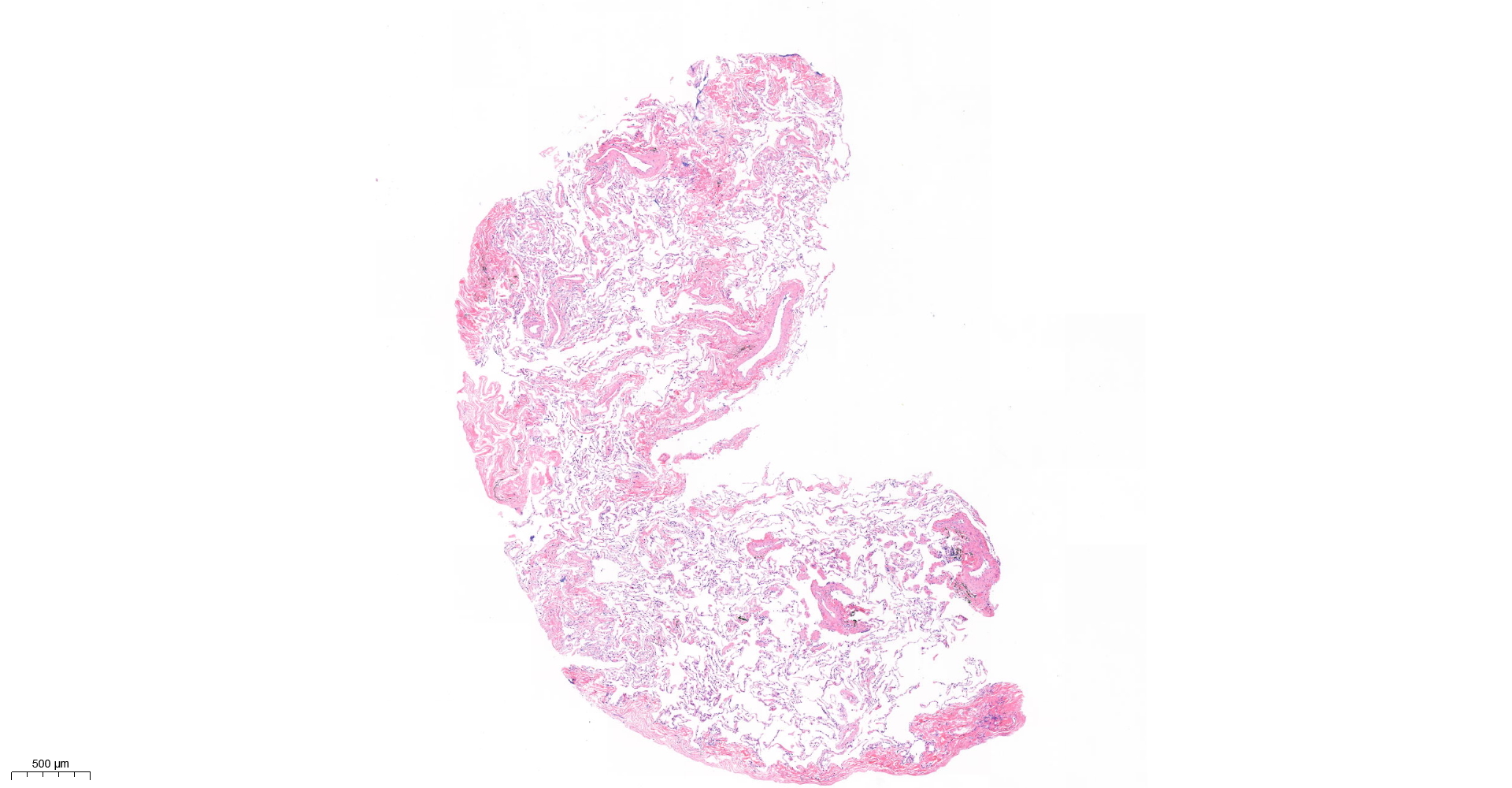


Sample 3.


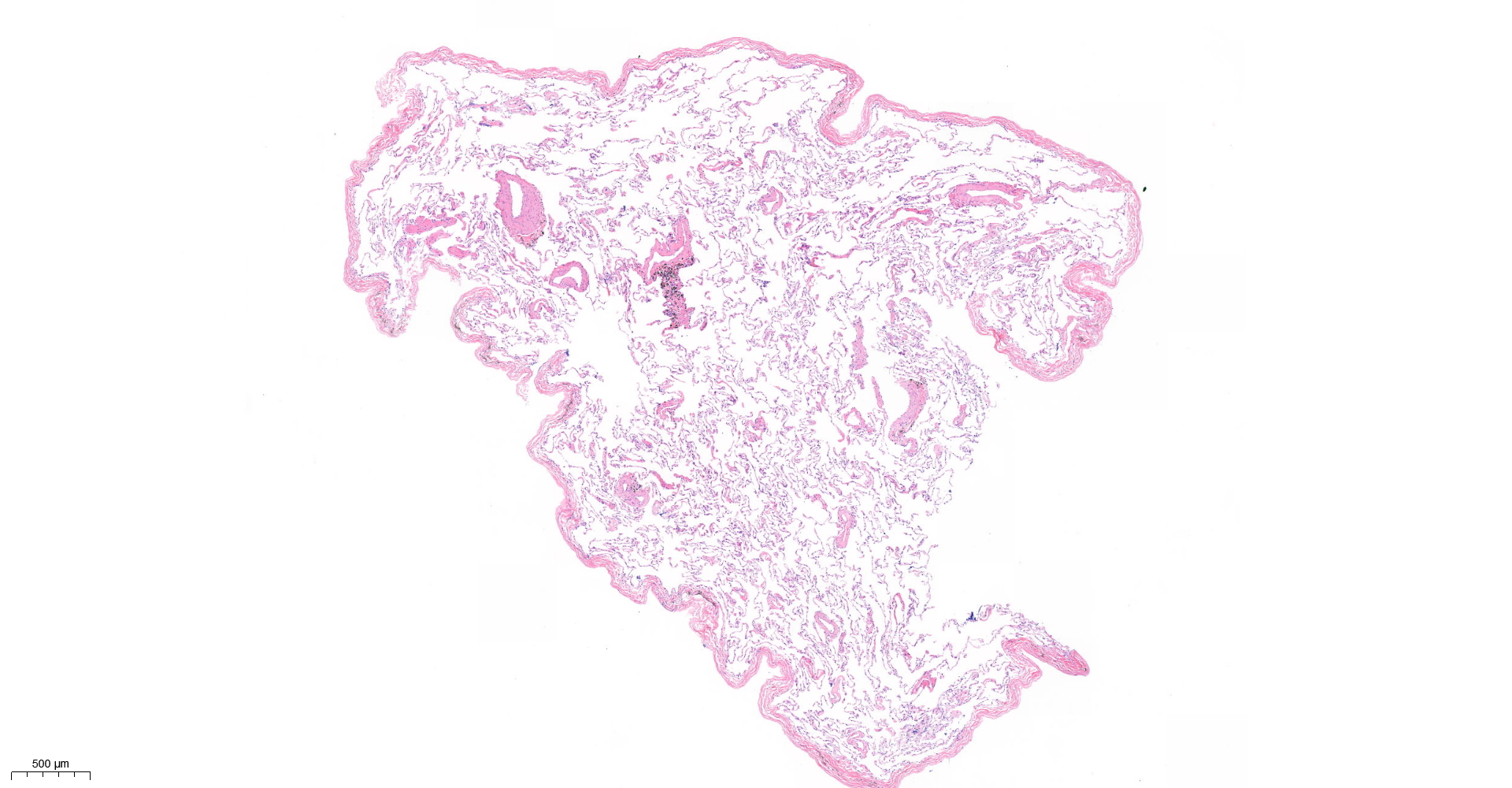


Sample 4.


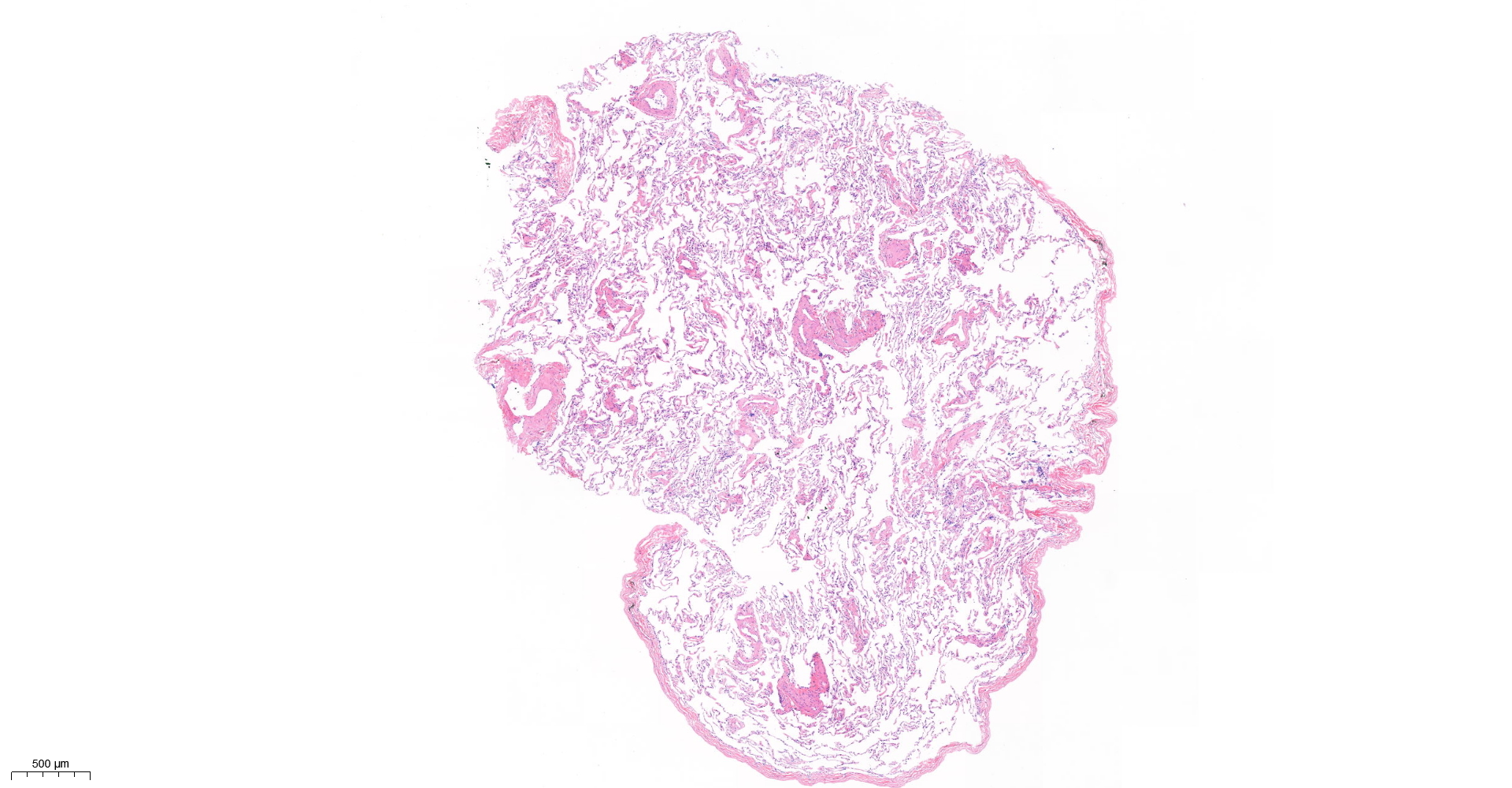


Sample 5.


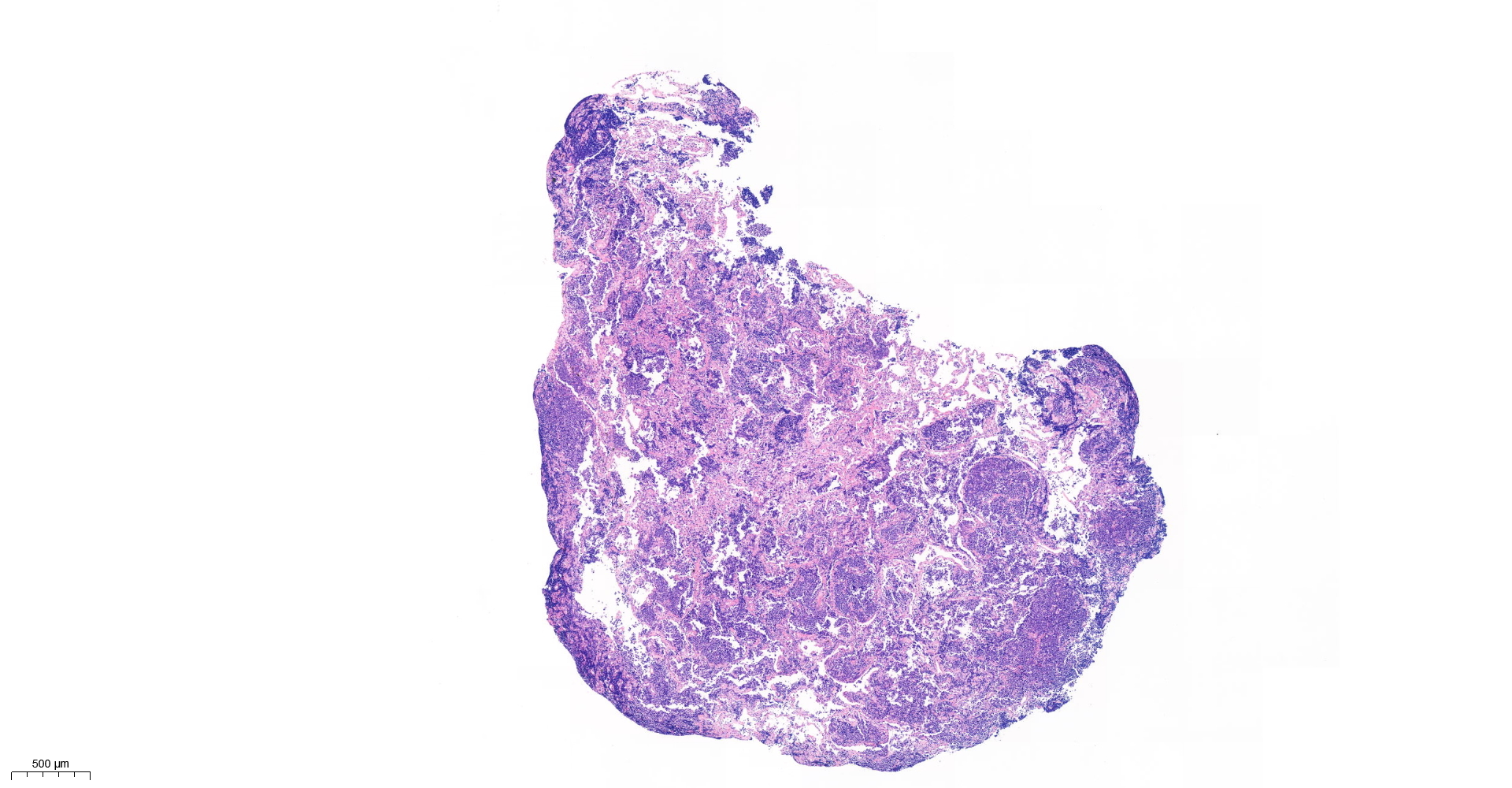


Sample 6.


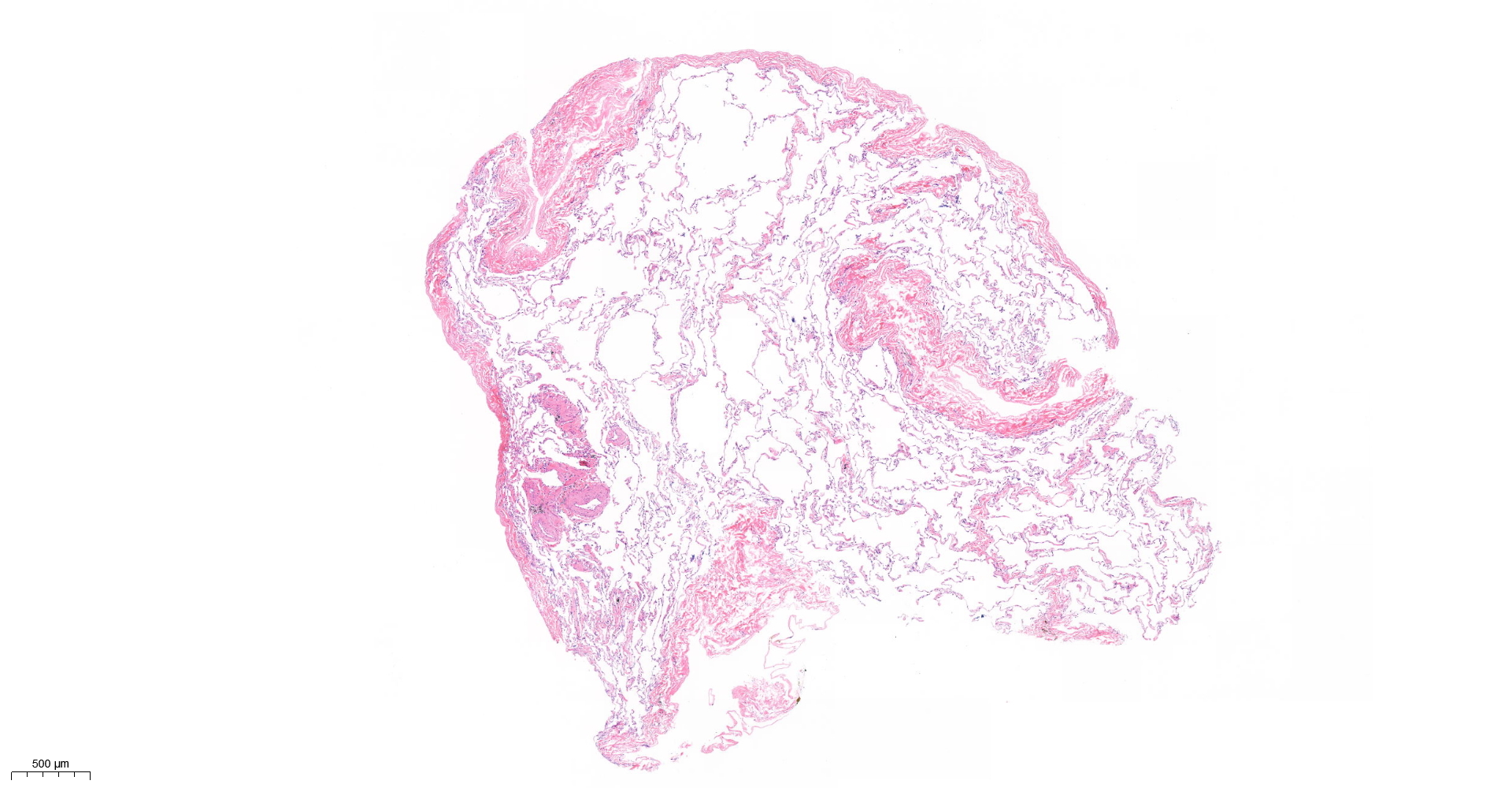


Sample 7.


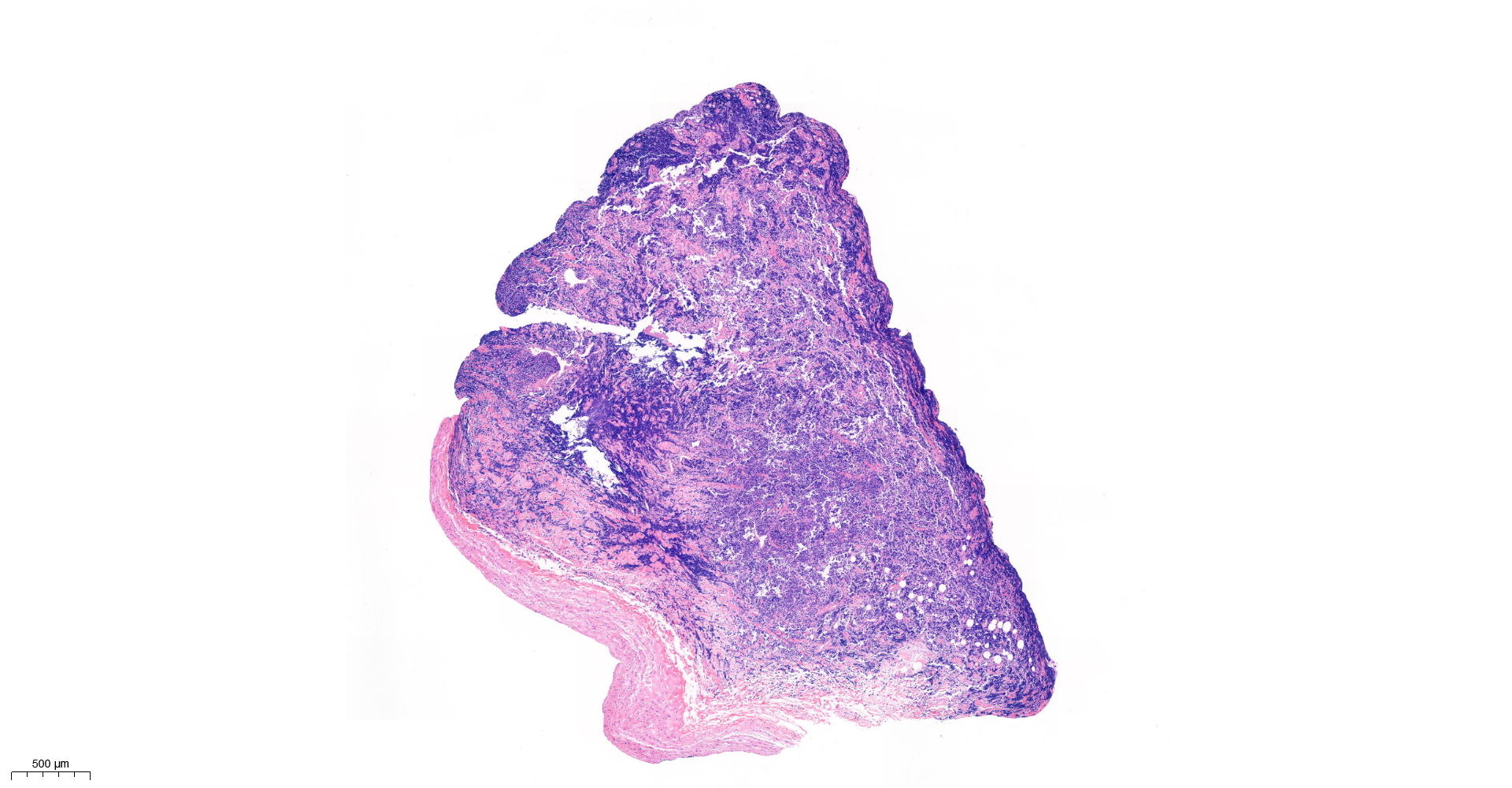


Sample 8.


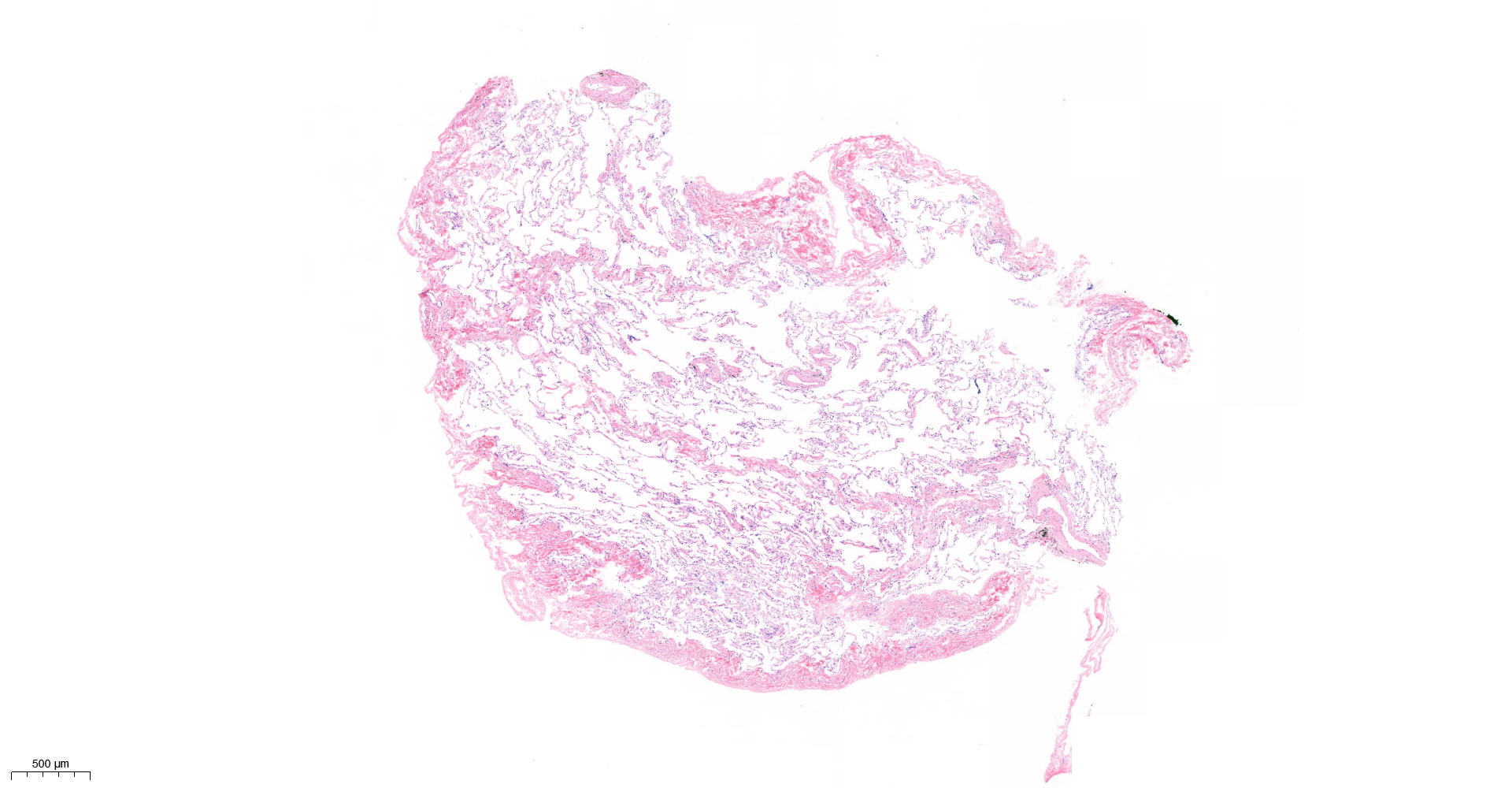


Sample 9.


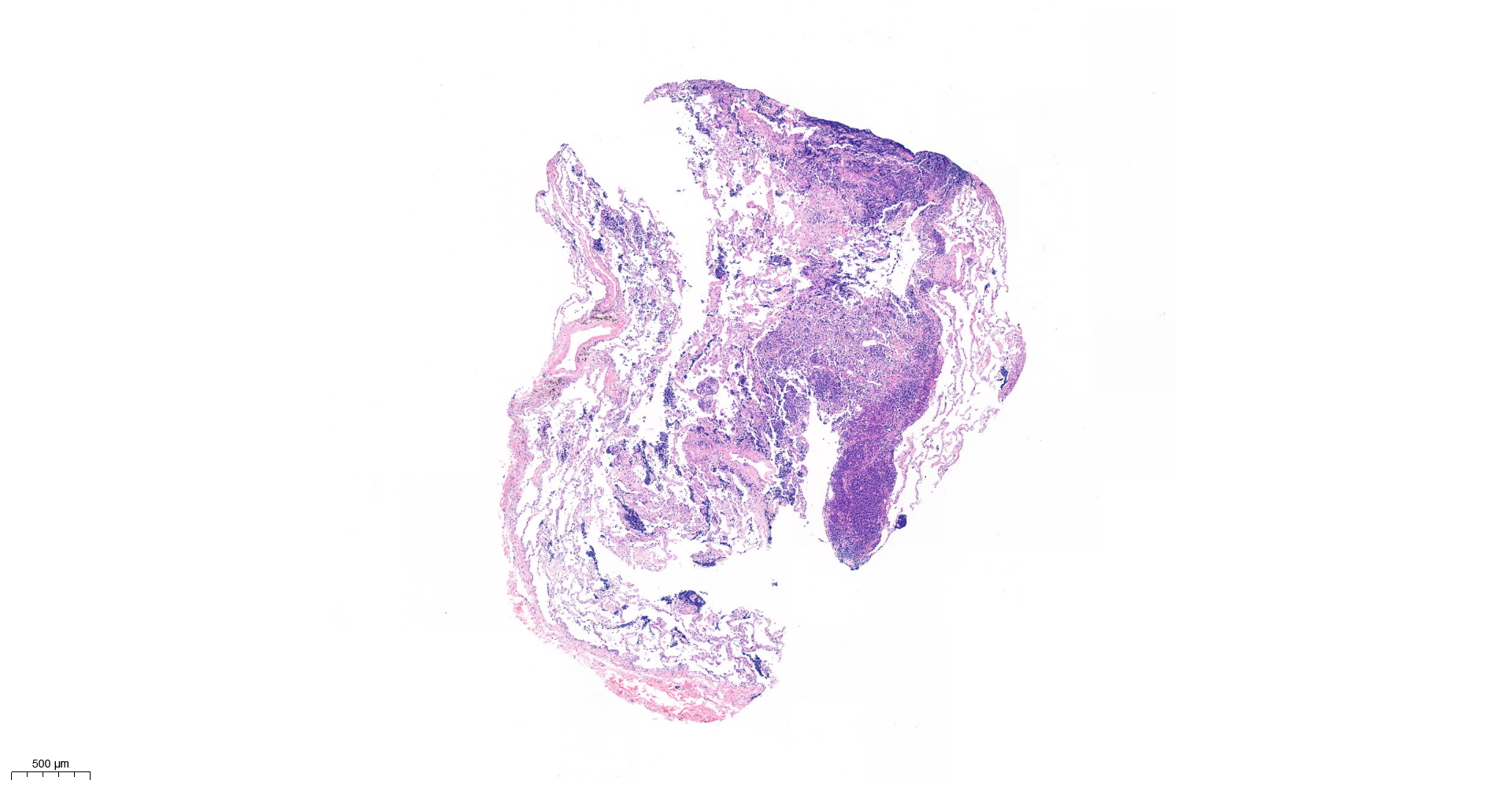


Sample 10.


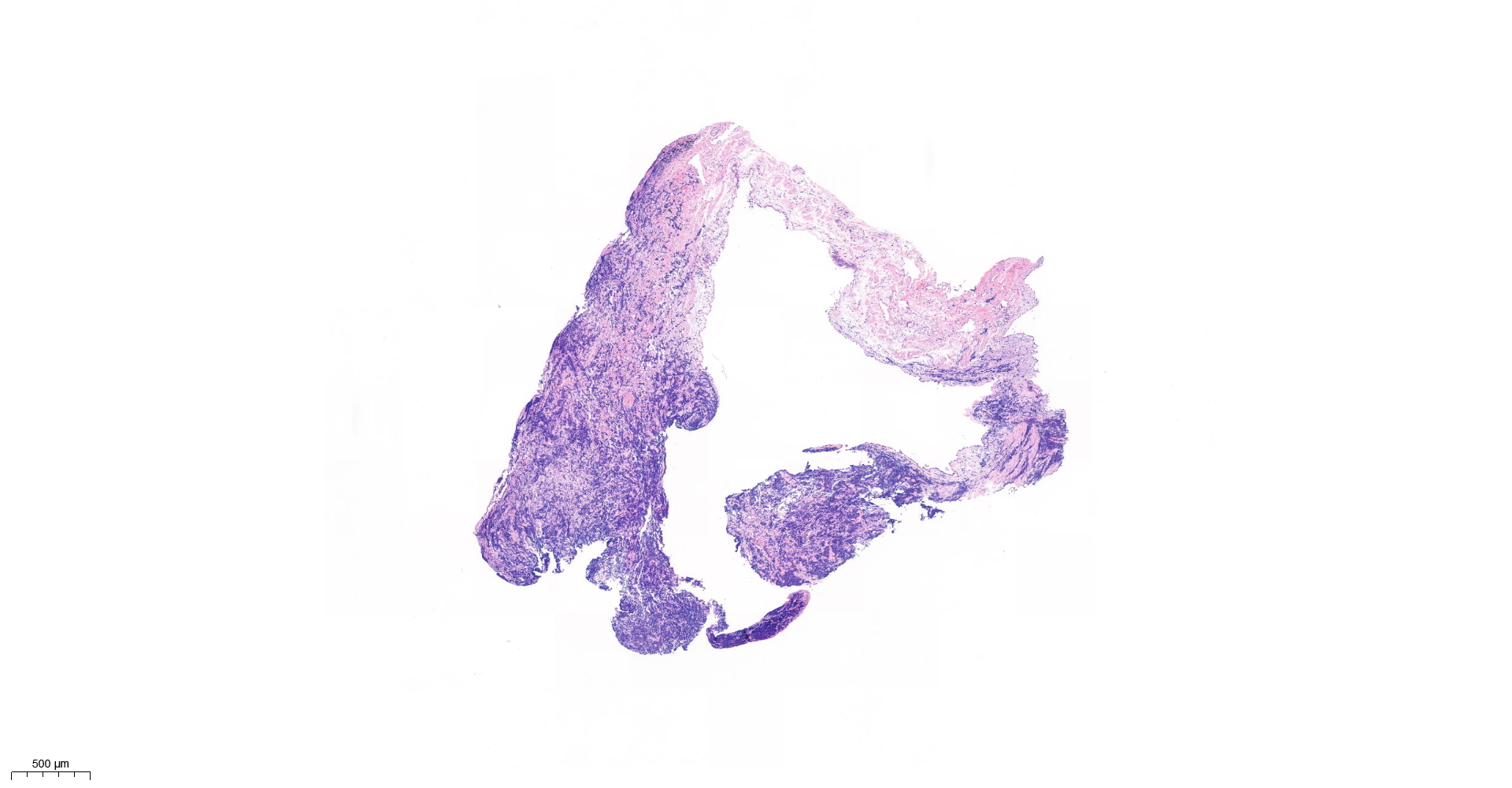


Sample 11.


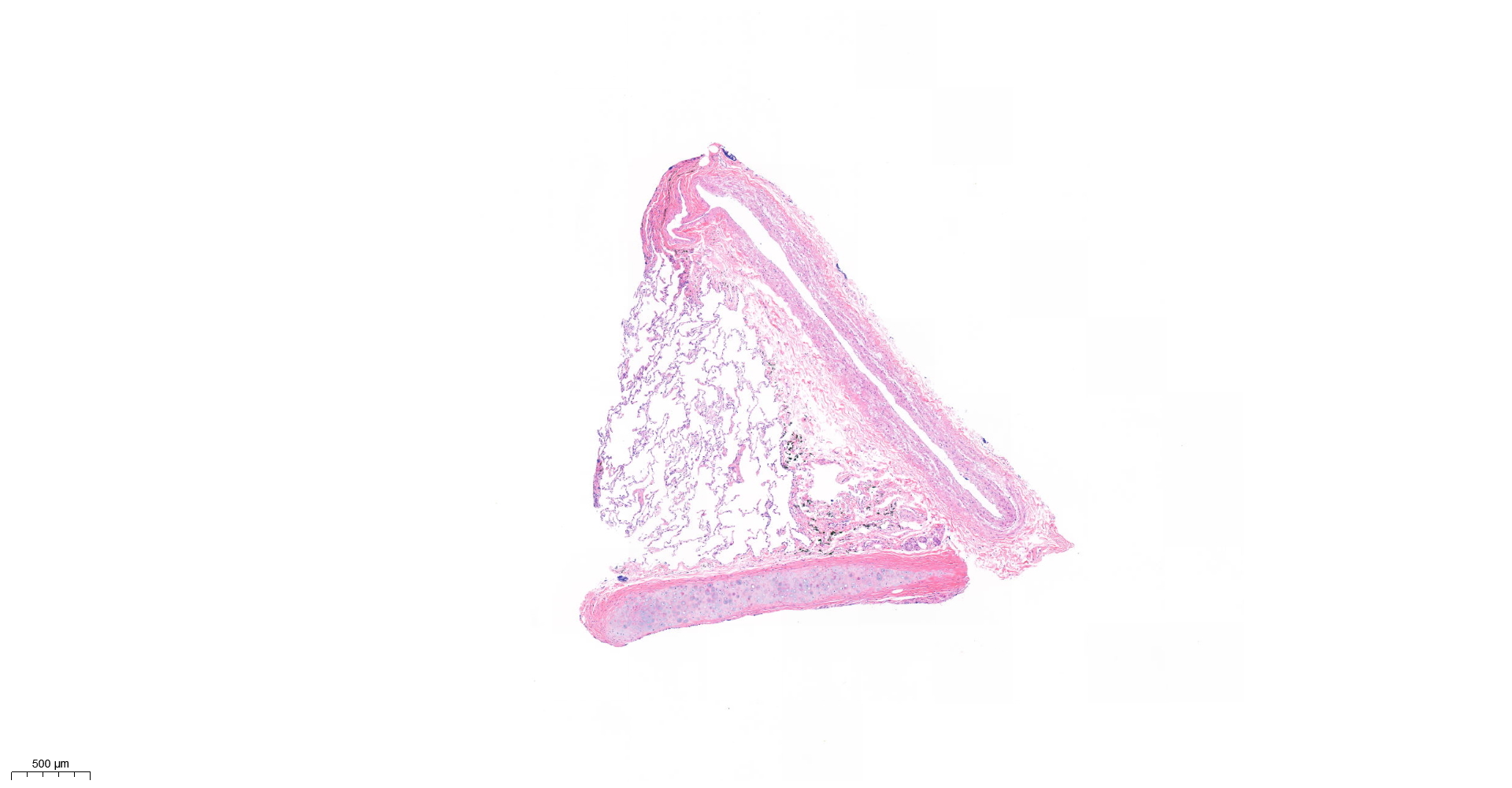


Sample 12.


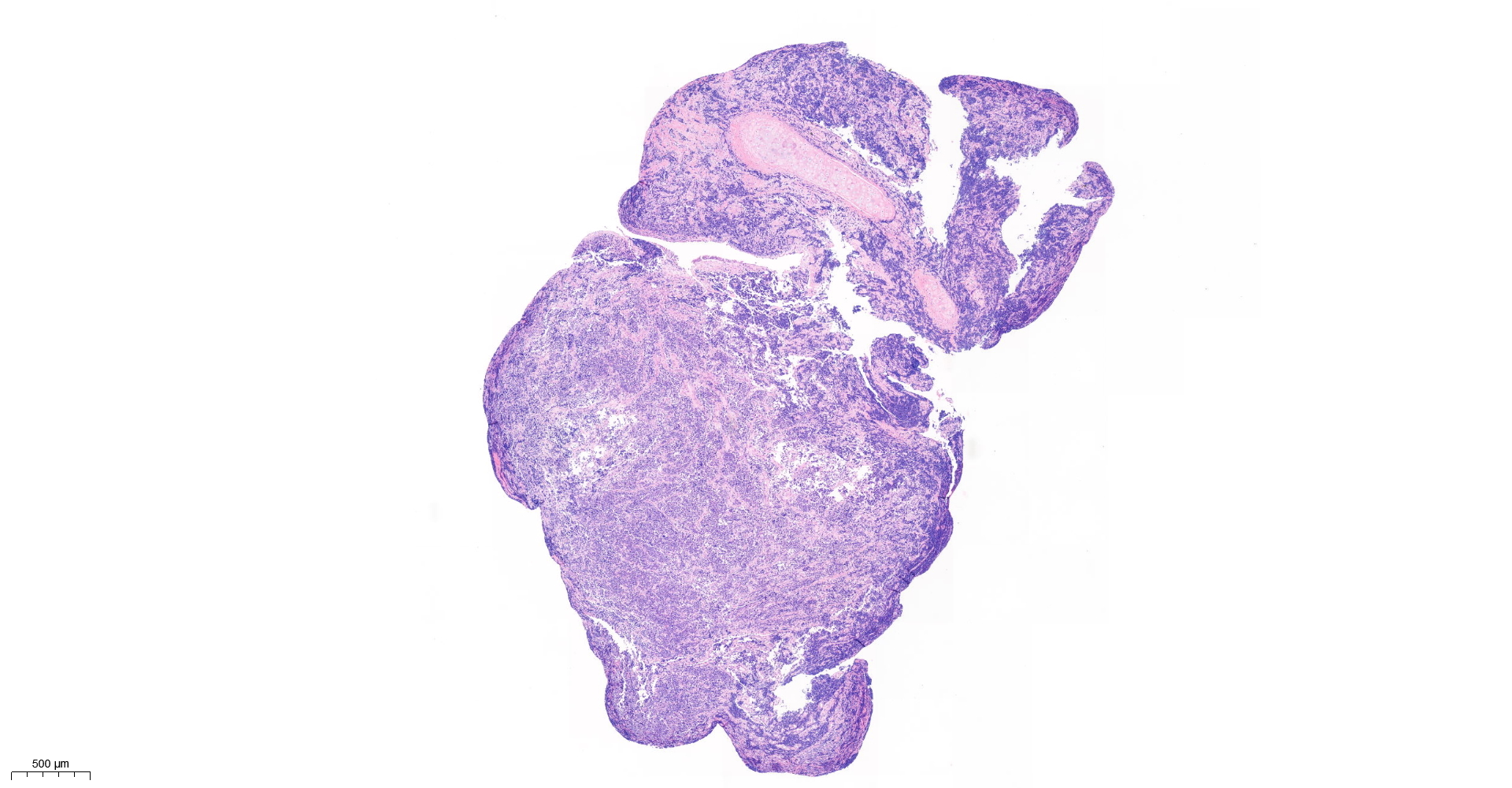


Sample 13.


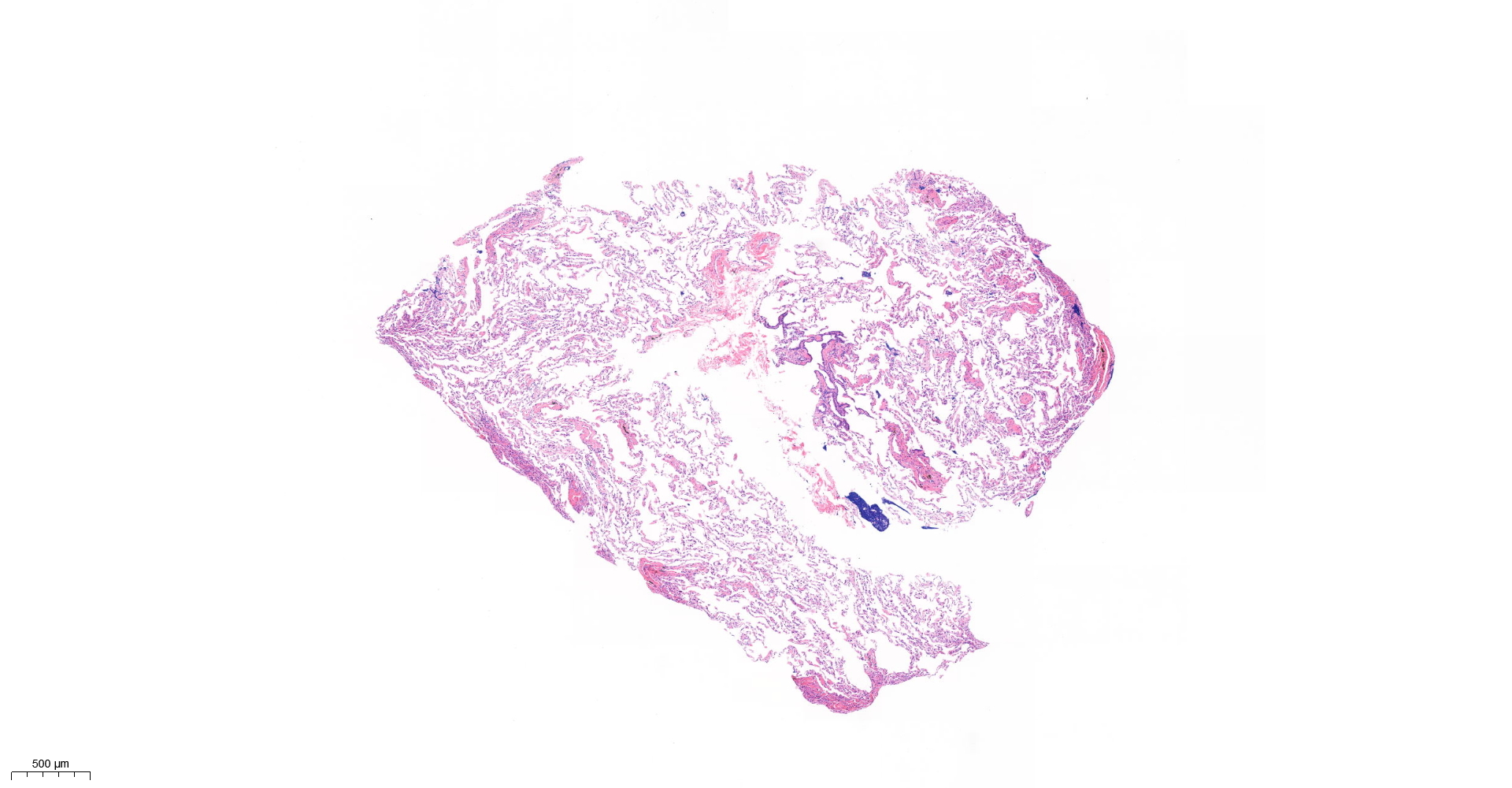


Sample 14.


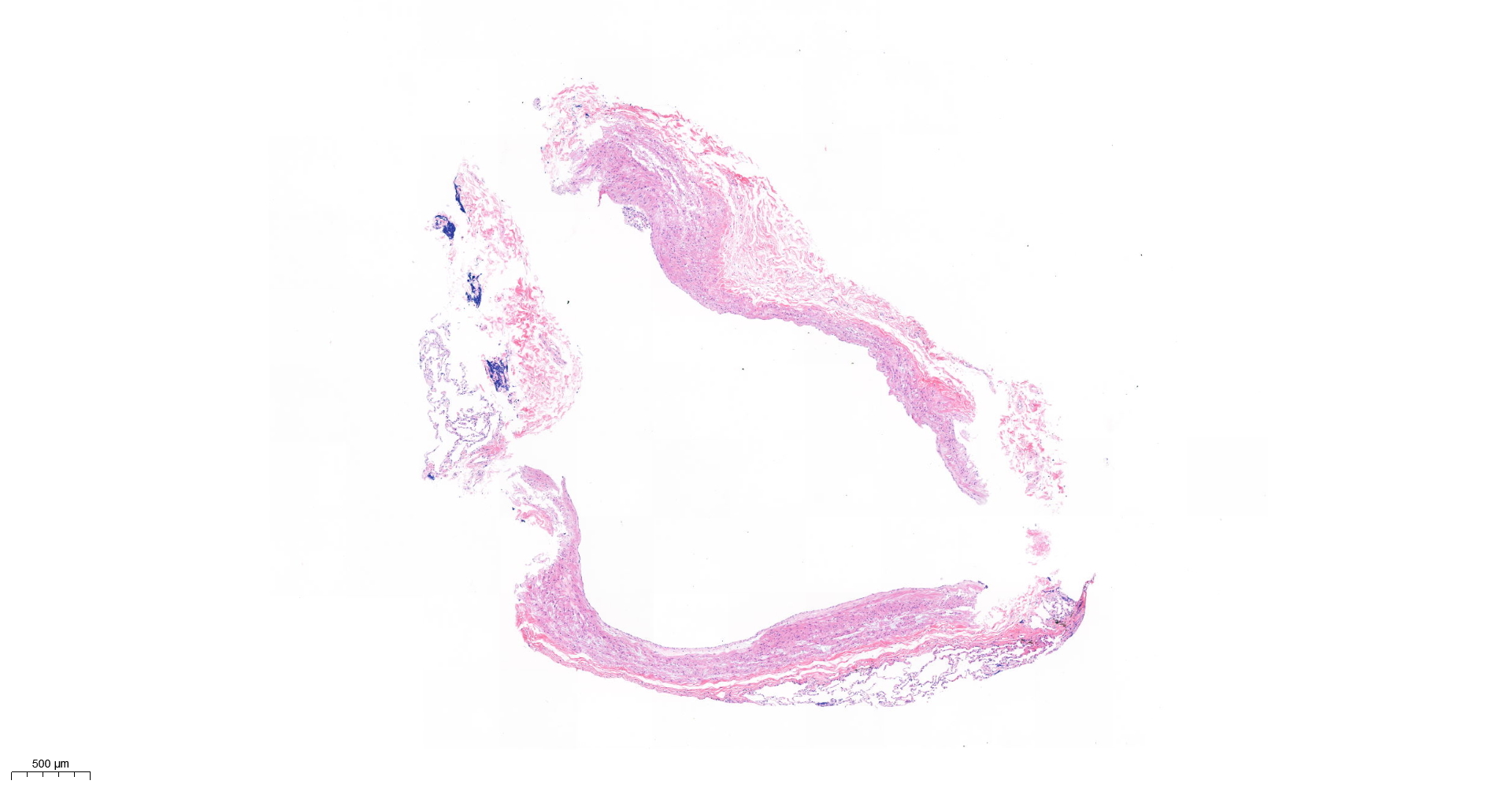


Sample 15.


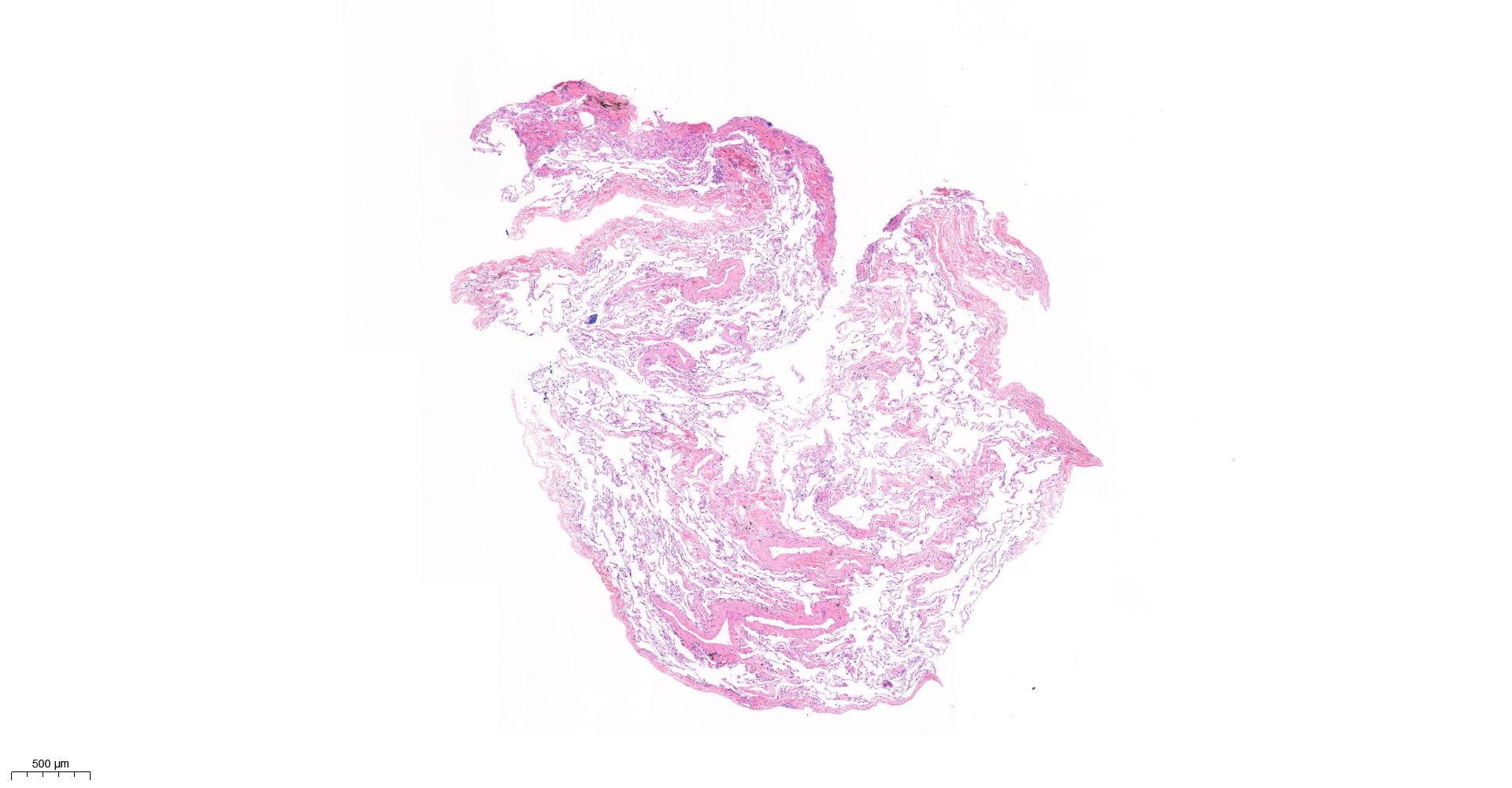


Sample 16.


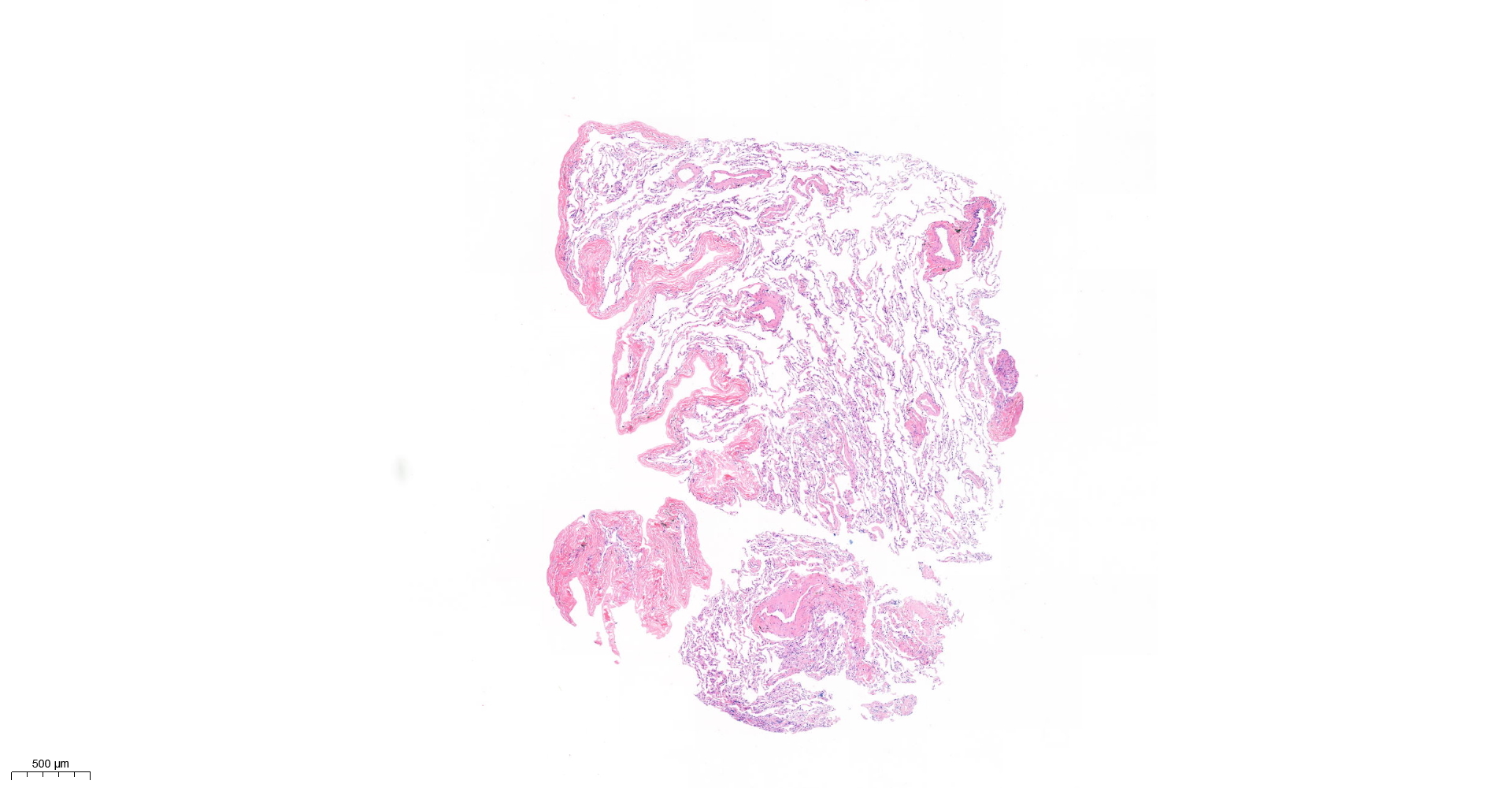

Supplement: Figure 7—source data 1. [file elife-70471-fig7-data1.zip › Figure 7-Source data/Raw data and Thumbnail images of HE staining images in Figure 7.docx]
