# Supplementary material for: A pre-screening strategy to assess resected tumor margins by imaging cytoplasmic viscosity and hypoxia
Source: eLife. 2021 Oct 11;10:e70471. doi: 10.7554/eLife.70471 (PMC8553343; doi:10.7554/eLife.70471)
Supplement: Supplementary file 1. [file elife-70471-supp1.docx]

**A Pre-Scree****ning Strategy to Assess Resected Tumor Margins by** **Imaging Cytoplasmic Viscosity and Hypoxia**

Hui Huang ^a,b^, Youpei Lin ^c^, Wenrui Ma ^d^, Jiannan Liu ^e^, Jing Han ^e^, Xiaoyi Hu ^f^, Meilin Tang ^a^, Shiqiang Yan ^a,b^, Mieradilijiang Abudupataer ^d^, Chenping Zhang ^e^, Qiang Gao ^c^, Weijia Zhang ^a,b,d,^*

^a^ Institutes of Biomedical Sciences and The State Key Laboratory of Molecular Engineering of Polymers, Fudan University, Shanghai 200032, China

^b^ Department of Systems Biology for Medicine and The Shanghai Key Laboratory of Medical Imaging Computing and Computer Assisted Intervention, Shanghai Medical College of Fudan University, Shanghai 200032, China

^c^ Department of Liver Surgery and Transplantation, Liver Cancer Institute, Zhongshan Hospital, Fudan University, Shanghai 200032, China

^d^ Department of Cardiac Surgery and Shanghai Institute of Cardiovascular Diseases, Zhongshan Hospital, Fudan University, Shanghai 200032, China

^e^ Department of Oromaxillofacial Head and Neck Oncology, Shanghai Ninth People’s Hospital, Shanghai Jiao Tong University School of Medicine, Shanghai 200011, China

^f^ Department of Urology, Zhongshan Hospital, Fudan University, Shanghai 200032, China

*Correspondence: [weijiazhang@fudan.edu.cn](mailto:weijiazhang@fudan.edu.cn) (Weijia Zhang)

**Supplementary File 1.** Clinical Characteristics of the patients

| **Cancer types** | **Hepatocellular cancer** | **Renal cancer** | **Oral cancer** | **Lung cancer** |
| --- | --- | --- | --- | --- |
| **N** | 9 | 6 | 5 | 5 |
| **Gender(%, male)** | 7 (77.8%) | 2 (33.3%) | 4 (80.0%) | 3 (60.0%) |
| **Age, average** | 49.7±9.2 | 58.0±13.3 | 67.8±7.5 | 65.8±8.1 |
